# Review of *Apanteles* *sensu stricto* (Hymenoptera, Braconidae, Microgastrinae) from Area de Conservación Guanacaste, northwestern Costa Rica, with keys to all described species from Mesoamerica

**DOI:** 10.3897/zookeys.383.6418

**Published:** 2014-02-24

**Authors:** Jose L. Fernández-Triana, James B. Whitfield, Josephine J. Rodriguez, M. Alex Smith, Daniel H. Janzen, Winnie D. Hallwachs, Mehrdad Hajibabaei, John M. Burns, M. Alma Solis, John Brown, Sophie Cardinal, Henri Goulet, Paul D. N. Hebert

**Affiliations:** 1 Department of Integrative Biology and the Biodiversity Institute of Ontario, University of Guelph, Guelph, ON N1G 2W1 Canada; 2 Canadian National Collection of Insects, 960 Carling Ave., Ottawa, ON K1A 0C6 Canada; 3 Department of Entomology, University of Illinois, Urbana, IL 61801 USA; 4 Dept. of Natural Sciences, Univerity of Virginia’s College at Wise, Wise, VA 24293 USA; 5 Department of Biology, University of Pennsylvania, Philadelphia, PA 19104-6018 USA; 6 Department of Entomology, National Museum of Natural History, Smithsonian Institution, P.O.Box37012, MRC127, Washington, DC 20013-7012 USA; 7 Systematic Entomology Laboratory, USDA, c/o National Museum of Natural History, P.O. Box 37012, Washington, DC 20013-7012, USA

**Keywords:** *Apanteles*, Microgastrinae, Braconidae, taxonomy, parasitoid biology, DNA barcoding, Lepidoptera, caterpillar rearing, Malaise traps, tropical biodiversity, Area de Conservación Guanacaste, Costa Rica, Mesoamerica, Lucid software, Hymenoptera Anatomy Ontology website

## Abstract

More than half a million specimens of wild-caught Lepidoptera caterpillars have been reared for their parasitoids, identified, and DNA barcoded over a period of 34 years (and ongoing) from Area de Conservación de Guanacaste (ACG), northwestern Costa Rica. This provides the world’s best location-based dataset for studying the taxonomy and host relationships of caterpillar parasitoids. Among Hymenoptera, Microgastrinae (Braconidae) is the most diverse and commonly encountered parasitoid subfamily, with many hundreds of species delineated to date, almost all undescribed. Here, we reassess the limits of the genus *Apanteles* *sensu stricto*, describe 186 new species from 3,200+ parasitized caterpillars of hundreds of ACG Lepidoptera species, and provide keys to all 205 described *Apanteles* from Mesoamerica – including 19 previously described species in addition to the new species. The Mesoamerican *Apanteles* are assigned to 32 species-groups, all but two of which are newly defined. Taxonomic keys are presented in two formats: traditional dichotomous print versions and links to electronic interactive versions (software Lucid 3.5). Numerous illustrations, computer-generated descriptions, distributional information, wasp biology, and DNA barcodes (where available) are presented for every species. All morphological terms are detailed and linked to the Hymenoptera Anatomy Ontology website. DNA barcodes (a standard fragment of the cytochrome *c* oxidase I (COI) mitochondrial gene), information on wasp biology (host records, solitary/gregariousness of wasp larvae), ratios of morphological features, and wasp microecological distributions were used to help clarify boundaries between morphologically cryptic species within species-complexes. Because of the high accuracy of host identification for about 80% of the wasp species studied, it was possible to analyze host relationships at a regional level. The ACG species of *Apanteles* attack mainly species of Hesperiidae, Elachistidae and Crambidae (Lepidoptera). About 90% of the wasp species with known host records seem to be monophagous or oligophagous at some level, parasitizing just one host family and commonly, just one species of caterpillar. Only 15 species (9%) parasitize species in more than one family, and some of these cases are likely to be found to be species complexes. We have used several information sources and techniques (traditional taxonomy, molecular, software-based, biology, and geography) to accelerate the process of finding and describing these new species in a hyperdiverse group such as *Apanteles*.

The following new taxonomic and nomenclatural acts are proposed. Four species previously considered to be *Apanteles* are transferred to other microgastrine genera: *Dolichogenidea hedyleptae* (Muesebeck, 1958), **comb. n.**, *Dolichogenidea politiventris* (Muesebeck, 1958), **comb. n.**, *Rhygoplitis sanctivincenti* (Ashmead, 1900), **comb. n.**, and *Illidops scutellaris* (Muesebeck, 1921), **comb. rev.** One European species that is a secondary homonym to a Mesoamerican species is removed from *Apanteles* and transferred to another genus: *Iconella albinervis* (Tobias, 1964), **stat. rev.** The name *Apanteles albinervican* Shenefelt, 1972, is an invalid replacement name for *Apanteles albinervis* (Cameron, 1904), **stat. rev.**, and thus the later name is reinstated as valid. The following 186 species, all in *Apanteles* and all authored by Fernández-Triana, are described as **species nova:** *adelinamoralesae*, *adrianachavarriae*, *adrianaguilarae*, *adrianguadamuzi*, *aichagirardae*, *aidalopezae*, *albanjimenezi*, *alejandromasisi*, *alejandromorai*, *minorcarmonai*, *alvarougaldei*, *federicomatarritai*, *anabellecordobae*, *rostermoragai*, *anamarencoae*, *anamartinesae*, *anapiedrae*, *anariasae*, *andreacalvoae*, *angelsolisi*, *arielopezi*, *bernardoespinozai*, *bernyapui*, *bettymarchenae*, *bienvenidachavarriae*, *calixtomoragai*, *carloscastilloi*, *carlosguadamuzi*, *eliethcantillanoae*, *carlosrodriguezi*, *carlosviquezi*, *carloszunigai*, *carolinacanoae*, *christianzunigai*, *cinthiabarrantesae*, *ciriloumanai*, *cristianalemani*, *cynthiacorderoae*, *deifiliadavilae*, *dickyui*, *didiguadamuzi*, *diegoalpizari*, *diegotorresi*, *diniamartinezae*, *duniagarciae*, *duvalierbricenoi*, *edgarjimenezi*, *edithlopezae*, *eduardoramirezi*, *edwinapui*, *eldarayae*, *erickduartei*, *esthercentenoae*, *eugeniaphilipsae*, *eulogiosequeira*, *felipechavarriai*, *felixcarmonai*, *fernandochavarriai*, *flormoralesae*, *franciscopizarroi*, *franciscoramirezi*, *freddyquesadai*, *freddysalazari*, *gabrielagutierrezae*, *garygibsoni*, *gerardobandoi*, *gerardosandovali*, *gladysrojasae*, *glenriverai*, *gloriasihezarae*, *guadaluperodriguezae*, *guillermopereirai*, *juanmatai*, *harryramirezi*, *hectorsolisi*, *humbertolopezi*, *inesolisae*, *irenecarrilloae*, *isaacbermudezi*, *isidrochaconi*, *isidrovillegasi*, *ivonnetranae*, *jairomoyai*, *javiercontrerasi*, *javierobandoi*, *javiersihezari*, *jesusbrenesi*, *jesusugaldei*, *jimmychevezi*, *johanvargasi*, *jorgecortesi*, *jorgehernandezi*, *josecalvoi*, *josecortesi*, *josediazi*, *josejaramilloi*, *josemonteroi*, *joseperezi*, *joserasi*, *juanapui*, *juancarrilloi*, *juangazoi*, *juanhernandezi*, *juanlopezi*, *juanvictori*, *juliodiazi*, *juniorlopezi*, *keineraragoni*, *laurahuberae*, *laurenmoralesae*, *leninguadamuzi*, *leonelgarayi*, *lilliammenae*, *lisabearssae*, *luciariosae*, *luisbrizuelai*, *luiscanalesi*, *luiscantillanoi*, *luisgarciai*, *luisgaritai*, *luishernandezi*, *luislopezi*, *luisvargasi*, *manuelarayai*, *manuelpereirai*, *manuelriosi*, *manuelzumbadoi*, *marcobustosi*, *marcogonzalezi*, *marcovenicioi*, *mariachavarriae mariaguevarae*, *marialuisariasae*, *mariamendezae*, *marianopereirai*, *mariatorrentesae*, *sigifredomarini*, *marisolarroyoae*, *marisolnavarroae*, *marvinmendozai*, *mauriciogurdiani*, *milenagutierrezae*, *monicachavarriae*, *oscarchavesi*, *osvaldoespinozai*, *pablotranai*, *pabloumanai*, *pablovasquezi*, *paulaixcamparijae*, *luzmariaromeroae*, *petronariosae*, *randallgarciai*, *randallmartinezi*, *raulacevedoi*, *raulsolorsanoi*, *wadyobandoi*, *ricardocaleroi*, *robertmontanoi*, *robertoespinozai*, *robertovargasi*, *rodrigogamezi*, *rogerblancoi*, *rolandoramosi*, *rolandovegai*, *ronaldcastroi*, *ronaldgutierrezi*, *ronaldmurilloi*, *ronaldnavarroi*, *ronaldquirosi*, *ronaldzunigai*, *rosibelelizondoae*, *ruthfrancoae*, *sergiocascantei*, *sergioriosi*, *tiboshartae*, *vannesabrenesae*, *minornavarroi*, *victorbarrantesi*, *waldymedinai*, *wilbertharayai*, *williamcamposi*, *yeissonchavesi*, *yilbertalvaradoi*, *yolandarojasae*, *hazelcambroneroae*, *zeneidabolanosae*.

## Introduction

The subfamily Microgastrinae (Hymenoptera: Braconidae) is one of the most speciose subfamilies of parasitoid wasps, with more than 2,200 described species (Yu et al. 2012) and many thousands more awaiting description (Mason 1981; Rodriguez et al. 2012). Microgastrine wasps are significant in biological control because they attack the larvae of most families of Lepidoptera (Whitfield 1995, 1997).

The genus *Apanteles* was erected by Förster (1862) to include all species of microgastrines lacking a second submarginal cell in the fore wing (from the Greek: *A* – without, *panteles* – complete, entire; referring to the “incomplete” venation, i.e., missing cell, when compared with the other genera of Microgastrinae known at the time). As the study of *Apanteles* progressed, it became evident that it included a huge number of species, and many attempts to subdivide the genus have been made since 1880; there are summarized in Mason (1981) and Whitfield et al. (2002). During the last 150 years more than two dozen new genera have been created as a result of those splitting efforts, but still more than one thousand described species belong to *Apanteles* (Yu et al. 2012), and thousands more await discovery. It is worth mention that many of these species still belong to *Apanteles* *sensu lato*, and have not yet been assigned to currently recognised genera (sensu Mason 1981).

Area de Conservación Guanacaste (ACG) is a single decentralized unit of Costa Rica’s Ministerio del Ambiente, Energia (MINAE; Ministry of Environment and Energy) covering about 2% of Costa Rica in its northwestern corner, slightly south of the southeastern border of Nicaragua (http://www.acguanacaste.ac.cr). Comprising ~1,200 km^2^ of terrestrial habitat (centered at 10.8 latitude, -85.6 longitude), it is a swath from Pacific coastal mangroves across lowland dry forest (dry season deciduous), up the slopes of three volcanoes to cloud forest (1400–2000 m), and down into Caribbean lowland (90 m) rain forest. It is only 85 km from east to west, yet contains portions of eight Holdridge Life Zones within mosaics of them, some as small as 5 km in linear dimensions and 20 km^2^. Nearly all of the ACG lowlands have been subjected to four centuries of light to intense cultivation, logging, burning, hunting, ranching, and other forms of habitat destruction, followed by explicit protection and restoration beginning in 1971 and intensifying after 1985 (Janzen 1988, 2000, 2002). The outcome is a mosaic of all imaginable ages and kinds of secondary succession intermingled with tiny to medium-sized fragments of approximations of intact forest (more intact in upper elevations than lower), as well as severe blurring and elimination of interdigitated boundaries between habitats and ecosystems (Janzen 1986-1988). All of the ACG region has also now experienced at least two decades of notable drying and increasing weather unpredictability, rendering it yet more difficult to know if the marked annual and decadal population changes are being generated by climate changes, successional changes, insularization of the ACG ecological island in the agroscape, species-by-species biological serendipity, and/or interactions among all of these (Janzen et al. 2011).

ACG has been the focus of 34+ years of inventory of wild-caught caterpillars, their food plants and their parasitoids, as described in detail in Janzen et al. (2009) and Janzen and Hallwachs (2011), and available in a rearing-by-rearing specimen-based public database at Janzen and Hallwachs (2013). The ACG is currently staffed and supported by about 180 Costa Ricans, nearly all of whom are honoured with patronynms in this paper.

Haphazardly placed Townes Malaise traps in all three major ACG terrestrial ecosystems have yielded another set of ACG *Apanteles* species, many of which have not yet been reared and are included here (and are so indicated as distinct from the species that have been reared, many of which have not yet been encountered by Malaise-trapping).

The rearing results have been complemented since 2003 by extensive DNA barcoding of one or more voucher specimens from each rearing, past and present (Janzen and Hallwachs 2011). This has provided an additional layer of data to study the ACG species of caterpillars, parasitoids, and food plants (e.g., Smith et al. 2006, 2007, 2008; Whitfield et al. 2012; Janzen et al. 2011, 2012).

DNA barcoding uses a short standardized region of the mitochondrial gene cytochrome *c* oxidase (COI) as a key character for species-level identification and discovery (Floyd et al. 2002, Hebert et al. 2003a and b, Janzen et al. 2009, Smith et al. 2006, 2007, 2008). Interspecific barcode variation can be used as part of a suite of characters for the discovery and description of new species (e.g., Hebert et al. 2004, Burns et al. 2008, Fisher and Smith 2008, Fernández-Triana 2010), and can speed the rate of taxonomic research by flagging otherwise cryptic diversity (e.g., Janzen et al. 2009, Fisher and Smith 2008, Smith and Fisher 2009, Smith et al. 2008). DNA barcoding has been extensively used in biodiversity and taxonomic studies of Microgastrinae during the past five years (e.g., Smith et al. 2008 and 2013, Janzen et al. 2009, Fernández-Triana 2010, Fernández-Triana et al. 2011, Rodriguez et al. 2012, Whitfield et al. 2012, Fernández-Triana et al. 2013).

Taxonomic studies of ACG Microgastrinae have been published elsewhere (e.g., Valerio et al. 2005, Grinter et al. 2009, Smith et al. 2008, Valerio et al. 2009, Janzen and Hallwachs 2011, Janzen et al. 2009, Whitfield et al. 2012, Arias-Penna et al. 2013, Fernández-Triana et al. 2013). However, the ACG species of *Apanteles* *sensu stricto* have never been treated in a taxonomic review.

The combination of this comprehensive inventory with the richness of biological, ecological and DNA barcoding data, allowed us to engage in the taxonomic study of ACG *Apanteles* as a whole, and within the context of the other hundreds of species of ACG Microgastrinae. In doing so, we also revised all 19 of the previously described *Apanteles* *sensu stricto* known from Mesoamerica and incorporate them here. However, no effort was made to study specimens representing undescribed species from areas outside ACG, areas that will certainly contain hundreds of other species of *Apanteles* as well as many of those in ACG. We hope that this study will be a foundation upon which future studies of tropical *Apanteles* and other microgastrine genera can be based.

## Methods

In this study, Mesoamerica is defined as the region from (and including) Mexico through Panama, and all the Caribbean islands, following Gauld (1988).

We studied 4,100+ specimens from 3,200+ individual caterpillar rearings, and 2,000+ DNA sequences (usually one sequence per rearing event) of *Apanteles* from ACG. Ecological, biological and distribution data for all of these records can be accessed at http://janzen.sas.upenn.edu/caterpillars/database.lasso (Janzen and Hallwachs 2013) by searching on the “DHJPARxxxxxxx” voucher code of the wasp, or the “yy-SRNP-xxxxx” voucher codes of the caterpillar. If a DHJPARxxxxxxx voucher code is cited, it is for a single specimen. If a yy-SRNP-xxxxx voucher code is cited, it is for 1 to *N* specimens reared from a single caterpillar and which are presumed siblings, but have not been individually vouchered, whether point-mounted or remaining preserved in ethanol. All holotypes bear a DHJPARxxxxxxx unique voucher code (and if there was more than one specimen in that rearing from that one caterpillar, all of them will bear the same yy-SRNP-xxxxx code). In this paper we refer to these voucher codes as “ACG database codes” when providing specimen details in the taxonomic treatment of species. In the case that a set of specimens reared from one individual caterpillar was not DNA barcoded, the vial containing those specimens has only the yy-SRNP-xxxxx code, while an individual wasp that has been barcoded from that sample bears both the SRNP code and the DHJPAR code. Each barcoded specimen also has an accession code from the Barcode of Life Data System (BOLD) and GenBank.

Type material for most of the 19 previously described Mesoamerican species was borrowed for study. However, no molecular data is available for any of those holotypes. It will not be surprising if some of their names are found to encompass complexes of species. Some members of such complexes may be some of the ACG species described here, but it would be premature to even speculate about that.

The following acronyms are used:

BMNH The Natural History Museum, London, United Kingdom

CNC Canadian National Collection of Insects, Arachnids and Nematodes, Ottawa, Canada

INHS Illinois Natural History Survey, Champaign, Illinois, United States

INBio Instituto Nacional de Biodiversidad, Santo Domingo de Heredia, Costa Rica

NMNH National Museum of Natural History, the Smithsonian Institution, Washington DC, United States

Morphological terms and measurements of structures are mostly as used by Mason (1981), Huber and Sharkey (1993), Sharkey and Wharton (1997), Whitfield (1997), and Valerio et al. (2009). However, we also incorporated a recent, comprehensive morphological treatment of Opiinae (Braconidae) by Karlsson and Ronquist (2012), which is part of a wider effort to standardize and homologize morphological terms and definitions across the order Hymenoptera, the Hymenoptera Anatomy Ontology (HAO) project (Yoder et al. 2010, Seltmann et al. 2012). As a result of adopting most HAO preferred terms (but see exceptions below), some of the morphological terms we apply have never been used in taxonomic papers treating Microgastrinae.

Karlsson and Ronquist (2012) named and numbered the first metasomal segment as “abdominal tergum/sternum 2”. Usually, that segment has been called (and numbered) “metasomal tergum/sternum 1” (Mason 1981, Whitfield 1997, 2006). Though both approaches are correct, we use “metasomal tergum/sternum 1” because we consider it is clearer and facilitates the counting of metasomal segments (as in Fig. 207). The same applies to its associate sclerites (mediotergites and laterotergites).

We considered that the “preferred label” (i.e., name) provided in the HAO website for “mesoscutellar arm” was better than the corresponding term, “posterior bar of mesoscutellum”, used by Karlsson and Ronquist (2012).

The terms “mesoscutellar trough” and “mesoscutellar arm” (Fig. 206) have been used extensively in taxonomy of Microgastrinae (e.g., Mason 1981, and many subsequent papers), usually under the name of “lateral face of scutellum”. Karlsson and Ronquist (2012) did not provide much detail for those areas because the two species of Opiinae they studied are relatively simple and non-differentiated in that body region.

We also calculated and compared many ratios between linear dimensions of structures (morphometric taxonomy), a common practice in the taxonomy of many groups of parasitoid wasps (Baur and Leuenberger 2011, and references cited there). However, most of the ratios presented here have not been used previously in Microgastrinae taxonomy.

To facilitate understanding of the traits and ratios, a detailed account of every morphological structure and measurement used in this study is provided in Appendix 1, including links to the HAO website and references to terms that have been commonly used previously in Microgastrinae taxonomy. The most important morphological characters used in this study are illustrated in Figs 206–209.

Throughout the text, especially in the keys, “body length” refers to the length of the anatomical line that is median and extends between the anteriormost point of the head and the posteriormost point of the metasoma (excluding ovipositor and ovipositor sheaths). “Fore wing length” refers to the length of the anatomical line that extends between the median margin of the first axillary sclerite and the distalmost point of the wing blade (Appendix 1).

The measurement of variables must be done as uniformly as possible, and special care must be taken when choosing the end points of any structure. It is also advisable to measure at the highest possible magnification to minimize errors. Some measurements that are particularly error-prone are discussed further in Appendix 1.

Throughout the keys the following acronyms are used for morphological terms: T1, T2, T3 (mediotergite 1, 2, 3). Whenever there is a “(N = a number)”, e.g., “(N = 4)” after a species name, it refers to the number of specimens studied morphologically for that species. It is only provided when the available number of specimens was less than 5.

Molecular analysis has revealed a large number of morphologically cryptic species, often possessing very subtle morphological differences that we found to correlate with ecological and host data. Certain features differ just slightly between species, and there may be overlap of values between individual specimens of different but very similar species. We studied as many specimens as were available. Our definition of a “species” is a postulated biological unit that differs from other species in its morphology (however subtle), COI barcode, and host use, and presumably represents a distinct breeding population. In the few cases where what we consider to be a species differs only in barcode and/or host, we indicate this. All the species in ACG are essentially fully sympatric to parapatric (the case when two ecosystems intergrade).

The dichotomous keys were built to accommodate, as much as possible, what appear to be potential natural groups, based on morphology, biology (host data), and DNA barcoding. However, in such a large assemblage of species there is likely to be considerable homoplasy and thus in some couplets we had to use logical characters (e.g., “***if***”, “***then***”, “***and***”, “***or***”, “***and/or***”). Those words are shown in bold and italic throughout the keys, to be explicit that in those cases more than one character system has to be considered.

The species descriptions are based on the holotype female, and we consider their DNA barcodes to be definitive when available (actual barcodes are available through the BOLD web site at http://www.boldsystems.org); when other specimens are available, their data are included to provide some idea of intraspecific variation. When the holotype was not examined or was lacking some body parts (some old holotypes), other specimens were used to complete the descriptions, and details were explained in the “Comments” section.

Males of Microgastrinae are difficult to key out under the present knowledge of the subfamily (Whitfield 1997, 2006), and may be difficult or impossible to identify unless associated with sibling females from the same host caterpillar, DNA barcodes, or host data. Thus, the keys are only intended for female specimens and use many characters only found in that sex (e.g., length of ovipositor sheaths).

Non-morphological characters are also provided whenever available, e.g., host species, whether there are one or many larvae per host caterpillar, microhabitat, microgeographical distribution, and molecular differences in the DNA barcode region, that may serve as diagnostic characters. Sometimes those features are included in brackets at the end of the corresponding couplet, intended as supplementary information that can help the user to correctly identify specimens. They are best not separated from the morphological features provided. However, in future, practical and routine identification may often depend heavily on these other traits because they are easier to assess than the morphology of wasps 1–5 mm in length.

Lucid 3.5.4 (http://www.lucidcentral.com/) software was used to automatically generate descriptions of the species and to prepare Lucid identification keys. A dataset of 41 characters and 239 character-states was used to provide uniform description formats for all species treated (except for the *leucostigmus* species-group, see next paragraph). The description format includes one sentence per character, with the character mentioned first and the character-state following after a colon, e.g., “Tarsal claws: simple”. Whenever a species scored more than one character-state, the description included all of the pertaining character-states separated by “or”, e.g., “Tarsal claws: simple or simple with single basal spine-like seta”. Whenever a character-state was coded as uncertain due to poor condition of the specimen(s), the description includes the details of the character-state as best assessed, followed by a question mark, e.g., “Tarsal claws: simple (?)”. Sometimes a character could not be coded due to missing body parts in the available specimens; in such instances the feature was left out of the description for that particular species.

The *leucostigmus* species-group was found to be exclusively composed of morphologically cryptic species (e.g., Smith et al. 2008). In this group we only provide “complete” classical descriptions (i.e., similar to other groups) for the two species previously described, *Apanteles albinervis* (Cameron, 1904) and *Apanteles leucostigmus* (Ashmead, 1900). Another 37 new species found to belong to this group are only described using a simplified set of 10 characters and 46 characters-states, and are most definitively defined by the combination of their barcodes and their hosts.

Accurate color description is a major difficulty in taxonomic works (Aguiar 2005), and often its variation within and between species can lead to confusion. In Microgastrinae, as in many other groups, the color pattern of body regions seems to be more important and taxonomically informative than the definition of the color *per se*. Accordingly, in the Lucid dataset, we used a simplified convention to code color, considering it as either pale (white, light yellow, orange-yellow, light brown-yellow) or dark (dark brown, black). For details on the exact color patterns on the body, we provide extensive illustrations for every species. When describing leg color (especially metacoxa, metafemur, and metatibia), we are referring to the outer side of the leg, e.g., *sensu* figure 1 of Goulet and Huber (1993).

Most of the photos were taken with a Canon EOS 60D with MPE-65 lenses (aperture: 4.0, ISO: 100, CR2 format images), and a 600EX-RT Speedlight (manual) flash. The camera was mounted on a Kaiser copy stand with a Z-stepper (Stackshot) to allow for taking of multiple images. A dozen species were photographed with a Keyence VHX-1000 Digital Microscope, using a lens with a range of 13–130 ×.

Multiple images through the focal plane were taken of a structure and these were combined to produce a single in-focus image. For the pictures taken with the Canon camera, the Zerene Stacker program (http://zerenesystems.com/cms/stacker) was used; the software associated with the Keyence System produced the focused images taken with that camera. Plates for the illustrations were prepared using Adobe Photoshop CS4.

Although the keys provided in this paper are based on morphological characters, in a few cases (mainly with species belonging to the *leucostigmus* group) we used molecular characters to differentiate species that are morphologically exceptionally similar to each other. In those cases we used characteristic loci in the DNA barcoding region in identification couplets. The bases are numbered from the start of the COI gene according to the reference sequence U37541 (*Drosophila melanogaster*). The bases noted are only diagnostic within the couplet and beneath that described split. A hypothetical example of the format used is: “A total of 11 diagnostic characters in the barcoding region: 12 A, 18 C, 22 A, 23 T, 44 C, 56 G, 120 G, 340 A, 488 T, 502 T, 601 A”. The letters A, C, G, and T correspond to adenine, cytosine, guanine, and thymine respectively.

DNA barcodes for all ACG inventory Microgastrinae (Appendix 2) were obtained using DNA extracts prepared from single legs using a glass fibre protocol (Ivanova et al. 2006). Extracts were re-suspended in 30 μl of dH2O, and a 658-bp region near the 5’ terminus of the COI gene was amplified using standard primers (LepF1–LepR1) following established protocols (Smith et al. 2006, 2007, 2008). If the initial 658 bp amplification was not successful, composite sequences were generated using internal primers. Primer information for individual sequences can be retrieved from the Barcode of Life Data System (BOLD) (Ratnasingham and Hebert 2007), but primers are as detailed in Smith et al. (2008). DNA barcoding data and related information for all specimens studied in this paper can be accessed at: http://dx.doi.org/10.5883/DATASET-ACGAP1.

Sequences were considered as “barcode-compliant” when they had 500 or more base pairs and correctly placed the species with its conspecifics in an NJ tree, the function of which is simple identification (as well as species discovery). We did not follow the more formal, but less pragmatically useful, definition of also having less than 1% ambiguous characters (Barcode Compliance standards as in http://www.boldsystems.org/index.php/resources/handbook?chapter=6_managingdata.html&section=record_list), though in selecting the holotype we strive to choose a specimen with a “full” and “complete” DNA barcode. The number of sequences available in BOLD for each species that we treat is provided in the “Molecular data” section within the taxonomic treatment of every species and all sequences can be obtained from BOLD.

In the taxonomic treatment of species, “Material Examined” presents the specimen’s information in the following format: “Number of females/males, acronym of the storing institution between parenthesis, COUNTRY: State/Province, city, other locality details, coordinates (in Decimal Degrees), date, collector name, biological information on host (starting with “ex”), ACG database codes (in the format “yy-SRNP-xxxxxx” for the host or “DHJPARxxxxxxx” for the wasp)”. For states of the United States and for Canadian provinces/territories, acronyms consisting of two capital letters are used, following Canada Post (http://www.canadapost.ca/tools/pg/manual/PGaddress-e.asp).

**Figure 1.**
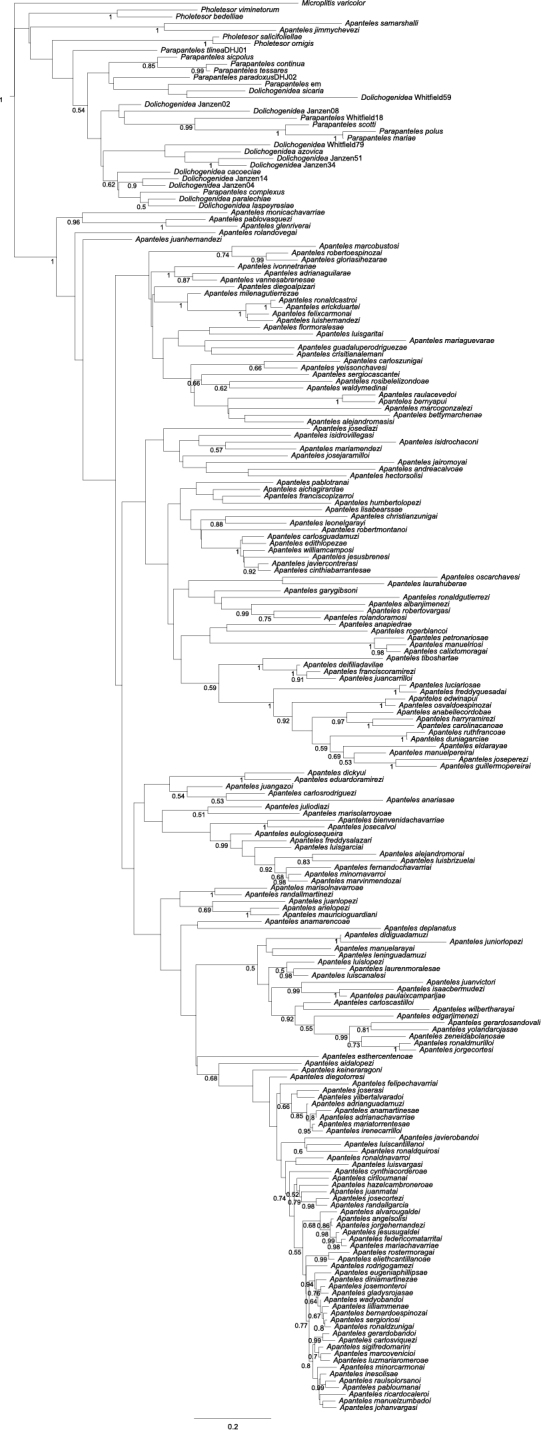
Bayesian Maximum Clade Credibility Tree (MCCT) for 180 species of Mesoamerican *Apanteles* with over 500 bp in the barcoding region and 29 species from other genera used as outgroups. Posterior probabilities over 0.50 are shown on the left side of nodes. Scale bar indicates branch length, expressed as the expected number of substitutions per site.

For phylogenetic analyses conducted outside of BOLD, DNA barcode sequences from 180 Mesoamerican *Apanteles* species were downloaded from BOLD along with 29 sequences from species of other Microgastrinae genera (*Microplitis*, *Pholetesor*, *Parapanteles*, *Dolichogenidea*). All sequences were imported into Geneious Pro 6.1 (Drummond et al. 2011) and aligned using the default settings for MUSCLE. The first and last nucleotide positions of the aligned dataset were deleted to reduce the amount of missing data. All sequences downloaded were over 500 bp long and the final aligned dataset contained 657 characters of which 315 were parsimony informative. The dataset was partitioned into two partitions, the first containing first and second codon positions and the second containing third codon positions. Model testing done in JModelTest v0.1.1 (Posada 2008) using the Bayesian Information Criterion selected the GTR+I+G model for both partitions. Two independent Bayesian analyses with 4 chains each were run in MrBayes v3.2.1 (Ronquist and Huelsenbeck 2003) for 31 253 000 generations each. Trace files of all parameters were examined in Tracer v1.5 (Rambaut and Drummond 2009) to verify that the runs had converged on the same stationary distribution, and to select the percentage of samples to remove as burn-in. A 10% burn-in was removed from both tree files which were then combined and resampled at 10%. A Maximum Clade Credibility Tree (MCCT) was made from the combined resampled tree file in TreeAnotator v1.7.5 (Rambaut and Drummond 2013). The MCCT only shows posterior probability values over 0.50 (Fig. 1). When discussing the support to particular clades (species-groups), those values are mentioned within the text in the format: “PP: 0.95” (i.e., posterior probability of 0.95). While this tree is obviously better than a simple NJ tree for displaying presumed phylogenetic relationships, we stress that the purpose of this paper is alpha taxonomy and not phylogeny, and any phylogenetic analyses are highly preliminary until hundreds more of the ACG and Mesoamerican species of *Apanteles* can be included.

**Figure 2.**
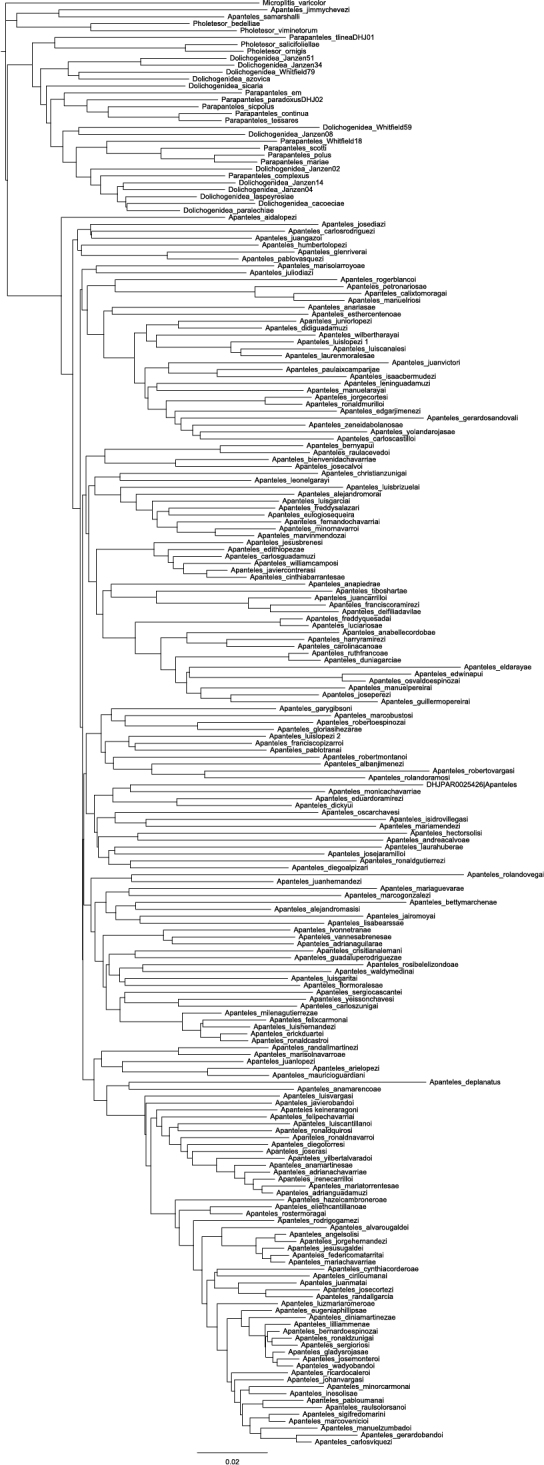
Neighbor-joining Tree for 180 species of Mesoamerican *Apanteles* with over 500 bp in the barcoding region and 29 species from other genera used as outgroups. Scale bar indicates branch length, expressed as the expected number of substitutions per site.

A neighbor-joining (Saitou and Nei 1987) tree was also constructed in Geneious Pro 6.1 (Drummond et al. 2011) using the TN93 model (Tamura and Nei 1993) (Fig. 2).

## Results

### Definition of the genus *Apanteles* *sensu stricto*

The limits of *Apanteles* and other genera of Microgastrinae have lately been the subject of debate (e.g., van Achterberg 2003), and some recent references use a rather broad generic concept, e.g., Fauna Europaea (van Achterberg 2012), and Taxapad (Yu et al. 2012). We follow here the generic concepts proposed by Mason (1981), which have been widely adopted in parts of Europe (Papp 1988, Broad et al. 2012), Australia (Austin and Dangerfield 1992), China (Chen and Song 2004), and the New World (Whitfield 1995 and 1997, Fernández-Triana 2010, Fernández-Triana et al. 2013). For the purpose of this review, we consider the genera *Choeras*, *Dolichogenidea*, *Exoryza*, *Iconella*, and *Illidops* to be valid and not synonyms or subgenera of *Apanteles* *sensu stricto.* We provide below definitions of all of these genera, and our reasons for keeping them separate from *Apanteles* *sensu stricto.*

*Choeras* was defined by Mason (1981) to include species with a median carina on the propodeum (the Greek name of the genus precisely refers to that character). In addition, many species, although not all, have a more or less complete areolet (i.e. second submarginal cell) on the fore wing. The genus is far from being resolved, as Mason himself stated in the original description. Based on extensive material worldwide that we have been able to study, some species groups (e.g. the *psarae* group, as defined by Nixon (1965) and followed by Mason (1981), and many undescribed species) currently thought to be *Choeras* may be better placed in a different (new) genus; this applies especially to most of the species from the Oriental and Australasian regions. However, all of those species have a more or less complete median carina on the propodeum, and never have any indication of a propodeal areola (in contrast to *Apanteles* *sensu stricto*). DNA barcoding tends to clearly cluster the species of both genera separately (e.g. Smith et al. 2013).

*Dolichogenidea*, described by Viereck (1911), is perhaps the closest genus to *Apanteles* *sensu stricto*, and also the most controversial and difficult to separate from *Apanteles*. Mason (1981: 53–54) devoted several pages to discussing the main defining characters and the difficulties in separating both genera; he concluded, rather hopelessly, that in practice the decision was still somewhat arbitrary (because of the apparent continuum of variation in character). We have examined thousands of specimens from the world fauna of Microgastrinae, and have found that the only reliable character is the number and density of setae fringing on the median portion of the vannal lobe. *Dolichogenidea* has a convex to almost straight vannal lobe, which is uniformly fringed by setae. In *Apanteles* the vannal lobe is strongly concave to almost straight, and is lacking setae at midlength; the lack of setae may be partial (i.e. there may be some small and sparse setae on the lobe) or total (i.e. no setae at all). The differences between vannal lobes of those two genera were illustrated by Whitfield (1997: 364, figures 92–94). Both Mason (1981) and Whitfield (1997) discussed other characters that work in some (but not all) cases. Apart from morphology, DNA barcoding tends to clearly cluster the species of both genera separately (e.g. Fernández-Triana 2010; Smith et al. 2013). Some differences in host ranges and geographical distribution have also been observed, but no comprehensive revision of the data is available yet.

The genus *Exoryza* was described by Mason (1981) to include two species. It was considered to be mainly distributed in temperate regions, although two additional species recently described, one from the Neotropics (Valerio et al. 2004) and one from China (Song and Chen 2003) suggest that the genus is more widely distributed. Valerio et al. (2004) considered it to be closely related to both *Dolichogenidea* and *Apanteles* (and in a lesser extent also to *Parapanteles* and *Pholetesor*). While its generic position is still unclear, the presence of a convex, straight vannal lobe, uniformly fringed by setae (similar to that of *Dolichogenidea*) is the main feature that distinguishes this genus from *Apanteles*.

Mason (1981) described *Iconella* as a new genus based on the sinuous vein cu-a in the hind wing as a plesiomorphic character that suggests its unique status among similar genera. Besides that, Fernández-Triana et al. (2013) also considered the presence of a median longitudinal carina on the propodeum (or the secondary loss of that carina, which occurs in some Palaeartic species but not in the New World species) as a strong support for its generic status. DNA barcoding tends to clearly cluster the species of both *Iconella* and *Apanteles* separately (e.g. Fernández-Triana et al. 2013; Smith et al. 2013).

Mason (1981) erected *Illidops* to accommodate a group of species with the lower margin of the eyes converging and metasomal terga 3–7 weakly sclerotized. However, those characters are not universal within the genus, being absent in several species. This might be one reason why the genus has not been universally recognized. After studying species from different regions of the world, we found features that permit better definition of the genus *Illidops*, such as a band of rugosity centrally on the posterior edge of the scutellar disc; a shortened fore wing vein R1; and propodeum fully sculptured but without areola (instead, with a series of short carinae medially on the posterior 0.2–0.3 of the propodeum near the nucha). DNA barcoding tends to clearly cluster species of *Illidops* and *Apanteles* separately (e.g. Smith et al. 2013).

Summarizing, our definition of *Apanteles* *sensu stricto*, as used in this study is based on the following characters: a) propodeum never with a median longitudinal carina; either with carinae defining a partial or complete areola (sometimes areola obscured by propodeum sculpture, but still evident as an impression) or, very rarely, without any definition of an areola; b) Vannal lobe margin strongly concave to almost straight, and lacking setae at midlength (if some setae are present, they are small and sparsely distributed); c) posterior edge of the scutellar disc smooth; d) vein cu-a in the hind wing not sinuate. There are other useful characters to distinguish the genus (e.g. see Mason 1981, Whitfield 1997); but the four detailed above serve well to separate the *Apanteles* *sensu stricto* described here from *Choeras* (character a), from *Dolichogenidea* (character b), from *Exoryza* (character b), from *Iconella* (character d), and from *Illidops* (character c).

A total of 23 *Apanteles* species were previously known from Mesoamerica (Yu et al. 2012, Smith et al. 2013). After studying the type material for these taxa, 19 are retained in *Apanteles* *sensu stricto* and treated in this paper, while four species are transferred to other Microgastrinae genera. The status of the Mesoamerican species *Apanteles albinervis* (Cameron 1904) is revised, and one European species that is involved in a secondary homonym with the former is transferred to *Iconella*. All of these nomenclatorial acts are detailed below.

When the 186 new species from ACG described in this paper are included, the *Apanteles* fauna of Mesoamerica comprises 205 species, or about 10 times more species than previously known from that region. There was only one described species recorded from Costa Rica, 19 from Mesoamerica, 86 from the Neotropical region, and 1010 worldwide (Yu et al. 2012).

This study emphasizes how much is still unknown about the diversity of parasitoid wasps in general, and Microgastrinae in particular (e.g., Rodriguez et al. 2012). It is unlikely that ACG contains 20% of the species of a global genus as widely distributed and diverse as *Apanteles*. A more logical explanation is that, whenever other regions are as comprehensively studied, many more undescribed species of *Apanteles* will be revealed.

Even for ACG we are far from completing the inventory of *Apanteles*. We are aware of another 19 species (which would represent an additional 10% of increase for ACG), which we had to exclude because the available specimens were in poor condition and/or were only represented by males (in most cases Microgastrinae male specimens cannot be taxonomically dealt with, except through their DNA barcodes, or by inference through membership in a presumed sib group containing females, or reared from only one caterpillar). Those species are not described in this paper, although some of their interim names are provided here, for future reference, *Apanteles* Janzen11, *Apanteles* Janzen16, *Apanteles* Janzen34, *Apanteles* Rodriguez50, *Apanteles* Rodriguez74, *Apanteles* Rodriguez75, *Apanteles* Rodriguez79, *Apanteles* Rodriguez109, *Apanteles* Rodriguez121, *Apanteles* Rodriguez127, *Apanteles* Rodriguez128, *Apanteles* Rodriguez138, *Apanteles* Rodriguez143, *Apanteles* Rodriguez149, *Apanteles* Rodriguez161, *Apanteles* Rodriguez185, *Apanteles* Rodriguez200, *Apanteles* Rodriguez216, and *Apanteles* Rodriguez250. Full details of these presumed species and many more can be found in the ACG database online (http://janzen.sas.upenn.edu/caterpillars/database.lasso). We will describe these species in subsequent papers on the ACG Microgastrinae.

### Species formerly described as *Apanteles* but here excluded from the genus

#### 1. Dolichogenidea hedyleptae

(Muesebeck, 1958) comb. n.

Apanteles hedyleptae Muesebeck, 1958: 443. (Puerto Rico, Trinidad and Tobago).

##### Note.

After examinating the holotype (NMNH), we consider this to be a species of *Dolichogenidea*-based on the evenly convex and uniformly setose vannal lobe.

#### 2. Dolichogenidea politiventris

(Muesebeck, 1958) comb. n.

Apanteles politiventris Muesebeck, 1958: 436. (Puerto Rico).

##### Note.

After examinating the holotype (NMNH), we consider this to be a species of *Dolichogenidea*-based on the evenly convex and uniformly setose vannal lobe.

#### 3. Iconella albinervis

(Tobias, 1964) stat rev.

Apanteles albinervis Tobias, 1964: 221. (Kazakhstan). Secondary homonym of *Apanteles albinervis* (Cameron, 1904).
Apanteles albinervis Tobias, 1964. Kept as a valid species by Shenefelt (1972: 438) as a result of an invalid replacement name for *Apanteles albinervis* (Cameron, 1904).
Iconella albinervis (Tobias, 1964). Transferred by Papp (1988: 151).
Apanteles albinervis Tobias, 1964. Transferred by van Achterberg (2003: 27).

##### Remarks.

The name *Apanteles albinervis* Tobias, 1964 could be considered a secondary homonym of *Apanteles albinervis* (Cameron, 1904) [see more explanations below, under the taxonomic treatment of *Apanteles albinervis* (Cameron, 1904) in the section “Taxonomic treatment of the Apanteles species in Mesoamerica, alphabetically by species-groups”]. However, Papp (1988) transferred the Tobias’ species to *Iconella* when treating the Palaearctic species under the (then new) reclassification of Microgastrinae proposed by Mason (1981). In a later paper, van Achterberg (2003) rejected Papp’s work and sank several genera of Microgastrinae under *Apanteles*, with *Iconella* being one of them. As part of those changes, *Apanteles albinervis* Tobias, 1964 was reinstated as a valid name – and, if accepted at present, would become a secondary homonym of *Apanteles albinervis* (Cameron, 1904). However, van Achterberg’s proposal is far from being widely accepted (Broad et al. 2012; Fernández-Triana 2010). We think that the best approach at present is to consider *Iconella* as a valid genus – not a junior synonym of *Apanteles*. Thus, we here transfer Tobias’s species back to *Iconella*, as done earlier by Papp (1988) – an arrangement also accepted by other workers (e.g., Inanç 1997, Kotenko 2007).

Article 59.2 of the International Code of Zoological Nomenclature (ICZN 1999) regulates the case of “Secondary homonyms not replaced when no longer considered congeneric” and states that “If in a case of secondary homonymy the junior species-group name has not been replaced [Art. 60], and the relevant taxa are no longer considered congeneric, the junior name is not to be rejected, even if one species-group name was originally proposed in the current genus of the other”. In accordance with that, the junior name *Iconella albinervis* (Tobias, 1964) is no longer considered congeneric with the senior name *Apanteles albinervis* (Cameron, 1904) and thus there is no need for a replacement name for the Tobias species. However, given previous changes in the generic status of this species, it should be kept in mind that, if future studies bring it back into *Apanteles*, at that moment a replacement name will be needed to avoid a secondary homonym with *Apanteles albinervis* (Cameron, 1904).

#### 4. Illidops scutellaris

(Muesebeck, 1921) comb. rev.

Apanteles scutellaris Muesebeck, 1921: 533.
Illidops scutellaris (Muesebeck). Papp 1988: 150.
Apanteles scutellaris Muesebeck. Austin and Dangerfield 1992: 9; Whitfield 1995: 247; van Achterberg 2003: 29.

##### Remarks.

After examining the holotype (NMNH), we consider this species as belonging to *Illidops*, as pointed out a quarter of a century ago by Papp (1988). Papp transferred the species from *Apanteles* to *Illidops* in his treatment of the European fauna, when he adopted the then recent reclassification of Microgastrinae by Mason (1981). Strangely, his action was overlooked, ignored or rejected by the taxonomic community, and even authors following the Mason system of genera (e.g., Austin and Dangerfield 1992, Whitfield 1995) did not treat the species as *Illidops* but kept it as *Apanteles*. Van Achterberg (2003) considered *Illidops* as a subgenus of *Apanteles*, and transferred all of the European species, including *Illidops scutellaris*, back to *Apanteles*. All of the character defining *Illidops* (as outlined in the above section “Definition of the genus Apanteles sensu stricto”) are present in *Illidops scutellaris*, and thus we here transfer the species *Apanteles scutellaris* back to *Illidops*.

#### 5. Rhygoplitis sanctivincenti

(Ashmead, 1900) comb. n.

Apanteles sanctivincenti Ashmead, 1900: 279. (Saint Vincent).

##### Remarks.

The species *Apanteles sanctivincenti* Ashmead, 1900 was described from a single male, but the type has never been found in the BMNH and is probably lost (Gavin Broad, personal communication). Thus, later researchers reviewing the genus (Muesebeck 1921, Nixon 1965) or cataloguing it (Szépligeti 1904, Shenefelt 1972) were unable to study it, and could only rely upon the very poor original description and key from Ashmead (1900: 279–280). Those five papers are the only publications citing the name *Apanteles sanctivincenti* Ashmead, and the species has been considered as belonging to *Apanteles* since its original description. However, after Mason’s (1981) paper splitting *Apanteles* into several genera, it is evident that *Apanteles sanctivincenti* Ashmead belongs to a different genus, based on its pronotum with a median longitudinal carina, a character that immediately excludes it from the current limits of *Apanteles*, but that occurs in several other genera of Microgastrinae. In his paper revising the fauna of the Caribbean islands of St. Vincent and Grenada, Ashmead (1900) treated five other genera of microgastrines: *Microplitis*, *Protapanteles*, *Protomicroplitis*, *Urogaster* and *Pseudapanteles*. The first three belong to completely different groups which can safely be excluded from the present analysis. *Urogaster* is no longer a valid genus (the majority of its species have been transferred to *Apanteles*). *Pseudapanteles* can also be excluded because its species have a median longitudinal groove on the first mediotergite, a trait not present in *Apanteles sanctivincenti* Ashmead, according to the original description. After carefully considering the distribution of other genera in the region, and comparing it with other species descriptions from the same paper (Ashmead 1900), we believe that the best generic placement for this species is *Rhygoplitis*.

It is worth mentioning that Ashmead (1900: 291) described two other species, *Urogaster aciculatus* and *Pseudapanteles sancti-vincentis*, which are now considered to be the same and to belong to *Rhygoplitis*; the valid species name currently is *Rhygoplitis aciculatus*. It is possible that *Apanteles sanctivincenti* is yet another name for that same species, meaning that three different names in three different genera were applied to the same species by the same author in the same paper! This case is not unlikely, due to Ashmead’s poor knowledge of the Microgastrinae (Mason 1981). In fact, the descriptions in his 1900 paper are not only very inconsistent (characters in the key do not correspond well to the descriptions, descriptions are not homogeneous, some body areas are named differently in the same paper, e.g., knees and femur) but they are also misleading, e.g., the original description of *Urogaster aciculatus* mentions the propodeum with a large, round areola, when it actually has no areola at all. We studied the three descriptions in detail to see if they could correspond to the same species. The lack of uniformity and different terminology prevents a certain conclusion, but they are similar in many regards, differing in minor details such as coloration (which may be meaningless anyway, because of the very small number of specimens examined by the author). Because the holotype of *Apanteles sanctivincenti* is lost, this situation may never be resolved unambiguously. Thus for the sake of name stability, and pending future studies on the genus, we just transfer *Apanteles sanctivincenti* to *Rhygoplitis*.

### ACG species wrongly assigned to *Apanteles* in the past

In a first, non-taxonomic analysis of the Microgastrinae fauna of ACG, Smith et al. (2008) included 136 interim, unnamed, species of *Apanteles* – detailed in their “Datasets 1 and 2” of their “Supporting Information”. After reviewing those specimens, we feel that 12 of those species are better placed in other microgastrine genera and therefore transfer them here (Table 1). The rest are described below, together with additional ACG species identified after the publication of the Smith et al. (2008) paper.

**Table 1.** Species considered as belonging to *Apanteles* by Smith et al. (2008) but transferred to other genera of Microgastrinae in the present paper. After the new assigned genus we provide the interim specific name whenever available (the format being “*Genus* Interim name”, e.g., *Dolichogenidea* Janzen90). The interim names allow for contemporary retrieval of full information of specimens in the online ACG database (http://janzen.sas.upenn.edu/caterpillars/database.lasso) as well as BOLD (www.barcodinglife.org). When those species are revised and published in their respective generic revisions, they will receive an appropriate formal scientific name.

Species name in Smith et al. (2008)	New generic and/or interim species name assigned here
*Apanteles* Rodriguez02	*Parapanteles* Rodriguez02
*Apanteles* Rodriguez45	*Parapanteles* Whitfield45
*Apanteles* Rodriguez90	*Dolichogenidea* Janzen90
*Apanteles* Rodriguez102	*Parapanteles* Whitfield102
*Apanteles* Rodriguez118	*Glyptapanteles* Whitfield175
*Apanteles* Rodriguez119	*Dolichogenidea* Janzen119
*Apanteles* Rodriguez133	*Parapanteles* Whitfield133
*Apanteles* Rodriguez136	*Parapanteles* Whitfield302
*Apanteles* Rodriguez137	*Parapanteles* Whitfield303
*Apanteles* Rodriguez157	The name was applied to several specimens that might represent more than one species. The ones we could study are *Dolichogenidea*, the others are left as *Apanteles* Rodriguez157 and will be dealt with in future papers.
*Apanteles* Rodriguez164	Probably *Dolichogenidea*.
*Apanteles* Rodriguez172	Probably *Dolichogenidea*. We have only seen one specimen lacking legs, antenna and metasoma, which cannot be properly assigned to genus until more material is available.

### General comments on the biology and morphology of *Apanteles* in Mesoamerica

At present we have biological information (host records, solitary/gregariousness of wasp larvae) for 169 (82%) of the described species of *Apanteles* in Mesoamerica. Some records may be questionable, especially early citations of hosts for *Apanteles carpatus* (Say, 1836) which may be incorrect (this is a cosmopolitan species and examination of vouchers from all biogeographic regions is needed to solve the problem). But for the vast majority of species (especially all of the ones reared in ACG) the records are accurate, – and comprehensive enough to draw conclusions on host relationships at a higher taxon level.

**Figure 3.**
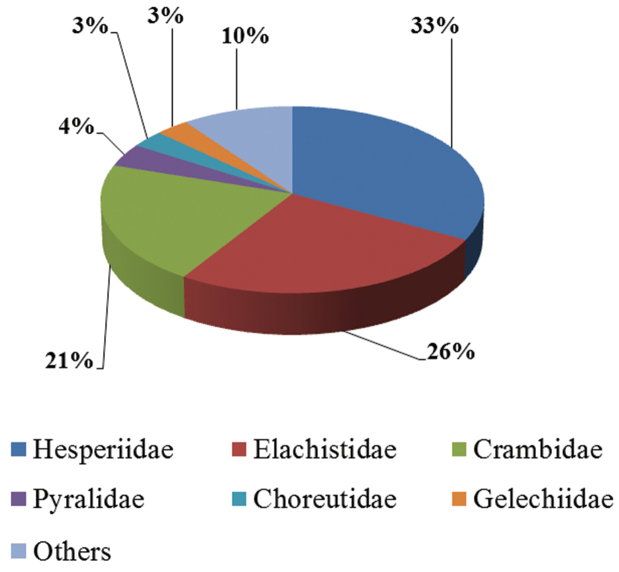
Proportion of Lepidoptera families parasitized by 169 species of *Apanteles* with known host records in Mesoamerica (data source mainly from the ACG inventory).

Twenty Lepidoptera families have been recorded as hosts of *Apanteles* in Mesoamerica (or 14 families, if six only recorded from *Apanteles carpatus*, – which are likely to be wrong –, are excluded). Most hosts species belong to just three families: Hesperiidae (33%), Elachistidae (26%) and Crambidae (21%), distantly followed by Pyralidae (4%), Choreutidae (3%) and Gelechiidae (3%) (Fig. 3). However, the boundary between ACG Elachistidae and Gelechiidae is very poorly defined. When the moth taxa are clearly worked out, the ratio of these two families may be quite different.

Most of the ACG reared *Apanteles* species (154 species, or 91%) are monophagous or oligophagous, attacking one host family, and usually only one or a very few species within the same genus. Only 15 species (9%) are somewhat polyphagous, parasitizing hosts in two or more Lepidoptera families, but even they tend to be very selective in their hosts. Furthermore, larger sample sizes and better DNA barcode data has generally shown that these “somewhat polyphagous” species in ACG are often complexes of narrow specialists, as for example the case of what was believed to be “*Apanteles leucostigmus*” (Smith et al. 2008).

Outside ACG, there is not enough data to assess if narrow host ranges per microgastrine wasp species is a widespread phenomenon (although unpublished evidence suggest that this might be the case and there is no reason to think that ACG *Apanteles* are abnormal). If this pattern proves to be commonplace worldwide, it will have a strong influence on biological control and on biodiversity studies (e.g., Rodriguez et al. 2012, Smith et al. 2013).

A total of 98 species (58%) of the Mesoamerican *Apanteles* with associated biological information have multiple larvae developing in one host caterpillar (gregarious), and these are generally viewed as originating from a single ovipositing female. In contrast, 71 species (42%) are solitary, having just one larva per parasitized caterpillar. Although there is no comparable information from other regions, about 80% of the Nearctic species of *Apanteles* with reliable data available are solitary (Whitfield unpublished data).

Most wasp cocoons (either solitary or gregarious) are stuck to the leaf substrate, and over, under, or near the host cadaver, which “lives” only a few days, if at all, after emergence of the wasp larvae. As cocoon structure often appears a species-level characteristic, it is shown for each species when possible (Figs 210–330).

The fauna of Mesoamerica, especially that of ACG, seems to have some peculiar morphological characteristics. For example, one species in ACG is the only known *Apanteles* in the world with partially white genae. That feature is present in *Alphomelon* and occasionally in a few other genera of Microgastrinae (e.g., Mason 1981, Deans et al. 2003), but had never before been found in *Apanteles*.

Although orange-yellow coloration is not uncommon in tropical *Apanteles*, it is mostly restricted to legs, portions of metasoma, and, rarely, spots on the mesosoma. Four ACG species (2%) are the first known members of the genus to have extensive orange coloration, including the whole head. Interestingly, none of these four species seem to be closely related.

Similarly, only five ACG species have pectinate tarsal claws, while one species has cleft tarsal claws. The vast majority (97.5%) of the Mesoamerican species either have simple tarsal claws, or with 1–2 basal spine-like setae.

About 10% of the Mesoamerican *Apanteles* within several groups (including *anabellecordobae*, which is the third largest species-group in the region) have the hypopygium either unfolded or with only 1–3 pleats. That is very unusual in *Apanteles* and may force a future redefinition of *Apanteles* limits.

Almost one quarter of the *Apanteles* species in Mesoamerica have a somewhat elongate glossa, although it is never as large and bilobate as in some other characteristic genera of Microgastrinae such as *Pseudapanteles*, *Promicrogaster*, etc.

### Species groups of Mesoamerican *Apanteles*

In order to deal with its high diversity, the genus *Apanteles* has been partitioned into species groups since 1880. Mason (1981) provides a summary of current understanding of the evolution of those groups as well as references to different papers on the topic. A total of 44 species-groups for the world fauna were proposed by Nixon (1965), an arrangement that has generally been accepted and incorporated into subsequent revisions, e.g., Mason (1981) and European fauna (revised by Papp between 1976 and 1990).

While some of these species groups appear to represent monophyletic or at least morphologically coherent groups, many are poorly defined, and some are just containers for species that do not fit into any other group. To further complicate things, many species have never been assigned to a particular species-group (e.g., only half of the previously described species of Mesoamerican *Apanteles* had been assigned to a group before this paper).

In spite of the shortcomings in the species-group system, it remains a useful tool for partitioning the large number of *Apanteles* species. Until a more comprehensive, phylogeny-based taxonomy is available, groups of species based on inference from morphology remain the most practical approach.

For the Mesoamerican region we recognize and propose 32 species-groups of *Apanteles* (Table 2) and we assign most of the species known for the region to one of them. All groups are new, except for two (*Apanteles ater* and *Apanteles diatraeae*) previously created and used by several authors (e.g., Nixon 1965, Mason 1981, Austin and Dangerfield 1989, Whitfield et al. 2001, 2002). For 30 species we did not have strong support to assign them to any of the 32 established groups; and neither the morphological, molecular nor biological data are sufficient to justify them as individual groups. Those 30 species are left out of groups, and categorized as “unassigned”.

**Table 2.** Species-groups of *Apanteles* in Mesoamerica, in alphabetical order. # **of Spp:** Total number of species currently within a group. **Lepidoptera host families:** “?” Unknown; for the *carpatus* group “??” refers to a mix of old and questionable references including nine different families (Gelechiidae, Lasiocampidae, Lecithoceridae, Lymantriidae, Pyralidae, Thaumetopoeidae, Tineidae, Tortricidae, Zygaenidae). **Larvae:** **S**–wasp larvae solitary; **S**?–wasp larvae strongly suspected to be solitary but not conclusive evidence; **G**–wasp larvae gregarious. ?–Unknown. In cases where a species-group has both solitary and gregarious larvae, the most common occurrence is indicated first. **MOR**, **DNA**, **BIO:** degree of group support by morphological (MOR), molecular (DNA), and biological (BIO) data. “+” Strong support, “-”No support, “**P**” Partial support, “?” Unknown.

Species-group	# of Spp	Lepidoptera host families	Larvae	MOR	DNA	B IO
*adelinamoralesae*	19	Elachistidae, Pyralidae	**G, S**	+	+	+
*adrianachavarriae*	9	Attevidae, Crambidae, Elachistidae, Tortricididae	**S, G**	**P**	**P**	**P**
*adrianaguilarae*	3	Tortricidae	**G**	+	+	+
*alejandromorai*	13	Elachistidae, Gelechiidae	**S**	+	+	+
*anabellecordobae*	14	Hesperiidae	**G, S**	+	+	+
*anamarencoae*	2	Elachistidae, Oecophoridae, Tortricidae	**S**	+	-	-
*arielopezi*	2	Elachistidae, Tortricidae	**G**	+	+	?
*ater*	9	Crambidae, Pyralidae	**S**	-	-	?
*bernyapui*	4	Crambidae, Elachistidae, Gelechiidae, Noctuidae	**S**	+	+	?
*bienvenidachavarriae*	3	Elachistidae	**S**	+	**P**	+
*calixtomoragai*	3	Hesperiidae	**S**	+	+	+
*carlosguadamuzi*	6	Choreutidae, Crambidae, Gelechiidae, Elachistidae	**G, S**	+	+	?
*carlosrodriguezi*	3	Elachistidae, Choreutidae, Crambidae	**G, S**	+	**P**	**P**
*carloszunigai*	2	?	?	+	+	?
*carpatus*	5	??	**G**	+	+	?
*coffeellae*	4	Gracillariidae, Lyonetiidae	**S**?	**P**	-	?
*diatraeae*	3	Crambidae	**G**	+	+	+
*dickyui*	2	?	?	+	+	?
*erickduartei*	5	Crambidae	**S**	+	+	+
*glenriverai*	2	Pyralidae	**G**	+	+	+
*guadaluperodriguezae*	2	Crambidae	**G**	+	-	+
*humbertolopezi*	2	Elachistidae	**S**	+	-	?
*isidrochaconi*	2	?	?	+	+	?
*javierobandoi*	2	Choreutidae	**S**	+	-	?
*Joserasi*	2	Hesperiidae	**S**	+	+	+
*keineraragoni*	2	Crambidae, Riodinidae	**G, S**	+	-	-
*Leucostigmus*	39	Hesperiidae	**G**	+	+	+
*marisolnavarroae*	2	Pyralidae	**S**	+	+	+
*megathymi*	2	Hesperiidae	**G**	+	?	-
*paranthrenidis*	4	Pyralidae, Crambidae, Gelechiidae, Noctuidae, Sesiidae	**S, G**	+	?	-
*ronaldgutierrezi*	2	Choreutidae	**S**	+	**P**	?
*Samarshalli*	2	?	?	+	+	?

Six groups each have nine or more species, jointly representing half of all described Mesoamerican species. The largest are the *leucostigmus* group (39 species parasitizing Hesperiidae) and the *adelinamoralesae* group (19 species attacking Elachistidae), both with many more Mesoamerican species awaiting description.

A total of 15 species-groups (47%) are represented by two species. This situation is mainly due to the fact that only the ACG fauna has been comprehensively studied. When the remainder of the Mesoamerican *Apanteles* fauna is revised, many of these groups are likely to have more species associated. We have seen in collections numerous undescribed species from the Neotropics other than ACG, species that fall into some of the new groups erected here (to encourage further study in the future we have noted those cases in the taxonomy treatment of species below).

Most groups (79%) are strongly supported by at least two of three sets of independent data: molecular (DNA barcodes), biology (host), and morphology (Table 2), and their component species can be clearly recognized and delimited. Others are defined mainly by shared morphological characters. In one case (*Apanteles joserasi*), the group could only be defined by a unique combination of hosts and barcoding characters (see couplet 29 of the key to species-groups).

Some groups partially overlap with others, e.g., the *adrianachavarriae, javierobandoi*, and *joserasi* groups, and future studies may reveal that they should be merged. However, without study of the whole Mesoamerican (or even Neotropical) fauna, we preferred to take a conservative approach in accommodating the perceived differences.

The non-ACG *ater*, *coffeellae, megathymi*, and *paranthrenidis* groups could not be defined unambiguously, and should only be considered as interim groupings of species; they will need to be revisited when more studies on the world fauna are undertaken.

The species *aidalopezae* and *leonelgarayi* (currently not assigned to any group), and the groups *carlosrodriguezi* and *samarshalli*, all comprise species that might be better placed in other genera in the future. For example, the *samarshalli* group clusters out of all other *Apanteles* species, strongly indicating (PP: 1.0 in the Bayesian analysis, Fig. 1) that its two species may best be placed in a (new) different genus. However, pending a comprehensive phylogenetic study of Microgastrinae, we decided that it is best to here describe all those species as belonging to *Apanteles*. The key to species groups separate those species in the first four couplets.

### Key to the species-groups of Mesoamerican *Apanteles*

[This section provides a key to all species-groups of *Apanteles* in Mesoamerica, including 30 species that could not be assigned to any current group and are keyed individually throughout the key. It is followed by keys to species within every species-group (the groups arranged in alphabetical order). After all keys, standardized descriptions of every species are provided (the species arranged in alphabetical order). To facilitate finding individual species, Table 3 provides alphabetical lists of species and species-groups].

**Table:** 

1	Fore wing with vein 2M very short, its anterior half very close to anterior half of vein 2RS, in a way that obliterates most of space of second submarginal cell (Figs 160b, 205a, c); antenna very short, 0.5 × body length, and not surpassing posterior margin of mesosoma (Figs 160e, 205a–c), ***and*** body not distinctly flattened dorsoventrally, ***and*** pro- and meso- femora yellow, ***and*** pterostigma relatively broad, its length less than 2.7 × its width [Distribution: Canada (ON), Costa Rica (ACG), Mexico and US (FL)]	*samarshalli* species-group [2 species]
–	Fore wing with vein 2M completely separated from vein 2RS (as in Fig. 4b); antenna usually as long or longer than body length, at least surpassing posterior margin of mesosoma; ***if*** antenna shorter (i.e., not surpassing posterior margin of mesosoma), ***then*** body distinctly flattened dorsoventrally (as in Fig. 203a), ***and/or*** profemur (partially or entirely) and mesofemur dark brown to black, ***and/or*** pterostigma usually relatively narrow, its length more than 3.0 × its width	2
2(1)	Ovipositor sheaths extremely short, 0.3 × or less metatibia length (Figs 138a, c); T2 relatively large, its median length 0.7–0.9 × as long as T3 median length (Fig. 138f); T1 mostly smooth (except for 2–3 small carinae centrally); body with extensive yellow-orange coloration (all legs except for metatarsus and posterior 0.2 of metatibia, tegula and humeral complex, all laterotergites and sternites, hypopygium)	*Apanteles leonelgarayi* Fernández-Triana, sp. n.
–	Ovipositor sheaths at least 0.4 × as long as metatibia (usually much more than that); T2 median length much shorter than T3 median length (almost always 0.5 × or less); T1 almost always with some sculpture; body color variable	3
3(2)	Hypopygium with a relatively wide but short fold, restricted to posterior 0.4–0.5 of hypopygium length, where no pleats are visible (or, rarely, at most with a single, weakly marked pleat); ovipositor short and slightly to strongly curved downwards (Figs 36a, c); ovipositor sheaths very short (0.4–0.5 × as long as metatibia, Fig. 36c); relatively small size, body length and fore wing length not surpassing 2.5 mm	4
–	Hypopygium usually with large fold and numerous pleats, ***if*** rarely with no visible pleats or just one pleat, ***then*** ovipositor relatively long and thick, not strongly curved downwards, ***and/or*** ovipositor sheaths longer than 0.5 × metatibia length (usually much longer), ***and/or*** body length and fore wing length surpassing 2.5 mm	5
4(3)	Pterostigma white (Fig. 36b); glossa elongate; antenna much shorter than body, not extending beyond mesosoma (Fig. 36a)	*Apanteles aidalopezae* Fernández-Triana, sp. n.
–	Pterostigma brown, with small pale spot at base (Fig. 96b); glossa not elongate; antenna usually as long as body or slightly shorter (extending beyond mesosoma)	*carlosrodriguezi* species-group [3 species]
5(3)	Head entirely orange (except for black interocellar area and/or small spot on upper part of gena), anteromesoscutum, scutellar disc, and axillar complex completely or almost completely orange (Figs 37, 135, 139, 163)	6
–	Head mostly black to dark brown (except for clypeus and labrum, which may be yellow-orange) ***or*** head black with gena partially white; anteromesoscutum and scutellar disc usually black to dark brown, at most with relatively small yellow or orange spots	9
6(5)	Mesopleuron and mesosternum dark brown to black, except for upper anterior and/or lower posterior corners of mesopleuron which are orange (Figs 37a, 163a)	7
–	Mesopleuron either completely orange, or mostly orange (upper anterior 1/3 dark brown to black), mesosternum fully orange (Figs 135a, 139a)	8
7(6)	Mesoscutellar disc smooth (Fig. 163g); all mediotergites dark brown to black (Fig. 163g); tarsal claws pectinate	*Apanteles waldymedinai* Fernández-Triana, sp. n.
–	Mesoscutellar disc mostly punctured (Fig. 37e); T1 mostly orange and T3 partially yellow (Fig. 37e); tarsal claws with one basal spine-like seta	*Apanteles alejandromasisi* Fernández-Triana, sp. n.
8(6)	T1 mostly white except for small black spot posteriorly (Fig. 135f); all laterotergites, most sternites, and hypopygium white; scutoscutellar sulcus almost obliterated, with less than 4 small impressions (Fig. 135f); propodeal areola open basally and without transverse carinae; tarsal claws pectinate	*Apanteles juliodiazi* Fernández-Triana, sp. n.
–	Metasoma entirely black (Fig. 139f); scutoscutellar sulcus not obliterated, with 5–6 small impressions (Fig. 139f); propodeal areola closed basally and with transverse carinae extending to spiracle; tarsal claws with one basal spine-like seta	*Apanteles luisgaritai* Fernández-Triana, sp. n.
9(5)	Head with gena partially white (Figs 155a, d)	*Apanteles rogerblancoi* Fernández-Triana, sp. n.
–	Head with gena entirely black	10
10(9)	Tarsal claws pectinate, ***or*** cleft with a basal spine [Hosts: Hesperiidae, Pyrginae]	11
–	Tarsal claws either simple ***or*** with 1–2 basal spine-like setae	12
11(10)	T1 coarsely sculptured with longitudinal and transverse striation; T2 with some sculpture, especially along posterior margin (Fig. 119f); tarsal claws cleft and with a basal spine	*Apanteles garygibsoni* Fernández-Triana, sp. n.
–	T1 smooth, at most with fine sculpture along lateral margins; T2 smooth and polished (Fig. 87e); tarsal claws pectinate	*calixtomoragai* species-group [3 species]
12(10)	Smaller individuals, body length 1.6–2.2 mm, and fore wing length 1.8–2.4 mm; ***and*** body distinctly flattened (as in Figs 203a, 204a) ***or*** T1 length >3.5 × its posterior width (as in Figs 106g, 107f, 108f, 203g)	13
–	Larger individuals, body length and fore wing lengths usually more than 2.5 mm; ***if*** rarely less than 2.5 mm, ***then*** body not distinctly flattened ***and*** T1 length <3.5 × its posterior width	17
13(12)	All legs, including coxae (except for small spot on anterior 0.2 of metacoxa), entirely yellow or whitish-yellow (Figs 83a, c, g)	*Apanteles bettymarchenae* Fernández-Triana, sp. n.
–	At least metacoxa completely, and part of femora and tibiae, with dark brown to black coloration	14
14(13)	Antenna as long as or longer than body length; T1 strongly narrowing toward apex from its apical half — ratio of T1 basal width/T1 apical width >2.0 × and ratio of T1 length/T1 apical width >3.5 × (Fig. 106g); body not distinctly flattened; parasites of leaf-mining Lepidoptera. [Hosts: Gracillariidae, Lyonetiidae. Distribution: Costa Rica (ACG), Guadeloupe, Puerto Rico]	*coffeellae* species-group [4 species]
–	Antenna shorter, its length at most 0.7 × body length, usually much less; T1 usually narrowing toward apex less strongly — ratio of T1 basal width/T1 apical width <2.0 ×, ratio of T1 length/T1 apical width usually <3.5 × (Figs 203g, 204g); body distinctly flattened dorsoventrally (as in Figs 203a, 204a); parasites of non-mining Lepidoptera	15
15(14)	Smooth area on lateral face of scutellum very narrow and small, its maximum height at most 0.2 × lateral face height (Fig. 67e); hypopygium inflexible, without any fold [Hosts: Tortricidae]	*Apanteles anapiedrae* Fernández-Triana, sp. n.
–	Smooth area on lateral face of scutellum at least 0.4 × lateral face height (usually much more) (Fig. 122f, 203g, 204g); hypopygium with a translucid median fold with at least one pleat visible	16
16(15)	Propodeal areola open anteriorly, elongate and more or less parallel-sided (Figs 203g, 204g), its maximum width (at around half of propodeum length) <1.3 × its width at posterior end (nucha); hypopygium with a wide median fold with usually four or more visible pleats [Hosts: stem-boring Crambidae]	*diatraeae* species-group [3 species]
–	Propodeal areola clearly closed anteriorly and widening centrally, its maximum width (at around half of propodeum length) >1.5 × its width at apex (nucha); hypopygium with a translucid median fold with 1–3 visible pleats [Hosts: leaf-folder Crambidae]	*guadaluperodriguezae* species-group [2 species]
17(12)	Hypopygium with outer margin inflexible, without a median fold (as in Figs 51c, 56c), ***or*** hypopygium with a median, transparent, semi-desclerotized fold with none or very few (usually 1–3) pleats occupying just outermost area of fold (as in Figs 52c, 55c)	18
–	Hypopygium with outer margin with a wide median, transparent, semi-desclerotized fold, with 4 or more pleats occupying most or whole fold (as in Fig. 145d)	23
18(17)	Ovipositor relatively thick, as thick or thicker than width of median flagellomerus, and with basal width 3.0–5.0 × its apical width posterior to constriction (Figs 51c, 52a, c, 54c, 56c)	*anabellecordobae* species-group [14 species]
–	Ovipositor relatively thin, thinner than width of median flagellomerus, and with basal width <2.0 × its apical width after constriction (as in Figs 68a, c, 142c)	19
19(18)	T1 mostly smooth (Fig. 156g); T1 slightly widening from anterior margin to 0.7–0.8 × mediotergite length (where maximum width is reached), then narrowing towards posterior margin	*Apanteles rolandovegai* Fernández-Triana, sp. n.
–	T1 mostly sculptured, at least on posterior half (Figs 68g, 142f); T1 more or less parallel-sided for its entire length, or parallel-sided for 0.5–0.7 × its length then narrowing posteriorly so mediotergite anterior width >1.1 × posterior width (Figs 68g, 142f)	20
20(19)	T1 length 1.4 × its width; fore wing length 3.3 mm	*Apanteles marialuisariasae* Fernández-Triana, sp. n.
–	T1 length at least 2.3 × its width; fore wing length at most 2.8 mm	21
21(20)	All coxae, profemur partially, and meso- and metafemora completely, dark brown to black (Fig. 68a); mesoscutellar disc mostly smooth (Fig. 68g); hypopygium with outer margin inflexible, without a median fold	*Apanteles andreacalvoae* Fernández-Triana, sp. n.
–	At least pro- and mesocoxae (and usually metacoxa), pro- and mesofemora, and most of metafemur (except for apical 0.2 or less), yellow to orange (Figs 99a, c, 149a, c); mesoscutellar disc mostly punctured, or with punctures near margins and centrally smooth (Figs 99g, 149f); hypopygium with a median, transparent, semi-desclerotized fold with none or very few (usually 1–3) pleats occupying just outermost area of fold	22
22(20)	Flagellomerus 14 1.0 × as long as wide; scutoscutellar sulcus with 9 impressed pits; tarsal claws with one basal spine-like seta; T1 length 2.3 × its width; T2 with some sculpture near its posterior margin (Fig. 149f)	*Apanteles oscarchavesi* Fernández-Triana, sp. n.
–	Flagellomerus 14 at least 1.6 × as long as wide; scutoscutellar sulcus with 5–6 impressed pits; tarsal claws simple; T1 length at least 3.2 × its width; T2 mostly smooth (Fig. 99g)	*carloszunigai* species-group [2 species]
23(17)	T2 broadly rectangular, its apical width 2.2 × or less than its median length (as in Figs 38e, 39g, 40f, 105g, 112f)	24
–	T2 transverse and relatively narrow, its apical width 2.5 × or more its median length	26
24(23)	Ovipositor relatively thick (Fig. 112c), as thick or thicker than width of median flagellomerus, and with basal width 3.0–5.0 × its apical width posterior to constriction [Hosts: Hesperiidae. Distribution: ACG]	*Apanteles diegotorresi* Fernández-Triana, sp. n.
–	Ovipositor relatively thin (as in Fig. 38a), thinner than width of median flagellomerus, and with basal width <2.0 × its apical width after constriction [Hosts: Elachistidae. Distribution: ACG]	25
25(24)	Ovipositor sheaths more than 1.2 × as long as metatibia, and usually longer than metasoma (as in Fig. 38a); fore wing with maximum width of first discal cell at most 1.1 × its maximum height (usually 1.0 × or less), second abscissa of vein 1CU slightly curved (as in Figs 38b, 39b, 40b, 41b, 42b, 43b, 44b, 46b); T1 less than 3.3 × as long as its apical width	*alejandromorai* species-group [13 species]
–	Ovipositor sheaths less than 1.0 × as long as metatibia, and much shorter than metasoma (Fig. 105a); fore wing with maximum width of first discal cell 1.3 × its maximum height, second abscissa of vein 1CU straight (Fig. 105b); T1 more than 3.4 × as long as its apical width	*Apanteles christianzunigai* Fernández-Triana, sp. n.
26(23)	Pterostigma relatively broad, its length less than 3.0 × its width (as in Fig. 104b), ***and*** T2 mostly sculptured with strong longitudinal striation (Figs 102g, 103g, 104g)	*carpatus* species-group [5 species]
–	Pterostigma relatively narrow, its length more than 3.0 × its width, ***and*** T2 *either* smooth ***or*** weakly sculptured, without strong longitudinal striation	27
27(26)	Ovipositor relatively thick and strong, as thick or thicker than width of median flagellomerus and with basal width 3.0-5.0 × its apical width posterior to constriction (Figs 133a, c, 168c, 172c, 179c)	28
–	Ovipositor relatively thin, thinner than width of median flagellomerus, and with basal width <2.0 × its apical width after constriction	30
28(27)	Maximum height of mesoscutellum lunules 0.4 × maximum height of lateral face of mesoscutellum (Fig. 120f); antenna shorter than body length; propodeum usually evenly sculptured in most of its surface (Fig. 120f) [Hosts: Pyralidae]	*glenriverai* species-group [2 species]
–	Maximum height of mesoscutellum lunules 0.7 × or more maximum height of lateral face of mesoscutellum (as in Fig. 133f); antenna as long or longer than body length; propodeum with strong sculpture limited to anterior half, posterior half mostly smooth and shiny; propodeum with transverse carinae complete and strongly raised, clearly delimited from background sculpture (as in Fig. 133f) [Hosts: Hesperiidae]	29
29(28)	Solitary parasitoids of *Venada* (Hesperiidae); cocoons as in Fig. 291 [See comments under species-group treatment for further justification on its status]	*joserasi* species-group [2 species, one undescribed]
–	Gregarious parasitoids of several genera of Hesperiidae but not *Venada*; cocoons as in Figs 304–329	*leucostigmus* species-group [39 species]
30(27)	Body with extensive yellow and/or orange coloration, including tegula and humeral complex, parts of the axillar complex, sometimes posterior margin of mesoscutum (right in front of scutoscutellar sulcus), all coxae (rarely metacoxa dark brown to black), sometimes lateral edges of T3 and T4, most of laterotergites 1–4, most sternites and hypopygium (partial or completely) (as in Figs 33a, f, 114a, f, 127a, f, 141a, f, 159g, 161a, c)	31
–	Body with much less extensive yellow-orange coloration: usually metacoxa (and sometimes also pro- and meso- coxae) partially to completely reddish, brown or black; axillar complex, tergites, most of laterotergites, and hypopygium (partial or completely) dark brown to black; tegula and humeral complex color variable but rarely both yellow	43
31(30)	T2 mostly sculptured (Fig. 159g)	*Apanteles rosibelelizondoae* Fernández-Triana, sp. n.
–	T2 mostly smooth, at most with some sculpture near the posterior margin	32
32(30)	T1 length at least 3.8 × (usually more than 4.0 ×) its width at posterior margin (Fig. 141f, 161h) ***and*** ovipositor sheaths 0.4 × as long as metatibia (Fig. 161a, c)	33
–	T1 length at most 3.2 × its width at posterior margin ***and/or*** ovipositor sheaths at least 0.6 × as long as metatibia	34
33(32)	Body length 3.0–3.2 mm, forewing length 3.1–3.3 mm; tegula and humeral complex dark brown; anteromesoscutum with two orange spots laterally near posterior margin (Fig. 141f); tarsal claws simple; ocular-ocellar line 2.1 × as long as posterior ocellus diameter; interocellar distance 1.6 × posterior ocellus diameter; flagellomerus 14 1.2 × as long as wide; flagellomerus 2 2.5 × as long as flagellomerus 14; T2 width at posterior margin 3.0–3.4 × its medial length [Hosts: Crambidae]	*Apanteles marcogonzalezi* Fernández-Triana, sp. n.
–	Body length 2.4–2.7 mm, forewing length 2.6–2.7 mm; tegula and humeral complex yellow; anteromesoscutum entirely black (Fig. 161h); tarsal claws with one basal spine-like seta; ocular-ocellar line 2.5 × as long as posterior ocellus diameter; interocellar distance 2.0 × posterior ocellus diameter; flagellomerus 14 1.6 × as long as wide; flagellomerus 2 2.0 × as long as flagellomerus 14; T2 width at posterior margin 3.6 × its medial length [Hosts: Choreutidae]	*Apanteles sergiocascantei* Fernández-Triana, sp. n.
34(32)	T1 length 1.8 × its width; ***and*** mesoscutellar disc smooth (Fig. 162e); ***and*** fore wing vein 2RS more than 2.0 × as long as vein 2M; ***and*** T2 width at posterior margin 3.9 × its length; ***and*** ocular-ocellar line 1.6 × posterior ocellus diameter [Hosts: Crambidae borers, *Diatraea* spp.]	*Apanteles vulgaris* Fernández-Triana, sp. n.
–	T1 length usually more than 2.3 × its width; ***and/or*** mesoscutellar disc with punctures; ***and/or*** fore wing with vein 2RS less than 2.0 × as long as vein 2M; ***and/or*** T2 width at posterior margin less than 3.6 × its length; ***and/or*** ocular-ocellar line at least 1.7 × posterior ocellus diameter [Hosts: Choreutidae, Elachistidae, Gelechiidae, Tortricidae; if Crambidae, not borers]	35
35(34)	Ovipositor sheaths 1.3 × as long as metatibia (Figs 127a, c, 128a, c); ***and*** body length and fore wing length 4.0 mm; ***and*** mesoscutellar disc smooth centrally (Figs 127f, 128f); ***and*** ocular-ocellar line 1.5 × posterior ocellus diameter	*isidrochaconi* species-group [2 species]
–	Ovipositor sheaths usually shorter than metatibia (rarely 1.1–1.2 ×); body length and fore wing length usually less than 3.0 mm, ***if*** more than that (up to 3.5 mm) ***then*** mesocutellar disc punctured, ***and/or*** ocular-ocellar line at least 2.0 × posterior ocellus diameter	36
36(35)	Metacoxae entirely or mostly (anterior 0.5 or more) dark brown to black (as in Figs 34a, 115f)	37
–	All coxae entirely white, yellow or bright orange, at most with small brown spot on anterior 0.1 or less (as in Figs 33f, 114f, 116a, f)	38
37(36)	Fore wing with length of vein r 1.4 × or less length of vein 2RS; ocular-ocellar line at least 2.4 × posterior ocellus diameter; T2 width at posterior margin at least 3.7 × its length	*adrianaguilarae* species-group (in part) [3 species]
–	Fore wing with length of vein r 2.4 × length of vein 2RS; ocular-ocellar line 2.2 × posterior ocellus diameter; T2 width at posterior margin at least 3.3 × its length	*erickduartei* species-group (in part) [5 species]
38(36)	Ocular-ocellar line 2.5 × as long as posterior ocellus diameter; ***and*** T2 width at posterior margin at least 4.0 × (usually more) as long as its medial length; ***and*** fore wing with vein 2M as long as vein (RS+M)b	*adrianaguilarae* species-group (in part) [3 species]
–	Ocular-ocellar line at most 2.2 × as long as posterior ocellus diameter; ***and/or*** T2 width at posterior margin usually 3.5 × (or much less) as long as its medial length; ***and/or*** fore wing with vein 2M usually shorter than vein (RS+M)b	39
39(38)	Mesoscutellar disc mostly punctured (as in Figs 114f, 115f)	40
–	Mesoscutellar disc mostly smooth, at most with few, scattered punctures near margins, central part smooth (as in Figs 80f, 81g, 134f); ***if*** rarely mostly punctured, ***then*** posterior 0.2–0.3 of anteromesoscutum (especially centrally and along posterior margin) and upper anterior corner of mesopleura orange (as in Figs 80f, 82g)	41
40(39)	Ovipositor sheaths clearly as long or longer as metatibia (1.0–1.2 ×, rarely 0.9 ×); tarsal claws with one basal spine-like seta	*erickduartei* species-group (in part) [5 species]
–	Ovipositor sheaths clearly shorter than metatibia (0.4 ×) (Figs 118a, c); tarsal claws simple	*Apanteles flormoralesae* Fernández-Triana, sp. n.
41(39)	T1 mostly sculptured, with excavated area centrally with transverse striation inside and polished knob centrally on posterior margin of mediotergite ***and*** T1 mostly parallel–sided for 0.5–0.7 of its length, then narrowing posteriorly so mediotergite anterior width >1.1 × posterior width (Fig. 134f), ***and*** anteromesoscutum ***and*** T1 entirely black; T2 width at posterior margin 5.4 × its length; metafemur length 3.5 × its width	*Apanteles juanhernandezi* Fernández-Triana, sp. n.
–	T1 mostly smooth (as in Fig. 90g), ***if*** mostly sculptured, ***then*** T1 mostly parallel-sided (as in Fig. 79g), ***or*** anteromesoscutum with posterior 0.2 orange (as in Fig. 80f) ***and/or*** T1 orange to light-brown; T2 width at posterior margin at most 4.0 × (usually much less) its length; metafemur length at most 3.2 × its width (usually 3.0 × or less)	42
42(41)	T1 almost always black, same color of propodeum (some decoloured specimens may have T1 dark brown); T1 length at most 2.3 × its width, and mostly strongly sculptured, with longitudinal striation laterally and a central excavated area with transverse striation (Fig. 79g)	*bernyapui* species-group [4 species]
–	T1 orange-yellow, orange or light brown, always lighter than propodeum color (as in Fig. 90g); T1 length at least 2.5 × its width (usually much more), with some weak sculpture on posterior 0.2–0.5 but mostly looking smooth (Fig. 90g)	*carlosguadamuzi* species-group [6 species]
43(30)	Tegula different in color from humeral complex	44
–	Tegula same color as humeral complex	57
44(43)	Pterostigma mostly transparent or white, with thin brown borders; ***and*** all coxae dark brown to black	45
–	Pterostigma either fully brown, mostly brown (at most with small pale area centrally or anteriorly), or fully white, without brown borders; ***and/or*** procoxa (sometimes also mesocoxa) yellow-orange to light brown	51
45(44)	T1 at most 1.3 × as long as wide at posterior margin, ***and*** T2 mostly smooth ***and/or*** pro- and mesocoxae yellow	*paranthrenidis* species-group [4 species]
–	T1 at least 1.7 × as long as wide (usually much more), ***if*** rarely 1.3 ×, ***then*** T2 fully sculptured with longitudinal striation ***and*** all coxae dark brown to black	46
46(45)	Glossa elongate in both sexes (Fig. 140e) [Hosts: Crambidae. Distribution: ACG]	*Apanteles luisvargasi* Fernández-Triana, sp. n.
–	Glossa not elongate	47
47(46)	Ovipositor sheaths usually more than 1.2 × metatibial length, ***if*** rarely 1.0–1.1 ×, ***then*** profemur at least partially, and meso- and metafemora completely, dark brown to black; ***and*** tegula yellow-white; ***and*** fore wing with vein 2RS less than 2.0 × length of vein 2M (usually less than 1.6 ×); ***and*** humeral complex half yellow-white, half dark brown; ***and*** T2 width at posterior margin at least 2.9 × its length [Host: Elachistidae]	*adelinamoralesae* species-group [19 species]
–	Ovipositor sheaths usually less than 1.1 × metatibial length; ***if*** rarely more than 1.2 ×, ***then*** pro- and mesofemora completely, and metafemur at least partially, yellow-orange; ***and/or*** fore wing with vein 2RS more than 2.0 × length of vein 2M; ***and/or*** humeral complex usually unicolour (or very rarely humeral complex half yellow-white, half dark, and tegula dark brown); ***and/or*** T2 width at posterior margin at most 2.7 × its length	48
48(47)	Propodeum with sculpture on anterior half different from posterior half (which is either smoother or with clearly different pattern of sculpture than the anterior half) (Figs 23e, 24e, 25f, 26f, 27f, 28f, 29f, 30g, 31g, 84g, 85f, 86f)	49
–	Propodeum fully sculptured, without much difference in sculpture between anterior and posterior halves (as in Fig. 146f)	50
49(48)	T2 width at posterior margin at most 2.7 × (usually 2.5 × or less) its length (Figs 84g, 85f, 86f) [Hosts: Elachistidae]	*bienvenidachavarriae* species-group [3 species]
–	T2 width at posterior margin at least 3.5 × its length (Figs 23e, 24e, 25f, 26f, 27f, 28f, 29f, 30g, 31g) [Hosts: Crambidae, Tortricidae, Yponomeutidae]	*adrianachavarriae* species-group [9 species]
50(48)	Profemur partially, and meso- and metafemora completely, dark brown to black; ovipositor relatively thick (anterior width 2.0 × as posterior width) (Fig. 132c)	*josediazi* species-group
–	Pro- and mesofemora completely to partially yellow-orange; ovipositor relatively thin, about same width throughout its length (Fig. 146c)	*megathymi* species-group [2 species]
51(44)	Ovipositor sheaths 1.4–1.5 × as long as metatibia length (Figs 129a, c); ***and*** body length and fore wing length at least 3.2 mm	*Apanteles isidrovillegasi* Fernández-Triana, sp. n.
–	Ovipositor sheaths usually less than 1.1 × as long as metatibia length; ***if*** rarely ovipositor sheaths 1.3 × as long as metatibia length, ***then*** body length and fore wing length at most 2.2 mm	52
52(51)	Glossa elongate (Fig. 143e) [Host: Elachistidae]	*Apanteles mariamendezae* Fernández-Triana, sp. n.
–	Glossa not elongate	53
53(51)	T1 mostly parallel-sided for 0.7 of its length, then strongly narrowing posteriorly so T1 length at least 3.0 × its width at posterior margin (Fig. 124f) [Host: Riodinidae]	*Apanteles hectorsolisi* Fernández-Triana, sp. n.
–	T1 either clearly widening towards posterior margin, or slightly widening from anterior margin to 0.7–0.8 mediotergite length (where maximum width is reached), then narrowing towards posterior margin, or parallel-sided so T1 length at most 2.5 × its width at posterior margin (usually much less than that)	54
54(53)	T1 clearly widening towards posterior margin, 1.3 × as long as wide at posterior margin; T2 with posterior margin sinuate (Fig. 35e), width at expanded central area 1.7 × as large as width at lateral area; T2 4.0 × as long as wide at posterior margin; ovipositor relatively thick, basal width about twice apical width	*Apanteles aichagirardae* Fernández-Triana, sp. n.
–	T1 parallel-sided or slightly widening from anterior margin to 0.7–0.8 mediotergite length (where maximum width is reached), then narrowing towards posterior margin, so T1 length at least 2.0 × its width at posterior margin; T2 with posterior margin straight, thus central and lateral areas of same length (as in Fig. 69g); T2 usually less than 4.0 × as long as wide at posterior margin; ovipositor about same width throughout its length	55
55(54)	Ovipositor sheaths 0.6 × as long as metatibia length (Figs 69a, c); humeral complex dark; metatrochanter, metatrochantellus, and anterior 0.2–0.3 of metafemur yellow or yellow-white (Fig. 69c)	*ronaldgutierrezi* species-group [2 species]
–	Ovipositor sheaths usually as long as or longer than metatibia length, ***if*** slightly shorter (0.9 ×) ***then*** antenna shorter than body (its length not surpassing half of metasoma); humeral complex half pale, half dark; metafemur, metatrochanter, and sometimes metatrochantellus dark brown to black	56
56(55)	Body length at most 2.4 mm, and fore wing length at most 2.7 mm; mesofemur fully yellow; mesoscutellar disc mostly smooth (Figs 69, 70); ovipositor sheaths at least 1.3 × as long as metatibia length ***or*** antenna shorter than body (its length not surpassing half of metasoma) [Hosts: Crambidae, Elachistidae, Riodinidae, Tortricidae]	*arielopezi* species-group [2 species]
–	Body length at least 3.3 mm (usually more), and fore wing length at least 3.3 mm (usually more); mesofemur anterior 0.5–0.8 dark brown (Figs 144a, 145a); mesoscutellar disc mostly punctured; ovipositor sheaths at most 1.1 × as long as metatibia length [Hosts: Pyralidae]	*marisolnavarroae* species-group [2 species]
57(43)	T1 length more than 4.5 × its posterior width (Fig. 154g); vannal lobe straight and fully setose (with slightly shorter and sparser setae centrally); metacoxa partially yellow and partially dark brown (Fig. 154a)	*Apanteles robertmontanoi* Fernández-Triana, sp. n.
–	T1 length less than 3.7 × its posterior width; vannal lobe usually strongly concave, centrally without setae (or with very small, very sparse setae); metacoxa usually entirely dark brown to black	58
58(57)	Ovipositor sheaths 0.5 × as long as metatibia (Fig. 136a, c); ***and*** relatively small size, body length 2.3 mm, and fore wing length 2.4 mm; ***and*** metatibial spurs at most 0.4 × as long as first segment of metatarsus (Fig. 136c)	*keineraragoni* species-group [2 species]
–	Ovipositor sheaths at least 0.8 × as long as metatibia; ***and/or*** relatively larger size, body length and fore wing length at least 2.5 mm; ***and/or*** metatibial spurs at least 0.5 × as long as first segment of metatarsus	59
59(58)	Glossa elongate (Fig. 130e, 131e); ***and*** tarsal claws simple	*javierobandoi* species-group [2 species]
–	Glossa not elongate; tarsal claws usually with single basal spine-like seta	60
60(59)	Pterostigma entirely brown or brown with pale spot at base	61
–	Pterostigma entirely transparent or mostly transparent with only thin brown borders	63
61(60)	At least pro- and mesocoxae entirely pale (white-yellow, yellow, or orange) (Figs 72a, 73a, 74a, 75a, 76a, 78a)	*ater* species-group (in part)
–	All coxae dark brown to black	62
62(61)	Tegula, humeral complex, all femora and tibiae yellow (metafemur with small brown spot on posterior 0.2 × or less) (Figs 65a, d, e, 66, a); T2 mostly smooth (Fig. 66f); ovipositor sheaths at least 1.4 × as long as metatibia length (Figs 65a, c, 66a, c)	*anamarencoae* species-group [2 species]
–	Tegula, humeral complex, meso- and metafemora dark, metatibia (partially), and usually mesotibia (partially) dark brown to black (Figs 125a, c, f, 126a, c, g); T2 fully sculptured with longitudinal striation (Figs 125f, 126g); ovipositor sheaths at most 1.3 × (usually less than 1.0 ×) as long as metatibia length (Figs 125c, 126c)	*humbertolopezi* species-group [2 species]
63(60)	Tegula and humeral complex dark brown to black, ***or*** pro-, meso-, and part of metacoxae yellow-orange	*ater* species-group (in part)
–	Tegula and humeral complex yellow, ***and*** meso- and metacoxae (sometimes also procoxa) dark brown to black	64
64(63)	T1 length 1.5 × its width (Fig. 148f); T2 mostly smooth (Fig. 148f); body length 3.2 mm, and fore wing length 3.7 mm	*Apanteles monicachavarriae* Fernández-Triana, sp. n.
–	T1 length at least 2.4 × its width (Figs 110g, 11f); T2 sculptured, mostly near posterior margin (Figs 110g, 11f); body length 2.2–2.6 mm, and fore wing length 2.2–2.6 mm	*dickyui* species-group

**Table 3.** Alphabetical lists of Mesoamerican *Apanteles*: by species (columns 1 and 2) and by species-groups (columns 3 and 4).

Ordered by species		Ordered by species-groups	
Species	Species-group	Species-group	Species
*Apanteles adelinamoralesae*	*adelinamoralesae*	*adelinamoralesae*	*Apanteles adelinamoralesae*
*Apanteles adrianachavarriae*	*adrianachavarriae*		*Apanteles carloscastilloi*
*Apanteles adrianaguilarae*	*adrianaguilarae*		*Apanteles didiguadamuzi*
*Apanteles adrianguadamuzi*	*adrianachavarriae*		*Apanteles edgarjimenezi*
*Apanteles aichagirardae*	Unassigned		*Apanteles gerardosandovali*
*Apanteles aidalopezae*	Unassigned		*Apanteles isaacbermudezi*
*Apanteles albanjimenezi*	*carpatus*		*Apanteles jorgecortesi*
*Apanteles albinervis*	*leucostigmus*		*Apanteles juanvictori*
*Apanteles alejandromasisi*	Unassigned		*Apanteles juniorlopezi*
*Apanteles alejandromorai*	*alejandromorai*		*Apanteles laurenmoralesae*
*Apanteles alvarougaldei*	*leucostigmus*		*Apanteles leninguadamuzi*
*Apanteles anabellecordobae*	*anabellecordobae*		*Apanteles luiscanalesi*
*Apanteles anamarencoae*	*anamarencoae*		*Apanteles luislopezi*
*Apanteles anamartinezae*	*adrianachavarriae*		*Apanteles manuelarayai*
*Apanteles anapiedrae*	Unassigned		*Apanteles paulaixcamparijae*
*Apanteles anariasae*	*ater*		*Apanteles ronaldmurilloi*
*Apanteles andreacalvoae*	Unassigned		*Apanteles wilbertharayai*
*Apanteles angelsolisi*	*leucostigmus*		*Apanteles yolandarojasae*
*Apanteles arielopezi*	*arielopezi*		*Apanteles zeneidabolanosae*
*Apanteles balthazari*	*megathymi*	*adrianachavarriae*	*Apanteles adrianachavarriae*
*Apanteles bernardoespinozai*	*leucostigmus*		*Apanteles adrianguadamuzi*
*Apanteles bernyapui*	*bernyapui*		*Apanteles anamartinezae*
*Apanteles bettymarchenae*	Unassigned		*Apanteles felipechavarriai*
*Apanteles bienvenidachavarriae*	*bienvenidachavarriae*		*Apanteles irenecarrilloi*
*Apanteles calixtomoragai*	*calixtomoragai*		*Apanteles luiscantillanoi*
*Apanteles carloscastilloi*	*adelinamoralesae*		*Apanteles mariatorrentesae*
*Apanteles carlosguadamuzi*	*carlosguadamuzi*		*Apanteles ronaldquirosi*
*Apanteles carlosrodriguezi*	*carlosrodriguezi*		*Apanteles yilbertalvaradoi*
*Apanteles carlosviquezi*	*leucostigmus*	*adrianaguilarae*	*Apanteles adrianaguilarae*
*Apanteles carloszunigai*	*carloszunigai*		*Apanteles ivonnetranae*
*Apanteles carolinacanoae*	*anabellecordobae*		*Apanteles vannesabrenesae*
*Apanteles carpatus*	*carpatus*	*alejandromorai*	*Apanteles alejandromorai*
*Apanteles christianzunigai*	Unassigned		*Apanteles deifiliadavilae*
*Apanteles cinthiabarrantesae*	*carlosguadamuzi*		*Apanteles eulogiosequeirai*
*Apanteles ciriloumanai*	*leucostigmus*		*Apanteles fernandochavarriai*
*Apanteles coffeellae*	*coffeellae*		*Apanteles franciscoramirezi*
*Apanteles cristianalemani*	*ater*		*Apanteles freddysalazari*
*Apanteles cynthiacorderoae*	*leucostigmus*		*Apanteles gabrielagutierrezae*
*Apanteles deifiliadavilae*	*alejandromorai*		*Apanteles juancarrilloi*
*Apanteles deplanatus*	*diatraeae*		*Apanteles luisbrizuelai*
*Apanteles diatraeae*	*diatraeae*		*Apanteles luisgarciai*
*Apanteles dickyui*	*dickyui*		*Apanteles marvinmendozai*
*Apanteles didiguadamuzi*	*adelinamoralesae*		*Apanteles minornavarroi*
*Apanteles diegoalpizari*	*ater*		*Apanteles tiboshartae*
*Apanteles diegotorresi*	Unassigned	*anabellecordobae*	*Apanteles anabellecordobae*
*Apanteles diniamartinezae*	*leucostigmus*		*Apanteles carolinacanoae*
*Apanteles duniagarciae*	*anabellecordobae*		*Apanteles duniagarciae*
*Apanteles duvalierbricenoi*	*leucostigmus*		*Apanteles edwinapui*
*Apanteles edgarjimenezi*	*adelinamoralesae*		*Apanteles eldarayae*
*Apanteles edithlopezae*	*carlosguadamuzi*		*Apanteles freddyquesadai*
*Apanteles eduardoramirezi*	*dickyui*		*Apanteles guillermopereirai*
*Apanteles edwinapui*	*anabellecordobae*		*Apanteles harryramirezi*
*Apanteles eldarayae*	*anabellecordobae*		*Apanteles joseperezi*
*Apanteles eliethcantillanoae*	*leucostigmus*		*Apanteles luciariosae*
*Apanteles erickduartei*	*erickduartei*		*Apanteles manuelpereirai*
*Apanteles esthercentenoae*	*paranthrenidis*		*Apanteles marianopereirai*
*Apanteles eugeniaphilipsae*	*leucostigmus*		*Apanteles osvaldoespinozai*
*Apanteles eulogiosequeirai*	*alejandromorai*		*Apanteles ruthfrancoae*
*Apanteles federicomatarritai*	*leucostigmus*	*anamarencoae*	*Apanteles anamarencoae*
*Apanteles felipechavarriai*	*adrianachavarriae*		*Apanteles juanlopezi*
*Apanteles felixcarmonai*	*erickduartei*	*arielopezi*	*Apanteles arielopezi*
*Apanteles fernandochavarriai*	*alejandromorai*		*Apanteles mauriciogurdiani*
*Apanteles flormoralesae*	Unassigned	*ater*	*Apanteles anariasae*
*Apanteles franciscopizarroi*	*ater*		*Apanteles cristianalemani*
*Apanteles franciscoramirezi*	*alejandromorai*		*Apanteles diegoalpizari*
*Apanteles freddyquesadai*	*anabellecordobae*		*Apanteles franciscopizarroi*
*Apanteles freddysalazari*	*alejandromorai*		*Apanteles galleriae*
*Apanteles fredi*	*diatraeae*		*Apanteles impiger*
*Apanteles gabrielagutierrezae*	*alejandromorai*		*Apanteles jairomoyai*
*Apanteles galleriae*	*ater*		*Apanteles josejaramilloi*
*Apanteles garygibsoni*	Unassigned		*Apanteles leucopus*
*Apanteles gerardobandoi*	*leucostigmus*	*bernyapui*	*Apanteles bernyapui*
*Apanteles gerardosandovali*	*adelinamoralesae*		*Apanteles javiersihezari*
*Apanteles gladysrojasae*	*leucostigmus*		*Apanteles raulacevedoi*
*Apanteles glenriverai*	*glenriverai*		*Apanteles victorbarrantesi*
*Apanteles gloriasihezarae*	*carlosrodriguezi*	*bienvenidachavarriae*	*Apanteles bienvenidachavarriae*
*Apanteles guadaluperodriguezae*	*guadaluperodriguezae*		*Apanteles josecalvoi*
*Apanteles guillermopereirai*	*anabellecordobae*		*Apanteles marisolarroyoae*
*Apanteles harryramirezi*	*anabellecordobae*	*calixtomoragai*	*Apanteles calixtomoragai*
*Apanteles hazelcambroneroae*	*leucostigmus*		*Apanteles manuelriosi*
*Apanteles hectorsolisi*	Unassigned		*Apanteles petronariosae*
*Apanteles humbertolopezi*	*humbertolopezi*	*carlosguadamuzi*	*Apanteles carlosguadamuzi*
*Apanteles impiger*	*ater*		*Apanteles cinthiabarrantesae*
*Apanteles inesolisae*	*leucostigmus*		*Apanteles edithlopezae*
*Apanteles insularis*	*ronaldgutierrezi*		*Apanteles javiercontrerasi*
*Apanteles irenecarrilloi*	*adrianachavarriae*		*Apanteles jesusbrenesi*
*Apanteles isaacbermudezi*	*adelinamoralesae*		*Apanteles williamcamposi*
*Apanteles isidrochaconi*	*isidrochaconi*	*carlosrodriguezi*	*Apanteles carlosrodriguezi*
*Apanteles isidrovillegasi*	Unassigned		*Apanteles gloriasihezarae*
*Apanteles ivonnetranae*	*adrianaguilarae*		*Apanteles robertoespinozai*
*Apanteles jairomoyai*	*ater*	*carloszunigai*	*Apanteles carloszunigai*
*Apanteles javiercontrerasi*	*carlosguadamuzi*		*Apanteles yeissonchavesi*
*Apanteles javierobandoi*	*javierobandoi*	*carpatus*	*Apanteles albanjimenezi*
*Apanteles javiersihezari*	*bernyapui*		*Apanteles carpatus*
*Apanteles jesusbrenesi*	*carlosguadamuzi*		*Apanteles rhomboidalis*
*Apanteles jesusugaldei*	*leucostigmus*		*Apanteles robertovargasi*
*Apanteles jimmychevezi*	*samarshalli*		*Apanteles rolandoramosi*
*Apanteles johanvargasi*	*leucostigmus*	*coffeellae*	*Apanteles coffeellae*
*Apanteles jorgecortesi*	*adelinamoralesae*		*Apanteles laurahuberae*
*Apanteles jorgehernandezi*	*leucostigmus*		*Apanteles lisabearssae*
*Apanteles josecalvoi*	*bienvenidachavarriae*		*Apanteles mariaguevarae*
*Apanteles josecortesi*	*leucostigmus*	*diatraeae*	*Apanteles deplanatus*
*Apanteles josediazi*	Unassigned		*Apanteles diatraeae*
*Apanteles josejaramilloi*	*ater*		*Apanteles fredi*
*Apanteles josemonteroi*	*leucostigmus*	*dickyui*	*Apanteles dickyui*
*Apanteles joseperezi*	*anabellecordobae*		*Apanteles eduardoramirezi*
*Apanteles joserasi*	*joserasi*	*erickduartei*	*Apanteles erickduartei*
*Apanteles juanapui*	*isidrochaconi*		*Apanteles felixcarmonai*
*Apanteles juancarrilloi*	*alejandromorai*		*Apanteles luishernandezi*
*Apanteles juangazoi*	*javierobandoi*		*Apanteles milenagutierrezae*
*Apanteles juanhernandezi*	Unassigned		*Apanteles ronaldcastroi*
*Apanteles juanlopezi*	*anamarencoae*	*glenriverai*	*Apanteles glenriverai*
*Apanteles juanmatai*	*leucostigmus*		*Apanteles pablovasquezi*
*Apanteles juanvictori*	*adelinamoralesae*	*guadaluperodriguezae*	*Apanteles guadaluperodriguezae*
*Apanteles juliodiazi*	Unassigned		*Apanteles marcobustosi*
*Apanteles juniorlopezi*	*adelinamoralesae*	*humbertolopezi*	*Apanteles humbertolopezi*
*Apanteles keineraragoni*	*keineraragoni*		*Apanteles pablotranai*
*Apanteles laurahuberae*	*coffeellae*	*isidrochaconi*	*Apanteles isidrochaconi*
*Apanteles laurenmoralesae*	*adelinamoralesae*		*Apanteles juanapui*
*Apanteles leninguadamuzi*	*adelinamoralesae*	*javierobandoi*	*Apanteles javierobandoi*
*Apanteles leonelgarayi*	Unassigned		*Apanteles juangazoi*
*Apanteles leucopus*	*ater*	*joserasi*	*Apanteles joserasi*
*Apanteles leucostigmus*	*leucostigmus*	*keineraragoni*	*Apanteles keineraragoni*
*Apanteles lilliammenae*	*leucostigmus*		*Apanteles ronaldnavarroi*
*Apanteles lisabearssae*	*coffeellae*	*leucostigmus*	*Apanteles albinervis*
*Apanteles luciariosae*	*anabellecordobae*		*Apanteles alvarougaldei*
*Apanteles luisbrizuelai*	*alejandromorai*		*Apanteles angelsolisi*
*Apanteles luiscanalesi*	*adelinamoralesae*		*Apanteles bernardoespinozai*
*Apanteles luiscantillanoi*	*adrianachavarriae*		*Apanteles carlosviquezi*
*Apanteles luisgarciai*	*alejandromorai*		*Apanteles ciriloumanai*
*Apanteles luisgaritai*	Unassigned		*Apanteles cynthiacorderoae*
*Apanteles luishernandezi*	*erickduartei*		*Apanteles diniamartinezae*
*Apanteles luislopezi*	*adelinamoralesae*		*Apanteles duvalierbricenoi*
*Apanteles luisvargasi*	Unassigned		*Apanteles eliethcantillanoae*
*Apanteles luzmariaromeroae*	*leucostigmus*		*Apanteles eugeniaphilipsae*
*Apanteles manuelarayai*	*adelinamoralesae*		*Apanteles federicomatarritai*
*Apanteles manuelpereirai*	*anabellecordobae*		*Apanteles gerardobandoi*
*Apanteles manuelriosi*	*calixtomoragai*		*Apanteles gladysrojasae*
*Apanteles manuelzumbadoi*	*leucostigmus*		*Apanteles hazelcambroneroae*
*Apanteles marcobustosi*	*guadaluperodriguezae*		*Apanteles inesolisae*
*Apanteles marcogonzalezi*	Unassigned		*Apanteles jesusugaldei*
*Apanteles marcovenicioi*	*leucostigmus*		*Apanteles johanvargasi*
*Apanteles mariachavarriae*	*leucostigmus*		*Apanteles jorgehernandezi*
*Apanteles mariaguevarae*	*coffeellae*		*Apanteles josecortesi*
*Apanteles marialuisariasae*	Unassigned		*Apanteles josemonteroi*
*Apanteles mariamendezae*	Unassigned		*Apanteles juanmatai*
*Apanteles marianopereirai*	*anabellecordobae*		*Apanteles leucostigmus*
*Apanteles mariatorrentesae*	*adrianachavarriae*		*Apanteles lilliammenae*
*Apanteles marisolarroyoae*	*bienvenidachavarriae*		*Apanteles luzmariaromeroae*
*Apanteles marisolnavarroae*	*marisolnavarroae*		*Apanteles manuelzumbadoi*
*Apanteles marvinmendozai*	*alejandromorai*		*Apanteles marcovenicioi*
*Apanteles mauriciogurdiani*	*arielopezi*		*Apanteles mariachavarriae*
*Apanteles megastidis*	*paranthrenidis*		*Apanteles minorcarmonai*
*Apanteles megathymi*	*megathymi*		*Apanteles pabloumanai*
*Apanteles milenagutierrezae*	*erickduartei*		*Apanteles randallgarciai*
*Apanteles minorcarmonai*	*leucostigmus*		*Apanteles raulsolorsanoi*
*Apanteles minornavarroi*	*alejandromorai*		*Apanteles ricardocaleroi*
*Apanteles monicachavarriae*	Unassigned		*Apanteles rodrigogamezi*
*Apanteles oscarchavezi*	Unassigned		*Apanteles ronaldzunigai*
*Apanteles osvaldoespinozai*	*anabellecordobae*		*Apanteles rostermoragai*
*Apanteles pablotranai*	*humbertolopezi*		*Apanteles sergioriosi*
*Apanteles pabloumanai*	*leucostigmus*		*Apanteles sigifredomarini*
*Apanteles pablovasquezi*	*glenriverai*		*Apanteles wadyobandoi*
*Apanteles paranthrenidis*	*paranthrenidis*	*marisolnavarroae*	*Apanteles marisolnavarroae*
*Apanteles paulaixcamparijae*	*adelinamoralesae*		*Apanteles randallmartinezi*
*Apanteles petronariosae*	*calixtomoragai*	*megathymi*	*Apanteles balthazari*
*Apanteles randallgarciai*	*leucostigmus*		*Apanteles megathymi*
*Apanteles randallmartinezi*	*marisolnavarroae*	*paranthrenidis*	*Apanteles esthercentenoae*
*Apanteles raulacevedoi*	*bernyapui*		*Apanteles megastidis*
*Apanteles raulsolorsanoi*	*leucostigmus*		*Apanteles paranthrenidis*
*Apanteles rhomboidalis*	*carpatus*		*Apanteles thurberiae*
*Apanteles ricardocaleroi*	*leucostigmus*	*ronaldgutierrezi*	*Apanteles insularis*
*Apanteles robertmontanoi*	Unassigned		*Apanteles ronaldgutierrezi*
*Apanteles robertoespinozai*	*carlosrodriguezi*	*samarshalli*	*Apanteles jimmychevezi*
*Apanteles robertovargasi*	*carpatus*		*Apanteles samarshalli*
*Apanteles rodrigogamezi*	*leucostigmus*	Unassigned	*Apanteles aichagirardae*
*Apanteles rogerblancoi*	Unassigned	Unassigned	*Apanteles aidalopezae*
*Apanteles rolandoramosi*	*carpatus*	Unassigned	*Apanteles alejandromasisi*
*Apanteles rolandovegai*	Unassigned	Unassigned	*Apanteles anapiedrae*
*Apanteles ronaldcastroi*	*erickduartei*	Unassigned	*Apanteles andreacalvoae*
*Apanteles ronaldgutierrezi*	*ronaldgutierrezi*	Unassigned	*Apanteles bettymarchenae*
*Apanteles ronaldmurilloi*	*adelinamoralesae*	Unassigned	*Apanteles christianzunigai*
*Apanteles ronaldnavarroi*	*keineraragoni*	Unassigned	*Apanteles diegotorresi*
*Apanteles ronaldquirosi*	*adrianachavarriae*	Unassigned	*Apanteles flormoralesae*
*Apanteles ronaldzunigai*	*leucostigmus*	Unassigned	*Apanteles garygibsoni*
*Apanteles rosibelelizondoae*	Unassigned	Unassigned	*Apanteles hectorsolisi*
*Apanteles rostermoragai*	*leucostigmus*	Unassigned	*Apanteles isidrovillegasi*
*Apanteles ruthfrancoae*	*anabellecordobae*	Unassigned	*Apanteles josediazi*
*Apanteles samarshalli*	*samarshalli*	Unassigned	*Apanteles juanhernandezi*
*Apanteles sergiocascantei*	Unassigned	Unassigned	*Apanteles juliodiazi*
*Apanteles sergioriosi*	*leucostigmus*	Unassigned	*Apanteles leonelgarayi*
*Apanteles sigifredomarini*	*leucostigmus*	Unassigned	*Apanteles luisgaritai*
*Apanteles thurberiae*	*paranthrenidis*	Unassigned	*Apanteles luisvargasi*
*Apanteles tiboshartae*	*alejandromorai*	Unassigned	*Apanteles marcogonzalezi*
*Apanteles vannesabrenesae*	*adrianaguilarae*	Unassigned	*Apanteles marialuisariasae*
*Apanteles victorbarrantesi*	*bernyapui*	Unassigned	*Apanteles mariamendezae*
*Apanteles vulgaris*	Unassigned	Unassigned	*Apanteles monicachavarriae*
*Apanteles wadyobandoi*	*leucostigmus*	Unassigned	*Apanteles oscarchavezi*
*Apanteles waldymedinai*	Unassigned	Unassigned	*Apanteles robertmontanoi*
*Apanteles wilbertharayai*	*adelinamoralesae*	Unassigned	*Apanteles rogerblancoi*
*Apanteles williamcamposi*	*carlosguadamuzi*	Unassigned	*Apanteles rolandovegai*
*Apanteles yeissonchavesi*	*carloszunigai*	Unassigned	*Apanteles rosibelelizondoae*
*Apanteles yilbertalvaradoi*	*adrianachavarriae*	Unassigned	*Apanteles sergiocascantei*
*Apanteles yolandarojasae*	*adelinamoralesae*	Unassigned	*Apanteles vulgaris*
*Apanteles zeneidabolanosae*	*adelinamoralesae*	Unassigned	*Apanteles waldymedinai*

### *adelinamoralesae* species-group

This group comprises 19 species, defined by having ovipositor sheaths usually >1.2 × metatibia length; femora mostly (except for posterior half of profemur) dark brown to black; tegula yellow-white and humeral complex half yellow-white, half dark brown; and mediotergite 2 width at posterior margin at least 2.9 × its median length. The group is supported by the Bayesian molecular analysis (PP: 0.5 for the whole group, most of its species have PP between 0.9–1.0; Fig. 1). Hosts: Elachistidae and on two occasions, Pyralidae. All described are from ACG, but many species attacking elachistids in Mesoamerica are likely to be part of this group.

#### Key to species of the *adelinamoralesae* group

**Table:** 

1	Metatibia entirely or mostly (>0.7 posteriorly) dark brown to black, with yellow-orange coloration restricted to anterior 0.2 or less (as in Figs 4a, 6c, 12c, a, 14a)	2
–	Metatibia yellow-orange at least on anterior 0.5 (usually more), with dark brown to black coloration restricted to posterior 0.5 or less (as in Figs 7c, 9a, c, 18c)	11
2(1)	Ovipositor sheaths 1.0–1.1 × as long as metatibia	3
–	Ovipositor sheaths 1.3–1.6 × as long as metatibia	5
3(2)	T1 parallel-sided for 0.7–0.8 of its length, then narrowing posteriorly so mediotergite anterior width >1.1 × posterior width; T2 width at posterior margin 4.4 × its medial length (Fig. 21h); metafemur 2.7 × as long as wide (Fig. 21c)	*Apanteles yolandarojasae* Fernández-Triana, sp. n.
–	T1 slightly widening from anterior margin to 0.7–0.8 mediotergite length (where maximum width is reached), then narrowing towards posterior margin, with widest part of tergite (centrally) being 1.2 × that of base and/or apex; T2 width at posterior margin at most 3.1 × its medial length (as in Fig. 12f); metafemur at least 2.9 × as long as wide (Figs 12c, 17c)	4
4(3)	Flagellomerus 2 2.4 × as long as wide; flagellomerus 14 1.3 × as long as wide; metafemur 3.3 × as long as wide; fore wing with vein 2RS 1.9 × as long as vein 2M	*Apanteles juniorlopezi* Fernández-Triana, sp. n.
–	Flagellomerus 2 2.9 × as long as wide; flagellomerus 14 1.7 × as long as wide; metafemur 2.9 × as long as wide; fore wing with vein 2RS 1.1 × as long as vein 2M	*Apanteles manuelarayai* Fernández-Triana, sp. n. (N = 5)
5(2)	Mesoscutellar disc mostly smooth (Figs 4e, 22g); tarsal claws simple	6
–	Mesoscutellar disc mostly punctured, or at least with punctures near margins; tarsal claws with single basal spine-like seta	7
6(5)	Metatibia with inner spur 2.0 × as long as outer spur; flagellomerus 2 2.2 × as long as wide; T1 2.0 × as long as wide at posterior margin; fore wing with vein r 1.2 × as long as vein 2RS, and vein 2RS 1.5 × as long as vein 2M [Cocoons: Gregarious. Hosts: *Lethata trochalosticta*]	*Apanteles zeneidabolanosae* Fernández-Triana, sp. n.
–	Metatibia with inner spur 1.3 × as long as outer spur; flagellomerus 2 2.9 × as long as wide; T1 2.6 × as long as wide at posterior margin; fore wing with vein r 1.8 × as long as vein 2RS, and vein 2RS 2.1 × as long as vein 2M [Cocoons: Solitary. Hosts: *Antaeotricha* sp., *Stenoma* sp.]	*Apanteles adelinamoralesae* Fernández-Triana, sp. n. (N = 1)
7(5)	Fore wing with vein r at most 1.4 × as long as vein 2RS; metafemur 3.4 × as long as wide; interocellar distance usually 1.5 × as long as ocellus diameter (rarely up to 1.7 ×); ovipositor sheaths at least 1.6 × as long as metatibia (very rarely 1.5 ×) (Fig. 6c); protibia completely yellow (Fig. 6a)	*Apanteles didiguadamuzi* Fernández-Triana, sp. n.
–	Fore wing with vein r at least 1.6 × as long as vein 2RS (usually more than 1.7 ×); metafemur at most 3.3 × as long as wide (usually much less); interocellar distance usually more than 1.8 × as long as ocellus diameter (rarely 1.7 ×); ovipositor sheaths at most 1.4 × as long as metatibia, usually less (very rarely 1.5 ×) (Figs 8a, 11a, 14c, 16a, c); protibia with anterior 0.5 yellow, posterior 0.5 dark brown to black (As in Fig. 16a)	8
8(7)	T2 width at posterior margin at most 2.9 × its length (Fig. 8f); metafemur length 3.3 × its width	*Apanteles gerardosandovali* Fernández-Triana, sp. n. (N = 5)
–	T2 width at posterior margin at least 3.2 × its length (usually 3.5 ×) (Figs 11f, 14f, 16f); metafemur length at most 3.0 × its width	9
9(8)	T2 mostly smooth; T2 width at posterior margin at most 3.2 × its length (Figs 11f)	*Apanteles juanvictori* Fernández-Triana, sp. n.
–	T2 usually with some sculpture; T2 width at posterior margin at least 3.5 × its length (Figs 14f, 16f)	10
10(9)	Fore wing with vein r 2.3 × as long as vein 2RS, and vein 2RS 1.2 × as long as vein 2M; mesoscutellar disc with punctures near margins, central part mostly smooth [Hosts: *Anadasmus* sp., *Cerconota* sp.]	*Apanteles luislopezi* Fernández-Triana, sp. n.
–	Fore wing with vein r 1.8 × as long as vein 2RS, and vein 2RS 1.5 × as long as vein 2M; mesoscutellar disc mostly punctured [Hosts: *Stenoma byssina*]	*Apanteles leninguadamuzi* Fernández-Triana, sp. n.
11(1)	Ovipositor sheaths 1.0 × as long as metatibia (rarely 1.1 ×) (Fig. 9c)	*Apanteles isaacbermudezi* Fernández-Triana, sp. n. (N = 4)
–	Ovipositor sheaths 1.3-1.6 × as long as metatibia (rarely 1.2 ×) (Figs 5a, 7a, 10a, 13a)	12
12(11)	T2 width at posterior margin at most 2.9 × its length, ***if*** rarely 3.0–3.2 × ***then*** T1 length at least 2.1 × its width at posterior margin ***and*** fore wing vein 2RS as long as vein 2M ***and*** vein 2M as long as vein (RS+M)b	13
–	T2 width at posterior margin at least 3.2 × its length (usually much more), ***and/or*** T1 length less than 2.0 × its width at posterior margin ***and/or*** fore wing vein 2RS longer than vein 2M ***and/or*** vein 2M shorter than vein (RS+M)b	15
13(12)	Tarsal claws simple; fore wing with vein r 1.6 × as long as vein 2Rs, vein 2RS 1.6 × as long as vein 2M, and vein 2M 0.6 × as long as vein (RS+M)b	*Apanteles edgarjimenezi* Fernández-Triana, sp. n.
–	Tarsal claws with single basal spine-like seta; fore wing with vein r at least 1.7 × as long as vein 2Rs, vein 2RS at most 1.3 × as long as vein 2M, and vein 2M at least 0.9 × as long as vein (RS+M)b	14
14(13)	Interocellar distance at most 2.0 × ocellus diameter (usually less than 1.8 ×); mesoscutellar disc with punctures near the margin, central part mostly smooth; T1 length 2.1 × its width at posterior margin; ovipositor sheaths usually 1.5–1.6 × as long as metatibia length; ***if*** very rarely ovipositor sheaths 1.3 × as long as metatibia length, ***then*** body length and fore wing length 2.0 mm (otherwise body and fore wing length 2.9–3.3 mm)	*Apanteles carloscastilloi* Fernández-Triana, sp. n.
–	Interocellar distance 2.1 × ocellus diameter; mesoscutellar disc mostly punctured; T1 length 1.7 × its width at posterior margin; ovipositor sheaths usually 1.3–1.4 × as long as metatibia length; body length 2.9–3.0 mm; fore wing length 3.1–3.4 mm	*Apanteles jorgecortesi* Fernández-Triana, sp. n.
15(12)	Ovipositor sheaths 1.6 × as long as metatibia; flagellomerus 2 2.5 × as long as wide; metatibial inner spur 1.7 × as long as outer spur	*Apanteles laurenmoralesae* Fernández-Triana, sp. n.
–	Ovipositor sheaths at most 1.4 × as long as metatibia; flagellomerus 2 at least 2.6 × as long as wide (usually 2.9 × or more); metatibial inner spur at most 1.5 × as long as outer spur (usually less than 1.4 ×)	16
16(15)	T2 fully sculptured; T2 width at posterior margin 4.6 × its length (Fig. 20g); body length 3.2 mm; fore wing length 3.4 mm	*Apanteles wilbertharayai* Fernández-Triana, sp. n. (N = 1)
–	T2 mostly smooth, at most with weak and sparse punctures laterally near posterior margin; T2 width at posterior margin at most 3.6 × its length (Figs 15f, 18g, 19g); body length and fore wing length usually less than 3.0 mm (***if*** rarely over 3.2 mm, ***then*** T2 width at posterior margin at most 3.2 × its length)	17
17(16)	Interocellar distance 2.2 × as long as posterior ocellus diameter; mesoscutellar disc with punctures near the margin, central part mostly smooth	*Apanteles luiscanalesi* Fernández-Triana, sp. n.
–	Interocellar distance 1.8 × as long as posterior ocellus diameter; mesoscutellar disc mostly punctured	18
18(17)	T1 parallel-sided; T2 with some sculpture, mostly near posterior margin; fore wing with vein 2RS 1.0 × as long as vein 2M; outer margin of hypopygium extending about the same length of last tergites	*Apanteles paulaixcamparijae* Fernández-Triana, sp. n.
–	T1 slightly widening from anterior margin to 0.7–0.8 mediotergite length (where maximum width is reached), then narrowing towards posterior margin; T2 mostly smooth; fore wing with vein 2RS 1.5 × as long as vein 2M; outer margin of hypopygium clearly extending beyond last tergites	*Apanteles ronaldmurilloi* Fernández-Triana, sp. n.

### *adrianachavarriae* species-group

This group comprises nine species with mesofemur, metafemur and all or most of metatibia dark brown to black; pterostigma with thin brown borders, centrally white or translucid; and mediotergite 1 with strong longitudinal striations. The group is likely to be artificial, at least partially, and it may end being part of a larger group (including the current *joserasi javierobandoi* groups). However, morphology, host data and DNA barcoding (Fig. 1), provide some support for most of its component species; and it seems better to keep this group separated for the time being. Hosts: Attevidae, Crambidae, Elachistidae, and Tortricidae. One of the species within this group, *Apanteles felipechavarriai*, is only known from a female in poor condition and cannot be keyed out using morphology alone beyond couplet 3 of the key below, thus barcoding data was used to distinguish that species from the remainder. All species described in this group are from ACG.

#### Key to species of the *adrianachavarriae* group

**Table:** 

1	Metatibia with black coloration at most on posterior 0.3–0.5 (Figs 29a, c) [Hosts: Crambidae, *Leucochromodes* sp.]	*Apanteles mariatorrentesae* Fernández-Triana, sp. n. (N = 2)
–	Metatibia almost completely black, except for anterior 0.2 or less which is yellow (as in Figs 23c, 25d, 27c, 28a, c, 30a, 31c)	2
2(1)	T1 length at least 2.1 × its width at posterior margin ***and*** T2 width at posterior margin at most 4.0 × its length (***if*** rarely T1 length 1.9 × its width at posterior margin, ***then*** T2 width at posterior margin less than 3.6 × its length)	3
–	T1 length at most 1.7 × (usually 1.6 × or less) its width at posterior margin ***and*** T2 width at posterior margin at least 4.3 × (usually 4.4 × or more) its length	7
3(2)	A total of 18 diagnostic characters in the barcoding region: 81 G, 82 C, 99 A, 129 C, 136 A, 144 T, 189 C, 237 T, 246 C, 264 A, 327 C, 348 T, 357 C, 363 T, 387 A, 392 T, 502 C, 573 C [Hosts: Crambidae, *Eulepte concordalis*]	*Apanteles felipechavarriai* Fernández-Triana, sp. n. (N = 1)
–	Barcoding region with 18 diagnostic nucleotides at positions: 81 A, 82 T, 99 T, 129 T, 136 T, 144 A, 189 T, 237 C, 246 T, 264 T or C, 327 T, 348 C, 357 T, 363 A, 387 T, 392 A or C, 502 T, 573 A or T	4
4(3)	Ovipositor sheaths 1.4 × as long as metatibia (Fig. 23c); T1 length at most 1.9 × its width at posterior margin [Hosts: Tortricidae, *Episimus* sp.; Yponomeutidae, *Atteva zebra*]	*Apanteles adrianachavarriae* Fernández-Triana, sp. n.
–	Ovipositor sheaths at most 1.2 × as long as metatibia; T1 length at least 2.1 × its width at posterior margin	5
5(4)	Ovipositor sheaths length 0.8–0.9 × metatibia length (Fig. 30a); T2 width at posterior margin at most 3.7 × its length; body length 2.8 mm; fore wing length 2.8 mm [Hosts: Crambidae, *Pilocrocis xanthozonalis*, Tortricidae, *Amorbia productana*]	*Apanteles ronaldquirosi* Fernández-Triana, sp. n. (N = 3)
–	Ovipositor sheaths length 1.0–1.2 × metatibia length (Figs 27c, 28a); T2 width at posterior margin at least 3.8 × its length; body length 2.2–2.4 mm (rarely 2.5 mm); fore wing length 2.4–2.6 mm	6
6(5)	Fore wing with vein r 1.7 × as long as vein 2RS; flagellomerus 2 2.9 × as long as wide; flagellomerus 14 1.7 × as long as wide [Hosts: Crambidae, *Asturodes fimbriauralis*]	*Apanteles irenecarrilloae* Fernández-Triana, sp. n. (N = 2)
–	Fore wing with vein r at most 1.4 × as long as vein 2RS; flagellomerus 2 3.1 × as long as wide; flagellomerus 14 at most 1.5 × as long as wide [Hosts: Crambidae, *Diacme* sp.]	*Apanteles luiscantillanoi* Fernández-Triana, sp. n. (N = 3)
7(2)	Ovipositor sheaths at most 0.8 × metatibia length (Figs 25a, d) [Hosts: Yponomeutidae, *Atteva* spp.]	*Apanteles anamartinesae* Fernández-Triana, sp. n.
–	Ovipositor sheaths at least 1.0 × metatibia length (Figs 24a, b, 31a, c)	8
8(7)	T1 length 1.7 × its width at posterior margin; T2 width at posterior margin 4.4 × its length [Hosts: Elachistidae, *Antaeotricha similis*, *Stenoma* sp.]	*Apanteles adrianguadamuzi* Fernández-Triana, sp. n. (N = 2)
–	T1 length 1.5 × its width at posterior margin; T2 width at posterior margin 5.2 × its length [Hosts: Tortricidae, *Episimus* spp.]	*Apanteles yilbertalvaradoi* Fernández-Triana, sp. n. (N = 2)

### *adrianaguilarae* species-group

This group comprises three species characterized by extensive yellow-orange coloration, ocular-ocellar line 2.5 × posterior ocellus diameter, and fore wing with vein 2M as long as vein (RS+M)b. The group is strongly supported by the Bayesian molecular analysis (PP: 1.0, Fig. 1). Hosts: Tortricidae. All the described species are from ACG.

#### Key to species of the *adrianaguilarae* group

**Table:** 

1	Ovipositor sheaths 0.9–1.0 × metatibia length (Figs 33a, c); fore wing with vein r 1.1 × as long as vein 2RS, vein 2RS 2.0 × as long as vein 2M, and vein 2M 0.7 × as long as vein (RS+M)b; pterostigma 3.6 × as long as wide; metafemur at least 3.1 × as long as wide	*Apanteles ivonnetranae* Fernández-Triana, sp. n.
–	Ovipositor sheaths at most 0.6 × metatibia length (Figs 32d, 34c); fore wing with vein r at least 1.4 × as long as vein 2RS, vein 2RS at most 1.2 × as long as vein 2M, and vein 2M at least 1.0 × as long as vein (RS+M)b; pterostigma at most 3.1 × as long as wide; metafemur at most 2.9 × as long as wide	2
2(1)	Metafemur mostly yellow, at most brown on posterior 0.3 (usually less) (Figs 32a, d); interocellar distance 2.2 × posterior ocellus diameter; T2 width at posterior margin 4.5 × its length; fore wing with vein 2RS 1.2 × vein 2M	*Apanteles adrianaguilarae* Fernández-Triana, sp. n.
–	Metafemur mostly brown, at most yellow on anterior 0.4 (usually less) (Figs 34a, d); interocellar distance 1.8 × posterior ocellus diameter; T2 width at posterior margin 3.7 × its length; fore wing with vein 2RS 0.9 × vein 2M	*Apanteles vannesabrenesae* Fernández-Triana, sp. n.

### *alejandromorai* species-group

This group comprises 13 species which are unique among all Mesoamerican *Apanteles* in having an almost quadrate mediotergite 2 and a very long ovipositor. Both the Bayesian and neighbour joining trees (Figs 1, 2) have the species of this group in two separate clusters, each of them strongly supported (PP: 0.99 and 1.0 respectively, Fig. 1). Whenever the wasp biology is known, all are solitary parasitoids, with individual, white cocoons attached to the leaves where the caterpillar was feeding. Hosts: Elachistidae and Gelechiidae. All described species are from ACG, although we have seen undescribed species from other Neotropical areas.

#### Key to species of the *alejandromorai* group

**Table:** 

1	Meso- and metafemora yellow (metafemora may have small, dark spot on posterior 0.1); metatibia mostly yellow, at most with dark brown to black spot in posterior 0.2 or less (rarely 0.3) of its length (Figs 39a, c, g, 42a, c, 45a)	2
–	Mesofemur (partially or completely) and metafemur (completely) dark brown to black; metatibia usually brown to black in posterior 0.3-0.5 (rarely 0.2) of its length (Figs 38a, c, e, 40a, c, 41a, c, 43a, c, 44a, 46a, 47a, c, 48a, 49a, c, 50a, c)	4
2(1)	Ovipositor sheaths 1.2 × metatibia length (Figs 42a, c); body and fore wing length at most 3.2 mm; ocular-ocellar line 2.6 × posterior ocellus diameter; interocellar distance 2.2 × posterior ocellus diameter [Hosts: Elachistidae, *Antaeotricha*]	*Apanteles franciscoramirezi* Fernández-Triana, sp. n. (N = 1)
–	Ovipositor sheaths at least 1.7 × metatibia length (Figs 39a, c, 45a, c); body and fore wing length at least 3.4 mm; ocular-ocellar line at most 1.9 × posterior ocellus diameter; interocellar distance at most 1.9 × posterior ocellus diameter; terostigma completely dark brown (at most with small pale spot at base); most of fore wing veins brown	3
3(2)	Ovipositor sheaths 1.8 mm long; fore wing length 1.9 × as long as ovipositor sheaths length [Hosts: *Antaeotricha radicalis* and other Elachistidae feeding on Melastomataceae]	*Apanteles deifiliadavilae* Fernández-Triana, sp. n. (N = 1)
–	Ovipositor sheaths 2.1–2.3 mm long; fore wing length 1.6–1.7 × as long as ovipositor sheaths length [Host: *Antaeotricha* spp. ]	*Apanteles juancarrilloi* Fernández-Triana, sp. n. (N = 5)
4(1)	All trochantelli, profemur, tegula and humeral complex entirely yellow (Figs 49a, c, g); mesofemur partially yellow, especially dorsally; metafemur white to yellow on anterior 0.1–0.2, giving the appareance of a light anellus (Fig. 49c)	*Apanteles tiboshartae* Fernández-Triana, sp. n.
–	All trochantelli and part of profemur (basal 0.2–0.5) dark brown to black, tegula yellow, humeral complex half brown, half yellow; meso- and metafemur completely dark brown to black (mesofemur rarely with 0.2 or less yellow)	5
5(4)	Ovipositor sheaths at most 1.6 × as long as metatibia length	6
–	Ovipositor sheaths at least 1.8 × as long as metatibia length	9
6(5)	Pterostigma mostly dark brown with small, paler area centrally (Fig. 44b); T1 length at least 3.0 × its width at posterior margin	*Apanteles gabrielagutierrezae* Fernández-Triana, sp. n. (N = 2)
–	Pterostigma mostly pale (yellow-white) or transparent, with only thin borders brown (Figs 43b, 46b, 47b); T1 length at most 2.8 × its width at posterior margin	7
7(6)	Body length and fore wing length 3.0 mm; T1 width at posterior margin 0.6 × width at anterior margin [Hosts: Choreutidae, *Tortyra*; Elachistidae, *Anacampsis*]	*Apanteles luisgarciai* Fernández-Triana, sp. n. (N = 1)
–	Body length and fore wing length at least 3.3 mm; T1 width at posterior margin 0.8 × width at anterior margin [Hosts: Elachistidae, *Antaeotricha* spp.]	8
8(7)	Scutoscutellar sulcus with 8 pits; fore wing with vein r 2.2 × vein 2RS, and vein 2RS 1.3 × vein 2M; T1 length 2.7 × its width at posterior margin; flagellomerus 2 2.9 × as long as wide; flagellomerus 14 1.8 × as long as wide; ocular-ocellar line 2.3 × posterior ocellus diameter	*Apanteles luisbrizuelai* Fernández-Triana, sp. n. (N = 1)
–	Scutoscutellar sulcus with at least 11 pits; fore wing with vein r 1.4 × vein 2RS, and vein 2RS 1.6 × vein 2M; T1 length 2.3 × its width at posterior margin; flagellomerus 2 2.6 × as long as wide; flagellomerus 14 1.5 × as long as wide; ocular-ocellar line 2.6 × posterior ocellus diameter	*Apanteles freddysalazari* Fernández-Triana, sp. n. (N = 2)
9(5)	Pterostigma mostly dark brown with small, paler area centrally (Fig. 38b); fore wing with vein 2RS 1.9 × vein 2M; flagellomerus 2 3.0 × as long as wide	*Apanteles alejandromorai* Fernández-Triana, sp. n.
–	Pterostigma mostly pale (yellow-white) or transparent, with only thin borders brown (Figs 40b, 41b, 48b, 50b); fore wing with vein 2RS at most 1.6 × vein 2M (usually much less); flagellomerus 2 at most 2.8 × as long as wide	10
10(9)	Metatibia mostly orange, with posterior 0.2 light brown (Figs 40a, c); flagellomerus 14 2.0 × as long as wide [Elachistidae]	*Apanteles eulogiosequeirai* Fernández-Triana,sp. n. (N = 1)
–	Metatibia with posterior 0.4–0.5 dark brown to black (Figs 41c, 48a, 50c); flagellomerus 14 at most 1.7 × as long as wide [Elachistidae]	11
11(10)	T1 length 2.2 × its width at posterior margin; T2 width at posterior margin 2.2 × its length; metafemur 3.2–3.3 × as long as wide [Elachistidae]	*Apanteles minornavarroi* Fernández-Triana, sp. n.
–	T1 length at least 2.4 × its width at posterior margin; T2 width at posterior margin at most 1.9 × its length; metafemur 2.9–3.1 × as long as wide [Elachistidae]	12
12(11)	T1 length 2.4 × its width at posterior margin; fore wing with vein r at least 2.3 × vein 2RS, vein 2RS 1.1 × vein 2M, and pterostigma 3.2 × as long as wide [Elachistidae]	*Apanteles marvinmendozai* Fernández-Triana, sp. n. (N = 1)
–	T1 length 2.9 × its width at posterior margin; fore wing with vein r 1.8 × vein 2RS, vein 2RS 1.5 × vein 2M, and pterostigma 3.8 × as long as wide [Elachistidae]	*Apanteles fernandochavarriai* Fernández-Triana, sp. n. (N = 4)

### *anabellecordobae* species-group

This group comprises 14 species and is defined by the hypopygium either unfolded or with a relatively wide and translucid fold with none or very few (1-3) pleats only in the outermost area of fold. The species have a thick ovipositor (as thick as or thicker than width of median flagellomerus), with anterior width 3.0-5.0 × its posterior width beyond the constriction. The group is strongly supported by the Bayesian molecular analysis (PP: 1.0, Fig. 1). Hosts: Hesperiidae: Eudaminae, Hesperiinae, and Pyrginae; mostly gregarious parasitoids of leaf-rolling caterpillars (only two species are solitary parasitoids, with molecular data suggesting they form a sub-group on its own). All described species are from ACG, although we have seen numerous undescribed species from other Neotropical areas.

#### Key to species of the *anabellecordobae* group

**Table:** 

1	Hypopygium without a median fold, with 0 or, at most, 1 small pleat visible (Figs 51c, 54c, 56c, 63c)	2
–	Hypopygium with a median fold and a few (1–3) pleats visible (Figs 52c, 55c, 57c, 58c, 59c, 64c)	6
2(1)	Meso and metafemur (completely), and metatibia (at least partially) dark brown to black (Fig. 51a); fore wing with pterostigma mostly brown (Fig. 51b); ovipositor sheaths at least 0.8 × as long as metatibia length (Figs 51a, c); T2 width at posterior margin 3.1 × its length [Hosts: Hesperiidae, *Achlyodes* spp.; hosts feeding on Rutaceae]	*Apanteles anabellecordobae* Fernández-Triana, sp. n.
–	All femora and tibiae yellow (at most with some infuscation on posterior 0.2 × or less of metafemur and metatibia) (Figs 54a, 56a, 60a, 63a); fore wing pterostigma either mostly pale or transparent with thin brown borders or brown with pale area centrally (Figs 54b, 56b, 60b, 63b); ovipositor sheaths at most 0.7 × as long as metatibia length (usually smaller) (Figs 54a, c, 56a, 63a, c); T2 width at posterior margin at least 3.3 × its length [Hosts: Hesperiidae, *Astraptes* spp., *Gorgythion begga pyralina* and *Sostrata bifasciata nordica*; hosts feeding on Fabaceae, Malpighiaceae, Malvaceae, and Sapindaceae]	3
3(2)	Metafemur and metatibia yellow to light brown, with posterior 0.2 × dark brown; tegula pale, humeral complex half pale, half dark; pterostigma brown, with small pale area centrally (Figs 54b, 63b) [Hosts: Hesperiidae, Eudaminae; hosts feeding on Fabaceae, Malvaceae, and Sapindaceae]	4
–	Metafemur, metatibia, tegula and humeral complex yellow; pterostigma mostly pale or transparent with thin brown borders (Figs 56b, 60b) [Hosts: Hesperiidae, Pyrginae; hosts feeding on Malpighiaceae]	5
4(3)	Flagellomerus 2 2.6 × as long as wide; flagellomerus 14 1.9 × as long as wide; mesoscutellar disc 1.5 × as long as wide; T1 3.4 × as long as wide at posterior margin [Hosts: Hesperiidae, *Astraptes* spp.; hosts feeding on Fabaceae, Malvaceae, and Sapindaceae]	*Apanteles osvaldoespinozai* Fernández-Triana, sp. n.
–	Flagellomerus 2 2.9 × as long as wide; flagellomerus 14 1.6 × as long as wide; mesoscutellar disc 1.2 × as long as wide; T1 2.7 × as long as wide at posterior margin [Hosts: Hesperiidae, *Astraptes* spp.; hosts feeding on Fabaceae]	*Apanteles edwinapui* Fernández-Triana, sp. n.
5(3)	Pro- and mesocoxae dark brown, metacoxa black; flagellomerus 2 2.2 × as long as wide; T2 width at posterior margin 3.6 × its length [Host: Hesperiidae, *Gorgythion begga pyralina* feeding on Malpighiaceae deep into rainforests]	*Apanteles luciariosae* Fernández-Triana, sp. n.
–	Pro- and mesocoxae yellow-brown, metacoxa dark brown; flagellomerus 2 3.0 × as long as wide; T2 width at posterior margin 4.7 × its length [Host: Hesperiidae, *Gorgythion begga pyralina* and *Sostrata bifasciata nordica*, feeding on Malpighiaceae in dry and rainforests]	*Apanteles freddyquesadai* Fernández-Triana, sp. n.
6(1)	T1 almost completely smooth and polished, at most with few punctures near posterior margin (Fig. 62g); propodeal areola with longitudinal carinae strongly converging posteriorly, running closely parallel (almost fused) for the posterior third of propodeum length until reaching nucha (Fig. 62g) [Hosts: Hesperiidae, *Polythrix kanshul*]	*Apanteles marianopereirai* Fernández-Triana, sp. n.
–	T1 with at least some sculpture in posterior 0.3-0.5 (Figs 52e, 53f, 57f, 58f, 59f, 61f, 64h); propodeal carina with longitudinal carinae converging right before reaching nucha, not running closely parallel (Figs 52e, 53f, 57f, 58f, 59f, 61f, 64h)	7
7(6)	Meso- and metafemora entirely or mostly dark brown to black (Figs 59a, c) [Host: Hesperiidae, *Noctuana lactifera*]	*Apanteles joseperezi* Fernández-Triana, sp. n.
–	All femora mostly yellow (sometimes a small dark spot present on posterior end of metafemur), or mesofemur yellow and metafemur brown dorsally and yellow ventrally (Figs 52a, 53a, c, 55a, c, 57a, 58a, 61a, 64a)	8
8(7)	Metasoma almost completely yellow (Figs 61a, c, f), except for T1 and T2 (males may have metasoma brown, if so then T3+ paler than T1-T2) [Hosts: Hesperiidae, Eudaminae, *Telemiades antiope*]	*Apanteles manuelpereirai* Fernández-Triana, sp. n.
–	Metasoma mostly dark brown to black, the yellow parts, if any, limited to some sternites and/or laterotergites [Hosts: Hesperiidae, Pyrginae]	9
9(8)	Pterostigma brown with at most a small pale spot at base, most veins brown (Figs 53b, 57b, 64b)	10
–	Pterostigma transparent or whitish with only thin brown borders, most veins transparent (Figs 52b, 55b, 58b)	12
10(9)	T1 3.0 × as long as wide at posterior margin (Fig. 57f); antenna about same length than body; flagellomerus 14 1.4 × as long as wide; metatibial inner spur 1.5 × as long as metatibial outer spur; fore wing with vein r 2.0 × as long as vein 2RS [Host: Hesperiidae, *Nisoniades godma*]	*Apanteles guillermopereirai* Fernández-Triana, sp. n.
–	T1 at least 3.6 × as long as wide at posterior margin (Fig. 64h); antenna clearly shorter than body; flagellomerus 14 at most 1.2 × as long as wide; metatibial inner spur at least 1.8 × as long as metatibial outer spur; fore wing with vein r 1.6 × as long as vein 2RS [Hosts: Hesperiidae, *Staphylus* spp.]	11
11(10)	Metafemur, metatibia and metatarsus yellow, at most with small dark spots in apex of metafemur and metatibia (Fig. 64a) [Hosts: Hesperiidae, *Staphylus vulgata*]	*Apanteles ruthfrancoae* Fernández-Triana, sp. n.
–	Metafemur brown dorsally and yellow ventrally, metatibia with a darker area on apical 0.2–0.3 ×, metatarsus dark (Figs 53a, c) [Hosts: Hesperiidae, *Staphylus evemerus*]	*Apanteles duniagarciae* Fernández-Triana, sp. n.
12(9)	T1 at least 4.0 × as long as posterior width (Fig. 55f); flagellomerus 14 2.3 × as long as wide; flagellomerus 2 1.6 × as long as flagellomerus 14; metafemur 3.3 × as long as wide; mesocutum and mesoscutellar disc mostly heavily and densely punctured; body length 3.3–3.6 mm and fore wing length 3.3–3.6 mm [Hosts: Hesperiidae, *Pyrrhopyge zenodorus*]	*Apanteles eldarayae* Fernández-Triana, sp. n.
–	T1 at most 2.6 × as long as posterior width (Figs 52e, 58f); flagellomerus 14 at most 1.4 × as long as wide; flagellomerus 2 at least 2.0 × as long as flagellomerus 14; metafemur at most 3.0 × as long as wide; mesocutum and mesoscutellar disc mostly smooth or with sparse, shallow punctures; body length 2.4–2.6 mm and fore wing length 2.5–2.7 mm	13
13(12)	T2 width at posterior margin 3.6 × its length; fore wing with vein r 2.4 × as long as vein 2RS, and vein 2RS 0.9 × as long as vein 2M [Hosts: Hesperiidae, *Timochreon satyrus*, *Anisochoria polysticta*]	*Apanteles harryramirezi* Fernández-Triana, sp. n.
–	T2 width at posterior margin 4.3 × its length; fore wing with vein r 1.6 × as long as vein 2RS, and vein 2RS 1.5 × as long as vein 2M [Hosts: Hesperiidae, *Pyrgus* spp., *Heliopetes arsalte*]	*Apanteles carolinacanoae* Fernández-Triana, sp. n.

### *anamarencoae* species-group

This group comprises two species, characterized by pterostigma fully brown; all coxae dark brown to black; tegula, humeral complex, all femora and all tibiae yellow (metafemur with small brown spot on posterior 0.2 × or less); and ovipositor sheaths at least 1.4 × as long as metatibia length. Molecular data does not support this group. Hosts: Tortricidae, Elachistidae, Oecophoridae. All described species are from ACG.

#### Key to species of the *anamarencoae* species-group

**Table:** 

1	Scape anterior 0.6–0.7, entire metatibia and metatarsus yellow (Figs 66a, c, e) [Hosts: Tortricidae]	*Apanteles juanlopezi* Fernández-Triana, sp. n. (N = 2)
–	Scape almost completely dark brown (Fig. 65d); metatibia with small dark spot on posterior 0.1 ×; metatarsus with segment 1 brown to dark brown on posterior 0.5–0.6, remaining segments with some brown marks (Figs 65a, c) [Hosts: Elachistidae, Oecophoridae]	*Apanteles anamarencoae* Fernández-Triana, sp. n. (N = 3)

### *arielopezi* species-group

This group comprises two species, characterized by relatively small body size (body length at most 2.4 mm and fore wing length at most 2.7 mm), mesoscutellar disc smooth, tegula and humeral complex of different color, and brown pterostigma. The group is strongly supported by the Bayesian molecular analysis (PP: 1.0, Fig. 1). Hosts: Tortricidae, Elachistidae. All described species are from ACG.

#### Key to species of the *arielopezi* group

**Table:** 

1	Antenna shorter than body length, extending to half metasoma length; ovipositor sheaths slightly shorter (0.9 ×) than metatibia length (Figs 69a, c)	*Apanteles arielopezi* Fernández-Triana, sp. n.
–	Antenna about same length than body; ovipositor sheaths 1.3 × as long as metatibia length (Figs 70a, c)	*Apanteles mauriciogurdiani* Fernández-Triana, sp. n.

### *ater* species-group

Proposed by Nixon, this is a heterogeneous assemble that contains “many aggregates of species that are not closely related but merge into one another through transitional forms”, and is characterized by having “a well defined areola and costulae in the propodeum, and a vannal lobe that is centrally concave and without setae” (Nixon 1965: 25). Such a general and vague definition created a largely artificial group, including many species worldwide (e.g., Nixon 1965; Mason 1981). Known hosts for the *ater* species-group vary considerably, and the molecular data available for some species (Figs 1, 2) does not support this group either. Future study of the world fauna will likely split the group into smaller, better defined units. For the time being, and just for Mesoamerica, we are keeping here three previously described species (*Apanteles galleriae*, *Apanteles impiger* and *Apanteles leucopus*), as well as six new species that do not fit into any of the other species-groups considered for the region which keeps this as a “garbage can” group. Another six previously described *Apanteles* with Mesoamerican distribution which used to be part of the *ater* group are here removed from that group and transferred as follows: *Apanteles carpatus* to the newly created *carpatus* species-group, *Apanteles leucostigmus* to the newly created *leucostigmus* group, *Apanteles megathymi* to the newly created *megathymi* species-group, *Apanteles paranthrenidis* and *Apanteles thurberiae* to the newly created *paranthrenidis* group, and *Apanteles vulgaris* to the newly created *vulgaris* species-group.

#### Key to species of the *ater* species-group

[The species *Apanteles leucopus* is placed in the *ater* species-group but we could not study any specimens, just photos of the holotype sent from the BMNH (Fig. 78). Unfortunately, the illustrations do not provide all details needed to include the species in any key of this paper]

**Table:** 

1	Pterostigma relatively broad, its length less than 2.5 × its width	*Apanteles galleriae* Wilkinson, 1932
–	Pterostigma relatively narrow, its length more than 3.0 × its width	2
2(1)	Pterostigma entirely brown or brown with pale spot at base (Figs 72b, 73b, 74b, 76b, 77b)	2
–	Pterostigma entirely transparent or mostly transparent with only thin brown borders (as in Fig. 71b)	7
3(2)	Tarsal claws simple	*Apanteles josejaramilloi* Fernández-Triana, sp. n. (N = 1)
–	Tarsal claws with a single basal spine-like seta	4
4(3)	Metacoxa entirely dark brown to black (Fig. 74b); scutoscutellar sulcus thin and with more than 10 close and small impressed pits	*Apanteles franciscopizarroi* Fernández-Triana, sp. n. (N = 1)
–	Metacoxa entirely yellow-white or orange, at most with small brown spot on anterior end (Figs 72a, c, 73a, c, f, 76a); scutoscutellar sulcus relatively wide, with at most 7 widely impressed pits	5
5(4)	Mesoscutellar disc mostly smooth; T2 and T3 yellow-orange (Fig. 76f)	*Apanteles jairomoyai* Fernández-Triana, sp. n. (N = 1)
–	Mesoscutellar disc mostly punctured; T2 and T3 black (Figs 72g, 73f)	6
6(5)	Mesocoxa yellow with anterior 0.3 brown (Fig. 72a); antenna dark brown to black (Figs 72d-f); labrum and tegula dark brown (Figs 72f, g); stigma brown; body length 2.3 mm, and fore wing length 2.6 mm; T1 3.5 × as long as wide; T2 with some sculpture on posterior margin	*Apanteles cristianalemani* Fernández-Triana, sp. n. (N = 1)
–	Mesocoxa entirely yellow (Fig. 73a); antenna with scape and pedicel yellow (Figs 73d, e); labrum yellow (Fig. 73e), tegula yellow-white (Fig. 73f); stigma brown with small pale spot at base; body length 3.7 mm, and fore wing length 3.7 mm; T1 2.4 × as long as wide; T2 smooth	*Apanteles diegoalpizari* Fernández-Triana, sp. n. (N = 4)
7(2)	Pro-, meso-, and part of metacoxa yellow-orange; tegula and humeral complex yellow (Fig. 75g)	*Apanteles impiger* Muesebeck, 1958
–	At least meso- and metacoxae (sometimes also procoxa) dark brown to black (Figs 71a, g); tegula and humeral complex dark brown to black (Fig. 71g)	*Apanteles anariasae* Fernández-Triana, sp. n. (N = 1)

### *bernyapui* species-group

This group comprises four species, characterized by extensive yellow coloration (and usually orange marks on posterior 0.2–0.3 × of anteromesoscutum and upper anterior corner of mesopleura), T1 black (same color of propodeum) and mostly strongly sculptured, with longitudinal striation laterally and a central excavated area with transverse striation. The group is strongly supported by the Bayesian molecular analysis (PP: 1.0, Fig. 1). Hosts: mostly Crambidae, with some records from Elachistidae, Gelechiidae and Noctuidae. All described species are from ACG.

#### Key to species of the *bernyapui* group

**Table:** 

1	Anteromesoscutum and mesopleura completely black (Figs 79a, g)	*Apanteles bernyapui* Fernández-Triana, sp. n.
–	Anteromesoscutum with posterior 0.2–0.3 (especially centrally and along posterior margin) and upper anterior corner of mesopleura orange (Figs 80f, 82g)	2
2(1)	Body length 2.3–2.4 mm; fore wing length 2.5–2.6 mm; ovipositor sheaths 0.6 × as long as metatibia; fore wing with vein r 1.7 × as long as vein 2RS; mesoscutellar disc rather strongly punctured near margins (Fig. 82g)	*Apanteles victorbarrantesi* Fernández-Triana, sp. n. (N = 4)
–	Body length length at least 2.7 mm (usually more); fore wing length at least 2.9 mm (usually more); ovipositor sheaths at least 0.8 × as long as metatibia; fore wing with vein r at most 1.4 × as long as vein 2RS; mesoscutellar disc either smooth, or with shallow punctures (Figs 80f, 81g)	3
3(2)	T1 2.3 × as long as wide at posterior margin; T2 3.9 × as wide as its medial length (Fig. 81g); ovipositor sheaths shorter (0.8 ×) than metatibia; mesoscutellar disc mostly smooth; mesofemur mostly light yellow, with posterior 0.1 light orange; metatibia with anterior 0.6 light yellow, posterior 0.4 orange; ocular-ocellar line 2.0 × as long as posterior ocellus diameter; interocellar distance 1.7 × as long as posterior ocellus diameter; second flagellomerus 2.4 × as long as wide; metafemur 2.9 × as long as wide	*Apanteles raulacevedoi* Fernández-Triana, sp. n.
–	T1 3.3 × as long as wide at posterior margin; T2 3.3 × as wide as its median length (Fig. 80f); ovipositor sheaths same length (1.0 ×) as metatibia; mesoscutellar disc with shallow punctures; mesofemur mostly yellow, with posterior 0.1–0.2 × dark brown; metatibia yellow, with posterior 0.3 dark brown; ocular-ocellar line 2.7 × as long as posterior ocellus diameter; interocellar distance 2.2 × as long as posterior ocellus diameter; second flagellomerus 3.0 × as long as wide; metafemur 3.3 × as long as wide	*Apanteles javiersihezari* Fernández-Triana, sp. n. (N = 3)

### *bienvenidachavarriae* species-group

This group comprises three species, sharing with the *adelinamoralesae* species-group similar morphological and biological (hosts) traits. They differ from the latter group in having meditergite 2 much less transverse, its width at posterior margin usually 2.5 × (at most 2.7 ×) its length – mediotergite 2 usually much more than 2.9 × in the *adelinamoralesae* species-group. The group is strongly supported by the Bayesian molecular analysis (PP: 1.0, Fig. 1); the single exception being *Apanteles marisolarroyoae*, which is included here interimly – its barcode does not cluster with the other two species although it shares with them morphological and host traits. Hosts: Elachistidae. All described species are from ACG.

#### Key to species of the *bienvenidachavarriae* group

**Table:** 

1	Profemur except for at most anterior 0.2, mesofemur in posterior 0.2, and metatibia in anterior 0.7 orange-yellow (Figs 84a, c); antenna as long as body; larger species, body length 3.8–4.0 mm and fore wing length 3.9–4.0 mm [Hosts: Elachistidae, *Anadasmus* spp.]	*Apanteles bienvenidachavarriae* Fernández-Triana, sp. n.
–	Promefur in anterior 0.5, mesofemur entirely, and metatibia in posterior 0.4–0.8 black to dark brown (Figs 85a, e, 86a, c); antenna shorter than body; smaller species, body length 3.0–3.3 mm and fore wing length 3.1–3.3 mm	2
2(1)	Metatibia almost completely black, except for anterior 0.2 or less which is yellow; T1 2.6 × as long as wide at posterior margin [Hosts: Elachistidae, undetermined species]	*Apanteles marisolarroyoae* Fernández-Triana, sp. n.
–	Metatibia at most with black on posterior 0.4–0.5; T1 2.3 × as long as wide at posterior margin [Hosts: Elachistidae, *Antaeotricha zelleri*, *Gonioterma anna*]	*Apanteles josecalvoi* Fernández-Triana, sp. n. (N = 2)

### *calixtomoragai* species-group

This group comprises three species with pectinate tarsal claws, an almost unique feature within the Mesoamerican species of *Apanteles* (the only two other species in the region known to have pectinate tarsal claws, *Apanteles juliodiazi* and *Apanteles waldymedinai*, can be easily separated based on its orange heads). Also, the *calixtomoragai* group contains the largest *Apanteles* in the region (+4.0 mm of body length). The group is strongly supported by the Bayesian molecular analysis (PP: 1.0, Fig. 1). All species are solitary, with the individual coccon (mostly white, but with basal 0.3–0.4 light brown) attached to the leaves where the caterpillar rests when not feeding. Hosts: Hesperiidae. All described species are from ACG, although we have seen undescribed species from other Neotropical areas.

#### Key to species of the *calixtomoragai* group

**Table:** 

1	Sternites and hypopygium dark brown to black (Fig. 89a); all femora dark orange to reddish (Figs 89a, d); fore wing with apical 0.3–0.4 (beyond veins r and 2RS) slightly infumated, clearly darker than rest of wing (Fig. 89b); T1 and T2 with some sculpture near lateral and/or posterior margins (Fig. 89h); fore wing with vein 2RS 1.4 × as long as vein 2M; flagellomerus 14 2.7 × as long as wide (rarely up to 2.8 ×); body length usually over 4.7 mm (range: 4.4–5.2 mm); fore wing length 5.2–5.4 mm; mesoscutellum lunules 0.6–0.7 × as high as maximum height of lateral face of mesoscutellum [Hosts: *Ouleus dilla baru*]	*Apanteles petronariosae* Fernández-Triana, sp. n.
–	Sternites and hypopygium mostly to completely yellow, at most light brown (as in Fig. 88a); pro- and mesofemora yellow, metafemur yellow or orange to reddish; fore wing mostly hyaline (if there is some infumation, it is very slightly and not restricted to wing apex) (Figs 87b, 88b); T1 and T2 mostly smooth (as in Fig. 87e); fore wing with vein 2RS 1.7–1.8 × as long as vein 2M; flagellomerus 14 2.8–3.1 × as long as wide; body length usually less than 4.5 mm (range: 4.0–4.9 mm); forewing length 4.5–5.1 mm; mesoscutellum lunules 0.4–0.5 × as high as maximum height of lateral face of mesoscutellum [Hosts: *Milanion marciana* and *Quadrus cerialis*]	2
2(1)	Mesoscutellum with non-polished area of lateral face with striae interrupted dorsally by a smooth area marking a clear separation from axilla (axilla also with striated sculpture) (Fig. 87e); fore wing length usually 4.8 mm or less (range: 4.5–4.9 mm); body length 4.3 mm (range: 4.0–4.7 mm) [Hosts: *Milanion marciana*. A total of 22 diagnostic characters in the barcoding region: 67 T, 124 T, 133 C, 139 C, 181 T, 194 T, 200 C, 278 C, 298 T, 300 G, 311 A, 319 T, 335 G, 340 C, 346 C, 347 C, 523 T, 595 C, 616 C, 628 T, 634 C, 640 T]	*Apanteles calixtomoragai* Fernández-Triana, sp. n.
–	Mesoscutellum with non-polished area of lateral face with striae that continue towards axilla, with no clear or polished area separating both striated surfaces (Fig. 88g); fore wing length almost always 5.0 mm or more (range: 4.8–5.1 mm); body length 4.5 mm (range: 4.1–4.9 mm) [Hosts: *Quadrus cerialis*. A total of 22 diagnostic characters in the barcoding region: 67 C, 124 C, 133 T, 139 T, 181 A, 194 C, 200 T, 278 T, 298 A, 300 A, 311 G, 319 A, 335 A, 340 T, 346 T, 347 T, 523 C, 595 T, 616 T, 628 A, 634 T, 640 C]	*Apanteles manuelriosi* Fernández-Triana, sp. n.

### *carlosguadamuzi* species-group

This group comprises six species with extensive yellow-orange coloration, smooth mesoscutellar disc, mediotergite 1 weakly sculptured and light coloured with orange-yellow to light brown (males tend to have tergites with darker coloration, compared to females). The group is strongly supported by the Bayesian molecular analysis (PP: 1.0, Fig. 1). Hosts: mostly Crambidae, but some species reared from Choreutidae, Elachistidae, and Gelechiidae. Some species are gregarious and some are solitary parasitoids. All described species are from ACG, although we have seen undescribed species from other Neotropical areas.

#### Key to species of the *carlosguadamuzi* group

**Table:** 

1	T1 light brown, distinctly darker than T2 (Figs 91g, 93f) [Host: *Ategumia lotanalis*]	2
–	T1 entirely orange or orange-yellow, same color as T2 (Figs 90g, 92f, 94f)	3
2(1)	Fore wing with vein r 1.8–2.0 × as long as vein 2RS, and vein 2RS 1.0 × as long as vein 2M	*Apanteles cinthiabarrantesae* Fernández-Triana, sp. n.
–	Fore wing with vein r 1.3 × as long as vein 2RS, and vein 2RS 1.6 × as long as vein 2M	*Apanteles javiercontrerasi* Fernández-Triana, sp. n.
3(1)	T2 width at posterior margin at most 3.1 × its median length (Fig. 94f); ocular-ocellar line at most 1.8 × posterior ocellus diameter	4
–	T2 width at posterior margin at least 3.9 × its median length (Figs 90g, 92f); ocular-ocellar line at least 2.1 × posterior ocellus diameter	5
4(3)	T1 2.5 × as long as wide at posterior margin; T2 width at posterior margin 3.1 × median length; fore wing with vein 2RS 1.6 × as long as vein 2M [Hosts: Gelechiidae]	*Apanteles jesusbrenesi* Fernández-Triana, sp. n. (N = 4)
–	T1 3.1 × as long as wide at posterior margin; T2 width at posterior margin 2.7 × median length; fore wing with vein 2RS 1.9 × as long as vein 2M [Hosts: Elachistidae]	*Apanteles williamcamposi* Fernández-Triana, sp. n. (N = 2)
5(3)	Metatarsus, posterior 0.3 of metatibia, and posterior 0.1 of metafemur brown to black, contrasting with rest of hind leg which is orange-yellow; body length 3.2–3.4 mm; fore wing length 3.4–3.6 mm; fore wing with vein r 2.1 × as long as 2RS; flagellomerus 2 2.6 × as long as wide; metafemur 3.2 × as long as wide [Hosts: Choreutidae, Crambidae]	*Apanteles carlosguadamuzi* Fernández-Triana, sp. n. (N = 5)
–	Metatarsus yellow or orange-yellow, same color as rest of hind leg, except for 0.2 or less of metatibia which is brown; body length usually 2.5–2.7 mm (rarely up to 3.0 mm); fore wing length 2.7–2.9 mm (rarely up to 3.2 mm); fore wing with vein r 1.3 × as long as 2RS; flagellomerus 2 3.2 × as long as wide; metafemur 2.9 × as long as wide [Hosts: Crambidae]	*Apanteles edithlopezae* Fernández-Triana, sp. n.

### *carlosrodriguezi* species-group

This group comprises three species, characterized by hypopygium with relatively short fold where no pleats (or at most one weak pleat) are visible, ovipositor sheaths very short (0.4–0.5 × as long as metatibia), and relatively small size (body length and fore wing length not surpassing 2.5 mm). Another Mesoamerican species, *Apanteles aidalopezae* shares that combination of characters, but can be separate from the *carlosrodriguezi* species-group because of its white pterostigma, transparent or white fore wing veins, and rather elongate glossa. The group is strongly supported by the Bayesian molecular analysis for two of its three component species (PP: 0.99, Fig. 1), however, *Apanteles carlosrodriguezi* clusters apart and future studies may find it is better to split it. Morphological data (especially shape of hypopygium and ovipositor sheaths length) suggest that the species might be placed on a new genus on their own when the phylogeny of Microgastrinae is better resolved. Because that is beyond the scope of this paper, we describe the species under *Apanteles* – the best arrangement at the moment. Hosts: Mostly gregarious on Crambidae; but *Apanteles carlosrodriguezi* is a solitary parasitoid on Elachistidae and possible Choreutidae. All described species are from ACG.

#### Key to species of the *carlosrodriguezi* group

**Table:** 

1	All coxae, most of metatibia, meso- and metafemora dark brown to black (Figs 96a, c, g); body length and fore wing length 1.9–2.0 mm [Solitary parasitoid]	*Apanteles carlosrodriguezi* Fernández-Triana, sp. n. (N = 3)
–	All coxae except for posterior 0.5 of metacoxa, at least anterior 0.3 × of metatibia, most of meso- and metafemora, yellow or white-yellow (Figs 97a, c, 98a, c); body length and fore wing length at least 2.2 mm [Gregarious parasitoids]	2
2(1)	Face reddish-brown, clearly different in color from rest of head, which is dark brown to black (Fig. 98d); metafemur entirely yellow or at most with brown spot dorsally on posterior 0.2–0.3 (Fig. 98c); metatibia brown on posterior 0.6–0.7 (Fig. 98a) [A total of 32 diagnostic characters in the barcoding region: 23 T, 37 G, 68 T, 74 C, 88 A, 181 T, 203 T, 247 C, 259 C, 271 T, 278 T, 295 C, 311 T, 328 A, 346 A, 359 C, 364 T, 385 T, 428 C, 445 C, 448 C, 451 T, 467 C, 490 C, 500 C, 531 C, 544 T, 547 T, 574 C, 577 T, 601 T, 628 A]	*Apanteles robertoespinozai* Fernández-Triana, sp. n.
–	Face almost always dark brown to black, same color as rest of head (Fig. 97e); metafemur brown dorsally on posterior 0.5–0.8 (Fig. 97c); metatibia brown on posterior 0.4–0.5 (Fig. 97a, c) [A total of 32 diagnostic characters in the barcoding region: 23 C, 37 A, 68 C, 74 T, 88 G, 181 A, 203 C, 247 T, 259 T, 271 C, 278 C, 295 T, 311 G, 328 T, 346 T, 359 T, 364 A, 385 C, 428 T, 445 T, 448 T, 451 C, 467 T, 490 T, 500 T, 531 T, 544 A, 547 A, 574 T, 577 C, 601 C, 628 T]	*Apanteles gloriasihezarae* Fernández-Triana, sp. n.

### *carloszunigai* species-group

This group comprises two species, characterized by the combination of folded hypopygium with very few (usually 1-3) pleats occupying just outermost area of fold, small size (fore wing less than 2.8 mm), and all coxae completely yellow. The group is supported by the Bayesian molecular analysis (PP: 0.66, Fig. 1). No host is known for this species-group. All the described species are from ACG; we have seen another species from ACG which cannot be described here because of poor condition of its known specimen.

#### Key to species of the *carloszunigai* group

**Table:** 

1	Metafemur and metatibia almost entirely orange, with light brown spot on posterior 0.1 × (Figs 100a, c); T1 length 3.7 × its width at posterior margin; T2 width at posterior margin 3.5 × its length (Fig. 100g); flagellomerus 2 2.6 × as long as wide	*Apanteles yeissonchavesi* Fernández-Triana, sp. n. (N = 1)
–	Metafemur and metatibia with posterior 0.2–0.3 × brown (Figs 99a, c); T1 length 3.2 × its width at posterior margin; T2 width at posterior margin 4.0 × its length (Fig. 99g); flagellomerus 2 3.0 × as long as wide	*Apanteles carloszunigai* Fernández-Triana, sp. n. (N = 1)

### *carpatus* species-group

Until now, *Apanteles carpatus* had been placed within the *ater* species group. However, we found that the combination of a relatively broad pterostigma (its length less than 3.0 × its width) and mediotergite 2 mostly sculptured with strong longitudinal striation, seems to be characteristic of several Mesoamerican species; which are also strongly supported as a group by the Bayesian molecular analysis (PP: 0.99, Fig. 1). Thus, we here consider them as a distinct group, which so far comprises five species but it is likely to include more when other Neotropical areas are studied. The only hosts known are for *Apanteles carpatus*, a cosmopolitan species with nine different families of host recorded, many of them dubious. More study will be required before accurate host families associated with this species-group can be established.

#### Key to species of the *carpatus* group

**Table:** 

1	T2 length at least 2.5 × its width at posterior margin (Figs 101f, 102g)	2
–	T2 length at most 1.6 × its width at posterior margin (Figs 103g, 104g)	3
2(1)	Metacoxa with posterior 0.3 yellow (Fig. 102a); body length and fore wing length at most 2.2 mm; mesoscutellar disc mostly smooth (Fig. 102f); scutoscutellar sulcus with 11–12 impressions (Fig. 102f); ocular-ocellar line at most 1.8 × posterior ocellus diameter	*Apanteles rhomboidalis* (Ashmead, 1900)
–	Metacoxa brown; body length and fore wing length at least 2.8 mm; mesoscutellar disc mostly sculptured or sculptured near margins (Fig. 101f); scutoscutellar sulcus with 5–6 impressions (Fig. 101f); ocular-ocellar line 2.4 × posterior ocellus diameter	*Apanteles albanjimenezi* Fernández-Triana, sp. n. (N = 1)
3(2)	Fore wing with vein 2RS 1.4 × vein 2M, and vein 2M 0.7 × vein (RS+M)b; body length usually 2.5–2.6 mm (rarely up to 2.8 mm) and fore wing length 2.6–2.7 mm (rarely up to 2.9 mm); anteromesoscutum with extensive orange coloration (Fig. 104g); metatibia inner spur 0.5 × metabasitarsus length	*Apanteles rolandoramosi* Fernández-Triana, sp. n. (N = 4)
–	Fore wing with vein 2RS at most 1.1 × vein 2M, and vein 2M at least 0.9 × vein (RS+M)b; body length and fore wing length usually 3.0 mm or more (rarely less); anteromesoscutum black (Fig. 103g); metatibia inner spur at least 0.6 × metabasitarsus length	4
4(3)	Flagellomerus 2 2.6 × as long as wide; flagellomerus 2 length 2.6 × flagellomerus 14 length; tarsal claws simple; T1 parallel-sided; metacoxa partially yellow (Fig. 103a); ocular-ocellar line 1.8 × posterior ocellus diameter	*Apanteles robertovargasi* Fernández-Triana, sp. n. (N = 1)
–	Flagellomerus 2 at most 2.2 × as long as wide; flagellomerus 2 length at most 2.2 × flagellomerus 14 length; tarsal claws with single basal spine-like seta; T1 clearly widening towards posterior margin; metacoxa entirely brown; ocular-ocellar line at least 2.0 × posterior ocellus diameter	*Apanteles carpatus* (Say, 1836)

### *coffeellae* species-group

This is an artificial group, neither supported by molecular nor host data, but only for some morphological resemblance of the species. It comprises *Apanteles coffeellae* (the only described species of *Apanteles* in Mesoamerica known to parasitize leaf-mining Lepidoptera), as well as three new species from ACG described below. It is characterized by its small size (body length 1.6–2.2 mm, fore wing length 2.0–2.2 mm), and mediotergite 1 strongly narrowing posteriorly. The known hosts (only for *Apanteles coffeellae*) include members of the Lepidoptera families Gracillariidae and Lyonetiidae, but no hosts are known for the other species. The described species are from the Caribbean and ACG, although it is likely that there are more undescribed species from other Neotropical areas. Future study might find this group to contain species of *Apanteles* parasitoids of leaf-mining Lepidoptera.

#### Key to species of the *coffeellae* group

**Table:** 

1	T1 smooth and more than 4.0 × as long as its posterior width (Fig. 106g); fore wing length at most 1.8 mm	*Apanteles coffeellae* Muesebeck, 1958
–	T1 mostly sculptured and less than 4.0 × as long as its posterior width (Fig. 107f, 108f, 109f); fore wing length at least 2.0 mm	2
2(1)	Ovipositor sheaths 1.2 × as long as metatibia (Fig. 108a, c); propodeal areola without transverse carinae extending to spiracle	*Apanteles lisabearssae* Fernández-Triana, sp. n.
–	Ovipositor sheaths at most 0.6 × as long as metatibia (Figs 107a, c, 109a, c); propodeal areola with transverse carinae extending to spiracle (as in Fig. 107f)	3
3(2)	Mesoscutellar disc mostly punctured (Fig. 107f); mesofemur yellow (Fig. 107c); metatibia mostly dark brown, except for anterior 0.3, which is yellow; ovipositor sheaths 0.6 × as long as metatibia	*Apanteles laurahuberae* Fernández-Triana, sp. n.
–	Mesoscutellar disc smooth (Fig. 109f); mesofemur dark brown on anterior 0.5 × (Fig. 109c); metatibia mostly yellow, except for posterior 0.3, which is dark brown; ovipositor sheaths 0.4 × as long as metatibia	*Apanteles mariaguevarae* Fernández-Triana, sp. n. (N = 2)

### *diatraeae* species-group

This group was proposed by Austin and Dangerfield (1989). Those authors considered it a monophyletic group, with striking body modifications associated with specialized parasitism of stem-borers in confined places. They included ten species in the group (seven from the New World, two from Africa and one from the Oriental region). It differs from the morphologically similar *guadaluperodriguezae* group in the propodeum areola shape and the parasitization of stem-borer Crambidae. In Mesoamerica three species are included: *Apanteles deplanatus*, *Apanteles diatraeae*, and *Apanteles fredi*. They are characterized by small size (1.8–2.2 mm in length), body distinctly flattened dorsoventrally, propodeal areola elongate and parallel-sided, and very short antenna (length not surpassing posterior margin of tergite 1). They are all greagarious. Hosts: Crambidae. Distribution: pantropical (Austin and Dangerfield 1989).

#### Key to species of the *diatraeae* group

**Table:** 

1	T1 less than 2.0 × as long as width at its posterior margin and sculptured on its anterior half; T2 width at posterior margin 3.0 × its length; fore wing with vein r 1.0 × as long as vein 2RS; metatibia with basal 0.3 yellow, rest brown; mesoscutellum lunules at least 0.8 × as high as maximum height of lateral face of scutellum [Host: Crambidae (*Diatraea* sp.). Distribution: Guatemala]	*Apanteles fredi* Austin & Dangerfield, 1989
–	T1 more than 2 × as long as width at its posterior margin and either mostly sculptured or with some sculpture near the lateral borders and/or the apical 0.3–0.5 (Figs 203g, 204g); T2 width at posterior margin less than 2.0 × its length; fore wing with vein r 1.4 × as long as vein 2RS; metatibia dark brown; mesoscutellum lunules 0.6–0.7 × as high as maximum height of lateral face of scutellum (Figs 203g, 204g)	2
2(1)	T1 usually less than 3 × as long as width at its posterior margin and heavily sculptured medially, with two strong, median longitudinal carinae on apical half (Fig. 204g); anteromesoscutum mostly smooth or with shallow sparse punctures, except for anterior 0.3 where punctures are deeper and/or denser (Fig. 204g); fore wing with vein 2RS 1.3 × as long as vein 2M; ovipositor sheaths 0.8 × metatibia length (Fig. 204a, c) [Hosts: Crambidae (*Diatraea* spp., *Galleria mellonella*). Distribution: several Caribbean islands, Central and South America, introduced into the US, France and India]	*Apanteles diatraeae* Muesebeck, 1921
–	T1 usually more than 3 × as long as width at its posterior margin and mostly smooth (Fig. 203g); anteromesoscutum mostly smooth (Fig. 203g); fore wing with vein 2RS 0.8 × as long as vein 2M; ovipositor sheaths 0.6 × metatibia length (Fig. 203a, c) [Host: Crambidae (*Diatraea* spp.). Distribution: Mexico]	*Apanteles deplanatus* Muesebeck, 1957

### *dickyui* species-group

This group comprises two species, characterized by pterostigma mostly transparent with only thin brown borders, tegula and humeral complex yellow, all coxae dark brown to black, mediotergite 1 at least 2.4 × as long as wide at posterior margin, and mediotergite 2 mostly scultured. The group is strongly supported by the Bayesian molecular analysis (PP: 1.0, Fig. 1). Hosts: Unknown. The described species are from ACG.

#### Key to species of the *dickyui* group

**Table:** 

1	Interocellar distance 2.1 × as long as ocellus diameter; ocular-ocellar line 2.4 × as long as posterior ocellus diameter; flagellomerus 2 2.8 as long as wide; fore wing with vein R1 4.0 × as long as distance between ends of veins R1 and 3RS	*Apanteles eduardoramirezi* Fernández-Triana, sp. n.
–	Interocellar distance 1.3 × as long as ocellus diameter; ocular-ocellar line 2.0 × as long as posterior ocellus diameter; flagellomerus 2 3.3 as long as wide; fore wing with vein R1 6.0 × as long as distance between ends of veins R1 and 3RS	*Apanteles dickyui* Fernández-Triana, sp. n. (N = 1)

### *erickduartei* species-group

This group comprises five species, characterized by extensive extensive yellow-orange coloration (including tegula and humeral complex, parts of the axillar complex, most of laterotergites 1–4, all sternites, and hypopygium), mesoscutellar disc mostly punctured, and mediotergite 1 more than 2.3 × as long as wide. The group is strongly supported by the Bayesian molecular analysis (PP: 1.0, Fig. 1). The species are solitary parasitoids. Hosts: Crambidae. All the described species are from ACG.

#### Key to species of the *erickduartei* group

**Table:** 

1	Ovipositor sheaths 0.5 × metatibia length (Figs 115a, c); fore wing with vein r 2.4 × vein 2RS; T1 length 2.3 × its width at posterior margin	*Apanteles luishernandezi* Fernández-Triana, sp. n. (N = 4)
–	Ovipositor sheaths at least 0.8 × metatibia length (usually more) (Figs 113a, c, 114a, c, 116a, c, 117a, c); fore wing with vein r at most 1.7 × vein 2RS; T1 length at least 2.5 × its width at posterior margin (usually more)	2
2(1)	T3 mostly yellow (except for thin brown border on anterior margin) (Fig. 117g); metafemur with anterior 0.3–0.4 yellow, rest brown (Figs 117a, c); flagellomerus 2 2.2 × as long as wide	*Apanteles ronaldcastroi* Fernández-Triana, sp. n. (N = 2)
–	T3 either entirely dark brown or with extensive, dark brown, central band, covering 0.4–0.5 of tergite and running from anterior to posterior margins (Figs 113g, 114f, 116f); metafemur either almost entirely dark brown, at most with small yellow spot on anterior 0.1 (usually), or entirely yellow (rarely) (Figs 113a, c, 114a, c, 115a); flagellomerus 2 at least 2.5 × as long as wide	3
3(2)	Ovipositor sheaths 0.8 × metatibia length (rarely up to 0.9 ×) (Fig. 116a, c); T1 strongly narrowing towards posterior margin (maximum width of tergite 1.7 × width at posterior margin) (Fig. 116f); T3 entirely dark brown (Fig. 116f); flagellomerus 2 2.5 × as long as wide; flagellomerus 2 length 2.2 × flagellomerus 14 length; ocular-ocellar line 2.3 × posterior ocellus diameter; interocellar distance 2.2 × posterior ocellus diameter	*Apanteles milenagutierrezae* Fernández-Triana, sp. n.
–	Ovipositor sheaths 1.0-1.2 × metatibia length (as in Figs 114a, c); T1 not so strongly narrowing towards posterior margin (maximum width of tergite 1.2–1.5 × width at posterior margin) (Figs 113g, 114f); T3 partially yellow (Figs 113g, 114f); flagellomerus 2 at least 2.7 × as long as wide; flagellomerus 2 length at least 2.5 × flagellomerus 14 length; ocular-ocellar line at most 2.1 × posterior ocellus diameter; interocellar distance 1.9 × posterior ocellus diameter	4
4(3)	T1 lenght 3.2 × its width at posterior margin; ocular-ocellar line 2.1 × posterior ocellus diameter; flagellomerus 2 2.9 × as long as wide	*Apanteles felixcarmonai* Fernández-Triana, sp. n. (N = 3)
–	T1 length 2.5–2.8 × its width at posterior margin; ocular-ocellar line 1.8 × posterior ocellus diameter; flagellomerus 2 2.7 × as long as wide	*Apanteles erickduartei* Fernández-Triana, sp. n.

### *glenriverai* species-group

This group contains two species characterized by its pleated hypopygium, thick and strong ovipositor (with basal width 3–5× its apical width posterior to constriction), antenna shorter than body, and maximum height of mesoscutellum lunules 0.4 × maximum height of lateral face of mesoscutellum. The group is strongly supported by the Bayesian molecular analysis (PP: 1.0, Fig. 1). Hosts: Pyralidae. The described species are from ACG.

#### Key to species of the *glenriverai* group

**Table:** 

1	Metatibia almost entirely dark brown, with at most anterior 0.2 yellow; body length at most 2.3 mm and fore wing length at most 2.5 mm; T1 mostly smooth, with some sculpture near postero-lateral margins (Fig. 121h); T2 mostly smooth (Fig. 121h); fore wing with vein r 2.3 × vein 2RS; ocular-ocellar line 2.6 × posterior ocellus diameter; interocellar distance 2.1 × posterior ocellus diameter; flagellomerus 2 2.7 × as long as wide	*Apanteles pablovasquezi* Fernández-Triana, sp. n.
–	Metatibia with anterior 0.5–0.6 yellow; body length at least 2.7 mm and fore wing length at least 2.8 mm; T1 with strong longitudinally striate sculpture at least on posterior 0.5 (Fig. 120f); T2 with some sculpture near posterior margin (Fig. 120f); fore wing with vein r 1.8 × vein 2RS; ocular-ocellar line 2.3 × posterior ocellus diameter; interocellar distance 1.9 × posterior ocellus diameter; flagellomerus 2 2.4 × as long as wide	*Apanteles glenriverai* Fernández-Triana, sp. n.

### *guadaluperodriguezae* species-group

This is a somewhat artificial group, not supported by molecular data, although the two component species share some morphological resemblance as well as similar hosts. It also looks morphologically similar to the *diatraeae* species-group, sharing with the latter a somewhat depressed body (dorso-ventrally), short antenna, and relatively small body size. However, it does not have the elongate and parallel-sided propodeal areola that Austin and Dangerfield (1989) considered as an apomorphic trait defining the *diatraeae* species-group. Additionally, the *guadaluperodriguezae* group, unlike the *diatraeae* group, attacks leaf-rolling Crambidae. The described species are from ACG.

#### Key to species of the *guadaluperodriguezae* group

**Table:** 

1	T1 at least 4.5 × as long as its posterior width (Fig. 122f); fore wing with veins r and 2RS meeting in a smooth angle, vein 3RSa absent (Fig. 122b) [Hosts: *Piletosoma thialis*. A total of 30 diagnostic characters in the barcoding region: 81 C, 86 A, 88 T, 91 G, 133 A, 172 T, 250 C, 274 A, 277 T, 310 C, 313 A, 325 A, 328 T, 359 C, 361 T, 364 A, 367 C, 400 A, 412 T, 418 T, 421 A, 424 C, 472 A, 500 T, 517 A, 529 C, 595 C, 631 T, 646 T, 658 C]	*Apanteles guadaluperodriguezae* Fernández-Triana, sp. n.
–	T1 at most 2.8 × as long as its posterior width (Fig. 123f); fore wing with veins r and 2RS meeting in a strong angle from where a clear vein 3RSa is visible (sometimes as a stub) (Fig. 123b) [Hosts: *Pantographa expansalis*, *Phostria mapetalis*. A total of 30 diagnostic characters in the barcoding region: 67 T, 91 T, 92 C, 136 C, 205 C, 212 C, 214 T, 217 A, 223 A, 235 T, 274 C, 299 G, 304 C, 313 C, 370 T, 379 C, 389 G, 391 T, 400 T, 421 C, 424 T, 433 T, 442 C, 481 C, 484 C, 499 T, 505 C, 542 C, 547 T, 548 C, 550 T, 565 T, 574 A, 604 C, 616 T, 622 A]	*Apanteles marcobustosi* Fernández-Triana, sp. n.

### *humbertolopezi* species-group

This group, comprising two species, should only be considered as interim, based on morphological evidence (strong, longitudinally striate sculpture on mediotergite 1; mediotergite 2 fully sculptured; all coxae black; pterostigma and most of veins on fore wing brown), although it is not supported by molecular data. Hosts: Elachistidae. All described species are from ACG, although we have seen other Neotropical species with similarly strong sculpture on mediotergites 1 and 2.

#### Key to species of the *humbertolopezi* group

**Table:** 

1	Ovipositor sheaths 0.9 × as long as metatibia (Fig. 125a, c); pterostigma brown with pale spot at base (Fig. 125b); body length 2.2 mm; fore wing length 2.3 mm; flagellomerus 2 2.7 as long as wide	*Apanteles humbertolopezi* Fernández-Triana, sp. n. (N = 1)
–	Ovipositor sheaths 1.2 × as long as metatibia (Fig. 126a, c); pterostigma brown (Fig. 126b); body length 2.6 mm; fore wing length 2.6 mm; flagellomerus 2 3.2 as long as wide	*Apanteles pablotranai* Fernández-Triana, sp. n. (N = 1)

### *isidrochaconi* species-group

This group comprises two species, characterized by extensive yellow coloration, smooth mediotergite 2, and ovipositor sheaths 1.4 × as long as metatibia. The long ovipositor differentiates this group from the rest of the Mesoamerican species with extensive yellow coloration (which usually have ovipositor sheaths shorter than metatibia, at most 1.2 × as long in a few cases). Also, the barcode for *isidrochaconi* is relatively unique (there is no molecular data for the other species) and provide additional support to consider this as a group on its own. There are no host records known – both species were collected by Malaise traps. Further study on its biology and/or additional DNA data will help to clarify the limits of this group in the future. The described species are from ACG.

#### Key to species of the *isidrochaconi* species-group

**Table:** 

1	T3, laterotergites 1–3, sternites, and hypopygium mostly yellow (at most light brown near margins of T3 and hypopygium) (Figs 127a, c, f); fore and middle legs, and metacoxa entirely orange-yellow (Figs 127a, e); mesoscutellum with maximum height of lunules 0.5 × maximum height of lateral face of mesoscutellum (Fig. 127f)	*Apanteles isidrochaconi* Fernández-Triana, sp. n. (N = 1)
–	T3 completely, and most of laterotergites 1–3, sternites, and hypopygium dark brown to black (Figs 128a, c, f); fore and middle legs yellow-white, metacoxa yellow-white except for anterior 0.1 which is dark brown (Fig. 128a); mesoscutellum with maximum height of lunules 0.2–0.3 × maximum height of lateral face of mesoscutellum (Fig. 128f)	*Apanteles juanapui* Fernández-Triana, sp. n. (N = 1)

### *javierobandoi* species-group

This comprises two species, characterized by glossa elongate (Figs 130e, 131e), tegula and humeral complex of same color (dark brown), and ovipositor about the same width from base to apex. Although the molecular data does not support the grouping of these species, and host information is only available for one of them, we have decided to consider them as a group because the combination of morphological characters detailed above is unique among Mesoamerican *Apanteles*. However, this group should be considered as preliminary and further study may change its status in the future. Hosts: Choreutidae. All described species are from ACG.

#### Key to species of the *javierobandoi* group

**Table:** 

1	Antenna shorter than body, at most extending to half of metasoma; body length and fore wing length 2.4 mm; T1 length 2.4 × its width at posterior margin; T2 mostly sculptured	*Apanteles juangazoi* Fernández-Triana, sp. n. (N = 1)
–	Antenna about same length or slightly larger than body; body length 2.5–3.0 mm, and fore wing length 2.6–3.0 mm; T1 length at most 2.0 × its width at posterior margin; T2 mostly smooth	*Apanteles javierobandoi* Fernández-Triana, sp. n. (N = 4)

### *joserasi* species-group

This group comprises one described species, although we have seen another undescribed species from the same area (with the interim name *Apanteles* Rodriguez79) which is only known from a male in poor condition and cannot be described in this paper. It is characterized by glossa elongate; ovipositor relatively thick and strong (with basal width more than 3.0 × its apical width posterior to constriction); maximum height of mesoscutellum lunules 0.7 × maximum height of lateral face of mesoscutellum; and propodeum with strong sculpture limited to anterior half, with posterior half mostly smooth and shiny, and with transverse carinae complete and strongly raised. All morphological traits mentioned above are similar to the *leucostigmus* species-group, and it might be that in the future this group is sunk within the much larger and widespread *leucostigmus*. However, molecular data (Fig. 1) as well as biological data (species are solitary and parasitize *Venada* in the *joserasi* group, whereas all known species in the *leucostigmus* group are gregarious and parasitize many genera of Eudaminae but not *Venada*) suggest that *joserasi* is better considered as a disctinct group for the time being. Hosts: Hesperiidae. The described species is from ACG.

### *keineraragoni* species-group

This group includes two species, characterized by ovipositor sheaths half the length of metatibia, relatively short inner metatibial spur (at most 0.4 × as long as first segment of metatarsus), and body extensively dark brown to black (including full meso- and meta- soma, and all coxae). All other known species of Mesoamerican *Apanteles* with relatively short ovipositor sheats (i.e., 0.6 × or shorter than metatibia) have a rather extensive yellow-orange coloration. The molecular data does not support this group (Fig. 1), nor does it biology (one species is solitary on crambids, and the other is gregarious on riodinids), but we have decided to keep it as a single group for now based on the distinctive morphological traits. Hosts: Crambidae, Riodinidae. The described species are from ACG.

#### Key to species of the *keineraragoni* group

**Table:** 

1	Fore wing with vein r 1.4 × as long as vein 2RS, vein 2M 1.5 × as long as vein (RS+M)b; flagellomerus 2 2.7 × as long as wide; interocellar distance 1.3 × as long as posterior ocelli diameter; metatibia dark brown to black on posterior 0.8 (Figs 136a, c) [Hosts: Crambidae]	*Apanteles keineraragoni* Fernández-Triana, sp. n. (N = 3)
–	Fore wing with vein r 1.7 × as long as vein 2RS, vein 2M 0.7 × as long as vein (RS+M)b; flagellomerus 2 3.2 × as long as wide; interocellar distance 1.7 × as long as posterior ocelli diameter; metatibia dark brown to black on posterior 0.4–0.5 (Figs 137a, c) [Hosts: Riodinidae]	*Apanteles ronaldnavarroi* Fernández-Triana, sp. n. (N = 1)

### *leucostigmus* species-group

This group, by far the largest in Mesoamerica, comprises 39 species in this paper. It is defined by a thick ovipositor (as thick or thicker than the width of the median flagellomeres, and with anterior width 3.0–5.0 × its posterior width beyond the constriction), ovipositor sheaths 0.5–1.1 × as long as metatibia, propodeum with strong sculpture limited to anterior half, the posterior half mostly smooth; mesoscutellum with lateral face bearing a polished area 0.7 × or more the height of the face, pterostigma and most of fore wing white or transparent, and mediotergite 1 widening towards posterior 0.7, then narrowing toward posterior margin. The group is supported by the Bayesian molecular analysis (PP: 0.74, Fig. 1). Hosts: Hesperiidae. Widely distributed in the Neotropics; we have seen many more undescribed species in collections.

This is the only group where we extensively used molecular (i.e., barcoding) and biological (i.e., host records) characters in the key. Likewise, the species descriptions were also simplified and only include some morphological traits (plus full details on barcoding and host data). This was mostly due to the paucity of morphological characters that serve to distinguish different species. Relying solely on DNA barcoding and/or host data to describe and key species has been done before in Braconidae (e.g., Butcher et al. 2012).

However, we did some preliminary study of using morphometrics to separate species, and the results (unpublished) suggest that morphometrics may work for many, although not all, of the species in this group. We describe here the species that have been found in ACG for the sake of completing its inventory of *Apanteles*.

#### Key to species of the *leucostigmus* group

The species *Apanteles albinervis*, included in this group because of its morphology, is only known from the male holotype, and our key is only to females. There are no hosts or molecular data available for the holotype, collected in “Mexico” in 1904. It is therefore impossible to key this species by any of the character systems used here.

**Table:** 

1	Metatibia entirely or mostly (>0.7) dark brown to black, with yellow to white usually restricted to anterior 0.2 at most (rarely with pale area extending up to anterior 0.3 of metatibia) (as in Figs 166a, d)	2
–	Metatibia light yellow to orange-yellow from 0.4 to almost entire metatibia (as in Figs 197c, 200c)	26
2(1)	Ovipositor sheaths at least 1.0 × as long as metatibia and 1.3 × as long as metafemur	3
–	Ovipositor sheaths at most 0.9 × as long as metatibia and 1.1 × as long as metafemur	4
3(2)	T1 length 2.7–2.8 × its width at posterior margin; T1 maximum width 1.6–1.7 × its width at posterior margin; metafemur usually more than 3.0 × as long as wide (rarely 2.8–2.9 ×) [Host species *Codatractus imalena*]	*Apanteles luzmariaromeroae* Fernández-Triana, sp. n.
–	T1 length 2.5–2.6 × its width at posterior margin; T1 maximum width 1.4–1.5 × its width at posterior margin; metafemur 2.8 × as long as wide [Host species *Astraptus talus*]	*Apanteles marcovenicioi* Fernández-Triana, sp. n. (N = 1)
4(2)	Ovipositor at most 0.7 × as long as metatibia and 0.8 × as long as metafemur	5
–	Ovipositor more than 0.7 × as long as metatibia and usually more than 0.8 × as long as metafemur	6
5(4)	Larger species, body length usually 2.3-2.5 mm (rarely 2.1 mm), and fore wing length usually 2.5–2.6 mm (rarely 2.3–2.4 mm); T1 length 2.7–2.8 × its width at posterior margin [Host species: *Bungalotis erythus*]	*Apanteles ciriloumanai* Fernández-Triana, sp. n.
–	Smaller species, body length at most 2.1 mm, and fore wing length at most 2.3 mm; T1 length 2.5-2.6 × its width at posterior margin [Host species: *Nascus* spp.]	*Apanteles josecortesi* Fernández-Triana, sp. n.
6(4)	Metafemur at most 2.8 × as long as wide (rarely 2.9 × in individual specimens), ***and*** ovipositor sheaths less than 0.9 × as long as metafemur	7
–	Metafemur at least 2.9 × as long as wide ***and/or*** ovipositor sheaths at least 0.9 × as long as metafemur	9
7(6)	Fore wing length 2.5–2.6 mm and body length at least 2.3 mm (usually more) [Host species: *Ocyba calathana*. A total of 18 diagnostic characters in the barcoding region: 38 C, 55 C, 61 C, 154 C, 235 T, 310 C, 316 T, 322 T, 358 C, 397 C, 405 G, 431 C, 457 C, 476 C, 604 T, 610 C, 637 A, 641 C]	*Apanteles cynthiacorderoae* Fernández-Triana, sp. n.
–	Fore wing length at most 2.4 mm (usually less) and body length usually less than 2.3 mm [Host species: *Cephise aelius* or *Phocides* spp. A total of 18 diagnostic characters in the barcoding region: 38 T, 55 T, 61 T, 154 T, 235 C, 310 T, 316 A, 322 A, 358 T, 397 T, 405 A, 431 A, 457 T, 476 A, 604 A, 610 T, 637 T, 641 T]	8
8(7)	T1 length 2.3–2.8 × its width at posterior margin (rarely 2.1–2.2 ×) [Host species: *Cephise aelius*. A total of 39 diagnostic characters in the barcoding region: 19 T, 43 A, 49 C, 98 A, 118 C, 170 A, 181 G, 184 A, 187 T, 212 C, 238 T, 259 C, 263 T, 284 C, 295 A, 298 A, 304 T, 340 C, 364 T, 379 T, 400 C, 421 T, 439 C, 448 T, 458 T, 490 C, 507 T, 508 T, 529 C, 536 T, 562 A, 574 A, 578 T, 589 T, 601 C, 616 T, 629 T, 646 T, 652 C]	*Apanteles hazelcambroneroae* Fernández-Triana, sp. n.
–	T1 length 2.1–2.2 × its width at posterior margin [Host species: *Phocides* spp. A total of 39 diagnostic characters in the barcoding region: 19 C, 43 T, 49 T, 98 G, 118 T, 170 G, 181 A, 184 T, 187 C, 212 T, 238 C, 259 T, 263 C, 284 T, 295 T, 298 G, 304 C, 340 T, 364 A, 379 C, 400 T, 421 C, 439 T, 448 C, 458 C, 490 T, 507 C, 508 C, 529 T, 536 C, 562 T, 574 T, 578 C, 589 C, 601 T, 616 C, 629 C, 646 C, 652 T]	*Apanteles randallgarciai* Fernández-Triana, sp. n.
9(6)	Fore wing with veins C+Sc+R and R1 mostly brown; usually veins r, 2RS, 2M, (RS+M)b, 1CU, 2Cua, and 1m-cu partially brown; interior area of other veins, and at least part of pterostigma, usually light brown or yellowish-white (as in Figs 165b, 172b, 189b)	10
–	Fore wing with veins C+Sc+R and R1 with brown coloration restricted narrowly to borders, interior area of those veins and pterostigma (and sometimes veins r, 2RS and 2M) transparent or white; other veins mostly transparent (as in Figs 173b, 174b, 175b)	19
10(9)	Metafemur 2.7 × as long as wide; ovipositor sheaths 0.9 × as long as metatibia and 1.1 × as long as metafemur	*Apanteles eugeniaphilipsae* Fernández-Triana, sp. n. (N = 2)
–	Metafemur at least 2.8 × as long as wide; ovipositor sheaths at most 0.8 × (rarely 0.9 ×) as long as metatibia and at most 1.0 × as long as metafemur	11
11(10)	Maximum width of T1 (at about 0.7–0.8 × its length) more than 1.7 × its width at posterior margin	*Apanteles rodrigogamezi* Fernández-Triana, sp. n.
–	Maximum width of T1 (at about 0.7–0.8 × its length) less than 1.6 × its width at posterior margin	12
12(11)	Maximum width of T1 (at about 0.7–0.8 × its length) usually at most 1.2 × its width at posterior margin; T1 appearing almost parallel-sided	*Apanteles gerardobandoi* Fernández-Triana, sp. n.
–	Maximum width of T1 at least 1.3 × its width at posterior margin; T1 clearly appearing to widen from base to 0.7–0.8 × its length, then narrowing towards posterior margin of mediotergite	13
13(12)	Ovipositor sheaths about 0.44 mm, metafemur 0.47 mm, metatibia 0.59 mm, and maximum width of T1 0.18 mm, much shorter than below; body length 1.9–2.0 mm and fore wing 2.1–2.2 mm	*Apanteles ricardocaleroi* Fernández-Triana, sp. n.
–	Ovipositor sheaths 0.49–0.59 mm, metafemur 0.54–0.59 mm, metatibia 0.63–0.72 mm and maximum width of T1 0.20–0.25 mm, much longer than above; body length and fore wing usually larger than 2.2 mm, very rarely smaller	14
14(13)	Ovipositor sheaths at most 2.0 × (rarely 2.3 ×) as long as maximum width of T1	*Apanteles diniamartinezae* Fernández-Triana, sp. n.
–	Ovipositor sheaths at least 2.4 × as long as maximum width of T1	15
15(14)	Host species: *Calliades zeutus* or *Urbanus doryssus*	16
–	Hosts species: *Telemiades* spp. (one single rearing record from *Phocides lilea*)	17
16(15)	Body length 1.9–2.0 mm; fore wing 2.1–2.2 mm [Host species: *Calliades zeutus*. A total of 23 diagnostic characters in the barcoding region: 30 C, 66 G, 75 G, 84 T, 138 T, 147 A, 192 T, 219 T, 264 A, 315 A, 352 C, 378 T, 388 A, 397 T, 414 A, 420 C, 528 C, 535 T, 547 T, 561 T, 627 T, 639 C, 645 C]	*Apanteles pabloumanai* Fernández-Triana, sp. n.
–	Body length 2.3 mm or more (rarely 2.1 mm); fore wing at least 2.5 mm [Host species: *Urbanus doryssus*. A total of 23 diagnostic characters in the barcoding region: 30 T, 66 A, 75 A, 84 C, 138 C, 147 G, 192 C, 219 C, 264 G, 315 T, 352 T, 378 C, 388 G, 397 G, 414 G, 420 A, 528 T, 535 C, 547 C, 561 A, 627 A, 639 T, 645 T]	*Apanteles josemonteroi* Fernández-Triana, sp. n.
17(15)	Host species: *Telemiades oiclus*. A total of 10 diagnostic characters in the barcoding region: 57 G, 144 T, 264 G, 273 C, 276 T, 339 C, 381 G, 477 T, 525 C, 645 C	*Apanteles carlosviquezi* Fernández-Triana, sp. n.
–	Hosts species: *Telemiades fides* (one single rearing record from *Phocides lilea*). A total of 10 diagnostic characters in the barcoding region: 57 A, 144 C, 264 A, 273 T, 276 A, 339 T, 381 A, 477 A, 525 T, 645 T	18
18(17)	A total of 18 diagnostic characters in the barcoding region: 73 C, 99 A, 205 C, 265 T, 270 T, 286 C, 315 T, 321 A, 358 T, 462 C, 489 T, 528 T, 535 T, 541 T, 564 T, 567 T, 573 A, 624 A,	*Apanteles inesolisae* Fernández-Triana, sp. n.
–	A total of 18 diagnostic characters in the barcoding region: 73 T, 99 G, 205 T, 265 C, 270 C, 286 T, 315 A, 312 T, 358 C, 462 T, 489 C, 528 C, 535 C, 541 C, 564 A, 567 C, 573 C, 624 T	*Apanteles manuelzumbadoi* Fernández-Triana, sp. n.
19(9)	Ovipositor sheaths 0.6–0.8 × (average 0.7 ×) as long as metatibia and 0.8–0.9 × as long as metafemur	20
–	Ovipositor sheaths 0.8–0.9 × (average at least 0.8 ×) as long as metatibia and at least 1.0 × as long as metafemur	21
20(19)	Antenna same length or longer than body; T1 length usually less than 2.3 × its width at posterior margin; ovipositor sheaths 0.7–0.8 × as long as metatibia and 0.8–1.0 × as long as metafemur	*Apanteles raulsolorsanoi* Fernández-Triana, sp. n.
–	Antenna shorter than body; T1 length 2.5–2.6 × its width at posterior margin; ovipositor sheaths 0.5–0.6 × as long as metatibia and 0.7–0.8 × as long as metafemur	*Apanteles juanmatai* Fernández-Triana, sp. n.
21(19)	Host species: *Aguna* spp	22
–	Host species: either *Bungalotis*, *Chioides*, *Polygonus*, *Telemiades*, or *Urbanus*	23
22(21)	Metatibia almost entirely dark brown to black, with yellow to white coloration restricted to anterior 0.1 at most; T1 length 2.3–2.4 × its width at posterior margin; T1 maximum width 1.2–1.3 × its width at posterior margin	*Apanteles minorcarmonai* Fernández-Triana, sp. n.
–	Metatibia with anterior 0.3 yellow; T1 length 2.9 × or more its width at posterior margin; T1 maximum width 1.8–1.9 × its width at posterior margin	*Apanteles jesusugaldei* Fernández-Triana, sp. n.
23(21)	Antenna clearly shorter than body length, usually 0.8–0.9 × as long as body; metatibia with anterior 0.3 yellow (a few specimens may have metatibia anterior 0.5 yellow, and will not run through here)	24
–	Antenna as long or slightly longer than body length; metatibia almost entirely dark brown to black, with yellow to white coloration restricted to anterior 0.1 at most	25
24(23)	T1 length more than 3.0 × its width at posterior margin; T1 maximum width 1.8–1.9 × its width at posterior margin [Host species: *Urbanus* spp.]	*Apanteles eliethcantillanoae* Fernández-Triana, sp. n.
–	T1 length 2.3–2.4 × its width at posterior margin; T1 maximum width 1.4–1.5 × its width at posterior margin [Hosts species: *Chioides zilpa*, *Polygonus leo*]	*Apanteles federicomatarritai* Fernández-Triana, sp. n.
25(23)	Body length 2.3–2.6 mm (rarely 2.1–2.2 mm); fore wing length at least 2.5 mm; metafemur length 2.7–3.0 × its width [Host species: *Bungalotis quadratum*]	*Apanteles alvarougaldei* Fernández-Triana, sp. n.
–	Body length 2.1–2.2 mm); fore wing length 2.3–2.4 mm; metafemur length 3.2–3.3 × its width [Host species: *Telemiades fides*]	*Apanteles johanvargasi* Fernández-Triana, sp. n. (N =3)
26(2)	Metatibia almost entirely yellow, at most with posterior 0.1 brown or just with slightly darker spot which is almost same color than rest of metatibia	27
–	Metatibia with posterior 0.3–0.4 dark brown, clearly darker than rest of metatibia	31
27(26)	Ovipositor sheaths averaging 0.44 mm (range 0.40–0.46 mm), their length 0.6–0.7 × metatibia length and 0.7–0.8 × metafemur length	*Apanteles mariachavarriae* Fernández-Triana, sp. n.
–	Ovipositor sheaths usually over 0.50 mm (***if*** rarely 0.45 mm in length, ***then*** species average over 0.48 mm), ovipositor sheaths 0.8 × metatibia length (rarely 0.7 ×) and 0.9–1.0 × as long as metafemur	28
28(27)	Antenna shorter than body; T1 length 2.7–2.8 × its width at posterior margin; T1 maximum width 1.6–1.7 × its width at posterior margin	*Apanteles duvalierbricenoi* Fernández-Triana, sp. n.
–	Antenna at least as long as body; T1 length 2.3-2.4 × its width at posterior margin; T1 maximum width 1.4–1.5 × its width at posterior margin	29
29(28)	Host species: *Astraptes anaphus*. A total of 14 diagnostic characters in the barcoding region: 73 T, 145 C, 193 T, 265 A, 293 A, 316 A, 343 G, 359 C, 401 C, 421 T, 476 C, 562 T, 571 C, 628 T	*Apanteles sigifredomarini* Fernández-Triana, sp. n.
–	Host species: *Urbanus* spp. (in two rare cases *Astraptes alardus*, Dan check that for species Rodriguez24). A total of 14 diagnostic characters in the barcoding region: 73 C, 145 T, 193 C, 265 G, 293 T, 316 T, 343 A, 359 T, 401 T, 421 A, 476 A, 562 A, 571 T, 628 A	30
30(29)	Host species: *Urbanus simplicius*. A total of four diagnostic characters in the barcoding region: 166 G, 232 C, 373 T, 379 T	*Apanteles sergioriosi* Fernández-Triana, sp. n.
–	Host species: *Urbanus dorantes* (plus 2 *Astraptes* records). A total of four diagnostic characters in the barcoding region: 166 A, 232 A, 373 A, 379 C	*Apanteles ronaldzunigai* Fernández-Triana, sp. n.
31(26)	Fore wing with veins C+Sc+R and R1 mostly brown; usually veins r, 2RS, 2M, (RS+M)b, 1CU, 2Cua, and 1m-cu partially brown; interior area of other veins, and at least part of pterostigma, usually light brown or yellowish-white (as in Figs 165b, 172b, 189b)	32
–	Fore wing with veins C+Sc+R and R1 with brown coloration restricted narrowly to borders, interior area of those veins and pterostigma (and sometimes veins r, 2RS and 2M) transparent or white; other veins mostly transparent (as in Figs 173b, 174b, 175b)	33
32(31)	Ovipositor sheaths 0.8 × as long as metatibia and 1.0 × as long as metafemur; T1 length 2.7–2.8 × its width at posterior margin [Host species: *Urbanus doryssus*]	*Apanteles lilliammenae* Fernández-Triana, sp. n.
–	Ovipositor sheaths 0.5 × as long as metatibia and 0.6 × as long as metafemur; T1 length 2.3–2.4 × its width at posterior margin [Host species: *Urbanus dorantes*, *Urbanus teleus*]	*Apanteles wadyobandoi* Fernández-Triana, sp. n.
33(31)	Ovipositor sheaths 0.7 × as long as metatibia ***and*** 0.7–0.8 × as long as metafemur; metafemur 3.2 × as long as wide	34
–	Ovipositor sheaths usually 0.8 × as long as metatibia (rarely 0.7 ×) ***and*** 0.9–1.0 × as long as metafemur; metafemur usually less than 3.0 × as long as wide (rarely up to 3.2 ×)	35
34(33)	Body length at most 2.2 mm and fore wing length at most 2.4 mm; metafemur at most 2.9 × as long as wide; T1 length less than 2.0 × its width at posterior margin [Host species: *Urbanus proteus*. Distribution: Caribbean islands (Cuba, Grenada, Puerto Rico, St. Vincent), and southern United States (Florida)]	*Apanteles leucostigmus* (Ashmead, 1900)
–	Body length at least 2.5 mm and fore wing length at least 2.7 mm; metafemur at least 3.2 × as long as wide; T1 length more than 2.6 × its width at posterior margin [Host species: mostly *Astraptes* spp., four known records of *Urbanus* spp. (all different species than *Urbanus proteus*). Distribution: Costa Rica (ACG)]	*Apanteles jorgehernandezi* Fernández-Triana, sp. n.
35(33)	T1 length 1.9–2.0 × its width at posterior margin [Host species: Mostly *Urbanus albimargo* and *Urbanus doryssus* (rarely also *Autochton* sp.). A total of 19 diagnostic characters in the barcoding region: 54 C, 99 A, 177 C, 186 C, 216 T, 237 T, 330 T, 343 A, 388 C, 387 T, 396 A, 423 T, 460 A, 461 T, 528 T, 534 T, 558 A, 580 T, 606 G]	*Apanteles rostermoragai* Fernández-Triana, sp. n.
–	T1 length 2.3–2.6 × its width at posterior margin [Host species: Mostly *Achalarus*, *Astraptus*, *Cogia* and *Thessia*; if from genus *Urbanus*, then almost always from other species than above (*Urbanus belli*, *Urbanus dorantes*, *Urbanus teleus* and *Urbanus viterboana*; very rarely from *Urbanus albimargo*). Barcoding region with different nucleotides at positions mentioned in first half of couplet]	36
36(35)	T1 length 2.5–2.6 × its width at posterior margin; T1 maximum width 1.6–1.7 × its width at posterior margin [Host species: *Urbanus albimargo*, and rarely from *Achalarus toxeus*, *Cogia calchas* and *Thessia jalapus*. A total of 10 diagnostic characters in the barcoding region: 57 C, 93 C, 111 T, 117 G, 150 T, 177 A, 183 T, 309 A, 4444 T, 606 C]	*Apanteles angelsolisi* Fernández-Triana, sp. n.
–	T1 length 2.3–2.4 × its width at posterior margin; T1 maximum width 1.4–1.5 × its width at posterior margin [Host species: *Astraptes* spp., and *Urbanus* spp. but not *Urbanus albimargo*. Barcoding region with different nucleotides at positions mentioned in first half of couplet]	37
37(36)	Metafemur length usually less than 3.0 × its width (range: 2.8–3.1 ×); fore wing length 2.2–2.5 mm [Host species: *Urbanus belli* (with one record of *Urbanus viterboana*). A total of five diagnostic characters in the barcoding region: 192 G, 225 T, 279 C, 615 C, 685 T]	*Apanteles gladysrojasae* Fernández-Triana, sp. n.
–	Metafemur length usually more than 3.0 × its width (range: 3.0–3.4 ×); fore wing length 2.5–2.7 mm [Host species: Mostly species of *Astraptes* (*Astraptes alardus*, *Apanteles apastus*, *Astraptes brevicauda*, *Astraptes talus*, *Astraptes tucuti*), with one record of *Urbanus belli*. Barcoding region with different nucleotides at positions mentioned in first half of couplet]	*Apanteles bernardoespinozai* Fernández-Triana, sp. n.

### *marisolnavarroae* species-group

This group comprises two species, characterized by relatively large body size (body and fore wing length at least 3.3 mm, usually longer), mesoscutellar disc punctured, tegula and humeral complex of different color, and brown pterostigma. The group is strongly supported by the Bayesian molecular analysis (PP: 1.0, Fig. 1). Hosts: Pyralidae. All described species are from ACG.

#### Key to species of the *marisolnavarroae* group

**Table:** 

1	Meso- and metatrochantellus yellow (Fig. 145a); metatibia mostly yellow, with only dark spot on posterior 0.1–0.2	*Apanteles randallmartinezi* Fernández-Triana, sp. n. (N = 2)
–	Meso- and metatrochantellus dark brown to black (Fig. 144a); metatibia with posterior 0.3–0.4 dark brown to black (Fig. 144c)	*Apanteles marisolnavarroae* Fernández-Triana, sp. n. (N = 2)

### *megathymi* species-group

This group comprises two species, characterized by the combination of relatively long ovipositor sheaths, 1.4–1.5 × as long as metatibia; mesoscutellar disc smooth, contrasting with strongly punctured anteromesoscutum; propodeum strongly carinated and sculptured; pterostigma mostly transparent, with thin brown borders; fore wing with shape of junction of veins r and 2RS strongly angulated, and often with a knob; metafemur and metatibia completely or at least partially yellow-orange; and all coxae dark brown to black. We have tentatively considered this as a group based on the morphological similarities; however, there is no molecular data available for those two species, and the host families are different. Future study might find this group to be completely artificial. Hosts: Gelechiidae, Hesperiidae. The two species are widely distributed in the New World, one mostly in the Nearctic, the other in the Neotropics.

#### Key to species of the *megathymi* group

**Table:** 

1	Body length at least 3.5 mm, and fore wing length at least 3.7 mm; T1 length 2.4–2.8 × its posterior width; T2 mostly smooth [Hosts: Hesperiidae. Distribution: Mexico, United States]	*Apanteles megathymi* Riley, 1881
–	Body length at most 3.0 mm, and fore wing length at most 3.2 mm; T1 length 1.3 × its posterior width; T2 entirely sculptured with longitudinal striation (Fig. 146f) [Hosts: Gelechiidae. Distribution: Brazil, Cuba, Grenada, St. Vincent]	*Apanteles balthazari* (Ashmead, 1900)

### *paranthrenidis* species-group

This group comprises four species, characterized by a relatively broad mediotergite 1 (its length at most 1.3 × its width); pterostigma transparent or whitish with only thin brown borders, and most of the fore wing veins transparent; vein 2M at most 0.6 × as long as vein (RS+M)b; and lateral face of scutellum with polished area 0.7–0.8 × maximum face height. Only for one species there are barcodes available, therefore more data will be needed for molecular analysis of this group, which is considered here as just a interim arrangement of species. Hosts: Crambidae, Gelechiidae, Noctuidae, Pyralidae, Sesiidae (some of those records may be questionable, especially those from old references). Most of the available host records are from miners. Distribution: Widely distributed in the New World.

#### Key to species of the *paranthrenidis* group

**Table:** 

1	Femora mostly yellow-orange, at most with small dark spot on posterior 0.1–0.2 of metafemur (Figs 151a, c, 152a, c)	2
–	Mesofemur dark brown to black on at least anterior 0.5, metafemur entirely dark brown to reddish (Figs 150a, c, 153a, c)	3
2(1)	Darker species, with all coxae dark brown to black, metafemur and metatibia with dark spot on posterior 0.1–0.2; flagellomerus 2 3.1 × as long as wide; scutellar suture with up to 13 pits; T2 mostly smooth and width at apex 3.1 × its length (Fig. 151g); fore wing with vein r 1.6 × as long as vein 2RS, and vein 2RS 1.7 × as long as vein 2M [Hosts: Crambidae]	*Apanteles megastidis* Muesebeck, 1958
–	Lighter species, with at least pro- and meso- coxae light brown to yellow, metafemur and metatibia completely yellow to orange; flagellomerus 2 2.2 × as long as wide; scutellar suture with at most 10 pits; T2 with some sculpture near posterior margin and width at apex at least 3.6 × its length (usually more) (Fig. 152f); fore wing with vein r 3.0 × as long as vein 2RS, and vein 2RS 1.1 × as long as vein 2M [Hosts: Noctuidae, Sesiidae]	*Apanteles paranthrenidis* Muesebeck, 1921
3(2)	Glossa weakly elongate (Fig. 150f); tarsal claws with a basal spine-like seta; metatibia with posterior 0.3 dark; metatarsus with segment 1 dark brown to black on posterior 0.8–0.9 (Fig. 150a); interocellar distance 1.6 × as long as posterior ocellus diameter; T2 with at posterior margin 4.0 × its length; metatibial inner spur 1.8 × as long as outer spur [Hosts: Crambidae, Pyralidae]	*Apanteles esthercentenoae* Fernández-Triana, sp. n.
–	Glossa not elongate (Fig. 153e); tarsal claws simple; metatibia with posterior 0.1 dark; metatarsus with segment 1 dark brown on posterior 0.5 (Fig. 153a); interocellar distance 1.9 × as long as posterior ocellus diameter; T2 with at posterior margin 3.4 × its length; metatibial inner spur 1.4 × as long as outer spur [Hosts: Gelechiidae, Noctuidae]	*Apanteles thurberiae* Muesebeck, 1921

### *ronaldgutierrezi* species-group

This group comprises two species, characterized by the combination of pale tegula and humeral complex dark, ovipositor sheaths much shorter (0.6 ×) than metatibia length, and metatrochanter, metatrochantellus, and anterior third of metafemur yellow-white. Molecular data also supports the species as divergent (Fig. 1). We have included here the species *Apanteles insularis* Muesebeck, 1921, based on the examination of few photos from the holotype (see more comments below under that species). However, we cannot be sure of the actual placement of *insularis* until more specimens can be examined, thus its placement here is preliminary and likely to change over time. Hosts: Choreutidae. The described species are from Costa Rica (ACG), Grenada and St. Vincent.

### *samarshalli* species-group

This group comprises two unique species among all described *Apanteles* in Mesoamerica, characterized by having fore wing vein 2M very short and close to vein 2RS (almost obliterating the space of the second submarginal cell), and antenna very short, not surpassing the mesosoma. The group is strongly supported by the Bayesian molecular analysis (PP: 1.0, Fig. 1), although it clusters apart from all other known species of Mesoamerican *Apanteles* – strongly suggesting it might better be placed on a different genus on its own when future studies on Microgastrinae phylogeny are done. No host is known. One of the species is rather widespread (Neartic and Neotropical) whereas the other one is only know from ACG.

#### Key to species of the *samarshalli* group

**Table:** 

1	Scape, pedicel (partially), anterior 0.7 of metatibia and anterior 0.6 of first segment of metatarsus yellow (Figs 205a, c); propodeum with areola weakly defined by central impression and few rugae or minute carinae arising from nucha (Fig. 205d)	*Apanteles samarshalli* Fernández-Triana, 2010
–	Scape and pedicel light brown (Fig. 160f); posterior 0.5 of metatibia and first segment of metatarsus dark brown (Figs 160a, d); propodeum with areola completely defined by carinae (Fig. 160g)	*Apanteles jimmychevezi* Fernández-Triana, sp. n. (N = 1)

## Taxonomic treatment of species (in alphabetical order)

### Apanteles adelinamoralesae

Fernández-Triana sp. n.

http://zoobank.org/FD4E4F59-D578-43EF-B96C-50BC17CB0AB0

http://species-id.net/wiki/Apanteles_adelinamoralesae

Figs 4 , 210

#### Type locality.

COSTA RICA, Alajuela, ACG, Sector Rincon Rain Forest, Estación Llanura, 135m, 10.93332, -85.25331.

#### Holotype.

♀ in CNC. Specimen labels: 1. Voucher: D.H.Janzen & W.Hallwachs, DB: http://janzen.sas.upenn.edu, Area de Conservación Guanacaste, COSTA RICA, 09-SRNP-75013. 2. DHJPAR0039774.

#### Description.

**Female.** Body color: body mostly dark except for some sternites which may be pale. Antenna color: scape, pedicel, and flagellum dark. Coxae color (pro-, meso-, metacoxa): dark, dark, dark. Femora color (pro-, meso-, metafemur): anteriorly dark/posteriorly pale, dark, dark. Tibiae color (pro-, meso-, metatibia): pale, pale, mostly dark but anterior 0.2 or less pale. Tegula and humeral complex color: tegula pale, humeral complex half pale/half dark. Pterostigma color: mostly pale and/or transparent, with thin dark borders. Fore wing veins color: partially pigmented (a few veins may be dark but most are pale). Antenna length/body length: antenna about as long as body (head to apex of metasoma); if slightly shorter, at least extending beyond anterior 0.7 metasoma length. Body in lateral view: not distinctly flattened dorso–ventrally. Body length (head to apex of metasoma): 3.1–3.2 mm. Fore wing length: 3.1–3.2 mm. Ocular–ocellar line/posterior ocellus diameter: 2.3–2.5. Interocellar distance/posterior ocellus diameter: 1.7–1.9. Antennal flagellomerus 2 length/width: 2.9–3.1. Antennal flagellomerus 14 length/width: 1.4–1.6. Length of flagellomerus 2/length of flagellomerus 14: 2.0–2.2. Tarsal claws: simple. Metafemur length/width: 3.2–3.3. Metatibia inner spur length/metabasitarsus length: 0.4–0.5. Anteromesoscutum: mostly with deep, dense punctures (separated by less than 2.0 × its maximum diameter). Mesoscutellar disc: mostly smooth. Number of pits in scutoscutellar sulcus: 9 or 10. Maximum height of mesoscutellum lunules/maximum height of lateral face of mesoscutellum: 0.6–0.7. Propodeum areola: completely defined by carinae, including transverse carina extending to spiracle. Propodeum background sculpture: mostly sculptured. Mediotergite 1 length/width at posterior margin: 2.6–2.8. Mediotergite 1 shape: mostly parallel–sided for 0.5–0.7 of its length, then narrowing posteriorly so mediotergite anterior width >1.1 × posterior width. Mediotergite 1 sculpture: mostly sculptured, excavated area centrally with transverse striation inside and/or a polished knob centrally on posterior margin of mediotergite. Mediotergite 2 width at posterior margin/length: 2.8–3.1. Mediotergite 2 sculpture: mostly smooth. Outer margin of hypopygium: with a wide, medially folded, transparent, semi–desclerotized area; usually with 4 or more pleats. Ovipositor thickness: about same width throughout its length. Ovipositor sheaths length/metatibial length: 1.2–1.3. Length of fore wing veins r/2RS: 1.7–1.9. Length of fore wing veins 2RS/2M: 2.1 or more. Length of fore wing veins 2M/(RS+M)b: 0.5–0.6. Pterostigma length/width: 3.1–3.5. Point of insertion of vein r in pterostigma: clearly beyond half way point length of pterostigma. Angle of vein r with fore wing anterior margin: more or less perpendicular to fore wing margin. Shape of junction of veins r and 2RS in fore wing: distinctly but not strongly angled.

**Male.** Unknown.

#### Molecular data.

Sequences in BOLD: 2, barcode compliant sequences: 2.

#### Biology/ecology.

Solitary (Fig. 210). Hosts: Elachistidae, *Antaeotricha* Janzen86, *Stenoma* Janzen148.

#### Distribution.

Costa Rica, ACG.

#### Etymology.

We dedicate this species to Adelina Morales for her diligent efforts as a parataxonomist in the ACG inventory of its plant viruses and for Estación Biológica Santa Rosa.

### Apanteles adrianachavarriae

Fernández-Triana sp. n.

http://zoobank.org/962A9F19-AF95-49DC-ABE3-B682599C05CC

http://species-id.net/wiki/Apanteles_adrianachavarriae

Figs 23 , 225

#### Type locality.

COSTA RICA, Alajuela, ACG, Sector Rincon Rain Forest, Sendero Tucan, 410m, 10.90424, -85.2712.

#### Holotype.

♀ in CNC. Specimen labels: 1. DHJPAR0039757. 2. COSTA RICA, Alajuela, ACG, Sector Rincon Rain Forest, Sendero Tucan, 10.90424, -85.2712, 410m, 16.vii.2009, DHJPAR0039757. 3. Voucher: D.H.Janzen & W.Hallwachs, DB: http://janzen.sas.upenn.edu, Area de Conservación Guanacaste, COSTA RICA, 09-SRNP-41735.

#### Paratypes.

2 ♀, 3 ♂ (CNC, NMNH). COSTA RICA, ACG database codes: DHJPAR0048170, DHJPAR0039073.

#### Description.

**Female.** Body color: body mostly dark except for some sternites which may be pale. Antenna color: scape, pedicel, and flagellum dark. Coxae color (pro-, meso-, metacoxa): dark, dark, dark. Femora color (pro-, meso-, metafemur): anteriorly dark/posteriorly pale, dark, dark. Tibiae color (pro-, meso-, metatibia): pale, pale, mostly dark but anterior 0.2 or less pale. Tegula and humeral complex color: tegula pale, humeral complex half pale/half dark. Pterostigma color: mostly pale and/or transparent, with thin dark borders. Fore wing veins color: partially pigmented (a few veins may be dark but most are pale). Antenna length/body length: antenna about as long as body (head to apex of metasoma); if slightly shorter, at least extending beyond anterior 0.7 metasoma length. Body in lateral view: not distinctly flattened dorso–ventrally. Body length (head to apex of metasoma): 2.5–2.6 mm. Fore wing length: 2.7–2.8 mm or 2.9–3.0 mm. Ocular–ocellar line/posterior ocellus diameter: 2.3–2.5. Interocellar distance/posterior ocellus diameter: 2.0–2.2. Antennal flagellomerus 2 length/width: 2.6–2.8. Antennal flagellomerus 14 length/width: 1.7–1.9. Length of flagellomerus 2/length of flagellomerus 14: 1.7–1.9. Tarsal claws: with single basal spine–like seta. Metafemur length/width: 3.0–3.1. Metatibia inner spur length/metabasitarsus length: 0.4–0.5. Anteromesoscutum: mostly with shallow, dense punctures (separated by less than 2.0 × its maximum diameter). Mesoscutellar disc: with a few sparse punctures. Number of pits in scutoscutellar sulcus: 9 or 10. Maximum height of mesoscutellum lunules/maximum height of lateral face of mesoscutellum: 0.8 or more. Propodeum areola: completely defined by carinae, including transverse carina extending to spiracle. Propodeum background sculpture: partly sculptured, especially on anterior 0.5. Mediotergite 1 length/width at posterior margin: 1.7–1.9. Mediotergite 1 shape: more or less parallel–sided. Mediotergite 1 sculpture: mostly sculptured, excavated area centrally with transverse striation inside and/or a polished knob centrally on posterior margin of mediotergite. Mediotergite 2 width at posterior margin/length: 3.6–3.9. Mediotergite 2 sculpture: mostly smooth. Outer margin of hypopygium: with a wide, medially folded, transparent, semi–desclerotized area; usually with 4 or more pleats. Ovipositor thickness: about same width throughout its length. Ovipositor sheaths length/metatibial length: 1.4–1.5. Length of fore wing veins r/2RS: 2.3 or more. Length of fore wing veins 2RS/2M: 0.9–1.0. Length of fore wing veins 2M/(RS+M)b: 0.5–0.6. Pterostigma length/width: 3.6 or more. Point of insertion of vein r in pterostigma: about half way point length of pterostigma. Angle of vein r with fore wing anterior margin: clearly outwards, inclined towards fore wing apex. Shape of junction of veins r and 2RS in fore wing: distinctly but not strongly angled.

**Male.** As in female, with slender mediotergite 1.

#### Molecular data.

Sequences in BOLD: 3, barcode compliant sequences: 3.

#### Biology/ecology.

Solitary (Fig. 225). Host: Elachistidae, *Stenoma* Janzen08 feeding on *Clusia* spp.

#### Distribution.

Costa Rica, ACG.

#### Etymology.

We dedicate this species to Adriana Chavarría in recognition of her diligent efforts for the ACG Programa de Ecoturismo.

### Apanteles adrianaguilarae

Fernández-Triana sp. n.

http://zoobank.org/73C1363A-38F8-408F-9F74-5DCBC2D10AC8

http://species-id.net/wiki/Apanteles_adrianaguilarae

Figs 32 , 232

Apanteles Rodriguez15. Smith et al. (2008). Interim name provided by the authors.

#### Type locality.

COSTA RICA, Alajuela, ACG, Sector Rincón Rain Forest, Rio Francia Arriba, 400m, 10.89666, -85.29003.

#### Holotype.

♀ in CNC. Specimen labels: 1. COSTA RICA, Alajuela, ACG, Sector Rincón Rain Forest, Rio Francia Arriba, 27.vii.2001, 400m, 10.89666, -85.29003, DHJPAR0001553.

#### Paratypes.

43 ♀, 14 ♂ (BMNH, CNC, INBIO, INHS, NMNH). COSTA RICA, ACG database codes: DHJPAR0003005, DHJPAR0003027, DHJPAR0034265, DHJPAR0034271, DHJPAR0038956, 01-SRNP-5505, 02-SRNP-1979, 04-SRNP-34656, 04-SRNP-34908, 04-SRNP-55638, 04-SRNP-55691.

#### Description.

**Female.** Body color: head dark, mesosoma dark with parts of axillar complex pale, metasoma with some mediotergites, most laterotergites, sternites, and/or hypopygium pale. Antenna color: scape, pedicel, and flagellum pale. Coxae color (pro-, meso-, metacoxa): pale, pale, pale or pale, pale, partially pale/partially dark. Femora color (pro-, meso-, metafemur): pale, pale, pale. Tibiae color (pro-, meso-, metatibia): pale, pale, mostly pale but with posterior 0.2 or less dark. Tegula and humeral complex color: both pale. Pterostigma color: dark. Fore wing veins color: mostly dark (a few veins may be unpigmented). Antenna length/body length: antenna about as long as body (head to apex of metasoma); if slightly shorter, at least extending beyond anterior 0.7 metasoma length. Body in lateral view: not distinctly flattened dorso–ventrally. Body length (head to apex of metasoma): 2.7–2.8 mm or 2.9–3.0 mm. Fore wing length: 2.7–2.8 mm, 2.9–3.0 mm or 3.1–3.2 mm. Ocular–ocellar line/posterior ocellus diameter: 2.3–2.5. Interocellar distance/posterior ocellus diameter: 2.0–2.2. Antennal flagellomerus 2 length/width: 2.6–2.8. Antennal flagellomerus 14 length/width: 1.7–1.9. Length of flagellomerus 2/length of flagellomerus 14: 2.0–2.2. Tarsal claws: simple or with single basal spine–like seta. Metafemur length/width: 2.8–2.9. Metatibia inner spur length/metabasitarsus length: 0.4–0.5. Anteromesoscutum: mostly with deep, dense punctures (separated by less than 2.0 × its maximum diameter). Mesoscutellar disc: with a few sparse punctures. Number of pits in scutoscutellar sulcus: 7 or 8. Maximum height of mesoscutellum lunules/maximum height of lateral face of mesoscutellum: 0.4–0.5. Propodeum areola: completely defined by carinae, but only partial or absent transverse carina. Propodeum background sculpture: mostly sculptured. Mediotergite 1 length/width at posterior margin: 2.3–2.5. Mediotergite 1 shape: mostly parallel–sided for 0.5–0.7 of its length, then narrowing posteriorly so mediotergite anterior width >1.1 × posterior width. Mediotergite 1 sculpture: with some sculpture near lateral margins and/or posterior 0.2–0.4 of mediotergite. Mediotergite 2 width at posterior margin/length: 4.4–4.7. Mediotergite 2 sculpture: mostly smooth. Outer margin of hypopygium: with a wide, medially folded, transparent, semi–desclerotized area; usually with 4 or more pleats. Ovipositor thickness: about same width throughout its length. Ovipositor sheaths length/metatibial length: 0.6–0.7. Length of fore wing veins r/2RS: 1.4–1.6. Length of fore wing veins 2RS/2M: 1.1–1.3. Length of fore wing veins 2M/(RS+M)b: 0.9–1.0. Pterostigma length/width: 3.1–3.5. Point of insertion of vein r in pterostigma: clearly beyond half way point length of pterostigma. Angle of vein r with fore wing anterior margin: clearly outwards, inclined towards fore wing apex. Shape of junction of veins r and 2RS in fore wing: strongly angulated, sometimes with a knob.

**Male.** Metacoxa tends to have an anterodorsal brown spot, otherwise similar to female.

#### Molecular data.

Sequences in BOLD: 37, barcode compliant sequences: 37.

#### Biology/ecology.

Gregarious (Fig. 232). Host: Tortricidae, *Anacrusis nephrodes*.

#### Distribution.

Costa Rica, ACG.

#### Etymology.

We dedicate this species to Adriana Aguilar in recogition of her diligent efforts for the ACG Programa Forestal.

### Apanteles adrianguadamuzi

Fernández-Triana sp. n.

http://zoobank.org/672C30FF-0A5A-447B-B16C-45A8DC3394CD

http://species-id.net/wiki/Apanteles_adrianguadamuzi

Figs 24 , 226

#### Type locality.

COSTA RICA, Guanacaste, ACG, Potrerillos, Río Azufrado, 95m, 10.81224, -85.54438.

#### Holotype.

♀ in CNC. Specimen labels: 1. DHJPAR0005279. 2. COSTA RICA, Guanacaste, ACG, Potrerillos, Río Azufrado, 23.vii.2000, gusaneros. 3. 00-SRNP-16110, Same as 00-16047, On *Inga vera*.

#### Paratypes.

1 ♂ (CNC). COSTA RICA, ACG database codes: DHJPAR0039780).

#### Description.

**Female.** Body color: body mostly dark except for some sternites which may be pale. Antenna color: scape, pedicel, and flagellum dark. Coxae color (pro-, meso-, metacoxa): dark, dark, dark. Femora color (pro-, meso-, metafemur): anteriorly dark/posteriorly pale, dark, dark. Tibiae color (pro-, meso-, metatibia): pale, anteriorly pale/posteriorly dark, dark. Tegula and humeral complex color: tegula dark, humeral complex half pale/half dark. Pterostigma color: dark with pale spot at base. Fore wing veins color: partially pigmented (a few veins may be dark but most are pale). Antenna length/body length: antenna about as long as body (head to apex of metasoma); if slightly shorter, at least extending beyond anterior 0.7 metasoma length. Body in lateral view: not distinctly flattened dorso–ventrally. Body length (head to apex of metasoma): 2.5–2.6 mm. Fore wing length: 2.5–2.6 mm. Ocular–ocellar line/posterior ocellus diameter: 2.3–2.5. Interocellar distance/posterior ocellus diameter: 1.7–1.9. Antennal flagellomerus 2 length/width: 2.6–2.8. Antennal flagellomerus 14 length/width: 1.4–1.6. Length of flagellomerus 2/length of flagellomerus 14: 2.0–2.2. Tarsal claws: simple. Metafemur length/width: 3.0–3.1. Metatibia inner spur length/metabasitarsus length: 0.4–0.5. Anteromesoscutum: mostly with deep, dense punctures (separated by less than 2.0 × its maximum diameter). Mesoscutellar disc: with punctures near margins, central part mostly smooth. Number of pits in scutoscutellar sulcus: 11 or 12. Maximum height of mesoscutellum lunules/maximum height of lateral face of mesoscutellum: 0.6–0.7. Propodeum areola: completely defined by carinae, including transverse carina extending to spiracle. Propodeum background sculpture: partly sculptured, especially on anterior 0.5. Mediotergite 1 length/width at posterior margin: 1.7–1.9. Mediotergite 1 shape: more or less parallel–sided. Mediotergite 1 sculpture: mostly sculptured, excavated area centrally with transverse striation inside and/or a polished knob centrally on posterior margin of mediotergite. Mediotergite 2 width at posterior margin/length: 4.0–4.3. Mediotergite 2 sculpture: mostly smooth. Outer margin of hypopygium: with a wide, medially folded, transparent, semi–desclerotized area; usually with 4 or more pleats. Ovipositor thickness: about same width throughout its length. Ovipositor sheaths length/metatibial length: 1.0–1.1. Length of fore wing veins r/2RS: 1.7–1.9. Length of fore wing veins 2RS/2M: 1.7–1.8. Length of fore wing veins 2M/(RS+M)b: 0.5–0.6. Pterostigma length/width: 2.6–3.0. Point of insertion of vein r in pterostigma: about half way point length of pterostigma. Angle of vein r with fore wing anterior margin: clearly outwards, inclined towards fore wing apex. Shape of junction of veins r and 2RS in fore wing: strongly angulated, sometimes with a knob.

**Male.** The only available specimen is in poor condition, missing metasoma, some legs and part of antennae.

#### Molecular data.

Sequences in BOLD: 3, barcode compliant sequences: 2.

#### Biology/ecology.

Solitary (Fig. 226). Hosts: Elachistidae, *Antaeotricha* similisEPR01, *Stenoma* Janzen07, *Stenoma* Janzen44; Crambidae, *Omiodes* Janzen03, *Omiodes* Janzen06.

#### Distribution.

Costa Rica, ACG.

#### Etymology.

We dedicate this species to Adrian Guadamuz in recognition of his diligent efforts for the ACG Programa de Parataxónomos and the plant inventory of ACG.

### Apanteles aichagirardae

Fernández-Triana sp. n.

http://zoobank.org/4131A739-EEDF-41A0-8DC4-B52F162FAC83

http://species-id.net/wiki/Apanteles_aichagirardae

Fig. 35

Apanteles Rodriguez150 (Smith et al. 2006). Interim name provided by the authors.

#### Type locality.

COSTA RICA, Guanacaste, ACG, Sector Cacao, Sendero Derrumbe, 1220 meters, 10.92918, -85.46426.

#### Holotype.

♀ in CNC. Specimen labels: 1. COSTA RICA, Guanacaste, ACG, Sector Cacao, Sendero Derrumbe, 1220 meters, 24.iv.2006, 10.92918, -85.46426, 01-SRNP6737. 2. DHJPAR0012466.

#### Paratypes.

1 ♀ (CNC). COSTA RICA: Guanacaste, ACG database code: DHJPAR0012468.

#### Description.

**Female.** Body color: body mostly dark except for some sternites which may be pale. Antenna color: scape, pedicel, and flagellum dark. Coxae color (pro-, meso-, metacoxa): dark, dark, dark. Femora color (pro-, meso-, metafemur): pale, pale, dark. Tibiae color (pro-, meso-, metatibia): pale, pale, anteriorly pale/posteriorly dark. Tegula and humeral complex color: tegula pale, humeral complex half pale/half dark. Pterostigma color: dark. Fore wing veins color: mostly dark (a few veins may be unpigmented). Antenna length/body length: antenna about as long as body (head to apex of metasoma); if slightly shorter, at least extending beyond anterior 0.7 metasoma length. Body in lateral view: not distinctly flattened dorso–ventrally. Body length (head to apex of metasoma): 3.1–3.2 mm or 3.3–3.4 mm. Fore wing length: 3.3–3.4 mm or 3.5–3.6 mm. Ocular–ocellar line/posterior ocellus diameter: 1.7–1.9. Interocellar distance/posterior ocellus diameter: 1.7–1.9. Antennal flagellomerus 2 length/width: 3.2 or more. Antennal flagellomerus 14 length/width: 1.4–1.6. Length of flagellomerus 2/length of flagellomerus 14: 2.3–2.5. Tarsal claws: with single basal spine–like seta. Metafemur length/width: 3.0–3.1. Metatibia inner spur length/metabasitarsus length: 0.6–0.7. Anteromesoscutum: mostly with deep, dense punctures (separated by less than 2.0 × its maximum diameter). Mesoscutellar disc: mostly smooth. Number of pits in scutoscutellar sulcus: 9 or 10. Maximum height of mesoscutellum lunules/maximum height of lateral face of mesoscutellum: 0.4–0.5. Propodeum areola: completely defined by carinae, including transverse carina extending to spiracle. Propodeum background sculpture: mostly sculptured. Mediotergite 1 length/width at posterior margin: 1.1–1.3. Mediotergite 1 shape: clearly widening towards posterior margin. Mediotergite 1 sculpture: mostly sculptured, excavated area centrally with transverse striation inside and/or a polished knob centrally on posterior margin of mediotergite. Mediotergite 2 width at posterior margin/length: 3.2–3.5. Mediotergite 2 sculpture: with some sculpture, mostly near posterior margin. Outer margin of hypopygium: with a wide, medially folded, transparent, semi–desclerotized area; usually with 4 or more pleats. Ovipositor thickness: about same width throughout its length. Ovipositor sheaths length/metatibial length: 1.0–1.1. Length of fore wing veins r/2RS: 1.7–1.9. Length of fore wing veins 2RS/2M: 1.4–1.6. Length of fore wing veins 2M/(RS+M)b: 0.7–0.8. Pterostigma length/width: 2.6–3.0. Point of insertion of vein r in pterostigma: clearly beyond half way point length of pterostigma. Angle of vein r with fore wing anterior margin: more or less perpendicular to fore wing margin. Shape of junction of veins r and 2RS in fore wing: distinctly but not strongly angled.

**Male.** Unknown.

#### Molecular data.

Sequences in BOLD: 2, barcode compliant sequences: 2.

#### Biology/ecology.

Solitary. Host: Elachistidae, specimen with ACG database code: 01-SRNP-6737.

#### Distribution.

Costa Rica, ACG.

#### Comments.

This species is characterized by the combination of tegula different color from humeral complex, pterostigma brown, mediotergite 1 clearly widening towards posterior margin (1.3 × as long as wide at posterior margin), mediotergite 2 with posterior margin sinuate (width at expanded central area 1.7 × as large as width at lateral area), and ovipositor relatively thick (basal width about twice apical width).

#### Etymology.

We dedicate this species to Aicha Girardi, daughter of Caroline Boudreault (CNC, Ottawa) as an appreciation for Caroline’s support, especially photographing many types for this paper.

### Apanteles aidalopezae

Fernández-Triana sp. n.

http://zoobank.org/15026F7A-C5E1-4E0D-8C54-7E06164E4473

http://species-id.net/wiki/Apanteles_aidalopezae

Fig. 36

#### Type locality.

COSTA RICA, Alajuela, ACG, Sector Pitilla, Bullas, 440 meters, 10.98670, -85.38503.

#### Holotype.

♀ in CNC. Specimen labels: 1. DHJPAR0042048. 2. Voucher: D.H.Janzen & W.Hallwachs, DB: http://janzen.sas.upenn.edu, Area de Conservación Guanacaste, COSTA RICA, 11-SRNP-7011.

#### Paratypes.

6 ♀, 1 ♂ (CNC, NMNH). COSTA RICA: ACG database codes: DHJPAR0038184, DHJPAR0038224, DHJPAR0042041, DHJPAR0042043, DHJPAR0042044, DHJPAR0042062, DHJPAR0042425

#### Description.

**Female.** Body color: body mostly dark except for some sternites which may be pale. Antenna color: scape, pedicel, and flagellum dark. Coxae color (pro-, meso-, metacoxa): dark, dark, dark. Femora color (pro-, meso-, metafemur): pale, dark, dark. Tibiae color (pro-, meso-, metatibia): pale, pale, mostly dark but anterior 0.2 or less pale. Tegula and humeral complex color: tegula pale, humeral complex half pale/half dark. Pterostigma color: strongly white. Fore wing veins color: mostly white or entirely transparent. Antenna length/body length: antenna very short, barely or not extending beyond mesosoma length. Body in lateral view: not distinctly flattened dorso–ventrally. Body length (head to apex of metasoma): 2.3–2.4 mm. Fore wing length: 2.5–2.6 mm. Ocular–ocellar line/posterior ocellus diameter: 2.6 or more. Interocellar distance/posterior ocellus diameter: 2.0–2.2. Antennal flagellomerus 2 length/width: 1.7–1.9. Antennal flagellomerus 14 length/width: 1.1–1.3. Length of flagellomerus 2/length of flagellomerus 14: 1.7–1.9. Tarsal claws: simple. Metafemur length/width: 2.8–2.9. Metatibia inner spur length/metabasitarsus length: 0.4–0.5. Anteromesoscutum: mostly with shallow, dense punctures (separated by less than 2.0 × its maximum diameter). Mesoscutellar disc: mostly smooth. Number of pits in scutoscutellar sulcus: 7 or 8. Maximum height of mesoscutellum lunules/maximum height of lateral face of mesoscutellum: 0.6–0.7. Propodeum areola: completely defined by carinae, including transverse carina extending to spiracle. Propodeum background sculpture: partly sculptured, especially on anterior 0.5. Mediotergite 1 length/width at posterior margin: 2.9–3.1. Mediotergite 1 shape: mostly parallel–sided for 0.5–0.7 of its length, then narrowing posteriorly so mediotergite anterior width >1.1 × posterior width. Mediotergite 1 sculpture: mostly smooth. Mediotergite 2 width at posterior margin/length: 3.2–3.5. Mediotergite 2 sculpture: mostly smooth. Outer margin of hypopygium: with a wide, medially folded, transparent, semi–desclerotized area; usually with 4 or more pleats. Ovipositor thickness: anterior width 3.0–5.0 × posterior width (beyond ovipositor constriction). Ovipositor sheaths length/metatibial length: 0.4–0.5. Length of fore wing veins r/2RS: 1.7–1.9. Length of fore wing veins 2RS/2M: 1.1–1.3. Length of fore wing veins 2M/(RS+M)b: 0.5–0.6. Pterostigma length/width: 2.6–3.0. Point of insertion of vein r in pterostigma: about half way point length of pterostigma. Angle of vein r with fore wing anterior margin: more or less perpendicular to fore wing margin. Shape of junction of veins r and 2RS in fore wing: distinctly but not strongly angled.

**Male.** The specimen available for study is in poor condition, but resemble the females.

#### Molecular data.

Sequences in BOLD: 10, barcode compliant sequences: 10.

#### Biology/ecology.

Solitary. Hosts: Crambidae, *Omiodes cuniculalis*, *Prenesta* Janzen196.

#### Comments.

This species is characterized by a very distinctive hypopygium (with a relatively wide fold where no pleats are visible), ovipositor sheaths (very short and shaped as a broad spatula) and ovipositor (short and strongly curved downwards); it is further distinguished by antenna much shorter than body, white pterostigma, white or transparent fore wing veins, and elongate glossa. The unique hypopygium, ovipositor sheaths, and ovipositor, suggest that this species may be placed in a new genus when there are more studies on the phylogeny of Microgastrinae. Because that is beyond the scope of this paper, we describe this species in *Apanteles*.

#### Etymology.

We dedicate this species to Aida López in recognition of her diligent efforts in the Programa del Comedor Santa Rosa.

### Apanteles albanjimenezi

Fernández-Triana sp. n.

http://zoobank.org/83A7F359-BE52-4B20-8F39-5041CC71FDA9

http://species-id.net/wiki/Apanteles_albanjimenezi

Fig. 101

#### Type locality.

COSTA RICA, Guanacaste, ACG, Sector Cacao, Sendero Cima, 1460m, 10.93328, -85.45729.

#### Holotype.

♀ in CNC. Specimen labels: 1. DHJPAR0012506. 2. 24–31 Aug. 1998, CLC.

#### Description.

Body color: head and mesosoma mostly dark, metasoma with some tergites and/or most of sternites pale. Antenna color: scape, pedicel, and flagellum dark. Coxae color (pro-, meso-, metacoxa): pale, pale, dark. Femora color (pro-, meso-, metafemur): pale, pale, mostly pale but with dark area dorsally. Tibiae color (pro-, meso-, metatibia): pale, pale, anteriorly pale/posteriorly dark. Tegula and humeral complex color: both pale. Pterostigma color: dark. Fore wing veins color: mostly dark (a few veins may be unpigmented). Antenna length/body length: antenna about as long as body (head to apex of metasoma); if slightly shorter, at least extending beyond anterior 0.7 metasoma length. Body in lateral view: not distinctly flattened dorso–ventrally. Body length (head to apex of metasoma): 2.7–2.8 mm. Fore wing length: 2.9–3.0 mm. Ocular–ocellar line/posterior ocellus diameter: 2.3–2.5. Interocellar distance/posterior ocellus diameter: 2.0–2.2. Antennal flagellomerus 2 length/width: 2.3–2.5. Antennal flagellomerus 14 length/width: 1.4–1.6. Length of flagellomerus 2/length of flagellomerus 14: 2.0–2.2. Tarsal claws: simple. Metafemur length/width: 3.2–3.3. Metatibia inner spur length/metabasitarsus length: 0.4–0.5. Anteromesoscutum: mostly with deep, dense punctures (separated by less than 2.0 × its maximum diameter). Mesoscutellar disc: mostly punctured. Number of pits in scutoscutellar sulcus: 5 or 6. Maximum height of mesoscutellum lunules/maximum height of lateral face of mesoscutellum: 0.4–0.5. Propodeum areola: completely defined by carinae, including transverse carina extending to spiracle. Propodeum background sculpture: mostly sculptured. Mediotergite 1 length/width at posterior margin: 2.3–2.5. Mediotergite 1 shape: mostly parallel–sided for 0.5–0.7 of its length, then narrowing posteriorly so mediotergite anterior width >1.1 × posterior width. Mediotergite 1 sculpture: mostly sculptured, excavated area centrally with transverse striation inside and/or a polished knob centrally on posterior margin of mediotergite. Mediotergite 2 width at posterior margin/length: 2.8–3.1. Mediotergite 2 sculpture: more or less fully sculptured, with longitudinal striation. Outer margin of hypopygium: with a wide, medially folded, transparent, semi–desclerotized area; usually with 4 or more pleats. Ovipositor thickness: about same width throughout its length. Ovipositor sheaths length/metatibial length: 1.0–1.1. Length of fore wing veins r/2RS: 1.4–1.6. Length of fore wing veins 2RS/2M: 0.8 or less. Length of fore wing veins 2M/(RS+M)b: 1.1–1.3. Pterostigma length/width: 2.6–3.0. Point of insertion of vein r in pterostigma: about half way point length of pterostigma. Angle of vein r with fore wing anterior margin: more or less perpendicular to fore wing margin. Shape of junction of veins r and 2RS in fore wing: distinctly but not strongly angled.

**Male.** Unknown.

#### Molecular data.

Sequences in BOLD: 4, barcode compliant sequences: 4.

#### Biology/ecology.

Host: Malaise-trapped.

#### Distribution.

Costa Rica, ACG.

#### Etymology.

We dedicate this species to Alban Jiménez in recognition of his diligent efforts for the ACG Programa de Educacion Biológica.

### Apanteles albinervis

(Cameron, 1904) stat. rev.

Fig. 164

Urogaster albinervis Cameron, 1904: 261.
Apanteles albinervican Shenefelt, 1972: 438. Invalid replacement name.

#### Type locality.

MEXICO.

#### Holotype.

♂, BMNH (examined).

#### Description.

**Male.** Body color: body mostly dark except for some sternites which may be pale. Antenna color: scape, pedicel, and flagellum dark. Coxae color (pro-, meso-, metacoxa): dark, dark, dark (?). Femora color (pro-, meso-, metafemur): anteriorly dark/posteriorly pale, dark, dark. Tibiae color (pro-, meso-, metatibia): pale, anteriorly pale/posteriorly dark, anteriorly pale/posteriorly dark. Tegula and humeral complex color: tegula pale, humeral complex half pale/half dark. Pterostigma color: mostly pale and/or transparent, with thin dark borders. Fore wing veins color: mostly white or entirely transparent. Antenna length/body length: antenna about as long as body (head to apex of metasoma); if slightly shorter, at least extending beyond anterior 0.7 metasoma length (?). Body in lateral view: not distinctly flattened dorso–ventrally. Body length (head to apex of metasoma): 2.3–2.4 mm. Fore wing length: 2.5–2.6 mm. Ocular–ocellar line/posterior ocellus diameter: 2.0–2.2. Interocellar distance/posterior ocellus diameter: 1.7–1.9. Tarsal claws: simple (?). Metafemur length/width: 3.0–3.1. Metatibia inner spur length/metabasitarsus length: 0.4–0.5. Anteromesoscutum: mostly with shallow, dense punctures (separated by less than 2.0 × its maximum diameter). Mesoscutellar disc: mostly smooth. Number of pits in scutoscutellar sulcus: 9 or 10. Maximum height of mesoscutellum lunules/maximum height of lateral face of mesoscutellum: 0.6–0.7. Propodeum areola: completely defined by carinae, including transverse carina extending to spiracle. Propodeum background sculpture: partly sculptured, especially on anterior 0.5. Mediotergite 1 length/width at posterior margin: 1.4–1.6. Mediotergite 1 shape: slightly widening from anterior margin to 0.7–0.8 mediotergite length (where maximum width is reached), then narrowing towards posterior margin. Mediotergite 1 sculpture: with some sculpture near lateral margins and/or posterior 0.2–0.4 of mediotergite. Mediotergite 2 width at posterior margin/length: 2.8–3.1. Mediotergite 2 sculpture: mostly smooth. Length of fore wing veins r/2RS: 1.7–1.9. Length of fore wing veins 2RS/2M: 1.4–1.6. Length of fore wing veins 2M/(RS+M)b: 0.5–0.6. Pterostigma length/width: 3.6 or more. Point of insertion of vein r in pterostigma: about half way point length of pterostigma. Angle of vein r with fore wing anterior margin: more or less perpendicular to fore wing margin. Shape of junction of veins r and 2RS in fore wing: distinctly but not strongly angled.

**Female.** Unknown.

#### Molecular data.

No molecular data available for this species.

#### Biology/ecology.

Nothing is known of its hosts.

#### Distribution.

Known only from the male holotype, which was collected in “Mexico”. There is no suggestion that this species occurs in Costa Rica or ACG.

#### Comments.

The history of the name “*Apanteles albinervis*” needs clarification. Cameron (1904) described the species “*Urogaster albinervis*” from Mexico. *Urogaster* was later synonymized under *Apanteles* by Szépligeti (1904), but Cameron’s species was not formally transferred to it until Shenefelt (1972)’s World Catalogue of Hymenoptera. In the meantime, Tobias (1964) had described a species from Kazakhstan as “*Apanteles albinervis*” – later found to be widely distributed in the Palearctic region (Yu et al. 2005) and not related at all to the Mexican species. Acting as the first reviser, Shenefelt realized the problem of a secondary homonym, but mistakenly assigned a replacement name for the oldest (Cameron 1904) instead of the youngest (Tobias 1964) name. As a result, *Apanteles albinervican* Shenefelt, 1972 became a replacement name for *Urogaster albinervis* Cameron, 1904, while *Apanteles albinervis* Tobias, 1964 remained unchanged (Shenefelt 1972). Article 24.2.5 of the International Code of Zoological Nomenclature (ICZN 1999) regulates “Unnecessary action by a First Reviser” and states that “if it is shown subsequently that the precedence of names, spellings or acts can be objectively determined, the action of the First Reviser is nullified”. Thus we consider here *Apanteles albinervican* Shenefelt, 1972 an invalid replacement name for *Apanteles albinervis* (Cameron, 1904) and reinstate the latter name. For details of the revised status of the Tobias species see section “Species excluded from *Apanteles*”.

Another unrelated use of the name “*Apanteles albinervis*”, was by Ashmead (1905), who described a species from the Philippines as “*Urogaster albinervis*”. That became a primary homonym of *Urogaster albinervis* Cameron; however, a replacement name for the Philipine species, *Apanteles lucidinervis*, was provided by Wilkinson (1928).

### Apanteles alejandromasisi

Fernández-Triana sp. n.

http://zoobank.org/C301B64A-5C8A-48FB-9181-C95438CE4EF9

http://species-id.net/wiki/Apanteles_alejandromasisi

Fig. 37

#### Type locality.

COSTA RICA, Guanacaste, ACG, Sector El Hacha, Sendero Bejuquilla, 280 m, 11.03004, -85.52699.

#### Holotype.

♀ in CNC. Specimen labels: 1. DHJPAR0012499. 2. COSTA RICA, Guanacaste, ACG, Sector El Hacha, Sendero Bejuquilla, 280 meters, 11.03004 Longitude: -85.52699, D.H. Janzen &♀ in CNC. Specimen labels: 1. DHJPAR0012499. 2. COSTA RICA, Guanacaste, ACG, Sector El Hacha, Sendero Bejuquilla, 280 meters, 11.03004 Longitude: -85.52699, D.H. Janzen & W. Hallwachs..

#### Description.

**Female.** Body color: head pale, mesosoma extensively pale (anteromesoscutum and scutellar disc). Antenna color: scape and/or pedicel pale, flagellum dark. Coxae color (pro-, meso-, metacoxa): pale, pale, partially pale/partially dark. Femora color (pro-, meso-, metafemur): pale, pale, mostly pale but posterior 0.2 or less dark. Tibiae color (pro-, meso-, metatibia): pale, pale, mostly pale but with posterior 0.2 or less dark. Tegula and humeral complex color: both pale. Pterostigma color: dark. Fore wing veins color: mostly dark (a few veins may be unpigmented). Body in lateral view: not distinctly flattened dorso–ventrally. Body length (head to apex of metasoma): 3.1–3.2 mm. Fore wing length: 3.3–3.4 mm. Ocular–ocellar line/posterior ocellus diameter: 2.3–2.5. Interocellar distance/posterior ocellus diameter: 2.3–2.5. Antennal flagellomerus 2 length/width: 2.6–2.8. Antennal flagellomerus 14 length/width: 1.7–1.9. Length of flagellomerus 2/length of flagellomerus 14: 2.3–2.5. Tarsal claws: with single basal spine–like seta. Metafemur length/width: 2.8–2.9. Anteromesoscutum: mostly with deep, dense punctures (separated by less than 2.0 × its maximum diameter). Mesoscutellar disc: mostly punctured. Number of pits in scutoscutellar sulcus: 5 or 6. Maximum height of mesoscutellum lunules/maximum height of lateral face of mesoscutellum: 0.4–0.5. Propodeum areola: partially defined by carinae on posterior 0.3–0.5 of its length, widely open anteriorly. Propodeum background sculpture: mostly sculptured. Mediotergite 1 length/width at posterior margin: 2.6–2.8. Mediotergite 1 shape: mostly parallel–sided for 0.5–0.7 of its length, then narrowing posteriorly so mediotergite anterior width >1.1 × posterior width. Mediotergite 1 sculpture: mostly sculptured, excavated area centrally with transverse striation inside and/or a polished knob centrally on posterior margin of mediotergite. Mediotergite 2 width at posterior margin/length: 3.2–3.5. Mediotergite 2 sculpture: with some sculpture, mostly near posterior margin. Outer margin of hypopygium: with a wide, medially folded, transparent, semi–desclerotized area; usually with 4 or more pleats. Ovipositor thickness: anterior width at most 2.0 × posterior width (beyond ovipositor constriction). Ovipositor sheaths length/metatibial length: 0.8–0.9. Length of fore wing veins r/2RS: 2.3 or more. Length of fore wing veins 2RS/2M: 0.9–1.0. Length of fore wing veins 2M/(RS+M)b: 0.7–0.8. Pterostigma length/width: 2.6–3.0. Point of insertion of vein r in pterostigma: about half way point length of pterostigma. Angle of vein r with fore wing anterior margin: clearly outwards, inclined towards fore wing apex. Shape of junction of veins r and 2RS in fore wing: distinctly but not strongly angled.

**Male.** Unknown.

#### Molecular data.

Sequences in BOLD: 1, barcode compliant sequences: 1.

#### Biology/ecology.

Malaise trapped.

#### Distribution.

Costa Rica, ACG.

#### Comments.

This species is very distinctive, characterized by head and most of mediotergite 1 orange, mediotergite 3 partially yellow, and mesoscutellar disc mostly punctured.

#### Etymology.

We dedicate this species to Alejandro Masis in recognition of his diligent efforts to administrate and protect the entire ACG.

### Apanteles alejandromorai

Fernández-Triana sp. n.

http://zoobank.org/A0B7FD53-F553-4F81-B94F-838846562A64

http://species-id.net/wiki/Apanteles_alejandromorai

Figs 38 , 234

#### Type locality.

COSTA RICA, Alajuela, ACG, Sector Rincon Rain Forest, Sendero Albergue Crater, 980m, 10.84886, -85.3281.

#### Holotype.

♀ in CNC. Specimen labels: 1. Voucher: D.H.Janzen & W.Hallwachs, DB: http://janzen.sas.upenn.edu, Area de Conservación Guanacaste, COSTA RICA, 09-SRNP-4936. 2. DHJPAR0039759.

#### Paratypes.

3 ♀, 3 ♂ (BMNH, CNC, INBIO, INHS, NMNH). COSTA RICA, ACG database codes: DHJPAR0027612, DHJPAR0035523, DHJPAR0038321, DHJPAR0039030, DHJPAR0039734, DHJPAR0039775.

#### Description.

**Female.** Body color: body mostly dark except for some sternites which may be pale. Antenna color: scape, pedicel, and flagellum dark. Coxae color (pro-, meso-, metacoxa): dark, dark, dark. Femora color (pro-, meso-, metafemur): anteriorly dark/posteriorly pale, dark, dark. Tibiae color (pro-, meso-, metatibia): pale, pale, anteriorly pale/posteriorly dark. Tegula and humeral complex color: tegula pale, humeral complex half pale/half dark. Pterostigma color: dark. Fore wing veins color: mostly dark (a few veins may be unpigmented). Antenna length/body length: antenna about as long as body (head to apex of metasoma); if slightly shorter, at least extending beyond anterior 0.7 metasoma length. Body in lateral view: not distinctly flattened dorso–ventrally. Body length (head to apex of metasoma): 3.3–3.4 mm or 3.5–3.6 mm. Fore wing length: 3.3–3.4 mm, 3.5–3.6 mm or 3.7–3.8 mm. Ocular–ocellar line/posterior ocellus diameter: 2.3–2.5. Interocellar distance/posterior ocellus diameter: 1.4–1.6. Antennal flagellomerus 2 length/width: 2.9–3.1. Antennal flagellomerus 14 length/width: 1.7–1.9. Length of flagellomerus 2/length of flagellomerus 14: 2.3–2.5. Tarsal claws: with single basal spine–like seta. Metafemur length/width: 3.0–3.1. Metatibia inner spur length/metabasitarsus length: 0.4–0.5. Anteromesoscutum: mostly with deep, dense punctures (separated by less than 2.0 × its maximum diameter). Mesoscutellar disc: mostly smooth. Number of pits in scutoscutellar sulcus: 11 or 12. Maximum height of mesoscutellum lunules/maximum height of lateral face of mesoscutellum: 0.6–0.7. Propodeum areola: completely defined by carinae, including transverse carina extending to spiracle. Propodeum background sculpture: mostly sculptured. Mediotergite 1 length/width at posterior margin: 2.3–2.5. Mediotergite 1 shape: mostly parallel–sided for 0.5–0.7 of its length, then narrowing posteriorly so mediotergite anterior width >1.1 × posterior width. Mediotergite 1 sculpture: mostly sculptured, excavated area centrally with transverse striation inside and/or a polished knob centrally on posterior margin of mediotergite. Mediotergite 2 width at posterior margin/length: 2.0–2.3. Mediotergite 2 sculpture: mostly smooth. Outer margin of hypopygium: with a wide, medially folded, transparent, semi–desclerotized area; usually with 4 or more pleats. Ovipositor thickness: about same width throughout its length. Ovipositor sheaths length/metatibial length: 1.8–1.9. Length of fore wing veins r/2RS: 1.7–1.9. Length of fore wing veins 2RS/2M: 1.9–2.0. Length of fore wing veins 2M/(RS+M)b: 0.5–0.6. Pterostigma length/width: 3.6 or more. Point of insertion of vein r in pterostigma: clearly beyond half way point length of pterostigma. Angle of vein r with fore wing anterior margin: more or less perpendicular to fore wing margin. Shape of junction of veins r and 2RS in fore wing: distinctly but not strongly angled.

**Male.** Similar to female, except for mediotergite 2 much less quadrate (i.e., much more transverse).

#### Molecular data.

Sequences in BOLD: 11, barcode compliant sequences: 10.

#### Biology/ecology.

Solitary (Fig. 234). Hosts: Elachistidae, *Antaeotricha* Janzen106, *Antaeotricha* Janzen366, *Lethata trochalosticta*.

#### Distribution.

Costa Rica, ACG.

#### Etymology.

We dedicate this species to Alejandro Mora in recognition of his diligent efforts for the ACG administration and Programa de Contabilidad.

### Apanteles alvarougaldei

Fernández-Triana sp. n.

http://zoobank.org/A19B70E1-1448-4C66-82EA-5C3E7F0A94C7

http://species-id.net/wiki/Apanteles_alvarougaldei

Figs 167 , 304

Apanteles Rodriguez40 (Smith et al. 2006). Interim name provided by the authors.

#### Type locality.

COSTA RICA, Alajuela, ACG, Sector San Cristobal, Puente Palma, 460m, 10.9163, -85.37869.

#### Holotype.

♀ in CNC. Specimen labels: 1. COSTA RICA, Alajuela, ACG, Sector San Cristobal, Puente Palma, 460m, 23.iv.2006, 10.9163, -85.37869, DHJPAR0011962.

#### Paratypes.

22 ♀, 9 ♂ (BMNH, CNC, INBIO, INHS, NMNH). COSTA RICA, ACG database codes: DHJPAR0001584, DHJPAR0002680, DHJPAR0003037, DHJPAR0011962, 93-SRNP-4213.

#### Description.

**Female.** Metatibia color (outer face): entirely or mostly (>0.7 metatibia length) dark brown to black, with yellow to white coloration usually restricted to anterior 0.2 or less. Fore wing veins color: veins C+Sc+R and R1 with brown coloration restricted narrowly to borders, interior area of those veins and pterostigma (and sometimes veins r, 2RS and 2M) transparent or white; other veins mostly transparent. Antenna length/body length: antenna about as long as body (head to apex of metasoma); if slightly shorter, at least extending beyond anterior 0.7 metasoma length. Body length (head to apex of metasoma): 2.3–2.4 mm, 2.5–2.6 mm, rarely 2.1–2.2 mm. Fore wing length: 2.5–2.6 mm or 2.7–2.8 mm. Metafemur length/width: 2.6–2.7, 2.8–2.9 or 3.0–3.1. Mediotergite 1 length/width at posterior margin: 2.3–2.4. Mediotergite 1 maximum width/width at posterior margin: 1.6–1.7. Ovipositor sheaths length/metafemur length: 0.9 or 1.0. Ovipositor sheaths length/metatibia length: 0.8 or 0.9.

#### Molecular data.

Sequences in BOLD: 8, barcode compliant sequences: 7.

#### Biology/ecology.

Gregarious (Fig. 304). Host: Hesperiidae, *Bungalotis quadratum*.

#### Distribution.

Costa Rica, ACG.

#### Etymology.

We dedicate this species to Alvaro Ugalde in recogniton of his diligent efforts in founding and guiding the National Park System of Costa Rica.

### Apanteles anabellecordobae

Fernández-Triana sp. n.

http://zoobank.org/80ACA979-8A0B-4EA2-A5C1-D33A871686F5

http://species-id.net/wiki/Apanteles_anabellecordobae

Figs 51 , 244

Apanteles Rodriguez05 (Smith et al. 2006). Interim name provided by the authors.

#### Type locality.

COSTA RICA, Guanacaste, ACG, Sector Cacao, Sendero Circular, 1185m, 10.92714, -85.46683.

#### Holotype.

♀ in CNC. Specimen labels: 1. COSTA RICA, Guanacaste, ACG, Sector Cacao, Sendero Circular, 01/05/2001, Mariano Pereira. 2. 01-SRNP-6021, Achlyodes selva, Zanthoxylum melanostictum. 3. DHJPAR0001550.

#### Paratypes.

68 ♀, 11 ♂ (BMNH, CNC, INBIO, INHS, NMNH). COSTA RICA: ACG database codes: See Appendix 2 for detailed label data.

#### Description.

**Female.** Body color: body mostly dark except for some sternites which may be pale. Antenna color: scape, pedicel, and flagellum dark. Coxae color (pro-, meso-, metacoxa): dark, dark, dark. Femora color (pro-, meso-, metafemur): anteriorly dark/posteriorly pale, dark, dark. Tibiae color (pro-, meso-, metatibia): pale, pale, anteriorly pale/posteriorly dark. Tegula and humeral complex color: tegula pale, humeral complex dark. Pterostigma color: dark with pale spot at base. Fore wing veins color: mostly dark (a few veins may be unpigmented). Antenna length/body length: antenna about as long as body (head to apex of metasoma); if slightly shorter, at least extending beyond anterior 0.7 metasoma length. Body in lateral view: not distinctly flattened dorso–ventrally. Body length (head to apex of metasoma): 2.7–2.8 mm, 2.9–3.0 mm or 3.1–3.2 mm. Fore wing length: 2.9–3.0 mm, 3.1–3.2 mm or 3.3–3.4 mm. Ocular–ocellar line/posterior ocellus diameter: 1.7–1.9. Interocellar distance/posterior ocellus diameter: 1.4–1.6. Antennal flagellomerus 2 length/width: 2.6–2.8. Antennal flagellomerus 14 length/width: 1.4–1.6. Length of flagellomerus 2/length of flagellomerus 14: 2.0–2.2. Tarsal claws: with single basal spine–like seta. Metafemur length/width: 3.0–3.1. Metatibia inner spur length/metabasitarsus length: 0.4–0.5. Anteromesoscutum: mostly with deep, dense punctures (separated by less than 2.0 × its maximum diameter). Mesoscutellar disc: with punctures near margins, central part mostly smooth. Number of pits in scutoscutellar sulcus: 9 or 10. Maximum height of mesoscutellum lunules/maximum height of lateral face of mesoscutellum: 0.6–0.7. Propodeum areola: completely defined by carinae, including transverse carina extending to spiracle. Propodeum background sculpture: mostly sculptured. Mediotergite 1 length/width at posterior margin: 4.1 or more. Mediotergite 1 shape: mostly parallel–sided for 0.5–0.7 of its length, then narrowing posteriorly so mediotergite anterior width >1.1 × posterior width. Mediotergite 1 sculpture: with some sculpture near lateral margins and/or posterior 0.2–0.4 of mediotergite. Mediotergite 2 width at posterior margin/length: 2.8–3.1. Mediotergite 2 sculpture: mostly smooth. Outer margin of hypopygium: inflexible (without a folded, transparent, semi–desclerotized area); with no pleats visible. Ovipositor thickness: anterior width 3.0–5.0 × posterior width (beyond ovipositor constriction). Ovipositor sheaths length/metatibial length: 0.8–0.9. Length of fore wing veins r/2RS: 2.3 or more. Length of fore wing veins 2RS/2M: 1.1–1.3. Length of fore wing veins 2M/(RS+M)b: 0.7–0.8. Pterostigma length/width: 3.1–3.5. Point of insertion of vein r in pterostigma: about half way point length of pterostigma. Angle of vein r with fore wing anterior margin: more or less perpendicular to fore wing margin. Shape of junction of veins r and 2RS in fore wing: distinctly but not strongly angled.

**Male.** Similar to female.

#### Molecular data.

Sequences in BOLD: 29, barcode compliant sequences: 24, haplotypes: 2.

#### Biology/ecology.

Gregarious (Fig. 244). Hosts: Hesperiidae, *Achlyodes busirus*, *Achlyodes pallida*.

#### Distribution.

Costa Rica, ACG.

#### Coments.

Five specimens (4 ♀ and 1 ♂ )were reared from the same *Achlyodes pallida* caterpillar as the barcoded specimen DHJPAR0005308 (all of them with ACG code 97-SRNP-984). The sequenced specimen clusters apart from the rest of the species, differing by 2.45 % base pairs. Additionally, the locality for specimens with code 97-SRNP-984 is at a lower altitude (90m) compared with the rest of the species (mostly found between 500-1140 m, with only four specimens between 280-290m). Those five specimens might represent a different species, but lacking the support of morphological data and host records (which do not seem to differ) we have decided to keep them under this species for the time being. We have, however, excluded them from the paratypes series.

#### Etymology.

We dedicate this species to Anabelle Cordoba in recognition of her diligent efforts for the ACG Programa de Parataxónomos and Estación Biológica Caribe of ACG.

### Apanteles anamarencoae

Fernández-Triana sp. n.

http://zoobank.org/37760B31-AC9E-401E-A945-26BF952A6738

http://species-id.net/wiki/Apanteles_anamarencoae

Fig. 65

#### Type locality.

COSTA RICA, Guanacaste, ACG,

#### Holotype.

♀ in CNC. Specimen labels: 1. DHJPAR0041897.

#### Paratypes.

2 ♀ (CNC). COSTA RICA: Guanacaste, ACG database code: DHJPAR0041983.

#### Description.

**Female.** Body color: body mostly dark except for some sternites which may be pale. Antenna color: scape, pedicel, and flagellum dark. Coxae color (pro-, meso-, metacoxa): dark, dark, dark. Femora color (pro-, meso-, metafemur): pale, pale, mostly pale but posterior 0.2 or less dark. Tibiae color (pro-, meso-, metatibia): pale, pale, mostly pale but with posterior 0.2 or less dark. Tegula and humeral complex color: both pale. Pterostigma color: dark with pale spot at base. Fore wing veins color: partially pigmented (a few veins may be dark but most are pale). Antenna length/body length: antenna about as long as body (head to apex of metasoma); if slightly shorter, at least extending beyond anterior 0.7 metasoma length. Body in lateral view: not distinctly flattened dorso–ventrally. Body length (head to apex of metasoma): 3.3–3.4 mm. Fore wing length: 3.5–3.6 mm. Ocular–ocellar line/posterior ocellus diameter: 2.0–2.2. Interocellar distance/posterior ocellus diameter: 1.7–1.9. Antennal flagellomerus 2 length/width: 2.9–3.1. Antennal flagellomerus 14 length/width: 1.7–1.9. Length of flagellomerus 2/length of flagellomerus 14: 2.3–2.5. Tarsal claws: simple. Metafemur length/width: 3.0–3.1. Metatibia inner spur length/metabasitarsus length: 0.4–0.5. Anteromesoscutum: mostly with deep, dense punctures (separated by less than 2.0 × its maximum diameter). Mesoscutellar disc: mostly smooth. Number of pits in scutoscutellar sulcus: 11 or 12. Maximum height of mesoscutellum lunules/maximum height of lateral face of mesoscutellum: 0.6–0.7. Propodeum areola: completely defined by carinae, including transverse carina extending to spiracle. Propodeum background sculpture: mostly sculptured. Mediotergite 1 length/width at posterior margin: 1.7–1.9. Mediotergite 1 shape: more or less parallel–sided. Mediotergite 1 sculpture: mostly sculptured, excavated area centrally with transverse striation inside and/or a polished knob centrally on posterior margin of mediotergite. Mediotergite 2 width at posterior margin/length: 2.8–3.1. Mediotergite 2 sculpture: mostly smooth. Outer margin of hypopygium: with a wide, medially folded, transparent, semi–desclerotized area; usually with 4 or more pleats. Ovipositor thickness: about same width throughout its length. Ovipositor sheaths length/metatibial length: 2.0 or more. Length of fore wing veins r/2RS: 2.0–2.2. Length of fore wing veins 2RS/2M: 1.4–1.6. Length of fore wing veins 2M/(RS+M)b: 0.5–0.6. Pterostigma length/width: 3.1–3.5. Point of insertion of vein r in pterostigma: clearly beyond half way point length of pterostigma. Angle of vein r with fore wing anterior margin: more or less perpendicular to fore wing margin. Shape of junction of veins r and 2RS in fore wing: distinctly but not strongly angled.

**Male.** Unknown.

#### Molecular data.

Sequences in BOLD: 6, barcode compliant sequences: 6.

#### Biology/ecology.

Solitary. Hosts: Elachistidae, elachjanzen01 Janzen120, *Antaeotricha* Janzen146, Oecophoridae, *Inga* biolep146DHJ01.

#### Distribution.

Costa Rica, ACG.

#### Etymology.

We dedicate this species to Ana Marenco in recognition of her diligent efforts for the ACG office administration in Sector Santa Rosa.

### Apanteles anamartinezae

Fernández-Triana sp. n.

http://zoobank.org/9BE69016-4E47-4E77-8684-E583304C8932

http://species-id.net/wiki/Apanteles_anamartinezae

Figs 25 , 227

Apanteles Rodriguez51 (Smith et al. 2006). Interim name provided by the authors.

#### Type locality.

COSTA RICA, Alajuela, ACG, Sector San Cristobal, Rio Blanco Abajo, 500m, 10.90037, -85.37254.

#### Holotype.

♀ in CNC. Specimen labels: 1. Costa Rica: Alajuela, ACG, Sector San Cristobal, Rio Blanco Abajo, 15.ii.2007, 500m, 10.90037, -85.37254, DHJPAR0012998.

#### Paratypes.

22 ♀, 9 ♂ (BMNH, CNC, INBIO, INHS, NMNH). COSTA RICA, ACG database codes: DHJPAR0002154, DHJPAR0002227, DHJPAR0004974 DHJPAR0012976, DHJPAR0020130, DHJPAR0031090, 99-SRNP-4439, 03-SRNP-5327.

#### Description.

**Female.** Body color: body mostly dark except for some sternites which may be pale. Antenna color: scape, pedicel, and flagellum dark. Coxae color (pro-, meso-, metacoxa): dark, dark, dark. Femora color (pro-, meso-, metafemur): anteriorly dark/posteriorly pale, dark, dark. Tibiae color (pro-, meso-, metatibia): pale, anteriorly pale/posteriorly dark, dark. Tegula and humeral complex color: tegula pale, humeral complex half pale/half dark. Pterostigma color: mostly pale and/or transparent, with thin dark borders. Fore wing veins color: partially pigmented (a few veins may be dark but most are pale). Antenna length/body length: antenna about as long as body (head to apex of metasoma); if slightly shorter, at least extending beyond anterior 0.7 metasoma length. Body in lateral view: not distinctly flattened dorso–ventrally. Body length (head to apex of metasoma): 2.1–2.2 mm, 2.3–2.4 mm or 2.5–2.6 mm. Fore wing length: 2.1–2.2 mm, 2.3–2.4 mm or 2.5–2.6 mm. Ocular–ocellar line/posterior ocellus diameter: 2.0–2.2. Interocellar distance/posterior ocellus diameter: 1.7–1.9. Antennal flagellomerus 2 length/width: 2.0–2.2. Antennal flagellomerus 14 length/width: 1.7–1.9. Length of flagellomerus 2/length of flagellomerus 14: 3.2 or more. Tarsal claws: with single basal spine–like seta. Metafemur length/width: 3.4–3.5. Metatibia inner spur length/metabasitarsus length: 0.4–0.5. Anteromesoscutum: mostly with deep, dense punctures (separated by less than 2.0 × its maximum diameter). Mesoscutellar disc: mostly smooth. Number of pits in scutoscutellar sulcus: 7 or 8. Maximum height of mesoscutellum lunules/maximum height of lateral face of mesoscutellum: 0.6–0.7. Propodeum areola: completely defined by carinae, including transverse carina extending to spiracle. Propodeum background sculpture: partly sculptured, especially on anterior 0.5. Mediotergite 1 length/width at posterior margin: 1.4–1.6. Mediotergite 1 shape: more or less parallel–sided. Mediotergite 1 sculpture: mostly sculptured, excavated area centrally with transverse striation inside and/or a polished knob centrally on posterior margin of mediotergite. Mediotergite 2 width at posterior margin/length: 4.0–4.3. Mediotergite 2 sculpture: mostly smooth. Outer margin of hypopygium: with a wide, medially folded, transparent, semi–desclerotized area; usually with 4 or more pleats. Ovipositor thickness: about same width throughout its length. Ovipositor sheaths length/metatibial length: 0.6–0.7 or 0.8–0.9. Length of fore wing veins r/2RS: 1.7–1.9. Length of fore wing veins 2RS/2M: 1.4–1.6. Length of fore wing veins 2M/(RS+M)b: 0.7–0.8. Pterostigma length/width: 3.1–3.5. Point of insertion of vein r in pterostigma: about half way point length of pterostigma. Angle of vein r with fore wing anterior margin: clearly outwards, inclined towards fore wing apex. Shape of junction of veins r and 2RS in fore wing: distinctly but not strongly angled.

**Male.** As in female, with slender mediotergite 1.

#### Molecular data.

Sequences in BOLD: 101, barcode compliant sequences: 98.

#### Biology/ecology.

Gregarious (Fig. 227). Hosts: Attevidae, *Atteva aurea*, *Atteva pustulella*, *Atteva zebra*.

#### Distribution.

Costa Rica, ACG.

#### Comments.

Although this species is clearly gregarious, in a few cases only one wasp cocoon is encountered, owing to the small size of the host caterpillar, which may support just one wasp larva, or just an artefact of the lightly silked cocoons falling apart, thus reducing the actual number of cocoons per caterpillar that are encountered when sampling.

#### Etymology.

We dedicate this species to Ana Martínez in recognition of her diligent efforts for the ACG Programa de Contabilidad.

### Apanteles anapiedrae

Fernández-Triana sp. n.

http://zoobank.org/92E5C788-4C54-45AC-8092-E3A8EDBDFBA2

http://species-id.net/wiki/Apanteles_anapiedrae

Figs 67 , 257

Apanteles Rodriguez156 (Smith et al. 2006). Interim name provided by the authors.

#### Type locality.

COSTA RICA, Alajuela, ACG, Sector San Cristobal, Finca San Gabriel, 645m, 10.87766, -85.39343.

#### Holotype.

♀ in CNC. Specimen labels: 1. COSTA RICA, ACG, Sector San Cristobal, Finca S. Gabriel, 645m, DHJPAR0039721.

#### Paratypes.

17 ♀, 5 ♂ (BMNH, CNC, INBIO, INHS, NMNH). COSTA RICA, Alajuela, ACG database codes: DHJPAR0039721, 09-SRNP-3890, 10-SRNP-1054.

#### Description.

**Female.** Body color: body mostly dark except for some sternites which may be pale. Antenna color: scape and/or pedicel pale, flagellum dark. Coxae color (pro-, meso-, metacoxa): dark, dark, dark. Femora color (pro-, meso-, metafemur): anteriorly dark/posteriorly pale, dark, dark. Tibiae color (pro-, meso-, metatibia): pale, pale, dark. Tegula and humeral complex color: both dark. Pterostigma color: dark. Fore wing veins color: mostly dark (a few veins may be unpigmented). Antenna length/body length: antenna shorter than body (head to apex of metasoma), not extending beyond anterior 0.7 metasoma length. Body in lateral view: distinctly flattened dorso–ventrally. Body length (head to apex of metasoma): 2.0 mm or less. Fore wing length: 2.0 mm or less. Ocular–ocellar line/posterior ocellus diameter: 2.6 or more. Interocellar distance/posterior ocellus diameter: 1.4–1.6. Antennal flagellomerus 2 length/width: 2.0–2.2. Antennal flagellomerus 14 length/width: 1.4–1.6. Length of flagellomerus 2/length of flagellomerus 14: 1.7–1.9. Tarsal claws: simple. Metafemur length/width: 2.5 or less. Metatibia inner spur length/metabasitarsus length: 0.4–0.5. Anteromesoscutum: mostly smooth or with shallow sparse punctures, except for anterior 0.3 where it has deeper and/or denser punctures. Mesoscutellar disc: mostly smooth. Number of pits in scutoscutellar sulcus: 11 or 12. Maximum height of mesoscutellum lunules/maximum height of lateral face of mesoscutellum: 0.2–0.3. Propodeum areola: partially defined by carinae on posterior 0.3–0.5 of its length, widely open anteriorly. Propodeum background sculpture: mostly sculptured. Mediotergite 1 length/width at posterior margin: 2.3–2.5. Mediotergite 1 shape: slightly widening from anterior margin to 0.7–0.8 mediotergite length (where maximum width is reached), then narrowing towards posterior margin. Mediotergite 1 sculpture: with some sculpture near lateral margins and/or posterior 0.2–0.4 of mediotergite. Mediotergite 2 width at posterior margin/length: 2.8–3.1. Mediotergite 2 sculpture: mostly smooth. Outer margin of hypopygium: inflexible (without a folded, transparent, semi–desclerotized area); with no pleats visible. Ovipositor thickness: anterior width at most 2.0 × posterior width (beyond ovipositor constriction) (?). Ovipositor sheaths length/metatibial length: 0.4–0.5. Length of fore wing veins r/2RS: 1.4–1.6. Length of fore wing veins 2RS/2M: 0.8 or less. Length of fore wing veins 2M/(RS+M)b: 1.1–1.3. Pterostigma length/width: 3.1–3.5. Point of insertion of vein r in pterostigma: about half way point length of pterostigma. Angle of vein r with fore wing anterior margin: more or less perpendicular to fore wing margin. Shape of junction of veins r and 2RS in fore wing: strongly angulated, sometimes with a knob.

**Male.** As in female.

#### Molecular data.

Sequences in BOLD: 6, barcode compliant sequences: 6.

#### Biology/ecology.

Gregarious (Fig. 257). Hosts: Tortricidae, *Aesiocopa necrofolia*.

#### Distribution.

Costa Rica, ACG.

#### Etymology.

We dedicate this species to Ana Piedra in recognition of her diligent efforts for the ACG Programa de Educacion Biológica.

#### Comments.

*Apanteles anapiedrae* shares with the *diatraeae* and *guadaluperodriguezae* groups a somewhat depressed body (dorso-ventrally), short antenna, and relatively small body size; however, it has an inflexible (unfolded) hypopygium without any pleats, a very small smooth area on lateral face of scutellum (0.2 × as high as maximum height of lateral face), and parasitizes a completely different group of Lepidoptera. The sculpture of propodeum and the areola shape are similar to species of the *diatraeae* group (but the latter group has a pleated hypopygium, a longer ovipositor, and the smooth area on lateral face of scutellum is at least 0.5 × as high as maximum height of lateral face). *Apanteles anapiedrae* does not resemble typical species of *Apanteles* because of its propodeal areola and unpleated hypopygium. It is likely to represent a derived species-group within *Apanteles*, or it might be placed in another genus. Pending further study of worldwide genera of Microgastrinae, we decided to describe the species under *Apanteles* because is the closest match at the moment.

### Apanteles anariasae

Fernández-Triana sp. n.

http://zoobank.org/6ABE9F0E-2996-4580-8943-F7216EFF341F

http://species-id.net/wiki/Apanteles_anariasae

Fig. 71

#### Type locality.

COSTA RICA, Guanacaste, ACG, Sector Santa Rosa, Bosque San Emilio, 300m, 10.84389, -85.61384.

#### Holotype.

♀ in CNC. Specimen labels: 1. DHJPAR0013054. 2. 24 Apr. 2000, San Emilio Trap.

#### Description.

**Female.** Body color: body mostly dark except for some sternites which may be pale. Antenna color: scape, pedicel, and flagellum dark. Coxae color (pro-, meso-, metacoxa): dark, dark, dark. Femora color (pro-, meso-, metafemur): anteriorly dark/posteriorly pale, dark, dark. Tibiae color (pro-, meso-, metatibia): pale, pale, anteriorly pale/posteriorly dark. Tegula and humeral complex color: both dark. Pterostigma color: mostly pale and/or transparent, with thin dark borders. Fore wing veins color: partially pigmented (a few veins may be dark but most are pale). Antenna length/body length: antenna shorter than body (head to apex of metasoma), not extending beyond anterior 0.7 metasoma length. Body in lateral view: not distinctly flattened dorso–ventrally. Body length (head to apex of metasoma): 2.0 mm or less. Fore wing length: 2.0 mm or less. Ocular–ocellar line/posterior ocellus diameter: 2.0–2.2. Interocellar distance/posterior ocellus diameter: 1.7–1.9. Antennal flagellomerus 2 length/width: 2.6–2.8. Antennal flagellomerus 14 length/width: 1.1–1.3. Length of flagellomerus 2/length of flagellomerus 14: 2.0–2.2. Tarsal claws: simple. Metafemur length/width: 2.8–2.9. Metatibia inner spur length/metabasitarsus length: 0.4–0.5. Anteromesoscutum: mostly with deep, dense punctures (separated by less than 2.0 × its maximum diameter). Mesoscutellar disc: mostly smooth. Number of pits in scutoscutellar sulcus: 11 or 12. Maximum height of mesoscutellum lunules/maximum height of lateral face of mesoscutellum: 0.6–0.7. Propodeum areola: completely defined by carinae, including transverse carina extending to spiracle. Propodeum background sculpture: partly sculptured, especially on anterior 0.5. Mediotergite 1 length/width at posterior margin: 1.1–1.3. Mediotergite 1 shape: slightly widening from anterior margin to 0.7–0.8 mediotergite length (where maximum width is reached), then narrowing towards posterior margin. Mediotergite 1 sculpture: mostly sculptured, excavated area centrally with transverse striation inside and/or a polished knob centrally on posterior margin of mediotergite. Mediotergite 2 width at posterior margin/length: 4.4–4.7. Mediotergite 2 sculpture: with some sculpture, mostly near posterior margin. Outer margin of hypopygium: with a wide, medially folded, transparent, semi–desclerotized area; usually with 4 or more pleats. Ovipositor thickness: about same width throughout its length. Ovipositor sheaths length/metatibial length: 1.0–1.1. Length of fore wing veins r/2RS: 1.0 or less. Length of fore wing veins 2RS/2M: 1.4–1.6. Length of fore wing veins 2M/(RS+M)b: 0.9–1.0. Pterostigma length/width: 2.6–3.0. Point of insertion of vein r in pterostigma: about half way point length of pterostigma. Angle of vein r with fore wing anterior margin: more or less perpendicular to fore wing margin. Shape of junction of veins r and 2RS in fore wing: distinctly but not strongly angled.

**Male.** Unknown.

#### Molecular data.

Sequences in BOLD: 1, barcode compliant sequences: 1.

#### Biology/ecology.

Malaise trapped.

#### Distribution.

Costa Rica, ACG.

#### Etymology.

We dedicate this species to Ana Arias in recognition of her diligent efforts for the ACG Comedor in Santa Rosa.

### Apanteles andreacalvoae

Fernández-Triana sp. n.

http://zoobank.org/176A4F60-5DB6-46F3-B83C-185F123842FB

http://species-id.net/wiki/Apanteles_andreacalvoae

Figs 68 , 258

Apanteles Rodriguez10 (Smith et al. 2006). Interim name provided by the authors.

#### Type locality.

COSTA RICA, Guanacaste, ACG, Sector Cacao, Estación Gongora, 570m, 10.88700, -85.47443.

#### Holotype.

♀ in CNC. Specimen labels: 1. COSTA RICA, Guanacaste, ACG, Sector Cacao, Estación Gongora, 14.vi.2004, 570m, 10.88700, -85.47443, 04-SRNP-45868. 2. DHJPAR0001602.

#### Paratypes.

20 ♀, 6 ♂ (BMNH, CNC, INBIO, INHS, NMNH). COSTA RICA: ACG database codes: 04-SRNP-33636, 04-SRNP-45868, 09-SRNP-31397, DHJPAR0001605, DHJPAR0013013, DHJPAR0035406.

#### Description.

**Female.** Body color: body mostly dark except for some sternites which may be pale. Antenna color: scape, pedicel, and flagellum dark. Coxae color (pro-, meso-, metacoxa): dark, dark, dark. Femora color (pro-, meso-, metafemur): anteriorly dark/posteriorly pale, dark, dark. Tibiae color (pro-, meso-, metatibia): pale, pale, mostly pale but with posterior 0.2 or less dark. Tegula and humeral complex color: both dark. Pterostigma color: dark. Fore wing veins color: mostly dark (a few veins may be unpigmented). Antenna length/body length: antenna about as long as body (head to apex of metasoma); if slightly shorter, at least extending beyond anterior 0.7 metasoma length. Body in lateral view: not distinctly flattened dorso–ventrally. Body length (head to apex of metasoma): 2.0 mm or less, 2.5–2.6 mm, rarely 2.1–2.2 mm. Fore wing length: 2.1–2.2 mm or 2.7–2.8 mm. Ocular–ocellar line/posterior ocellus diameter: 2.0–2.2. Interocellar distance/posterior ocellus diameter: 1.7–1.9. Antennal flagellomerus 2 length/width: 3.2 or more. Antennal flagellomerus 14 length/width: 2.0–2.2. Length of flagellomerus 2/length of flagellomerus 14: 2.0–2.2. Tarsal claws: with single basal spine–like seta. Metafemur length/width: 3.2–3.3. Metatibia inner spur length/metabasitarsus length: 0.6–0.7. Anteromesoscutum: mostly with deep, dense punctures (separated by less than 2.0 × its maximum diameter). Mesoscutellar disc: mostly smooth. Number of pits in scutoscutellar sulcus: 7 or 8. Maximum height of mesoscutellum lunules/maximum height of lateral face of mesoscutellum: 0.6–0.7. Propodeum areola: completely defined by carinae, including transverse carina extending to spiracle. Propodeum background sculpture: partly sculptured, especially on anterior 0.5. Mediotergite 1 length/width at posterior margin: 2.9–3.1. Mediotergite 1 shape: more or less parallel–sided. Mediotergite 1 sculpture: with some sculpture near lateral margins and/or posterior 0.2–0.4 of mediotergite. Mediotergite 2 width at posterior margin/length: 2.8–3.1. Mediotergite 2 sculpture: mostly smooth. Outer margin of hypopygium: inflexible (without a folded, transparent, semi–desclerotized area); with no pleats visible. Ovipositor thickness: anterior width at most 2.0 × posterior width (beyond ovipositor constriction). Ovipositor sheaths length/metatibial length: 0.8–0.9. Length of fore wing veins r/2RS: 2.0–2.2. Length of fore wing veins 2RS/2M: 1.1–1.3. Length of fore wing veins 2M/(RS+M)b: 0.9–1.0. Pterostigma length/width: 3.6 or more. Point of insertion of vein r in pterostigma: about half way point length of pterostigma. Angle of vein r with fore wing anterior margin: more or less perpendicular to fore wing margin. Shape of junction of veins r and 2RS in fore wing: distinctly but not strongly angled.

**Male.** Similar to female.

#### Molecular data.

Sequences in BOLD: 7, barcode compliant sequences: 6.

#### Biology/ecology.

Gregarious (Fig. 258). Hosts: Hesperiidae, *Perichares geonomaphaga*, *Perichares prestoeaphaga*, *Perichares poaceaphaga*.

#### Distribution.

Costa Rica, ACG.

#### Comments.

Adult show discontinuous variation in body length (ranges: 2.0–2.2 mm, 2.5–2.6 mm) and in fore wing length (2.1–2.2 mm or 2.7–2.8 mm). This is an unusual pattern among the Mesoamerican species of *Apanteles* we have examined so far, but might reflect the size of the caterpillar host when parasitized. Because we have not found consistent differences among the specimens other than size, we keep them as the same species. Also, this species has an inflexible (unfolded) hypopygium. Unlike other species with similar type of hypopygium (all of which belong to the *anabellecordobae* species-group); the ovipositor of *andracalvoae* is thin (thinner than width of median flagellomerus), and with basal width <2.0 × its apical width after constriction. It can be differenced from other species with thinner ovipositor by having all coxae, profemur partially, and meso- and meta- femora completely, dark brown to black, and mesoscutellar disc mostly smooth.

#### Etymology.

We dedicate this species to Andrea Calvo in recognition of her diligent efforts for the ACG Department of Human Resources.

### Apanteles angelsolisi

Fernández-Triana sp. n.

http://zoobank.org/97A13CA1-B037-40CA-A134-3D2F7F1BF74F

http://species-id.net/wiki/Apanteles_angelsolisi

Figs 170 , 305

Apanteles Rodriguez27 (Smith et al. 2006). Interim name provided by the authors.

#### Type locality.

COSTA RICA, Guanacaste, ACG, Sector Santa Rosa, Quebrada Guapote, 240m, 10.82690, -85.60413.

#### Holotype.

♀ in CNC. Specimen labels: 1. COSTA RICA, Guanacaste, ACG, Sector Santa Rosa, Quebrada Guapote, 07.viii.1996, 240m, 10.82690, -85.60413, DHJPAR0004186.

#### Paratypes.

58 ♀, 19 ♂ (BMNH, CNC, INBIO, INHS, NMNH). COSTA RICA, ACG database codes: See Appendix 2 for detailed label data.

#### Description.

**Female.** Metatibia color (outer face): with extended pale coloration (light yellow to orange–yellow), ranging from 0.4 to almost entire metatibia length. Fore wing veins color: veins C+Sc+R and R1 with brown coloration restricted narrowly to borders, interior area of those veins and pterostigma (and sometimes veins r, 2RS and 2M) transparent or white; other veins mostly transparent. Antenna length/body length: antenna about as long as body (head to apex of metasoma); if slightly shorter, at least extending beyond anterior 0.7 metasoma length. Body length (head to apex of metasoma): 2.3–2.4 mm, 2.5–2.6 mm, rarely 2.1–2.2 mm. Fore wing length: 2.3–2.4 mm, 2.5–2.6 mm, rarely 2.1–2.2 mm. Metafemur length/width: 2.8–2.9 or 3.0–3.1. Mediotergite 1 length/width at posterior margin: 2.5–2.6. Mediotergite 1 maximum width/width at posterior margin: 1.6–1.7. Ovipositor sheaths length/metafemur length: 0.9 or 1.0. Ovipositor sheaths length/metatibia length: 0.7 or 0.8.

#### Molecular data.

Sequences in BOLD: 11, barcode compliant sequences: 9.

#### Biology/ecology.

Gregarious (Fig. 305). Hosts: Hesperiidae, *Achalarus toxeus*, *Thessia jalapus*, *Urbanus albimargo*.

#### Distribution.

Costa Rica, ACG.

#### Etymology.

We dedicate this species to Angel Solís in recognition of his diligent efforts in curation and taxonomy of Coleoptera for INBio, Costa Rica’s Instituto Nacional de Biodiversidad.

### Apanteles arielopezi

Fernández-Triana sp. n.

http://zoobank.org/F8EAA3B7-25DF-4B91-9AC8-9D68B3B5AA62

http://species-id.net/wiki/Apanteles_arielopezi

Figs 69 , 259

#### Type locality.

COSTA RICA, Alajuela, Sector Rincon Rain Forest, Rinconcito.

#### Holotype.

♀ in CNC. Specimen labels: 1. DHJPAR0041762. 2. COSTA RICA, Alajuela, Sector Rincon Rain Forest, Rinconcito, 28.ix.2010, DHJPAR0041762. 3. Voucher: D.H.Janzen & W.Hallwachs, DB: http://janzen.sas.upenn.edu, Area de Conservación Guanacaste, COSTA RICA, 10-SRNP-43550.

#### Paratypes.

41 ♀ (BMNH, CNC, INBIO, INHS, NMNH). COSTA RICA: Guanacaste, ACG database code: 10-SRNP-43550, 10-SRNP-44058.

#### Description.

**Female.** Body color: body mostly dark except for some sternites which may be pale. Antenna color: scape, pedicel, and flagellum dark. Coxae color (pro-, meso-, metacoxa): dark, dark, dark. Femora color (pro-, meso-, metafemur): pale, pale, mostly dark but with pale spot antero–ventrally. Tibiae color (pro-, meso-, metatibia): pale, pale, anteriorly pale/posteriorly dark. Tegula and humeral complex color: tegula pale, humeral complex half pale/half dark. Pterostigma color: mostly dark, with small pale area centrally. Fore wing veins color: mostly dark (a few veins may be unpigmented). Antenna length/body length: antenna shorter than body (head to apex of metasoma), not extending beyond anterior 0.7 metasoma length. Body in lateral view: not distinctly flattened dorso–ventrally. Body length (head to apex of metasoma): 2.3–2.4 mm. Fore wing length: 2.3–2.4 mm. Ocular–ocellar line/posterior ocellus diameter: 2.0–2.2. Interocellar distance/posterior ocellus diameter: 1.4–1.6. Antennal flagellomerus 2 length/width: 1.7–1.9. Antennal flagellomerus 14 length/width: 1.1–1.3. Length of flagellomerus 2/length of flagellomerus 14: 1.7–1.9. Tarsal claws: simple. Metafemur length/width: 2.8–2.9. Metatibia inner spur length/metabasitarsus length: 0.4–0.5. Anteromesoscutum: mostly with deep, dense punctures (separated by less than 2.0 × its maximum diameter). Mesoscutellar disc: mostly smooth. Number of pits in scutoscutellar sulcus: 7 or 8. Maximum height of mesoscutellum lunules/maximum height of lateral face of mesoscutellum: 0.4–0.5. Propodeum areola: completely defined by carinae, including transverse carina extending to spiracle. Propodeum background sculpture: partly sculptured, especially on anterior 0.5. Mediotergite 1 length/width at posterior margin: 2.9–3.1. Mediotergite 1 shape: slightly widening from anterior margin to 0.7–0.8 mediotergite length (where maximum width is reached), then narrowing towards posterior margin. Mediotergite 1 sculpture: with some sculpture near lateral margins and/or posterior 0.2–0.4 of mediotergite. Mediotergite 2 width at posterior margin/length: 4.0–4.3. Mediotergite 2 sculpture: mostly smooth. Outer margin of hypopygium: with a wide, medially folded, transparent, semi–desclerotized area; usually with 4 or more pleats. Ovipositor thickness: about same width throughout its length. Ovipositor sheaths length/metatibial length: 0.8–0.9. Length of fore wing veins r/2RS: 1.1–1.3. Length of fore wing veins 2RS/2M: 1.1–1.3. Length of fore wing veins 2M/(RS+M)b: 0.9–1.0. Pterostigma length/width: 3.1–3.5. Point of insertion of vein r in pterostigma: clearly beyond half way point length of pterostigma. Angle of vein r with fore wing anterior margin: more or less perpendicular to fore wing margin. Shape of junction of veins r and 2RS in fore wing: distinctly but not strongly angled.

**Male.** Unknown.

#### Molecular data.

Sequences in BOLD: 4, barcode compliant sequences: 4.

#### Biology/ecology.

Gregarious (Fig. 259). Hosts: Tortricidae, *Paramorbia* Brown001DHJ03, *Paramorbia* Brown001DHJ01.

#### Distribution.

Costa Rica, ACG.

#### Etymology.

We dedicate this species to Ariel López in recognition of his diligent efforts for the ACG Programa de Sectores.

### Apanteles balthazari

(Ashmead, 1900)

http://species-id.net/wiki/Apanteles_balthazari

Fig. 146

Urogaster balthazari Ashmead, 1900: 284.
Apanteles balthazari (Ashmead, 1900). Transferred by Szépligeti (1904: 109).
Urogaster meridionalis Ashmead, 1900: 285. Synonymized by Muesebeck (1958: 431).

#### Type locality.

ST. VINCENT, Lesser Antilles.

#### Holotype.

♀, BMNH (examined).

#### Description.

**Female.** Body color: body mostly dark except for some sternites which may be pale. Antenna color: scape, pedicel, and flagellum dark. Coxae color (pro-, meso-, metacoxa): dark, dark, dark. Femora color (pro-, meso-, metafemur): pale, pale, pale. Tibiae color (pro-, meso-, metatibia): pale, pale, pale. Tegula and humeral complex color: tegula pale, humeral complex half pale/half dark. Pterostigma color: mostly pale and/or transparent, with thin dark borders. Fore wing veins color: mostly white or entirely transparent. Antenna length/body length: antenna about as long as body (head to apex of metasoma); if slightly shorter, at least extending beyond anterior 0.7 metasoma length. Body in lateral view: not distinctly flattened dorso–ventrally. Body length (head to apex of metasoma): 2.9–3.0 mm. Fore wing length: 3.1–3.2 mm. Ocular–ocellar line/posterior ocellus diameter: 1.7–1.9. Interocellar distance/posterior ocellus diameter: 1.7–1.9. Antennal flagellomerus 2 length/width: 3.2 or more. Antennal flagellomerus 14 length/width: 1.7–1.9. Length of flagellomerus 2/length of flagellomerus 14: 1.7–1.9. Tarsal claws: simple. Metafemur length/width: 2.8–2.9. Metatibia inner spur length/metabasitarsus length: 0.4–0.5. Anteromesoscutum: mostly with deep, dense punctures (separated by less than 2.0 × its maximum diameter). Mesoscutellar disc: mostly smooth. Number of pits in scutoscutellar sulcus: 7 or 8. Maximum height of mesoscutellum lunules/maximum height of lateral face of mesoscutellum: 0.6–0.7. Propodeum areola: completely defined by carinae, including transverse carina extending to spiracle. Propodeum background sculpture: mostly sculptured. Mediotergite 1 length/width at posterior margin: 1.1–1.3. Mediotergite 1 shape: more or less parallel–sided. Mediotergite 1 sculpture: mostly sculptured, excavated area centrally with transverse striation inside and/or a polished knob centrally on posterior margin of mediotergite. Mediotergite 2 width at posterior margin/length: 2.8–3.1. Mediotergite 2 sculpture: more or less fully sculptured, with longitudinal striation. Outer margin of hypopygium: with a wide, medially folded, transparent, semi–desclerotized area; usually with 4 or more pleats. Ovipositor thickness: about same width throughout its length. Ovipositor sheaths length/metatibial length: 1.4–1.5. Length of fore wing veins r/2RS: 1.1–1.3. Length of fore wing veins 2RS/2M: 2.1 or more. Length of fore wing veins 2M/(RS+M)b: 0.5–0.6. Pterostigma length/width: 3.1–3.5. Point of insertion of vein r in pterostigma: clearly beyond half way point length of pterostigma. Angle of vein r with fore wing anterior margin: clearly inwards, inclined towards fore wing base. Shape of junction of veins r and 2RS in fore wing: strongly angulated, sometimes with a knob.

#### Molecular data.

No molecular data available for this species.

#### Biology/ecology.

Hosts: Gelechiidae, *Pectinophora gossypiella*.

#### Distribution.

Brazil, Cuba, Grenada, St. Vincent. There is no suggestion that this species occurs in ACG.

#### Comments.

The original description from Ashmead (1900) does not match the holotype. His description of the T1 shape, T2 sculpture, and coloration of meso and metafemora are completely different from the actual specimen.

### Apanteles bernardoespinozai

Fernández-Triana sp. n.

http://zoobank.org/AB6E338B-0F0F-4AAE-BA14-97AC24C3FE00

http://species-id.net/wiki/Apanteles_bernardoespinozai

Figs 171 , 306

Apanteles Rodriguez23 (Smith et al. 2006). Interim name provided by the authors.

#### Type locality.

COSTA RICA, Alajuela, ACG, Sector Rincon Rain Forest, Sendero Juntas, 400m, 10.90661, -85.28784.

#### Holotype.

♀ in CNC. Specimen labels: 1. DHJPAR0002269. 2. COSTA RICA, Alajuela, ACG, Sector Rincon Rain Forest, Sendero Juntas, 25.viii.2005, 10.90661°N, 85.28784°W, 400m, DHJPAR0002269.

#### Paratypes.

152 ♀, 46 ♂ (BMNH, CNC, INBIO, INHS, NMNH). COSTA RICA, ACG database codes: See Appendix 2 for detailed label data.

#### Description.

**Female.** Metatibia color (outer face): with extended pale coloration (light yellow to orange–yellow), ranging from 0.4 to almost entire metatibia length. Fore wing veins color: veins C+Sc+R and R1 with brown coloration restricted narrowly to borders, interior area of those veins and pterostigma (and sometimes veins r, 2RS and 2M) transparent or white; other veins mostly transparent. Antenna length/body length: antenna about as long as body (head to apex of metasoma); if slightly shorter, at least extending beyond anterior 0.7 metasoma length. Body length (head to apex of metasoma): 2.3–2.4 mm or 2.5–2.6 mm. Fore wing length: 2.5–2.6 mm or 2.7–2.8 mm. Metafemur length/width: 3.0–3.1, 3.2–3.3, rarely 3.4–3.5. Mediotergite 1 length/width at posterior margin: 2.3–2.4. Mediotergite 1 maximum width/width at posterior margin: 1.4–1.5. Ovipositor sheaths length/metafemur length: 0.9 or 1.0. Ovipositor sheaths length/metatibia length: 0.7 or 0.8.

#### Molecular data.

Sequences in BOLD: 59, barcode compliant sequences: 50.

#### Biology/ecology.

Gregarious (Fig. 306). Hosts: Hesperiidae, *Astraptes alardus*, *Astraptes brevicauda*, *Astraptes talus*, *Astraptes tucuti*, *Narcosius samson*, *Urbanus belli*, *Urbanus dorantes*, *Urbanus doryssus*.

#### Distribution.

Costa Rica, ACG.

#### Etymology.

We dedicate this species to Bernardo Espinoza in recognition of his diligent efforts for the ACG Programa de Parataxónomos, and Lepidoptera curatorial taxonomy for INBio, Costa Rica’s Instituto Nacional de Biodiversidad, and for ACG.

### Apanteles bernyapui

Fernández-Triana sp. n.

http://zoobank.org/706706AA-55ED-4F56-80C2-E232AD93A3BA

http://species-id.net/wiki/Apanteles_bernyapui

Fig. 79

Apanteles Rodriguez73. Smith et al. (2008). Interim name provided by the authors.

#### Type locality.

COSTA RICA, Guanacaste, ACG, Sector Santa Rosa, Area Administrativa, 295m, 10.83764, -85.61871.

#### Holotype.

♀ in CNC. Specimen labels: 1. DHJPAR0005188. 2. 95-SRNP-10552.

#### Paratypes.

9 ♀, 4 ♂ (BMNH, CNC, INBIO, INHS, NMNH). COSTA RICA, ACG database codes: DHJPAR0002918, DHJPAR0005173, DHJPAR0005179, DHJPAR0005190, DHJPAR0012498, DHJPAR0012533, DHJPAR0013022, DHJPAR0013031, DHJPAR0013043, DHJPAR0013107, DHJPAR0013205, DHJPAR0024735, DHJPAR0024740.

#### Description.

**Female.** Body color: head dark, mesosoma dark with parts of axillar complex pale, metasoma with some mediotergites, most laterotergites, sternites, and/or hypopygium pale. Antenna color: scape and/or pedicel pale, flagellum dark. Coxae color (pro-, meso-, metacoxa): pale, pale, pale. Femora color (pro-, meso-, metafemur): pale, pale, pale. Tibiae color (pro-, meso-, metatibia): pale, pale, mostly pale but with posterior 0.2 or less dark. Tegula and humeral complex color: both pale. Pterostigma color: dark. Fore wing veins color: mostly dark (a few veins may be unpigmented). Antenna length/body length: antenna about as long as body (head to apex of metasoma); if slightly shorter, at least extending beyond anterior 0.7 metasoma length. Body in lateral view: not distinctly flattened dorso–ventrally. Body length (head to apex of metasoma): 2.5–2.6 mm, 2.7–2.8 mm or 3.1–3.2 mm. Fore wing length: 2.7–2.8 mm or 3.1–3.2 mm. Ocular–ocellar line/posterior ocellus diameter: 2.0–2.2. Interocellar distance/posterior ocellus diameter: 2.0–2.2. Antennal flagellomerus 2 length/width: 2.3–2.5. Antennal flagellomerus 14 length/width: 1.4–1.6. Length of flagellomerus 2/length of flagellomerus 14: 1.7–1.9. Tarsal claws: with single basal spine–like seta. Metafemur length/width: 3.0–3.1. Metatibia inner spur length/metabasitarsus length: 0.4–0.5. Anteromesoscutum: mostly with deep, dense punctures (separated by less than 2.0 × its maximum diameter). Mesoscutellar disc: with punctures near margins, central part mostly smooth. Number of pits in scutoscutellar sulcus: 7 or 8. Maximum height of mesoscutellum lunules/maximum height of lateral face of mesoscutellum: 0.2–0.3. Propodeum areola: completely defined by carinae, including transverse carina extending to spiracle. Propodeum background sculpture: mostly sculptured. Mediotergite 1 length/width at posterior margin: 1.7–1.9. Mediotergite 1 shape: mostly parallel–sided for 0.5–0.7 of its length, then narrowing posteriorly so mediotergite anterior width >1.1 × posterior width. Mediotergite 1 sculpture: mostly sculptured, excavated area centrally with transverse striation inside and/or a polished knob centrally on posterior margin of mediotergite. Mediotergite 2 width at posterior margin/length: 2.8–3.1. Mediotergite 2 sculpture: with some sculpture, mostly near posterior margin. Outer margin of hypopygium: with a wide, medially folded, transparent, semi–desclerotized area; usually with 4 or more pleats. Ovipositor thickness: about same width throughout its length. Ovipositor sheaths length/metatibial length: 0.8–0.9. Length of fore wing veins r/2RS: 1.7–1.9. Length of fore wing veins 2RS/2M: 1.1–1.3. Length of fore wing veins 2M/(RS+M)b: 0.7–0.8. Pterostigma length/width: 3.1–3.5. Point of insertion of vein r in pterostigma: clearly beyond half way point length of pterostigma. Angle of vein r with fore wing anterior margin: clearly outwards, inclined towards fore wing apex. Shape of junction of veins r and 2RS in fore wing: distinctly but not strongly angled.

**Male.** As in female, but with metacoxae dark brown and tergites darker in coloration.

#### Molecular data.

Sequences in BOLD: 28, barcode compliant sequences: 26.

#### Biology/ecology.

Solitary. Hosts: Crambidae, *Eulepte alialis*, *Lygropia tripunctata*, and other *Conchylodes ovulalis*. Also Malaise-trapped.

#### Distribution.

Costa Rica, ACG.

#### Etymology.

We dedicate this species to Berny Apu in reocognition of his diligent efforts for the ACG Programa de Seguridad.

### Apanteles bettymarchenae

Fernández-Triana sp. n.

http://zoobank.org/AD0A0340-E391-423C-82C2-95291DA42E79

http://species-id.net/wiki/Apanteles_bettymarchenae

Fig. 83

#### Type locality.

COSTA RICA, Alajuela, ACG, Sector San Cristobal, Potrero Argentina, 520 m, 10.89021, -85.38803.

#### Holotype.

♀ in CNC. Specimen labels: 1. DHJPAR0025757. 2. San Gerardo: Sitio Argentina, 7–16 Jun. 2007.

#### Paratypes.

1 ♂ (CNC). COSTA RICA: Alajuela, ACG database code: DHJPAR0027469.

#### Description.

**Female.** Body color: body mostly dark except for some sternites which may be pale. Antenna color: scape and/or pedicel pale, flagellum dark. Coxae color (pro-, meso-, metacoxa): pale, pale, pale. Femora color (pro-, meso-, metafemur): pale, pale, pale. Tibiae color (pro-, meso-, metatibia): pale, pale, pale. Tegula and humeral complex color: both pale. Pterostigma color: dark. Fore wing veins color: mostly dark (a few veins may be unpigmented). Antenna length/body length: antenna about as long as body (head to apex of metasoma); if slightly shorter, at least extending beyond anterior 0.7 metasoma length. Body in lateral view: not distinctly flattened dorso–ventrally. Body length (head to apex of metasoma): 2.0 mm or less. Fore wing length: 2.3–2.4 mm. Ocular–ocellar line/posterior ocellus diameter: 2.0–2.2. Interocellar distance/posterior ocellus diameter: 1.4–1.6. Antennal flagellomerus 2 length/width: 2.9–3.1. Antennal flagellomerus 14 length/width: 1.4–1.6. Length of flagellomerus 2/length of flagellomerus 14: 2.3–2.5. Tarsal claws: with single basal spine–like seta. Metafemur length/width: 3.2–3.3. Anteromesoscutum: mostly with shallow, dense punctures (separated by less than 2.0 × its maximum diameter). Mesoscutellar disc: with a few sparse punctures. Number of pits in scutoscutellar sulcus: 5 or 6. Maximum height of mesoscutellum lunules/maximum height of lateral face of mesoscutellum: 0.4–0.5. Propodeum areola: completely defined by carinae, including transverse carina extending to spiracle. Propodeum background sculpture: partly sculptured, especially on anterior 0.5. Mediotergite 1 length/width at posterior margin: 3.8–4.0. Mediotergite 1 shape: clearly narrowing towards posterior margin. Mediotergite 1 sculpture: mostly sculptured, excavated area centrally with transverse striation inside and/or a polished knob centrally on posterior margin of mediotergite. Mediotergite 2 width at posterior margin/length: 3.6–3.9. Mediotergite 2 sculpture: mostly smooth. Outer margin of hypopygium: with a wide, medially folded, transparent, semi–desclerotized area; usually with 4 or more pleats. Ovipositor thickness: about same width throughout its length. Ovipositor sheaths length/metatibial length: 0.8–0.9. Length of fore wing veins r/2RS: 1.4–1.6. Length of fore wing veins 2RS/2M: 1.1–1.3. Length of fore wing veins 2M/(RS+M)b: 0.9–1.0. Pterostigma length/width: 3.1–3.5. Point of insertion of vein r in pterostigma: about half way point length of pterostigma. Angle of vein r with fore wing anterior margin: clearly outwards, inclined towards fore wing apex. Shape of junction of veins r and 2RS in fore wing: strongly angulated, sometimes with a knob.

**Male.** The only male specimen has dark brown metacoxa and metatarsus.

#### Molecular data.

Sequences in BOLD: 2, barcode compliant sequences: 2.

#### Biology/ecology.

Malaise trapped.

#### Distribution.

Costa Rica, ACG

#### Comments.

This species is characterized by the combination of small size (body length 2.0 mm, fore wing length 2.4 mm), and all legs (including coxae) fully yellow or whitish-yellow.

#### Etymology.

We dedicate this species to Betty Marchena in recognition of her diligent efforts for the ACG Programa de Asesoría Legal.

### Apanteles bienvenidachavarriae

Fernández-Triana sp. n.

http://zoobank.org/DD7FE90B-714A-4610-9172-C0B02FB922F0

http://species-id.net/wiki/Apanteles_bienvenidachavarriae

Figs 84 , 264

Apanteles Rodriguez71 (Smith et al. 2006). Interim name provided by the authors.

#### Type locality.

COSTA RICA, Alajuela, ACG, Sector Rincon Rain Forest, Jacobo, 461m, 10.94076, -85.3177.

#### Holotype.

♀ in CNC. Specimen labels: 1. DHJPAR0038167. 2. Voucher: D.H.Janzen & W.Hallwachs, DB: http://janzen.sas.upenn.edu, Area de Conservación Guanacaste, COSTA RICA, 09-SRNP-80097.

#### Paratypes.

6 ♀ (BMNH, CNC, INBIO, NMNH). COSTA RICA, ACG database codes: DHJPAR0035361, DHJPAR0038169, DHJPAR0038223, DHJPAR0039755, DHJPAR0039758, DHJPAR0039766.

#### Description.

**Female.** Body color: body mostly dark except for some sternites which may be pale. Antenna color: scape, pedicel, and flagellum dark. Coxae color (pro-, meso-, metacoxa): dark, dark, dark. Femora color (pro-, meso-, metafemur): pale, anteriorly dark/posteriorly pale, dark. Tibiae color (pro-, meso-, metatibia): pale, pale, anteriorly pale/posteriorly dark. Tegula and humeral complex color: tegula pale, humeral complex half pale/half dark. Pterostigma color: mostly pale and/or transparent, with thin dark borders. Fore wing veins color: partially pigmented (a few veins may be dark but most are pale). Antenna length/body length: antenna about as long as body (head to apex of metasoma); if slightly shorter, at least extending beyond anterior 0.7 metasoma length. Body in lateral view: not distinctly flattened dorso–ventrally. Body length (head to apex of metasoma): 3.7–3.8 mm or 3.9–4.0 mm. Fore wing length: 3.9–4.0 mm. Ocular–ocellar line/posterior ocellus diameter: 2.0–2.2. Interocellar distance/posterior ocellus diameter: 1.4–1.6. Antennal flagellomerus 2 length/width: 2.9–3.1. Antennal flagellomerus 14 length/width: 1.7–1.9. Length of flagellomerus 2/length of flagellomerus 14: 2.0–2.2. Tarsal claws: with single basal spine–like seta. Metafemur length/width: 3.2–3.3. Metatibia inner spur length/metabasitarsus length: 0.4–0.5. Anteromesoscutum: mostly with deep, dense punctures (separated by less than 2.0 × its maximum diameter). Mesoscutellar disc: mostly smooth. Number of pits in scutoscutellar sulcus: 11 or 12. Maximum height of mesoscutellum lunules/maximum height of lateral face of mesoscutellum: 0.6–0.7. Propodeum areola: completely defined by carinae, including transverse carina extending to spiracle. Propodeum background sculpture: mostly sculptured. Mediotergite 1 length/width at posterior margin: 2.9–3.1. Mediotergite 1 shape: mostly parallel–sided for 0.5–0.7 of its length, then narrowing posteriorly so mediotergite anterior width >1.1 × posterior width. Mediotergite 1 sculpture: mostly sculptured, excavated area centrally with transverse striation inside and/or a polished knob centrally on posterior margin of mediotergite. Mediotergite 2 width at posterior margin/length: 2.4–2.7. Mediotergite 2 sculpture: mostly smooth. Outer margin of hypopygium: with a wide, medially folded, transparent, semi–desclerotized area; usually with 4 or more pleats. Ovipositor thickness: anterior width at most 2.0 × posterior width (beyond ovipositor constriction). Ovipositor sheaths length/metatibial length: 1.4–1.5. Length of fore wing veins r/2RS: 1.4–1.6. Length of fore wing veins 2RS/2M: 2.1 or more. Length of fore wing veins 2M/(RS+M)b: 0.4 or less. Pterostigma length/width: 3.1–3.5. Point of insertion of vein r in pterostigma: clearly beyond half way point length of pterostigma. Angle of vein r with fore wing anterior margin: clearly outwards, inclined towards fore wing apex. Shape of junction of veins r and 2RS in fore wing: distinctly but not strongly angled.

**Male.** Unknown.

#### Molecular data.

Sequences in BOLD: 16, barcode compliant sequences: 16.

#### Biology/ecology.

Solitary (Fig. 264). Hosts: Elachistidae, four species of *Anadasmus*.

#### Distribution.

Costa Rica, ACG.

#### Etymology.

We dedicate this species to Bienvenida Chavarría in recognition of her diligent efforts for the ACG Programa de Sectores

### Apanteles calixtomoragai

Fernández-Triana sp. n.

http://zoobank.org/52CFFE3D-CC57-45A2-BCE0-3BCF6F2ADFE9

http://species-id.net/wiki/Apanteles_calixtomoragai

Figs 87 , 266

Apanteles Rodriguez03 (Smith et al. 2006). Interim name provided by the authors.

#### Type locality.

COSTA RICA, Guanacaste, Rincón Rainforest, Camino Río Francia, 410m, 10.90425, -85.28651.

#### Holotype.

♀ in CNC. Specimen labels: 1. COSTA RICA: Guanacaste, Area de Conservación Guanacaste, Rincón Rainforest, Camino Río Francia, 23.viii.2001, Jose Perez. 2. 01-SRNP-5632, ex *Milanion marciana* on *Annona papilionella*.

#### Paratypes.

8 ♀, 5 ♂ (BMNH, CNC, INBIO, INHS, NMNH). COSTA RICA, ACG database codes: 00-SRNP-20822, 01-SRNP-5630, 01-SRNP-5658, 01-SRNP-5661, 01-SRNP-5663, 02-SRNP-7548, 02-SRNP-7624, 03-SRNP-12942.1, 04-SRNP-809, 05-SRNP-189, 05-SRNP-41778, 06-SRNP-44306, 07-SRNP-40064.

#### Description.

**Female.** Body color: body mostly dark except for some sternites which may be pale. Antenna color: scape, pedicel, and flagellum dark. Coxae color (pro-, meso-, metacoxa): dark, dark, dark. Femora color (pro-, meso-, metafemur): pale, pale, pale. Tibiae color (pro-, meso-, metatibia): pale, pale, mostly pale but with posterior 0.2 or less dark. Tegula and humeral complex color: tegula pale, humeral complex dark. Pterostigma color: dark. Fore wing veins color: mostly dark (a few veins may be unpigmented). Antenna length/body length: antenna about as long as body (head to apex of metasoma); if slightly shorter, at least extending beyond anterior 0.7 metasoma length. Body in lateral view: not distinctly flattened dorso–ventrally. Body length (head to apex of metasoma): 4.0 mm or more. Fore wing length: 4.0 mm or more. Ocular–ocellar line/posterior ocellus diameter: 1.7–1.9. Interocellar distance/posterior ocellus diameter: 1.7–1.9. Antennal flagellomerus 2 length/width: 2.3–2.5, 2.6–2.8, rarely 2.9–3.1. Antennal flagellomerus 14 length/width: 2.6–2.9 or 3.0 or more. Length of flagellomerus 2/length of flagellomerus 14: 1.1–1.3, rarely 1.4–1.6. Tarsal claws: pectinate. Metafemur length/width: 2.8–2.9. Metatibia inner spur length/metabasitarsus length: 0.6–0.7. Anteromesoscutum: mostly with deep, dense punctures (separated by less than 2.0 × its maximum diameter). Mesoscutellar disc: with punctures near margins, central part mostly smooth. Number of pits in scutoscutellar sulcus: 7 or 8, rarely 9 or 10. Maximum height of mesoscutellum lunules/maximum height of lateral face of mesoscutellum: 0.4–0.5. Propodeum areola: completely defined by carinae, including transverse carina extending to spiracle. Propodeum background sculpture: mostly sculptured. Mediotergite 1 length/width at posterior margin: 2.0–2.2. Mediotergite 1 shape: more or less parallel–sided. Mediotergite 1 sculpture: mostly smooth. Mediotergite 2 width at posterior margin/length: 4.4–4.7. Mediotergite 2 sculpture: mostly smooth. Outer margin of hypopygium: with a wide, medially folded, transparent, semi–desclerotized area; usually with 4 or more pleats. Ovipositor thickness: anterior width 3.0–5.0 × posterior width (beyond ovipositor constriction). Ovipositor sheaths length/metatibial length: 0.8–0.9, rarely 1.0–1.1. Length of fore wing veins r/2RS: 1.7–1.9. Length of fore wing veins 2RS/2M: 1.7–1.8. Length of fore wing veins 2M/(RS+M)b: 0.5–0.6. Pterostigma length/width: 3.6 or more. Point of insertion of vein r in pterostigma: clearly beyond half way point length of pterostigma. Angle of vein r with fore wing anterior margin: clearly outwards, inclined towards fore wing apex. Shape of junction of veins r and 2RS in fore wing: distinctly but not strongly angled.

**Male.** Metasomal terga may be darker than in females, otherwise specimens are similar.

#### Molecular data.

Sequences in BOLD: 22, barcode compliant sequences: 18.

#### Biology/ecology.

Solitary (Fig. 266). Host: Hesperiidae, *Milanion marciana*.

#### Distribution.

Costa Rica, ACG.

#### Etymology.

We dedicate this species to Calixto Moraga in recognition of his diligent efforts for the ACG Programa de Parataxónomos and Estación Biológica Pitilla of ACG.

### Apanteles carloscastilloi

Fernández-Triana sp. n.

http://zoobank.org/3504EC92-8C9C-4B9A-943C-B2DD464DEF85

http://species-id.net/wiki/Apanteles_carloscastilloi

Figs 5 , 211

Apanteles Rodriguez12. Smith et al. (2008). Interim name used by the authors.

#### Type locality.

COSTA RICA, Alajuela, ACG, Sector San Cristobal, Rio Blanco Abajo, 500m, 10.90037, -85.37254.

#### Holotype.

♀ in CNC. Specimen labels: 1. Costa Rica: Alajuela, ACG, Sector San Cristobal, Rio Blanco Abajo, 04.iv.2002, 500m, 10.90037, -85.37254, DHJPAR0002960.

#### Paratypes.

40 ♀, 6 ♂ (BMNH, CNC, INBIO, INHS, NMNH). COSTA RICA, ACG database codes: See Appendix 2 for detailed label data.

#### Description.

**Female.** Body color: body mostly dark except for some sternites which may be pale. Antenna color: scape, pedicel, and flagellum dark. Coxae color (pro-, meso-, metacoxa): dark, dark, dark. Femora color (pro-, meso-, metafemur): anteriorly dark/posteriorly pale, dark, dark. Tibiae color (pro-, meso-, metatibia): pale, pale, anteriorly pale/posteriorly dark. Tegula and humeral complex color: tegula pale, humeral complex half pale/half dark. Pterostigma color: mostly pale and/or transparent, with thin dark borders. Fore wing veins color: partially pigmented (a few veins may be dark but most are pale). Antenna length/body length: antenna about as long as body (head to apex of metasoma); if slightly shorter, at least extending beyond anterior 0.7 metasoma length. Body in lateral view: not distinctly flattened dorso–ventrally. Body length (head to apex of metasoma): 2.9–3.0 mm, 3.1–3.2 mm, rarely 2.0 mm or less or 3.3–3.4 mm. Fore wing length: 2.9–3.0 mm, 3.1–3.2 mm, rarely 2.0 mm or less or 3.3–3.4 mm. Ocular–ocellar line/posterior ocellus diameter: 2.3–2.5, rarely 2.0–2.2. Interocellar distance/posterior ocellus diameter: 1.7–1.9. Antennal flagellomerus 2 length/width: 2.6–2.8. Antennal flagellomerus 14 length/width: 1.4–1.6. Length of flagellomerus 2/length of flagellomerus 14: 2.0–2.2. Tarsal claws: with single basal spine–like seta. Metafemur length/width: 3.2–3.3. Metatibia inner spur length/metabasitarsus length: 0.4–0.5. Anteromesoscutum: mostly with deep, dense punctures (separated by less than 2.0 × its maximum diameter). Mesoscutellar disc: with punctures near margins, central part mostly smooth. Number of pits in scutoscutellar sulcus: 5 or 6 or 7 or 8. Maximum height of mesoscutellum lunules/maximum height of lateral face of mesoscutellum: 0.4–0.5. Propodeum areola: completely defined by carinae, including transverse carina extending to spiracle. Propodeum background sculpture: mostly sculptured. Mediotergite 1 length/width at posterior margin: 2.0–2.2. Mediotergite 1 shape: mostly parallel–sided for 0.5–0.7 of its length, then narrowing posteriorly so mediotergite anterior width >1.1 × posterior width. Mediotergite 1 sculpture: mostly sculptured, excavated area centrally with transverse striation inside and/or a polished knob centrally on posterior margin of mediotergite. Mediotergite 2 width at posterior margin/length: 2.8–3.1. Mediotergite 2 sculpture: mostly smooth. Outer margin of hypopygium: with a wide, medially folded, transparent, semi–desclerotized area; usually with 4 or more pleats. Ovipositor thickness: about same width throughout its length. Ovipositor sheaths length/metatibial length: 1.6–1.7, rarely 1.2–1.3. Length of fore wing veins r/2RS: 1.7–1.9. Length of fore wing veins 2RS/2M: 0.9–1.0. Length of fore wing veins 2M/(RS+M)b: 0.9–1.0. Pterostigma length/width: 3.6 or more. Point of insertion of vein r in pterostigma: clearly beyond half way point length of pterostigma. Angle of vein r with fore wing anterior margin: clearly outwards, inclined towards fore wing apex. Shape of junction of veins r and 2RS in fore wing: distinctly but not strongly angled.

**Male.** As in female but with darker legs and smoother mediotergite 1.

#### Molecular data.

Sequences in BOLD: 18, barcode compliant sequences: 16.

#### Biology/ecology.

Gregarious (Fig. 211). Host: Elachistidae, *Stenoma completella*.

#### Distribution.

Costa Rica, ACG.

#### Etymology.

We dedicate this species to Carlos Castillo in recognition of his diligent efforts in the ACG Programa de Seguridad.

### Apanteles carlosguadamuzi

Fernández-Triana sp. n.

http://zoobank.org/D916782A-17F2-4E49-845F-D9C452700510

http://species-id.net/wiki/Apanteles_carlosguadamuzi

Figs 90 , 269

#### Type locality.

COSTA RICA, Alajuela, ACG, Sector San Cristobal, Rio Blanco Abajo, 500m, 10.90037, -85.37254.

#### Holotype.

♀ in CNC. Specimen labels: 1. DHJPAR0026391. 2. San Gerardo, Rio Blanco Abajo, Date: 26 Oct-1 Nov 2007.

#### Paratypes.

1#F, 3#M (CNC, NMNH). COSTA RICA, ACG database codes: DHJPAR0025069, DHJPAR0025349, DHJPAR0026047, DHJPAR0026632.

#### Description.

**Female.** Body color: head dark, mesosoma dark with parts of axillar complex pale, metasoma with some mediotergites, most laterotergites, sternites, and/or hypopygium pale. Antenna color: scape and/or pedicel pale, flagellum dark. Coxae color (pro-, meso-, metacoxa): pale, pale, pale. Femora color (pro-, meso-, metafemur): pale, pale, mostly pale but posterior 0.2 or less dark. Tibiae color (pro-, meso-, metatibia): pale, pale, anteriorly pale/posteriorly dark. Tegula and humeral complex color: both pale. Pterostigma color: dark. Fore wing veins color: mostly dark (a few veins may be unpigmented). Antenna length/body length: antenna about as long as body (head to apex of metasoma); if slightly shorter, at least extending beyond anterior 0.7 metasoma length. Body in lateral view: not distinctly flattened dorso–ventrally. Body length (head to apex of metasoma): 3.1–3.2 mm or 3.3–3.4 mm. Fore wing length: 3.3–3.4 mm or 3.5–3.6 mm. Ocular–ocellar line/posterior ocellus diameter: 2.0–2.2. Interocellar distance/posterior ocellus diameter: 2.0–2.2. Antennal flagellomerus 2 length/width: 2.6–2.8. Antennal flagellomerus 14 length/width: 1.4–1.6. Length of flagellomerus 2/length of flagellomerus 14: 2.3–2.5. Tarsal claws: simple. Metafemur length/width: 3.2–3.3. Metatibia inner spur length/metabasitarsus length: 0.4–0.5. Anteromesoscutum: mostly with deep, dense punctures (separated by less than 2.0 × its maximum diameter). Mesoscutellar disc: mostly smooth. Number of pits in scutoscutellar sulcus: 7 or 8. Maximum height of mesoscutellum lunules/maximum height of lateral face of mesoscutellum: 0.2–0.3. Propodeum areola: completely defined by carinae, including transverse carina extending to spiracle. Propodeum background sculpture: mostly sculptured. Mediotergite 1 length/width at posterior margin: 2.9–3.1. Mediotergite 1 shape: more or less parallel–sided. Mediotergite 1 sculpture: with some sculpture near lateral margins and/or posterior 0.2–0.4 of mediotergite. Mediotergite 2 width at posterior margin/length: 3.6–3.9. Mediotergite 2 sculpture: mostly smooth. Outer margin of hypopygium: with a wide, medially folded, transparent, semi–desclerotized area; usually with 4 or more pleats. Ovipositor thickness: about same width throughout its length. Ovipositor sheaths length/metatibial length: 0.6–0.7. Length of fore wing veins r/2RS: 2.0–2.2. Length of fore wing veins 2RS/2M: 1.1–1.3. Length of fore wing veins 2M/(RS+M)b: 0.7–0.8. Pterostigma length/width: 2.6–3.0. Point of insertion of vein r in pterostigma: clearly beyond half way point length of pterostigma. Angle of vein r with fore wing anterior margin: clearly outwards, inclined towards fore wing apex. Shape of junction of veins r and 2RS in fore wing: distinctly but not strongly angled.

**Male.** As in female, except for darker metasomal terga.

#### Molecular data.

Sequences in BOLD: 14, barcode compliant sequences: 14.

#### Biology/ecology.

Solitary (Fig. 269). Host: Choreutidae, *Zodia* Janzen02; Crambidae, *Syllepte nitidalis* DHJ01, *Syllepte* Janzen03.

#### Distribution.

Costa Rica, ACG.

#### Etymology.

We dedicate this species to Carlos Guadamuz in recognition of his diligent efforts for the ACG Programa de Mantenimiento.

### Apanteles carlosrodriguezi

Fernández-Triana sp. n.

http://zoobank.org/51CD1517-B560-4E1F-B793-D47FBD8A85BB

http://species-id.net/wiki/Apanteles_carlosrodriguezi

Figs 96 , 330

Apanteles Rodriguez160 (Smith et al. 2006). Interim name provided by the authors.

#### Type locality.

COSTA RICA, Alajuela, ACG, Sector Pitilla, Sendero Cuestona, 640m, 10.99455, -85.41461.

#### Holotype.

♀ in CNC. Specimen labels: 1. DHJPAR0035504. 2. COSTA RICA, Guanacaste, ACG, Sector Pitilla, Sendero Cuestona Site 27.iii.2009, 10.99455°N, -85.41461°W, 640m, DHJPAR0035504. 3. Voucher: D.H.Janzen & W.Hallwachs, DB: http://janzen.sas.upenn.edu, Area de Conservación Guanacaste, COSTA RICA, 09-SRNP-31005.

#### Paratypes.

1 ♀, 1 ♂ (CNC). COSTA RICA, ACG database codes: DHJPAR0035342, DHJPAR0035500.

#### Description.

**Female.** Body color: body mostly dark except for some sternites which may be pale. Antenna color: scape, pedicel, and flagellum dark. Coxae color (pro-, meso-, metacoxa): dark, dark, dark. Femora color (pro-, meso-, metafemur): pale, dark, dark. Tibiae color (pro-, meso-, metatibia): pale, pale, mostly dark but anterior 0.2 or less pale. Tegula and humeral complex color: both dark. Pterostigma color: dark with pale spot at base. Fore wing veins color: partially pigmented (a few veins may be dark but most are pale). Antenna length/body length: antenna about as long as body (head to apex of metasoma); if slightly shorter, at least extending beyond anterior 0.7 metasoma length. Body in lateral view: not distinctly flattened dorso–ventrally. Body length (head to apex of metasoma): 2.0 mm or less. Fore wing length: 2.1–2.2 mm. Ocular–ocellar line/posterior ocellus diameter: 2.6 or more. Interocellar distance/posterior ocellus diameter: 1.7–1.9. Antennal flagellomerus 2 length/width: 2.9–3.1. Antennal flagellomerus 14 length/width: 1.7–1.9. Length of flagellomerus 2/length of flagellomerus 14: 2.0–2.2. Tarsal claws: simple. Metafemur length/width: 3.2–3.3. Metatibia inner spur length/metabasitarsus length: 0.4–0.5. Anteromesoscutum: mostly with shallow, dense punctures (separated by less than 2.0 × its maximum diameter). Mesoscutellar disc: mostly smooth. Number of pits in scutoscutellar sulcus: 7 or 8. Maximum height of mesoscutellum lunules/maximum height of lateral face of mesoscutellum: 0.4–0.5. Propodeum areola: completely defined by carinae, including transverse carina extending to spiracle. Propodeum background sculpture: partly sculptured, especially on anterior 0.5. Mediotergite 1 length/width at posterior margin: 3.2–3.4. Mediotergite 1 shape: mostly parallel–sided for 0.5–0.7 of its length, then narrowing posteriorly so mediotergite anterior width >1.1 × posterior width. Mediotergite 1 sculpture: mostly sculptured, excavated area centrally with transverse striation inside and/or a polished knob centrally on posterior margin of mediotergite. Mediotergite 2 width at posterior margin/length: 4.4–4.7. Mediotergite 2 sculpture: mostly smooth. Outer margin of hypopygium: with a medially folded, transparent, semi–desclerotized area; with 0–3 pleats visible. Ovipositor thickness: about same width throughout its length (?). Ovipositor sheaths length/metatibial length: 0.4–0.5. Length of fore wing veins r/2RS: 1.0 or less. Length of fore wing veins 2RS/2M: 1.4–1.6. Length of fore wing veins 2M/(RS+M)b: 0.7–0.8. Pterostigma length/width: 3.1–3.5. Point of insertion of vein r in pterostigma: about half way point length of pterostigma. Angle of vein r with fore wing anterior margin: more or less perpendicular to fore wing margin. Shape of junction of veins r and 2RS in fore wing: evenly curved.

**Male.** As in female but with slender mediotergite 1 and pterostigma mostly transparent.

#### Molecular data.

Sequences in BOLD: 6, barcode compliant sequences: 6.

#### Biology/ecology.

Solitary (Fig. 330). Hosts: Elachistidae, two species of *Antaeotricha* and probably two species of Choreutidae (to be confirmed eventually).

#### Distribution.

Costa Rica, ACG.

#### Etymology.

We dedicate this species to Carlos Rodríguez in recognition of his efforts for the ACG Programa de Ecoturismo.

### Apanteles carlosviquezi

Fernández-Triana sp. n.

http://zoobank.org/390076C2-52FD-4DD3-BC57-BD0AF6EA27F6

http://species-id.net/wiki/Apanteles_carlosviquezi

Figs 173 , 307

Apanteles Rodriguez25 (Smith et al. 2006). Interim name provided by the authors.

#### Type locality.

COSTA RICA, Guanacaste, ACG, Sector Pitilla, Loaiciga, 445m, 11.01983, -85.41342.

#### Holotype.

♀ in CNC. Specimen labels: 1. DHJPAR0002716. 2. COSTA RICA, Guanacaste, ACG, Sector Pitilla, Loaiciga, 15.iii.2004, 445m, 11.01983, -85.41342, 04-SRNP-31273.

#### Paratypes.

37 ♀, 8 ♂ (BMNH, CNC, INBIO, INHS, NMNH). COSTA RICA, ACG database codes: DHJPAR0001596, DHJPAR0002716, DHJPAR0002907.

#### Description.

**Female.** Metatibia color (outer face): entirely or mostly (>0.7 metatibia length) dark brown to black, with yellow to white coloration usually restricted to anterior 0.2 or less. Fore wing veins color: veins C+Sc+R and R1 with brown coloration restricted narrowly to borders, interior area of those veins and pterostigma (and sometimes veins r, 2RS and 2M) transparent or white; other veins mostly transparent. Antenna length/body length: antenna about as long as body (head to apex of metasoma); if slightly shorter, at least extending beyond anterior 0.7 metasoma length. Body length (head to apex of metasoma): 2.1–2.2 mm or 2.3–2.4 mm. Fore wing length: 2.3–2.4 mm or 2.5–2.6 mm. Metafemur length/width: 3.0–3.1. Mediotergite 1 length/width at posterior margin: 2.3–2.4. Mediotergite 1 maximum width/width at posterior margin: 1.4–1.5. Ovipositor sheaths length/metafemur length: 1.0. Ovipositor sheaths length/metatibia length: 0.8.

#### Molecular data.

Sequences in BOLD: 4, barcode compliant sequences: 4.

#### Biology/ecology.

Gregarious (Fig. 307). Host: Hesperiidae, *Telemiades oiclus*.

#### Distribution.

Costa Rica, ACG.

#### Etymology.

We dedicate this species to Carlos Viquez in recognition of his diligent efforts for administration and spider curatorial taxonomy for INBio, Costa Rica’s Instituto Nacional de Biodiversidad.

### Apanteles carloszunigai

Fernández-Triana sp. n.

http://zoobank.org/3C7EF174-68BF-4E7D-8293-538D965316EE

http://species-id.net/wiki/Apanteles_carloszunigai

Fig. 99

#### Type locality.

COSTA RICA, Alajuela, ACG, Sector San Cristobal, Potrero Argentina, 520m, 10.89021, -85.38803.

#### Holotype.

♀ in CNC. Specimen labels: 1. San Gerardo, Sitio Argentina, 28 Jul-3 Aug/2007. 2. DHJPAR0027484.

#### Description.

**Female.** Body color: body mostly dark except for some sternites which may be pale. Antenna color: scape and/or pedicel pale, flagellum dark. Coxae color (pro-, meso-, metacoxa): pale, pale, pale. Femora color (pro-, meso-, metafemur): pale, pale, mostly pale but posterior 0.2 or less dark. Tibiae color (pro-, meso-, metatibia): pale, pale, mostly pale but with posterior 0.2 or less dark. Tegula and humeral complex color: both dark. Pterostigma color: dark. Fore wing veins color: mostly dark (a few veins may be unpigmented). Antenna length/body length: antenna about as long as body (head to apex of metasoma); if slightly shorter, at least extending beyond anterior 0.7 metasoma length. Body in lateral view: not distinctly flattened dorso–ventrally. Body length (head to apex of metasoma): 2.3–2.4 mm. Fore wing length: 2.7–2.8 mm. Ocular–ocellar line/posterior ocellus diameter: 2.0–2.2. Interocellar distance/posterior ocellus diameter: 1.7–1.9. Antennal flagellomerus 2 length/width: 2.9–3.1. Antennal flagellomerus 14 length/width: 1.7–1.9. Length of flagellomerus 2/length of flagellomerus 14: 2.0–2.2. Tarsal claws: simple. Metafemur length/width: 3.0–3.1. Metatibia inner spur length/metabasitarsus length: 0.4–0.5. Anteromesoscutum: mostly with deep, dense punctures (separated by less than 2.0 × its maximum diameter). Mesoscutellar disc: mostly punctured. Number of pits in scutoscutellar sulcus: 5 or 6. Maximum height of mesoscutellum lunules/maximum height of lateral face of mesoscutellum: 0.4–0.5. Propodeum areola: completely defined by carinae, including transverse carina extending to spiracle. Propodeum background sculpture: mostly sculptured. Mediotergite 1 length/width at posterior margin: 3.2–3.4. Mediotergite 1 shape: mostly parallel–sided for 0.5–0.7 of its length, then narrowing posteriorly so mediotergite anterior width >1.1 × posterior width. Mediotergite 1 sculpture: with some sculpture near lateral margins and/or posterior 0.2–0.4 of mediotergite. Mediotergite 2 width at posterior margin/length: 4.0–4.3. Mediotergite 2 sculpture: mostly smooth. Outer margin of hypopygium: with a medially folded, transparent, semi–desclerotized area; with 0–3 pleats visible. Ovipositor thickness: anterior width at most 2.0 × posterior width (beyond ovipositor constriction). Ovipositor sheaths length/metatibial length: 0.4–0.5. Length of fore wing veins r/2RS: 1.4–1.6. Length of fore wing veins 2RS/2M: 1.1–1.3. Length of fore wing veins 2M/(RS+M)b: 0.9–1.0. Pterostigma length/width: 3.1–3.5. Point of insertion of vein r in pterostigma: clearly beyond half way point length of pterostigma. Angle of vein r with fore wing anterior margin: more or less perpendicular to fore wing margin. Shape of junction of veins r and 2RS in fore wing: strongly angulated, sometimes with a knob.

**Male.** Unknown.

#### Molecular data.

Sequences in BOLD: 3, barcode compliant sequences: 3.

#### Biology/ecology.

Malaise-trapped.

#### Distribution.

Costa Rica, ACG.

#### Etymology.

We dedicate this species to Carlos Zúñiga in recognition of his diligent efforts for the ACG Programa de Sectores.

### Apanteles carolinacanoae

Fernández-Triana sp. n.

http://zoobank.org/8A4E4AAC-D056-4A54-AE5A-03DC4377DEF0

http://species-id.net/wiki/Apanteles_carolinacanoae

Figs 52 , 245

Apanteles Rodriguez04 (Smith et al. 2006). Interim name provided by the authors.

#### Type locality.

COSTA RICA, Guanacaste, ACG, Sector Horizontes, Sitio La Dama, 105m, 10.78626, -85.55835.

#### Holotype.

♀ in CNC. Specimen labels: 1. COSTA RICA, Guanacaste, ACG, Horizontes: Esperanza, 09/21/1994. 2. 94-SRNP-7527, *Pyrgus adepta*, *Sida acuta*. 3. DHJPAR0001561.

#### Paratypes.

29 ♀, 11 ♂ (BMNH, CNC, INBIO, INHS, NMNH). COSTA RICA: ACG database codes: 92-SRNP-4401, 92-SRNP-5190, 93-SRNP-3572, 93-SRNP-3573, 93-SRNP-3574, 93-SRNP-3681, 93-SRNP-3735, 93-SRNP-4176, 93-SRNP-6388, 93-SRNP-6748, 93-SRNP-7311, 95-SRNP-8417, 95-SRNP-8471, 97-SRNP-3898, 04-SRNP-46868, 04-SRNP-50139, 05-SRNP-4197, 06-SRNP-311, 09-SRNP-72610, 10-SRNP-67708, 11-SRNP-69640.

#### Description.

**Female.** Body color: head and mesosoma mostly dark, metasoma with some tergites and/or most of sternites pale. Antenna color: scape and/or pedicel pale, flagellum dark. Coxae color (pro-, meso-, metacoxa): pale, pale, pale. Femora color (pro-, meso-, metafemur): pale, pale, pale. Tibiae color (pro-, meso-, metatibia): pale, pale, pale. Tegula and humeral complex color: both pale. Pterostigma color: mostly pale and/or transparent, with thin dark borders. Fore wing veins color: mostly white or entirely transparent. Antenna length/body length: antenna shorter than body (head to apex of metasoma), not extending beyond anterior 0.7 metasoma length. Body in lateral view: not distinctly flattened dorso–ventrally. Body length (head to apex of metasoma): 2.3–2.4 mm or 2.5–2.6 mm. Fore wing length: 2.5–2.6 mm or 2.7–2.8 mm. Ocular–ocellar line/posterior ocellus diameter: 2.3–2.5. Interocellar distance/posterior ocellus diameter: 1.4–1.6. Antennal flagellomerus 2 length/width: 2.3–2.5. Antennal flagellomerus 14 length/width: 1.4–1.6. Length of flagellomerus 2/length of flagellomerus 14: 2.0–2.2. Tarsal claws: with single basal spine–like seta. Metafemur length/width: 3.0–3.1. Metatibia inner spur length/metabasitarsus length: 0.4–0.5. Anteromesoscutum: mostly with shallow, dense punctures (separated by less than 2.0 × its maximum diameter). Mesoscutellar disc: mostly smooth. Number of pits in scutoscutellar sulcus: 9 or 10. Maximum height of mesoscutellum lunules/maximum height of lateral face of mesoscutellum: 0.4–0.5. Propodeum areola: completely defined by carinae, but only partial or absent transverse carina. Propodeum background sculpture: mostly sculptured. Mediotergite 1 length/width at posterior margin: 2.3–2.5. Mediotergite 1 shape: more or less parallel–sided. Mediotergite 1 sculpture: mostly sculptured, excavated area centrally with transverse striation inside and/or a polished knob centrally on posterior margin of mediotergite. Mediotergite 2 width at posterior margin/length: 4.0–4.3. Mediotergite 2 sculpture: mostly smooth. Outer margin of hypopygium: inflexible (without a folded, transparent, semi–desclerotized area); with no pleats visible. Ovipositor thickness: anterior width 3.0–5.0 × posterior width (beyond ovipositor constriction). Ovipositor sheaths length/metatibial length: 1.0–1.1. Length of fore wing veins r/2RS: 1.4–1.6. Length of fore wing veins 2RS/2M: 1.4–1.6. Length of fore wing veins 2M/(RS+M)b: 0.7–0.8. Pterostigma length/width: 3.1–3.5. Point of insertion of vein r in pterostigma: clearly beyond half way point length of pterostigma. Angle of vein r with fore wing anterior margin: more or less perpendicular to fore wing margin. Shape of junction of veins r and 2RS in fore wing: distinctly but not strongly angled.

**Male.** Darker specimens, with narrower mediotergites 1 and 2.

#### Molecular data.

Sequences in BOLD: 15, barcode compliant sequences: 9.

#### Biology/ecology.

Gregarious (Fig. 245). Hosts: Hesperiidae, *Pyrgus adepta*, *Pyrgus oileus*.

#### Distribution.

Costa Rica, ACG.

#### Etymology.

We dedicate this species to Carolina Cano in recognition of her diligent efforts for the ACG Programa de Parataxónomos and Estación Biológica San Gerardo of ACG.

### Apanteles carpatus

(Say, 1836)

http://species-id.net/wiki/Apanteles_carpatus

Microgaster carpata Say, 1836: 263.
Apanteles carpatus (Say, 1836). Transferred by Riley 1881: 19.
Urogaster solitarius Ashmead, 1900: 287. See *Apanteles piceoventris* Muesebeck below.
Protapanteles hawaiiensis Ashmead, 1901: 362. Synonymized by Muesebeck and Walkley 1951: 125.
Urogaster fuscicornis Cameron, 1910: 479. Synonymized by Wilkinson 1932: 313.
Apanteles igae Watanabe, 1932: 97. Synonymized by Watanabe 1933: 97.
Apanteles piceoventris Muesebeck, 1921: 515. Replacement name for *Urogaster solitarius* Ashmead, 1900. Synonymized by Muesebeck 1958: 431.
Apanteles sarcitorius Telenga, 1955: 55. Synonymized by Papp 1980: 269.
Apanteles ultericus Telenga, 1955: 57. Synonymized by Papp 1980: 269.

#### Type locality.

UNITED STATES, Indiana, locality not specified.

#### Holotype.

♀, Destroyed.

#### Material Examined.

28 ♀, 7 ♂ (CNC), CANADA: ON, Biscotasing, Ottawa, Vineland; NB, York County; BC, Aldergrove, Vancouver; PUERTO RICO: Cueva Tuna; UKRAINE: Kiev; UNITED STATES: NC, Bertie County, near Cahaba.

#### Description.

**Female.** Body color: body mostly dark except for some sternites which may be pale. Antenna color: scape and/or pedicel dark, flagellum pale. Coxae color (pro-, meso-, metacoxa): pale, pale, dark. Femora color (pro-, meso-, metafemur): pale, pale, pale. Tibiae color (pro-, meso-, metatibia): pale, pale, pale, rarely pale, pale, mostly pale but with posterior 0.2 or less dark. Tegula and humeral complex color: both pale. Pterostigma color: dark with pale spot at base. Fore wing veins color: partially pigmented (a few veins may be dark but most are pale). Antenna length/body length: antenna shorter than body (head to apex of metasoma), not extending beyond anterior 0.7 metasoma length. Body in lateral view: not distinctly flattened dorso–ventrally. Body length (head to apex of metasoma): 2.7–2.8 mm, 2.9–3.0 mm or 3.1–3.2 mm. Fore wing length: 2.5–2.6 mm, 2.7–2.8 mm or 2.9–3.0 mm. Ocular–ocellar line/posterior ocellus diameter: 2.0–2.2. Interocellar distance/posterior ocellus diameter: 1.7–1.9. Antennal flagellomerus 2 length/width: 2.0–2.2. Antennal flagellomerus 14 length/width: 1.0 or less or 1.1–1.3. Length of flagellomerus 2/length of flagellomerus 14: 2.0–2.2. Tarsal claws: with single basal spine–like seta. Metafemur length/width: 2.8–2.9. Metatibia inner spur length/metabasitarsus length: 0.6–0.7. Anteromesoscutum: mostly with deep, dense punctures (separated by less than 2.0 × its maximum diameter). Mesoscutellar disc: with punctures near margins, central part mostly smooth. Number of pits in scutoscutellar sulcus: 9 or 10. Maximum height of mesoscutellum lunules/maximum height of lateral face of mesoscutellum: 0.2–0.3. Propodeum areola: completely defined by carinae, including transverse carina extending to spiracle. Propodeum background sculpture: partly sculptured, especially on anterior 0.5. Mediotergite 1 length/width at posterior margin: 1.4–1.6. Mediotergite 1 shape: clearly widening towards posterior margin. Mediotergite 1 sculpture: mostly sculptured, excavated area centrally with transverse striation inside and/or a polished knob centrally on posterior margin of mediotergite. Mediotergite 2 width at posterior margin/length: 3.2–3.5. Mediotergite 2 sculpture: more or less fully sculptured, with longitudinal striation. Outer margin of hypopygium: with a wide, medially folded, transparent, semi–desclerotized area; usually with 4 or more pleats. Ovipositor thickness: about same width throughout its length. Ovipositor sheaths length/metatibial length: 1.0–1.1. Length of fore wing veins r/2RS: 1.7–1.9. Length of fore wing veins 2RS/2M: 0.9–1.0. Length of fore wing veins 2M/(RS+M)b: 0.9–1.0. Pterostigma length/width: 2.1–2.5. Point of insertion of vein r in pterostigma: about half way point length of pterostigma. Angle of vein r with fore wing anterior margin: more or less perpendicular to fore wing margin. Shape of junction of veins r and 2RS in fore wing: distinctly but not strongly angled.

**Male.** Similar to female, except for shape of mediotergite 1 which is more rectangular, and coloration of meso and metafemur which tends to be darker in some specimens.

#### Molecular data.

Sequences in BOLD: 16, barcode compliant sequences: 15, haplotypes: 2.

#### Biology/ecology.

Gregarious. Mostly recorded from Lepidoptera species on stored products – its cosmopolitan distribution is likely due to human transfer from an unknown source. Hosts: Gelechiidae, Lasiocampidae, Lecithoceridae, Lymantriidae, Pyralidae, Thaumetopoeidae, Tineidae, Tortricidae, Zygaenidae. The correctness of some of these host records is questionable because it is unlikely that a single species has such a wide host range.

#### Distribution.

Cosmopolitan, this species has been recorded from 50 countries in all continents but there is no suggestion that it occurs in ACG.

#### Comments.

The geographical coverage of the barcoded specimens includes Canada and New Zealand, but all sequences are almost identical. The only exceptions are some extralimital specimens (British Columbia, Canada) which seem to represent a different species based on body color and the barcode of one specimen. Because those specimens are not from Mesoamerica, they will be dealt with elsewhere.

### Apanteles christianzunigai

Fernández-Triana sp. n.

http://zoobank.org/7E1ED712-0B24-443C-9663-683070D45C9B

http://species-id.net/wiki/Apanteles_christianzunigai

Figs 105 , 277

Apanteles Rodriguez86 (Smith et al. 2006). Interim name provided by the authors.

#### Type locality.

COSTA RICA, Guanacaste, ACG, Sector Pitilla, Sendero Trichoptera, 655m, 10.98571, -85.41869.

#### Holotype.

♀ in CNC. Specimen labels: 1. Voucher: D.H.Janzen & W.Hallwachs, DB: http://janzen.sas.upenn.edu, Area de Conservación Guanacaste, COSTA RICA, 09-SRNP-32760. 2. DHJPAR0039745.

#### Paratypes.

3 ♀, 1 ♂ (CNC, NMNH). COSTA RICA, ACG database codes: DHJPAR00215, DHJPAR0038242, DHJPAR0038323, DHJPAR0038349.

#### Description.

**Female.** Body color: body mostly dark except for some sternites which may be pale. Antenna color: scape, pedicel, and flagellum dark. Coxae color (pro-, meso-, metacoxa): dark, dark, dark. Femora color (pro-, meso-, metafemur): pale, dark, dark. Tibiae color (pro-, meso-, metatibia): pale, pale, anteriorly pale/posteriorly dark. Tegula and humeral complex color: tegula pale, humeral complex dark. Pterostigma color: dark. Fore wing veins color: mostly dark (a few veins may be unpigmented). Antenna length/body length: antenna about as long as body (head to apex of metasoma); if slightly shorter, at least extending beyond anterior 0.7 metasoma length. Body in lateral view: not distinctly flattened dorso–ventrally. Body length (head to apex of metasoma): 3.1–3.2 mm, rarely 3.3–3.4 mm. Fore wing length: 2.9–3.0 mm. Ocular–ocellar line/posterior ocellus diameter: 1.7–1.9. Interocellar distance/posterior ocellus diameter: 1.4–1.6. Antennal flagellomerus 2 length/width: 3.2 or more. Antennal flagellomerus 14 length/width: 1.7–1.9. Length of flagellomerus 2/length of flagellomerus 14: 2.3–2.5. Tarsal claws: simple. Metafemur length/width: 3.2–3.3. Metatibia inner spur length/metabasitarsus length: 0.4–0.5. Anteromesoscutum: mostly with deep, dense punctures (separated by less than 2.0 × its maximum diameter). Mesoscutellar disc: with punctures near margins, central part mostly smooth. Number of pits in scutoscutellar sulcus: 7 or 8. Maximum height of mesoscutellum lunules/maximum height of lateral face of mesoscutellum: 0.4–0.5. Propodeum areola: completely defined by carinae, including transverse carina extending to spiracle. Propodeum background sculpture: mostly sculptured. Mediotergite 1 length/width at posterior margin: 3.2–3.4. Mediotergite 1 shape: more or less parallel–sided. Mediotergite 1 sculpture: mostly sculptured, excavated area centrally with transverse striation inside and/or a polished knob centrally on posterior margin of mediotergite. Mediotergite 2 width at posterior margin/length: 2.0–2.3. Mediotergite 2 sculpture: with some sculpture, mostly near posterior margin. Outer margin of hypopygium: with a wide, medially folded, transparent, semi–desclerotized area; usually with 4 or more pleats. Ovipositor thickness: anterior width at most 2.0 × posterior width (beyond ovipositor constriction). Ovipositor sheaths length/metatibial length: 0.8–0.9 or 1.0–1.1. Length of fore wing veins r/2RS: 2.0–2.2. Length of fore wing veins 2RS/2M: 1.1–1.3. Length of fore wing veins 2M/(RS+M)b: 0.7–0.8. Pterostigma length/width: 3.1–3.5. Point of insertion of vein r in pterostigma: about half way point length of pterostigma. Angle of vein r with fore wing anterior margin: more or less perpendicular to fore wing margin. Shape of junction of veins r and 2RS in fore wing: distinctly but not strongly angled.

**Male.** As in female.

#### Molecular data.

Sequences in BOLD: 12, barcode compliant sequences: 12.

#### Biology/ecology.

Solitary (Fig. 277). Hosts: Elachistidae, five species of *Stenoma*.

#### Distribution.

Costa Rica, ACG.

#### Comments.

This species is characterized by the combination of broadly rectangular mediotergite 2 (its width at posterior margin 2.2 × its length), ovipositor sheaths shorter than metatibia, and ovipositor relatively thin, with basal width less than 2.0 × its apical width after constriction.

#### Etymology.

We dedicate this species to Christian Zúñiga in recognition of his diligent efforts for the ACG Programa de Ecoturismo.

### Apanteles cinthiabarrantesae

Fernández-Triana sp. n.

http://zoobank.org/C994D2D7-C0FB-4997-938C-E6C5078C103D

http://species-id.net/wiki/Apanteles_cinthiabarrantesae

Figs 91 , 270

Apanteles Rodriguez11. Smith et al. (2008). Interim name provided by the authors.

#### Type locality.

COSTA RICA, Alajuela, ACG, Sector Rincon Rain Forest, Sendero Tucan, 410m, 10.90424, -85.2712.

#### Holotype.

♀ in CNC. Specimen labels: 1. Costa Rica: Alajuela, ACG, Sector Rincon Rain Forest, Sendero Tucan, 19.vii.2004, 410m, 10.90424, -85.2712, 04-SRNP-41852.

#### Paratypes.

34 ♀, 7 ♂ (BMNH, CNC, INBIO, INHS, NMNH). COSTA RICA, ACG database codes: See Appendix 2 for detailed label data.

#### Description.

**Female.** Body color: head dark, mesosoma dark with parts of axillar complex pale, metasoma with some mediotergites, most laterotergites, sternites, and/or hypopygium pale. Antenna color: scape, pedicel, and flagellum pale. Coxae color (pro-, meso-, metacoxa): pale, pale, pale. Femora color (pro-, meso-, metafemur): pale, pale, pale. Tibiae color (pro-, meso-, metatibia): pale, pale, anteriorly pale/posteriorly dark. Tegula and humeral complex color: both pale. Pterostigma color: dark with pale spot at base. Fore wing veins color: mostly dark (a few veins may be unpigmented). Antenna length/body length: antenna about as long as body (head to apex of metasoma); if slightly shorter, at least extending beyond anterior 0.7 metasoma length. Body in lateral view: not distinctly flattened dorso–ventrally. Body length (head to apex of metasoma): 2.5–2.6 mm or 2.7–2.8 mm. Fore wing length: 2.7–2.8 mm or 2.9–3.0 mm. Ocular–ocellar line/posterior ocellus diameter: 2.0–2.2. Interocellar distance/posterior ocellus diameter: 1.7–1.9. Antennal flagellomerus 2 length/width: 2.3–2.5. Antennal flagellomerus 14 length/width: 1.1–1.3. Length of flagellomerus 2/length of flagellomerus 14: 2.3–2.5. Tarsal claws: simple. Metafemur length/width: 2.8–2.9. Metatibia inner spur length/metabasitarsus length: 0.4–0.5. Anteromesoscutum: mostly with deep, dense punctures (separated by less than 2.0 × its maximum diameter). Mesoscutellar disc: mostly smooth. Number of pits in scutoscutellar sulcus: 9 or 10. Maximum height of mesoscutellum lunules/maximum height of lateral face of mesoscutellum: 0.2–0.3. Propodeum areola: completely defined by carinae, including transverse carina extending to spiracle. Propodeum background sculpture: mostly sculptured. Mediotergite 1 length/width at posterior margin: 2.6–2.8. Mediotergite 1 shape: more or less parallel–sided. Mediotergite 1 sculpture: mostly smooth. Mediotergite 2 width at posterior margin/length: 3.6–3.9. Mediotergite 2 sculpture: mostly smooth. Outer margin of hypopygium: with a wide, medially folded, transparent, semi–desclerotized area; usually with 4 or more pleats. Ovipositor thickness: about same width throughout its length. Ovipositor sheaths length/metatibial length: 0.4–0.5. Length of fore wing veins r/2RS: 1.7–1.9. Length of fore wing veins 2RS/2M: 0.9–1.0. Length of fore wing veins 2M/(RS+M)b: 0.7–0.8. Pterostigma length/width: 2.6–3.0. Point of insertion of vein r in pterostigma: clearly beyond half way point length of pterostigma. Angle of vein r with fore wing anterior margin: clearly outwards, inclined towards fore wing apex. Shape of junction of veins r and 2RS in fore wing: distinctly but not strongly angled.

**Male.** As in female.

#### Molecular data.

Sequences in BOLD: 58, barcode compliant sequences: 50.

#### Biology/ecology.

Gregarious (Fig. 270). Host: Crambidae, *Ategumia lotanalis*.

#### Distribution.

Costa Rica, ACG.

#### Etymology.

We dedicate this species to Cinthia Barrantes in recognition of her diligent efforts for the ACG Programa Forestal.

### Apanteles ciriloumanai

Fernández-Triana sp. n.

http://zoobank.org/6D45529A-AB4F-46D7-A9D0-15DCD8F56AD8

http://species-id.net/wiki/Apanteles_ciriloumanai

Figs 174 , 308

Apanteles Rodriguez18 (Smith et al. 2006). Interim name provided by the authors.

#### Type locality.

COSTA RICA, Alajuela, ACG, Sector San Cristobal, Sendero Corredor, 620m, 10.87868, -85.38963.

#### Holotype.

♀ in CNC. Specimen labels: 1. DHJPAR0001616. 2. COSTA RICA: Alajuela, ACG, Sector San Cristobal, Sendero Corredor, 30.vii.2002, 620m, 10.87868, -85.38963, 02-SRNP-18526.

#### Paratypes.

20 ♀, 9 ♂ (BMNH, CNC, INBIO, INHS, NMNH). COSTA RICA, ACG database codes: DHJPAR0001562, DHJPAR0001616, DHJPAR0002645, DHJPAR0002659, DHJPAR0002685, DHJPAR0005299.

#### Description.

**Female.** Metatibia color (outer face): entirely or mostly (>0.7 metatibia length) dark brown to black, with yellow to white coloration usually restricted to anterior 0.2 or less. Fore wing veins color: veins C+Sc+R and R1 with brown coloration restricted narrowly to borders, interior area of those veins and pterostigma (and sometimes veins r, 2RS and 2M) transparent or white; other veins mostly transparent. Antenna length/body length: antenna about as long as body (head to apex of metasoma); if slightly shorter, at least extending beyond anterior 0.7 metasoma length. Body length (head to apex of metasoma): 2.3–2.4 mm, rarely 2.1–2.2 mm or 2.5–2.6 mm. Fore wing length: 2.5–2.6 mm, rarely 2.3–2.4 mm. Metafemur length/width: 2.8–2.9. Mediotergite 1 length/width at posterior margin: 2.7–2.8. Mediotergite 1 maximum width/width at posterior margin: 1.6–1.7. Ovipositor sheaths length/metafemur length: 0.7 or 0.8. Ovipositor sheaths length/metatibia length: 0.6 or 0.7.

#### Molecular data.

Sequences in BOLD: 10, barcode compliant sequences: 10.

#### Biology/ecology.

Gregarious (Fig. 308). Hosts: Hesperiidae, *Bungalotis erythus*.

#### Distribution.

Costa Rica, ACG.

#### Etymology.

We dedicate this species to Cirilo Umana recognition of his diligent efforts for the ACG Programa de Parataxónomos and Estación Biológica Llanura.

### Apanteles coffeellae

Muesebeck, 1958

http://species-id.net/wiki/Apanteles_coffeellae

Fig. 106

Apanteles coffeellae Muesebeck, 1958: 431.

#### Type locality.

GUADELOUPE, Lesser Antilles, locality not specified.

#### Holotype.

♀, NMNH (examined).

#### Description.

**Female.** Body color: body mostly dark except for some sternites which may be pale. Antenna color: scape, pedicel, and flagellum pale. Coxae color (pro-, meso-, metacoxa): pale, dark, dark or pale, pale, dark. Femora color (pro-, meso-, metafemur): pale, pale, pale. Tibiae color (pro-, meso-, metatibia): pale, pale, pale. Tegula and humeral complex color: both pale. Pterostigma color: dark. Fore wing veins color: partially pigmented (a few veins may be dark but most are pale). Antenna length/body length: antenna about as long as body (head to apex of metasoma); if slightly shorter, at least extending beyond anterior 0.7 metasoma length. Body in lateral view: not distinctly flattened dorso–ventrally. Body length (head to apex of metasoma): 2.0 mm or less. Fore wing length: 2.0 mm or less. Ocular–ocellar line/posterior ocellus diameter: 2.6 or more. Interocellar distance/posterior ocellus diameter: 1.4–1.6. Antennal flagellomerus 2 length/width: 2.3–2.5. Antennal flagellomerus 14 length/width: 1.4–1.6. Length of flagellomerus 2/length of flagellomerus 14: 2.0–2.2. Tarsal claws: simple. Metafemur length/width: 3.0–3.1. Metatibia inner spur length/metabasitarsus length: 0.4–0.5. Anteromesoscutum: mostly with shallow, sparse punctures (separated by more than 2.0 × its maximum diameter). Mesoscutellar disc: mostly smooth. Number of pits in scutoscutellar sulcus: 13 or 14. Maximum height of mesoscutellum lunules/maximum height of lateral face of mesoscutellum: 0.8 or more. Propodeum areola: completely defined by carinae, including transverse carina extending to spiracle. Propodeum background sculpture: partly sculptured, especially on anterior 0.5. Mediotergite 1 length/width at posterior margin: 4.1 or more. Mediotergite 1 shape: mostly parallel–sided for 0.5–0.7 of its length, then narrowing posteriorly so mediotergite anterior width >1.1 × posterior width. Mediotergite 1 sculpture: mostly smooth. Mediotergite 2 width at posterior margin/length: 4.0–4.3. Mediotergite 2 sculpture: mostly smooth. Outer margin of hypopygium: with a wide, medially folded, transparent, semi–desclerotized area; usually with 4 or more pleats (?). Ovipositor thickness: about same width throughout its length. Ovipositor sheaths length/metatibial length: 0.8–0.9. Length of fore wing veins r/2RS: 1.0 or less (?). Length of fore wing veins 2RS/2M: 2.1 or more. Length of fore wing veins 2M/(RS+M)b: 1.1–1.3. Pterostigma length/width: 2.1–2.5. Point of insertion of vein r in pterostigma: about half way point length of pterostigma. Angle of vein r with fore wing anterior margin: more or less perpendicular to fore wing margin. Shape of junction of veins r and 2RS in fore wing: evenly curved.

**Male.** Differs only in the darker legs, with metafemur and apical half of metatibia dark brown (Muesebeck 1958).

#### Molecular data.

No molecular data available for this species.

#### Biology/ecology.

Probably solitary. Hosts: Gracillariidae, *Acrocercops dives*; Lyonetiidae, *Leucoptera coffeella*.

#### Distribution.

Guadeloupe, Puerto Rico. No records known or expected for ACG or Costa Rica.

#### Comments.

Only known from specimens studied by Muesebeck (1958) when describing the species.

### Apanteles cristianalemani

Fernández-Triana sp. n.

http://zoobank.org/FBDD63B5-7D2C-4F5A-BFC5-11C158DC018F

http://species-id.net/wiki/Apanteles_cristianalemani

Fig. 72

#### Type locality.

COSTA RICA, Alajuela, ACG, Sector San Cristobal, Bosque Trampa Malaise, 815 meters, 10.86280, -85.38460.

#### Holotype.

♀ in CNC. Specimen labels: 1. DHJPAR0026016. 2. San Gerardo, MT, San Cristobal, 27 Aug-2 Sep 2007.

#### Description.

**Female.** Body color: body mostly dark except for some sternites which may be pale. Antenna color: scape, pedicel, and flagellum dark. Coxae color (pro-, meso-, metacoxa): pale, pale, partially pale/partially dark. Femora color (pro-, meso-, metafemur): pale, pale, mostly dark but with pale spot antero–ventrally. Tibiae color (pro-, meso-, metatibia): pale, pale, mostly pale but with posterior 0.2 or less dark. Tegula and humeral complex color: both dark. Pterostigma color: dark. Fore wing veins color: mostly dark (a few veins may be unpigmented). Antenna length/body length: antenna about as long as body (head to apex of metasoma); if slightly shorter, at least extending beyond anterior 0.7 metasoma length. Body in lateral view: not distinctly flattened dorso–ventrally. Body length (head to apex of metasoma): 2.3–2.4 mm. Fore wing length: 2.5–2.6 mm. Ocular–ocellar line/posterior ocellus diameter: 2.3–2.5. Interocellar distance/posterior ocellus diameter: 1.4–1.6. Antennal flagellomerus 2 length/width: 3.2 or more. Antennal flagellomerus 14 length/width: 1.7–1.9. Length of flagellomerus 2/length of flagellomerus 14: 2.0–2.2. Tarsal claws: with single basal spine–like seta. Metafemur length/width: 3.2–3.3. Metatibia inner spur length/metabasitarsus length: 0.4–0.5. Anteromesoscutum: mostly with deep, dense punctures (separated by less than 2.0 × its maximum diameter). Mesoscutellar disc: mostly punctured. Number of pits in scutoscutellar sulcus: 5 or 6. Maximum height of mesoscutellum lunules/maximum height of lateral face of mesoscutellum: 0.4–0.5. Propodeum areola: completely defined by carinae, including transverse carina extending to spiracle. Propodeum background sculpture: mostly sculptured. Mediotergite 1 length/width at posterior margin: 3.5–3.7. Mediotergite 1 shape: clearly narrowing towards posterior margin. Mediotergite 1 sculpture: mostly sculptured, excavated area centrally with transverse striation inside and/or a polished knob centrally on posterior margin of mediotergite. Mediotergite 2 width at posterior margin/length: 3.6–3.9. Mediotergite 2 sculpture: with some sculpture, mostly near posterior margin. Outer margin of hypopygium: with a wide, medially folded, transparent, semi–desclerotized area; usually with 4 or more pleats. Ovipositor thickness: about same width throughout its length (?). Ovipositor sheaths length/metatibial length: 0.8–0.9. Length of fore wing veins r/2RS: 1.0 or less. Length of fore wing veins 2RS/2M: 1.4–1.6. Length of fore wing veins 2M/(RS+M)b: 1.1–1.3. Pterostigma length/width: 3.1–3.5. Point of insertion of vein r in pterostigma: clearly beyond half way point length of pterostigma. Angle of vein r with fore wing anterior margin: clearly outwards, inclined towards fore wing apex. Shape of junction of veins r and 2RS in fore wing: strongly angulated, sometimes with a knob.

**Male.** Unknown.

#### Molecular data.

Sequences in BOLD: 1, barcode compliant sequences: 1.

#### Biology/ecology.

Malaise-trapped.

#### Distribution.

Costa Rica, ACG.

#### Etymology.

We dedicate this species to Cristián Alemán in recognition of his diligent efforts for the ACG Sector Marino.

### Apanteles cynthiacorderoae

Fernández-Triana sp. n.

http://zoobank.org/051A5346-EB12-4AC4-AB09-D731914A5564

http://species-id.net/wiki/Apanteles_cynthiacorderoae

Fig. 175

Apanteles Rodriguez59 (Smith et al. 2006). Interim name provided by the authors.

#### Type locality.

COSTA RICA, Guanacaste, ACG, Sector El Hacha, Sendero Bejuquilla, 280m, 11.03004, -85.52699.

#### Holotype.

♀ in CNC. Specimen labels: 1. COSTA RICA: Guanacaste, ACG, Sector El Hacha, Sendero Bejuquilla, 05.v.2000, 280m, 11.03004, -85.52699, DHJPAR0001578.

#### Paratypes.

2 ♀, 6 ♂ (CNC, NMNH). COSTA RICA, ACG database codes: DHJPAR0001578.

#### Description.

**Female.** Metatibia color (outer face): entirely or mostly (>0.7 metatibia length) dark brown to black, with yellow to white coloration usually restricted to anterior 0.2 or less. Fore wing veins color: veins C+Sc+R and R1 with brown coloration restricted narrowly to borders, interior area of those veins and pterostigma (and sometimes veins r, 2RS and 2M) transparent or white; other veins mostly transparent. Antenna length/body length: antenna about as long as body (head to apex of metasoma); if slightly shorter, at least extending beyond anterior 0.7 metasoma length. Body length (head to apex of metasoma): 2.3–2.4 mm, rarely 2.5–2.6 mm. Fore wing length: 2.5–2.6 mm. Metafemur length/width: 2.6–2.7 or 2.8–2.9. Mediotergite 1 length/width at posterior margin: 2.1–2.2. Mediotergite 1 maximum width/width at posterior margin: 1.4–1.5 or 1.6–1.7. Ovipositor sheaths length/metafemur length: 0.8 or 0.9. Ovipositor sheaths length/metatibia length: 0.7.

#### Molecular data.

Sequences in BOLD: 1, barcode compliant sequences: 1.

#### Biology/ecology.

Gregarious. Hosts: Hesperiidae, *Ocyba calathana*.

#### Distribution.

Costa Rica, ACG.

#### Etymology.

We dedicate this species to Cynthia Cordero in recognition of her diligent efforts to support accounting for INBio, Costa Rica’s Instituto Nacional de Biodiversidad.

### Apanteles deifiliadavilae

Fernández-Triana sp. n.

http://zoobank.org/027ADA8E-6CEA-4B61-955E-26F9843E9EE5

http://species-id.net/wiki/Apanteles_deifiliadavilae

Fig. 39 , 235

Apanteles Rodriguez171 (Smith et al. 2006). Interim name provided by the authors.

#### Type locality.

COSTA RICA, Alajuela, ACG, Sector Rincon Rain Forest, Estación Llanura, 135m, 10.93332, -85.25331.

#### Holotype.

♀ in CNC. Specimen labels: 1. DHJPAR0035483. 2. Voucher: D.H.Janzen & W.Hallwachs, DB: http://janzen.sas.upenn.edu, Area de Conservación Guanacaste, COSTA RICA, 09-SRNP-44142.

#### Description.

**Female.** Body color: body mostly dark except for some sternites which may be pale. Antenna color: scape, pedicel, and flagellum dark. Coxae color (pro-, meso-, metacoxa): dark, dark, dark. Femora color (pro-, meso-, metafemur): pale, pale, mostly pale but posterior 0.2 or less dark. Tibiae color (pro-, meso-, metatibia): pale, pale, mostly pale but with posterior 0.2 or less dark. Tegula and humeral complex color: both pale. Pterostigma color: dark. Fore wing veins color: mostly dark (a few veins may be unpigmented). Antenna length/body length: antenna about as long as body (head to apex of metasoma); if slightly shorter, at least extending beyond anterior 0.7 metasoma length. Body in lateral view: not distinctly flattened dorso–ventrally. Body length (head to apex of metasoma): 3.5–3.6 mm. Fore wing length: 3.3–3.4 mm. Ocular–ocellar line/posterior ocellus diameter: 1.7–1.9. Interocellar distance/posterior ocellus diameter: 1.4–1.6. Antennal flagellomerus 2 length/width: 2.3–2.5. Antennal flagellomerus 14 length/width: 1.4–1.6. Length of flagellomerus 2/length of flagellomerus 14: 2.3–2.5. Tarsal claws: with single basal spine–like seta. Metafemur length/width: 2.8–2.9. Metatibia inner spur length/metabasitarsus length: 0.4–0.5. Anteromesoscutum: mostly with shallow, dense punctures (separated by less than 2.0 × its maximum diameter). Mesoscutellar disc: mostly smooth. Number of pits in scutoscutellar sulcus: 11 or 12. Maximum height of mesoscutellum lunules/maximum height of lateral face of mesoscutellum: 0.4–0.5. Propodeum areola: completely defined by carinae, but only partial or absent transverse carina. Propodeum background sculpture: mostly sculptured. Mediotergite 1 length/width at posterior margin: 2.6–2.8. Mediotergite 1 shape: mostly parallel–sided for 0.5–0.7 of its length, then narrowing posteriorly so mediotergite anterior width >1.1 × posterior width. Mediotergite 1 sculpture: mostly sculptured, excavated area centrally with transverse striation inside and/or a polished knob centrally on posterior margin of mediotergite. Mediotergite 2 width at posterior margin/length: 1.6–1.9. Mediotergite 2 sculpture: mostly smooth. Outer margin of hypopygium: with a wide, medially folded, transparent, semi–desclerotized area; usually with 4 or more pleats. Ovipositor thickness: about same width throughout its length. Ovipositor sheaths length/metatibial length: 1.6–1.7. Length of fore wing veins r/2RS: 1.7–1.9. Length of fore wing veins 2RS/2M: 1.4–1.6. Length of fore wing veins 2M/(RS+M)b: 0.5–0.6. Pterostigma length/width: 2.6–3.0. Point of insertion of vein r in pterostigma: clearly beyond half way point length of pterostigma. Angle of vein r with fore wing anterior margin: more or less perpendicular to fore wing margin. Shape of junction of veins r and 2RS in fore wing: distinctly but not strongly angled.

**Male.** Unknown.

#### Molecular data.

Sequences in BOLD: 12, barcode compliant sequences:12.

#### Biology/ecology.

Solitary (Fig. 235). Hosts: Elachistidae, *Antaeotricha marmorea*, *Antaeotricha radicalis*, *Antaeotricha* spp., *Chlamydastis* Janzen10, *Stenoma* spp., feeding on Melastomataceae.

#### Distribution.

Costa Rica, ACG.

#### Etymology.

We dedicate this species to Deifilia Dávila in recognition of her diligent efforts for the ACG Programa de Asesoría Legal.

### Apanteles deplanatus

Muesebeck, 1957

http://species-id.net/wiki/Apanteles_deplanatus

Fig. 203

Apanteles deplanatus Muesebeck, 1957: 24.

#### Type locality.

MEXICO, locality not specified (Muesebeck 1957).

#### Holotype.

♀, NMNH (examined).

#### Material Examined.

1 ♀, 1 ♂ (CNC), MEXICO: Nayarit, Tepic, Ingenio de Puga, 21-24.v.1984, Bennet, Browning & Melton.

#### Description.

**Female.** Body color: body mostly dark except for some sternites which may be pale. Antenna color: scape and/or pedicel pale, flagellum dark or scape, pedicel, and flagellum pale. Coxae color (pro-, meso-, metacoxa): pale, dark, dark. Femora color (pro-, meso-, metafemur): anteriorly dark/posteriorly pale, dark, dark. Tibiae color (pro-, meso-, metatibia): pale, pale, dark. Tegula and humeral complex color: both pale. Pterostigma color: mostly pale and/or transparent, with thin dark borders. Fore wing veins color: mostly white or entirely transparent. Antenna length/body length: antenna very short, barely or not extending beyond mesosoma length. Body in lateral view: distinctly flattened dorso–ventrally. Body length (head to apex of metasoma): 2.0 mm or less or 2.1–2.2 mm. Fore wing length: 2.0 mm or less or 2.1–2.2 mm. Ocular–ocellar line/posterior ocellus diameter: 2.6 or more. Interocellar distance/posterior ocellus diameter: 1.7–1.9. Antennal flagellomerus 2 length/width: 1.4–1.6. Antennal flagellomerus 14 length/width: 1.0 or less. Length of flagellomerus 2/length of flagellomerus 14: 1.7–1.9. Tarsal claws: simple. Metafemur length/width: 2.6–2.7. Metatibia inner spur length/metabasitarsus length: 0.4–0.5. Anteromesoscutum: mostly smooth. Mesoscutellar disc: mostly smooth. Number of pits in scutoscutellar sulcus: 11 or 12 or 13 or 14. Maximum height of mesoscutellum lunules/maximum height of lateral face of mesoscutellum: 0.6–0.7. Propodeum areola: partially defined by carinae on posterior 0.3–0.5 of its length, widely open anteriorly. Propodeum background sculpture: mostly smooth except around the areola or partly sculptured, especially on posterior 0.5. Mediotergite 1 length/width at posterior margin: 2.9–3.1. Mediotergite 1 shape: clearly narrowing towards posterior margin. Mediotergite 1 sculpture: with some sculpture near lateral margins and/or posterior 0.2–0.4 of mediotergite. Mediotergite 2 width at posterior margin/length: 1.6–1.9. Mediotergite 2 sculpture: mostly smooth. Outer margin of hypopygium: with a wide, medially folded, transparent, semi–desclerotized area; usually with 4 or more pleats. Ovipositor thickness: about same width throughout its length (?) or anterior width at most 2.0 × posterior width (beyond ovipositor constriction) (?). Ovipositor sheaths length/metatibial length: 0.6–0.7. Length of fore wing veins r/2RS: 1.4–1.6. Length of fore wing veins 2RS/2M: 0.8 or less. Length of fore wing veins 2M/(RS+M)b: 1.1–1.3. Pterostigma length/width: 2.6–3.0. Point of insertion of vein r in pterostigma: about half way point length of pterostigma. Angle of vein r with fore wing anterior margin: more or less perpendicular to fore wing margin. Shape of junction of veins r and 2RS in fore wing: distinctly but not strongly angled.

#### Molecular data.

Sequences in BOLD: 2, barcode compliant sequences: 2.

#### Biology/ecology.

Gregarious. Hosts: Crambidae, *Diatraea considerata*, *Diatraea magnifactella*.

#### Distribution.

Mexico. We have no reason to suspect that this species occurs in ACG.

#### Comments.

Austin and Dangerfield (1989) revised this species and provided additional photos and details.

### Apanteles diatraeae

Muesebeck, 1921

http://species-id.net/wiki/Apanteles_diatraeae

Fig. 204

Apanteles diatraeae Muesebeck, 1921: 520.

#### Type locality.

CUBA, Central Mercedes.

#### Holotype.

♀, NMNH (examined).

#### Material Examined.

1 ♀, paratype (CNC), CUBA: Central Mercedes, ix.1918, T.E. Holloway, ex *Diatraea* sp.; 2 ♀ (CNC), UNITED STATES: AZ, Tucson, 23.vi.1923, E.V. Walter.

#### Description.

**Female.** Body color: body mostly dark except for some sternites which may be pale. Antenna color: scape and/or pedicel dark, flagellum pale. Coxae color (pro-, meso-, metacoxa): pale, dark, dark. Femora color (pro-, meso-, metafemur): anteriorly dark/posteriorly pale, dark, dark. Tibiae color (pro-, meso-, metatibia): pale, pale, dark. Tegula and humeral complex color: both dark or both pale (?). Pterostigma color: entirely pale or transparent, translucent. Fore wing veins color: mostly white or entirely transparent. Antenna length/body length: antenna very short, barely or not extending beyond mesosoma length. Body in lateral view: distinctly flattened dorso–ventrally. Body length (head to apex of metasoma): 2.1–2.2 mm. Fore wing length: 2.1–2.2 mm. Ocular–ocellar line/posterior ocellus diameter: 2.6 or more. Interocellar distance/posterior ocellus diameter: 2.3–2.5. Antennal flagellomerus 2 length/width: 2.0–2.2. Antennal flagellomerus 14 length/width: 1.1–1.3. Length of flagellomerus 2/length of flagellomerus 14: 1.7–1.9. Tarsal claws: with single basal spine–like seta. Metafemur length/width: 2.8–2.9. Metatibia inner spur length/metabasitarsus length: 0.4–0.5. Anteromesoscutum: mostly smooth or with shallow sparse punctures, except for anterior 0.3 where it has deeper and/or denser punctures. Mesoscutellar disc: mostly smooth. Number of pits in scutoscutellar sulcus: 7 or 8. Maximum height of mesoscutellum lunules/maximum height of lateral face of mesoscutellum: 0.6–0.7. Propodeum areola: completely defined by carinae, but only partial or absent transverse carina. Propodeum background sculpture: partly sculptured, especially on posterior 0.5. Mediotergite 1 length/width at posterior margin: 2.0–2.2. Mediotergite 1 shape: more or less parallel–sided. Mediotergite 1 sculpture: mostly sculptured, excavated area centrally with transverse striation inside and/or a polished knob centrally on posterior margin of mediotergite. Mediotergite 2 width at posterior margin/length: 1.6–1.9. Mediotergite 2 sculpture: mostly smooth. Outer margin of hypopygium: with a wide, medially folded, transparent, semi–desclerotized area; usually with 4 or more pleats. Ovipositor thickness: anterior width at most 2.0 × posterior width (beyond ovipositor constriction). Ovipositor sheaths length/metatibial length: 0.8–0.9. Length of fore wing veins r/2RS: 1.4–1.6. Length of fore wing veins 2RS/2M: 1.1–1.3. Length of fore wing veins 2M/(RS+M)b: 0.7–0.8. Pterostigma length/width: 2.6–3.0. Point of insertion of vein r in pterostigma: about half way point length of pterostigma. Angle of vein r with fore wing anterior margin: more or less perpendicular to fore wing margin. Shape of junction of veins r and 2RS in fore wing: strongly angulated, sometimes with a knob.

#### Molecular data.

No molecular data available for this species.

#### Biology/ecology.

Gregarious, dirty whitish cocoons, cemented together in a long slender row but not surrounded by loose silk (Muesebeck 1921). Hosts: Crambidae (commonly called Pyralidae in older literature), *Diatraea grandiosella*, *Diatraea impersonatella*, *Diatraea lineolata*, *Diatraea magnificata*, *Diatraea muellerella*, *Diatraea saccharalis*, *Diatraea* sp. In the past, the Pyralidae species *Galleria mellonella* has also been recorded as a host (Paddock 1933), a record that might best be questioned.

#### Distribution.

Widely distributed in southern US, Mesoamerica and the northern part of South America; introduced in France and India (Yu et al. 2012). We have no reason to suspect that this species occurs in ACG.

#### Comments.

This is the commonest braconid parasitoid of *Diatraea* spp., along with *Cotesia flavipes*, but it does not appear to extend much into South America (Austin and Dangerfield 1989).

### Apanteles dickyui

Fernández-Triana sp. n.

http://zoobank.org/8461CE9C-688B-41C1-A4BC-701B6E5FD850

http://species-id.net/wiki/Apanteles_dickyui

Fig. 110

#### Type locality.

COSTA RICA, Alajuela, ACG, Sector Rincon Rain Forest, Estación Caribe, 415m, 10.90187, -85.27495.

#### Holotype.

♀ in CNC. Specimen labels: 1. DHJPAR0026091. 2. COSTA RICA, Alajuela, ACG, Sector Rincon Rain Forest, Estación Caribe, 10.viii.2007, 10.90187°N, 85.27495°W, 415m, DHJPAR0026091. 3. Caribe: Est. Caribe, Date: 4-10 Aug 07.

#### Description.

**Female.** Body color: body mostly dark except for some sternites which may be pale. Antenna color: scape, pedicel, and flagellum dark. Coxae color (pro-, meso-, metacoxa): pale, dark, dark. Femora color (pro-, meso-, metafemur): pale, pale, dark. Tibiae color (pro-, meso-, metatibia): pale, pale, anteriorly pale/posteriorly dark. Tegula and humeral complex color: both pale. Pterostigma color: mostly pale and/or transparent, with thin dark borders. Fore wing veins color: partially pigmented (a few veins may be dark but most are pale). Antenna length/body length: antenna about as long as body (head to apex of metasoma); if slightly shorter, at least extending beyond anterior 0.7 metasoma length. Body in lateral view: not distinctly flattened dorso–ventrally. Body length (head to apex of metasoma): 2.1–2.2 mm. Fore wing length: 2.3–2.4 mm. Ocular–ocellar line/posterior ocellus diameter: 2.0–2.2. Interocellar distance/posterior ocellus diameter: 1.1–1.3. Antennal flagellomerus 2 length/width: 3.2 or more. Antennal flagellomerus 14 length/width: 1.4–1.6. Length of flagellomerus 2/length of flagellomerus 14: 2.0–2.2. Tarsal claws: simple. Metafemur length/width: 3.2–3.3. Metatibia inner spur length/metabasitarsus length: 0.4–0.5. Anteromesoscutum: mostly with deep, dense punctures (separated by less than 2.0 × its maximum diameter). Mesoscutellar disc: with a few sparse punctures. Number of pits in scutoscutellar sulcus: 9 or 10. Maximum height of mesoscutellum lunules/maximum height of lateral face of mesoscutellum: 0.6–0.7. Propodeum areola: completely defined by carinae, including transverse carina extending to spiracle. Propodeum background sculpture: partly sculptured, especially on anterior 0.5. Mediotergite 1 length/width at posterior margin: 2.3–2.5. Mediotergite 1 shape: mostly parallel–sided for 0.5–0.7 of its length, then narrowing posteriorly so mediotergite anterior width >1.1 × posterior width. Mediotergite 1 sculpture: mostly sculptured, excavated area centrally with transverse striation inside and/or a polished knob centrally on posterior margin of mediotergite. Mediotergite 2 width at posterior margin/length: 4.0–4.3. Mediotergite 2 sculpture: mostly smooth, with weak sculpture on anterior margin. Outer margin of hypopygium: with a wide, medially folded, transparent, semi–desclerotized area; usually with 4 or more pleats. Ovipositor thickness: about same width throughout its length. Ovipositor sheaths length/metatibial length: 1.0–1.1. Length of fore wing veins r/2RS: 1.4–1.6. Length of fore wing veins 2RS/2M: 1.4–1.6. Length of fore wing veins 2M/(RS+M)b: 0.7–0.8. Pterostigma length/width: 3.1–3.5. Point of insertion of vein r in pterostigma: clearly beyond half way point length of pterostigma. Angle of vein r with fore wing anterior margin: more or less perpendicular to fore wing margin. Shape of junction of veins r and 2RS in fore wing: distinctly but not strongly angled.

**Male.** Unknown.

#### Molecular data.

Sequences in BOLD: 1, barcode compliant sequences: 1.

#### Biology/ecology.

Malaise-trapped.

#### Distribution.

Costa Rica, ACG.

#### Etymology.

The senior author dedicates this species to Dicky Yu (CNC, Ottawa, Canada) in appreciation of his support, and for creating the extremely valuable tool that is Taxapad.

### Apanteles didiguadamuzi

Fernández-Triana sp. n.

http://zoobank.org/20F36781-6A01-4C96-92FE-7A179812BC18

http://species-id.net/wiki/Apanteles_didiguadamuzi

Figs 6 , 212

Apanteles Rodriguez33. Smith et al. (2008). Interim name provided by the authors.

#### Type locality.

COSTA RICA, Alajuela, ACG, Sector San Cristobal, Sendero Perdido, 620m, 10.8794, -85.38607.

#### Holotype.

♀ in CNC. Specimen labels: 1. Costa Rica: Alajuela, ACG, Sector San Cristobal, Sendero Perdido, 18.vii.2000, 620m, 10.8794, -85.38607, DHJPAR0001552.

#### Paratypes.

24 ♀, 3 ♂ (BMNH, CNC, INBIO, INHS, NMNH). COSTA RICA, ACG database codes: DHJPAR0001552, DHJPAR0038142, 00-SRNP-12094, 00-SRNP-12099, 09-SRNP-5112.

#### Description.

**Female.** Body color: body mostly dark except for some sternites which may be pale. Antenna color: scape, pedicel, and flagellum dark. Coxae color (pro-, meso-, metacoxa): dark, dark, dark. Femora color (pro-, meso-, metafemur): pale, dark, dark. Tibiae color (pro-, meso-, metatibia): pale, pale, dark. Tegula and humeral complex color: tegula pale, humeral complex half pale/half dark. Pterostigma color: mostly pale and/or transparent, with thin dark borders. Fore wing veins color: mostly white or entirely transparent. Antenna length/body length: antenna shorter than body (head to apex of metasoma), not extending beyond anterior 0.7 metasoma length. Body in lateral view: not distinctly flattened dorso–ventrally. Body length (head to apex of metasoma): 2.7–2.8 mm or 2.9–3.0 mm. Fore wing length: 2.7–2.8 mm or 2.9–3.0 mm. Ocular–ocellar line/posterior ocellus diameter: 2.0–2.2 or 2.3–2.5. Interocellar distance/posterior ocellus diameter: 1.4–1.6, rarely 1.7–1.9. Antennal flagellomerus 2 length/width: 2.6–2.8. Antennal flagellomerus 14 length/width: 1.1–1.3. Length of flagellomerus 2/length of flagellomerus 14: 2.0–2.2. Tarsal claws: with single basal spine–like seta. Metafemur length/width: 3.4–3.5. Metatibia inner spur length/metabasitarsus length: 0.4–0.5. Anteromesoscutum: mostly with deep, dense punctures (separated by less than 2.0 × its maximum diameter). Mesoscutellar disc: with a few sparse punctures. Number of pits in scutoscutellar sulcus: 9 or 10. Maximum height of mesoscutellum lunules/maximum height of lateral face of mesoscutellum: 0.4–0.5. Propodeum areola: completely defined by carinae, including transverse carina extending to spiracle. Propodeum background sculpture: mostly sculptured. Mediotergite 1 length/width at posterior margin: 1.7–1.9 or 2.0–2.2. Mediotergite 1 shape: more or less parallel–sided. Mediotergite 1 sculpture: mostly sculptured, excavated area centrally with transverse striation inside and/or a polished knob centrally on posterior margin of mediotergite. Mediotergite 2 width at posterior margin/length: 2.8–3.1 or 3.2–3.5. Mediotergite 2 sculpture: mostly smooth. Outer margin of hypopygium: with a wide, medially folded, transparent, semi–desclerotized area; usually with 4 or more pleats. Ovipositor thickness: about same width throughout its length. Ovipositor sheaths length/metatibial length: 1.4–1.5 or 1.6–1.7. Length of fore wing veins r/2RS: 1.1–1.3. Length of fore wing veins 2RS/2M: 1.1–1.3. Length of fore wing veins 2M/(RS+M)b: 0.9–1.0. Pterostigma length/width: 3.1–3.5. Point of insertion of vein r in pterostigma: about half way point length of pterostigma. Angle of vein r with fore wing anterior margin: more or less perpendicular to fore wing margin. Shape of junction of veins r and 2RS in fore wing: distinctly but not strongly angled.

**Male.** As in female but with darker legs and smoother mediotergite 1.

#### Molecular data.

Sequences in BOLD: 10, barcode compliant sequences: 9.

#### Biology/ecology.

Gregarious (Fig. 212). Host: Elachistidae, *Stenoma* spp., *Anadasmus* Janzen25.

#### Distribution.

Costa Rica, ACG.

#### Etymology.

We dedicate this species to Didi Guadamuz in recognition of his diligent efforts in the ACG Programa de Seguridad.

### Apanteles diegoalpizari

Fernández-Triana sp. n.

http://zoobank.org/66FB70BB-9DAC-4E01-AE90-5CF5FE252040

http://species-id.net/wiki/Apanteles_diegoalpizari

Figs 73 , 261

Apanteles Rodriguez114 (Smith et al. 2006). Interim name provided by the authors.

#### Type locality.

COSTA RICA, Guanacaste, ACG, Sector Del Oro, Monte Cristo, 525m, 11.01373, -85.42531.

#### Holotype.

♀ in CNC. Specimen labels: 1. DHJPAR0020597. 2. Voucher: D.H.Janzen & W.Hallwachs, DB: http://janzen.sas.upenn.edu, Area de Conservación Guanacaste, COSTA RICA, 07-SRNP-24360.

#### Paratypes.

1 ♀, 2 ♂ (CNC). COSTA RICA, ACG database codes: DHJPAR0020599, DHJPAR0020601, DHJPAR0039031.

#### Description.

**Female.** Body color: body mostly dark except for some sternites which may be pale. Antenna color: scape and/or pedicel pale, flagellum dark. Coxae color (pro-, meso-, metacoxa): pale, pale, pale. Femora color (pro-, meso-, metafemur): pale, pale, mostly pale but posterior 0.2 or less dark. Tibiae color (pro-, meso-, metatibia): pale, pale, anteriorly pale/posteriorly dark. Tegula and humeral complex color: both pale. Pterostigma color: dark with pale spot at base. Fore wing veins color: mostly dark (a few veins may be unpigmented). Antenna length/body length: antenna about as long as body (head to apex of metasoma); if slightly shorter, at least extending beyond anterior 0.7 metasoma length. Body in lateral view: not distinctly flattened dorso–ventrally. Body length (head to apex of metasoma): 3.7–3.8 mm. Fore wing length: 3.7–3.8 mm. Ocular–ocellar line/posterior ocellus diameter: 2.0–2.2. Interocellar distance/posterior ocellus diameter: 2.0–2.2. Antennal flagellomerus 2 length/width: 2.6–2.8. Tarsal claws: with single basal spine–like seta. Metafemur length/width: 3.0–3.1. Metatibia inner spur length/metabasitarsus length: 0.4–0.5. Anteromesoscutum: mostly with deep, dense punctures (separated by less than 2.0 × its maximum diameter). Mesoscutellar disc: mostly punctured. Number of pits in scutoscutellar sulcus: 7 or 8. Maximum height of mesoscutellum lunules/maximum height of lateral face of mesoscutellum: 0.4–0.5. Propodeum areola: completely defined by carinae, including transverse carina extending to spiracle. Propodeum background sculpture: mostly sculptured. Mediotergite 1 length/width at posterior margin: 2.3–2.5. Mediotergite 1 shape: mostly parallel–sided for 0.5–0.7 of its length, then narrowing posteriorly so mediotergite anterior width >1.1 × posterior width. Mediotergite 1 sculpture: with some sculpture near lateral margins and/or posterior 0.2–0.4 of mediotergite. Mediotergite 2 width at posterior margin/length: 3.2–3.5. Mediotergite 2 sculpture: mostly smooth. Outer margin of hypopygium: with a wide, medially folded, transparent, semi–desclerotized area; usually with 4 or more pleats. Ovipositor thickness: anterior width at most 2.0 × posterior width (beyond ovipositor constriction). Ovipositor sheaths length/metatibial length: 1.0–1.1. Length of fore wing veins r/2RS: 2.3 or more. Length of fore wing veins 2RS/2M: 1.1–1.3. Length of fore wing veins 2M/(RS+M)b: 0.5–0.6. Pterostigma length/width: 2.6–3.0. Point of insertion of vein r in pterostigma: about half way point length of pterostigma. Angle of vein r with fore wing anterior margin: clearly outwards, inclined towards fore wing apex. Shape of junction of veins r and 2RS in fore wing: distinctly but not strongly angled.

**Male.** As female, but scape brown.

#### Molecular data.

Sequences in BOLD: 10, barcode compliant sequences: 10.

#### Biology/ecology.

Solitary (Fig. 261). Hosts: Crambidae, *Omiodes humeralis*, *Omiodes* Janzen05.

#### Distribution.

Costa Rica, ACG.

#### Etymology.

We dedicate this species to Diego Alpízar in recognition of his diligent efforts for the ACG Sector Marino.

### Apanteles diegotorresi

Fernández-Triana sp. n.

http://zoobank.org/91DF0E35-1105-443A-8E29-1821E6A380D2

http://species-id.net/wiki/Apanteles_diegotorresi

Figs 112 , 278

Apanteles Rodriguez16 (Smith et al. 2006). Interim name provided by the authors.

#### Type locality.

COSTA RICA, Guanacaste, ACG, Sector Del Oro, Bosque Aguirre, 620m, 11.00060, -85.43800.

#### Holotype.

♀ in CNC. Specimen labels: 1. Costa Rica, Guanacaste, ACG, Del Oro, Bosque Aguirre, 14 May 2002, Manuel Pereira. 2. 02-SRNP-14931, Achlyodes busirus, on Citrus sinensis. 3. DHJPAR0005259.

#### Paratypes.

9 ♀, 4 ♂ (BMNH, CNC, INBIO, INHS, NMNH). COSTA RICA, ACG database codes: DHJPAR0004054, DHJPAR0004060, DHJPAR0004064, DHJPAR0004069, DHJPAR0004076, DHJPAR0004088, DHJPAR0005257, DHJPAR0005258, DHJPAR0005260, DHJPAR0005262, DHJPAR0005282, DHJPAR0012477, DHJPAR00012966.

#### Description.

**Female.** Body color: body mostly dark except for some sternites which may be pale. Antenna color: scape, pedicel, and flagellum dark. Coxae color (pro-, meso-, metacoxa): dark, dark, dark. Femora color (pro-, meso-, metafemur): pale, dark, dark. Tibiae color (pro-, meso-, metatibia): pale, pale, anteriorly pale/posteriorly dark. Tegula and humeral complex color: tegula pale, humeral complex half pale/half dark. Pterostigma color: mostly pale and/or transparent, with thin dark borders. Fore wing veins color: mostly white or entirely transparent. Antenna length/body length: antenna about as long as body (head to apex of metasoma); if slightly shorter, at least extending beyond anterior 0.7 metasoma length. Body in lateral view: not distinctly flattened dorso–ventrally. Body length (head to apex of metasoma): 3.9–4.0 mm, rarely 4.0 mm or more. Fore wing length: 3.7–3.8 mm, rarely 3.9–4.0 mm. Ocular–ocellar line/posterior ocellus diameter: 1.7–1.9. Interocellar distance/posterior ocellus diameter: 2.3–2.5. Antennal flagellomerus 2 length/width: 2.6–2.8. Antennal flagellomerus 14 length/width: 1.4–1.6. Length of flagellomerus 2/length of flagellomerus 14: 2.0–2.2. Tarsal claws: with single basal spine–like seta. Metafemur length/width: 3.0–3.1. Metatibia inner spur length/metabasitarsus length: 0.4–0.5. Anteromesoscutum: mostly with deep, dense punctures (separated by less than 2.0 × its maximum diameter). Mesoscutellar disc: with punctures near margins, central part mostly smooth. Number of pits in scutoscutellar sulcus: 9 or 10. Maximum height of mesoscutellum lunules/maximum height of lateral face of mesoscutellum: 0.6–0.7. Propodeum areola: completely defined by carinae, including transverse carina extending to spiracle. Propodeum background sculpture: mostly sculptured. Mediotergite 1 length/width at posterior margin: 3.2–3.4. Mediotergite 1 shape: mostly parallel–sided for 0.5–0.7 of its length, then narrowing posteriorly so mediotergite anterior width >1.1 × posterior width. Mediotergite 1 sculpture: mostly sculptured, excavated area centrally with transverse striation inside and/or a polished knob centrally on posterior margin of mediotergite. Mediotergite 2 width at posterior margin/length: 2.0–2.3. Mediotergite 2 sculpture: mostly smooth. Outer margin of hypopygium: with a wide, medially folded, transparent, semi–desclerotized area; usually with 4 or more pleats. Ovipositor thickness: anterior width 3.0–5.0 × posterior width (beyond ovipositor constriction). Ovipositor sheaths length/metatibial length: 1.2–1.3. Length of fore wing veins r/2RS: 2.0–2.2. Length of fore wing veins 2RS/2M: 1.4–1.6. Length of fore wing veins 2M/(RS+M)b: 0.7–0.8. Pterostigma length/width: 3.6 or more. Point of insertion of vein r in pterostigma: about half way point length of pterostigma. Angle of vein r with fore wing anterior margin: more or less perpendicular to fore wing margin. Shape of junction of veins r and 2RS in fore wing: distinctly but not strongly angled.

**Male.** Similar to female, but with legs having a darker coloration and mediotergite 2 being more trapezoidal.

#### Molecular data.

Sequences in BOLD: 30, barcode compliant sequences: 18.

#### Biology/ecology.

Solitary (Fig. 278). Host: Hesperiidae, *Achlyodes busirus*.

#### Distribution.

Costa Rica, ACG.

#### Comments.

The 5 mm length cocoons are about usual for solitary species but about twice the length of the cocoons of gregarious species. This species is unique in its combination of broadly rectangular mediotergite 2 (its apical width 2.2 × or less than its median length), lateral face of scutellum with polished area 0.7 × face height, and thick ovipositor (as thick as or thicker than width of median flagellomerus, and with anterior width 3.0–5.0 × its posterior width beyond the constriction). The ovipositor thickness clearly separates this species from other groups with relatively broad mediotergite 2 (all of which have a thin ovipositor).

#### Etymology.

We dedicate this species to Diego Torres in recognition of his diligent efforts for the ACG Programa de Ecoturismo.

### Apanteles diniamartinezae

Fernández-Triana sp. n.

http://zoobank.org/523950F7-C926-4391-8D27-0D7A18852EA1

http://species-id.net/wiki/Apanteles_diniamartinezae

Figs 176 , 309

Apanteles Rodriguez20 (Smith et al. 2006). Interim name provided by the authors.

#### Type locality.

COSTA RICA, Guanacaste, ACG, Sector Cacao, Estación Cacao, 1150m, 10.92691, -85.46822.

#### Holotype.

♀ in CNC. Specimen labels: 1. DHJPAR0002688. 2. COSTA RICA: Guanacaste, Area de Consveración Guanacaste: Sector Cacao: Estación Cacao, 10/15/2002, Mariano Pereira. 3. 02-SRNP-24195, *Astraptes augeas*, feeding on *Hampea appendiculata*.

#### Paratypes.

39 ♀, 40 ♂ (BMNH, CNC, INBIO, INHS, NMNH). COSTA RICA, ACG database codes: See Appendix 2 for detailed label data.

#### Description.

**Female.** Metatibia color (outer face): entirely or mostly (>0.7 metatibia length) dark brown to black, with yellow to white coloration usually restricted to anterior 0.2 or less. Fore wing veins color: veins C+Sc+R and R1 with brown coloration restricted narrowly to borders, interior area of those veins and pterostigma (and sometimes veins r, 2RS and 2M) transparent or white; other veins mostly transparent. Antenna length/body length: antenna shorter than body (head to apex of metasoma), not extending beyond anterior 0.7 metasoma length. Body length (head to apex of metasoma): 2.1–2.2 mm or 2.3–2.4 mm. Fore wing length: 2.3–2.4 mm or 2.5–2.6 mm. Metafemur length/width: 2.8–2.9, 3.0–3.1, rarely 3.2–3.3. Mediotergite 1 length/width at posterior margin: 2.1–2.2, 2.3–2.4, rarely 2.9 or more. Mediotergite 1 maximum width/width at posterior margin: 1.4–1.5 or 1.6–1.7. Ovipositor sheaths length/metafemur length: 0.8 or 0.9. Ovipositor sheaths length/metatibia length: 0.7 or 0.8.

#### Molecular data.

Sequences in BOLD: 75, barcode compliant sequences: 63.

#### Biology/ecology.

Gregarious (Fig. 309). Hosts: Hesperiidae, *Astraptes augeas*, *Astraptes obstupefactus*, *Astraptes syncedoche*, *Astraptes inflatio*, *Astraptes fruticibus*.

#### Distribution.

Costa Rica, ACG.

#### Etymology.

We dedicate this species to Dinia Martínez recognition of her diligent efforts for the ACG Programa de Parataxónomos and Estación Biológica Quica.

### Apanteles duniagarciae

Fernández-Triana sp. n.

http://zoobank.org/BE0A4467-E634-46DB-B91D-E8163D53E968

http://species-id.net/wiki/Apanteles_duniagarciae

Figs 53 , 246

Apanteles Rodriguez07 (Smith et al. 2006), in part. Interim name provided by the authors.

#### Type locality.

COSTA RICA, Guanacaste, ACG, Sector Cacao, Sendero Arenales, 1080m, 10.92471, -85.46738.

#### Holotype.

♀ in CNC. Specimen labels: 1. COSTA RICA, Guanacaste, ACG, Sector Cacao, Sendero Arenales, 7.x.2000, Mariano Pereira. 2. 00-SRNP-10830, *Staphylus* same as 00-10628, On *Pleuropetalum sprucei*. 3. DHJPAR0001655.

#### Paratypes.

23 ♀, 5 ♂ (BMNH, CNC, INBIO, INHS, NMNH). COSTA RICA: ACG database codes: DHJPAR0005311, DHJPAR0005271, DHJPAR0003974, DHJPAR0003977, DHJPAR0005217, DHJPAR0003961, DHJPAR0012307.

#### Description.

**Female.** Body color: body mostly dark except for some sternites which may be pale. Antenna color: scape, pedicel, and flagellum dark. Coxae color (pro-, meso-, metacoxa): pale, dark, dark. Femora color (pro-, meso-, metafemur): pale, pale, mostly dark but with pale spot antero–ventrally. Tibiae color (pro-, meso-, metatibia): pale, pale, mostly pale but with posterior 0.2 or less dark. Tegula and humeral complex color: tegula dark, humeral complex pale. Pterostigma color: dark with pale spot at base. Fore wing veins color: mostly dark (a few veins may be unpigmented). Antenna length/body length: antenna shorter than body (head to apex of metasoma), not extending beyond anterior 0.7 metasoma length. Body in lateral view: not distinctly flattened dorso–ventrally. Body length (head to apex of metasoma): 2.7–2.8 mm or 2.9–3.0 mm. Fore wing length: 2.9–3.0 mm, rarely 3.1–3.2 mm. Ocular–ocellar line/posterior ocellus diameter: 2.3–2.5. Interocellar distance/posterior ocellus diameter: 1.7–1.9. Antennal flagellomerus 2 length/width: 2.6–2.8. Antennal flagellomerus 14 length/width: 1.1–1.3. Length of flagellomerus 2/length of flagellomerus 14: 2.3–2.5. Tarsal claws: with single basal spine–like seta. Metafemur length/width: 2.8–2.9. Metatibia inner spur length/metabasitarsus length: 0.6–0.7. Anteromesoscutum: mostly with deep, dense punctures (separated by less than 2.0 × its maximum diameter). Mesoscutellar disc: mostly punctured. Number of pits in scutoscutellar sulcus: 7 or 8 or 9 or 10. Maximum height of mesoscutellum lunules/maximum height of lateral face of mesoscutellum: 0.6–0.7. Propodeum areola: completely defined by carinae, including transverse carina extending to spiracle. Propodeum background sculpture: mostly sculptured. Mediotergite 1 length/width at posterior margin: 3.5–3.7. Mediotergite 1 shape: mostly parallel–sided for 0.5–0.7 of its length, then narrowing posteriorly so mediotergite anterior width >1.1 × posterior width. Mediotergite 1 sculpture: mostly sculptured, excavated area centrally with transverse striation inside and/or a polished knob centrally on posterior margin of mediotergite. Mediotergite 2 width at posterior margin/length: 2.0–2.3. Mediotergite 2 sculpture: mostly smooth or with some sculpture, mostly near posterior margin. Outer margin of hypopygium: with a medially folded, transparent, semi–desclerotized area; with 0–3 pleats visible. Ovipositor thickness: anterior width 3.0–5.0 × posterior width (beyond ovipositor constriction). Ovipositor sheaths length/metatibial length: 0.8–0.9, rarely 1.0–1.1. Length of fore wing veins r/2RS: 1.4–1.6. Length of fore wing veins 2RS/2M: 1.4–1.6. Length of fore wing veins 2M/(RS+M)b: 0.7–0.8. Pterostigma length/width: 2.6–3.0. Point of insertion of vein r in pterostigma: clearly beyond half way point length of pterostigma. Angle of vein r with fore wing anterior margin: clearly outwards, inclined towards fore wing apex. Shape of junction of veins r and 2RS in fore wing: distinctly but not strongly angled.

**Male.** As female, but darker coloured (especially on legs), and longer, narrower mediotergite 1.

#### Molecular data.

Sequences in BOLD: 6, barcode compliant sequences: 4.

#### Biology/ecology.

Gregarious (Fig. 246). Hosts: Hesperiidae: *Staphylus evemerus*, *Bolla zorilla* DHJ02. While this wasp is unambiguously an upper elevation species parasitizing the upper elevation ACG *Staphylus evemerus* (800–1000 m), the single rearing from the lower elevation very similar *Bolla zorilla* DHJ92 (620 m), a rain forest analogue to dry forest *Staphylus* is the product of the intesection of these two distributions.

#### Distribution.

Costa Rica, ACG.

#### Comments.

This species is closely related to *Apanteles ruthfrancoae* (see below) and was originally included under that species as *Apanteles* Rodriguez07 (Smith et al. 2006). However, consistent differences in morphology, elevational distribution, host records and barcodes support its status as a species on its own.

#### Etymology.

We dedicate this species to Dunia Garcia in recognition of her diligent efforts for the ACG Programa de Parataxónomos and Estación Biológica Cacao of ACG.

### Apanteles duvalierbricenoi

Fernández-Triana sp. n.

http://zoobank.org/57C7EE3F-4C49-41FE-9259-DA2FCDDF3CC0

http://species-id.net/wiki/Apanteles_duvalierbricenoi

Fig. 177

Apanteles Rodriguez165 (Smith et al. 2006). Interim name provided by the authors.

#### Type locality.

COSTA RICA, Guanacaste, ACG, Sector Santa Rosa, Cafetal, 280m, 10.85827, -85.61089.

#### Holotype.

♀ in CNC. Specimen labels: 1. DHJPAR0005228. 2. COSTA RICA: Guanacaste: Area de Conservación Guanacaste: Santa Rosa: Cafetal, 07/23/1992, gusaneros. 3. 92-SRNP-3782, Urbanus dorantes, Desmodium distortum.

#### Paratypes.

6 ♀, 2 ♂ (BMNH, CNC, NMNH). COSTA RICA, ACG database codes: 92-SRNP-3775, 92-SRNP-3792, 95-SRNP-20.

#### Description.

**Female.** Metatibia color (outer face): with extended pale coloration (light yellow to orange–yellow), ranging from 0.4 to almost entire metatibia length. Fore wing veins color: veins C+Sc+R and R1 with brown coloration restricted narrowly to borders, interior area of those veins and pterostigma (and sometimes veins r, 2RS and 2M) transparent or white; other veins mostly transparent. Antenna length/body length: antenna shorter than body (head to apex of metasoma), not extending beyond anterior 0.7 metasoma length. Body length (head to apex of metasoma): 2.3–2.4 mm or 2.5–2.6 mm. Fore wing length: 2.5–2.6 mm. Metafemur length/width: 3.0–3.1 or 3.2–3.3. Mediotergite 1 length/width at posterior margin: 2.7–2.8. Mediotergite 1 maximum width/width at posterior margin: 1.6–1.7. Ovipositor sheaths length/metafemur length: 0.8 or 0.9. Ovipositor sheaths length/metatibia length: 0.7 or 0.8.

#### Molecular data.

Sequences in BOLD: 7, barcode compliant sequences: 0.

#### Biology/ecology.

Gregarious. Host: Hesperiidae, *Urbanus dorantes*.

#### Distribution.

Costa Rica, ACG.

#### Etymology.

We dedicate this species to Duvalier Briceño in recognition of his diligent efforts for the ACG Parataxonomist Program and Estación Biológica Brasilia of ACG.

### Apanteles edgarjimenezi

Fernández-Triana sp. n.

http://zoobank.org/6F173ABA-82A3-48BF-ABCB-E58A4C0F0783

http://species-id.net/wiki/Apanteles_edgarjimenezi

Fig. 7

Apanteles Rodriguez107. Smith et al. (2008). Interim name provided by the authors.

#### Type locality.

COSTA RICA, Guanacaste, ACG, Sector Potrerillos, Potrerillos, 90m, 10.81534, -85.54359.

#### Holotype.

♀ in CNC. Specimen labels: 1. DHJPAR0034281. 2. COSTA RICA, Guanacaste, ACG, Sector Potrerillos, 90m, 10.81534 N, -85.54359 W, 19.i.2009, DHJPAR0034281. 3. Voucher: D.H.Janzen & W.Hallwachs, DB: http://janzen.sas.upenn.edu, Area de Conservación Guanacaste, COSTA RICA, 09-SRNP-12236.

#### Paratypes.

12 ♀, 2 ♂ (BMNH, CNC, INBIO, INHS, NMNH). COSTA RICA, ACG database codes: DHJPAR0034228, DHJPAR0034281.

#### Description.

**Female.** Body color: body mostly dark except for some sternites which may be pale. Antenna color: scape, pedicel, and flagellum dark. Coxae color (pro-, meso-, metacoxa): dark, dark, dark. Femora color (pro-, meso-, metafemur): anteriorly dark/posteriorly pale, dark, dark. Tibiae color (pro-, meso-, metatibia): pale, pale, anteriorly pale/posteriorly dark. Tegula and humeral complex color: tegula pale, humeral complex half pale/half dark. Pterostigma color: mostly pale and/or transparent, with thin dark borders. Fore wing veins color: partially pigmented (a few veins may be dark but most are pale). Antenna length/body length: antenna about as long as body (head to apex of metasoma); if slightly shorter, at least extending beyond anterior 0.7 metasoma length. Body in lateral view: not distinctly flattened dorso–ventrally. Body length (head to apex of metasoma): 2.7–2.8 mm or 2.9–3.0 mm. Fore wing length: 2.9–3.0 mm. Ocular–ocellar line/posterior ocellus diameter: 2.3–2.5. Interocellar distance/posterior ocellus diameter: 1.7–1.9. Antennal flagellomerus 2 length/width: 2.6–2.8. Antennal flagellomerus 14 length/width: 1.7–1.9. Length of flagellomerus 2/length of flagellomerus 14: 1.7–1.9. Tarsal claws: simple. Metafemur length/width: 3.2–3.3. Metatibia inner spur length/metabasitarsus length: 0.6–0.7. Anteromesoscutum: mostly with deep, dense punctures (separated by less than 2.0 × its maximum diameter). Mesoscutellar disc: with punctures near margins, central part mostly smooth. Number of pits in scutoscutellar sulcus: 7 or 8. Maximum height of mesoscutellum lunules/maximum height of lateral face of mesoscutellum: 0.4–0.5. Propodeum areola: completely defined by carinae, including transverse carina extending to spiracle. Propodeum background sculpture: mostly sculptured. Mediotergite 1 length/width at posterior margin: 2.0–2.2. Mediotergite 1 shape: mostly parallel–sided for 0.5–0.7 of its length, then narrowing posteriorly so mediotergite anterior width >1.1 × posterior width. Mediotergite 1 sculpture: mostly sculptured, excavated area centrally with transverse striation inside and/or a polished knob centrally on posterior margin of mediotergite. Mediotergite 2 width at posterior margin/length: 2.8–3.1. Mediotergite 2 sculpture: mostly smooth. Outer margin of hypopygium: with a wide, medially folded, transparent, semi–desclerotized area; usually with 4 or more pleats. Ovipositor thickness: about same width throughout its length. Ovipositor sheaths length/metatibial length: 1.4–1.5, rarely 1.6–1.7. Length of fore wing veins r/2RS: 1.4–1.6. Length of fore wing veins 2RS/2M: 1.4–1.6. Length of fore wing veins 2M/(RS+M)b: 0.5–0.6. Pterostigma length/width: 3.6 or more. Point of insertion of vein r in pterostigma: clearly beyond half way point length of pterostigma. Angle of vein r with fore wing anterior margin: clearly outwards, inclined towards fore wing apex. Shape of junction of veins r and 2RS in fore wing: distinctly but not strongly angled.

**Male.** As in female.

#### Molecular data.

Sequences in BOLD: 7, barcode compliant sequences: 7.

#### Biology/ecology.

Gregarious. Hosts: Elachistidae, *Stenoma completella*, *Stenoma luctifica*.

#### Distribution.

Costa Rica, ACG.

#### Etymology.

We dedicate this species to Edgar Jiménez in recognition of his diligent efforts for the ACG Programa de Educacion Biológica.

### Apanteles edithlopezae

Fernández-Triana sp. n.

http://zoobank.org/3522C140-F624-4B79-BF81-E6CF6E81B469

http://species-id.net/wiki/Apanteles_edithlopezae

Figs 92 , 271

#### Type locality.

COSTA RICA, Alajuela, ACG, Sector Rincon Rain Forest, Camino Albergue Oscar, 560m, 10.87741, -85.32363.

#### Holotype.

♀ in CNC. Specimen labels: 1. DHJPAR0038264. 2. Voucher: D.H.Janzen & W.Hallwachs, DB: http://janzen.sas.upenn.edu, Area de Conservación Guanacaste, COSTA RICA, 10-SRNP-445.

#### Paratypes.

14 ♀, 5 ♂ (BMNH, CNC, INBIO, INHS, NMNH). COSTA RICA, ACG database codes: DHJPAR0038143, DHJPAR0045146, 09-SRNP-32173, 10-SRNP-445.

#### Description.

**Female.** Body color: head dark, mesosoma dark with parts of axillar complex pale, metasoma with some mediotergites, most laterotergites, sternites, and/or hypopygium pale. Antenna color: scape, pedicel, and flagellum pale. Coxae color (pro-, meso-, metacoxa): pale, pale, pale. Femora color (pro-, meso-, metafemur): pale, pale, pale. Tibiae color (pro-, meso-, metatibia): pale, pale, mostly pale but with posterior 0.2 or less dark. Tegula and humeral complex color: both pale. Pterostigma color: dark. Fore wing veins color: mostly dark (a few veins may be unpigmented). Antenna length/body length: antenna about as long as body (head to apex of metasoma); if slightly shorter, at least extending beyond anterior 0.7 metasoma length. Body in lateral view: not distinctly flattened dorso–ventrally. Body length (head to apex of metasoma): 2.5–2.6 mm, 2.7–2.8 mm or 2.9–3.0 mm. Fore wing length: 2.7–2.8 mm, 2.9–3.0 mm or 3.1–3.2 mm. Ocular–ocellar line/posterior ocellus diameter: 2.0–2.2. Interocellar distance/posterior ocellus diameter: 1.7–1.9. Antennal flagellomerus 2 length/width: 3.2 or more. Antennal flagellomerus 14 length/width: 1.4–1.6. Length of flagellomerus 2/length of flagellomerus 14: 2.3–2.5. Tarsal claws: simple. Metafemur length/width: 2.8–2.9. Metatibia inner spur length/metabasitarsus length: 0.4–0.5. Anteromesoscutum: mostly with deep, dense punctures (separated by less than 2.0 × its maximum diameter). Mesoscutellar disc: mostly smooth. Number of pits in scutoscutellar sulcus: 7 or 8. Maximum height of mesoscutellum lunules/maximum height of lateral face of mesoscutellum: 0.2–0.3. Propodeum areola: completely defined by carinae, including transverse carina extending to spiracle. Propodeum background sculpture: mostly sculptured. Mediotergite 1 length/width at posterior margin: 2.9–3.1. Mediotergite 1 shape: more or less parallel–sided. Mediotergite 1 sculpture: with some sculpture near lateral margins and/or posterior 0.2–0.4 of mediotergite. Mediotergite 2 width at posterior margin/length: 4.0–4.3. Mediotergite 2 sculpture: mostly smooth. Outer margin of hypopygium: with a wide, medially folded, transparent, semi–desclerotized area; usually with 4 or more pleats. Ovipositor thickness: about same width throughout its length. Ovipositor sheaths length/metatibial length: 0.6–0.7 or 0.8–0.9. Length of fore wing veins r/2RS: 1.1–1.3. Length of fore wing veins 2RS/2M: 1.4–1.6. Length of fore wing veins 2M/(RS+M)b: 0.7–0.8. Pterostigma length/width: 3.1–3.5. Point of insertion of vein r in pterostigma: about half way point length of pterostigma. Angle of vein r with fore wing anterior margin: clearly outwards, inclined towards fore wing apex. Shape of junction of veins r and 2RS in fore wing: distinctly but not strongly angled.

**Male.** As in female, but T2 is brown (female has T2 orange-yellow).

#### Molecular data.

Sequences in BOLD: 22, barcode compliant sequences: 22.

#### Biology/ecology.

Gregarious (Fig. 271). Host: Crambidae, *Ategumia* lotanalisDHJ07.

#### Distribution.

Costa Rica, ACG.

#### Etymology.

We dedicate this species to Edith López in recognition of her diligent efforts for the ACG Programa de Mantenimiento.

### Apanteles eduardoramirezi

Fernández-Triana sp. n.

http://zoobank.org/AED41110-AE94-4E87-8B36-D96E8C95316B

http://species-id.net/wiki/Apanteles_eduardoramirezi

Fig. 111

#### Type locality.

COSTA RICA, Guanacaste. ACG, Sector Santa Rosa, Bosque San Emilio, 300m, 10.84389, -85.61384.

#### Holotype.

♀ in CNC. Specimen labels: 1. DHJPAR0013080. 2. 15-May-2000, San Emilio Trap 1.

#### Paratypes.

14 ♀, 1 ♂ (BMNH, CNC, INBIO, INHS, NMNH). COSTA RICA, ACG database codes: DHJPAR0012527, DHJPAR0013024, DHJPAR0013029, DHJPAR0013032, DHJPAR0013041, DHJPAR0013045, DHJPAR0013046, DHJPAR0013048, DHJPAR0013052, DHJPAR0013059, DHJPAR0013060, DHJPAR0013074, DHJPAR0013085, DHJPAR0013091, DHJPAR0024699.

#### Description.

**Female.** Body color: body mostly dark except for some sternites which may be pale. Antenna color: scape, pedicel, and flagellum dark. Coxae color (pro-, meso-, metacoxa): pale, dark, dark. Femora color (pro-, meso-, metafemur): pale, anteriorly dark/posteriorly pale, dark. Tibiae color (pro-, meso-, metatibia): pale, pale, anteriorly pale/posteriorly dark. Tegula and humeral complex color: both pale. Pterostigma color: mostly pale and/or transparent, with thin dark borders. Fore wing veins color: partially pigmented (a few veins may be dark but most are pale). Antenna length/body length: antenna about as long as body (head to apex of metasoma); if slightly shorter, at least extending beyond anterior 0.7 metasoma length. Body in lateral view: not distinctly flattened dorso–ventrally. Body length (head to apex of metasoma): 2.3–2.4 mm, 2.5–2.6 mm, rarely 2.1–2.2 mm. Fore wing length: 2.1–2.2 mm, 2.3–2.4 mm or 2.5–2.6 mm. Ocular–ocellar line/posterior ocellus diameter: 2.3–2.5. Interocellar distance/posterior ocellus diameter: 2.0–2.2. Antennal flagellomerus 2 length/width: 2.6–2.8. Antennal flagellomerus 14 length/width: 1.1–1.3. Length of flagellomerus 2/length of flagellomerus 14: 2.3–2.5. Tarsal claws: with single basal spine–like seta. Metafemur length/width: 3.2–3.3. Metatibia inner spur length/metabasitarsus length: 0.4–0.5. Anteromesoscutum: mostly with deep, dense punctures (separated by less than 2.0 × its maximum diameter). Mesoscutellar disc: with punctures near margins, central part mostly smooth. Number of pits in scutoscutellar sulcus: 11 or 12. Maximum height of mesoscutellum lunules/maximum height of lateral face of mesoscutellum: 0.6–0.7. Propodeum areola: completely defined by carinae, including transverse carina extending to spiracle. Propodeum background sculpture: partly sculptured, especially on anterior 0.5. Mediotergite 1 length/width at posterior margin: 2.3–2.5. Mediotergite 1 shape: mostly parallel–sided for 0.5–0.7 of its length, then narrowing posteriorly so mediotergite anterior width >1.1 × posterior width. Mediotergite 1 sculpture: mostly sculptured, excavated area centrally with transverse striation inside and/or a polished knob centrally on posterior margin of mediotergite. Mediotergite 2 width at posterior margin/length: 4.0–4.3. Mediotergite 2 sculpture: with some sculpture, mostly near posterior margin. Outer margin of hypopygium: with a wide, medially folded, transparent, semi–desclerotized area; usually with 4 or more pleats. Ovipositor thickness: about same width throughout its length. Ovipositor sheaths length/metatibial length: 1.2–1.3, rarely 1.0–1.1. Length of fore wing veins r/2RS: 1.4–1.6. Length of fore wing veins 2RS/2M: 1.4–1.6. Length of fore wing veins 2M/(RS+M)b: 0.7–0.8. Pterostigma length/width: 2.6–3.0. Point of insertion of vein r in pterostigma: about half way point length of pterostigma. Angle of vein r with fore wing anterior margin: clearly outwards, inclined towards fore wing apex. Shape of junction of veins r and 2RS in fore wing: distinctly but not strongly angled.

**Male.** As in female but with narrower mediotergite 1.

#### Molecular data.

Sequences in BOLD: 99, barcode compliant sequences: 94.

#### Biology/ecology.

Malaise-trapped.

#### Distribution.

Costa Rica, ACG.

#### Etymology.

We dedicate this species to Eduardo Ramírez in recognition of his diligent efforts for ACG acquisitioning (Proveedor).

### Apanteles edwinapui

Fernández-Triana sp. n.

http://zoobank.org/8C59A4B0-026B-4EE8-B640-11ADA0208FDD

http://species-id.net/wiki/Apanteles_edwinapui

Figs 54 , 247

#### Type locality.

COSTA RICA, Guanacaste, ACG, Sector Cacao, Estación Gongora, 570m, 10.88700, -85.47443.

#### Holotype.

♀ in CNC. Specimen labels: 1. DHJPAR0005342. 2. COSTA RICA, Guanacaste, ACG, Sector Cacao, Estación Gongora Site, 9.viii.1995, 10.88700 N, -85.47443 W, 570m, DHJPAR0005342.

#### Paratypes.

18 ♀, 5 ♂ (BMNH, CNC, INBIO, INHS, NMNH). COSTA RICA: ACG database codes:, DHJPAR0020609.

#### Description.

**Female.** Body color: body mostly dark except for some sternites which may be pale. Antenna color: scape, pedicel, and flagellum dark. Coxae color (pro-, meso-, metacoxa): dark, dark, dark or pale, dark, dark. Femora color (pro-, meso-, metafemur): pale, pale, mostly pale but posterior 0.2 or less dark. Tibiae color (pro-, meso-, metatibia): pale, pale, mostly pale but with posterior 0.2 or less dark. Tegula and humeral complex color: tegula pale, humeral complex half pale/half dark. Pterostigma color: mostly dark, with small pale area centrally. Fore wing veins color: mostly dark (a few veins may be unpigmented). Antenna length/body length: antenna shorter than body (head to apex of metasoma), not extending beyond anterior 0.7 metasoma length, rarely antenna about as long as body (head to apex of metasoma); if slightly shorter, at least extending beyond anterior 0.7 metasoma length. Body in lateral view: not distinctly flattened dorso–ventrally. Body length (head to apex of metasoma): 2.9–3.0 mm, 3.1–3.2 mm or 3.3–3.4 mm. Fore wing length: 3.1–3.2 mm, 3.3–3.4 mm or 3.5–3.6 mm. Ocular–ocellar line/posterior ocellus diameter: 2.0–2.2. Interocellar distance/posterior ocellus diameter: 1.4–1.6. Antennal flagellomerus 2 length/width: 2.9–3.1. Antennal flagellomerus 14 length/width: 1.4–1.6. Length of flagellomerus 2/length of flagellomerus 14: 2.0–2.2. Tarsal claws: with single basal spine–like seta. Metafemur length/width: 3.0–3.1. Metatibia inner spur length/metabasitarsus length: 0.4–0.5. Anteromesoscutum: mostly with deep, dense punctures (separated by less than 2.0 × its maximum diameter). Mesoscutellar disc: mostly punctured. Number of pits in scutoscutellar sulcus: 7 or 8. Maximum height of mesoscutellum lunules/maximum height of lateral face of mesoscutellum: 0.4–0.5. Propodeum areola: completely defined by carinae, including transverse carina extending to spiracle. Propodeum background sculpture: partly sculptured, especially on anterior 0.5. Mediotergite 1 length/width at posterior margin: 2.6–2.8. Mediotergite 1 shape: mostly parallel–sided for 0.5–0.7 of its length, then narrowing posteriorly so mediotergite anterior width >1.1 × posterior width. Mediotergite 1 sculpture: with some sculpture near lateral margins and/or posterior 0.2–0.4 of mediotergite. Mediotergite 2 width at posterior margin/length: 3.2–3.5. Mediotergite 2 sculpture: mostly smooth. Outer margin of hypopygium: inflexible (without a folded, transparent, semi–desclerotized area); with no pleats visible. Ovipositor thickness: anterior width 3.0–5.0 × posterior width (beyond ovipositor constriction). Ovipositor sheaths length/metatibial length: 0.6–0.7. Length of fore wing veins r/2RS: 2.3 or more. Length of fore wing veins 2RS/2M: 0.9–1.0. Length of fore wing veins 2M/(RS+M)b: 0.7–0.8. Pterostigma length/width: 3.1–3.5. Point of insertion of vein r in pterostigma: about half way point length of pterostigma, rarely clearly beyond half way point length of pterostigma. Angle of vein r with fore wing anterior margin: more or less perpendicular to fore wing margin. Shape of junction of veins r and 2RS in fore wing: distinctly but not strongly angled.

**Male.** As in female, but with darker coloration, especially on metafemur.

#### Molecular data.

Sequences in BOLD: 2, barcode compliant sequences: 2.

#### Biology/ecology.

Gregarious (Fig. 247). Hosts: Hesperiidae, *Astraptes inflatio*, *Astraptes fruticibus*.

#### Distribution.

Costa Rica, ACG.

#### Etymology.

We dedicate this species to Edwin Apu in recognition of his diligent efforts for the ACG Programa de Parataxónomos and Estación Biológica Leiva of ACG.

### Apanteles eldarayae

Fernández-Triana sp. n.

http://zoobank.org/FC2EE032-980A-4D5D-872A-D54C0C3A0393

http://species-id.net/wiki/Apanteles_eldarayae

Figs 55 , 248

Apanteles Rodriguez01 (Smith et al. 2006). Interim name provided by the authors.

#### Type locality.

COSTA RICA, Alajuela, ACG, Sector San Cristobal, Potrero Argentina, 520m, 10.89021, -85.38803.

#### Holotype.

♀ in CNC. Specimen labels: 1. COSTA RICA, Alajuela, ACG, San Cristobal: Potrero Argentina, 03/13/1999, Gloria Sihezar. 2. 99-SRNP-12461 (dried), Pyrrhopyge zenodorus, Vismia baccifera.

#### Paratypes.

53 ♀, 38 ♂ (BMNH, CNC, INBIO, INHS, NMNH). COSTA RICA, ACG database codes: See Appendix 2 for detailed label data.

#### Description.

**Female.** Body color: body mostly dark except for some sternites which may be pale. Antenna color: scape, pedicel, and flagellum dark. Coxae color (pro-, meso-, metacoxa): dark, dark, dark. Femora color (pro-, meso-, metafemur): pale, pale, pale, rarely pale, pale, mostly pale but posterior 0.2 or less dark. Tibiae color (pro-, meso-, metatibia): pale, pale, pale. Tegula and humeral complex color: both pale. Pterostigma color: mostly pale and/or transparent, with thin dark borders. Fore wing veins color: partially pigmented (a few veins may be dark but most are pale). Antenna length/body length: antenna about as long as body (head to apex of metasoma); if slightly shorter, at least extending beyond anterior 0.7 metasoma length. Body in lateral view: not distinctly flattened dorso–ventrally. Body length (head to apex of metasoma): 3.3–3.4 mm or 3.5–3.6 mm. Fore wing length: 3.3–3.4 mm or 3.5–3.6 mm. Ocular–ocellar line/posterior ocellus diameter: 2.3–2.5. Interocellar distance/posterior ocellus diameter: 2.0–2.2. Antennal flagellomerus 2 length/width: 2.3–2.5. Antennal flagellomerus 14 length/width: 2.3–2.5. Length of flagellomerus 2/length of flagellomerus 14: 1.4–1.6. Tarsal claws: simple or with single basal spine–like seta. Metafemur length/width: 3.2–3.3. Metatibia inner spur length/metabasitarsus length: 0.4–0.5. Anteromesoscutum: mostly with deep, dense punctures (separated by less than 2.0 × its maximum diameter). Mesoscutellar disc: mostly punctured. Number of pits in scutoscutellar sulcus: 7 or 8. Maximum height of mesoscutellum lunules/maximum height of lateral face of mesoscutellum: 0.4–0.5. Propodeum areola: completely defined by carinae, including transverse carina extending to spiracle. Propodeum background sculpture: mostly sculptured. Mediotergite 1 length/width at posterior margin: 4.1 or more. Mediotergite 1 shape: slightly widening from anterior margin to 0.7–0.8 mediotergite length (where maximum width is reached), then narrowing towards posterior margin. Mediotergite 1 sculpture: with some sculpture near lateral margins and/or posterior 0.2–0.4 of mediotergite. Mediotergite 2 width at posterior margin/length: 3.2–3.5. Mediotergite 2 sculpture: with some sculpture, mostly near posterior margin. Outer margin of hypopygium: with a medially folded, transparent, semi–desclerotized area; with 0–3 pleats visible. Ovipositor thickness: anterior width 3.0–5.0 × posterior width (beyond ovipositor constriction). Ovipositor sheaths length/metatibial length: 1.0–1.1, rarely 1.2–1.3. Length of fore wing veins r/2RS: 2.3 or more. Length of fore wing veins 2RS/2M: 1.7–1.8. Length of fore wing veins 2M/(RS+M)b: 0.5–0.6. Pterostigma length/width: 3.6 or more. Point of insertion of vein r in pterostigma: clearly beyond half way point length of pterostigma. Angle of vein r with fore wing anterior margin: clearly inwards, inclined towards fore wing base. Shape of junction of veins r and 2RS in fore wing: strongly angulated, sometimes with a knob.

**Male.** Similar to female.

#### Molecular data.

Sequences in BOLD: 27, barcode compliant sequences: 15.

#### Biology/ecology.

Gregarious (Fig. 248). Host: Hesperiidae, *Pyrrhopyge zenodorus*.

#### Distribution.

Costa Rica, ACG.

#### Etymology.

We dedicate this species to Elda Araya in recognition of her diligent efforts for the ACG Programa de Parataxónomos and Estación Biológica San Gerardo of ACG.

### Apanteles eliethcantillanoae

Fernández-Triana sp. n.

http://zoobank.org/B2352F1A-6D93-4663-82A5-8F47FF3AF303

http://species-id.net/wiki/Apanteles_eliethcantillanoae

Figs 172 , 310

Apanteles Rodriguez87 (Smith et al. 2006). Interim name provided by the authors.

#### Type locality.

COSTA RICA, Guanacaste, ACG, Sector El Hacha, Finca Araya, 295m, 11.01541, -85.51125.

#### Holotype.

♀ in CNC. Specimen labels: 1. DHJPAR0002687. 2. COSTA RICA, Guanacaste, ACG, Sector El Hacha, Finca Araya, 23.vii.2002, 11.01541°N, 85.51125°W, 295m, DHJPAR0002687.

#### Paratypes.

40 ♀, 10 ♂ (BMNH, CNC, INBIO, INHS, NMNH). COSTA RICA, ACG database codes: DHJPAR0002202, DHJPAR0002687, DHJPAR0005288, DHJPAR0005317, DHJPAR0011953.

#### Description.

**Female.** Metatibia color (outer face): entirely or mostly (>0.7 metatibia length) dark brown to black, with yellow to white coloration usually restricted to anterior 0.2 or less, rarely with extended pale coloration (light yellow to orange–yellow), ranging from 0.4 to almost entire metatibia length. Fore wing veins color: veins C+Sc+R and R1 with brown coloration restricted narrowly to borders, interior area of those veins and pterostigma (and sometimes veins r, 2RS and 2M) transparent or white; other veins mostly transparent. Antenna length/body length: antenna shorter than body (head to apex of metasoma), not extending beyond anterior 0.7 metasoma length. Body length (head to apex of metasoma): 2.3–2.4 mm or 2.5–2.6 mm. Fore wing length: 2.3–2.4 mm or 2.5–2.6 mm. Metafemur length/width: 2.8–2.9 or 3.0–3.1. Mediotergite 1 length/width at posterior margin: 2.9 or more. Mediotergite 1 maximum width/width at posterior margin: 1.8–1.9. Ovipositor sheaths length/metafemur length: 1.0. Ovipositor sheaths length/metatibia length: 0.8 or 0.9.

#### Molecular data.

Sequences in BOLD: 7, barcode compliant sequences: 1.

#### Biology/ecology.

Gregarious (Fig. 310). Hosts: Hesperiidae, *Urbanus doryssus* DHJ02.

#### Distribution.

Costa Rica, ACG.

#### Comments.

A total of 21 ♀ and 5 ♂ (ACG codes DHJPAR0004612 and DHJPAR0004619) are named as *Apanteles eliethcantillanoae*, but are likely to be another species; they are reared from the same host as the other specimens of this species, but from a different place in ACG (Estación La Perla, Sector Mundo Nuevo). Both failed sequencing. They are excluded from the paratype series and will be revisted later.

#### Etymology.

We dedicate this species to Elieth Cantillano in recognition of her diligent efforts for the ACG Programa de Parataxnomos and Estación Biológica Los Almendros in Sector Los Almendros and Sector Del Oro.

### Apanteles erickduartei

Fernández-Triana sp. n.

http://zoobank.org/A2405234-0B5C-4427-BC53-FF246BCF94DC

http://species-id.net/wiki/Apanteles_erickduartei

Figs 113 , 279

Apanteles Rodriguez84 (Smith et al. 2006). Interim name provided by the authors.

#### Type locality.

COSTA RICA, Alajuela, ACG, Sector San Cristobal, Sendero Huerta, 527m, 10.9305, -85.37223.

#### Holotype.

♀ in CNC. Specimen labels: 1. Voucher: D.H.Janzen & W.Hallwachs, DB: http://janzen.sas.upenn.edu, Area de Conservación Guanacaste, COSTA RICA, 09-SRNP-2303. 2. DHJPAR0035478.

#### Paratypes.

3 ♀, 3 ♂ (CNC, NMNH). COSTA RICA, ACG database codes: DHJPAR0035256, DHJPAR0035376, DHJPAR0035368, DHJPAR0035481, DHJPAR0035508, DHJPAR0038233.

#### Description.

**Female.** Body color: head dark, mesosoma dark with parts of axillar complex pale, metasoma with some mediotergites, most laterotergites, sternites, and/or hypopygium pale. Antenna color: scape, pedicel, and flagellum dark or scape and/or pedicel pale, flagellum dark. Coxae color (pro-, meso-, metacoxa): pale, pale, dark or pale, pale, pale. Femora color (pro-, meso-, metafemur): pale, pale, anteriorly pale/posteriorly dark, pale, pale, pale or pale, pale, mostly pale but posterior 0.2 or less dark. Tibiae color (pro-, meso-, metatibia): pale, pale, anteriorly pale/posteriorly dark or pale, pale, mostly pale but with posterior 0.2 or less dark. Tegula and humeral complex color: both pale. Pterostigma color: dark. Fore wing veins color: mostly dark (a few veins may be unpigmented). Antenna length/body length: antenna about as long as body (head to apex of metasoma); if slightly shorter, at least extending beyond anterior 0.7 metasoma length. Body in lateral view: not distinctly flattened dorso–ventrally. Body length (head to apex of metasoma): 3.3–3.4 mm, rarely 3.7–3.8 mm. Fore wing length: 3.3–3.4 mm, 3.5–3.6 mm, rarely 3.7–3.8 mm. Ocular–ocellar line/posterior ocellus diameter: 1.7–1.9. Interocellar distance/posterior ocellus diameter: 1.7–1.9. Antennal flagellomerus 2 length/width: 2.6–2.8. Antennal flagellomerus 14 length/width: 1.4–1.6. Length of flagellomerus 2/length of flagellomerus 14: 2.6–2.8. Tarsal claws: with single basal spine–like seta. Metafemur length/width: 2.8–2.9. Metatibia inner spur length/metabasitarsus length: 0.4–0.5. Anteromesoscutum: mostly with deep, dense punctures (separated by less than 2.0 × its maximum diameter). Mesoscutellar disc: mostly punctured. Number of pits in scutoscutellar sulcus: 5 or 6 or 7 or 8. Maximum height of mesoscutellum lunules/maximum height of lateral face of mesoscutellum: 0.2–0.3. Propodeum areola: completely defined by carinae, including transverse carina extending to spiracle. Propodeum background sculpture: mostly sculptured. Mediotergite 1 length/width at posterior margin: 2.3–2.5 or 2.6–2.8. Mediotergite 1 shape: more or less parallel–sided or mostly parallel–sided for 0.5–0.7 of its length, then narrowing posteriorly so mediotergite anterior width >1.1 × posterior width. Mediotergite 1 sculpture: mostly sculptured, excavated area centrally with transverse striation inside and/or a polished knob centrally on posterior margin of mediotergite. Mediotergite 2 width at posterior margin/length: 2.8–3.1. Mediotergite 2 sculpture: mostly smooth. Outer margin of hypopygium: with a wide, medially folded, transparent, semi–desclerotized area; usually with 4 or more pleats. Ovipositor thickness: anterior width at most 2.0 × posterior width (beyond ovipositor constriction). Ovipositor sheaths length/metatibial length: 1.0–1.1, rarely 1.2–1.3. Length of fore wing veins r/2RS: 1.7–1.9. Length of fore wing veins 2RS/2M: 1.4–1.6. Length of fore wing veins 2M/(RS+M)b: 0.7–0.8. Pterostigma length/width: 3.1–3.5. Point of insertion of vein r in pterostigma: about half way point length of pterostigma. Angle of vein r with fore wing anterior margin: clearly outwards, inclined towards fore wing apex. Shape of junction of veins r and 2RS in fore wing: strongly angulated, sometimes with a knob.

**Male.** Coloration (especially legs) tends to be darker than females, and mediotergite 2 is less transverse, i.e., more quadrate.

#### Molecular data.

Sequences in BOLD: 9, barcode compliant sequences: 7.

#### Biology/ecology.

Solitary (Fig. 279). Host: Crambidae, *Asturodes* fimbriauralisDHJ01, *Asturodes* fimbriauralisDHJ02, *Piletosoma thialis*, *Spilomela discordens*, *Eulepte* Janzen03.

#### Distribution.

Costa Rica, ACG.

#### Etymology.

We dedicate this species to Erick Duarte in recognition of his diligent efforts for the ACG Programa de Transporte.

### Apanteles esthercentenoae

Fernández-Triana sp. n.

http://zoobank.org/04D6050B-1A9B-4504-8D6F-97F60C586642

http://species-id.net/wiki/Apanteles_esthercentenoae

Fig. 150

Apanteles Rodriguez105 (Smith et al. 2006). Interim name provided by the authors.

#### Type locality.

COSTA RICA, Guanacaste, ACG, Sector Santa Rosa, Area Administrativa, 295m, 10.83764, -85.61871.

#### Holotype.

♀ in CNC. Specimen labels: 1. DHJPAR0005275. 2. COSTA RICA, Guanacaste, ACG, Sector Santa Rosa, Area Administrativa, 12.vi.2000, Victor Chien. 3. 00-SRNP-8744, *Palpita venatalis* feeding on fallen flowers of *Stemmadenia obovata* (Apocynaceae).

#### Paratypes.

2 ♀, 4 ♂ (CNC, NMNH). COSTA RICA, ACG database codes: DHJPAR0005268, DHJPAR0005272-DHJPAR0005274, DHJPAR0012475, DHJPAR0012476.

#### Description.

**Female.** Body color: body mostly dark except for some sternites which may be pale. Antenna color: scape, pedicel, and flagellum dark. Coxae color (pro-, meso-, metacoxa): dark, dark, dark. Femora color (pro-, meso-, metafemur): pale, anteriorly dark/posteriorly pale, dark. Tibiae color (pro-, meso-, metatibia): pale, pale, anteriorly pale/posteriorly dark. Tegula and humeral complex color: tegula pale, humeral complex half pale/half dark. Pterostigma color: mostly pale and/or transparent, with thin dark borders. Fore wing veins color: mostly white or entirely transparent. Antenna length/body length: antenna about as long as body (head to apex of metasoma); if slightly shorter, at least extending beyond anterior 0.7 metasoma length. Body in lateral view: not distinctly flattened dorso–ventrally. Body length (head to apex of metasoma): 3.5–3.6 mm or 3.7–3.8 mm. Fore wing length: 3.9–4.0 mm. Ocular–ocellar line/posterior ocellus diameter: 2.0–2.2. Interocellar distance/posterior ocellus diameter: 1.4–1.6. Antennal flagellomerus 2 length/width: 2.6–2.8. Antennal flagellomerus 14 length/width: 1.4–1.6. Length of flagellomerus 2/length of flagellomerus 14: 2.0–2.2. Tarsal claws: with single basal spine–like seta. Metafemur length/width: 3.0–3.1. Metatibia inner spur length/metabasitarsus length: 0.4–0.5. Anteromesoscutum: mostly with shallow, dense punctures (separated by less than 2.0 × its maximum diameter). Mesoscutellar disc: mostly smooth. Number of pits in scutoscutellar sulcus: 11 or 12. Maximum height of mesoscutellum lunules/maximum height of lateral face of mesoscutellum: 0.8 or more. Propodeum areola: completely defined by carinae, including transverse carina extending to spiracle. Propodeum background sculpture: mostly sculptured. Mediotergite 1 length/width at posterior margin: 1.1–1.3. Mediotergite 1 shape: clearly widening towards posterior margin. Mediotergite 1 sculpture: mostly sculptured, excavated area centrally with transverse striation inside and/or a polished knob centrally on posterior margin of mediotergite. Mediotergite 2 width at posterior margin/length: 3.6–3.9. Mediotergite 2 sculpture: with some sculpture, mostly near posterior margin. Outer margin of hypopygium: with a wide, medially folded, transparent, semi–desclerotized area; usually with 4 or more pleats. Ovipositor thickness: about same width throughout its length. Ovipositor sheaths length/metatibial length: 1.2–1.3. Length of fore wing veins r/2RS: 2.0–2.2 or 2.3 or more. Length of fore wing veins 2RS/2M: 1.7–1.8. Length of fore wing veins 2M/(RS+M)b: 0.5–0.6. Pterostigma length/width: 3.1–3.5. Point of insertion of vein r in pterostigma: about half way point length of pterostigma. Angle of vein r with fore wing anterior margin: clearly outwards, inclined towards fore wing apex. Shape of junction of veins r and 2RS in fore wing: distinctly but not strongly angled.

**Male.** Mediotergite 2 tends to be less transverse, and mediotergite 1 is rather parallel-sided.

#### Molecular data.

Sequences in BOLD: 8, barcode compliant sequences: 8.

#### Biology/ecology.

Solitary. Hosts: Crambidae, *Palpita venatalis*, Pyralidae, *Cromarcha stroudagnesia*.

#### Distribution.

Costa Rica, ACG.

#### Comments.

This species was reared from *Palpita venatalis* inside of fallen *Stemmadenia obovata* (Apocynaceae) flowers, and from *Cromarcha stroudagnesia* mining in *Tabebuia ochracea* (Bignoniaceae) stems. No evidence suggests that the specimens are different species, thus they are kept as one here. Based on morphology alone, *Apanteles esthercentenoae* is very similar to *Apanteles thurberiae* (see comments under that species).

#### Etymology.

We dedicate this species to Esther Centeno in recognition of her diligent efforts for the ACG Programa de Ecoturismo.

### Apanteles eugeniaphilipsae

Fernández-Triana sp. n.

http://zoobank.org/0B0AEF1A-11FC-4015-AD9C-5260890A2653

http://species-id.net/wiki/Apanteles_eugeniaphilipsae

Fig. 178

Apanteles Rodriguez22 (Smith et al. 2006). Interim name provided by the authors.

#### Type locality.

COSTA RICA, Alajuela, ACG, Sector Rincon Rain Forest, San Lucas, 320m, 10.91847, -85.30338.

#### Holotype.

♀ in CNC. Specimen labels: 1. DHJPAR0001592. 2. COSTA RICA, Alajuela, ACG, Sector Rincon Rain Forest, San Lucas, 23.ii.2004, 320m, 10.91847, -85.30338, 04-SRNP-40552.

#### Paratype.

1 ♀, 1 ♂ (CNC). COSTA RICA: Guanacaste, ACG database codes: DHJPAR0012272, 04-SRNP-40552.

#### Description.

**Female.** Metatibia color (outer face): entirely or mostly (>0.7 metatibia length) dark brown to black, with yellow to white coloration usually restricted to anterior 0.2 or less. Fore wing veins color: veins C+Sc+R and R1 with brown coloration restricted narrowly to borders, interior area of those veins and pterostigma (and sometimes veins r, 2RS and 2M) transparent or white; other veins mostly transparent. Antenna length/body length: antenna about as long as body (head to apex of metasoma); if slightly shorter, at least extending beyond anterior 0.7 metasoma length. Body length (head to apex of metasoma): 2.3–2.4 mm. Fore wing length: 2.5–2.6 mm. Metafemur length/width: 2.6–2.7. Mediotergite 1 length/width at posterior margin: 2.3–2.4. Mediotergite 1 maximum width/width at posterior margin: 1.6–1.7. Ovipositor sheaths length/metafemur length: 1.1. Ovipositor sheaths length/metatibia length: 0.9.

#### Molecular data.

Sequences in BOLD: 2, barcode compliant sequences: 1.

#### Biology/ecology.

Gregarious. Host: Hesperiidae, *Narcosius samson*.

#### Distribution.

Costa Rica, ACG.

#### Etymology.

We dedicate this species to Eugenia Philips in recognition of her diligent efforts for the ACG Programa de Parataxónomos, and Lepidoptera curatorial taxonomy for INBio, Costa Rica’s Instituto Nacional de Biodiversidad, and for ACG.

### Apanteles eulogiosequeirai

Fernández-Triana sp. n.

http://zoobank.org/227D78D4-7E77-42CB-9306-4378136C916C

http://species-id.net/wiki/Apanteles_eulogiosequeirai

Figs 40 , 236

#### Type locality.

COSTA RICA, Guanacaste, ACG, Sector Rincon Rain Forest, Conguera, 420m, 10.91589, -85.26631.

#### Holotype.

♀ in CNC. Specimen labels: 1. DHJPAR0045279. 2. COSTA RICA, Guanacaste, ACG, Sector Rincon Rain Forest, Conguera, 11.vii.2011, 10.91589N, 85.26631W, 420m, DHJPAR0045279. Voucher: D.H.Janzen & W.Hallwachs, DB: http://janzen.sas.upenn.edu, Area de Conservación Guanacaste, COSTA RICA, 11-SRNP-43309.

#### Description.

**Female.** Body color: body mostly dark except for some sternites which may be pale. Antenna color: scape, pedicel, and flagellum dark. Coxae color (pro-, meso-, metacoxa): dark, dark, dark. Femora color (pro-, meso-, metafemur): anteriorly dark/posteriorly pale, dark, dark. Tibiae color (pro-, meso-, metatibia): pale, pale, mostly pale but with posterior 0.2 or less dark. Tegula and humeral complex color: tegula pale, humeral complex half pale/half dark. Pterostigma color: mostly pale and/or transparent, with thin dark borders. Fore wing veins color: mostly white or entirely transparent. Antenna length/body length: antenna about as long as body (head to apex of metasoma); if slightly shorter, at least extending beyond anterior 0.7 metasoma length. Body in lateral view: not distinctly flattened dorso–ventrally. Body length (head to apex of metasoma): 3.5–3.6 mm. Fore wing length: 3.5–3.6 mm. Ocular–ocellar line/posterior ocellus diameter: 2.3–2.5. Interocellar distance/posterior ocellus diameter: 1.7–1.9. Antennal flagellomerus 2 length/width: 2.3–2.5. Antennal flagellomerus 14 length/width: 2.0–2.2. Length of flagellomerus 2/length of flagellomerus 14: 1.7–1.9. Tarsal claws: with single basal spine–like seta. Metafemur length/width: 3.2–3.3. Metatibia inner spur length/metabasitarsus length: 0.4–0.5. Anteromesoscutum: mostly with deep, dense punctures (separated by less than 2.0 × its maximum diameter). Mesoscutellar disc: mostly smooth. Number of pits in scutoscutellar sulcus: 7 or 8 or 9 or 10. Maximum height of mesoscutellum lunules/maximum height of lateral face of mesoscutellum: 0.6–0.7. Propodeum areola: completely defined by carinae, including transverse carina extending to spiracle. Propodeum background sculpture: mostly sculptured. Mediotergite 1 length/width at posterior margin: 2.9–3.1. Mediotergite 1 shape: mostly parallel–sided for 0.5–0.7 of its length, then narrowing posteriorly so mediotergite anterior width >1.1 × posterior width. Mediotergite 1 sculpture: mostly sculptured, excavated area centrally with transverse striation inside and/or a polished knob centrally on posterior margin of mediotergite. Mediotergite 2 width at posterior margin/length: 1.6–1.9. Mediotergite 2 sculpture: mostly smooth. Outer margin of hypopygium: with a wide, medially folded, transparent, semi–desclerotized area; usually with 4 or more pleats. Ovipositor thickness: about same width throughout its length. Ovipositor sheaths length/metatibial length: 1.8–1.9. Length of fore wing veins r/2RS: 2.3 or more. Length of fore wing veins 2RS/2M: 1.4–1.6. Length of fore wing veins 2M/(RS+M)b: 0.5–0.6. Pterostigma length/width: 3.1–3.5. Point of insertion of vein r in pterostigma: about half way point length of pterostigma. Angle of vein r with fore wing anterior margin: clearly outwards, inclined towards fore wing apex. Shape of junction of veins r and 2RS in fore wing: distinctly but not strongly angled.

**Male.** Unknown.

#### Molecular data.

Sequences in BOLD: 6, barcode compliant sequences: 6.

#### Biology/ecology.

Solitary (Fig. 236). Hosts: Elachistidae, *Stenoma* Janzen08.

#### Distribution.

Costa Rica, ACG.

#### Etymology.

We dedicate this species to Eulogio Sequeira in recognition of his diligent efforts for the ACG Sector Marino.

### Apanteles federicomatarritai

Fernández-Triana sp. n.

http://zoobank.org/6F24D2BE-D3E2-4B03-8047-0B30859F0318

http://species-id.net/wiki/Apanteles_federicomatarritai

Figs 168 , 327

Apanteles Rodriguez41 (Smith et al. 2006). Interim name provided by the authors.

#### Type locality.

COSTA RICA, Guanacaste, ACG, Sector Mundo Nuevo, Mamones, 365m, 10.77074, -85.42874.

#### Holotype.

♀ in CNC. Specimen labels: 1. DHJPAR0020615. 2. COSTA RICA, Guanacaste, ACG, Sector Mundo Nuevo, Mamones, 24.xii.2007, 10.77074°N, 85.42874°W, 365m, DHJPAR0020615.

#### Paratypes.

151 ♀, 139 ♂ (BMNH, CNC, INBIO, INHS, NMNH). COSTA RICA, ACG database codes: See Appendix 2 for detailed label data.

#### Description.

**Female.** Metatibia color (outer face): entirely or mostly (>0.7 metatibia length) dark brown to black, with yellow to white coloration usually restricted to anterior 0.2 or less. Fore wing veins color: veins C+Sc+R and R1 with brown coloration restricted narrowly to borders, interior area of those veins and pterostigma (and sometimes veins r, 2RS and 2M) transparent or white; other veins mostly transparent. Antenna length/body length: antenna shorter than body (head to apex of metasoma), not extending beyond anterior 0.7 metasoma length. Body length (head to apex of metasoma): 2.1–2.2 mm or 2.3–2.4 mm. Fore wing length: 2.3–2.4 mm or 2.5–2.6 mm. Metafemur length/width: 2.8–2.9. Mediotergite 1 length/width at posterior margin: 2.3–2.4. Mediotergite 1 maximum width/width at posterior margin: 1.4–1.5. Ovipositor sheaths length/metafemur length: 0.9. Ovipositor sheaths length/metatibia length: 0.8.

#### Molecular data.

Sequences in BOLD: 38, barcode compliant sequences: 30.

#### Biology/ecology.

Gregarious (Fig. 327). Hosts: Hesperiidae, *Chioides zilpa*, *Polygonus leo*.

#### Distribution.

Costa Rica, ACG.

#### Etymology.

We dedicate this species to Federico Matarrita in recognition of his diligent efforts to rebuild and develop the ACG web site at http://www.acguanacaste.ac.cr and guide the parataxonomists into displaying their Species Pages there.

### Apanteles felipechavarriai

Fernández-Triana sp. n.

http://zoobank.org/4E1C0812-B109-43A9-A77F-27EF92E5B4A9

http://species-id.net/wiki/Apanteles_felipechavarriai

Figs 26 , 228

Apanteles Rodriguez108 (Smith et al. 2006). Interim name provided by the authors.

#### Type locality.

COSTA RICA, Alajuela, ACG, Sector Rincon Rain Forest, Finca Esmeralda, 123m, 10.93548, -85.25314.

#### Holotype.

♀ in CNC. Specimen labels: 1. DHJPAR0039769. 2. COSTA RICA, Alajuela, ACG, Sector Rincon Rain Forest, Finca Esmeralda, 23.ix.2009, 10.93548, -85.25314, 123m, DHJPAR0039769. 3. Voucher: D.H.Janzen & W.Hallwachs, DB: http://janzen.sas.upenn.edu, Area de Conservación Guanacaste, COSTA RICA, 09-SRNP-75876.

#### Description.

**Female.** Body color: body mostly dark except for some sternites which may be pale. Antenna color: scape and/or pedicel dark, flagellum pale (?). Coxae color (pro-, meso-, metacoxa): dark, dark, dark (?). Femora color (pro-, meso-, metafemur): pale, dark, dark (?). Tegula and humeral complex color: tegula pale, humeral complex half pale/half dark. Pterostigma color: mostly pale and/or transparent, with thin dark borders. Fore wing veins color: mostly white or entirely transparent. Antenna length/body length: antenna shorter than body (head to apex of metasoma), not extending beyond anterior 0.7 metasoma length. Body in lateral view: not distinctly flattened dorso–ventrally. Body length (head to apex of metasoma): 3.1–3.2 mm. Fore wing length: 3.1–3.2 mm. Ocular–ocellar line/posterior ocellus diameter: 1.7–1.9. Interocellar distance/posterior ocellus diameter: 2.0–2.2. Antennal flagellomerus 2 length/width: 2.0–2.2. Antennal flagellomerus 14 length/width: 1.1–1.3. Length of flagellomerus 2/length of flagellomerus 14: 2.0–2.2. Tarsal claws: simple (?). Metafemur length/width: 2.8–2.9. Anteromesoscutum: mostly with shallow, dense punctures (separated by less than 2.0 × its maximum diameter). Mesoscutellar disc: mostly smooth. Number of pits in scutoscutellar sulcus: 7 or 8. Maximum height of mesoscutellum lunules/maximum height of lateral face of mesoscutellum: 0.6–0.7. Propodeum areola: completely defined by carinae, including transverse carina extending to spiracle. Propodeum background sculpture: partly sculptured, especially on anterior 0.5. Mediotergite 1 length/width at posterior margin: 2.0–2.2. Mediotergite 1 shape: mostly parallel–sided for 0.5–0.7 of its length, then narrowing posteriorly so mediotergite anterior width >1.1 × posterior width. Mediotergite 1 sculpture: mostly sculptured, excavated area centrally with transverse striation inside and/or a polished knob centrally on posterior margin of mediotergite. Mediotergite 2 width at posterior margin/length: 3.6–3.9. Mediotergite 2 sculpture: mostly smooth. Outer margin of hypopygium: with a wide, medially folded, transparent, semi–desclerotized area; usually with 4 or more pleats. Ovipositor thickness: anterior width at most 2.0 × posterior width (beyond ovipositor constriction). Length of fore wing veins r/2RS: 2.3 or more. Length of fore wing veins 2RS/2M: 1.1–1.3. Length of fore wing veins 2M/(RS+M)b: 0.5–0.6. Pterostigma length/width: 3.1–3.5. Point of insertion of vein r in pterostigma: clearly beyond half way point length of pterostigma. Angle of vein r with fore wing anterior margin: clearly outwards, inclined towards fore wing apex. Shape of junction of veins r and 2RS in fore wing: distinctly but not strongly angled.

**Male.** Unknown.

#### Molecular data.

Sequences in BOLD: 1, barcode compliant sequences: 1.

#### Biology/ecology.

Solitary (Fig. 228). Host: Crambidae, *Eulepte concordalis*.

#### Distribution.

Costa Rica, ACG.

#### Comments.

The only know specimen is in rather poor condition, bleached (i.e., decolored) and missing most of the legs. Because of that, the description is incomplete, and it is impossible to key it out based on morphological characters only.

#### Etymology.

We dedicate this species to Felipe Chavarría in recognition of his diligent efforts for the ACG Programa de Parataxónomos and administrating project accounting.

### Apanteles felixcarmonai

Fernández-Triana sp. n.

http://zoobank.org/658550EB-8F80-45E3-B2B3-0D5285654976

http://species-id.net/wiki/Apanteles_felixcarmonai

Figs 114 , 280

Apanteles Rodriguez85 (Smith et al. 2006). Interim name provided by the authors.

#### Type locality.

COSTA RICA, Guanacaste, ACG, Sector Pitilla, Sendero Naciente, 700m, 10.98705, -85.42816.

#### Holotype.

♀ in CNC. Specimen labels: 1. Voucher: D.H.Janzen & W.Hallwachs, DB: http://janzen.sas.upenn.edu, Area de Conservación Guanacaste, COSTA RICA, 10-SRNP-30442. 2. DHJPAR0039049.

#### Paratypes.

2 ♀ (CNC, NMNH). COSTA RICA, ACG database codes: DHJPAR0026992, DHJPAR0039055.

#### Description.

**Female.** Body color: head dark, mesosoma dark with parts of axillar complex pale, metasoma with some mediotergites, most laterotergites, sternites, and/or hypopygium pale. Antenna color: scape, pedicel, and flagellum dark. Coxae color (pro-, meso-, metacoxa): pale, pale, pale. Femora color (pro-, meso-, metafemur): pale, pale, anteriorly pale/posteriorly dark. Tibiae color (pro-, meso-, metatibia): pale, pale, anteriorly pale/posteriorly dark. Tegula and humeral complex color: both pale. Pterostigma color: dark. Fore wing veins color: mostly dark (a few veins may be unpigmented). Antenna length/body length: antenna about as long as body (head to apex of metasoma); if slightly shorter, at least extending beyond anterior 0.7 metasoma length. Body in lateral view: not distinctly flattened dorso–ventrally. Body length (head to apex of metasoma): 3.5–3.6 mm or 3.7–3.8 mm. Fore wing length: 3.5–3.6 mm, 3.7–3.8 mm or 3.9–4.0 mm. Ocular–ocellar line/posterior ocellus diameter: 2.0–2.2. Interocellar distance/posterior ocellus diameter: 1.7–1.9. Antennal flagellomerus 2 length/width: 2.9–3.1. Antennal flagellomerus 14 length/width: 1.4–1.6. Length of flagellomerus 2/length of flagellomerus 14: 2.3–2.5. Tarsal claws: with single basal spine–like seta. Metafemur length/width: 2.8–2.9. Metatibia inner spur length/metabasitarsus length: 0.4–0.5. Anteromesoscutum: mostly with deep, dense punctures (separated by less than 2.0 × its maximum diameter). Mesoscutellar disc: mostly punctured. Number of pits in scutoscutellar sulcus: 5 or 6. Maximum height of mesoscutellum lunules/maximum height of lateral face of mesoscutellum: 0.2–0.3. Propodeum areola: completely defined by carinae, including transverse carina extending to spiracle. Propodeum background sculpture: mostly sculptured. Mediotergite 1 length/width at posterior margin: 3.2–3.4. Mediotergite 1 shape: mostly parallel–sided for 0.5–0.7 of its length, then narrowing posteriorly so mediotergite anterior width >1.1 × posterior width. Mediotergite 1 sculpture: with some sculpture near lateral margins and/or posterior 0.2–0.4 of mediotergite. Mediotergite 2 width at posterior margin/length: 3.2–3.5. Mediotergite 2 sculpture: mostly smooth. Outer margin of hypopygium: with a wide, medially folded, transparent, semi–desclerotized area; usually with 4 or more pleats. Ovipositor thickness: anterior width at most 2.0 × posterior width (beyond ovipositor constriction). Ovipositor sheaths length/metatibial length: 1.0–1.1. Length of fore wing veins r/2RS: 1.4–1.6. Length of fore wing veins 2RS/2M: 1.4–1.6. Length of fore wing veins 2M/(RS+M)b: 0.5–0.6. Pterostigma length/width: 3.1–3.5. Point of insertion of vein r in pterostigma: about half way point length of pterostigma. Angle of vein r with fore wing anterior margin: clearly outwards, inclined towards fore wing apex. Shape of junction of veins r and 2RS in fore wing: distinctly but not strongly angled.

**Male.** Unknown.

#### Molecular data.

Sequences in BOLD: 8, barcode compliant sequences: 8.

#### Biology/ecology.

Solitary (Fig. 280). Host: Crambidae, *Phostria euagra*, *Phostria latiapicalis*, *Phostria metalobalis*, *Phostria* Solis237, *Pilocrocis purpurascens*, *Pilocrocis xanthozonalis*.

#### Distribution.

Costa Rica, ACG.

#### Etymology.

We dedicate this species to Felix Carmona in recognition of his diligent efforts for the ACG Programa Forestal in the Estación Experimental Forestal Horizontes.

### Apanteles fernandochavarriai

Fernández-Triana sp. n.

http://zoobank.org/C7662104-18D3-4F1B-BA05-452E170E557D

http://species-id.net/wiki/Apanteles_fernandochavarriai

Figs 41 , 237

#### Type locality.

COSTA RICA, Guanacaste, ACG, Sector Del Oro, Tangelo, 410m, 11.01823, -85.45024.

#### Holotype.

♀ in CNC. Specimen labels: 1. DHJPAR0020604. 2. Voucher: D.H.Janzen & W.Hallwachs, DB: http://janzen.sas.upenn.edu, Area de Conservación Guanacaste, COSTA RICA, 07-SRNP-24436.

#### Paratypes.

2 ♀, 1 ♂ (CNC, NMNH). COSTA RICA, ACG database codes: DHJPAR0038306, DHJPAR0038317, DHJPAR0038318.

#### Description.

**Female.** Body color: body mostly dark except for some sternites which may be pale. Antenna color: scape, pedicel, and flagellum dark. Coxae color (pro-, meso-, metacoxa): dark, dark, dark. Femora color (pro-, meso-, metafemur): anteriorly dark/posteriorly pale, dark, dark. Tibiae color (pro-, meso-, metatibia): pale, pale, anteriorly pale/posteriorly dark. Tegula and humeral complex color: tegula pale, humeral complex half pale/half dark. Pterostigma color: mostly pale and/or transparent, with thin dark borders. Fore wing veins color: mostly white or entirely transparent. Antenna length/body length: antenna about as long as body (head to apex of metasoma); if slightly shorter, at least extending beyond anterior 0.7 metasoma length. Body in lateral view: not distinctly flattened dorso–ventrally. Body length (head to apex of metasoma): 3.1–3.2 mm, 3.3–3.4 mm or 3.5–3.6 mm. Fore wing length: 3.1–3.2 mm, 3.3–3.4 mm or 3.5–3.6 mm. Ocular–ocellar line/posterior ocellus diameter: 2.6 or more. Interocellar distance/posterior ocellus diameter: 1.7–1.9. Antennal flagellomerus 2 length/width: 2.6–2.8. Antennal flagellomerus 14 length/width: 1.7–1.9. Length of flagellomerus 2/length of flagellomerus 14: 2.0–2.2. Tarsal claws: with single basal spine–like seta. Metafemur length/width: 2.8–2.9. Metatibia inner spur length/metabasitarsus length: 0.4–0.5. Anteromesoscutum: mostly with deep, dense punctures (separated by less than 2.0 × its maximum diameter). Mesoscutellar disc: mostly smooth. Number of pits in scutoscutellar sulcus: 9 or 10. Maximum height of mesoscutellum lunules/maximum height of lateral face of mesoscutellum: 0.6–0.7. Propodeum areola: completely defined by carinae, including transverse carina extending to spiracle. Propodeum background sculpture: mostly sculptured. Mediotergite 1 length/width at posterior margin: 2.9–3.1. Mediotergite 1 shape: mostly parallel–sided for 0.5–0.7 of its length, then narrowing posteriorly so mediotergite anterior width >1.1 × posterior width. Mediotergite 1 sculpture: mostly sculptured, excavated area centrally with transverse striation inside and/or a polished knob centrally on posterior margin of mediotergite. Mediotergite 2 width at posterior margin/length: 1.6–1.9. Mediotergite 2 sculpture: mostly smooth. Outer margin of hypopygium: with a wide, medially folded, transparent, semi–desclerotized area; usually with 4 or more pleats. Ovipositor thickness: about same width throughout its length. Ovipositor sheaths length/metatibial length: 1.8–1.9. Length of fore wing veins r/2RS: 1.7–1.9. Length of fore wing veins 2RS/2M: 1.4–1.6. Length of fore wing veins 2M/(RS+M)b: 0.5–0.6. Pterostigma length/width: 3.6 or more. Point of insertion of vein r in pterostigma: clearly beyond half way point length of pterostigma. Angle of vein r with fore wing anterior margin: more or less perpendicular to fore wing margin. Shape of junction of veins r and 2RS in fore wing: distinctly but not strongly angled.

**Male.** As in female, except for a more transverse mediotergite 2.

#### Molecular data.

Sequences in BOLD: 8, barcode compliant sequences: 8.

#### Biology/ecology.

Solitary (Fig. 237). Hosts: Elachistidae, *Antaeotricha* Janzen77, *Antaeotricha* Janzen31, *Antaeotricha* Janzen140DHJ01, *Cerconota* Janzen82.

#### Distribution.

Costa Rica, ACG

#### Etymology.

We dedicate this species to Fernando Chavarría in recognition of his diligent efforts for the ACG Programa de Seguridad.

### Apanteles flormoralesae

Fernández-Triana sp. n.

http://zoobank.org/2862862A-2028-4C30-892B-3CFE16FFB675

http://species-id.net/wiki/Apanteles_flormoralesae

Figs 118 , 284

#### Type locality.

COSTA RICA, Guanacaste, ACG, Sector Santa Maria, Sendero Canal, 799m, 10.76544, -85.28539.

#### Holotype.

♀ in CNC. Specimen labels: 1. Voucher: D.H.Janzen & W.Hallwachs, DB: http://janzen.sas.upenn.edu, Area de Conservación Guanacaste, COSTA RICA, 09-SRNP-56182. 2. DHJPAR0039782.

#### Paratypes.

1 ♂ (CNC). COSTA RICA: Guanacaste, ACG database code: DHJPAR0039773.

#### Description.

**Female.** Body color: head dark, mesosoma dark with parts of axillar complex pale, metasoma with some mediotergites, most laterotergites, sternites, and/or hypopygium pale. Antenna color: scape, pedicel, and flagellum pale. Coxae color (pro-, meso-, metacoxa): pale, pale, pale. Femora color (pro-, meso-, metafemur): pale, pale, pale. Tibiae color (pro-, meso-, metatibia): pale, pale, mostly pale but with posterior 0.2 or less dark. Tegula and humeral complex color: both pale. Pterostigma color: dark. Fore wing veins color: mostly dark (a few veins may be unpigmented). Antenna length/body length: antenna about as long as body (head to apex of metasoma); if slightly shorter, at least extending beyond anterior 0.7 metasoma length. Body in lateral view: not distinctly flattened dorso–ventrally. Body length (head to apex of metasoma): 2.9–3.0 mm. Fore wing length: 3.1–3.2 mm. Ocular–ocellar line/posterior ocellus diameter: 1.7–1.9. Interocellar distance/posterior ocellus diameter: 1.7–1.9. Antennal flagellomerus 2 length/width: 2.6–2.8. Antennal flagellomerus 14 length/width: 1.7–1.9. Length of flagellomerus 2/length of flagellomerus 14: 2.0–2.2. Tarsal claws: simple. Metafemur length/width: 3.0–3.1. Metatibia inner spur length/metabasitarsus length: 0.4–0.5. Anteromesoscutum: mostly with deep, dense punctures (separated by less than 2.0 × its maximum diameter). Mesoscutellar disc: mostly punctured. Number of pits in scutoscutellar sulcus: 5 or 6. Maximum height of mesoscutellum lunules/maximum height of lateral face of mesoscutellum: 0.4–0.5. Propodeum areola: completely defined by carinae, including transverse carina extending to spiracle. Propodeum background sculpture: mostly sculptured. Mediotergite 1 length/width at posterior margin: 2.9–3.1. Mediotergite 1 shape: mostly parallel–sided for 0.5–0.7 of its length, then narrowing posteriorly so mediotergite anterior width >1.1 × posterior width. Mediotergite 1 sculpture: mostly sculptured, excavated area centrally with transverse striation inside and/or a polished knob centrally on posterior margin of mediotergite. Mediotergite 2 width at posterior margin/length: 3.2–3.5. Mediotergite 2 sculpture: mostly smooth. Outer margin of hypopygium: with a wide, medially folded, transparent, semi–desclerotized area; usually with 4 or more pleats. Ovipositor thickness: anterior width at most 2.0 × posterior width (beyond ovipositor constriction) (?). Ovipositor sheaths length/metatibial length: 0.4–0.5. Length of fore wing veins r/2RS: 2.0–2.2. Length of fore wing veins 2RS/2M: 0.9–1.0. Length of fore wing veins 2M/(RS+M)b: 0.9–1.0. Pterostigma length/width: 3.6 or more. Point of insertion of vein r in pterostigma: about half way point length of pterostigma. Angle of vein r with fore wing anterior margin: more or less perpendicular to fore wing margin. Shape of junction of veins r and 2RS in fore wing: strongly angulated, sometimes with a knob.

**Male.** As in female, but metafemur darker.

#### Molecular data.

Sequences in BOLD: 5, barcode compliant sequences: 5.

#### Biology/ecology.

Solitary (Fig. 284). Host: Crambidae. *Herpetogramma* Solis10.

#### Distribution.

Costa Rica, ACG.

#### Comments.

This species is characterized by extensive yellow coloration, mesoscutellar disc mostly punctured and very short ovipositor sheaths (0.4 × as long as metatibia length).

#### Etymology.

We dedicate this species to Flor Morales in recognition of her diligent efforts for the ACG Programa de Mantenimento.

### Apanteles franciscopizarroi

Fernández-Triana sp. n.

http://zoobank.org/84DCE9B2-79E9-47CF-8CC2-8DA9F53F3AD1

http://species-id.net/wiki/Apanteles_franciscopizarroi

Figs 74

#### Type locality.

COSTA RICA, Guanacaste, ACG, Sector Santa Rosa, Bosque Humedo, 290m, 10.85145, -85.60801.

#### Holotype.

♀ in CNC. Specimen labels: 1. DHJPAR0013119. 2. 17 Jan. 2000, Bosque Humedo Trap.

#### Description.

**Female.** Body color: body mostly dark except for some sternites which may be pale. Antenna color: scape, pedicel, and flagellum dark. Coxae color (pro-, meso-, metacoxa): pale, pale, dark. Femora color (pro-, meso-, metafemur): pale, pale, mostly pale but posterior 0.2 or less dark. Tibiae color (pro-, meso-, metatibia): pale, pale, anteriorly pale/posteriorly dark. Tegula and humeral complex color: both pale. Pterostigma color: dark. Fore wing veins color: partially pigmented (a few veins may be dark but most are pale). Antenna length/body length: antenna about as long as body (head to apex of metasoma); if slightly shorter, at least extending beyond anterior 0.7 metasoma length. Body in lateral view: not distinctly flattened dorso–ventrally. Body length (head to apex of metasoma): 2.1–2.2 mm. Fore wing length: 2.3–2.4 mm. Ocular–ocellar line/posterior ocellus diameter: 2.0–2.2. Interocellar distance/posterior ocellus diameter: 1.7–1.9. Antennal flagellomerus 2 length/width: 2.6–2.8. Antennal flagellomerus 14 length/width: 1.4–1.6. Length of flagellomerus 2/length of flagellomerus 14: 1.7–1.9. Tarsal claws: with single basal spine–like seta. Metafemur length/width: 3.4–3.5. Metatibia inner spur length/metabasitarsus length: 0.4–0.5. Anteromesoscutum: mostly with deep, dense punctures (separated by less than 2.0 × its maximum diameter). Mesoscutellar disc: with a few sparse punctures. Number of pits in scutoscutellar sulcus: 11 or 12. Maximum height of mesoscutellum lunules/maximum height of lateral face of mesoscutellum: 0.2–0.3. Propodeum areola: completely defined by carinae, including transverse carina extending to spiracle. Propodeum background sculpture: partly sculptured, especially on anterior 0.5. Mediotergite 1 length/width at posterior margin: 2.0–2.2. Mediotergite 1 shape: mostly parallel–sided for 0.5–0.7 of its length, then narrowing posteriorly so mediotergite anterior width >1.1 × posterior width. Mediotergite 1 sculpture: mostly sculptured, excavated area centrally with transverse striation inside and/or a polished knob centrally on posterior margin of mediotergite. Mediotergite 2 width at posterior margin/length: 3.6–3.9. Mediotergite 2 sculpture: with some sculpture, mostly near posterior margin. Outer margin of hypopygium: with a wide, medially folded, transparent, semi–desclerotized area; usually with 4 or more pleats. Ovipositor thickness: about same width throughout its length. Ovipositor sheaths length/metatibial length: 1.0–1.1. Length of fore wing veins r/2RS: 1.1–1.3. Length of fore wing veins 2RS/2M: 1.4–1.6. Length of fore wing veins 2M/(RS+M)b: 1.1–1.3. Pterostigma length/width: 3.1–3.5. Point of insertion of vein r in pterostigma: about half way point length of pterostigma. Angle of vein r with fore wing anterior margin: more or less perpendicular to fore wing margin. Shape of junction of veins r and 2RS in fore wing: distinctly but not strongly angled.

**Male.** Unknown.

#### Molecular data.

Sequences in BOLD: 2, barcode compliant sequences: 2.

#### Biology/ecology.

Malaise-trapped.

#### Distribution.

Costa Rica, ACG.

#### Etymology.

We dedicate this species to Francisco Pizarro in recognition of his diligent efforts for the ACG Programa de Ecoturismo.

### Apanteles franciscoramirezi

Fernández-Triana sp. n.

http://zoobank.org/B5B6BE94-34BD-4D2E-9194-08B3AD6EF616

http://species-id.net/wiki/Apanteles_franciscoramirezi

Fig. 42

#### Type locality.

COSTA RICA, Alajuela, ACG, Sector San Cristobal, Estación San Gerardo, 575m, 10.88009, -85.38887.

#### Holotype.

♀ in CNC. Specimen labels: 1. DHJPAR0025841. 2. San Gerardo: Est. San Gerardo, 16-22 Jun. 2007.

#### Description.

**Female.** Body color: body mostly dark except for some sternites which may be pale. Antenna color: scape, pedicel, and flagellum dark. Coxae color (pro-, meso-, metacoxa): dark, dark, dark. Femora color (pro-, meso-, metafemur): pale, pale, mostly pale but posterior 0.2 or less dark. Tibiae color (pro-, meso-, metatibia): pale, pale, anteriorly pale/posteriorly dark. Tegula and humeral complex color: both pale. Pterostigma color: dark with pale spot at base. Fore wing veins color: mostly dark (a few veins may be unpigmented). Antenna length/body length: antenna about as long as body (head to apex of metasoma); if slightly shorter, at least extending beyond anterior 0.7 metasoma length. Body in lateral view: not distinctly flattened dorso–ventrally. Body length (head to apex of metasoma): 3.1–3.2 mm. Fore wing length: 3.1–3.2 mm. Ocular–ocellar line/posterior ocellus diameter: 2.6 or more. Interocellar distance/posterior ocellus diameter: 2.0–2.2. Antennal flagellomerus 2 length/width: 2.3–2.5. Antennal flagellomerus 14 length/width: 1.4–1.6. Length of flagellomerus 2/length of flagellomerus 14: 2.0–2.2. Tarsal claws: with single basal spine–like seta. Metafemur length/width: 2.8–2.9. Metatibia inner spur length/metabasitarsus length: 0.4–0.5. Anteromesoscutum: mostly with deep, dense punctures (separated by less than 2.0 × its maximum diameter). Mesoscutellar disc: mostly smooth. Number of pits in scutoscutellar sulcus: 9 or 10. Maximum height of mesoscutellum lunules/maximum height of lateral face of mesoscutellum: 0.4–0.5. Propodeum areola: completely defined by carinae, including transverse carina extending to spiracle. Propodeum background sculpture: partly sculptured, especially on anterior 0.5. Mediotergite 1 length/width at posterior margin: 2.3–2.5. Mediotergite 1 shape: slightly widening from anterior margin to 0.7–0.8 mediotergite length (where maximum width is reached), then narrowing towards posterior margin. Mediotergite 1 sculpture: mostly sculptured, excavated area centrally with transverse striation inside and/or a polished knob centrally on posterior margin of mediotergite. Mediotergite 2 width at posterior margin/length: 1.6–1.9. Mediotergite 2 sculpture: mostly smooth. Outer margin of hypopygium: with a wide, medially folded, transparent, semi–desclerotized area; usually with 4 or more pleats. Ovipositor thickness: about same width throughout its length. Ovipositor sheaths length/metatibial length: 1.2–1.3. Length of fore wing veins r/2RS: 2.3 or more. Length of fore wing veins 2RS/2M: 1.4–1.6. Length of fore wing veins 2M/(RS+M)b: 0.5–0.6. Pterostigma length/width: 2.6–3.0. Point of insertion of vein r in pterostigma: about half way point length of pterostigma. Angle of vein r with fore wing anterior margin: more or less perpendicular to fore wing margin. Shape of junction of veins r and 2RS in fore wing: distinctly but not strongly angled.

**Male.** Unknown.

#### Molecular data.

Sequences in BOLD: 2, barcode compliant sequences: 2.

#### Biology/ecology.

Solitary. Host: Elachistidae, *Antaeotricha* Janzen727.

#### Distribution.

Costa Rica, ACG.

#### Etymology.

We dedicate this species to Francisco Ramírez in recognition of his diligent efforts for the administration of ACG and Area de Conservacion Huetar Norte.

### Apanteles freddyquesadai

Fernández-Triana sp. n.

http://zoobank.org/4F7FC2BF-5838-4FA0-85F0-D8F2A5AD7075

http://species-id.net/wiki/Apanteles_freddyquesadai

Figs 56 , 249

Apanteles Rodriguez46 (Smith et al. 2006). Interim name provided by the authors.

#### Type locality.

COSTA RICA, Guanacaste, ACG, Sector El Hacha, Sendero Potrero, 290m, 11.02842, -85.52779.

#### Holotype.

♀ in CNC. Specimen labels: 1. COSTA RICA, Guanacaste, ACG, El Hacha, Estación los Almendros, 07/02/2000, Lucia Rios. 2. 00-SRNP-3040, Atarnes sallei on Annona reticulata. 3. DHJPAR0012472.

#### Paratypes.

7 ♀, 5 ♂ (BMNH, CNC, INBIO, NMNH). COSTA RICA, ACG database codes: DHJPAR0002316, DHJPAR0002328, DHJPAR0002337, DHJPAR0002910, DHJPAR0002913, DHJPAR0002916, DHJPAR0002921, DHJPAR0004059, DHJPAR0004086, DHJPAR0005276, DHJPAR0011975, DHJPAR0034223.

#### Description.

**Female.** Body color: body mostly dark except for some sternites which may be pale. Antenna color: scape, pedicel, and flagellum dark. Coxae color (pro-, meso-, metacoxa): pale, pale, dark. Femora color (pro-, meso-, metafemur): pale, pale, pale. Tibiae color (pro-, meso-, metatibia): pale, pale, pale. Tegula and humeral complex color: both pale. Pterostigma color: mostly pale and/or transparent, with thin dark borders. Fore wing veins color: partially pigmented (a few veins may be dark but most are pale). Antenna length/body length: antenna about as long as body (head to apex of metasoma); if slightly shorter, at least extending beyond anterior 0.7 metasoma length. Body in lateral view: not distinctly flattened dorso–ventrally. Body length (head to apex of metasoma): 2.9–3.0 mm or 3.1–3.2 mm. Fore wing length: 3.1–3.2 mm, 3.3–3.4 mm, rarely 3.5–3.6 mm. Ocular–ocellar line/posterior ocellus diameter: 2.0–2.2. Interocellar distance/posterior ocellus diameter: 1.7–1.9. Antennal flagellomerus 2 length/width: 2.9–3.1. Antennal flagellomerus 14 length/width: 1.4–1.6. Length of flagellomerus 2/length of flagellomerus 14: 2.0–2.2. Tarsal claws: with single basal spine–like seta. Metafemur length/width: 3.0–3.1. Metatibia inner spur length/metabasitarsus length: 0.6–0.7. Anteromesoscutum: mostly with deep, dense punctures (separated by less than 2.0 × its maximum diameter). Mesoscutellar disc: with punctures near margins, central part mostly smooth. Number of pits in scutoscutellar sulcus: 7 or 8. Maximum height of mesoscutellum lunules/maximum height of lateral face of mesoscutellum: 0.6–0.7. Propodeum areola: completely defined by carinae, including transverse carina extending to spiracle. Propodeum background sculpture: mostly sculptured. Mediotergite 1 length/width at posterior margin: 3.5–3.7, 3.8–4.0, rarely 4.1 or more. Mediotergite 1 shape: mostly parallel–sided for 0.5–0.7 of its length, then narrowing posteriorly so mediotergite anterior width >1.1 × posterior width. Mediotergite 1 sculpture: with some sculpture near lateral margins and/or posterior 0.2–0.4 of mediotergite. Mediotergite 2 width at posterior margin/length: 4.4–4.7. Mediotergite 2 sculpture: mostly smooth. Outer margin of hypopygium: inflexible (without a folded, transparent, semi–desclerotized area); with no pleats visible. Ovipositor thickness: anterior width 3.0–5.0 × posterior width (beyond ovipositor constriction). Ovipositor sheaths length/metatibial length: 0.4–0.5. Length of fore wing veins r/2RS: 2.3 or more. Length of fore wing veins 2RS/2M: 1.1–1.3. Length of fore wing veins 2M/(RS+M)b: 0.5–0.6. Pterostigma length/width: 3.1–3.5. Point of insertion of vein r in pterostigma: clearly beyond half way point length of pterostigma. Angle of vein r with fore wing anterior margin: clearly outwards, inclined towards fore wing apex. Shape of junction of veins r and 2RS in fore wing: distinctly but not strongly angled.

**Male.** Similar to female.

#### Molecular data.

Sequences in BOLD: 14, barcode compliant sequences: 9.

#### Biology/ecology.

Solitary (Fig. 249). Host: Hesperiidae, *Sostrata bifasciata nordica*, *Gorgythion begga pyralina*.

#### Distribution.

Costa Rica, ACG.

#### Comments.

The barcode sequences of *Apanteles freddyquesadai* only differ from those of *Apanteles luciariosae* by 1.3% (~ 8bp), but there are clear diagnostic characters for each species in the barcoding region. In addition to molecular and slight morphological differences, *Apanteles freddyquesadai* seems to be an ecologist generalist, having been found in dry forest, mix of dry and rainforests and deep rainforests, while *Apanteles luciariosae* is an ecological specialist, only found on a small piece of deep rainforest (i.e., many kilometres into the rain forest, far from the dry forest and dry-rain forest interface).

#### Etymology.

We dedicate this species to Freddy Quesada in recognition of his diligent efforts for the ACG Programa de Parataxónomos and Estación Biológica Pitilla of ACG.

### Apanteles freddysalazari

Fernández-Triana sp. n.

http://zoobank.org/AAD74783-16D1-4641-957B-3727FDCF3621

http://species-id.net/wiki/Apanteles_freddysalazari

Fig. 43

#### Type locality.

COSTA RICA, Alajuela, ACG, Sector San Cristobal, Finca San Gabriel, 645m, 10.87766, -85.39343.

#### Holotype.

♀ in CNC. Specimen labels: 1. DHJPAR0038227. 2. Voucher: D.H.Janzen & W.Hallwachs, DB: http://janzen.sas.upenn.edu, Area de Conservación Guanacaste, COSTA RICA, 09-SRNP-6784.

#### Paratypes.

1 ♀ (CNC). COSTA RICA: Guanacaste, ACG database code: DHJPAR0045282.

#### Description.

**Female.** Body color: body mostly dark except for some sternites which may be pale. Antenna color: scape, pedicel, and flagellum dark. Coxae color (pro-, meso-, metacoxa): dark, dark, dark. Femora color (pro-, meso-, metafemur): anteriorly dark/posteriorly pale, dark, dark. Tibiae color (pro-, meso-, metatibia): pale, pale, anteriorly pale/posteriorly dark. Tegula and humeral complex color: tegula pale, humeral complex half pale/half dark. Pterostigma color: mostly pale and/or transparent, with thin dark borders. Fore wing veins color: partially pigmented (a few veins may be dark but most are pale). Antenna length/body length: antenna about as long as body (head to apex of metasoma); if slightly shorter, at least extending beyond anterior 0.7 metasoma length. Body in lateral view: not distinctly flattened dorso–ventrally. Body length (head to apex of metasoma): 3.3–3.4 mm. Fore wing length: 3.1–3.2 mm. Ocular–ocellar line/posterior ocellus diameter: 2.6 or more. Interocellar distance/posterior ocellus diameter: 1.7–1.9. Antennal flagellomerus 2 length/width: 2.6–2.8. Antennal flagellomerus 14 length/width: 1.4–1.6. Length of flagellomerus 2/length of flagellomerus 14: 2.0–2.2. Tarsal claws: with single basal spine–like seta. Metafemur length/width: 3.2–3.3. Metatibia inner spur length/metabasitarsus length: 0.4–0.5. Anteromesoscutum: mostly with shallow, dense punctures (separated by less than 2.0 × its maximum diameter). Mesoscutellar disc: mostly smooth. Number of pits in scutoscutellar sulcus: 11 or 12. Maximum height of mesoscutellum lunules/maximum height of lateral face of mesoscutellum: 0.6–0.7. Propodeum areola: completely defined by carinae, including transverse carina extending to spiracle. Propodeum background sculpture: mostly sculptured. Mediotergite 1 length/width at posterior margin: 2.3–2.5. Mediotergite 1 shape: mostly parallel–sided for 0.5–0.7 of its length, then narrowing posteriorly so mediotergite anterior width >1.1 × posterior width. Mediotergite 1 sculpture: mostly sculptured, excavated area centrally with transverse striation inside and/or a polished knob centrally on posterior margin of mediotergite. Mediotergite 2 width at posterior margin/length: 1.6–1.9. Mediotergite 2 sculpture: with some sculpture, mostly near posterior margin. Outer margin of hypopygium: with a wide, medially folded, transparent, semi–desclerotized area; usually with 4 or more pleats. Ovipositor thickness: about same width throughout its length. Ovipositor sheaths length/metatibial length: 1.6–1.7. Length of fore wing veins r/2RS: 1.4–1.6. Length of fore wing veins 2RS/2M: 1.4–1.6. Length of fore wing veins 2M/(RS+M)b: 0.7–0.8. Pterostigma length/width: 3.6 or more. Point of insertion of vein r in pterostigma: clearly beyond half way point length of pterostigma. Angle of vein r with fore wing anterior margin: clearly outwards, inclined towards fore wing apex. Shape of junction of veins r and 2RS in fore wing: distinctly but not strongly angled.

**Male.** Unknown.

#### Molecular data.

Sequences in BOLD: 2, barcode compliant sequences: 2.

#### Biology/ecology.

Solitary. Hosts: Elachistidae, *Antaeotricha* Janzen370, elachJanzen01 Janzen227.

#### Distribution.

Costa Rica, ACG.

#### Etymology.

We dedicate this species to Freddy Salazar in recognition of his diligent efforts for the ACG Sector Marino.

### Apanteles fredi

Austin & Dangerfield, 1989

http://species-id.net/wiki/Apanteles_fredi

Apanteles fredi Austin & Dangerfield, 1989: 135.

#### Type locality.

GUATEMALA, Ingenio Pantaleón.

#### Holotype.

♀, BMNH (not examined).

#### Material Examined.

1 ♀, paratype (CNC), GUATEMALA, Ingenio Pantaleón S.A., 3.iii.1984, ex larva of *Diatraea* sp.

#### Description.

**Female.** Body color: body mostly dark except for some sternites which may be pale. Antenna color: scape, pedicel, and flagellum pale. Coxae color (pro-, meso-, metacoxa): pale, pale, partially pale/partially dark. Femora color (pro-, meso-, metafemur): anteriorly dark/posteriorly pale, dark, dark. Tibiae color (pro-, meso-, metatibia): pale, pale, anteriorly pale/posteriorly dark. Tegula and humeral complex color: both pale. Pterostigma color: mostly pale and/or transparent, with thin dark borders. Fore wing veins color: partially pigmented (a few veins may be dark but most are pale). Antenna length/body length: antenna very short, barely or not extending beyond mesosoma length. Body in lateral view: distinctly flattened dorso–ventrally. Body length (head to apex of metasoma): 2.0 mm or less. Fore wing length: 2.0 mm or less. Ocular–ocellar line/posterior ocellus diameter: 2.6 or more. Interocellar distance/posterior ocellus diameter: 1.7–1.9. Antennal flagellomerus 2 length/width: 1.4–1.6. Antennal flagellomerus 14 length/width: 1.0 or less. Length of flagellomerus 2/length of flagellomerus 14: 1.7–1.9. Tarsal claws: simple. Metafemur length/width: 2.5 or less. Metatibia inner spur length/metabasitarsus length: 0.4–0.5. Anteromesoscutum: mostly smooth or with shallow sparse punctures, except for anterior 0.3 where it has deeper and/or denser punctures. Mesoscutellar disc: mostly smooth. Number of pits in scutoscutellar sulcus: 13 or 14. Maximum height of mesoscutellum lunules/maximum height of lateral face of mesoscutellum: 0.8 or more. Propodeum areola: partially defined by carinae on posterior 0.3–0.5 of its length, widely open anteriorly. Propodeum background sculpture: partly sculptured, especially on posterior 0.5. Mediotergite 1 length/width at posterior margin: 1.7–1.9. Mediotergite 1 shape: more or less parallel–sided. Mediotergite 1 sculpture: with some sculpture near lateral margins and/or posterior 0.2–0.4 of mediotergite. Mediotergite 2 width at posterior margin/length: 3.2–3.5. Mediotergite 2 sculpture: mostly smooth. Outer margin of hypopygium: with a wide, medially folded, transparent, semi–desclerotized area; usually with 4 or more pleats. Ovipositor thickness: anterior width at most 2.0 × posterior width (beyond ovipositor constriction). Ovipositor sheaths length/metatibial length: 0.6–0.7. Length of fore wing veins r/2RS: 1.0 or less. Length of fore wing veins 2RS/2M: 0.9–1.0. Length of fore wing veins 2M/(RS+M)b: 1.1–1.3. Pterostigma length/width: 2.6–3.0. Point of insertion of vein r in pterostigma: about half way point length of pterostigma. Angle of vein r with fore wing anterior margin: clearly outwards, inclined towards fore wing apex. Shape of junction of veins r and 2RS in fore wing: distinctly but not strongly angled.

**Male.** As in female, except for longer antenna, mediotergite 2 more rectangular and elongate, and legs darker in color (Austin and Dangerfield 1989).

#### Molecular data.

No molecular data available for this species.

#### Biology/ecology.

Probably gregarious. Hosts: Crambidae, *Diatraea* sp.

#### Distribution.

Guatemala (Austin and Dangerfield 1989). We have no reason to suspect that this species occurs in ACG.

### Apanteles gabrielagutierrezae

Fernández-Triana sp. n.

http://zoobank.org/A6BA4C66-41DD-452A-B744-246D30731F3F

http://species-id.net/wiki/Apanteles_gabrielagutierrezae

Figs 44 , 238

#### Type locality.

COSTA RICA, Guanacaste, ACG, Sector Cacao, Quebrada Otilio, 550m, 10.88996, -85.47966.

#### Holotype.

♀ in CNC. Specimen labels: 1. DHJPAR0020456. 2. Voucher: D.H.Janzen & W.Hallwachs, DB: http://janzen.sas.upenn.edu, Area de Conservación Guanacaste, COSTA RICA, 07-SRNP-46409.

#### Paratypes.

1 ♀ (CNC). COSTA RICA: Guanacaste, ACG database code: DHJPAR0020458.

#### Description.

**Female.** Body color: body mostly dark except for some sternites which may be pale. Antenna color: scape, pedicel, and flagellum dark. Coxae color (pro-, meso-, metacoxa): dark, dark, dark. Femora color (pro-, meso-, metafemur): anteriorly dark/posteriorly pale, dark, dark. Tibiae color (pro-, meso-, metatibia): pale, pale, anteriorly pale/posteriorly dark. Tegula and humeral complex color: tegula pale, humeral complex half pale/half dark. Pterostigma color: mostly dark, with small pale area centrally. Fore wing veins color: mostly dark (a few veins may be unpigmented). Antenna length/body length: antenna about as long as body (head to apex of metasoma); if slightly shorter, at least extending beyond anterior 0.7 metasoma length. Body in lateral view: not distinctly flattened dorso–ventrally. Body length (head to apex of metasoma): 3.7–3.8 mm. Fore wing length: 3.5–3.6 mm. Ocular–ocellar line/posterior ocellus diameter: 2.3–2.5. Interocellar distance/posterior ocellus diameter: 1.7–1.9. Antennal flagellomerus 2 length/width: 2.6–2.8. Antennal flagellomerus 14 length/width: 1.7–1.9. Length of flagellomerus 2/length of flagellomerus 14: 2.0–2.2. Tarsal claws: simple. Metafemur length/width: 3.2–3.3. Metatibia inner spur length/metabasitarsus length: 0.4–0.5. Anteromesoscutum: mostly with deep, dense punctures (separated by less than 2.0 × its maximum diameter). Mesoscutellar disc: mostly smooth. Number of pits in scutoscutellar sulcus: 11 or 12. Maximum height of mesoscutellum lunules/maximum height of lateral face of mesoscutellum: 0.6–0.7. Propodeum areola: completely defined by carinae, including transverse carina extending to spiracle. Propodeum background sculpture: mostly sculptured. Mediotergite 1 length/width at posterior margin: 2.9–3.1 or 3.2–3.4. Mediotergite 1 shape: mostly parallel–sided for 0.5–0.7 of its length, then narrowing posteriorly so mediotergite anterior width >1.1 × posterior width. Mediotergite 1 sculpture: mostly sculptured, excavated area centrally with transverse striation inside and/or a polished knob centrally on posterior margin of mediotergite. Mediotergite 2 width at posterior margin/length: 1.5 or less. Mediotergite 2 sculpture: mostly smooth. Outer margin of hypopygium: with a wide, medially folded, transparent, semi–desclerotized area; usually with 4 or more pleats. Ovipositor thickness: about same width throughout its length. Ovipositor sheaths length/metatibial length: 1.4–1.5. Length of fore wing veins r/2RS: 1.7–1.9. Length of fore wing veins 2RS/2M: 1.7–1.8. Length of fore wing veins 2M/(RS+M)b: 0.5–0.6. Pterostigma length/width: 3.6 or more. Point of insertion of vein r in pterostigma: clearly beyond half way point length of pterostigma. Angle of vein r with fore wing anterior margin: clearly outwards, inclined towards fore wing apex. Shape of junction of veins r and 2RS in fore wing: distinctly but not strongly angled.

**Male.** Unknown.

#### Molecular data.

Sequences in BOLD: 3, barcode compliant sequences: 3.

#### Biology/ecology.

Solitary (Fig. 238). Hosts: Elachistidae, *Antaeotricha* Phillips01, *Antaeotricha* Janzen301.

#### Distribution.

Costa Rica, ACG.

#### Etymology.

We dedicate this species to Gabriela Gutiérrez in recognition of her diligent efforts for the ACG Programa de Educacion Biológica.

### Apanteles galleriae

Wilkinson, 1932

http://species-id.net/wiki/Apanteles_galleriae

Apanteles galleriae Wilkinson, 1932: 139.

#### Type locality.

FRANCE, Montpellier (Shenefelt 1972: 516).

#### Holotype.

♀, NMNH (not examined).

#### Description.

Whitfield et al. (2001) provided a comprehensive description and numerous black and white illustrations.

#### Molecular data.

Sequences in BOLD: 16, barcode compliant sequences: 12, haplotypes: 2 (but see **Comments** below).

#### Biology/ecology.

Solitary, parasitoid of early-instar larva of wax moths and emerges to spin its white cocoon and pupate well before the host larva reaches full size (Whitfield et al. 2001). Hosts: Pyralidae, *Achroia grisella*, *Achroia innonata*, *Galleria mellonella*, *Vitula edmandsii*.

#### Distribution.

Worldwide. This is a cosmopolitan species that has been introduced to many countries inadvertently with the transport of honey bees (Whitfield et al. 2001); it may occur in Guanacaste, owing to the commercial honey bee industry, but there is no evidence that it occurs in ACG.

#### Comments.

One of the sequences in BOLD (code: GBAH3182-07), was mined from GenBank and does not contain any information about its original source and/or sample identifier. It most likely represents a different species not related at all to *Apanteles galleriae*. In fact, blasting of that sequence reveals that its very close to the species *Apanteles ensiger* (Say, 1836). Because all other sequences of *Apanteles galleriae* represent the same haplotype, we here recommend that such a dubious sequence be excluded from further consideration and analysis when dealing with the species *Apanteles galleriae*.

### Apanteles garygibsoni

Fernández-Triana sp. n.

http://zoobank.org/0C2B3884-1517-4FF4-B6D9-1A67E8A95EE1

http://species-id.net/wiki/Apanteles_garygibsoni

Figs 119 , 285

Apanteles Rodriguez81 (Smith et al. 2006). Interim name provided by the authors.

#### Type locality.

COSTA RICA, Guanacaste, ACG, Sector Cacao, Quebrada Otilio, 550m, 10.88996, -85.47966.

#### Holotype.

♀ in CNC. Specimen labels: 1. Voucher: D.H.Janzen & W.Hallwachs, DB: http://janzen.sas.upenn.edu, Area de Conservación Guanacaste, COSTA RICA, 07-SRNP-45039. 2: DHJPAR0012761.

#### Paratypes.

2 ♀ (CNC, NMNH). COSTA RICA, ACG database codes: DHJPAR0012467, DHJPAR0005283.

#### Description.

**Female.** Body color: body mostly dark except for some sternites which may be pale. Antenna color: scape, pedicel, and flagellum dark. Coxae color (pro-, meso-, metacoxa): dark, dark, dark. Femora color (pro-, meso-, metafemur): pale, pale, mostly pale but with dark area dorsally. Tibiae color (pro-, meso-, metatibia): pale, pale, mostly pale but with posterior 0.2 or less dark. Tegula and humeral complex color: both pale. Pterostigma color: dark with pale spot at base. Fore wing veins color: partially pigmented (a few veins may be dark but most are pale). Antenna length/body length: antenna about as long as body (head to apex of metasoma); if slightly shorter, at least extending beyond anterior 0.7 metasoma length. Body in lateral view: not distinctly flattened dorso–ventrally. Body length (head to apex of metasoma): 3.1–3.2 mm. Fore wing length: 3.3–3.4 mm or 3.5–3.6 mm. Ocular–ocellar line/posterior ocellus diameter: 2.0–2.2. Interocellar distance/posterior ocellus diameter: 2.0–2.2. Antennal flagellomerus 2 length/width: 2.3–2.5. Antennal flagellomerus 14 length/width: 1.4–1.6. Length of flagellomerus 2/length of flagellomerus 14: 2.0–2.2. Tarsal claws: cleft, with wide, basal tooth. Metafemur length/width: 2.8–2.9. Metatibia inner spur length/metabasitarsus length: 0.6–0.7. Anteromesoscutum: mostly with deep, dense punctures (separated by less than 2.0 × its maximum diameter). Mesoscutellar disc: with a few sparse punctures. Number of pits in scutoscutellar sulcus: 11 or 12. Maximum height of mesoscutellum lunules/maximum height of lateral face of mesoscutellum: 0.4–0.5. Propodeum areola: completely defined by carinae, but only partial or absent transverse carina. Propodeum background sculpture: mostly sculptured. Mediotergite 1 length/width at posterior margin: 1.4–1.6. Mediotergite 1 shape: more or less parallel–sided. Mediotergite 1 sculpture: mostly sculptured, excavated area centrally with transverse striation inside and/or a polished knob centrally on posterior margin of mediotergite. Mediotergite 2 width at posterior margin/length: 4.8 or more. Mediotergite 2 sculpture: more or less fully sculptured, with longitudinal striation. Outer margin of hypopygium: with a wide, medially folded, transparent, semi–desclerotized area; usually with 4 or more pleats. Ovipositor thickness: anterior width at most 2.0 × posterior width (beyond ovipositor constriction). Ovipositor sheaths length/metatibial length: 1.2–1.3. Length of fore wing veins r/2RS: 2.3 or more. Length of fore wing veins 2RS/2M: 1.1–1.3. Length of fore wing veins 2M/(RS+M)b: 0.7–0.8. Pterostigma length/width: 3.1–3.5. Point of insertion of vein r in pterostigma: about half way point length of pterostigma. Angle of vein r with fore wing anterior margin: clearly outwards, inclined towards fore wing apex. Shape of junction of veins r and 2RS in fore wing: distinctly but not strongly angled.

**Male.** Unknown.

#### Molecular data.

Sequences in BOLD: 8, barcode compliant sequences: 8.

#### Biology/ecology.

Solitary (Fig. 285). Host: Hesperiidae, *Xenophanes tryxus*.

#### Distribution.

Costa Rica, ACG

#### Comments.

This is the only species of Microgastrinae known to parasitize the genus *Xenophanes*. The species is also unique on the account of its cleft tarsal claws, broad mediotergite 1, coarsely sculptured with longitudinal and transverse striation, strongly transverse mediotergite 2 with sculpture along the apical border.

#### Etymology.

The senior author dedicates this species to Gary Gibson (CNC, Ottawa) in appreciation of his support and the valuable suggestions regarding many technical details of this paper.

### Apanteles gerardobandoi

Fernández-Triana sp. n.

http://zoobank.org/9862F30A-BB63-47EA-AB41-74B0FCA1A6F5

http://species-id.net/wiki/Apanteles_gerardobandoi

Fig. 179

Apanteles Rodriguez155 (Smith et al. 2006). Interim name provided by the authors.

#### Type locality.

COSTA RICA, Guanacaste, ACG, Sector Pocosol, Quebrada Aserradero, 160m, 10.89857, -85.56419.

#### Holotype.

♀ in CNC. Specimen labels: 1. DHJPAR0013696, 94-SRNP-6192. 2. Guanacaste, ACG, Sector Pocosol, Quebrada Aserradero, 02.xii.1994, 160m, 10.89857, -85.56419, 94-SRNP-6192.

#### Paratypes.

8 ♀, 4 ♂ (BMNH, CNC, INBIO, INHS, NMNH). COSTA RICA, ACG database codes: DHJPAR0012485, DHJPAR0013681, DHJPAR0013691, 93-SRNP-996.

#### Description.

**Female.** Metatibia color (outer face): entirely or mostly (>0.7 metatibia length) dark brown to black, with yellow to white coloration usually restricted to anterior 0.2 or less, rarely with extended pale coloration (light yellow to orange–yellow), ranging from 0.4 to almost entire metatibia length. Fore wing veins color: veins C+Sc+R and R1 with brown coloration restricted narrowly to borders, interior area of those veins and pterostigma (and sometimes veins r, 2RS and 2M) transparent or white; other veins mostly transparent. Body length (head to apex of metasoma): 2.0 mm or less or 2.1–2.2 mm. Fore wing length: 2.1–2.2 mm or 2.3–2.4 mm. Metafemur length/width: 2.8–2.9, 3.0–3.1, rarely 3.2–3.3. Mediotergite 1 length/width at posterior margin: 1.7–1.8, 1.9–2.0 or 2.1–2.2. Mediotergite 1 maximum width/width at posterior margin: 1.0–1.1 or 1.2–1.3. Ovipositor sheaths length/metafemur length: 1.0 or 1.1. Ovipositor sheaths length/metatibia length: 0.8 or 0.9.

#### Molecular data.

Sequences in BOLD: 4, barcode compliant sequences: 2.

#### Biology/ecology.

Gregarious. Host: Hesperiidae, *Telemiades fides*.

#### Distribution.

Costa Rica, ACG.

#### Comments.

A few specimens have lighter coloured tibiae but are kept as part of this species.

#### Etymology.

We dedicate this species to Gerardo Obando in recognition of his diligent efforts for the ACG Programa de Sectores.

### Apanteles gerardosandovali

Fernández-Triana sp. n.

http://zoobank.org/D8C48382-533C-449D-945F-C494DE98906B

http://species-id.net/wiki/Apanteles_gerardosandovali

Fig. 8

Apanteles Rodriguez68. Smith et al. (2008). Interim name provided by the authors.

#### Type locality.

COSTA RICA, Guanacaste, ACG, Sector Pitilla, Pasmompa, 440m, 11.01926, -85.40997.

#### Holotype.

♀ in CNC. Specimen labels: 1. Costa Rica: Guanacaste, ACG, Sector Pitilla, Pasmompa, 22.vi.2004, 440m, 11.01926, -85.40997, 04-SRNP-33582.

#### Paratypes.

1 ♀, 3 ♂ (CNC). COSTA RICA, ACG database codes: DHJPAR0004091, 04-SRNP-33582, 04-SRNP-34511.

#### Description.

**Female.** Body color: body mostly dark except for some sternites which may be pale. Antenna color: scape, pedicel, and flagellum dark. Coxae color (pro-, meso-, metacoxa): dark, dark, dark. Femora color (pro-, meso-, metafemur): anteriorly dark/posteriorly pale, dark, dark. Tibiae color (pro-, meso-, metatibia): pale, pale, mostly dark but anterior 0.2 or less pale. Tegula and humeral complex color: tegula pale, humeral complex half pale/half dark. Pterostigma color: mostly pale and/or transparent, with thin dark borders. Fore wing veins color: partially pigmented (a few veins may be dark but most are pale). Antenna length/body length: antenna about as long as body (head to apex of metasoma); if slightly shorter, at least extending beyond anterior 0.7 metasoma length. Body in lateral view: not distinctly flattened dorso–ventrally. Body length (head to apex of metasoma): 2.5–2.6 mm or 2.7–2.8 mm. Fore wing length: 2.9–3.0 mm. Ocular–ocellar line/posterior ocellus diameter: 2.0–2.2, rarely 2.3–2.5. Interocellar distance/posterior ocellus diameter: 2.0–2.2. Antennal flagellomerus 2 length/width: 2.6–2.8, 2.9–3.1, rarely 2.3–2.5. Antennal flagellomerus 14 length/width: 1.4–1.6, rarely 1.7–1.9. Length of flagellomerus 2/length of flagellomerus 14: 2.0–2.2. Tarsal claws: with single basal spine–like seta. Metafemur length/width: 3.2–3.3. Metatibia inner spur length/metabasitarsus length: 0.4–0.5. Anteromesoscutum: mostly with deep, dense punctures (separated by less than 2.0 × its maximum diameter). Mesoscutellar disc: mostly punctured. Number of pits in scutoscutellar sulcus: 7 or 8. Maximum height of mesoscutellum lunules/maximum height of lateral face of mesoscutellum: 0.6–0.7. Propodeum areola: completely defined by carinae, including transverse carina extending to spiracle. Propodeum background sculpture: mostly sculptured. Mediotergite 1 length/width at posterior margin: 2.0–2.2. Mediotergite 1 shape: mostly parallel–sided for 0.5–0.7 of its length, then narrowing posteriorly so mediotergite anterior width >1.1 × posterior width. Mediotergite 1 sculpture: mostly sculptured, excavated area centrally with transverse striation inside and/or a polished knob centrally on posterior margin of mediotergite. Mediotergite 2 width at posterior margin/length: 2.8–3.1. Mediotergite 2 sculpture: mostly smooth. Outer margin of hypopygium: with a wide, medially folded, transparent, semi–desclerotized area; usually with 4 or more pleats. Ovipositor thickness: about same width throughout its length. Ovipositor sheaths length/metatibial length: 1.2–1.3. Length of fore wing veins r/2RS: 1.4–1.6. Length of fore wing veins 2RS/2M: 1.1–1.3. Length of fore wing veins 2M/(RS+M)b: 0.7–0.8. Pterostigma length/width: 3.1–3.5. Point of insertion of vein r in pterostigma: clearly beyond half way point length of pterostigma. Angle of vein r with fore wing anterior margin: clearly outwards, inclined towards fore wing apex. Shape of junction of veins r and 2RS in fore wing: distinctly but not strongly angled.

**Male.** As in female.

#### Molecular data.

Sequences in BOLD: 2, barcode compliant sequences: 1.

#### Biology/ecology.

Gregarious. Host: Elachistidae, *Stenoma fenestra*.

#### Distribution.

Costa Rica, ACG.

#### Etymology.

We dedicate this species to Gerardo Sandoval in recognition of his diligent efforts for the ACG Programa de Seguridad.

### Apanteles gladysrojasae

Fernández-Triana sp. n.

http://zoobank.org/779DFEFE-F4D7-40E3-AC10-E99AA2687F56

http://species-id.net/wiki/Apanteles_gladysrojasae

Figs 180 , 311

Apanteles Rodriguez65 (Smith et al. 2006). Interim name provided by the authors.

#### Type locality.

COSTA RICA, Alajuela, ACG, Sector San Cristobal, Rio Blanco Abajo, 500m, 10.90037, -85.37254.

#### Holotype.

♀ in CNC. Specimen labels: 1. COSTA RICA, Alajuela, ACG, Sector San Cristobal, Rio Blanco Abajo, 05.ii.2002, 500m, 10.90037, -85.37254, DHJPAR0002683.

#### Paratypes.

29 ♀, 19 ♂ (BMNH, CNC, INBIO, INHS, NMNH). COSTA RICA, ACG database codes: See Appendix 2 for detailed label data.

#### Description.

**Female.** Metatibia color (outer face): with extended pale coloration (light yellow to orange–yellow), ranging from 0.4 to almost entire metatibia length. Fore wing veins color: veins C+Sc+R and R1 with brown coloration restricted narrowly to borders, interior area of those veins and pterostigma (and sometimes veins r, 2RS and 2M) transparent or white; other veins mostly transparent. Antenna length/body length: antenna about as long as body (head to apex of metasoma); if slightly shorter, at least extending beyond anterior 0.7 metasoma length. Body length (head to apex of metasoma): 2.1–2.2 mm or 2.3–2.4 mm. Fore wing length: 2.1–2.2 mm, 2.3–2.4 mm or 2.5–2.6 mm. Metafemur length/width: 2.8–2.9 or 3.0–3.1. Mediotergite 1 length/width at posterior margin: 2.3–2.4. Mediotergite 1 maximum width/width at posterior margin: 1.4–1.5. Ovipositor sheaths length/metafemur length: 0.8 or 0.9. Ovipositor sheaths length/metatibia length: 0.7 or 0.8.

#### Molecular data.

Sequences in BOLD: 13, barcode compliant sequences: 13.

#### Biology/ecology.

Gregarious (Fig. 311). Hosts: Hesperiidae, *Urbanus belli*, *Urbanus viterboana*.

#### Distribution.

Costa Rica, ACG.

#### Etymology.

We dedicate this species to Gladys Rojas in recognition of her diligent efforts in the Administration of INBio, Costa Rica’s Instituto Nacional de Biodiversidad.

### Apanteles glenriverai

Fernández-Triana sp. n.

http://zoobank.org/D7B70708-4F50-46D7-9929-7C1CD6CD2657

http://species-id.net/wiki/Apanteles_glenriverai

Figs 120 , 286

#### Type locality.

COSTA RICA, Guanacaste, ACG, Sector Mundo Nuevo, Vado Ocotea, 565m, 10.76387, -85.37840.

#### Holotype.

♀ in CNC. Specimen labels: 1. DHJPAR0031075. 2. Voucher: D.H.Janzen & W.Hallwachs, DB: http://janzen.sas.upenn.edu, Area de Conservación Guanacaste, COSTA RICA, 08-SRNP-56543.

#### Paratypes.

4 ♀, 6 ♂ (BMNH, CNC, INBIO, INHS, NMNH). COSTA RICA, ACG database codes: DHJPAR0031075, DHJPAR0031078.

#### Description.

**Female.** Body color: body mostly dark except for some sternites which may be pale. Antenna color: scape, pedicel, and flagellum dark. Coxae color (pro-, meso-, metacoxa): dark, dark, dark. Femora color (pro-, meso-, metafemur): pale, dark, dark. Tibiae color (pro-, meso-, metatibia): pale, pale, anteriorly pale/posteriorly dark. Tegula and humeral complex color: tegula pale, humeral complex half pale/half dark. Pterostigma color: mostly pale and/or transparent, with thin dark borders. Fore wing veins color: partially pigmented (a few veins may be dark but most are pale). Antenna length/body length: antenna shorter than body (head to apex of metasoma), not extending beyond anterior 0.7 metasoma length. Body in lateral view: not distinctly flattened dorso–ventrally. Body length (head to apex of metasoma): 2.7–2.8 mm. Fore wing length: 2.7–2.8 mm or 2.9–3.0 mm. Ocular–ocellar line/posterior ocellus diameter: 2.3–2.5. Interocellar distance/posterior ocellus diameter: 1.7–1.9. Antennal flagellomerus 2 length/width: 2.3–2.5. Antennal flagellomerus 14 length/width: 1.1–1.3. Length of flagellomerus 2/length of flagellomerus 14: 2.0–2.2. Tarsal claws: simple. Metafemur length/width: 3.0–3.1. Metatibia inner spur length/metabasitarsus length: 0.4–0.5. Anteromesoscutum: mostly with deep, dense punctures (separated by less than 2.0 × its maximum diameter). Mesoscutellar disc: with a few sparse punctures. Number of pits in scutoscutellar sulcus: 9 or 10. Maximum height of mesoscutellum lunules/maximum height of lateral face of mesoscutellum: 0.4–0.5. Propodeum areola: completely defined by carinae, including transverse carina extending to spiracle. Propodeum background sculpture: mostly sculptured. Mediotergite 1 length/width at posterior margin: 2.0–2.2. Mediotergite 1 shape: slightly widening from anterior margin to 0.7–0.8 mediotergite length (where maximum width is reached), then narrowing towards posterior margin. Mediotergite 1 sculpture: mostly sculptured, excavated area centrally with transverse striation inside and/or a polished knob centrally on posterior margin of mediotergite. Mediotergite 2 width at posterior margin/length: 4.8 or more. Mediotergite 2 sculpture: with some sculpture, mostly near posterior margin. Outer margin of hypopygium: with a wide, medially folded, transparent, semi–desclerotized area; usually with 4 or more pleats. Ovipositor thickness: anterior width 3.0–5.0 × posterior width (beyond ovipositor constriction). Ovipositor sheaths length/metatibial length: 0.6–0.7. Length of fore wing veins r/2RS: 1.7–1.9. Length of fore wing veins 2RS/2M: 1.4–1.6. Length of fore wing veins 2M/(RS+M)b: 0.5–0.6. Pterostigma length/width: 2.6–3.0. Point of insertion of vein r in pterostigma: about half way point length of pterostigma. Angle of vein r with fore wing anterior margin: clearly outwards, inclined towards fore wing apex. Shape of junction of veins r and 2RS in fore wing: distinctly but not strongly angled.

**Male.** Similar to female, but with darker coloration in hind legs (especially metatibia) and narrower mediotergite 1.

#### Molecular data.

Sequences in BOLD: 6, barcode compliant sequences: 6.

#### Biology/ecology.

Gregarious (Fig. 286). Host: Pyralidae, one of the three cryptic species of *Accinctapubes albifasciata*.

#### Distribution.

Costa Rica, ACG.

#### Etymology.

We dedicate this species to Glen Rivera in recognition of his diligent efforts for the ACG Programa de Recursos Humanos.

### Apanteles gloriasihezarae

Fernández-Triana sp. n.

http://zoobank.org/19F9E2DE-53D2-4542-8134-78905349154D

http://species-id.net/wiki/Apanteles_gloriasihezarae

Figs 97 , 276

Apanteles Rodriguez13 (Smith et al. 2006). Interim name provided by the authors.

#### Type locality.

COSTA RICA, Guanacaste, ACG, Sector Cacao, Sendero Arenales, 1080m, 10.92471, -85.46738.

#### Holotype.

♀ in CNC. Specimen labels: 1. COSTA RICA, Guanacaste, ACG, Sector Cacao, Sendero Arenales, 9.x.2003, 10.92471°N, -85.46738°W, 1080m, DHJPAR0002988.

#### Paratypes.

6 ♀, 1 ♂ (BMNH, CNC, NMNH). COSTA RICA, ACG database codes:, 03-SRNP-23277, 03-SRNP-23282.

#### Description.

**Female.** Body color: body mostly dark except for some sternites which may be pale. Antenna color: scape and/or pedicel pale, flagellum dark. Coxae color (pro-, meso-, metacoxa): pale, pale, partially pale/partially dark. Femora color (pro-, meso-, metafemur): pale, pale, mostly pale but with dark area dorsally. Tibiae color (pro-, meso-, metatibia): pale, pale, anteriorly pale/posteriorly dark. Tegula and humeral complex color: both pale. Pterostigma color: dark with pale spot at base. Fore wing veins color: partially pigmented (a few veins may be dark but most are pale). Antenna length/body length: antenna shorter than body (head to apex of metasoma), not extending beyond anterior 0.7 metasoma length. Body in lateral view: not distinctly flattened dorso–ventrally. Body length (head to apex of metasoma): 2.5–2.6 mm. Fore wing length: 2.5–2.6 mm. Ocular–ocellar line/posterior ocellus diameter: 2.6 or more. Interocellar distance/posterior ocellus diameter: 1.4–1.6. Antennal flagellomerus 2 length/width: 2.0–2.2. Antennal flagellomerus 14 length/width: 1.4–1.6. Length of flagellomerus 2/length of flagellomerus 14: 1.7–1.9. Tarsal claws: simple. Metafemur length/width: 2.5 or less. Metatibia inner spur length/metabasitarsus length: 0.4–0.5. Anteromesoscutum: mostly with shallow, sparse punctures (separated by more than 2.0 × its maximum diameter). Mesoscutellar disc: mostly smooth. Number of pits in scutoscutellar sulcus: 9 or 10. Maximum height of mesoscutellum lunules/maximum height of lateral face of mesoscutellum: 0.2–0.3. Propodeum areola: completely defined by carinae, including transverse carina extending to spiracle. Propodeum background sculpture: partly sculptured, especially on anterior 0.5. Mediotergite 1 length/width at posterior margin: 4.1 or more. Mediotergite 1 shape: clearly narrowing towards posterior margin. Mediotergite 1 sculpture: more or less fully sculptured with longitudinal striation. Mediotergite 2 width at posterior margin/length: 2.4–2.7. Mediotergite 2 sculpture: mostly smooth. Outer margin of hypopygium: inflexible (without a folded, transparent, semi–desclerotized area); with no pleats visible. Ovipositor thickness: anterior width at most 2.0 × posterior width (beyond ovipositor constriction). Ovipositor sheaths length/metatibial length: 0.4–0.5. Length of fore wing veins r/2RS: 1.4–1.6. Length of fore wing veins 2RS/2M: 0.8 or less. Length of fore wing veins 2M/(RS+M)b: 1.1–1.3. Pterostigma length/width: 3.1–3.5. Point of insertion of vein r in pterostigma: about half way point length of pterostigma. Angle of vein r with fore wing anterior margin: clearly outwards, inclined towards fore wing apex. Shape of junction of veins r and 2RS in fore wing: strongly angulated, sometimes with a knob.

**Male.** As in female but with darker hind legs (metacoxa, metafemur and most of metatibia fully dark brown).

#### Molecular data.

Sequences in BOLD: 12, barcode compliant sequences: 12.

#### Biology/ecology.

Gregarious (Fig. 276). Hosts: Crambidae, four species of *Desmia* and one inexplicable record from *Hyalorista exuvialis*.

#### Distribution.

Costa Rica, ACG.

#### Comments.

This species is very close to *Apanteles robertoespinozai*, and the morphological characters used in the key may appear to be a continuum of variation within one single species. However, they are unambiguously two species as based on their barcodes and their host records.

#### Etymology.

We dedicate this species to Gloria Sihezar in recognition of her diligent efforts for the ACG Programa de Parataxónomos and Estación Biológica San Gerardo.

### Apanteles guadaluperodriguezae

Fernández-Triana sp. n.

http://zoobank.org/7476A0C0-5F6E-4908-99DE-51F5CF76632D

http://species-id.net/wiki/Apanteles_guadaluperodriguezae

Fig. 122

#### Type locality.

COSTA RICA, Alajuela, ACG, Sector Rincon Rain Forest, Estación Botarrama, 10.95991, -85.28298, 160m.

#### Holotype.

♀ in CNC. Specimen labels: 1. DHJPAR0038116. 2. Voucher: D.H.Janzen & W.Hallwachs, DB: http://janzen.sas.upenn.edu, Area de Conservación Guanacaste, COSTA RICA, 09-SRNP-68294.

#### Paratypes.

21 ♀, 3 ♂ (CNC, NMNH). COSTA RICA, ACG database codes: DHJPAR0035392, 09-SRNP-68294, 10-SRNP-71302.

#### Description.

**Female.** Body color: body mostly dark except for some sternites which may be pale. Antenna color: scape, pedicel, and flagellum pale. Coxae color (pro-, meso-, metacoxa): pale, dark, dark. Femora color (pro-, meso-, metafemur): pale, dark, dark. Tibiae color (pro-, meso-, metatibia): pale, pale, anteriorly pale/posteriorly dark. Tegula and humeral complex color: both pale. Pterostigma color: entirely pale or transparent, translucent. Fore wing veins color: mostly white or entirely transparent. Antenna length/body length: antenna very short, barely or not extending beyond mesosoma length. Body in lateral view: distinctly flattened dorso–ventrally. Body length (head to apex of metasoma): 2.0 mm or less. Fore wing length: 2.0 mm or less or 2.1–2.2 mm. Ocular–ocellar line/posterior ocellus diameter: 2.3–2.5. Interocellar distance/posterior ocellus diameter: 1.1–1.3. Antennal flagellomerus 2 length/width: 1.4–1.6. Antennal flagellomerus 14 length/width: 1.1–1.3. Length of flagellomerus 2/length of flagellomerus 14: 1.4–1.6. Tarsal claws: simple. Metafemur length/width: 2.6–2.7. Metatibia inner spur length/metabasitarsus length: 0.4–0.5. Anteromesoscutum: mostly with shallow, sparse punctures (separated by more than 2.0 × its maximum diameter). Mesoscutellar disc: mostly smooth. Number of pits in scutoscutellar sulcus: 7 or 8 or 9 or 10. Maximum height of mesoscutellum lunules/maximum height of lateral face of mesoscutellum: 0.4–0.5. Propodeum areola: completely defined by carinae, but only partial or absent transverse carina. Propodeum background sculpture: partly sculptured, especially on anterior 0.5. Mediotergite 1 length/width at posterior margin: 4.1 or more. Mediotergite 1 shape: clearly narrowing towards posterior margin. Mediotergite 1 sculpture: with some sculpture near lateral margins and/or posterior 0.2–0.4 of mediotergite. Mediotergite 2 width at posterior margin/length: 3.6–3.9. Mediotergite 2 sculpture: mostly smooth. Outer margin of hypopygium: with a medially folded, transparent, semi–desclerotized area; with 0–3 pleats visible. Ovipositor thickness: anterior width at most 2.0 × posterior width (beyond ovipositor constriction). Ovipositor sheaths length/metatibial length: 0.4–0.5. Length of fore wing veins r/2RS: 1.0 or less. Length of fore wing veins 2RS/2M: 0.9–1.0. Length of fore wing veins 2M/(RS+M)b: 1.1–1.3. Pterostigma length/width: 3.6 or more. Point of insertion of vein r in pterostigma: clearly beyond half way point length of pterostigma. Angle of vein r with fore wing anterior margin: clearly outwards, inclined towards fore wing apex. Shape of junction of veins r and 2RS in fore wing: distinctly but not strongly angled.

**Male.** As in female, but with long antenna.

#### Molecular data.

Sequences in BOLD: 3, barcode compliant sequences: 3.

#### Biology/ecology.

Gregarious. Host: Crambidae, *Piletosoma thialis*.

#### Distribution.

Costa Rica, ACG.

#### Etymology.

We dedicate this species to Guadalupe Rodríguez in recognition of her diligent efforts for the ACG Programa de Ecoturismo.

### Apanteles guillermopereirai

Fernández-Triana sp. n.

http://zoobank.org/8DD8BA96-2362-40CB-A13D-A57600BD5048

http://species-id.net/wiki/Apanteles_guillermopereirai

Figs 57 , 250

Apanteles Rodriguez08 (Smith et al. 2006). Interim name provided by the authors.

#### Type locality.

COSTA RICA, Guanacaste, ACG, Sector Santa Rosa, Bosque San Emilio, 300m, 10.84389, -85.61384.

#### Holotype.

♀ in CNC. Specimen labels: 1. COSTA RICA, Guanacaste, ACG, Sector Santa Rosa, Bosque San Emilio, 11/22/1993, gusaneros. 2. 93-SRNP-8055, Pellicia dimidiata, Ipomoea 13066.

#### Paratypes.

13 ♀, 5 ♂ (BMNH, CNC, INBIO, INHS, NMNH). COSTA RICA, ACG database codes: 93-SRNP-8055, 99-SRNP-2575, 02-SRNP-15073, 03-SRNP-1279, 04-SRNP-2263, 04-SRNP-41764, 04-SRNP-41765, 04-SRNP-46971,.

#### Description.

**Female.** Body color: body mostly dark except for some sternites which may be pale. Antenna color: scape, pedicel, and flagellum dark. Coxae color (pro-, meso-, metacoxa): dark, dark, dark. Femora color (pro-, meso-, metafemur): pale, pale, mostly pale but posterior 0.2 or less dark. Tibiae color (pro-, meso-, metatibia): pale, pale, mostly pale but with posterior 0.2 or less dark. Tegula and humeral complex color: both pale. Pterostigma color: dark with pale spot at base. Fore wing veins color: mostly dark (a few veins may be unpigmented). Antenna length/body length: antenna about as long as body (head to apex of metasoma); if slightly shorter, at least extending beyond anterior 0.7 metasoma length. Body in lateral view: not distinctly flattened dorso–ventrally. Body length (head to apex of metasoma): 2.5–2.6 mm or 2.7–2.8 mm. Fore wing length: 2.5–2.6 mm or 2.7–2.8 mm. Ocular–ocellar line/posterior ocellus diameter: 2.6 or more. Interocellar distance/posterior ocellus diameter: 1.4–1.6. Antennal flagellomerus 2 length/width: 2.3–2.5. Antennal flagellomerus 14 length/width: 1.4–1.6. Length of flagellomerus 2/length of flagellomerus 14: 2.0–2.2. Tarsal claws: simple or with single basal spine–like seta. Metafemur length/width: 3.0–3.1. Metatibia inner spur length/metabasitarsus length: 0.4–0.5. Anteromesoscutum: mostly with deep, dense punctures (separated by less than 2.0 × its maximum diameter). Mesoscutellar disc: with punctures near margins, central part mostly smooth. Number of pits in scutoscutellar sulcus: 9 or 10. Maximum height of mesoscutellum lunules/maximum height of lateral face of mesoscutellum: 0.6–0.7. Propodeum areola: completely defined by carinae, including transverse carina extending to spiracle. Propodeum background sculpture: partly sculptured, especially on anterior 0.5. Mediotergite 1 length/width at posterior margin: 2.9–3.1. Mediotergite 1 shape: mostly parallel–sided for 0.5–0.7 of its length, then narrowing posteriorly so mediotergite anterior width >1.1 × posterior width. Mediotergite 1 sculpture: with some sculpture near lateral margins and/or posterior 0.2–0.4 of mediotergite. Mediotergite 2 width at posterior margin/length: 2.0–2.3. Mediotergite 2 sculpture: with some sculpture, mostly near posterior margin. Outer margin of hypopygium: with a medially folded, transparent, semi–desclerotized area; with 0–3 pleats visible. Ovipositor thickness: anterior width 3.0–5.0 × posterior width (beyond ovipositor constriction). Ovipositor sheaths length/metatibial length: 0.8–0.9. Length of fore wing veins r/2RS: 2.0–2.2. Length of fore wing veins 2RS/2M: 1.4–1.6. Length of fore wing veins 2M/(RS+M)b: 0.7–0.8. Pterostigma length/width: 3.1–3.5. Point of insertion of vein r in pterostigma: about half way point length of pterostigma. Angle of vein r with fore wing anterior margin: more or less perpendicular to fore wing margin. Shape of junction of veins r and 2RS in fore wing: distinctly but not strongly angled.

**Male.** As in female, but with darker coloration, especially on metafemur.

#### Molecular data.

Sequences in BOLD: 14, barcode compliant sequences: 9.

#### Biology/ecology.

Gregarious (Fig. 250). Host: Hesperiidae, *Nisoniades godma*.

#### Distribution.

Costa Rica, ACG.

#### Comments.

The holotype specimen has a short barcode but matches all the other specimens in morphology, host caterpillar and remainder of its barcode.

#### Etymology.

We dedicate this species to Guillermo Pereira in recognition of his diligent efforts for the ACG Programa de Parataxónomos and Estación Biológica Santa Rosa of ACG.

### Apanteles harryramirezi

Fernández-Triana sp. n.

http://zoobank.org/11F3C981-CE52-4B81-86AB-41F7D61B9EFF

http://species-id.net/wiki/Apanteles_harryramirezi

Figs 58 , 251

Apanteles Rodriguez72 (Smith et al. 2006). Interim name provided by the authors.

#### Type locality.

COSTA RICA, Guanacaste, ACG, Sector Cacao, Gongora Bananal, 600m, 10.88919, -85.47609.

#### Holotype.

♀ in CNC. Specimen labels: 1. DHJPAR0002982. 2. COSTA RICA, Guanacaste, ACG, Sector Cacao, Gongora Bananal, 27.ii.2004, 600m, 10.88919, -85.47609, 04-SRNP-45081.

#### Paratypes.

3 ♀, 2 ♂ (CNC, NMNH). COSTA RICA, ACG database codes: DHJPAR0004176, 92-SRNP-4988, 93-SRNP-3544, 04-SRNP-45081.

#### Description.

**Female.** Body color: body mostly dark except for some sternites which may be pale. Antenna color: scape, pedicel, and flagellum dark. Coxae color (pro-, meso-, metacoxa): pale, pale, pale. Femora color (pro-, meso-, metafemur): pale, pale, pale. Tibiae color (pro-, meso-, metatibia): pale, pale, pale. Tegula and humeral complex color: both pale. Pterostigma color: mostly pale and/or transparent, with thin dark borders. Fore wing veins color: mostly white or entirely transparent. Antenna length/body length: antenna shorter than body (head to apex of metasoma), not extending beyond anterior 0.7 metasoma length. Body in lateral view: not distinctly flattened dorso–ventrally. Body length (head to apex of metasoma): 2.3–2.4 mm or 2.5–2.6 mm. Fore wing length: 2.5–2.6 mm or 2.7–2.8 mm. Ocular–ocellar line/posterior ocellus diameter: 2.3–2.5. Interocellar distance/posterior ocellus diameter: 1.7–1.9. Antennal flagellomerus 2 length/width: 2.3–2.5. Antennal flagellomerus 14 length/width: 1.1–1.3. Length of flagellomerus 2/length of flagellomerus 14: 2.0–2.2. Tarsal claws: simple. Metafemur length/width: 2.8–2.9. Metatibia inner spur length/metabasitarsus length: 0.4–0.5. Anteromesoscutum: mostly smooth or with shallow sparse punctures, except for lateral and/or posterior margins where it has deeper and/or denser punctures. Mesoscutellar disc: with punctures near margins, central part mostly smooth. Number of pits in scutoscutellar sulcus: 9 or 10. Maximum height of mesoscutellum lunules/maximum height of lateral face of mesoscutellum: 0.6–0.7. Propodeum areola: completely defined by carinae, but only partial or absent transverse carina. Propodeum background sculpture: mostly sculptured. Mediotergite 1 length/width at posterior margin: 2.6–2.8. Mediotergite 1 shape: mostly parallel–sided for 0.5–0.7 of its length, then narrowing posteriorly so mediotergite anterior width >1.1 × posterior width. Mediotergite 1 sculpture: mostly sculptured, excavated area centrally with transverse striation inside and/or a polished knob centrally on posterior margin of mediotergite. Mediotergite 2 width at posterior margin/length: 3.6–3.9. Mediotergite 2 sculpture: mostly smooth. Outer margin of hypopygium: with a medially folded, transparent, semi–desclerotized area; with 0–3 pleats visible. Ovipositor thickness: anterior width 3.0–5.0 × posterior width (beyond ovipositor constriction). Ovipositor sheaths length/metatibial length: 1.0–1.1. Length of fore wing veins r/2RS: 2.3 or more. Length of fore wing veins 2RS/2M: 0.9–1.0. Length of fore wing veins 2M/(RS+M)b: 0.7–0.8. Pterostigma length/width: 3.1–3.5. Point of insertion of vein r in pterostigma: clearly beyond half way point length of pterostigma. Angle of vein r with fore wing anterior margin: more or less perpendicular to fore wing margin. Shape of junction of veins r and 2RS in fore wing: distinctly but not strongly angled.

**Male.** Similar to female, except for darker legs.

#### Molecular data.

Sequences in BOLD: 5, barcode compliant sequences: 5.

#### Biology/ecology.

Gregarious (Fig. 251). Hosts: Hesperiidae, *Anisochoria polysticta*, *Timochreon satyrus*.

#### Distribution.

Costa Rica, ACG.

#### Comments.

One female (deposited in the CNC) has a much darker coloration, especially on legs.

#### Etymology.

We dedicate this species to Harry Ramírez recognition of his diligent efforts for the ACG Programa de Parataxónomos and Estación Biológica Cacao.

### Apanteles hazelcambroneroae

Fernández-Triana sp. n.

http://zoobank.org/8C6793F4-43E8-4792-9750-483330228637

http://species-id.net/wiki/Apanteles_hazelcambroneroae

Figs 202 , 312

Apanteles Rodriguez54 (Smith et al. 2006). Interim name provided by the authors.

#### Type locality.

COSTA RICA, Guanacaste, ACG, Sector Horizontes, Bejuco, 180m, 10.76712, -85.59663.

#### Holotype.

♀ in CNC. Specimen labels: 1. DHJPAR0003982. 2. COSTA RICA, Guanacaste, Area de Conservacion Guanacaste, Sector Horizontes, Bejuco, 21-I-2010, gusaneros. 3. 00-SRNP-6072, Cephise aelius, On Combretum farinsoum.

#### Paratypes.

27 ♀, 19 ♂ (BMNH, CNC, INBIO, INHS, NMNH). COSTA RICA: ACG database codes: DHJPAR0001582, DHJPAR0001586, DHJPAR0002707, DHJPAR0003020, DHJPAR0003038, DHJPAR0003042, DHJPAR0003049, DHJPAR0003982, DHJPAR0005233, DHJPAR0005237, DHJPAR0005245, DHJPAR0005249, DHJPAR0005292, DHJPAR0005301, 04-SRNP-21566.

#### Description.

**Female.** Metatibia color (outer face): entirely or mostly (>0.7 metatibia length) dark brown to black, with yellow to white coloration usually restricted to anterior 0.2 or less. Fore wing veins color: veins C+Sc+R and R1 with brown coloration restricted narrowly to borders, interior area of those veins and pterostigma (and sometimes veins r, 2RS and 2M) transparent or white; other veins mostly transparent. Antenna length/body length: antenna about as long as body (head to apex of metasoma); if slightly shorter, at least extending beyond anterior 0.7 metasoma length. Body length (head to apex of metasoma): 2.0 mm or less or 2.1–2.2 mm. Fore wing length: 2.1–2.2 mm or 2.3–2.4 mm. Metafemur length/width: 2.6–2.7 or 2.8–2.9. Mediotergite 1 length/width at posterior margin: 2.3–2.4, 2.5–2.6, 2.7–2.8, rarely 2.1–2.2. Mediotergite 1 maximum width/width at posterior margin: 1.2–1.3 or 1.4–1.5. Ovipositor sheaths length/metafemur length: 0.9, rarely 0.8. Ovipositor sheaths length/metatibia length: 0.7 or 0.8.

#### Molecular data.

Sequences in BOLD: 24, barcode compliant sequences: 22.

#### Biology/ecology.

Gregarious (Fig. 312). Hosts: Hesperiidae, *Cephise aelius*.

#### Distribution.

Costa Rica, ACG.

#### Etymology.

We dedicate this species to Hazel Cambronero in recognition of her diligent efforts for the ACG Parataxonomist Program and BioLep, the inventory of the adult ACG Lepidoptera.

### Apanteles hectorsolisi

Fernández-Triana sp. n.

http://zoobank.org/BBBD59A2-1C70-4E79-BBA1-A1B0E3C55304

http://species-id.net/wiki/Apanteles_hectorsolisi

Figs 124 , 289

#### Type locality.

COSTA RICA, Alajuela, ACG, Sector Rincon Rain Forest, Sendero Venado, 420m, 10.89678, -85.27001.

#### Holotype.

♀ in CNC. Specimen labels: 1. DHJPAR0040433. 2. COSTA RICA, Alajuela, ACG, Sector Rincon Rain Forest, Sendero Venado, 29.vii.2010, 10.89678°N, -85.27001°W, 420m, DHJPAR0040433. 3. Voucher: D.H.Janzen & W.Hallwachs, DB: http://janzen.sas.upenn.edu, Area de Conservación Guanacaste, COSTA RICA, 10-SRNP-42701.

#### Paratypes.

2 ♀, 2 ♂ (CNC, NMNH). Same labels as holotype.

#### Description.

**Female.** Body color: body mostly dark except for some sternites which may be pale. Antenna color: scape, pedicel, and flagellum dark. Coxae color (pro-, meso-, metacoxa): dark, dark, dark. Femora color (pro-, meso-, metafemur): pale, anteriorly dark/posteriorly pale, dark. Tibiae color (pro-, meso-, metatibia): pale, pale, anteriorly pale/posteriorly dark. Tegula and humeral complex color: tegula pale, humeral complex half pale/half dark. Pterostigma color: dark with pale spot at base. Fore wing veins color: mostly dark (a few veins may be unpigmented). Antenna length/body length: antenna shorter than body (head to apex of metasoma), not extending beyond anterior 0.7 metasoma length. Body in lateral view: not distinctly flattened dorso–ventrally. Body length (head to apex of metasoma): 2.5–2.6 mm. Fore wing length: 2.7–2.8 mm. Ocular–ocellar line/posterior ocellus diameter: 2.3–2.5. Interocellar distance/posterior ocellus diameter: 1.7–1.9. Antennal flagellomerus 2 length/width: 2.3–2.5. Antennal flagellomerus 14 length/width: 1.4–1.6. Length of flagellomerus 2/length of flagellomerus 14: 2.0–2.2. Tarsal claws: simple. Metafemur length/width: 2.8–2.9. Metatibia inner spur length/metabasitarsus length: 0.4–0.5. Anteromesoscutum: mostly with deep, dense punctures (separated by less than 2.0 × its maximum diameter). Mesoscutellar disc: mostly smooth. Number of pits in scutoscutellar sulcus: 9 or 10. Maximum height of mesoscutellum lunules/maximum height of lateral face of mesoscutellum: 0.4–0.5. Propodeum areola: partially defined by carinae on posterior 0.3–0.5 of its length, widely open anteriorly. Propodeum background sculpture: partly sculptured, especially on anterior 0.5. Mediotergite 1 length/width at posterior margin: 4.1 or more. Mediotergite 1 shape: mostly parallel–sided for 0.5–0.7 of its length, then narrowing posteriorly so mediotergite anterior width >1.1 × posterior width. Mediotergite 1 sculpture: mostly smooth. Mediotergite 2 width at posterior margin/length: 2.4–2.7. Mediotergite 2 sculpture: mostly smooth. Outer margin of hypopygium: with a wide, medially folded, transparent, semi–desclerotized area; usually with 4 or more pleats. Ovipositor thickness: about same width throughout its length. Ovipositor sheaths length/metatibial length: 0.8–0.9. Length of fore wing veins r/2RS: 1.7–1.9. Length of fore wing veins 2RS/2M: 1.1–1.3. Length of fore wing veins 2M/(RS+M)b: 0.9–1.0. Pterostigma length/width: 2.6–3.0. Point of insertion of vein r in pterostigma: about half way point length of pterostigma. Angle of vein r with fore wing anterior margin: clearly outwards, inclined towards fore wing apex. Shape of junction of veins r and 2RS in fore wing: distinctly but not strongly angled.

**Male.** Like female.

#### Molecular data.

Sequences in BOLD: 1, barcode compliant sequences: 1.

#### Biology/ecology.

Gregarious (Fig. 289). Host: Riodinidae, *Argyrogrammama venilia crocea*.

#### Distribution.

Costa Rica, ACG.

#### Comments.

This species is characterized by T1 mostly parallel-sided for 0.7 of its length, then strongly narrowing posteriorly, so T1 length at least 3.0 × its width at posterior margin. It is the only Mesoamerican species with that T1 shape and the combination of tegula and humeral complex of different color, and pterostigma brown.

#### Etymology.

We dedicate this species to Héctor Solís in recognition of his diligent efforts for the ACG Programa Forestal.

### Apanteles humbertolopezi

Fernández-Triana sp. n.

http://zoobank.org/90092F8E-3464-40D3-99D8-FB3B04B48FCB

http://species-id.net/wiki/Apanteles_humbertolopezi

Fig. 125

#### Type locality.

COSTA RICA, Alajuela, ACG, Sector San Cristobal, Rio Blanco Abajo, 500m, 10.90037, -85.37254.

#### Holotype.

♀ in CNC. Specimen labels: 1. San Gerardo: Rio Blanco Abajo, Date: 21-27 Aug. 2007. 2. DHJPAR0024860.

#### Description.

**Female.** Body color: body mostly dark except for some sternites which may be pale. Antenna color: scape, pedicel, and flagellum dark. Coxae color (pro-, meso-, metacoxa): dark, dark, dark. Femora color (pro-, meso-, metafemur): anteriorly dark/posteriorly pale, dark, dark. Tibiae color (pro-, meso-, metatibia): pale, dark, dark. Tegula and humeral complex color: both dark. Pterostigma color: dark with pale spot at base. Fore wing veins color: mostly dark (a few veins may be unpigmented). Antenna length/body length: antenna about as long as body (head to apex of metasoma); if slightly shorter, at least extending beyond anterior 0.7 metasoma length. Body in lateral view: not distinctly flattened dorso–ventrally. Body length (head to apex of metasoma): 2.1–2.2 mm. Fore wing length: 2.3–2.4 mm. Ocular–ocellar line/posterior ocellus diameter: 2.3–2.5. Interocellar distance/posterior ocellus diameter: 1.7–1.9. Antennal flagellomerus 2 length/width: 2.6–2.8. Tarsal claws: with single basal spine–like seta. Metafemur length/width: 3.4–3.5. Metatibia inner spur length/metabasitarsus length: 0.4–0.5. Anteromesoscutum: mostly with deep, dense punctures (separated by less than 2.0 × its maximum diameter). Mesoscutellar disc: mostly smooth. Number of pits in scutoscutellar sulcus: 9 or 10. Maximum height of mesoscutellum lunules/maximum height of lateral face of mesoscutellum: 0.4–0.5. Propodeum areola: completely defined by carinae, including transverse carina extending to spiracle. Propodeum background sculpture: mostly sculptured. Mediotergite 1 length/width at posterior margin: 1.4–1.6. Mediotergite 1 shape: clearly widening towards posterior margin. Mediotergite 1 sculpture: mostly sculptured, excavated area centrally with transverse striation inside and/or a polished knob centrally on posterior margin of mediotergite. Mediotergite 2 width at posterior margin/length: 4.0–4.3. Mediotergite 2 sculpture: more or less fully sculptured, with longitudinal striation. Outer margin of hypopygium: with a wide, medially folded, transparent, semi–desclerotized area; usually with 4 or more pleats. Ovipositor thickness: about same width throughout its length. Ovipositor sheaths length/metatibial length: 0.8–0.9. Length of fore wing veins r/2RS: 1.1–1.3. Length of fore wing veins 2RS/2M: 1.4–1.6. Length of fore wing veins 2M/(RS+M)b: 0.7–0.8. Pterostigma length/width: 3.1–3.5. Point of insertion of vein r in pterostigma: about half way point length of pterostigma. Angle of vein r with fore wing anterior margin: more or less perpendicular to fore wing margin. Shape of junction of veins r and 2RS in fore wing: distinctly but not strongly angled.

**Male.** Unknown.

#### Molecular data.

Sequences in BOLD: 2, barcode compliant sequences: 2.

#### Biology/ecology.

Solitary. Host: malaise-trapped and Elachistidae, *Antaeotricha* sp. with ACG code 10-SRNP-55263.

#### Distribution.

Costa Rica, ACG.

#### Comments.

The vannal lobe is more like that of many species of *Dolichogenidea*, but the species is described as *Apanteles* based on other information.

#### Etymology.

We dedicate this species to Humberto López in recognition of his diligent efforts for the ACG Programa de Sectores.

### Apanteles impiger

Muesebeck, 1958

http://species-id.net/wiki/Apanteles_impiger

Fig. 75

Apanteles impiger Muesebeck, 1958: 437.

#### Type locality.

PUERTO RICO, Mayaguez.

#### Holotype.

♀, NMNH (examined).

#### Description.

**Female.** Body color: body mostly dark except for some sternites which may be pale. Antenna color: scape, pedicel, and flagellum dark. Coxae color (pro-, meso-, metacoxa): pale, pale, partially pale/partially dark. Femora color (pro-, meso-, metafemur): pale, pale, pale. Tibiae color (pro-, meso-, metatibia): pale, pale, mostly pale but with posterior 0.2 or less dark. Tegula and humeral complex color: both pale. Pterostigma color: entirely pale or transparent, translucent. Fore wing veins color: partially pigmented (a few veins may be dark but most are pale). Antenna length/body length: antenna about as long as body (head to apex of metasoma); if slightly shorter, at least extending beyond anterior 0.7 metasoma length. Body in lateral view: not distinctly flattened dorso–ventrally. Body length (head to apex of metasoma): 2.3–2.4 mm. Fore wing length: 2.5–2.6 mm. Ocular–ocellar line/posterior ocellus diameter: 2.0–2.2. Interocellar distance/posterior ocellus diameter: 2.0–2.2. Antennal flagellomerus 2 length/width: 2.9–3.1. Antennal flagellomerus 14 length/width: 1.1–1.3. Length of flagellomerus 2/length of flagellomerus 14: 2.6–2.8. Tarsal claws: with single basal spine–like seta. Metafemur length/width: 3.2–3.3. Metatibia inner spur length/metabasitarsus length: 0.4–0.5. Anteromesoscutum: mostly with shallow, sparse punctures (separated by more than 2.0 × its maximum diameter). Mesoscutellar disc: mostly smooth. Number of pits in scutoscutellar sulcus: 11 or 12. Maximum height of mesoscutellum lunules/maximum height of lateral face of mesoscutellum: 0.4–0.5. Propodeum areola: completely defined by carinae, including transverse carina extending to spiracle. Propodeum background sculpture: mostly sculptured. Mediotergite 1 length/width at posterior margin: 2.9–3.1. Mediotergite 1 shape: mostly parallel–sided for 0.5–0.7 of its length, then narrowing posteriorly so mediotergite anterior width >1.1 × posterior width. Mediotergite 1 sculpture: mostly sculptured, excavated area centrally with transverse striation inside and/or a polished knob centrally on posterior margin of mediotergite. Mediotergite 2 width at posterior margin/length: 3.6–3.9. Mediotergite 2 sculpture: with some sculpture, mostly near posterior margin. Outer margin of hypopygium: with a wide, medially folded, transparent, semi–desclerotized area; usually with 4 or more pleats. Ovipositor thickness: about same width throughout its length. Ovipositor sheaths length/metatibial length: 0.8–0.9. Length of fore wing veins r/2RS: 2.0–2.2 or 2.3 or more. Length of fore wing veins 2RS/2M: 1.1–1.3. Length of fore wing veins 2M/(RS+M)b: 0.7–0.8. Pterostigma length/width: 3.6 or more. Point of insertion of vein r in pterostigma: about half way point length of pterostigma. Angle of vein r with fore wing anterior margin: more or less perpendicular to fore wing margin. Shape of junction of veins r and 2RS in fore wing: distinctly but not strongly angled.

#### Molecular data.

No molecular data available for this species.

#### Biology/ecology.

Solitary, white cocoons (Muesebeck 1921 and 1958). Hosts: Crambidae, *Diaphania hyalinata*.

#### Distribution.

Cuba, Puerto Rico; there is no suggestion that this wasp occurs in ACG, though there are numerous species of *Diaphania* that breed in ACG.

### Apanteles inesolisae

Fernández-Triana sp. n.

http://zoobank.org/6C93BB5D-1552-484E-BE22-F16188F8CBAC

http://species-id.net/wiki/Apanteles_inesolisae

Figs 182 , 313

Apanteles Rodriguez58 (Smith et al. 2006). Interim name provided by the authors.

#### Type locality.

COSTA RICA, Alajuela, ACG, Sector Pitilla, Pasmompa, 440m, 11.01926, -85.40997.

#### Holotype.

♀ in CNC. Specimen labels: 1. DHJPAR0002706. 2. COSTA RICA, Guanacaste, ACG, Sector Pitilla, Pasmompa, 11.iii.2004, 440m, 11.01926, -85.40997, 04-SRNP-31228.

#### Paratypes.

17 ♀, 5 ♂ (BMNH, CNC, INBIO, INHS, NMNH). COSTA RICA: ACG database codes: DHJPAR0003003, 02-SRNP-5614, 03-SRNP-21916, 04-SRNP-31228.

#### Description.

**Female.** Metatibia color (outer face): entirely or mostly (>0.7 metatibia length) dark brown to black, with yellow to white coloration usually restricted to anterior 0.2 or less. Fore wing veins color: veins C+Sc+R and R1 mostly brown; usually veins r, 2RS, 2M, (RS+M)b, 1CU, 2Cua, and 1m–cu partially brown; interior area of other veins, and at least part of pterostigma, usually light brown or yellowish–white. Antenna length/body length: antenna shorter than body (head to apex of metasoma), not extending beyond anterior 0.7 metasoma length, rarely antenna about as long as body (head to apex of metasoma); if slightly shorter, at least extending beyond anterior 0.7 metasoma length. Body length (head to apex of metasoma): 2.0 mm or less or 2.3–2.4 mm. Fore wing length: 2.1–2.2 mm or 2.5–2.6 mm. Metafemur length/width: 2.6–2.7, 2.8–2.9 or 3.0–3.1. Mediotergite 1 length/width at posterior margin: 2.3–2.4. Mediotergite 1 maximum width/width at posterior margin: 1.4–1.5. Ovipositor sheaths length/metafemur length: 0.9, 1.0 or 1.1. Ovipositor sheaths length/metatibia length: 0.8 or 0.9.

#### Molecular data.

Sequences in BOLD: 10, barcode compliant sequences: 10.

#### Biology/ecology.

Gregarious (Fig. 313). Hosts: Hesperiidae, *Telemiades antiope*, *Telemiades fides*.

#### Distribution.

Costa Rica, ACG.

#### Etymology.

We dedicate this species to Inés Solís in recognition of her diligent efforts for ACG and for the accounting office in INBio, Costa Rica’s Instituto Nacional de Biodiversidad.

### Apanteles insularis

Muesebeck, 1921

http://species-id.net/wiki/Apanteles_insularis

Fig. 158

Urogaster grenadensis Ashmead, 1900: 285.
Apanteles grenadensis (Ashmead). Transferred by Szépligeti (1904: 110), but that name was preoccupied by *Apanteles grenadensis* Ashmead, 1900: 277.
Apanteles insularis Muesebeck, 1921: 514. New replacement name.

#### Type locality.

GRENADA, Balthazar.

#### Holotype.

♀, BMNH (examined photos of the holotype).

#### Molecular data.

No molecular data available for this species.

#### Biology/ecology.

No host known.

#### Distribution.

Grenada, St. Vincent. There is no suggestion that this species occurs in ACG.

#### Comments.

The placement of *insularis* within this group is only based on examination of photos of the holotype. The holotype has a body length of 2.4 mm and fore wing length of 2.7 mm (Gavin Broad, personal communication), as well as the tegula and the humeral complex are pale.

### Apanteles irenecarrilloi

Fernández-Triana sp. n.

http://zoobank.org/D182A22A-BF57-4324-98DD-E5094A61B04D

http://species-id.net/wiki/Apanteles_irenecarrilloi

Figs 27 , 229

#### Type locality.

COSTA RICA, Alajuela, ACG, Sector Rincon Rain Forest, San Lucas, 320m, 10.91847, -85.30338.

#### Holotype.

♀ in CNC. Specimen labels: 1. DHJPAR0045190. 2. COSTA RICA, Guanacaste, ACG, Sector Rincon Rain Forest, San Lucas, 30.vi.2011, 10.91847, -85.30338, 320m, DHJPAR0045190. 3. Voucher: D.H.Janzen & W.Hallwachs, DB: http://janzen.sas.upenn.edu, Area de Conservación Guanacaste, COSTA RICA, 11-SRNP-43138.

#### Paratypes.

1 ♀ (CNC). COSTA RICA, ACG database codes: DHJPAR0043146.

#### Description.

**Female.** Body color: body mostly dark except for some sternites which may be pale. Antenna color: scape, pedicel, and flagellum dark. Coxae color (pro-, meso-, metacoxa): dark, dark, dark. Femora color (pro-, meso-, metafemur): anteriorly dark/posteriorly pale, dark, dark. Tibiae color (pro-, meso-, metatibia): pale, anteriorly pale/posteriorly dark, anteriorly pale/posteriorly dark. Tegula and humeral complex color: tegula pale, humeral complex half pale/half dark. Pterostigma color: mostly pale and/or transparent, with thin dark borders. Fore wing veins color: partially pigmented (a few veins may be dark but most are pale). Antenna length/body length: antenna about as long as body (head to apex of metasoma); if slightly shorter, at least extending beyond anterior 0.7 metasoma length. Body in lateral view: not distinctly flattened dorso–ventrally. Body length (head to apex of metasoma): 2.3–2.4 mm. Fore wing length: 2.5–2.6 mm. Ocular–ocellar line/posterior ocellus diameter: 2.3–2.5. Interocellar distance/posterior ocellus diameter: 1.7–1.9. Antennal flagellomerus 2 length/width: 2.9–3.1. Antennal flagellomerus 14 length/width: 1.7–1.9. Length of flagellomerus 2/length of flagellomerus 14: 2.0–2.2. Tarsal claws: simple (?). Metafemur length/width: 2.8–2.9. Metatibia inner spur length/metabasitarsus length: 0.4–0.5. Anteromesoscutum: mostly with shallow, dense punctures (separated by less than 2.0 × its maximum diameter). Mesoscutellar disc: mostly smooth. Number of pits in scutoscutellar sulcus: 9 or 10. Maximum height of mesoscutellum lunules/maximum height of lateral face of mesoscutellum: 0.8 or more. Propodeum areola: completely defined by carinae, including transverse carina extending to spiracle. Propodeum background sculpture: partly sculptured, especially on anterior 0.5. Mediotergite 1 length/width at posterior margin: 2.0–2.2 or 2.3–2.5. Mediotergite 1 shape: mostly parallel–sided for 0.5–0.7 of its length, then narrowing posteriorly so mediotergite anterior width >1.1 × posterior width. Mediotergite 1 sculpture: mostly sculptured, excavated area centrally with transverse striation inside and/or a polished knob centrally on posterior margin of mediotergite. Mediotergite 2 width at posterior margin/length: 3.6–3.9 or 4.0–4.3. Mediotergite 2 sculpture: mostly smooth. Outer margin of hypopygium: with a wide, medially folded, transparent, semi–desclerotized area; usually with 4 or more pleats. Ovipositor thickness: about same width throughout its length. Ovipositor sheaths length/metatibial length: 1.2–1.3. Length of fore wing veins r/2RS: 1.7–1.9. Length of fore wing veins 2RS/2M: 1.4–1.6. Length of fore wing veins 2M/(RS+M)b: 0.7–0.8. Pterostigma length/width: 3.6 or more. Point of insertion of vein r in pterostigma: about half way point length of pterostigma. Angle of vein r with fore wing anterior margin: more or less perpendicular to fore wing margin. Shape of junction of veins r and 2RS in fore wing: strongly angulated, sometimes with a knob.

**Male.** Unknown.

#### Molecular data.

Sequences in BOLD: 3, barcode compliant sequences: 3.

#### Biology/ecology.

Solitary (Fig. 229). Host: Crambidae, *Asturodes fimbriauralis* DHJ03.

#### Distribution.

Costa Rica, ACG.

#### Etymology.

We dedicate this species to Irene Carrillo in recognition of her diligent efforts for the ACG Programa del Comedor Santa Rosa.

### Apanteles isaacbermudezi

Fernández-Triana sp. n.

http://zoobank.org/9FB63DF1-B8BA-471B-AE84-7E50F3F1F355

http://species-id.net/wiki/Apanteles_isaacbermudezi

Fig. 9

#### Type locality.

COSTA RICA, Alajuela, ACG, Sector Pitilla, Leonel, 510m, 10.99637, -85.40195.

#### Holotype.

♀ in CNC. Specimen labels: 1. DHJPAR0045253. 2. COSTA RICA, Guanacaste, ACG, Sector Pitilla, Leonel, 10.99637N, 85.40195W, 23.vii.2011, 510m, DHJPAR0045253. 3. Voucher: D.H.Janzen & W.Hallwachs, DB: http://janzen.sas.upenn.edu, Area de Conservación Guanacaste, COSTA RICA, 11-SRNP-71609.

#### Paratypes.

3 ♀ (CNC, NMNH). COSTA RICA, ACG database codes: 11-SRNP-71609.

#### Description.

**Female.** Body color: body mostly dark except for some sternites which may be pale. Antenna color: scape, pedicel, and flagellum dark. Coxae color (pro-, meso-, metacoxa): dark, dark, dark. Femora color (pro-, meso-, metafemur): anteriorly dark/posteriorly pale, dark, dark. Tibiae color (pro-, meso-, metatibia): pale, anteriorly pale/posteriorly dark, anteriorly pale/posteriorly dark. Tegula and humeral complex color: tegula pale, humeral complex half pale/half dark. Pterostigma color: mostly pale and/or transparent, with thin dark borders. Fore wing veins color: partially pigmented (a few veins may be dark but most are pale). Antenna length/body length: antenna about as long as body (head to apex of metasoma); if slightly shorter, at least extending beyond anterior 0.7 metasoma length. Body in lateral view: not distinctly flattened dorso–ventrally. Body length (head to apex of metasoma): 2.5–2.6 mm. Fore wing length: 2.7–2.8 mm. Ocular–ocellar line/posterior ocellus diameter: 2.3–2.5. Interocellar distance/posterior ocellus diameter: 1.7–1.9. Antennal flagellomerus 2 length/width: 2.3–2.5. Tarsal claws: simple (?). Metafemur length/width: 3.0–3.1. Metatibia inner spur length/metabasitarsus length: 0.4–0.5. Anteromesoscutum: mostly with deep, dense punctures (separated by less than 2.0 × its maximum diameter). Mesoscutellar disc: mostly smooth. Number of pits in scutoscutellar sulcus: 9 or 10. Maximum height of mesoscutellum lunules/maximum height of lateral face of mesoscutellum: 0.6–0.7. Propodeum areola: completely defined by carinae, including transverse carina extending to spiracle. Propodeum background sculpture: partly sculptured, especially on anterior 0.5. Mediotergite 1 length/width at posterior margin: 1.7–1.9. Mediotergite 1 shape: more or less parallel–sided. Mediotergite 1 sculpture: with some sculpture near lateral margins and/or posterior 0.2–0.4 of mediotergite. Mediotergite 2 width at posterior margin/length: 3.2–3.5. Mediotergite 2 sculpture: mostly smooth. Outer margin of hypopygium: with a wide, medially folded, transparent, semi–desclerotized area; usually with 4 or more pleats. Ovipositor thickness: about same width throughout its length (?). Ovipositor sheaths length/metatibial length: 1.0–1.1. Length of fore wing veins r/2RS: 1.7–1.9. Length of fore wing veins 2RS/2M: 1.1–1.3. Length of fore wing veins 2M/(RS+M)b: 0.9–1.0. Pterostigma length/width: 3.1–3.5. Point of insertion of vein r in pterostigma: about half way point length of pterostigma. Angle of vein r with fore wing anterior margin: more or less perpendicular to fore wing margin. Shape of junction of veins r and 2RS in fore wing: distinctly but not strongly angled.

**Male.** Unknown.

#### Molecular data.

Sequences in BOLD: 1, barcode compliant sequences: 1.

#### Biology/ecology.

Gregarious. Hosts: Elachistidae, *Stenoma patens*.

#### Distribution.

Costa Rica, ACG.

#### Etymology.

We dedicate this species to Isaac Bermúdez in recognition of his diligent efforts for the ACG Programa de Sectores.

### Apanteles isidrochaconi

Fernández-Triana sp. n.

http://zoobank.org/D052D494-98F0-4015-80BD-33A9BE5C2495

http://species-id.net/wiki/Apanteles_isidrochaconi

Fig. 127

#### Type locality.

COSTA RICA, Alajuela, ACG, Sector Rincon Rain Forest, Vado Rio Francia, 400m, 10.90093, -85.28915.

#### Holotype.

♀ in CNC. Specimen labels: 1. DHJPAR0025426. 2. COSTA RICA, Alajuela, ACG, Sector Rincon Rain Forest, Vado Rio Francia, 5-11.vii.2007, 10.90093°N, 85.28915°W, 400m, DHJPAR0025426. 3. Caribe: Rio Francia, Date: 5–11 Jul 07.

#### Description.

**Female.** Body color: head dark, mesosoma dark with parts of axillar complex pale, metasoma with some mediotergites, most laterotergites, sternites, and/or hypopygium pale. Antenna color: scape, pedicel, and flagellum dark. Coxae color (pro-, meso-, metacoxa): pale, pale, pale. Femora color (pro-, meso-, metafemur): pale, pale, pale. Tibiae color (pro-, meso-, metatibia): pale, pale, pale. Tegula and humeral complex color: both pale. Pterostigma color: dark with pale spot at base. Fore wing veins color: mostly dark (a few veins may be unpigmented). Antenna length÷body length: antenna shorter than body (head to apex of metasoma), not extending beyond anterior 0.7 metasoma length. Body in lateral view: not distinctly flattened dorso-ventrally. Body length (head to apex of metasoma): 4.0 mm or more. Fore wing length: 4.0 mm or more. Ocular-ocellar line÷posterior ocellus diameter: 1.4–1.6. Interocellar distance÷posterior ocellus diameter: 1.4–1.6. Antennal flagellomere 2 length÷width: 2.3–2.5. Antennal flagellomere 14 length÷width: 1.1–1.3. Length of flagellomere 2÷length of flagellomere 14: 2.3–2.5. Tarsal claws: with single basal spine-like seta. Metafemur length÷width: 3.4–3.5. Anteromesoscutum: mostly with deep, dense punctures (separated by less than 2.0 x its maximum diameter). Mesoscutellar disc: mostly smooth. Number of pits in scutoscutellar sulcus: 5 or 6. Maximum height of mesoscutellum lunules÷maximum height of lateral face of mesoscutellum: 0.6–0.7. Propodeum areola: completely defined by carinae, but only partial or absent transverse carina. Propodeum background sculpture: partly sculptured, especially on anterior 0.5. Mediotergite 1 length÷width at posterior margin: 2.6–2.8. Mediotergite 1 shape: mostly parallel-sided for 0.5–0.7 of its length, then narrowing posteriorly so mediotergite anterior width >1.1 x posterior width. Mediotergite 1 sculpture: mostly sculptured, excavated area centrally with transverse striation inside and/or a polished knob centrally on posterior margin of mediotergite. Mediotergite 2 width at posterior margin÷length: 3.6–3.9. Mediotergite 2 sculpture: mostly smooth. Outer margin of hypopygium: with a wide, medially folded, transparent, semi-desclerotized area; usually with 4 or more pleats. Ovipositor thickness: about same width throughout its length. Ovipositor sheaths length÷metatibial length: 1.2–1.3. Length of fore wing veins r÷2RS: 1.7–1.9. Length of fore wing veins 2RS÷2M: 1.7–1.8. Length of fore wing veins 2M÷(RS+M)b: 0.7–0.8. Pterostigma length÷width: 2.1–2.5. Point of insertion of vein r in pterostigma: clearly beyond half way point length of pterostigma. Angle of vein r with fore wing anterior margin: clearly outwards, inclined towards fore wing apex. Shape of junction of veins r and 2RS in fore wing: distinctly but not strongly angled.

#### Molecular data.

Sequences in BOLD: 2, barcode compliant sequences: 2.

#### Biology/ecology.

Malaise-trapped.

#### Distribution.

Costa Rica, ACG.

#### Etymology.

We dedicate this species to Isidro Chacón in recognition of his diligent efforts for the ACG Programa de Parataxónomos, and Lepidoptera curatorial taxonomy for INBio, Costa Rica’s Instituto Nacional de Biodiversidad, and for ACG.

### Apanteles isidrovillegasi

Fernández-Triana sp. n.

http://zoobank.org/F34AD351-6E76-4DF8-AC90-B35873437850

http://species-id.net/wiki/Apanteles_isidrovillegasi

Figs 129 , 290

#### Type locality.

COSTA RICA, Alajuela, ACG, Sector Rincon Rain Forest, San Lucas, 320m, 10.91847, -85.30338.

#### Holotype.

♀ in CNC. Specimen labels: 1. DHJPAR0042063. 2. Voucher: D.H.Janzen & W.Hallwachs, DB: http://janzen.sas.upenn.edu, Area de Conservación Guanacaste, COSTA RICA, 11-SRNP-212.

#### Paratypes.

1 ♀ (CNC). COSTA RICA: Guanacaste, ACG database code: DHJPAR0039021.

#### Description.

**Female.** Body color: body mostly dark except for some sternites which may be pale. Antenna color: scape, pedicel, and flagellum dark. Coxae color (pro-, meso-, metacoxa): dark, dark, dark. Femora color (pro-, meso-, metafemur): pale, anteriorly dark/posteriorly pale, dark. Tibiae color (pro-, meso-, metatibia): pale, pale, anteriorly pale/posteriorly dark. Tegula and humeral complex color: tegula dark, humeral complex half pale/half dark. Pterostigma color: dark. Fore wing veins color: partially pigmented (a few veins may be dark but most are pale). Antenna length/body length: antenna about as long as body (head to apex of metasoma); if slightly shorter, at least extending beyond anterior 0.7 metasoma length. Body in lateral view: not distinctly flattened dorso–ventrally. Body length (head to apex of metasoma): 3.1–3.2 mm. Fore wing length: 3.1–3.2 mm. Ocular–ocellar line/posterior ocellus diameter: 2.3–2.5. Interocellar distance/posterior ocellus diameter: 1.7–1.9. Antennal flagellomerus 2 length/width: 2.9–3.1. Antennal flagellomerus 14 length/width: 1.4–1.6. Length of flagellomerus 2/length of flagellomerus 14: 2.3–2.5. Tarsal claws: simple. Metafemur length/width: 3.0–3.1. Metatibia inner spur length/metabasitarsus length: 0.4–0.5. Anteromesoscutum: mostly with deep, dense punctures (separated by less than 2.0 × its maximum diameter). Mesoscutellar disc: mostly smooth. Number of pits in scutoscutellar sulcus: 7 or 8. Maximum height of mesoscutellum lunules/maximum height of lateral face of mesoscutellum: 0.4–0.5. Propodeum areola: completely defined by carinae, including transverse carina extending to spiracle. Propodeum background sculpture: mostly sculptured. Mediotergite 1 length/width at posterior margin: 2.3–2.5. Mediotergite 1 shape: slightly widening from anterior margin to 0.7–0.8 mediotergite length (where maximum width is reached), then narrowing towards posterior margin. Mediotergite 1 sculpture: mostly sculptured, excavated area centrally with transverse striation inside and/or a polished knob centrally on posterior margin of mediotergite. Mediotergite 2 width at posterior margin/length: 2.8–3.1. Mediotergite 2 sculpture: mostly smooth. Outer margin of hypopygium: with a wide, medially folded, transparent, semi–desclerotized area; usually with 4 or more pleats. Ovipositor thickness: about same width throughout its length. Ovipositor sheaths length/metatibial length: 1.4–1.5. Length of fore wing veins r/2RS: 1.7–1.9. Length of fore wing veins 2RS/2M: 1.7–1.8. Length of fore wing veins 2M/(RS+M)b: 0.5–0.6. Pterostigma length/width: 3.1–3.5. Point of insertion of vein r in pterostigma: about half way point length of pterostigma. Angle of vein r with fore wing anterior margin: more or less perpendicular to fore wing margin. Shape of junction of veins r and 2RS in fore wing: distinctly but not strongly angled.

**Male.** Unknown.

#### Molecular data.

Sequences in BOLD: 2, barcode compliant sequences: 2.

#### Biology/ecology.

Solitary (Fig. 290). Host: Elachistidae, species with interim names elachJanzen01 Janzen244, elachJanzen01 Janzen250.

#### Distribution.

Costa Rica, ACG.

#### Comments.

This species is characterized by tegula and humeral complex of different color, pterostigma brown, ovipositor sheaths 1.4-1.5 × as long as metatibia length, and body length and fore wing length at least 3.2 mm. The relatively long ovipositor sheaths and molecular data support this species as unique among the Mesoamerican fauna so far known.

#### Etymology.

We dedicate this species to Isidro Villegas in recognition of his diligent efforts for the ACG Programa de Sectores.

### Apanteles ivonnetranae

Fernández-Triana sp. n.

http://zoobank.org/A28DD4AF-9E38-4047-AE34-0B7362126F8D

http://species-id.net/wiki/Apanteles_ivonnetranae

Fig. 33

#### Type locality.

COSTA RICA, Alajuela, ACG, Sector Rincon Rain Forest, Quebrada Escondida, 420m, 10.89928, -85.27486.

#### Holotype.

♀ in CNC. Specimen labels: 1. DHJPAR0045176. 2. COSTA RICA, Guanacaste, ACG, Sector Rincon Rain Forest, Quebrada Escondida, 17.vi.2011, 10.89928 N, -85.27486 W, 420m, DHJPAR0045176. 3. Voucher: D.H.Janzen & W.Hallwachs, DB: http://janzen.sas.upenn.edu, Area de Conservación Guanacaste, COSTA RICA, 11-SRNP-42893.

#### Paratypes.

16 ♀, 1 ♂ (BMNH, CNC, INBIO, INHS, NMNH). COSTA RICA: Guanacaste, ACG database codes 11-SRNP-42893.

#### Description.

**Female.** Body color: head dark, mesosoma dark with parts of axillar complex pale, metasoma with some mediotergites, most laterotergites, sternites, and/or hypopygium pale. Antenna color: scape and/or pedicel pale, flagellum dark. Coxae color (pro-, meso-, metacoxa): pale, pale, pale. Femora color (pro-, meso-, metafemur): pale, pale, mostly pale but with dark area dorsally. Tibiae color (pro-, meso-, metatibia): pale, pale, mostly pale but with posterior 0.2 or less dark. Tegula and humeral complex color: both pale. Pterostigma color: mostly dark, with small pale area centrally. Fore wing veins color: mostly dark (a few veins may be unpigmented). Antenna length/body length: antenna about as long as body (head to apex of metasoma); if slightly shorter, at least extending beyond anterior 0.7 metasoma length. Body in lateral view: not distinctly flattened dorso–ventrally. Body length (head to apex of metasoma): 2.7–2.8 mm. Fore wing length: 2.9–3.0 mm. Ocular–ocellar line/posterior ocellus diameter: 2.0–2.2. Interocellar distance/posterior ocellus diameter: 2.3–2.5. Antennal flagellomerus 2 length/width: 2.6–2.8. Antennal flagellomerus 14 length/width: 1.4–1.6. Length of flagellomerus 2/length of flagellomerus 14: 2.0–2.2. Tarsal claws: with single basal spine–like seta. Metafemur length/width: 3.0–3.1. Metatibia inner spur length/metabasitarsus length: 0.4–0.5. Anteromesoscutum: mostly with deep, dense punctures (separated by less than 2.0 × its maximum diameter). Mesoscutellar disc: mostly smooth. Number of pits in scutoscutellar sulcus: 9 or 10. Maximum height of mesoscutellum lunules/maximum height of lateral face of mesoscutellum: 0.2–0.3. Propodeum areola: completely defined by carinae, including transverse carina extending to spiracle. Propodeum background sculpture: mostly sculptured. Mediotergite 1 length/width at posterior margin: 2.3–2.5. Mediotergite 1 shape: clearly narrowing towards posterior margin. Mediotergite 1 sculpture: mostly sculptured, excavated area centrally with transverse striation inside and/or a polished knob centrally on posterior margin of mediotergite. Mediotergite 2 width at posterior margin/length: 4.0–4.3. Mediotergite 2 sculpture: mostly smooth. Outer margin of hypopygium: with a wide, medially folded, transparent, semi–desclerotized area; usually with 4 or more pleats. Ovipositor thickness: about same width throughout its length. Ovipositor sheaths length/metatibial length: 0.8–0.9. Length of fore wing veins r/2RS: 1.1–1.3. Length of fore wing veins 2RS/2M: 1.9–2.0. Length of fore wing veins 2M/(RS+M)b: 0.7–0.8. Pterostigma length/width: 3.6 or more. Point of insertion of vein r in pterostigma: about half way point length of pterostigma. Angle of vein r with fore wing anterior margin: clearly outwards, inclined towards fore wing apex. Shape of junction of veins r and 2RS in fore wing: strongly angulated, sometimes with a knob.

**Male.** The only specimen available for study has metacoxa almost complete brown (fully white-yellow in females), and metafemur and metatibia are darker in coloration.

#### Molecular data.

Sequences in BOLD: 1, barcode compliant sequences: 1.

#### Biology/ecology.

Gregarious. Host: Tortricidae, *Anacrusis nephrodes*.

#### Distribution.

Costa Rica, ACG.

#### Etymology.

We dedicate this species to Ivonne Traña in recognition of her diligent efforts for the ACG Comedor in Puesto Pocosol.

### Apanteles jairomoyai

Fernández-Triana sp. n.

http://zoobank.org/A38C7C46-A8E2-491F-A91F-37045E790494

http://species-id.net/wiki/Apanteles_jairomoyai

Fig. 76

#### Type locality.

COSTA RICA, Alajuela, ACG, Sector San Cristobal, Potrero Argentina, 520m, 10.89021, -85.38803.

#### Holotype.

♀ in CNC. Specimen labels: 1. DHJPAR0027548. 2. San Gerardo, Sitio Argentina, 12-18 Jan. 2008.

#### Description.

**Female.** Body color: head and mesosoma mostly dark, metasoma with some tergites and/or most of sternites pale. Antenna color: scape, pedicel, and flagellum dark. Coxae color (pro-, meso-, metacoxa): pale, pale, pale. Femora color (pro-, meso-, metafemur): pale, pale, pale. Tibiae color (pro-, meso-, metatibia): pale, pale, pale. Tegula and humeral complex color: both pale. Pterostigma color: dark. Fore wing veins color: mostly dark (a few veins may be unpigmented). Antenna length/body length: antenna about as long as body (head to apex of metasoma); if slightly shorter, at least extending beyond anterior 0.7 metasoma length. Body in lateral view: not distinctly flattened dorso–ventrally. Body length (head to apex of metasoma): 2.3–2.4 mm. Fore wing length: 2.7–2.8 mm. Ocular–ocellar line/posterior ocellus diameter: 1.7–1.9. Interocellar distance/posterior ocellus diameter: 1.4–1.6. Antennal flagellomerus 2 length/width: 2.9–3.1. Antennal flagellomerus 14 length/width: 2.0–2.2. Length of flagellomerus 2/length of flagellomerus 14: 2.0–2.2. Tarsal claws: with single basal spine–like seta (?). Metafemur length/width: 3.0–3.1. Anteromesoscutum: mostly with deep, dense punctures (separated by less than 2.0 × its maximum diameter). Mesoscutellar disc: mostly smooth. Number of pits in scutoscutellar sulcus: 5 or 6. Maximum height of mesoscutellum lunules/maximum height of lateral face of mesoscutellum: 0.4–0.5. Propodeum areola: completely defined by carinae, including transverse carina extending to spiracle. Propodeum background sculpture: mostly sculptured. Mediotergite 1 length/width at posterior margin: 2.9–3.1. Mediotergite 1 shape: mostly parallel–sided for 0.5–0.7 of its length, then narrowing posteriorly so mediotergite anterior width >1.1 × posterior width. Mediotergite 1 sculpture: mostly sculptured, excavated area centrally with transverse striation inside and/or a polished knob centrally on posterior margin of mediotergite. Mediotergite 2 width at posterior margin/length: 3.6–3.9. Mediotergite 2 sculpture: mostly smooth. Outer margin of hypopygium: with a wide, medially folded, transparent, semi–desclerotized area; usually with 4 or more pleats. Ovipositor thickness: about same width throughout its length. Ovipositor sheaths length/metatibial length: 0.8–0.9. Length of fore wing veins r/2RS: 1.1–1.3. Length of fore wing veins 2RS/2M: 1.4–1.6. Length of fore wing veins 2M/(RS+M)b: 0.9–1.0. Pterostigma length/width: 3.1–3.5. Point of insertion of vein r in pterostigma: clearly beyond half way point length of pterostigma. Angle of vein r with fore wing anterior margin: clearly outwards, inclined towards fore wing apex. Shape of junction of veins r and 2RS in fore wing: strongly angulated, sometimes with a knob.

**Male.** Unknown.

#### Molecular data.

Sequences in BOLD: 1, barcode compliant sequences: 1.

#### Biology/ecology.

Malaise-trapped.

#### Distribution.

Costa Rica, ACG.

#### Etymology.

We dedicate this species to Jairo Moya in recognition of his diligent efforts for the ACG Programa de Sectores.

### Apanteles javiercontrerasi

Fernández-Triana sp. n.

http://zoobank.org/7994CB5E-9CF8-4C49-B270-62CDDD4220CC

http://species-id.net/wiki/Apanteles_javiercontrerasi

Figs 93 , 272

Apanteles Rodriguez123. Smith et al. (2008). Interim name provided by the authors.

#### Type locality.

COSTA RICA, Guanacaste, ACG, Sector Del Oro, Bosque Aguirre, 620m, 11.00060, -85.43800.

#### Holotype.

♀ in CNC. Specimen labels: 1. DHJPAR0002284, 04-SRNP-26851. 2. COSTA RICA, Guanacaste, ACG, Sector Del Oro, Bosque Aguirre, 02.xii.2004, 620m, 11.00060, -85.43800, 04-SRNP-26851.

#### Paratypes.

32 ♀, 21 ♂ (BMNH, CNC, INBIO, INHS, NMNH). COSTA RICA, ACG database codes: DHJPAR0004073, DHJPAR0002234, DHJPAR0002287, DHJPAR0031088, DHJPAR0035444, 09-SRNP-69957.

#### Description.

**Female.** Body color: head dark, mesosoma dark with parts of axillar complex pale, metasoma with some mediotergites, most laterotergites, sternites, and/or hypopygium pale. Antenna color: scape and/or pedicel pale, flagellum dark. Coxae color (pro-, meso-, metacoxa): pale, pale, pale. Femora color (pro-, meso-, metafemur): pale, pale, pale. Tibiae color (pro-, meso-, metatibia): pale, pale, anteriorly pale/posteriorly dark. Tegula and humeral complex color: both pale. Pterostigma color: dark with pale spot at base. Fore wing veins color: mostly dark (a few veins may be unpigmented). Antenna length/body length: antenna about as long as body (head to apex of metasoma); if slightly shorter, at least extending beyond anterior 0.7 metasoma length. Body in lateral view: not distinctly flattened dorso–ventrally. Body length (head to apex of metasoma): 2.9–3.0 mm, rarely 2.3–2.4 mm, 2.5–2.6 mm or 2.7–2.8 mm. Fore wing length: 2.7–2.8 mm, 2.9–3.0 mm, rarely 2.5–2.6 mm. Ocular–ocellar line/posterior ocellus diameter: 2.0–2.2. Interocellar distance/posterior ocellus diameter: 2.0–2.2. Antennal flagellomerus 2 length/width: 2.3–2.5. Antennal flagellomerus 14 length/width: 1.1–1.3. Length of flagellomerus 2/length of flagellomerus 14: 2.3–2.5. Tarsal claws: with single basal spine–like seta. Metafemur length/width: 2.8–2.9. Metatibia inner spur length/metabasitarsus length: 0.4–0.5. Anteromesoscutum: mostly smooth or with shallow sparse punctures, except for lateral and/or posterior margins where it has deeper and/or denser punctures. Mesoscutellar disc: mostly smooth. Number of pits in scutoscutellar sulcus: 9 or 10. Maximum height of mesoscutellum lunules/maximum height of lateral face of mesoscutellum: 0.4–0.5. Propodeum areola: completely defined by carinae, including transverse carina extending to spiracle. Propodeum background sculpture: mostly sculptured. Mediotergite 1 length/width at posterior margin: 2.9–3.1. Mediotergite 1 shape: mostly parallel–sided for 0.5–0.7 of its length, then narrowing posteriorly so mediotergite anterior width >1.1 × posterior width. Mediotergite 1 sculpture: with some sculpture near lateral margins and/or posterior 0.2–0.4 of mediotergite. Mediotergite 2 width at posterior margin/length: 3.2–3.5. Mediotergite 2 sculpture: mostly smooth. Outer margin of hypopygium: with a wide, medially folded, transparent, semi–desclerotized area; usually with 4 or more pleats. Ovipositor thickness: anterior width at most 2.0 × posterior width (beyond ovipositor constriction). Ovipositor sheaths length/metatibial length: 0.4–0.5, rarely 0.6–0.7. Length of fore wing veins r/2RS: 1.1–1.3. Length of fore wing veins 2RS/2M: 1.4–1.6. Length of fore wing veins 2M/(RS+M)b: 0.7–0.8. Pterostigma length/width: 2.6–3.0. Point of insertion of vein r in pterostigma: about half way point length of pterostigma. Angle of vein r with fore wing anterior margin: clearly outwards, inclined towards fore wing apex. Shape of junction of veins r and 2RS in fore wing: distinctly but not strongly angled.

**Male.** As in female.

#### Molecular data.

Sequences in BOLD: 33, barcode compliant sequences: 31.

#### Biology/ecology.

Gregarious (Fig. 272). Hosts: Crambidae, *Ategumia lotanalis*, *Ategumia matutinalis* and sibling cryptic species hiding inside these names.

#### Distribution.

Costa Rica, ACG.

#### Etymology.

We dedicate this species to Javier Contreras in recognition of his diligent efforts for the ACG Programa de Mantenimiento mechanico.

### Apanteles javierobandoi

Fernández-Triana sp. n.

http://zoobank.org/ED3598BD-ED38-4280-8A79-9E878394BDF8

http://species-id.net/wiki/Apanteles_javierobandoi

Fig. 130

Apanteles Rodriguez101. Smith et al. (2008). Interim name provided by the authors.

#### Type locality.

COSTA RICA, Guanacaste, ACG, Sector Pitilla, Colocho, 375m, 11.02367, -85.41884.

#### Holotype.

♀ in CNC. Specimen labels: 1. DHJPAR0039054. 2. Voucher: D.H.Janzen & W.Hallwachs, DB: http://janzen.sas.upenn.edu, Area de Conservación Guanacaste, COSTA RICA, 10-SRNP-30592.

#### Paratypes.

2 ♀, 1 ♂ (CNC). COSTA RICA, ACG database codes: DHJPAR0039057, DHJPAR0039066, DHJPAR0039095.

#### Description.

**Female.** Body color: body mostly dark except for some sternites which may be pale. Antenna color: scape, pedicel, and flagellum dark. Coxae color (pro-, meso-, metacoxa): dark, dark, dark. Femora color (pro-, meso-, metafemur): anteriorly dark/posteriorly pale, dark, dark. Tibiae color (pro-, meso-, metatibia): pale, anteriorly pale/posteriorly dark, anteriorly pale/posteriorly dark. Tegula and humeral complex color: both dark. Pterostigma color: dark. Fore wing veins color: mostly dark (a few veins may be unpigmented). Antenna length/body length: antenna about as long as body (head to apex of metasoma); if slightly shorter, at least extending beyond anterior 0.7 metasoma length. Body in lateral view: not distinctly flattened dorso–ventrally. Body length (head to apex of metasoma): 2.5–2.6 mm or 3.1–3.2 mm. Fore wing length: 2.5–2.6 mm or 2.9–3.0 mm. Ocular–ocellar line/posterior ocellus diameter: 2.3–2.5. Interocellar distance/posterior ocellus diameter: 2.0–2.2. Antennal flagellomerus 2 length/width: 2.6–2.8. Antennal flagellomerus 14 length/width: 1.4–1.6. Length of flagellomerus 2/length of flagellomerus 14: 1.7–1.9. Tarsal claws: simple. Metafemur length/width: 3.0–3.1. Metatibia inner spur length/metabasitarsus length: 0.4–0.5. Anteromesoscutum: mostly with deep, dense punctures (separated by less than 2.0 × its maximum diameter). Mesoscutellar disc: with punctures near margins, central part mostly smooth. Number of pits in scutoscutellar sulcus: 7 or 8. Maximum height of mesoscutellum lunules/maximum height of lateral face of mesoscutellum: 0.4–0.5. Propodeum areola: completely defined by carinae, including transverse carina extending to spiracle. Propodeum background sculpture: mostly sculptured. Mediotergite 1 length/width at posterior margin: 1.7–1.9 or 2.0–2.2. Mediotergite 1 shape: more or less parallel–sided. Mediotergite 1 sculpture: mostly sculptured, excavated area centrally with transverse striation inside and/or a polished knob centrally on posterior margin of mediotergite. Mediotergite 2 width at posterior margin/length: 3.6–3.9. Mediotergite 2 sculpture: mostly smooth. Outer margin of hypopygium: with a wide, medially folded, transparent, semi–desclerotized area; usually with 4 or more pleats. Ovipositor thickness: about same width throughout its length. Ovipositor sheaths length/metatibial length: 0.8–0.9. Length of fore wing veins r/2RS: 1.7–1.9. Length of fore wing veins 2RS/2M: 1.1–1.3. Length of fore wing veins 2M/(RS+M)b: 0.5–0.6. Pterostigma length/width: 2.6–3.0. Point of insertion of vein r in pterostigma: about half way point length of pterostigma. Angle of vein r with fore wing anterior margin: clearly outwards, inclined towards fore wing apex. Shape of junction of veins r and 2RS in fore wing: distinctly but not strongly angled.

**Male.** As in female.

#### Molecular data.

Sequences in BOLD: 4, barcode compliant sequences: 4.

#### Biology/ecology.

Solitary. Host: Choreutidae, *Rhobonda gaurisana*.

#### Distribution.

Costa Rica, ACG.

#### Etymology.

We dedicate this species to Javier Obando in recognition of his diligent efforts for the ACG Programa de Sectores.

### Apanteles javiersihezari

Fernández-Triana sp. n.

http://zoobank.org/9A22A6F4-635C-4AA8-9161-86CBCEDDC732

http://species-id.net/wiki/Apanteles_javiersihezari

Figs 80 , 262

#### Type locality.

COSTA RICA, Guanacaste, ACG, Sector Pitilla, Estación Pitilla, 675m, 10.98931, -85.42581.

#### Holotype.

♀ in CNC. Specimen labels: 1. DHJPAR0038337. 2. Voucher: D.H.Janzen & W.Hallwachs, DB: http://janzen.sas.upenn.edu, Area de Conservación Guanacaste, COSTA RICA, 10-SRNP-30311.

#### Paratypes.

2 ♀ (CNC, NMNH). COSTA RICA, ACG database codes: DHJPAR0038189, DHJPAR0038333.

#### Description.

**Female.** Body color: head and mesosoma mostly dark, metasoma with some tergites and/or most of sternites pale. Antenna color: scape, pedicel, and flagellum dark. Coxae color (pro-, meso-, metacoxa): pale, pale, pale. Femora color (pro-, meso-, metafemur): pale, pale, mostly pale but posterior 0.2 or less dark. Tibiae color (pro-, meso-, metatibia): pale, pale, anteriorly pale/posteriorly dark. Tegula and humeral complex color: both pale. Pterostigma color: dark with pale spot at base. Fore wing veins color: mostly dark (a few veins may be unpigmented). Antenna length/body length: antenna about as long as body (head to apex of metasoma); if slightly shorter, at least extending beyond anterior 0.7 metasoma length. Body in lateral view: not distinctly flattened dorso–ventrally. Body length (head to apex of metasoma): 3.1–3.2 mm. Fore wing length: 2.9–3.0 mm. Ocular–ocellar line/posterior ocellus diameter: 2.6 or more. Interocellar distance/posterior ocellus diameter: 2.0–2.2. Antennal flagellomerus 2 length/width: 2.9–3.1. Antennal flagellomerus 14 length/width: 1.4–1.6. Length of flagellomerus 2/length of flagellomerus 14: 2.0–2.2. Tarsal claws: simple. Metafemur length/width: 3.4–3.5. Metatibia inner spur length/metabasitarsus length: 0.4–0.5. Anteromesoscutum: mostly with shallow, dense punctures (separated by less than 2.0 × its maximum diameter). Mesoscutellar disc: mostly punctured. Number of pits in scutoscutellar sulcus: 5 or 6. Maximum height of mesoscutellum lunules/maximum height of lateral face of mesoscutellum: 0.4–0.5. Propodeum areola: completely defined by carinae, including transverse carina extending to spiracle. Propodeum background sculpture: mostly sculptured. Mediotergite 1 length/width at posterior margin: 3.2–3.4. Mediotergite 1 shape: mostly parallel–sided for 0.5–0.7 of its length, then narrowing posteriorly so mediotergite anterior width >1.1 × posterior width. Mediotergite 1 sculpture: mostly sculptured, excavated area centrally with transverse striation inside and/or a polished knob centrally on posterior margin of mediotergite. Mediotergite 2 width at posterior margin/length: 3.2–3.5. Mediotergite 2 sculpture: mostly smooth. Outer margin of hypopygium: with a wide, medially folded, transparent, semi–desclerotized area; usually with 4 or more pleats. Ovipositor thickness: about same width throughout its length. Ovipositor sheaths length/metatibial length: 1.0–1.1. Length of fore wing veins r/2RS: 1.1–1.3. Length of fore wing veins 2RS/2M: 1.1–1.3. Length of fore wing veins 2M/(RS+M)b: 0.9–1.0. Pterostigma length/width: 2.6–3.0. Point of insertion of vein r in pterostigma: about half way point length of pterostigma. Angle of vein r with fore wing anterior margin: clearly outwards, inclined towards fore wing apex. Shape of junction of veins r and 2RS in fore wing: strongly angulated, sometimes with a knob.

**Male.** Unknown.

#### Molecular data.

No molecular data available for this species.

#### Biology/ecology.

Solitary (Fig. 262). Hosts: Crambidae, *Diacme* BioLep02, *Neurophyseta* BioLep237.

#### Distribution.

Costa Rica, ACG.

#### Comments.

The barcodes were not included in the molecular phylogenetic analysis done for this paper.

#### Etymology.

We dedicate this species to Javier Sihezar in recognition of his diligent efforts for the ACG Programa de Sectores.

### Apanteles jesusbrenesi

Fernández-Triana sp. n.

http://zoobank.org/8A6965F3-887C-4203-83C9-786C169F1CBD

http://species-id.net/wiki/Apanteles_jesusbrenesi

Figs 94 , 273

Apanteles Rodriguez91. Smith et al. (2008). Interim name provided by the authors.

#### Type locality.

COSTA RICA, Alajuela, ACG, Sector Pitilla, Manguera, 470m, 10.99590, -85.39842.

#### Holotype.

♀ in CNC. Specimen labels: 1. DHJPAR0039115. 2. COSTA RICA, Guanacaste, ACG, Sector Pitilla, Manguera, 4.iii.2010, 10.99590°N, 85.398423°W, 470m, DHJPAR0039115. 3. Voucher: D.H.Janzen & W.Hallwachs, DB: http://janzen.sas.upenn.edu, Area de Conservación Guanacaste, COSTA RICA, 10-SRNP-71103.

#### Paratypes.

3 ♂ (CNC). COSTA RICA, ACG database codes: DHJPAR0039015, DHJPAR0038218, DHJPAR0039011.

#### Description.

**Female.** Body color: head dark, mesosoma dark with parts of axillar complex pale, metasoma with some mediotergites, most laterotergites, sternites, and/or hypopygium pale. Antenna color: scape and/or pedicel pale, flagellum dark. Coxae color (pro-, meso-, metacoxa): pale, pale, pale. Femora color (pro-, meso-, metafemur): pale, pale, pale. Tibiae color (pro-, meso-, metatibia): pale, pale, mostly pale but with posterior 0.2 or less dark. Tegula and humeral complex color: both pale. Pterostigma color: dark with pale spot at base. Fore wing veins color: mostly dark (a few veins may be unpigmented). Antenna length/body length: antenna about as long as body (head to apex of metasoma); if slightly shorter, at least extending beyond anterior 0.7 metasoma length. Body in lateral view: not distinctly flattened dorso–ventrally. Body length (head to apex of metasoma): 3.1–3.2 mm. Fore wing length: 3.1–3.2 mm. Ocular–ocellar line/posterior ocellus diameter: 1.4–1.6. Interocellar distance/posterior ocellus diameter: 1.7–1.9. Antennal flagellomerus 2 length/width: 2.9–3.1. Antennal flagellomerus 14 length/width: 1.4–1.6. Length of flagellomerus 2/length of flagellomerus 14: 2.0–2.2. Tarsal claws: with single basal spine–like seta. Metafemur length/width: 2.8–2.9. Metatibia inner spur length/metabasitarsus length: 0.4–0.5. Anteromesoscutum: mostly with deep, dense punctures (separated by less than 2.0 × its maximum diameter). Mesoscutellar disc: with a few sparse punctures. Number of pits in scutoscutellar sulcus: 7 or 8. Maximum height of mesoscutellum lunules/maximum height of lateral face of mesoscutellum: 0.4–0.5. Propodeum areola: completely defined by carinae, including transverse carina extending to spiracle. Propodeum background sculpture: mostly sculptured. Mediotergite 1 length/width at posterior margin: 2.3–2.5. Mediotergite 1 shape: mostly parallel–sided for 0.5–0.7 of its length, then narrowing posteriorly so mediotergite anterior width >1.1 × posterior width. Mediotergite 1 sculpture: with some sculpture near lateral margins and/or posterior 0.2–0.4 of mediotergite. Mediotergite 2 width at posterior margin/length: 2.8–3.1. Mediotergite 2 sculpture: mostly smooth. Outer margin of hypopygium: with a wide, medially folded, transparent, semi–desclerotized area; usually with 4 or more pleats. Ovipositor thickness: about same width throughout its length. Ovipositor sheaths length/metatibial length: 0.6–0.7. Length of fore wing veins r/2RS: 1.4–1.6. Length of fore wing veins 2RS/2M: 1.4–1.6. Length of fore wing veins 2M/(RS+M)b: 0.7–0.8. Pterostigma length/width: 2.6–3.0. Point of insertion of vein r in pterostigma: about half way point length of pterostigma. Angle of vein r with fore wing anterior margin: clearly outwards, inclined towards fore wing apex. Shape of junction of veins r and 2RS in fore wing: distinctly but not strongly angled.

**Male.** As in female, but tergites slightly darker, including T3 completely dark brown (mostly yellow in female).

#### Molecular data.

Sequences in BOLD: 24, barcode compliant sequences: 23.

#### Biology/ecology.

Solitary (Fig. 273). Host: four species of Gelechiidae feeding on woody Violaceae.

#### Distribution.

Costa Rica, ACG.

#### Etymology.

We dedicate this species to Jesús Brenes in recognition of his diligent efforts for the ACG Programa Forestal.

### Apanteles jesusugaldei

Fernández-Triana sp. n.

http://zoobank.org/C0BB9361-FD92-4FA0-BA18-F1CE98D6279C

http://species-id.net/wiki/Apanteles_jesusugaldei

Figs 183 , 314

Apanteles Rodriguez28 (Smith et al. 2006). Interim name provided by the authors.

#### Type locality.

COSTA RICA, Alajuela, ACG, Sector Rincon Rain Forest, Sendero Juntas, 400m, 10.90661, -85.28784.

#### Holotype.

♀ in CNC. Specimen labels: 1. DHJPAR0001591. 2. COSTA RICA, Alajuela, ACG, Sector Rincon Rain Forest, Sendero Juntas, 21.i.2004, 400m, 10.90661, -85.28784, 04-SRNP-40254.

#### Paratypes.

16 ♀, 9 ♂ (BMNH, CNC, INBIO, INHS, NMNH). COSTA RICA: ACG database codes: DHJPAR0001583, DHJPAR0001588, DHJPAR0001590, DHJPAR0001591, DHJPAR0002700, DHJPAR0002711, DHJPAR0002715.

#### Description.

**Female.** Metatibia color (outer face): entirely or mostly (>0.7 metatibia length) dark brown to black, with yellow to white coloration usually restricted to anterior 0.2 or less, rarely with extended pale coloration (light yellow to orange–yellow), ranging from 0.4 to almost entire metatibia length. Fore wing veins color: veins C+Sc+R and R1 with brown coloration restricted narrowly to borders, interior area of those veins and pterostigma (and sometimes veins r, 2RS and 2M) transparent or white; other veins mostly transparent. Antenna length/body length: antenna about as long as body (head to apex of metasoma); if slightly shorter, at least extending beyond anterior 0.7 metasoma length. Body length (head to apex of metasoma): 2.3–2.4 mm. Fore wing length: 2.5–2.6 mm, rarely 2.7–2.8 mm. Metafemur length/width: 2.8–2.9, 3.0–3.1 or 3.2–3.3. Mediotergite 1 length/width at posterior margin: 2.9 or more. Mediotergite 1 maximum width/width at posterior margin: 1.8–1.9. Ovipositor sheaths length/metafemur length: 1.0 or 1.1. Ovipositor sheaths length/metatibia length: 0.9.

#### Molecular data.

Sequences in BOLD: 16, barcode compliant sequences: 15.

#### Biology/ecology.

Gregarious (Fig. 314). Host: Hesperiidae, *Aguna* Burns02.

#### Distribution.

Costa Rica, ACG.

#### Etymology.

We dedicate this species to Jesús Ugalde in recognition of his diligent efforts for the administration of INBio, Costa Rica’s Instituto Nacional de Biodiversidad.

### Apanteles jimmychevezi

Fernández-Triana sp. n.

http://zoobank.org/987EE0A1-15B4-43AD-8C10-4708F1917C9A

http://species-id.net/wiki/Apanteles_jimmychevezi

Fig. 160

#### Type locality.

COSTA RICA, Alajuela, ACG, Sector San Cristobal, Rio Blanco Abajo, 500m, 10.90037, -85.37254.

#### Holotype.

♀ in CNC. Specimen labels: 1. DHJPAR0027417. 2. San Gerardo: Rio Abajo Blanco, Date: 12-18 May 2008. 3. COSTA RICA: Alajuela, ACG, Sector San Cristobal, Rio Blanco Abajo, 18.v.2008, 10.90037, -85.37254, 500m, DHJPAR0027417.

#### Description.

**Female.** Body color: body mostly dark except for some sternites which may be pale. Antenna color: scape and/or pedicel pale, flagellum dark. Coxae color (pro-, meso-, metacoxa): pale, pale, dark. Femora color (pro-, meso-, metafemur): pale, pale, mostly dark but with pale spot antero–ventrally. Tibiae color (pro-, meso-, metatibia): pale, pale, anteriorly pale/posteriorly dark. Tegula and humeral complex color: tegula dark, humeral complex pale. Pterostigma color: dark with pale spot at base. Fore wing veins color: mostly dark (a few veins may be unpigmented). Antenna length/body length: antenna very short, barely or not extending beyond mesosoma length. Body in lateral view: not distinctly flattened dorso–ventrally. Body length (head to apex of metasoma): 2.0 mm or less. Fore wing length: 2.0 mm or less. Ocular–ocellar line/posterior ocellus diameter: 2.0–2.2. Interocellar distance/posterior ocellus diameter: 1.7–1.9. Antennal flagellomerus 2 length/width: 1.7–1.9. Antennal flagellomerus 14 length/width: 1.0 or less. Length of flagellomerus 2/length of flagellomerus 14: 1.4–1.6. Tarsal claws: simple. Metafemur length/width: 3.0–3.1. Metatibia inner spur length/metabasitarsus length: 0.4–0.5. Anteromesoscutum: mostly with deep, dense punctures (separated by less than 2.0 × its maximum diameter). Mesoscutellar disc: with punctures near margins, central part mostly smooth. Number of pits in scutoscutellar sulcus: 5 or 6. Maximum height of mesoscutellum lunules/maximum height of lateral face of mesoscutellum: 0.6–0.7. Propodeum areola: completely defined by carinae, but only partial or absent transverse carina. Propodeum background sculpture: partly sculptured, especially on anterior 0.5. Mediotergite 1 length/width at posterior margin: 3.2–3.4. Mediotergite 1 shape: mostly parallel–sided for 0.5–0.7 of its length, then narrowing posteriorly so mediotergite anterior width >1.1 × posterior width. Mediotergite 1 sculpture: mostly sculptured, excavated area centrally with transverse striation inside and/or a polished knob centrally on posterior margin of mediotergite. Mediotergite 2 width at posterior margin/length: 3.6–3.9. Mediotergite 2 sculpture: mostly smooth. Outer margin of hypopygium: with a wide, medially folded, transparent, semi–desclerotized area; usually with 4 or more pleats. Ovipositor thickness: anterior width at most 2.0 × posterior width (beyond ovipositor constriction). Ovipositor sheaths length/metatibial length: 0.4–0.5. Length of fore wing veins r/2RS: 2.3 or more. Length of fore wing veins 2RS/2M: 0.8 or less. Length of fore wing veins 2M/(RS+M)b: 0.9–1.0. Pterostigma length/width: 2.1–2.5. Point of insertion of vein r in pterostigma: about half way point length of pterostigma. Angle of vein r with fore wing anterior margin: clearly outwards, inclined towards fore wing apex. Shape of junction of veins r and 2RS in fore wing: evenly curved.

**Male.** Unknown.

#### Molecular data.

Sequences in BOLD: 1, barcode compliant sequences: 1.

#### Biology/ecology.

Malaise-trapped.

#### Distribution.

Costa Rica, ACG.

#### Etymology.

We dedicate this species to Jimmy Chavez in recognition of his diligent efforts as ACG administrative assistant.

### Apanteles johanvargasi

Fernández-Triana sp. n.

http://zoobank.org/EEED2D61-C276-4B5B-B93B-B2B1301DD5A0

http://species-id.net/wiki/Apanteles_johanvargasi

Fig. 184

#### Type locality.

COSTA RICA, Guanacaste, ACG, Sector Del Oro, Quebrada Serrano, 585m, 11.00025, -85.45614.

#### Holotype.

♀ in CNC. Specimen labels: 1. DHJPAR0013702.

#### Paratypes.

1 ♀, 1 ♂ (CNC). COSTA RICA: ACG database codes: DHJPAR0013702.

#### Description.

**Female.** Metatibia color (outer face): entirely or mostly (>0.7 metatibia length) dark brown to black, with yellow to white coloration usually restricted to anterior 0.2 or less. Fore wing veins color: veins C+Sc+R and R1 with brown coloration restricted narrowly to borders, interior area of those veins and pterostigma (and sometimes veins r, 2RS and 2M) transparent or white; other veins mostly transparent. Antenna length/body length: antenna about as long as body (head to apex of metasoma); if slightly shorter, at least extending beyond anterior 0.7 metasoma length. Body length (head to apex of metasoma): 2.1–2.2 mm. Fore wing length: 2.3–2.4 mm. Metafemur length/width: 3.2–3.3. Mediotergite 1 length/width at posterior margin: 2.5–2.6. Mediotergite 1 maximum width/width at posterior margin: 1.6–1.7. Ovipositor sheaths length/metafemur length: 1.0. Ovipositor sheaths length/metatibia length: 0.9.

#### Molecular data.

Sequences in BOLD: 1, barcode compliant sequences: 1.

#### Biology/ecology.

Gregarious. Host: Hesperiidae, *Telemiades fides*.

#### Distribution.

Costa Rica, ACG.

#### Etymology.

We dedicate this species to Johan Vargas in recognition of his diligent efforts for the ACG Programa de Parataxónomos at Estación Biologico Santa Rosa.

### Apanteles jorgecortesi

Fernández-Triana sp. n.

http://zoobank.org/D2BDE1A4-015C-479F-A66F-D94C9B8D43C4

http://species-id.net/wiki/Apanteles_jorgecortesi

Figs 10 , 213

Apanteles Rodriguez32. Smith et al. (2008). Interim name provided by the authors.

#### Type locality.

COSTA RICA, Alajuela, ACG, Sector San Cristobal, Rio Blanco Abajo, 500m, 10.90037, -85.37254.

#### Holotype.

♀ in CNC. Specimen labels: 1. Costa Rica: Alajuela, ACG, Sector San Cristobal, Rio Blanco Abajo, 08.06.2001, Carolina Cano. 2. 01-SRNP-2855, Stenoma Janzen07, Vismia baccifera.

#### Paratypes.

15 ♀, 11 ♂ (BMNH, CNC, INBIO, INHS, NMNH). COSTA RICA, ACG database codes: DHJPAR0003006, DHJPAR0035341, 00-SRNP-2013, 01-SRNP-2855, 03-SRNP-21344, 03-SRNP-21404, 04-SRNP-33985, 04-SRNP-45058.

#### Description.

**Female.** Body color: body mostly dark except for some sternites which may be pale. Antenna color: scape, pedicel, and flagellum dark. Coxae color (pro-, meso-, metacoxa): dark, dark, dark. Femora color (pro-, meso-, metafemur): anteriorly dark/posteriorly pale, dark, dark. Tibiae color (pro-, meso-, metatibia): pale, pale, anteriorly pale/posteriorly dark. Tegula and humeral complex color: tegula pale, humeral complex half pale/half dark. Pterostigma color: mostly pale and/or transparent, with thin dark borders. Fore wing veins color: partially pigmented (a few veins may be dark but most are pale). Antenna length/body length: antenna about as long as body (head to apex of metasoma); if slightly shorter, at least extending beyond anterior 0.7 metasoma length. Body in lateral view: not distinctly flattened dorso–ventrally. Body length (head to apex of metasoma): 2.9–3.0 mm, rarely 3.1–3.2 mm. Fore wing length: 3.1–3.2 mm or 3.3–3.4 mm. Ocular–ocellar line/posterior ocellus diameter: 2.3–2.5. Interocellar distance/posterior ocellus diameter: 2.0–2.2. Antennal flagellomerus 2 length/width: 2.9–3.1. Antennal flagellomerus 14 length/width: 1.7–1.9. Length of flagellomerus 2/length of flagellomerus 14: 2.0–2.2. Tarsal claws: with single basal spine–like seta. Metafemur length/width: 3.2–3.3. Metatibia inner spur length/metabasitarsus length: 0.4–0.5. Anteromesoscutum: mostly with deep, dense punctures (separated by less than 2.0 × its maximum diameter). Mesoscutellar disc: mostly punctured. Number of pits in scutoscutellar sulcus: 7 or 8. Maximum height of mesoscutellum lunules/maximum height of lateral face of mesoscutellum: 0.4–0.5. Propodeum areola: completely defined by carinae, including transverse carina extending to spiracle. Propodeum background sculpture: mostly sculptured. Mediotergite 1 length/width at posterior margin: 1.7–1.9. Mediotergite 1 shape: slightly widening from anterior margin to 0.7–0.8 mediotergite length (where maximum width is reached), then narrowing towards posterior margin. Mediotergite 1 sculpture: mostly sculptured, excavated area centrally with transverse striation inside and/or a polished knob centrally on posterior margin of mediotergite. Mediotergite 2 width at posterior margin/length: 2.8–3.1. Mediotergite 2 sculpture: mostly smooth. Outer margin of hypopygium: with a wide, medially folded, transparent, semi–desclerotized area; usually with 4 or more pleats. Ovipositor thickness: about same width throughout its length. Ovipositor sheaths length/metatibial length: 1.2–1.3 or 1.4–1.5. Length of fore wing veins r/2RS: 1.7–1.9. Length of fore wing veins 2RS/2M: 1.1–1.3. Length of fore wing veins 2M/(RS+M)b: 0.9–1.0. Pterostigma length/width: 3.1–3.5. Point of insertion of vein r in pterostigma: clearly beyond half way point length of pterostigma. Angle of vein r with fore wing anterior margin: clearly outwards, inclined towards fore wing apex. Shape of junction of veins r and 2RS in fore wing: distinctly but not strongly angled.

**Male.** As female but with darker legs.

#### Molecular data.

Sequences in BOLD: 67, barcode compliant sequences: 60.

#### Biology/ecology.

Gregarious (Fig. 213). Host: Elachistidae, *Stenoma* Janzen07.

#### Distribution.

Costa Rica, ACG.

#### Etymology.

We dedicate this species to Jorge Cortés in recognition of his diligent efforts for the ACG Programa de Seguridad.

### Apanteles jorgehernandezi

Fernández-Triana sp. n.

http://zoobank.org/1EC7291F-B239-4FA1-9DB4-75E1245DA446

http://species-id.net/wiki/Apanteles_jorgehernandezi

Figs 185 , 329

Apanteles Rodriguez163 (Smith et al. 2006). Interim name provided by the authors.

#### Type locality.

COSTA RICA, Alajuela, ACG, Sector Rincon Rain Forest, Sendero Albergue, 980m, 10.84886, -85.3281.

#### Holotype.

♀ in CNC. Specimen labels: 1. COSTA RICA, Alajuela, ACG, Sector Rincon Rain Forest, Sendero Albergue Crater, 22.iv.2006, 980m, 10.84886, -85.3281, DHJPAR0004996.

#### Paratypes.

34 ♀, 12 ♂ (BMNH, CNC, INBIO, INHS, NMNH). COSTA RICA, ACG database codes: DHJPAR0004996, DHJPAR0005221, DHJPAR0005235, DHJPAR0012785, DHJPAR0030903, DHJPAR0030927, DHJPAR0034260.

#### Description.

**Female.** Metatibia color (outer face): with extended pale coloration (light yellow to orange–yellow), ranging from 0.4 to almost entire metatibia length. Fore wing veins color: veins C+Sc+R and R1 with brown coloration restricted narrowly to borders, interior area of those veins and pterostigma (and sometimes veins r, 2RS and 2M) transparent or white; other veins mostly transparent. Antenna length/body length: antenna about as long as body (head to apex of metasoma); if slightly shorter, at least extending beyond anterior 0.7 metasoma length. Body length (head to apex of metasoma): 2.5–2.6 mm. Fore wing length: 2.7–2.8 mm. Metafemur length/width: 3.2–3.3. Mediotergite 1 length/width at posterior margin: 2.7–2.8. Mediotergite 1 maximum width/width at posterior margin: 1.6–1.7. Ovipositor sheaths length/metafemur length: 0.8. Ovipositor sheaths length/metatibia length: 0.7.

#### Molecular data.

Sequences in BOLD: 17, barcode compliant sequences: 11.

#### Biology/ecology.

Gregarious (Fig. 329). Hosts: Hesperiidae, *Astraptes alardus*, *Astraptes brevicauda*, *Astraptes*. *talus*, *Astraptes tucuti*, *Urbanus dorantes*.

#### Distribution.

Costa Rica, ACG.

#### Comments.

Some very minor differences in the barcoding region, but we are considering all those specimens as belonging to the same species except that the single dry forest *Urbanus dorantes* record is almost undoubtedly a specimen of *Apanteles duvalierbricenoi* placed incorrectly by a defective bardode.

#### Etymology.

We dedicate this species to Jorge Hernández in recognition of his diligent efforts for the ACG Programa de Parataxónomos and Estación Biológica Caribe, and the ACG plant inventory.

### Apanteles josecalvoi

Fernández-Triana sp. n.

http://zoobank.org/7FF8F57A-98E5-48C8-857C-1FA5CAE0B035

http://species-id.net/wiki/Apanteles_josecalvoi

Fig. 85

Apanteles Rodriguez44 (Smith et al. 2006). Interim name provided by the authors.

#### Type locality.

COSTA RICA, Guanacaste, ACG, Sector Pitilla, Medrano, 380m, 11.01602, -85.38053.

#### Holotype.

♀ in CNC. Specimen labels: 1. DHJPAR0038204. 2. Voucher: D.H.Janzen & W.Hallwachs, DB: http://janzen.sas.upenn.edu, Area de Conservación Guanacaste, COSTA RICA, 09-SRNP-73999.

#### Paratypes.

1 ♀ (CNC). COSTA RICA: Guanacaste, ACG database code: DHJPAR0045122.

#### Description.

**Female.** Body color: body mostly dark except for some sternites which may be pale. Antenna color: scape, pedicel, and flagellum dark. Coxae color (pro-, meso-, metacoxa): dark, dark, dark. Femora color (pro-, meso-, metafemur): anteriorly dark/posteriorly pale, dark, dark. Tibiae color (pro-, meso-, metatibia): pale, pale, anteriorly pale/posteriorly dark. Tegula and humeral complex color: tegula pale, humeral complex half pale/half dark. Pterostigma color: mostly pale and/or transparent, with thin dark borders. Fore wing veins color: mostly white or entirely transparent. Antenna length/body length: antenna about as long as body (head to apex of metasoma); if slightly shorter, at least extending beyond anterior 0.7 metasoma length. Body in lateral view: not distinctly flattened dorso–ventrally. Body length (head to apex of metasoma): 2.7–2.8 mm. Fore wing length: 2.9–3.0 mm. Ocular–ocellar line/posterior ocellus diameter: 2.6 or more. Interocellar distance/posterior ocellus diameter: 2.0–2.2. Antennal flagellomerus 2 length/width: 2.9–3.1. Antennal flagellomerus 14 length/width: 1.4–1.6. Length of flagellomerus 2/length of flagellomerus 14: 2.3–2.5. Tarsal claws: with single basal spine–like seta. Anteromesoscutum: mostly with deep, dense punctures (separated by less than 2.0 × its maximum diameter). Mesoscutellar disc: mostly smooth. Number of pits in scutoscutellar sulcus: 9 or 10. Maximum height of mesoscutellum lunules/maximum height of lateral face of mesoscutellum: 0.6–0.7. Propodeum areola: completely defined by carinae, including transverse carina extending to spiracle. Propodeum background sculpture: partly sculptured, especially on anterior 0.5. Mediotergite 1 length/width at posterior margin: 2.3–2.5 or 2.6–2.8. Mediotergite 1 shape: mostly parallel–sided for 0.5–0.7 of its length, then narrowing posteriorly so mediotergite anterior width >1.1 × posterior width. Mediotergite 1 sculpture: mostly sculptured, excavated area centrally with transverse striation inside and/or a polished knob centrally on posterior margin of mediotergite. Mediotergite 2 width at posterior margin/length: 2.4–2.7. Mediotergite 2 sculpture: mostly smooth. Outer margin of hypopygium: with a wide, medially folded, transparent, semi–desclerotized area; usually with 4 or more pleats. Ovipositor thickness: about same width throughout its length. Ovipositor sheaths length/metatibial length: 1.6–1.7. Length of fore wing veins r/2RS: 1.4–1.6. Length of fore wing veins 2RS/2M: 1.4–1.6. Length of fore wing veins 2M/(RS+M)b: 0.7–0.8. Pterostigma length/width: 3.1–3.5. Point of insertion of vein r in pterostigma: clearly beyond half way point length of pterostigma. Angle of vein r with fore wing anterior margin: clearly outwards, inclined towards fore wing apex. Shape of junction of veins r and 2RS in fore wing: distinctly but not strongly angled.

**Male.** Unknown.

#### Molecular data.

Sequences in BOLD: 8; barcode compliant sequences: 8.

#### Biology/ecology.

Solitary. Host: Elachistidae, *Antaeotricha zelleri*, *Gonioterma anna*.

#### Distribution.

Costa Rica, ACG.

#### Etymology.

We dedicate this species to José Calvo in recognition of his diligent efforts for the ACG Programa de Sectores.

### Apanteles josecortesi

Fernández-Triana sp. n.

http://zoobank.org/0A32E352-9F1E-427F-92A0-1CB4163C8E79

http://species-id.net/wiki/Apanteles_josecortesi

Figs 186 , 315

Apanteles Rodriguez17 (Smith et al. 2006). Interim name provided by the authors.

#### Type locality.

COSTA RICA, Alajuela, ACG, Sector San Cristobal, Sendero Vivero, 730m, 10.86739, -85.38744.

#### Holotype.

♀ in CNC. Specimen labels: 1. DHJPAR0002654. 2. COSTA RICA, Guanacaste, Area de Conservación Guanacaste, Sector San Cristobal, Sendero Vivero, 27 Sept. 1999. Carolina Cano. 3. 99-SRNP-13121, Nascus broteas, On Cupania glabra.

#### Paratypes.

66 ♀, 55 ♂ (BMNH, CNC, INBIO, INHS, NMNH). COSTA RICA, ACG database codes: See Appendix 2 for detailed label data.

#### Description.

**Female.** Metatibia color (outer face): entirely or mostly (>0.7 metatibia length) dark brown to black, with yellow to white coloration usually restricted to anterior 0.2 or less. Fore wing veins color: veins C+Sc+R and R1 with brown coloration restricted narrowly to borders, interior area of those veins and pterostigma (and sometimes veins r, 2RS and 2M) transparent or white; other veins mostly transparent. Antenna length/body length: antenna about as long as body (head to apex of metasoma); if slightly shorter, at least extending beyond anterior 0.7 metasoma length. Body length (head to apex of metasoma): 2.0 mm or less or 2.1–2.2 mm. Fore wing length: 2.1–2.2 mm or 2.3–2.4 mm. Metafemur length/width: 2.8–2.9 or 3.0–3.1. Mediotergite 1 length/width at posterior margin: 2.5–2.6. Mediotergite 1 maximum width/width at posterior margin: 1.6–1.7. Ovipositor sheaths length/metafemur length: 0.7 or 0.8. Ovipositor sheaths length/metatibia length: 0.5, 0.6 or 0.7.

#### Molecular data.

Sequences in BOLD: 55, barcode compliant sequences: 50.

#### Biology/ecology.

Gregarious (Fig. 315). Hosts: Hesperiidae, *Nascus broteas*, *Nascus solon*, *Nascus* sp.

#### Distribution.

Costa Rica, ACG.

#### Etymology.

We dedicate this species to José Cortes in recognition of his diligent efforts for the ACG Programa de Parataxónomos and Estación Biológica La Perla of Sector Mundo Nuevo of ACG.

### Apanteles josediazi

Fernández-Triana sp. n.

http://zoobank.org/F673AB9C-A2C9-43D5-A33A-251B59E9707E

http://species-id.net/wiki/Apanteles_josediazi

Fig. 132

#### Type locality.

COSTA RICA, Guanacaste, ACG, Sector Santa Rosa, Bosque San Emilio, 300m, 10.84389, -85.61384.

#### Holotype.

♀ in CNC. Specimen labels: 1. DHJPAR0024715. 2. COSTA RICA, Guanacaste, ACG, Sector Santa Rosa, Bosque San Emilio, 2.viii.1999, 10.84389°N, -85.61384°W, 300m, DHJPAR0024715. 3. San Emilio, Date: 2 Aug 99.

#### Description.

**Female.** Body color: body mostly dark except for some sternites which may be pale. Antenna color: scape, pedicel, and flagellum dark. Coxae color (pro-, meso-, metacoxa): dark, dark, dark. Femora color (pro-, meso-, metafemur): anteriorly dark/posteriorly pale, dark, dark. Tibiae color (pro-, meso-, metatibia): pale, pale, anteriorly pale/posteriorly dark. Tegula and humeral complex color: tegula dark, humeral complex half pale/half dark. Pterostigma color: mostly pale and/or transparent, with thin dark borders. Fore wing veins color: partially pigmented (a few veins may be dark but most are pale). Antenna length/body length: antenna shorter than body (head to apex of metasoma), not extending beyond anterior 0.7 metasoma length. Body in lateral view: not distinctly flattened dorso–ventrally. Body length (head to apex of metasoma): 2.7–2.8 mm. Fore wing length: 2.9–3.0 mm. Ocular–ocellar line/posterior ocellus diameter: 2.0–2.2. Interocellar distance/posterior ocellus diameter: 1.1–1.3. Antennal flagellomerus 2 length/width: 2.3–2.5. Antennal flagellomerus 14 length/width: 1.4–1.6. Length of flagellomerus 2/length of flagellomerus 14: 2.3–2.5. Tarsal claws: simple (?). Anteromesoscutum: mostly with deep, dense punctures (separated by less than 2.0 × its maximum diameter). Mesoscutellar disc: mostly smooth. Number of pits in scutoscutellar sulcus: 9 or 10. Maximum height of mesoscutellum lunules/maximum height of lateral face of mesoscutellum: 0.6–0.7. Propodeum areola: completely defined by carinae, including transverse carina extending to spiracle. Propodeum background sculpture: mostly sculptured. Mediotergite 1 length/width at posterior margin: 2.3–2.5. Mediotergite 1 shape: mostly parallel–sided for 0.5–0.7 of its length, then narrowing posteriorly so mediotergite anterior width >1.1 × posterior width. Mediotergite 1 sculpture: mostly sculptured, excavated area centrally with transverse striation inside and/or a polished knob centrally on posterior margin of mediotergite. Mediotergite 2 width at posterior margin/length: 4.0–4.3. Mediotergite 2 sculpture: with some sculpture, mostly near posterior margin. Outer margin of hypopygium: with a wide, medially folded, transparent, semi–desclerotized area; usually with 4 or more pleats. Ovipositor thickness: anterior width at most 2.0 × posterior width (beyond ovipositor constriction). Length of fore wing veins r/2RS: 1.7–1.9. Length of fore wing veins 2RS/2M: 1.9–2.0. Length of fore wing veins 2M/(RS+M)b: 0.7–0.8. Pterostigma length/width: 3.1–3.5. Point of insertion of vein r in pterostigma: clearly beyond half way point length of pterostigma. Angle of vein r with fore wing anterior margin: more or less perpendicular to fore wing margin. Shape of junction of veins r and 2RS in fore wing: evenly curved.

**Male.** Unknown.

#### Molecular data.

Sequences in BOLD: 1, barcode compliant sequences: 1.

#### Biology/ecology.

Malaise-trapped.

#### Distribution.

Costa Rica, ACG.

#### Comments.

This species is characterized by propodeum fully sculptured, without differences in sculpture between anterior and posterior halves; profemur partially, and meso- and meta- femora completely dark brown to black; and ovipositor relatively thick (anterior width 2.0 × as posterior width).

#### Etymology.

We dedicate this species to José Díaz in recognition of his diligent efforts for the ACG Programa de Sectores.

### Apanteles josejaramilloi

Fernández-Triana sp. n.

http://zoobank.org/A6F2CBCC-215F-4B47-B255-C36C1FC59506

http://species-id.net/wiki/Apanteles_josejaramilloi

Fig. 77

#### Type locality.

COSTA RICA, Guanacaste. ACG, Sector Santa Rosa, Bosque San Emilio, 300m, 10.84389, -85.61384.

#### Holotype.

♀ in CNC. Specimen labels: 1. DHJPAR0013070. 2. COSTA RICA, Guanacaste. ACG, Sector Santa Rosa, Bosque San Emilio, 300m, 10.84389°N, 85.61384°W, 8.iii.1999, DHJPAR0013070.

#### Description.

**Female.** Body color: body mostly dark except for some sternites which may be pale. Antenna color: scape, pedicel, and flagellum dark. Coxae color (pro-, meso-, metacoxa): pale, pale, dark. Femora color (pro-, meso-, metafemur): pale, pale, dorsally pale and ventrally dark. Tibiae color (pro-, meso-, metatibia): pale, pale, mostly pale but with posterior 0.2 or less dark. Tegula and humeral complex color: both pale. Pterostigma color: dark. Fore wing veins color: mostly dark (a few veins may be unpigmented). Antenna length/body length: antenna about as long as body (head to apex of metasoma); if slightly shorter, at least extending beyond anterior 0.7 metasoma length. Body in lateral view: not distinctly flattened dorso–ventrally. Body length (head to apex of metasoma): 2.7–2.8 mm. Fore wing length: 2.9–3.0 mm. Ocular–ocellar line/posterior ocellus diameter: 1.7–1.9. Interocellar distance/posterior ocellus diameter: 1.7–1.9. Antennal flagellomerus 2 length/width: 2.6–2.8. Antennal flagellomerus 14 length/width: 1.7–1.9. Length of flagellomerus 2/length of flagellomerus 14: 2.0–2.2. Tarsal claws: simple. Metafemur length/width: 3.2–3.3. Metatibia inner spur length/metabasitarsus length: 0.4–0.5. Anteromesoscutum: mostly with deep, dense punctures (separated by less than 2.0 × its maximum diameter). Mesoscutellar disc: mostly punctured. Number of pits in scutoscutellar sulcus: 7 or 8. Maximum height of mesoscutellum lunules/maximum height of lateral face of mesoscutellum: 0.4–0.5. Propodeum areola: completely defined by carinae, including transverse carina extending to spiracle. Propodeum background sculpture: mostly sculptured. Mediotergite 1 length/width at posterior margin: 2.9–3.1. Mediotergite 1 shape: mostly parallel–sided for 0.5–0.7 of its length, then narrowing posteriorly so mediotergite anterior width >1.1 × posterior width. Mediotergite 1 sculpture: mostly sculptured, excavated area centrally with transverse striation inside and/or a polished knob centrally on posterior margin of mediotergite. Mediotergite 2 width at posterior margin/length: 4.4–4.7. Mediotergite 2 sculpture: with some sculpture, mostly near posterior margin. Outer margin of hypopygium: with a wide, medially folded, transparent, semi–desclerotized area; usually with 4 or more pleats. Ovipositor thickness: about same width throughout its length. Ovipositor sheaths length/metatibial length: 0.8–0.9. Length of fore wing veins r/2RS: 1.4–1.6. Length of fore wing veins 2RS/2M: 1.7–1.8. Length of fore wing veins 2M/(RS+M)b: 0.7–0.8. Pterostigma length/width: 3.1–3.5. Angle of vein r with fore wing anterior margin: more or less perpendicular to fore wing margin. Shape of junction of veins r and 2RS in fore wing: distinctly but not strongly angled.

**Male.** Unknown.

#### Molecular data.

Sequences in BOLD: 1, barcode compliant sequences: 1.

#### Biology/ecology.

Malaise trapped.

#### Distribution.

Costa Rica, ACG.

#### Etymology.

We dedicate this species to José Jaramillo in recognition of his dilligent efforts for the ACG Programa de Computación e Informatica.

### Apanteles josemonteroi

Fernández-Triana sp. n.

http://zoobank.org/B9A26858-B4F9-4CB3-BF3B-0FFBB2D4DA47

http://species-id.net/wiki/Apanteles_josemonteroi

Figs 187 , 316

#### Type locality.

COSTA RICA, Alajuela, ACG, Sector San Cristobal, Sendero Huerta, 527m, 10.9305, -85.37223.

#### Holotype.

♀ in CNC. Specimen labels: 1 DHJPAR0011908. 2. COSTA RICA, Alajuela, ACG, Sector San Cristobal, Sendero Huerta, 20.iv.2006, 10.9305, -85.37223, 527m, DHJPAR0011908. 3. Voucher: D.H.Janzen & W.Hallwachs, DB: http://janzen.sas.upenn.edu, Area de Conservación Guanacaste, COSTA RICA, 06-SRNP-3215.

#### Paratypes.

22 ♀, 11 ♂ (BMNH, CNC, INBIO, INHS, NMNH). COSTA RICA, ACG database codes: DHJPAR0004998.

#### Description.

**Female.** Metatibia color (outer face): entirely or mostly (>0.7 metatibia length) dark brown to black, with yellow to white coloration usually restricted to anterior 0.2 or less. Fore wing veins color: veins C+Sc+R and R1 mostly brown; usually veins r, 2RS, 2M, (RS+M)b, 1CU, 2Cua, and 1m–cu partially brown; interior area of other veins, and at least part of pterostigma, usually light brown or yellowish–white. Antenna length/body length: antenna about as long as body (head to apex of metasoma); if slightly shorter, at least extending beyond anterior 0.7 metasoma length. Body length (head to apex of metasoma): 2.0 mm or less. Fore wing length: 2.1–2.2 mm. Metafemur length/width: 3.0–3.1. Mediotergite 1 length/width at posterior margin: 2.3–2.4. Mediotergite 1 maximum width/width at posterior margin: 1.4–1.5. Ovipositor sheaths length/metafemur length: 0.9. Ovipositor sheaths length/metatibia length: 0.8.

#### Molecular data.

Sequences in BOLD: 5, barcode compliant sequences: 5.

#### Biology/ecology.

Gregarious (Fig. 316). Host: Hesperiidae, *Urbanus doryssus* DHJ02.

#### Distribution.

Costa Rica, ACG.

#### Etymology.

We dedicate this species to José Montero in recognition of his diligent efforts for the ACG Programa de Parataxónomos, and Lepidoptera curatorial taxonomy for INBio, Costa Rica’s Instituto Nacional de Biodiversidad, and for ACG.

### Apanteles joseperezi

Fernández-Triana sp. n.

http://zoobank.org/5B10A0A9-E521-4503-A94E-2E8FC9C9ED01

http://species-id.net/wiki/Apanteles_joseperezi

Figs 59 , 252

Apanteles Rodriguez31 (Smith et al. 2006). Interim name provided by the authors.

#### Type locality.

COSTA RICA, Guanacaste, ACG, Sector Cacao, Estación Cacao, 1150m, 10.92691, -85.46822.

#### Holotype.

♀ in CNC. Specimen labels: 1. COSTA RICA, Guanacaste, ACG, Sector Cacao, Estación Cacao, 01/04/2001, Mariano Pereira. 2. 01-SRNP-6002, *Noctuana lactifera*, *Heliocarpus americanus*. 3. DHJPAR0003990.

#### Paratypes.

18 ♀, 6 ♂ (BMNH, CNC, INBIO, INHS, NMNH). COSTA RICA, ACG database codes: 01-SRNP-6002, 01-SRNP-6003, 01-SRNP-6012, 01-SRNP-7360, 01-SRNP-21077, 02-SRNP-23921, DHJPAR0001579.

#### Description.

**Female.** Body color: body mostly dark except for some sternites which may be pale. Antenna color: scape, pedicel, and flagellum dark. Coxae color (pro-, meso-, metacoxa): dark, dark, dark. Femora color (pro-, meso-, metafemur): pale, pale, dark. Tibiae color (pro-, meso-, metatibia): pale, pale, anteriorly pale/posteriorly dark. Tegula and humeral complex color: both dark. Pterostigma color: dark or mostly dark, with small pale area centrally. Fore wing veins color: mostly dark (a few veins may be unpigmented). Antenna length/body length: antenna shorter than body (head to apex of metasoma), not extending beyond anterior 0.7 metasoma length. Body in lateral view: not distinctly flattened dorso–ventrally. Body length (head to apex of metasoma): 2.9–3.0 mm or 3.1–3.2 mm. Fore wing length: 3.1–3.2 mm. Ocular–ocellar line/posterior ocellus diameter: 2.3–2.5. Interocellar distance/posterior ocellus diameter: 1.7–1.9. Antennal flagellomerus 2 length/width: 2.3–2.5. Antennal flagellomerus 14 length/width: 1.4–1.6. Length of flagellomerus 2/length of flagellomerus 14: 2.0–2.2. Tarsal claws: with two basal spine–like setae. Metafemur length/width: 3.0–3.1. Metatibia inner spur length/metabasitarsus length: 0.6–0.7. Anteromesoscutum: mostly with deep, dense punctures (separated by less than 2.0 × its maximum diameter). Mesoscutellar disc: with punctures near margins, central part mostly smooth. Number of pits in scutoscutellar sulcus: 7 or 8, rarely 9 or 10. Maximum height of mesoscutellum lunules/maximum height of lateral face of mesoscutellum: 0.4–0.5. Propodeum areola: completely defined by carinae, including transverse carina extending to spiracle. Propodeum background sculpture: mostly sculptured. Mediotergite 1 length/width at posterior margin: 2.6–2.8 or 2.9–3.1. Mediotergite 1 shape: mostly parallel–sided for 0.5–0.7 of its length, then narrowing posteriorly so mediotergite anterior width >1.1 × posterior width or slightly widening from anterior margin to 0.7–0.8 mediotergite length (where maximum width is reached), then narrowing towards posterior margin. Mediotergite 1 sculpture: mostly sculptured, excavated area centrally with transverse striation inside and/or a polished knob centrally on posterior margin of mediotergite. Mediotergite 2 width at posterior margin/length: 2.8–3.1 or 3.2–3.5. Mediotergite 2 sculpture: mostly smooth. Outer margin of hypopygium: inflexible (without a folded, transparent, semi–desclerotized area); with no pleats visible. Ovipositor thickness: anterior width 3.0–5.0 × posterior width (beyond ovipositor constriction). Ovipositor sheaths length/metatibial length: 0.8–0.9, rarely 1.0–1.1. Length of fore wing veins r/2RS: 2.3 or more. Length of fore wing veins 2RS/2M: 1.1–1.3. Length of fore wing veins 2M/(RS+M)b: 1.1–1.3. Pterostigma length/width: 3.1–3.5. Point of insertion of vein r in pterostigma: about half way point length of pterostigma. Angle of vein r with fore wing anterior margin: more or less perpendicular to fore wing margin. Shape of junction of veins r and 2RS in fore wing: distinctly but not strongly angled.

**Male.** Similar to female.

#### Molecular data.

Sequences in BOLD: 6, barcode compliant sequences: 5.

#### Biology/ecology.

Gregarious (Fig. 252). Host: Hesperiidae, *Noctuana lactifera*.

#### Distribution.

Costa Rica, ACG.

#### Etymology.

We dedicate this species to José Perez in recognition of his diligent efforts for the ACG Programa de Parataxónomos and Estación Biológica Caribe of ACG.

### Apanteles joserasi

Fernández-Triana sp. n.

http://zoobank.org/63DA1E6F-14F2-453C-91BC-25781C2F1A82

http://species-id.net/wiki/Apanteles_joserasi

Figs 133 , 291

#### Type locality.

COSTA RICA, Guanacaste, ACG, Sector Cacao, Naranjales, 1030m, 10.92268, -85.46405.

#### Holotype.

1 ♀ in CNC. Specimen labels: 1. DHJPAR0039764. 2. COSTA RICA, Guanacaste, ACG, Sector Cacao, Naranjales, 22.vi.2009, 10.92268° N, -85.46405° W, 1030m, DHJPAR0039764. 3. Voucher: D.H.Janzen & W.Hallwachs, DB: http://janzen.sas.upenn.edu, Area de Conservación Guanacaste, COSTA RICA, 09-SRNP-36372.

#### Description.

**Female.** Body color: body mostly dark except for some sternites which may be pale. Antenna color: scape, pedicel, and flagellum pale. Coxae color (pro-, meso-, metacoxa): pale, dark, dark. Femora color (pro-, meso-, metafemur): pale, dark, dark. Tibiae color (pro-, meso-, metatibia): pale, pale, mostly dark but anterior 0.2 or less pale. Tegula and humeral complex color: tegula pale, humeral complex half pale/half dark. Pterostigma color: entirely pale or transparent, translucent. Fore wing veins color: mostly white or entirely transparent. Antenna length/body length: antenna about as long as body (head to apex of metasoma); if slightly shorter, at least extending beyond anterior 0.7 metasoma length. Body in lateral view: not distinctly flattened dorso–ventrally. Body length (head to apex of metasoma): 2.9–3.0 mm. Fore wing length: 3.1–3.2 mm. Ocular–ocellar line/posterior ocellus diameter: 2.0–2.2. Interocellar distance/posterior ocellus diameter: 2.0–2.2. Antennal flagellomerus 2 length/width: 3.2 or more. Antennal flagellomerus 14 length/width: 2.0–2.2. Length of flagellomerus 2/length of flagellomerus 14: 2.0–2.2. Tarsal claws: simple. Metafemur length/width: 3.4–3.5. Metatibia inner spur length/metabasitarsus length: 0.4–0.5. Anteromesoscutum: mostly with shallow, dense punctures (separated by less than 2.0 × its maximum diameter). Mesoscutellar disc: mostly smooth. Number of pits in scutoscutellar sulcus: 7 or 8. Maximum height of mesoscutellum lunules/maximum height of lateral face of mesoscutellum: 0.6–0.7. Propodeum areola: completely defined by carinae, including transverse carina extending to spiracle. Propodeum background sculpture: partly sculptured, especially on anterior 0.5. Mediotergite 1 length/width at posterior margin: 2.6–2.8. Mediotergite 1 shape: mostly parallel–sided for 0.5–0.7 of its length, then narrowing posteriorly so mediotergite anterior width >1.1 × posterior width. Mediotergite 1 sculpture: mostly sculptured, excavated area centrally with transverse striation inside and/or a polished knob centrally on posterior margin of mediotergite. Mediotergite 2 width at posterior margin/length: 2.8–3.1. Mediotergite 2 sculpture: mostly smooth. Outer margin of hypopygium: with a wide, medially folded, transparent, semi–desclerotized area; usually with 4 or more pleats. Ovipositor thickness: anterior width 3.0–5.0 × posterior width (beyond ovipositor constriction). Ovipositor sheaths length/metatibial length: 1.0–1.1. Length of fore wing veins r/2RS: 2.0–2.2. Length of fore wing veins 2RS/2M: 1.9–2.0. Length of fore wing veins 2M/(RS+M)b: 0.4 or less. Pterostigma length/width: 3.6 or more. Point of insertion of vein r in pterostigma: about half way point length of pterostigma. Angle of vein r with fore wing anterior margin: more or less perpendicular to fore wing margin. Shape of junction of veins r and 2RS in fore wing: strongly angulated, sometimes with a knob.

**Male.** Unknown.

#### Molecular data.

Sequences in BOLD: 1, barcode compliant sequences: 1.

#### Biology/ecology.

Solitary (Fig. 291). Host: Hesperiidae, *Venada lamella*.

#### Distribution.

Costa Rica, ACG.

#### Comments.

The only specimen available (holotype female) is in rather poor condition.

#### Etymology.

We dedicate this species to José Eras in recognition of his diligent efforts for the ACG Comedor in Santa Rosa.

### Apanteles juanapui

Fernández-Triana sp. n.

http://zoobank.org/17633B30-C2C5-4C87-8D0D-EAAC8089B26A

http://species-id.net/wiki/Apanteles_juanapui

Fig. 128

#### Type locality.

COSTA RICA, Alajuela, ACG, Sector San Cristobal, Bosque Trampa Malaise, 815m, 10.86280, -85.38460.

#### Holotype.

♀ in CNC. Specimen labels: 1. San Gerardo: MT, San Cristobal, 26 Oct.-1 Nov. 2007. 2. DHJPAR0027635.

#### Description.

**Female.** Body color: head and mesosoma mostly dark, metasoma with some tergites and/or most of sternites pale. Antenna color: scape and/or pedicel pale, flagellum dark. Coxae color (pro-, meso-, metacoxa): pale, pale, pale. Femora color (pro-, meso-, metafemur): pale, pale, pale. Tibiae color (pro-, meso-, metatibia): pale, pale, mostly pale but with posterior 0.2 or less dark. Tegula and humeral complex color: both pale. Pterostigma color: dark. Fore wing veins color: mostly dark (a few veins may be unpigmented). Antenna length/body length: antenna about as long as body (head to apex of metasoma); if slightly shorter, at least extending beyond anterior 0.7 metasoma length. Body in lateral view: not distinctly flattened dorso–ventrally. Body length (head to apex of metasoma): 3.3–3.4 mm. Fore wing length: 3.3–3.4 mm. Ocular–ocellar line/posterior ocellus diameter: 1.7–1.9. Interocellar distance/posterior ocellus diameter: 1.7–1.9. Antennal flagellomerus 2 length/width: 2.6–2.8. Antennal flagellomerus 14 length/width: 1.4–1.6. Length of flagellomerus 2/length of flagellomerus 14: 2.0–2.2. Tarsal claws: with single basal spine–like seta. Metafemur length/width: 3.2–3.3. Metatibia inner spur length/metabasitarsus length: 0.4–0.5. Anteromesoscutum: mostly with deep, dense punctures (separated by less than 2.0 × its maximum diameter). Mesoscutellar disc: mostly smooth. Number of pits in scutoscutellar sulcus: 7 or 8. Maximum height of mesoscutellum lunules/maximum height of lateral face of mesoscutellum: 0.2–0.3. Propodeum areola: completely defined by carinae, including transverse carina extending to spiracle. Propodeum background sculpture: mostly sculptured. Mediotergite 1 length/width at posterior margin: 2.0–2.2. Mediotergite 1 shape: mostly parallel–sided for 0.5–0.7 of its length, then narrowing posteriorly so mediotergite anterior width >1.1 × posterior width. Mediotergite 1 sculpture: with some sculpture near lateral margins and/or posterior 0.2–0.4 of mediotergite. Mediotergite 2 width at posterior margin/length: 2.8–3.1. Mediotergite 2 sculpture: mostly smooth. Outer margin of hypopygium: with a wide, medially folded, transparent, semi–desclerotized area; usually with 4 or more pleats. Ovipositor thickness: anterior width at most 2.0 × posterior width (beyond ovipositor constriction). Ovipositor sheaths length/metatibial length: 1.4–1.5. Length of fore wing veins r/2RS: 1.7–1.9. Length of fore wing veins 2RS/2M: 1.1–1.3. Length of fore wing veins 2M/(RS+M)b: 0.9–1.0. Pterostigma length/width: 2.6–3.0. Point of insertion of vein r in pterostigma: about half way point length of pterostigma. Angle of vein r with fore wing anterior margin: clearly outwards, inclined towards fore wing apex. Shape of junction of veins r and 2RS in fore wing: distinctly but not strongly angled.

**Male.** Unknown.

#### Molecular data.

Sequences in BOLD: 1, barcode compliant sequences: 1.

#### Biology/ecology.

Malaise-trapped.

#### Distribution.

Costa Rica, ACG.

#### Etymology.

We dedicate this species to Juan Apu in recognition of his diligent efforts for the ACG Programa Forestal.

### Apanteles juancarrilloi

Fernández-Triana sp. n.

http://zoobank.org/7F0871C1-1DDB-49CB-B071-EC105FD764CE

http://species-id.net/wiki/Apanteles_juancarrilloi

Figs 45 , 239

Apanteles Rodriguez42 (Smith et al. 2006). Interim name provided by the authors.

#### Type locality.

COSTA RICA, Guanacaste, ACG, Sector Pitilla, Sendero Orosilito, 900m, 10.98332, -85.43623.

#### Holotype.

♀ in CNC. Specimen labels: 1. DHJPAR0038197. 2. Voucher: D.H.Janzen & W.Hallwachs, DB: http://janzen.sas.upenn.edu, Area de Conservación Guanacaste, COSTA RICA, 09-SRNP-33340.

#### Paratypes.

4 ♀ (CNC, NMNH). COSTA RICA, ACG database codes: DHJPAR0012470, DHJPAR0012471, DHJPAR0013023, DHJPAR0024704.

#### Description.

**Female.** Body color: body mostly dark except for some sternites which may be pale. Antenna color: scape, pedicel, and flagellum dark. Coxae color (pro-, meso-, metacoxa): pale, dark, dark. Femora color (pro-, meso-, metafemur): pale, pale, mostly pale but posterior 0.2 or less dark. Tibiae color (pro-, meso-, metatibia): pale, pale, mostly pale but with posterior 0.2 or less dark. Tegula and humeral complex color: both pale. Pterostigma color: dark. Fore wing veins color: mostly dark (a few veins may be unpigmented). Antenna length/body length: antenna about as long as body (head to apex of metasoma); if slightly shorter, at least extending beyond anterior 0.7 metasoma length. Body in lateral view: not distinctly flattened dorso–ventrally. Body length (head to apex of metasoma): 3.5–3.6 mm or 3.7–3.8 mm. Fore wing length: 3.5–3.6 mm or 3.7–3.8 mm. Ocular–ocellar line/posterior ocellus diameter: 1.7–1.9. Interocellar distance/posterior ocellus diameter: 1.7–1.9. Antennal flagellomerus 2 length/width: 2.3–2.5. Antennal flagellomerus 14 length/width: 1.4–1.6. Length of flagellomerus 2/length of flagellomerus 14: 2.0–2.2. Tarsal claws: simple (?). Metafemur length/width: 3.0–3.1. Metatibia inner spur length/metabasitarsus length: 0.6–0.7. Anteromesoscutum: mostly with deep, dense punctures (separated by less than 2.0 × its maximum diameter). Mesoscutellar disc: mostly smooth. Number of pits in scutoscutellar sulcus: 11 or 12. Maximum height of mesoscutellum lunules/maximum height of lateral face of mesoscutellum: 0.4–0.5. Propodeum areola: completely defined by carinae, but only partial or absent transverse carina (?). Propodeum background sculpture: mostly sculptured. Mediotergite 1 length/width at posterior margin: 2.6–2.8. Mediotergite 1 shape: mostly parallel–sided for 0.5–0.7 of its length, then narrowing posteriorly so mediotergite anterior width >1.1 × posterior width. Mediotergite 1 sculpture: mostly sculptured, excavated area centrally with transverse striation inside and/or a polished knob centrally on posterior margin of mediotergite. Mediotergite 2 width at posterior margin/length: 1.6–1.9. Mediotergite 2 sculpture: mostly smooth. Outer margin of hypopygium: with a wide, medially folded, transparent, semi–desclerotized area; usually with 4 or more pleats. Ovipositor thickness: about same width throughout its length. Ovipositor sheaths length/metatibial length: 1.8–1.9. Length of fore wing veins r/2RS: 2.3 or more. Length of fore wing veins 2RS/2M: 1.1–1.3. Length of fore wing veins 2M/(RS+M)b: 0.5–0.6. Pterostigma length/width: 2.6–3.0. Point of insertion of vein r in pterostigma: about half way point length of pterostigma. Angle of vein r with fore wing anterior margin: more or less perpendicular to fore wing margin. Shape of junction of veins r and 2RS in fore wing: distinctly but not strongly angled.

**Male.** Unknown.

#### Molecular data.

Sequences in BOLD: 26, barcode compliant sequences: 25.

#### Biology/ecology.

Solitary (Fig. 239). Host: Elachistidae, six species of *Antaeotricha*, *Stenoma* Janzen58.

#### Distribution.

Costa Rica, ACG.

#### Etymology.

We dedicate this species to Juan Carlos Carrillo in recognition of his diligent efforts for the ACG Programa de Ecoturismo.

### Apanteles juangazoi

Fernández-Triana sp. n.

http://zoobank.org/C130A607-00B2-4A2A-A965-A0C83D842D0F

http://species-id.net/wiki/Apanteles_juangazoi

Fig. 131

#### Type locality.

COSTA RICA, Alajuela, ACG, Sector San Cristobal, Rio Blanco Abajo, 500m, 10.90037, -85.37254.

#### Holotype.

♀ in CNC. Specimen labels: 1. DHJPAR0027225. 2. San Gerardo, Rio Blanco Abajo, 17-23 April 2008.

#### Description.

**Female.** Body color: body mostly dark except for some sternites which may be pale. Antenna color: scape, pedicel, and flagellum dark. Coxae color (pro-, meso-, metacoxa): dark, dark, dark. Femora color (pro-, meso-, metafemur): anteriorly dark/posteriorly pale, dark, dark. Tibiae color (pro-, meso-, metatibia): pale, anteriorly pale/posteriorly dark, anteriorly pale/posteriorly dark. Tegula and humeral complex color: both dark. Pterostigma color: dark. Fore wing veins color: partially pigmented (a few veins may be dark but most are pale). Antenna length/body length: antenna shorter than body (head to apex of metasoma), not extending beyond anterior 0.7 metasoma length. Body in lateral view: not distinctly flattened dorso–ventrally. Body length (head to apex of metasoma): 2.3–2.4 mm. Fore wing length: 2.3–2.4 mm. Ocular–ocellar line/posterior ocellus diameter: 2.0–2.2. Interocellar distance/posterior ocellus diameter: 1.7–1.9. Antennal flagellomerus 2 length/width: 2.6–2.8. Antennal flagellomerus 14 length/width: 1.4–1.6. Length of flagellomerus 2/length of flagellomerus 14: 2.0–2.2. Tarsal claws: simple. Metafemur length/width: 3.0–3.1. Metatibia inner spur length/metabasitarsus length: 0.4–0.5. Anteromesoscutum: mostly with deep, dense punctures (separated by less than 2.0 × its maximum diameter). Mesoscutellar disc: with punctures near margins, central part mostly smooth. Number of pits in scutoscutellar sulcus: 7 or 8. Maximum height of mesoscutellum lunules/maximum height of lateral face of mesoscutellum: 0.4–0.5. Propodeum areola: completely defined by carinae, including transverse carina extending to spiracle. Propodeum background sculpture: mostly sculptured. Mediotergite 1 length/width at posterior margin: 2.3–2.5. Mediotergite 1 shape: slightly widening from anterior margin to 0.7–0.8 mediotergite length (where maximum width is reached), then narrowing towards posterior margin. Mediotergite 1 sculpture: mostly sculptured, excavated area centrally with transverse striation inside and/or a polished knob centrally on posterior margin of mediotergite. Mediotergite 2 width at posterior margin/length: 3.2–3.5. Mediotergite 2 sculpture: more or less fully sculptured, with longitudinal striation. Outer margin of hypopygium: with a wide, medially folded, transparent, semi–desclerotized area; usually with 4 or more pleats. Ovipositor thickness: about same width throughout its length. Ovipositor sheaths length/metatibial length: 0.8–0.9. Length of fore wing veins r/2RS: 1.4–1.6. Length of fore wing veins 2RS/2M: 1.4–1.6. Length of fore wing veins 2M/(RS+M)b: 0.7–0.8. Pterostigma length/width: 3.1–3.5. Point of insertion of vein r in pterostigma: clearly beyond half way point length of pterostigma. Angle of vein r with fore wing anterior margin: more or less perpendicular to fore wing margin. Shape of junction of veins r and 2RS in fore wing: evenly curved.

**Male.** Unknown.

#### Molecular data.

Sequences in BOLD: 1, barcode compliant sequences: 1.

#### Biology/ecology.

Malaise-trapped.

#### Distribution.

Costa Rica, ACG.

#### Etymology.

We dedicate this species to Juan Gazo in recognition of his diligent efforts for the ACG Programa de Sectores.

### Apanteles juanhernandezi

Fernández-Triana sp. n.

http://zoobank.org/918B7F49-9A46-45AF-8EAC-BE4E564D4A10

http://species-id.net/wiki/Apanteles_juanhernandezi

Figs 134 , 292

#### Type locality.

COSTA RICA, Alajuela, ACG, Sector San Cristobal, Bosque Trampa Malaise, 815m, 10.86280, -85.38460.

#### Holotype.

♀ in CNC. Specimen labels: 1. DHJPAR0027672. 2. San Gerardo, MT, San Cristobal, 18-24 Mar. 2008.

#### Description.

**Female.** Body color: head dark, mesosoma dark with parts of axillar complex pale, metasoma with some mediotergites, most laterotergites, sternites, and/or hypopygium pale. Antenna color: scape, pedicel, and flagellum pale. Coxae color (pro-, meso-, metacoxa): pale, pale, pale. Femora color (pro-, meso-, metafemur): pale, pale, mostly pale but posterior 0.2 or less dark. Tibiae color (pro-, meso-, metatibia): pale, pale, anteriorly pale/posteriorly dark. Tegula and humeral complex color: both pale. Pterostigma color: dark with pale spot at base. Fore wing veins color: mostly dark (a few veins may be unpigmented). Antenna length/body length: antenna about as long as body (head to apex of metasoma); if slightly shorter, at least extending beyond anterior 0.7 metasoma length. Body in lateral view: not distinctly flattened dorso–ventrally. Body length (head to apex of metasoma): 2.7–2.8 mm. Fore wing length: 3.1–3.2 mm. Ocular–ocellar line/posterior ocellus diameter: 1.7–1.9. Interocellar distance/posterior ocellus diameter: 1.7–1.9. Antennal flagellomerus 2 length/width: 2.9–3.1. Tarsal claws: with single basal spine–like seta. Metafemur length/width: 3.4–3.5. Anteromesoscutum: mostly with deep, dense punctures (separated by less than 2.0 × its maximum diameter). Mesoscutellar disc: with a few sparse punctures. Number of pits in scutoscutellar sulcus: 7 or 8. Maximum height of mesoscutellum lunules/maximum height of lateral face of mesoscutellum: 0.4–0.5. Propodeum areola: completely defined by carinae, including transverse carina extending to spiracle. Propodeum background sculpture: mostly sculptured. Mediotergite 1 length/width at posterior margin: 3.2–3.4. Mediotergite 1 shape: mostly parallel–sided for 0.5–0.7 of its length, then narrowing posteriorly so mediotergite anterior width >1.1 × posterior width. Mediotergite 1 sculpture: mostly sculptured, excavated area centrally with transverse striation inside and/or a polished knob centrally on posterior margin of mediotergite. Mediotergite 2 width at posterior margin/length: 4.8 or more. Mediotergite 2 sculpture: mostly smooth. Outer margin of hypopygium: with a wide, medially folded, transparent, semi–desclerotized area; usually with 4 or more pleats. Ovipositor thickness: about same width throughout its length. Ovipositor sheaths length/metatibial length: 0.6–0.7. Length of fore wing veins r/2RS: 1.7–1.9. Length of fore wing veins 2RS/2M: 1.1–1.3. Length of fore wing veins 2M/(RS+M)b: 0.5–0.6. Pterostigma length/width: 2.6–3.0. Point of insertion of vein r in pterostigma: about half way point length of pterostigma. Angle of vein r with fore wing anterior margin: clearly outwards, inclined towards fore wing apex. Shape of junction of veins r and 2RS in fore wing: strongly angulated, sometimes with a knob.

**Male.** Unknown.

#### Molecular data.

Sequences in BOLD: 3, barcode compliant sequences: 3.

#### Biology/ecology.

Solitary (Fig. 292). Hosts: Crambidae, *Triuncidia* eupalusalisDHJ02, *Aponia minnithalis*; Gelechiidae, gelJanzen01 Janzen294.

#### Distribution.

Costa Rica, ACG.

#### Comment.

This species is characterized by extensive yellow coloration, mesoscutellar disc mostly smooth, mediotergite 2 width at posterior margin 5.4 × its length, and metafemur relatively thin (its length 3.5 × its width).

#### Etymology.

We dedicate this species to Juan Hernández in recognition of his diligent efforts for the ACG Unidad de Transporte.

### Apanteles juanlopezi

Fernández-Triana sp. n.

http://zoobank.org/08F4CA22-0F1E-41D5-949E-23BE120CFD80

http://species-id.net/wiki/Apanteles_juanlopezi

Fig. 66

#### Type locality.

COSTA RICA, Alajuela, ACG, Sector Rincon Rain Forest, Estación Caribe, 415m, 10.90187, -85.27495.

#### Holotype.

♀ in CNC. Specimen labels: 1. DHJPAR0043018. 2. COSTA RICA, Guanacaste, ACG, Sector Rincon Rain Forest, Estación Caribe, 16.ii.2011, 10.90187°N, -85.27495°W, 415m, DHJPAR0043018. 3. Voucher: D.H.Janzen & W.Hallwachs, DB: http://janzen.sas.upenn.edu, Area de Conservación Guanacaste, COSTA RICA, 11-SRNP-40820.

#### Paratypes.

1 ♀ (CNC). COSTA RICA: Guanacaste, ACG database code: DHJPAR0040377.

#### Description.

**Female.** Body color: body mostly dark except for some sternites which may be pale. Antenna color: scape and/or pedicel pale, flagellum dark. Coxae color (pro-, meso-, metacoxa): dark, dark, dark. Femora color (pro-, meso-, metafemur): pale, pale, mostly pale but posterior 0.2 or less dark. Tibiae color (pro-, meso-, metatibia): pale, pale, pale. Tegula and humeral complex color: both pale. Pterostigma color: dark with pale spot at base. Fore wing veins color: mostly dark (a few veins may be unpigmented). Antenna length/body length: antenna about as long as body (head to apex of metasoma); if slightly shorter, at least extending beyond anterior 0.7 metasoma length. Body in lateral view: not distinctly flattened dorso–ventrally. Body length (head to apex of metasoma): 3.3–3.4 mm. Fore wing length: 3.3–3.4 mm. Ocular–ocellar line/posterior ocellus diameter: 2.3–2.5. Interocellar distance/posterior ocellus diameter: 1.7–1.9. Antennal flagellomerus 2 length/width: 2.6–2.8. Antennal flagellomerus 14 length/width: 1.4–1.6. Length of flagellomerus 2/length of flagellomerus 14: 2.3–2.5. Tarsal claws: with single basal spine–like seta. Metafemur length/width: 3.0–3.1. Metatibia inner spur length/metabasitarsus length: 0.4–0.5. Anteromesoscutum: mostly with deep, dense punctures (separated by less than 2.0 × its maximum diameter). Mesoscutellar disc: with punctures near margins, central part mostly smooth. Number of pits in scutoscutellar sulcus: 7 or 8. Maximum height of mesoscutellum lunules/maximum height of lateral face of mesoscutellum: 0.4–0.5. Propodeum areola: completely defined by carinae, including transverse carina extending to spiracle. Propodeum background sculpture: mostly sculptured. Mediotergite 1 length/width at posterior margin: 1.7–1.9. Mediotergite 1 shape: clearly widening towards posterior margin. Mediotergite 1 sculpture: mostly sculptured, excavated area centrally with transverse striation inside and/or a polished knob centrally on posterior margin of mediotergite. Mediotergite 2 width at posterior margin/length: 4.0–4.3. Mediotergite 2 sculpture: mostly smooth, with weak sculpture on anterior margin. Outer margin of hypopygium: with a wide, medially folded, transparent, semi–desclerotized area; usually with 4 or more pleats. Ovipositor thickness: about same width throughout its length. Ovipositor sheaths length/metatibial length: 1.4–1.5. Length of fore wing veins r/2RS: 1.4–1.6. Length of fore wing veins 2RS/2M: 1.7–1.8. Length of fore wing veins 2M/(RS+M)b: 0.5–0.6. Pterostigma length/width: 2.6–3.0. Point of insertion of vein r in pterostigma: clearly beyond half way point length of pterostigma. Angle of vein r with fore wing anterior margin: clearly outwards, inclined towards fore wing apex. Shape of junction of veins r and 2RS in fore wing: distinctly but not strongly angled.

**Male.** Unknown.

#### Molecular data.

Sequences in BOLD: 7, barcode compliant sequences: 7.

#### Biology/ecology.

Solitary. Host: Tortricidae, *Paramorbia* Brown001DHJ03.

#### Distribution.

Costa Rica, ACG.

#### Etymology.

We dedicate this species to Juan López in recognition of his diligent efforts for the ACG Programa de Seguridad.

### Apanteles juanmatai

Fernández-Triana sp. n.

http://zoobank.org/D59BF5ED-4196-4FFA-B778-D4ADBD100E07

http://species-id.net/wiki/Apanteles_juanmatai

Figs 181 , 317

Apanteles Rodriguez98 (Smith et al. 2006). Interim name provided by the authors.

#### Type locality.

COSTA RICA, Guanacaste, ACG, Sector Cacao, Estación Cacao, 1150m, 10.92691, -85.46822.

#### Holotype.

♀ in CNC. Specimen labels: 1. DHJPAR0012791. 2. COSTA RICA, Guanacaste, ACG, Sector Cacao, Estación Cacao, 3.xii.2006, 1150m, 10.92691, -85.46822, 1150m, DHJPAR0012791.

#### Paratypes.

17 ♀, 6 ♂ (BMNH, CNC, INBIO, INHS, NMNH). COSTA RICA, ACG database codes: DHJPAR0011960, DHJPAR0012791.

#### Description.

**Female.** Metatibia color (outer face): entirely or mostly (>0.7 metatibia length) dark brown to black, with yellow to white coloration usually restricted to anterior 0.2 or less. Fore wing veins color: veins C+Sc+R and R1 with brown coloration restricted narrowly to borders, interior area of those veins and pterostigma (and sometimes veins r, 2RS and 2M) transparent or white; other veins mostly transparent. Antenna length/body length: antenna shorter than body (head to apex of metasoma), not extending beyond anterior 0.7 metasoma length. Body length (head to apex of metasoma): 2.1–2.2 mm, 2.3–2.4 mm, rarely 2.0 mm or less. Fore wing length: 2.3–2.4 mm or 2.5–2.6 mm. Metafemur length/width: 2.6–2.7 or 2.8–2.9. Mediotergite 1 length/width at posterior margin: 2.5–2.6. Mediotergite 1 maximum width/width at posterior margin: 1.6–1.7. Ovipositor sheaths length/metafemur length: 0.7 or 0.8. Ovipositor sheaths length/metatibia length: 0.5 or 0.6.

#### Molecular data.

Sequences in BOLD: 10, barcode compliant sequences: 10.

#### Biology/ecology.

Gregarious (Fig. 317). Host: Hesperiidae, *Phocides lilea*.

#### Distribution.

Costa Rica, ACG.

#### Etymology.

We dedicate this species to Juan Mata in recognition of his diligent efforts for the ACG biodiversity inventory information and specimen management by INBio, Costa Rica’s Instituto Nacional de Biodiverisdad.

### Apanteles juanvictori

Fernández-Triana sp. n.

http://zoobank.org/87F73F3B-384A-4FE2-B966-F70EA904FC8B

http://species-id.net/wiki/Apanteles_juanvictori

Figs 11 , 214

Apanteles Rodriguez69. Smith et al. (2008). Interim name provided by the authors.

#### Type locality.

COSTA RICA, Guanacaste, ACG, Sector Cacao, Sendero Ponderosa, 1060m, 10.91460, -85.46262.

#### Holotype.

♀ in CNC. Specimen labels: 1. Costa Rica: Guanacaste, ACG, Sector Cacao, Sendero Ponderosa, 07.ii.2003, 1060m, 10.91460, -85.46262, 03-SRNP-3275.

#### Paratypes.

43 ♀, 10 ♂ (BMNH, CNC, INBIO, INHS, NMNH). COSTA RICA, ACG database codes: See Appendix 2 for detailed label data.

#### Description.

**Female.** Body color: body mostly dark except for some sternites which may be pale. Antenna color: scape, pedicel, and flagellum dark. Coxae color (pro-, meso-, metacoxa): dark, dark, dark. Femora color (pro-, meso-, metafemur): anteriorly dark/posteriorly pale, dark, dark. Tibiae color (pro-, meso-, metatibia): pale, pale, mostly dark but anterior 0.2 or less pale. Tegula and humeral complex color: tegula pale, humeral complex half pale/half dark. Pterostigma color: mostly pale and/or transparent, with thin dark borders. Fore wing veins color: partially pigmented (a few veins may be dark but most are pale). Antenna length/body length: antenna about as long as body (head to apex of metasoma); if slightly shorter, at least extending beyond anterior 0.7 metasoma length. Body in lateral view: not distinctly flattened dorso–ventrally. Body length (head to apex of metasoma): 2.9–3.0 mm, 3.1–3.2 mm, rarely 3.3–3.4 mm. Fore wing length: 3.3–3.4 mm, rarely 3.1–3.2 mm. Ocular–ocellar line/posterior ocellus diameter: 2.3–2.5. Interocellar distance/posterior ocellus diameter: 1.7–1.9. Antennal flagellomerus 2 length/width: 2.9–3.1. Antennal flagellomerus 14 length/width: 1.7–1.9. Length of flagellomerus 2/length of flagellomerus 14: 2.0–2.2. Tarsal claws: with single basal spine–like seta. Metafemur length/width: 3.0–3.1. Metatibia inner spur length/metabasitarsus length: 0.4–0.5. Anteromesoscutum: mostly with deep, dense punctures (separated by less than 2.0 × its maximum diameter). Mesoscutellar disc: mostly punctured. Number of pits in scutoscutellar sulcus: 7 or 8. Maximum height of mesoscutellum lunules/maximum height of lateral face of mesoscutellum: 0.4–0.5. Propodeum areola: completely defined by carinae, including transverse carina extending to spiracle. Propodeum background sculpture: mostly sculptured. Mediotergite 1 length/width at posterior margin: 2.0–2.2. Mediotergite 1 shape: mostly parallel–sided for 0.5–0.7 of its length, then narrowing posteriorly so mediotergite anterior width >1.1 × posterior width. Mediotergite 1 sculpture: mostly sculptured, excavated area centrally with transverse striation inside and/or a polished knob centrally on posterior margin of mediotergite. Mediotergite 2 width at posterior margin/length: 3.2–3.5. Mediotergite 2 sculpture: mostly smooth. Outer margin of hypopygium: with a wide, medially folded, transparent, semi–desclerotized area; usually with 4 or more pleats. Ovipositor thickness: anterior width at most 2.0 × posterior width (beyond ovipositor constriction). Ovipositor sheaths length/metatibial length: 1.2–1.3. Length of fore wing veins r/2RS: 1.7–1.9. Length of fore wing veins 2RS/2M: 1.1–1.3. Length of fore wing veins 2M/(RS+M)b: 0.7–0.8. Pterostigma length/width: 3.6 or more. Point of insertion of vein r in pterostigma: about half way point length of pterostigma. Angle of vein r with fore wing anterior margin: more or less perpendicular to fore wing margin. Shape of junction of veins r and 2RS in fore wing: distinctly but not strongly angled.

**Male.** As in female, with narrower mediotergite 1.

#### Molecular data.

Sequences in BOLD: 11, barcode compliant sequences: 11.

#### Biology/ecology.

Gregarious (Fig. 214). Host: Elachistidae, *Stenoma patens*, *Stenoma patens* DHJ03, *Stenoma patens* DHJ04.

#### Distribution.

Costa Rica, ACG.

#### Etymology.

We dedicate this species to Juan Victor in recognition of his diligent efforts for the ACG Programa de Seguridad.

### Apanteles juliodiazi

Fernández-Triana sp. n.

http://zoobank.org/61A58A43-7796-49BA-AB15-C7E5C3BC567E

http://species-id.net/wiki/Apanteles_juliodiazi

Fig. 135

#### Type locality.

COSTA RICA, Guanacaste, ACG, Sector Cacao, Sendero Cima, 1460m, 10.93328, -85.45729.

#### Holotype.

♀ in CNC. Specimen labels: 1. COSTA RICA, Guanacaste, ACG, Sector Cacao, Sendero Cima, 08.ix.1999, 1460m, 10.93328, -85.45729, DHJPAR0012515.

#### Paratypes.

1 ♀, 2 ♂ (CNC). COSTA RICA: ACG database codes: DHJPAR0012518, DHJPAR0013098, DHJPAR0013099.

#### Description.

**Female.** Body color: head pale, mesosoma extensively pale (anteromesoscutum and scutellar disc). Antenna color: scape and/or pedicel pale, flagellum dark. Coxae color (pro-, meso-, metacoxa): pale, pale, partially pale/partially dark. Femora color (pro-, meso-, metafemur): pale, pale, pale. Tibiae color (pro-, meso-, metatibia): pale, pale, pale with anterior 0.1 and posterior 0.2 dark. Tegula and humeral complex color: both pale. Pterostigma color: dark. Fore wing veins color: mostly dark (a few veins may be unpigmented). Antenna length/body length: antenna about as long as body (head to apex of metasoma); if slightly shorter, at least extending beyond anterior 0.7 metasoma length. Body in lateral view: not distinctly flattened dorso–ventrally. Body length (head to apex of metasoma): 3.7–3.8 mm. Fore wing length: 4.0 mm or more. Ocular–ocellar line/posterior ocellus diameter: 2.0–2.2. Interocellar distance/posterior ocellus diameter: 2.0–2.2. Antennal flagellomerus 2 length/width: 2.9–3.1. Antennal flagellomerus 14 length/width: 1.4–1.6. Length of flagellomerus 2/length of flagellomerus 14: 2.3–2.5. Tarsal claws: pectinate. Metafemur length/width: 3.2–3.3. Metatibia inner spur length/metabasitarsus length: 0.4–0.5. Anteromesoscutum: mostly with shallow, dense punctures (separated by less than 2.0 × its maximum diameter). Mesoscutellar disc: mostly smooth. Number of pits in scutoscutellar sulcus: scutoscutellar sulcus almost obliterated, with less than 4 small impressions. Maximum height of mesoscutellum lunules/maximum height of lateral face of mesoscutellum: 0.4–0.5. Propodeum areola: completely defined by carinae, but only partial or absent transverse carina. Propodeum background sculpture: mostly smooth except around the areola. Mediotergite 1 length/width at posterior margin: 2.9–3.1. Mediotergite 1 shape: mostly parallel–sided for 0.5–0.7 of its length, then narrowing posteriorly so mediotergite anterior width >1.1 × posterior width. Mediotergite 1 sculpture: mostly smooth. Mediotergite 2 width at posterior margin/length: 4.0–4.3. Mediotergite 2 sculpture: mostly smooth. Outer margin of hypopygium: with a wide, medially folded, transparent, semi–desclerotized area; usually with 4 or more pleats. Ovipositor thickness: about same width throughout its length. Ovipositor sheaths length/metatibial length: 0.8–0.9. Length of fore wing veins r/2RS: 2.0–2.2. Length of fore wing veins 2RS/2M: 0.9–1.0. Length of fore wing veins 2M/(RS+M)b: 0.7–0.8. Pterostigma length/width: 3.6 or more. Point of insertion of vein r in pterostigma: about half way point length of pterostigma. Angle of vein r with fore wing anterior margin: more or less perpendicular to fore wing margin. Shape of junction of veins r and 2RS in fore wing: distinctly but not strongly angled.

**Male.** Like female, but mediotergite 1 is darker, with brown spots on anterior and posterior margins, in one specimen almost the whole mediotergite is dark brown, with just a small central area which is whitish.

#### Molecular data.

Sequences in BOLD: 7, barcode compliant sequences: 5.

#### Biology/ecology.

Malaise-trapped.

#### Distribution.

Costa Rica, ACG.

#### Comments.

This species is characterized by its orange head and mesosternum, scutello-scutellar sulcus almost obliterated, head in frontal view rather elongate, tarsal claws pectinated, and propodeal areola open basally and without transverse carinae.

#### Etymology.

We dedicate this species to Julio Diaz in recognition of his diligent efforts for the ACG Programa de Seguridad y Prevencion de Incendios.

### Apanteles juniorlopezi

Fernández-Triana sp. n.

http://zoobank.org/57F93B18-B616-445A-B8E7-3A2128C4D84F

http://species-id.net/wiki/Apanteles_juniorlopezi

Figs 12 , 215

Apanteles Rodriguez67. Smith et al. (2008). Interim name provided by the authors.

#### Type locality.

COSTA RICA, Guanacaste, ACG, Sector Del Oro, Quebrada Raiz, 280m, 11.02865, -85.48669.

#### Holotype.

♀ in CNC. Specimen labels: 1. Costa Rica: Guanacaste, Sector Del Oro, Quebrada Raiz, 19.ii.2002, 280m, 11.02865, -85.48669, DHJPAR0003086.

#### Paratypes.

14 ♀, 2 ♂ (BMNH, CNC, INBIO, INHS, NMNH). COSTA RICA, ACG database codes: DHJPAR0003086, DHJPAR0034222, DHJPAR0034276, 02-SRNP-5765.

#### Description.

**Female.** Body color: body mostly dark except for some sternites which may be pale. Antenna color: scape, pedicel, and flagellum dark. Coxae color (pro-, meso-, metacoxa): dark, dark, dark. Femora color (pro-, meso-, metafemur): anteriorly dark/posteriorly pale, dark, dark. Tibiae color (pro-, meso-, metatibia): pale, pale, mostly dark but anterior 0.2 or less pale. Tegula and humeral complex color: tegula pale, humeral complex half pale/half dark. Pterostigma color: mostly pale and/or transparent, with thin dark borders. Fore wing veins color: partially pigmented (a few veins may be dark but most are pale). Antenna length/body length: antenna about as long as body (head to apex of metasoma); if slightly shorter, at least extending beyond anterior 0.7 metasoma length. Body in lateral view: not distinctly flattened dorso–ventrally. Body length (head to apex of metasoma): 2.5–2.6 mm. Fore wing length: 2.5–2.6 mm. Ocular–ocellar line/posterior ocellus diameter: 2.3–2.5. Interocellar distance/posterior ocellus diameter: 1.4–1.6. Antennal flagellomerus 2 length/width: 2.3–2.5. Antennal flagellomerus 14 length/width: 1.1–1.3. Length of flagellomerus 2/length of flagellomerus 14: 2.0–2.2. Tarsal claws: with single basal spine–like seta. Metafemur length/width: 3.2–3.3. Metatibia inner spur length/metabasitarsus length: 0.4–0.5. Anteromesoscutum: mostly with deep, dense punctures (separated by less than 2.0 × its maximum diameter). Mesoscutellar disc: with punctures near margins, central part mostly smooth. Number of pits in scutoscutellar sulcus: 9 or 10. Maximum height of mesoscutellum lunules/maximum height of lateral face of mesoscutellum: 0.4–0.5. Propodeum areola: completely defined by carinae, including transverse carina extending to spiracle. Propodeum background sculpture: partly sculptured, especially on anterior 0.5. Mediotergite 1 length/width at posterior margin: 2.0–2.2. Mediotergite 1 shape: slightly widening from anterior margin to 0.7–0.8 mediotergite length (where maximum width is reached), then narrowing towards posterior margin. Mediotergite 1 sculpture: mostly sculptured, excavated area centrally with transverse striation inside and/or a polished knob centrally on posterior margin of mediotergite. Mediotergite 2 width at posterior margin/length: 2.8–3.1. Mediotergite 2 sculpture: mostly smooth. Outer margin of hypopygium: with a wide, medially folded, transparent, semi–desclerotized area; usually with 4 or more pleats. Ovipositor thickness: anterior width at most 2.0 × posterior width (beyond ovipositor constriction). Ovipositor sheaths length/metatibial length: 1.0–1.1, rarely 1.2–1.3. Length of fore wing veins r/2RS: 1.4–1.6. Length of fore wing veins 2RS/2M: 1.9–2.0. Length of fore wing veins 2M/(RS+M)b: 0.7–0.8. Pterostigma length/width: 3.1–3.5. Point of insertion of vein r in pterostigma: about half way point length of pterostigma. Angle of vein r with fore wing anterior margin: clearly outwards, inclined towards fore wing apex. Shape of junction of veins r and 2RS in fore wing: distinctly but not strongly angled.

**Male.** As in female.

#### Molecular data.

Sequences in BOLD: 7, barcode compliant sequences: 7.

#### Biology/ecology.

Gregarious (Fig. 215). Host: Elachistidae, *Anadasmus* Janzen25, *Anadasmus* Janzen26.

#### Distribution.

Costa Rica, ACG.

#### Etymology.

We dedicate this species to Junior López in recognition of his diligent efforts for the ACG Programa de Seguridad.

### Apanteles keineraragoni

Fernández-Triana sp. n.

http://zoobank.org/1BC887E2-4C38-4E5B-885C-96088179581D

http://species-id.net/wiki/Apanteles_keineraragoni

Figs 136 , 293

#### Type locality.

COSTA RICA, Alajuela, ACG, Sector Rincon Rain Forest, Conguera, 420m, 10.91589, -85.26631.

#### Holotype.

♀ in CNC. Specimen labels: 1. DHJPAR0041926. 2. COSTA RICA, Alajuela, ACG, Sector Rincon Rain Forest, Conguera, 16.xii.2010, 10.91589°N, -85.26631°W, 420m, DHJPAR0041926. 3. Voucher: D.H.Janzen & W.Hallwachs, DB: http://janzen.sas.upenn.edu, Area de Conservación Guanacaste, COSTA RICA, 10-SRNP-44887.

#### Paratypes.

1 ♀, 1 ♂ (CNC). COSTA RICA, ACG database codes: DHJPAR0041935, DHJPAR0042997.

#### Description.

**Female.** Body color: body mostly dark except for some sternites which may be pale. Antenna color: scape and/or pedicel pale, flagellum dark. Coxae color (pro-, meso-, metacoxa): dark, dark, dark. Femora color (pro-, meso-, metafemur): anteriorly dark/posteriorly pale, dark, dark. Tibiae color (pro-, meso-, metatibia): pale, pale, mostly dark but anterior 0.2 or less pale. Tegula and humeral complex color: both dark. Pterostigma color: dark with pale spot at base. Fore wing veins color: mostly dark (a few veins may be unpigmented). Antenna length/body length: antenna about as long as body (head to apex of metasoma); if slightly shorter, at least extending beyond anterior 0.7 metasoma length. Body in lateral view: not distinctly flattened dorso–ventrally. Body length (head to apex of metasoma): 2.5–2.6 mm. Fore wing length: 2.7–2.8 mm. Ocular–ocellar line/posterior ocellus diameter: 2.3–2.5. Interocellar distance/posterior ocellus diameter: 1.7–1.9. Antennal flagellomerus 2 length/width: 2.9–3.1. Antennal flagellomerus 14 length/width: 1.4–1.6. Length of flagellomerus 2/length of flagellomerus 14: 2.0–2.2. Tarsal claws: simple. Metafemur length/width: 3.4–3.5. Metatibia inner spur length/metabasitarsus length: 0.4–0.5. Anteromesoscutum: mostly with deep, dense punctures (separated by less than 2.0 × its maximum diameter). Mesoscutellar disc: with punctures near margins, central part mostly smooth. Number of pits in scutoscutellar sulcus: 7 or 8. Maximum height of mesoscutellum lunules/maximum height of lateral face of mesoscutellum: 0.6–0.7. Propodeum areola: completely defined by carinae, including transverse carina extending to spiracle. Propodeum background sculpture: partly sculptured, especially on anterior 0.5. Mediotergite 1 length/width at posterior margin: 2.6–2.8. Mediotergite 1 shape: mostly parallel–sided for 0.5–0.7 of its length, then narrowing posteriorly so mediotergite anterior width >1.1 × posterior width. Mediotergite 1 sculpture: mostly sculptured, excavated area centrally with transverse striation inside and/or a polished knob centrally on posterior margin of mediotergite. Mediotergite 2 width at posterior margin/length: 4.4–4.7. Mediotergite 2 sculpture: with some sculpture, mostly near posterior margin. Outer margin of hypopygium: with a wide, medially folded, transparent, semi–desclerotized area; usually with 4 or more pleats. Ovipositor thickness: about same width throughout its length. Ovipositor sheaths length/metatibial length: 0.6–0.7. Length of fore wing veins r/2RS: 1.4–1.6. Length of fore wing veins 2RS/2M: 1.1–1.3. Length of fore wing veins 2M/(RS+M)b: 0.9–1.0. Pterostigma length/width: 2.6–3.0. Point of insertion of vein r in pterostigma: about half way point length of pterostigma. Angle of vein r with fore wing anterior margin: more or less perpendicular to fore wing margin. Shape of junction of veins r and 2RS in fore wing: strongly angulated, sometimes with a knob.

**Male.** The only specimen available is in poor condition.

#### Molecular data.

Sequences in BOLD: 5, barcode compliant sequences: 5.

#### Biology/ecology.

Solitary (Fig. 293). Hosts: Crambidae, *Neurophyseta clymenalis*.

#### Distribution.

Costa Rica, ACG.

#### Comments.

The humeral complex has a small area on outer margin that is slightly lighter in color than the rest, but we still consider it as fully brown – and thus it is coded as such in the Lucid software.

#### Etymology.

We dedicate this species to Keiner Aragón recognition of his diligent efforts for the ACG Programa de Parataxónomos and Estación Biológica Botarrama.

### Apanteles laurahuberae

Fernández-Triana sp. n.

http://zoobank.org/09AF6181-B47F-40EB-ACB5-1B169A76854A

http://species-id.net/wiki/Apanteles_laurahuberae

Fig. 107

#### Type locality.

COSTA RICA, Alajuela, ACG, Sector Rincon Rain Forest, Vado Rio Francia, 400m, 10.90093, -85.28915.

#### Holotype.

♀ in CNC. Specimen labels: 1. DHJPAR0026209. 2. Caribe, Rio Francia, 19–25-Mar-2008.

#### Paratypes.

1 ♀ (CNC). COSTA RICA: Guanacaste, ACG database code: DHJPAR0026208.

#### Description.

**Female.** Body color: body mostly dark except for some sternites which may be pale. Antenna color: scape, pedicel, and flagellum dark. Coxae color (pro-, meso-, metacoxa): dark, dark, dark. Femora color (pro-, meso-, metafemur): pale, pale, anteriorly pale/posteriorly dark. Tibiae color (pro-, meso-, metatibia): pale, pale, mostly dark but anterior 0.2 or less pale. Tegula and humeral complex color: both dark. Pterostigma color: dark. Fore wing veins color: mostly dark (a few veins may be unpigmented). Antenna length/body length: antenna about as long as body (head to apex of metasoma); if slightly shorter, at least extending beyond anterior 0.7 metasoma length. Body in lateral view: not distinctly flattened dorso–ventrally. Body length (head to apex of metasoma): 2.0 mm or less or 2.1–2.2 mm. Fore wing length: 2.1–2.2 mm. Ocular–ocellar line/posterior ocellus diameter: 2.3–2.5. Interocellar distance/posterior ocellus diameter: 2.0–2.2. Antennal flagellomerus 2 length/width: 3.2 or more. Tarsal claws: simple. Metafemur length/width: 3.4–3.5. Metatibia inner spur length/metabasitarsus length: 0.4–0.5. Anteromesoscutum: mostly with shallow, dense punctures (separated by less than 2.0 × its maximum diameter). Mesoscutellar disc: mostly punctured. Number of pits in scutoscutellar sulcus: 5 or 6 or 7 or 8. Maximum height of mesoscutellum lunules/maximum height of lateral face of mesoscutellum: 0.4–0.5. Propodeum areola: completely defined by carinae, including transverse carina extending to spiracle. Propodeum background sculpture: partly sculptured, especially on anterior 0.5. Mediotergite 1 length/width at posterior margin: 3.5–3.7. Mediotergite 1 shape: mostly parallel–sided for 0.5–0.7 of its length, then narrowing posteriorly so mediotergite anterior width >1.1 × posterior width. Mediotergite 1 sculpture: mostly sculptured, excavated area centrally with transverse striation inside and/or a polished knob centrally on posterior margin of mediotergite. Mediotergite 2 width at posterior margin/length: 3.2–3.5. Mediotergite 2 sculpture: mostly smooth. Outer margin of hypopygium: with a wide, medially folded, transparent, semi–desclerotized area; usually with 4 or more pleats. Ovipositor thickness: about same width throughout its length. Ovipositor sheaths length/metatibial length: 0.6–0.7. Length of fore wing veins r/2RS: 1.4–1.6. Length of fore wing veins 2RS/2M: 1.1–1.3. Length of fore wing veins 2M/(RS+M)b: 0.9–1.0. Pterostigma length/width: 2.6–3.0. Point of insertion of vein r in pterostigma: about half way point length of pterostigma. Angle of vein r with fore wing anterior margin: clearly outwards, inclined towards fore wing apex. Shape of junction of veins r and 2RS in fore wing: distinctly but not strongly angled.

**Male.** Unknown.

#### Molecular data.

Sequences in BOLD: 2, barcode compliant sequences: 2.

#### Biology/ecology.

Malaise-trapped.

#### Distribution.

Costa Rica, ACG.

#### Etymology.

The senior author dedicates this species to Laura Huber, daughter of John Huber (CNC, Ottawa) as an appreciation for John Huber’s support, especially reading and improving several drafts of this paper.

### Apanteles laurenmoralesae

Fernández-Triana sp. n.

http://zoobank.org/40C90BB4-29B9-4DC6-99A0-450306EBDAA3

http://species-id.net/wiki/Apanteles_laurenmoralesae

Fig. 13

Apanteles Rodriguez106. Smith et al. (2008). Interim name provided by the authors.

#### Type locality.

COSTA RICA, Alajuela, ACG, Brasilia, Moga, 320m, 11.01227, -85.34929.

#### Holotype.

♀ in CNC. Specimen labels: 1. Voucher: D.H.Janzen & W.Hallwachs, DB: http://janzen.sas.upenn.edu, Area de Conservación Guanacaste, COSTA RICA, 09-SRNP-65331. 2. DHJPAR0035502.

#### Paratypes.

2 ♀, 5 ♂ (BMNH, CNC, INBIO, NMNH). COSTA RICA, ACG database codes: DHJPAR0005172, DHJPAR0005174, DHJPAR0005176, DHJPAR0035495, DHJPAR0039767.

#### Description.

**Female.** Body color: body mostly dark except for some sternites which may be pale. Antenna color: scape, pedicel, and flagellum dark. Coxae color (pro-, meso-, metacoxa): dark, dark, dark. Femora color (pro-, meso-, metafemur): anteriorly dark/posteriorly pale, dark, dark. Tibiae color (pro-, meso-, metatibia): pale, pale, anteriorly pale/posteriorly dark. Tegula and humeral complex color: tegula pale, humeral complex half pale/half dark. Pterostigma color: mostly pale and/or transparent, with thin dark borders. Fore wing veins color: partially pigmented (a few veins may be dark but most are pale). Antenna length/body length: antenna shorter than body (head to apex of metasoma), not extending beyond anterior 0.7 metasoma length. Body in lateral view: not distinctly flattened dorso–ventrally. Body length (head to apex of metasoma): 3.1–3.2 mm or 3.3–3.4 mm. Fore wing length: 3.1–3.2 mm or 3.3–3.4 mm. Ocular–ocellar line/posterior ocellus diameter: 2.0–2.2. Interocellar distance/posterior ocellus diameter: 2.0–2.2. Antennal flagellomerus 2 length/width: 2.3–2.5. Antennal flagellomerus 14 length/width: 1.4–1.6. Length of flagellomerus 2/length of flagellomerus 14: 2.0–2.2. Tarsal claws: with single basal spine–like seta. Metafemur length/width: 3.2–3.3. Metatibia inner spur length/metabasitarsus length: 0.4–0.5. Anteromesoscutum: mostly with deep, dense punctures (separated by less than 2.0 × its maximum diameter). Mesoscutellar disc: with punctures near margins, central part mostly smooth. Number of pits in scutoscutellar sulcus: 7 or 8. Maximum height of mesoscutellum lunules/maximum height of lateral face of mesoscutellum: 0.6–0.7. Propodeum areola: completely defined by carinae, including transverse carina extending to spiracle. Propodeum background sculpture: mostly sculptured. Mediotergite 1 length/width at posterior margin: 2.0–2.2. Mediotergite 1 shape: slightly widening from anterior margin to 0.7–0.8 mediotergite length (where maximum width is reached), then narrowing towards posterior margin. Mediotergite 1 sculpture: mostly sculptured, excavated area centrally with transverse striation inside and/or a polished knob centrally on posterior margin of mediotergite. Mediotergite 2 width at posterior margin/length: 3.2–3.5. Mediotergite 2 sculpture: mostly smooth. Outer margin of hypopygium: with a wide, medially folded, transparent, semi–desclerotized area; usually with 4 or more pleats. Ovipositor thickness: about same width throughout its length. Ovipositor sheaths length/metatibial length: 1.6–1.7. Length of fore wing veins r/2RS: 2.0–2.2. Length of fore wing veins 2RS/2M: 1.1–1.3. Length of fore wing veins 2M/(RS+M)b: 0.5–0.6. Pterostigma length/width: 3.1–3.5. Point of insertion of vein r in pterostigma: clearly beyond half way point length of pterostigma. Angle of vein r with fore wing anterior margin: clearly outwards, inclined towards fore wing apex. Shape of junction of veins r and 2RS in fore wing: distinctly but not strongly angled.

**Male.** As in female.

#### Molecular data.

Sequences in BOLD: 13, barcode compliant sequences: 12.

#### Biology/ecology.

Solitary. Host: Elachistidae, *Antaeotricha* Janzen109, *Stenoma* BioLep86, elachBioLep01 BioLep754DHJ01, elachBioLep01 BioLep754, elachJanzen01 Janzen211.

#### Distribution.

Costa Rica, ACG.

#### Etymology.

We dedicate this species to Lauren Morales for her diligent efforts in ACG Planificacion.

### Apanteles leninguadamuzi

Fernández-Triana sp. n.

http://zoobank.org/32822E91-4526-40BB-BB4F-4F685938C7EE

http://species-id.net/wiki/Apanteles_leninguadamuzi

Figs 14 , 216

Apanteles Rodriguez47. Smith et al. (2008). Interim name provided by the authors.

#### Type locality.

COSTA RICA, Guanacaste, ACG, Sector Cacao, Cuesta Caimito, 640m, 10.89080, -85.47192.

#### Holotype.

♀ in CNC. Specimen labels: 1. Costa Rica: Guanacaste, ACG, Sector Cacao, Cuesta Caimito, 10.vii.2004, 640m, 10.89080, -85.47192, 04-SRNP-47126.

#### Paratypes.

41 ♀, 12 ♂ (BMNH, CNC, INBIO, INHS, NMNH). COSTA RICA, ACG database codes: DHJPAR0002208, DHJPAR0002216, DHJPAR0020106, 04-SRNP-45135, 04-SRNP-47126.

#### Description.

**Female.** Body color: body mostly dark except for some sternites which may be pale. Antenna color: scape, pedicel, and flagellum dark. Coxae color (pro-, meso-, metacoxa): dark, dark, dark. Femora color (pro-, meso-, metafemur): anteriorly dark/posteriorly pale, dark, dark. Tibiae color (pro-, meso-, metatibia): pale, pale, mostly dark but anterior 0.2 or less pale. Tegula and humeral complex color: tegula pale, humeral complex half pale/half dark. Pterostigma color: mostly pale and/or transparent, with thin dark borders. Fore wing veins color: partially pigmented (a few veins may be dark but most are pale). Antenna length/body length: antenna about as long as body (head to apex of metasoma); if slightly shorter, at least extending beyond anterior 0.7 metasoma length or antenna shorter than body (head to apex of metasoma), not extending beyond anterior 0.7 metasoma length. Body in lateral view: not distinctly flattened dorso–ventrally. Body length (head to apex of metasoma): 2.5–2.6 mm, 2.7–2.8 mm or 2.9–3.0 mm. Fore wing length: 2.7–2.8 mm or 2.9–3.0 mm. Ocular–ocellar line/posterior ocellus diameter: 2.3–2.5. Interocellar distance/posterior ocellus diameter: 1.7–1.9. Antennal flagellomerus 2 length/width: 2.3–2.5 or 2.6–2.8. Antennal flagellomerus 14 length/width: 1.4–1.6, rarely 1.1–1.3. Length of flagellomerus 2/length of flagellomerus 14: 2.0–2.2, rarely 2.3–2.5. Tarsal claws: with single basal spine–like seta. Metafemur length/width: 2.8–2.9, rarely 3.0–3.1. Metatibia inner spur length/metabasitarsus length: 0.4–0.5. Anteromesoscutum: mostly with deep, dense punctures (separated by less than 2.0 × its maximum diameter). Mesoscutellar disc: mostly punctured. Number of pits in scutoscutellar sulcus: 7 or 8. Maximum height of mesoscutellum lunules/maximum height of lateral face of mesoscutellum: 0.4–0.5. Propodeum areola: completely defined by carinae, including transverse carina extending to spiracle. Propodeum background sculpture: mostly sculptured. Mediotergite 1 length/width at posterior margin: 2.0–2.2 or 2.3–2.5. Mediotergite 1 shape: slightly widening from anterior margin to 0.7–0.8 mediotergite length (where maximum width is reached), then narrowing towards posterior margin. Mediotergite 1 sculpture: mostly sculptured, excavated area centrally with transverse striation inside and/or a polished knob centrally on posterior margin of mediotergite. Mediotergite 2 width at posterior margin/length: 3.2–3.5. Mediotergite 2 sculpture: with some sculpture, mostly near posterior margin. Outer margin of hypopygium: with a wide, medially folded, transparent, semi–desclerotized area; usually with 4 or more pleats. Ovipositor thickness: about same width throughout its length. Ovipositor sheaths length/metatibial length: 1.2–1.3 or 1.4–1.5. Length of fore wing veins r/2RS: 1.7–1.9. Length of fore wing veins 2RS/2M: 1.4–1.6. Length of fore wing veins 2M/(RS+M)b: 0.5–0.6. Pterostigma length/width: 3.1–3.5. Point of insertion of vein r in pterostigma: clearly beyond half way point length of pterostigma. Angle of vein r with fore wing anterior margin: clearly outwards, inclined towards fore wing apex. Shape of junction of veins r and 2RS in fore wing: distinctly but not strongly angled.

**Male.** As female but with darker tibiae.

#### Molecular data.

Sequences in BOLD: 39, barcode compliant sequences: 39.

#### Biology/ecology.

Gregarious (Fig. 216). Host: Elachistidae, *Stenoma byssina*.

#### Distribution.

Costa Rica, ACG.

#### Etymology.

We dedicate this species to Lenin Guadamuz in recognition of his diligent efforts for the ACG Programa de Seguridad.

### Apanteles leonelgarayi

Fernández-Triana sp. n.

http://zoobank.org/5E309A2B-9257-4AF2-8493-659C427E93B3

http://species-id.net/wiki/Apanteles_leonelgarayi

Figs 138 , 294

#### Type locality.

COSTA RICA, Alajuela, ACG, Sector Pitilla, Quebradona, 475m, 10.99102, -85.39539.

#### Holotype.

♀ in CNC. Specimen labels: 1. DHJPAR0040434. 2. COSTA RICA, Alajuela, ACG, Sector Pitilla, Quebradona, 27.vii.2010, 10.99102°N, -85.39539°W, 475m, DHJPAR0040434. 3. Voucher: D.H.Janzen & W.Hallwachs, DB: http://janzen.sas.upenn.edu, Area de Conservación Guanacaste, COSTA RICA, 10-SRNP-72417.

#### Paratypes.

29 ♀, 12 ♂ (BMNH, CNC, INBIO, INHS, NMNH). COSTA RICA: Guanacaste, ACG database code: DHJPAR0042944, 10-SRNP-72417, 10-SRNP-73384.

#### Description.

**Female.** Body color: head dark, mesosoma dark with parts of axillar complex pale, metasoma with some mediotergites, most laterotergites, sternites, and/or hypopygium pale. Antenna color: scape and/or pedicel pale, flagellum dark. Coxae color (pro-, meso-, metacoxa): pale, pale, pale. Femora color (pro-, meso-, metafemur): pale, pale, pale. Tibiae color (pro-, meso-, metatibia): pale, pale, anteriorly pale/posteriorly dark. Tegula and humeral complex color: both pale. Pterostigma color: dark with pale spot at base. Fore wing veins color: mostly dark (a few veins may be unpigmented). Antenna length/body length: antenna about as long as body (head to apex of metasoma); if slightly shorter, at least extending beyond anterior 0.7 metasoma length. Body in lateral view: not distinctly flattened dorso–ventrally. Body length (head to apex of metasoma): 2.3–2.4 mm. Fore wing length: 2.3–2.4 mm. Ocular–ocellar line/posterior ocellus diameter: 1.7–1.9. Interocellar distance/posterior ocellus diameter: 1.4–1.6. Antennal flagellomerus 2 length/width: 2.9–3.1. Antennal flagellomerus 14 length/width: 1.1–1.3. Length of flagellomerus 2/length of flagellomerus 14: 2.3–2.5. Tarsal claws: simple. Metafemur length/width: 3.0–3.1. Metatibia inner spur length/metabasitarsus length: 0.4–0.5. Anteromesoscutum: mostly smooth or with shallow sparse punctures, except for anterior 0.3 where it has deeper and/or denser punctures. Mesoscutellar disc: mostly smooth. Number of pits in scutoscutellar sulcus: 7 or 8 or 9 or 10. Maximum height of mesoscutellum lunules/maximum height of lateral face of mesoscutellum: 0.4–0.5. Propodeum areola: completely defined by carinae, including transverse carina extending to spiracle. Propodeum background sculpture: partly sculptured, especially on anterior 0.5. Mediotergite 1 length/width at posterior margin: 2.0–2.2. Mediotergite 1 shape: more or less parallel–sided. Mediotergite 1 sculpture: with some sculpture near lateral margins and/or posterior 0.2–0.4 of mediotergite. Mediotergite 2 width at posterior margin/length: 3.2–3.5. Mediotergite 2 sculpture: mostly smooth. Outer margin of hypopygium: with a medially folded, transparent, semi–desclerotized area; with 0–3 pleats visible. Ovipositor thickness: anterior width at most 2.0 × posterior width (beyond ovipositor constriction). Ovipositor sheaths length/metatibial length: 0.3 or less. Length of fore wing veins r/2RS: 1.0 or less. Length of fore wing veins 2RS/2M: 1.4–1.6. Length of fore wing veins 2M/(RS+M)b: 0.9–1.0. Pterostigma length/width: 2.6–3.0. Point of insertion of vein r in pterostigma: about half way point length of pterostigma. Angle of vein r with fore wing anterior margin: clearly outwards, inclined towards fore wing apex. Shape of junction of veins r and 2RS in fore wing: distinctly but not strongly angled.

**Male.** As in female but with a relatively shorter mediotergite 2.

#### Molecular data.

Sequences in BOLD: 6, barcode compliant sequences: 6.

#### Biology/ecology.

Gregarious (Fig. 294). Hosts: Elachistidae, elachJanzen01 Janzen835.

#### Distribution.

Costa Rica, ACG.

#### Comments.

This species is characterized by an extremely short ovipositor (0.3 × as long as metatibia), mediotergite 2 relatively large (its medial length almost as long as medial length of mediotergite 3), mediotergite 1 almost completely smooth, and relatively extensive yellow coloration. The first two characters are unique among Mesoamerican *Apanteles*, and suggest that the species may better be placed on a different genus when more studies on Microgastrinae phylogeny are done. It also has a tooth-like projection in the center of postscutellum, which so far seems unique among all Mesoamerican *Apanteles* as well. It is here described as *Apanteles* because that is the best arrangement at present.

#### Etymology.

We dedicate this species to Leonel Garay in recognition of his diligent efforts for the ACG Programa de Contabilidad.

### Apanteles leucopus

(Ashmead, 1900)

http://species-id.net/wiki/Apanteles_leucopus

Fig. 78

Urogaster leucopus Ashmead, 1900: 287. Transferred by Szépligeti (1904: 110).

#### Type locality.

GRENADA, Balthazar.

#### Holotype.

♀, BMNH (examined photos of the holotype).

#### Molecular data.

No molecular data available for this species.

#### Biology/ecology.

Unknonwn.

#### Distribution.

Grenada, St. Vincent.

#### Comments.

We could only see three photos of the holotype, which were not enough to provide a description of the species. For the same reason, we could not include the species in the above key to the *ater* group. We have no reason to suspect that this species occurs in ACG.

### Apanteles leucostigmus

(Ashmead, 1900)

http://species-id.net/wiki/Apanteles_leucostigmus

Fig. 188

Urogaster leucostigmus Ashmead, 1900: 289.
Apanteles leucostigmus (Ashmead). Transferred by Szépligeti (1904: 110).
Apanteles leucostigmus (Ashmead). Misidentification of the species by Smith et al. (2008).

#### Type locality.

ST. VINCENT, Lesser Antilles.

#### Holotype.

♀, BMNH (examined).

#### Material examined.

1 ♀, United States: Florida, Belle Glade; v.1941; D. J. Taylor coll.; ex: *Urbanus proteus* (CNC); 3 ♀, St Vincent Is. (CNC).

#### Description.

**Female.** Body color: body mostly dark except for some sternites which may be pale. Antenna color: scape, pedicel, and flagellum dark (?). Coxae color (pro-, meso-, metacoxa): dark, dark, dark. Femora color (pro-, meso-, metafemur): pale, dark, dark. Tibiae color (pro-, meso-, metatibia): pale, pale, anteriorly pale/posteriorly dark. Tegula and humeral complex color: both pale. Pterostigma color: mostly pale and/or transparent, with thin dark borders. Fore wing veins color: mostly white or entirely transparent. Antenna length/body length: antenna about as long as body (head to apex of metasoma); if slightly shorter, at least extending beyond anterior 0.7 metasoma length (?). Body in lateral view: not distinctly flattened dorso–ventrally. Body length (head to apex of metasoma): 2.1–2.2 mm. Fore wing length: 2.3–2.4 mm. Ocular–ocellar line/posterior ocellus diameter: 2.3–2.5. Interocellar distance/posterior ocellus diameter: 2.0–2.2. Tarsal claws: simple (?). Metafemur length/width: 2.8–2.9. Anteromesoscutum: mostly with deep, dense punctures (separated by less than 2.0 × its maximum diameter). Mesoscutellar disc: mostly smooth. Number of pits in scutoscutellar sulcus: 9 or 10. Maximum height of mesoscutellum lunules/maximum height of lateral face of mesoscutellum: 0.8 or more. Propodeum areola: completely defined by carinae, including transverse carina extending to spiracle. Propodeum background sculpture: partly sculptured, especially on anterior 0.5. Mediotergite 1 length/width at posterior margin: 1.7–1.9. Mediotergite 1 shape: slightly widening from anterior margin to 0.7–0.8 mediotergite length (where maximum width is reached), then narrowing towards posterior margin. Mediotergite 1 sculpture: with some sculpture near lateral margins and/or posterior 0.2–0.4 of mediotergite. Mediotergite 2 width at posterior margin/length: 4.0–4.3. Mediotergite 2 sculpture: mostly smooth. Outer margin of hypopygium: with a wide, medially folded, transparent, semi–desclerotized area; usually with 4 or more pleats (?). Ovipositor thickness: anterior width 3.0–5.0 × posterior width (beyond ovipositor constriction) (?). Ovipositor sheaths length/metatibial length: 0.6–0.7. Length of fore wing veins r/2RS: 1.7–1.9. Length of fore wing veins 2RS/2M: 1.1–1.3. Length of fore wing veins 2M/(RS+M)b: 0.7–0.8. Pterostigma length/width: 3.1–3.5. Point of insertion of vein r in pterostigma: about half way point length of pterostigma. Angle of vein r with fore wing anterior margin: clearly inwards, inclined towards fore wing base. Shape of junction of veins r and 2RS in fore wing: distinctly but not strongly angled.

#### Molecular data.

No molecular data available for this species.

#### Biology/ecology.

Gregarious. Hosts: Hesperiidae, *Urbanus proteus* (of specimens identified as this species in United States, FL).

#### Distribution.

Cuba, Grenada, Puerto Rico, St. Vincent, United States (FL). There is no suggestion that this species occurs in ACG or Costa Rica.

#### Comments.

The holotype is missing the antennae, one forewing and some legs – but it is possible to see a full set of legs, except for the sole hind leg remaining where some tarsal segments are missing. The name *Apanteles leucostigmus* was applied by Smith et al. (2008) to a complex of around 40 species reared from hesperiids in ACG. At that time it was thought that one of those species might correspond to the actual *Apanteles leucostigmus*. After examining the holotype of *Apanteles leucostigmus*, it is clear, however, that none of the ACG species correspond to it. However, all are related and belong to the same species-group. Thus, the name of *Apanteles leucostigmus*, as used as the base for an interim name in Smith et al. (2008), should be considered as a misidentification, as well as was the case when applied to members of this group before it was realized that it is a speciose group composed of morphologically similar species. We examined a specimen from Peru, deposited in the CNC and labelled as “*Apanteles leucostigmus*”, and believe is not that species either, but just another member of the *leucostigmus* group.

### Apanteles lilliammenae

Fernández-Triana sp. n.

http://zoobank.org/E479CB5B-6058-47F3-9CE0-305E423B7C55

http://species-id.net/wiki/Apanteles_lilliammenae

Figs 189 , 318

Apanteles Rodriguez64 (Smith et al. 2006). Interim name provided by the authors.

#### Type locality.

COSTA RICA, Guanacaste, ACG, Sector Horizontes, Vado Esteron, 95m, 10.76271, -85.56004.

#### Holotype.

♀ in CNC. Specimen labels: 1. DHJPAR0001675. 2. COSTA RICA, Guanacaste, ACG, Sector Horizontes, Vado Esteron, 03.ix.1996, 95m, 10.76271, -85.56004, DHJPAR0001675.<<Hmm, got a problem here. Your holotype is a 375 bp specimen that treed in Apanteles Rodriguez23 but by host, clearly does not belong there; it may well be R64 and happy to have it there, but a complete barcode is the only way I have of moving it from 23 to 64, unless you have some distinguising morphological trait. None of those short sequence specimens from the wrong host in Apanteles Rodriguez23 should be used for paratatypes or holotypes, so please select one from the large group of R64 that gave really good barcodes. I am doing my best to avoid this kind of perturbation to the paper, but here we really do need to do it (I also note that this DHJPAR was also placed in the paratype series, and best not to put it there either).

#### Paratypes.

44 ♀, 19 ♂ (BMNH, CNC, INBIO, INHS, NMNH). COSTA RICA, ACG database codes: DHJPAR0002713, DHJPAR0004637, DHJPAR0005241, DHJPAR0005250, DHJPAR0011963, 96-SRNP-9935.

#### Description.

**Female.** Metatibia color (outer face): with extended pale coloration (light yellow to orange–yellow), ranging from 0.4 to almost entire metatibia length. Fore wing veins color: veins C+Sc+R and R1 mostly brown; usually veins r, 2RS, 2M, (RS+M)b, 1CU, 2Cua, and 1m–cu partially brown; interior area of other veins, and at least part of pterostigma, usually light brown or yellowish–white. Antenna length/body length: antenna about as long as body (head to apex of metasoma); if slightly shorter, at least extending beyond anterior 0.7 metasoma length. Body length (head to apex of metasoma): 2.3–2.4 mm or 2.5–2.6 mm. Fore wing length: 2.5–2.6 mm. Metafemur length/width: 3.0–3.1. Mediotergite 1 length/width at posterior margin: 2.7–2.8. Mediotergite 1 maximum width/width at posterior margin: 1.4–1.5. Ovipositor sheaths length/metafemur length: 1.0. Ovipositor sheaths length/metatibia length: 0.8.

#### Molecular data.

Sequences in BOLD: 19, barcode compliant sequences: 19.

#### Biology/ecology.

Gregarious (Fig. 318). Host: Hesperiidae, *Urbanus doryssus* DHJ02.

#### Distribution.

Costa Rica, ACG.

#### Comments.

A few females (2-3 out of 45 studied) have the metatibia with yellow color 0.3 × or so (i.e., not like the other, +93% of the specimens which have the yellow area extending to at least 0.4 × metatibia length, usually up to 0.5 ×).

#### Etymology.

We dedicate this species to Lilliam Mena in recognition of her diligent efforts for the administration of INBio, Costa Rica’s Instituto Nacional de Biodiversidad and support of the INBio directorate.

### Apanteles lisabearssae

Fernández-Triana sp. n.

http://zoobank.org/8F46EB36-2C49-4D74-8BEB-ED314C7271A1

http://species-id.net/wiki/Apanteles_lisabearssae

Fig. 108

#### Type locality.

COSTA RICA, Guanacaste, ACG, Sector El Hacha, Sendero Bejuquilla, 280m, 11.03004, -85.52699.

#### Holotype.

♀ in CNC. Specimen labels: 1. DHJPAR0012531. 2. 77-3May99-LASB.

#### Paratypes.

7 ♀, 3 ♂ (CNC, NMNH). COSTA RICA, ACG database codes: DHJPAR0013065, DHJPAR0013201, DHJPAR0013568.

#### Description.

**Female.** Body color: body mostly dark except for some sternites which may be pale. Antenna color: scape, pedicel, and flagellum dark. Coxae color (pro-, meso-, metacoxa): pale, pale, dark. Femora color (pro-, meso-, metafemur): pale, pale, dark. Tibiae color (pro-, meso-, metatibia): pale, pale, anteriorly pale/posteriorly dark. Tegula and humeral complex color: both pale. Pterostigma color: mostly pale and/or transparent, with thin dark borders. Fore wing veins color: mostly dark (a few veins may be unpigmented). Antenna length/body length: antenna about as long as body (head to apex of metasoma); if slightly shorter, at least extending beyond anterior 0.7 metasoma length. Body in lateral view: not distinctly flattened dorso–ventrally. Body length (head to apex of metasoma): 2.1–2.2 mm. Fore wing length: 2.0 mm or less. Ocular–ocellar line/posterior ocellus diameter: 2.3–2.5. Interocellar distance/posterior ocellus diameter: 2.0–2.2. Antennal flagellomerus 2 length/width: 2.6–2.8. Antennal flagellomerus 14 length/width: 1.1–1.3. Length of flagellomerus 2/length of flagellomerus 14: 2.0–2.2. Tarsal claws: simple. Metafemur length/width: 3.2–3.3. Metatibia inner spur length/metabasitarsus length: 0.4–0.5. Anteromesoscutum: mostly with deep, dense punctures (separated by less than 2.0 × its maximum diameter). Mesoscutellar disc: mostly smooth. Number of pits in scutoscutellar sulcus: 11 or 12. Maximum height of mesoscutellum lunules/maximum height of lateral face of mesoscutellum: 0.4–0.5. Propodeum areola: completely defined by carinae, but only partial or absent transverse carina. Propodeum background sculpture: mostly sculptured. Mediotergite 1 length/width at posterior margin: 3.8–4.0. Mediotergite 1 shape: clearly narrowing towards posterior margin. Mediotergite 1 sculpture: mostly sculptured, excavated area centrally with transverse striation inside and/or a polished knob centrally on posterior margin of mediotergite. Mediotergite 2 width at posterior margin/length: 2.8–3.1. Mediotergite 2 sculpture: with some sculpture, mostly near posterior margin. Outer margin of hypopygium: with a wide, medially folded, transparent, semi–desclerotized area; usually with 4 or more pleats. Ovipositor thickness: about same width throughout its length. Ovipositor sheaths length/metatibial length: 1.2–1.3. Length of fore wing veins r/2RS: 1.4–1.6. Length of fore wing veins 2RS/2M: 1.4–1.6. Length of fore wing veins 2M/(RS+M)b: 0.7–0.8. Pterostigma length/width: 2.6–3.0. Point of insertion of vein r in pterostigma: about half way point length of pterostigma. Angle of vein r with fore wing anterior margin: clearly outwards, inclined towards fore wing apex. Shape of junction of veins r and 2RS in fore wing: distinctly but not strongly angled.

**Male.** As in female.

#### Molecular data.

Sequences in BOLD: 46, barcode compliant sequences: 42.

#### Biology/ecology.

Malaise-trapped.

#### Distribution.

Costa Rica, AGC.

#### Etymology.

The senior author dedicates this species to Lisa Bearss (CNC, Ottawa), as an appreciation for her support, and for kindly sharing her knowledge on the Keyence photographic equipment.

### Apanteles luciariosae

Fernández-Triana sp. n.

http://zoobank.org/D0BDDCFC-C5C9-4DD5-9E60-75945F14BA53

http://species-id.net/wiki/Apanteles_luciariosae

Figs 60 , 253

Apanteles Rodriguez124 (Smith et al. 2006). Interim name provided by the authors.

#### Type locality.

COSTA RICA, Guanacaste, ACG, Sector Pitilla, Pasmompa, 440m, 11.01926, -85.40997.

#### Holotype.

♀ in CNC. Specimen labels: 1. DHJPAR0004978. 2. COSTA RICA, Guanacaste, ACG, Sector Pitilla, Pasmompa, 10.v.2006, 440m, 11.01926, -85.40997, 06-SRNP-31784.

#### Paratypes.

1 ♀, 5 ♂ (CNC). COSTA RICA, ACG database codes: DHJPAR0002249, DHJPAR0002315, DHJPAR0002327, DHJPAR0002334, DHJPAR0005001, DHJPAR0030958.

#### Description.

**Female.** Body color: body mostly dark except for some sternites which may be pale. Antenna color: scape, pedicel, and flagellum dark. Coxae color (pro-, meso-, metacoxa): dark, dark, dark. Femora color (pro-, meso-, metafemur): pale, pale, pale. Tibiae color (pro-, meso-, metatibia): pale, pale, pale. Tegula and humeral complex color: both pale. Pterostigma color: mostly pale and/or transparent, with thin dark borders or entirely pale or transparent, translucent. Fore wing veins color: partially pigmented (a few veins may be dark but most are pale). Antenna length/body length: antenna about as long as body (head to apex of metasoma); if slightly shorter, at least extending beyond anterior 0.7 metasoma length. Body in lateral view: not distinctly flattened dorso–ventrally. Body length (head to apex of metasoma): 2.9–3.0 mm or 3.1–3.2 mm. Fore wing length: 3.3–3.4 mm or 3.5–3.6 mm. Ocular–ocellar line/posterior ocellus diameter: 2.0–2.2. Interocellar distance/posterior ocellus diameter: 1.7–1.9. Antennal flagellomerus 2 length/width: 2.0–2.2. Antennal flagellomerus 14 length/width: 1.4–1.6. Length of flagellomerus 2/length of flagellomerus 14: 1.7–1.9. Tarsal claws: with single basal spine–like seta. Metafemur length/width: 3.0–3.1. Metatibia inner spur length/metabasitarsus length: 0.6–0.7. Anteromesoscutum: mostly with deep, dense punctures (separated by less than 2.0 × its maximum diameter). Mesoscutellar disc: with punctures near margins, central part mostly smooth. Number of pits in scutoscutellar sulcus: 9 or 10. Maximum height of mesoscutellum lunules/maximum height of lateral face of mesoscutellum: 0.6–0.7. Propodeum areola: completely defined by carinae, including transverse carina extending to spiracle. Propodeum background sculpture: mostly sculptured. Mediotergite 1 length/width at posterior margin: 3.2–3.4 or 3.5–3.7. Mediotergite 1 shape: mostly parallel–sided for 0.5–0.7 of its length, then narrowing posteriorly so mediotergite anterior width >1.1 × posterior width. Mediotergite 1 sculpture: with some sculpture near lateral margins and/or posterior 0.2–0.4 of mediotergite. Mediotergite 2 width at posterior margin/length: 3.6–3.9. Mediotergite 2 sculpture: mostly smooth. Outer margin of hypopygium: inflexible (without a folded, transparent, semi–desclerotized area); with no pleats visible. Ovipositor thickness: anterior width 3.0–5.0 × posterior width (beyond ovipositor constriction). Ovipositor sheaths length/metatibial length: 0.4–0.5. Length of fore wing veins r/2RS: 2.3 or more. Length of fore wing veins 2RS/2M: 1.1–1.3. Length of fore wing veins 2M/(RS+M)b: 0.5–0.6. Pterostigma length/width: 2.6–3.0. Point of insertion of vein r in pterostigma: clearly beyond half way point length of pterostigma. Angle of vein r with fore wing anterior margin: clearly outwards, inclined towards fore wing apex. Shape of junction of veins r and 2RS in fore wing: distinctly but not strongly angled.

**Male.** Similar to female, except for darker legs.

#### Molecular data.

Sequences in BOLD: 11, barcode compliant sequences: 11. Specimen with code DHJPAR0002305 has a barcode that differs from the others by 1.3%, but we consider it to be the same species, given its identical morphology and host record.

#### Biology/ecology.

Solitary (Fig. 253). Host: Hesperiidae, *Gorgythion begga pyralina*.

#### Distribution.

Costa Rica, ACG.

#### Comments.

See comments for *Apanteles freddyquesadai*.

#### Etymology.

We dedicate this species to Lucía Ríos recognition of her diligent efforts for the ACG Programa de Parataxónomos and Estación Biológica Los Almendros of Sector El Hacha and Sector Del Oro.

### Apanteles luisbrizuelai

Fernández-Triana sp. n.

http://zoobank.org/75BB27A0-43DC-46B8-A8CD-A8D4DB37E70B

http://species-id.net/wiki/Apanteles_luisbrizuelai

Fig. 46

#### Type locality.

COSTA RICA, Guanacaste, ACG, Sector Rincon Rain Forest, Sendero Venado, 420m, 10.89678, -85.27001.

#### Holotype.

♀ in CNC. Specimen labels: 1. DHJPAR0042981. 2. COSTA RICA, Guanacaste, ACG, Rincon Rain Forest, Sendero Venado, 13.iii.2011, 10.89678 N, -85.27001 W, 420m, DHJPAR0042981. 3. Voucher: D.H.Janzen & W.Hallwachs, DB: http://janzen.sas.upenn.edu, Area de Conservación Guanacaste, COSTA RICA, 11-SRNP-41236.

#### Description.

**Female.** Body color: body mostly dark except for some sternites which may be pale. Antenna color: scape, pedicel, and flagellum dark. Coxae color (pro-, meso-, metacoxa): dark, dark, dark. Femora color (pro-, meso-, metafemur): anteriorly dark/posteriorly pale, dark, dark. Tibiae color (pro-, meso-, metatibia): pale, pale, anteriorly pale/posteriorly dark. Tegula and humeral complex color: tegula pale, humeral complex half pale/half dark. Pterostigma color: mostly pale and/or transparent, with thin dark borders. Fore wing veins color: partially pigmented (a few veins may be dark but most are pale). Antenna length/body length: antenna about as long as body (head to apex of metasoma); if slightly shorter, at least extending beyond anterior 0.7 metasoma length. Body in lateral view: not distinctly flattened dorso–ventrally. Body length (head to apex of metasoma): 3.3–3.4 mm. Fore wing length: 3.3–3.4 mm. Ocular–ocellar line/posterior ocellus diameter: 2.3–2.5. Interocellar distance/posterior ocellus diameter: 1.7–1.9. Antennal flagellomerus 2 length/width: 2.9–3.1. Antennal flagellomerus 14 length/width: 1.7–1.9. Length of flagellomerus 2/length of flagellomerus 14: 2.0–2.2. Tarsal claws: with single basal spine–like seta. Metafemur length/width: 3.0–3.1. Metatibia inner spur length/metabasitarsus length: 0.4–0.5. Anteromesoscutum: mostly with deep, dense punctures (separated by less than 2.0 × its maximum diameter). Mesoscutellar disc: mostly smooth. Number of pits in scutoscutellar sulcus: 7 or 8. Maximum height of mesoscutellum lunules/maximum height of lateral face of mesoscutellum: 0.6–0.7. Propodeum areola: completely defined by carinae, including transverse carina extending to spiracle. Propodeum background sculpture: mostly sculptured. Mediotergite 1 length/width at posterior margin: 2.6–2.8. Mediotergite 1 shape: mostly parallel–sided for 0.5–0.7 of its length, then narrowing posteriorly so mediotergite anterior width >1.1 × posterior width. Mediotergite 1 sculpture: mostly sculptured, excavated area centrally with transverse striation inside and/or a polished knob centrally on posterior margin of mediotergite. Mediotergite 2 width at posterior margin/length: 2.0–2.3. Mediotergite 2 sculpture: mostly smooth. Outer margin of hypopygium: with a wide, medially folded, transparent, semi–desclerotized area; usually with 4 or more pleats. Ovipositor thickness: about same width throughout its length. Ovipositor sheaths length/metatibial length: 1.4–1.5. Length of fore wing veins r/2RS: 2.0–2.2. Length of fore wing veins 2RS/2M: 1.1–1.3. Length of fore wing veins 2M/(RS+M)b: 0.7–0.8. Pterostigma length/width: 3.6 or more. Point of insertion of vein r in pterostigma: clearly beyond half way point length of pterostigma. Angle of vein r with fore wing anterior margin: clearly outwards, inclined towards fore wing apex. Shape of junction of veins r and 2RS in fore wing: distinctly but not strongly angled.

**Male.** Unknown.

#### Molecular data.

Sequences in BOLD: 6, barcode compliant sequences: 5.

#### Biology/ecology.

Solitary. Hosts: Elachistidae, *Antaeotricha* Janzen128.

#### Distribution.

Costa Rica, ACG.

#### Etymology.

We dedicate this species to Luis Brizuela in recognition of his diligent efforts for the ACG Programa de Seguridad.

### Apanteles luiscanalesi

Fernández-Triana sp. n.

http://zoobank.org/B12204D5-5AD4-4E42-B887-DCC694EB5F26

http://species-id.net/wiki/Apanteles_luiscanalesi

Figs 15 , 217

#### Type locality.

COSTA RICA, Alajuela, ACG, Sector Rincon Rain Forest, Estación Caribe, 415m, 10.90187, -85.27495.

#### Holotype.

♀ in CNC. Specimen labels: 1. DHJPAR0035393. 2. Voucher: D.H.Janzen & W.Hallwachs, DB: http://janzen.sas.upenn.edu, Area de Conservación Guanacaste, COSTA RICA, 09-SRNP-40853.

#### Paratypes.

7 ♀, 11 ♂ (BMNH, CNC, INBIO, INHS, NMNH). COSTA RICA, ACG database codes: DHJPAR0035393.

#### Description.

**Female.** Body color: body mostly dark except for some sternites which may be pale. Antenna color: scape, pedicel, and flagellum dark. Coxae color (pro-, meso-, metacoxa): dark, dark, dark. Femora color (pro-, meso-, metafemur): anteriorly dark/posteriorly pale, dark, dark. Tibiae color (pro-, meso-, metatibia): pale, pale, anteriorly pale/posteriorly dark. Tegula and humeral complex color: tegula pale, humeral complex half pale/half dark. Pterostigma color: mostly pale and/or transparent, with thin dark borders. Fore wing veins color: partially pigmented (a few veins may be dark but most are pale). Antenna length/body length: antenna about as long as body (head to apex of metasoma); if slightly shorter, at least extending beyond anterior 0.7 metasoma length. Body in lateral view: not distinctly flattened dorso–ventrally. Body length (head to apex of metasoma): 2.3–2.4 mm, rarely 2.5–2.6 mm. Fore wing length: 2.5–2.6 mm, rarely 2.3–2.4 mm. Ocular–ocellar line/posterior ocellus diameter: 2.3–2.5. Interocellar distance/posterior ocellus diameter: 2.0–2.2. Antennal flagellomerus 2 length/width: 2.9–3.1. Antennal flagellomerus 14 length/width: 1.4–1.6. Length of flagellomerus 2/length of flagellomerus 14: 2.0–2.2. Tarsal claws: with single basal spine–like seta. Metafemur length/width: 3.0–3.1. Metatibia inner spur length/metabasitarsus length: 0.4–0.5. Anteromesoscutum: mostly with deep, dense punctures (separated by less than 2.0 × its maximum diameter). Mesoscutellar disc: with punctures near margins, central part mostly smooth. Number of pits in scutoscutellar sulcus: 7 or 8. Maximum height of mesoscutellum lunules/maximum height of lateral face of mesoscutellum: 0.6–0.7. Propodeum areola: completely defined by carinae, including transverse carina extending to spiracle. Propodeum background sculpture: mostly sculptured. Mediotergite 1 length/width at posterior margin: 1.7–1.9. Mediotergite 1 shape: slightly widening from anterior margin to 0.7–0.8 mediotergite length (where maximum width is reached), then narrowing towards posterior margin. Mediotergite 1 sculpture: mostly sculptured, excavated area centrally with transverse striation inside and/or a polished knob centrally on posterior margin of mediotergite. Mediotergite 2 width at posterior margin/length: 3.6–3.9. Mediotergite 2 sculpture: mostly smooth. Outer margin of hypopygium: with a wide, medially folded, transparent, semi–desclerotized area; usually with 4 or more pleats. Ovipositor thickness: about same width throughout its length. Ovipositor sheaths length/metatibial length: 1.2–1.3. Length of fore wing veins r/2RS: 1.7–1.9. Length of fore wing veins 2RS/2M: 1.4–1.6. Length of fore wing veins 2M/(RS+M)b: 0.5–0.6. Pterostigma length/width: 3.6 or more. Point of insertion of vein r in pterostigma: clearly beyond half way point length of pterostigma. Angle of vein r with fore wing anterior margin: clearly outwards, inclined towards fore wing apex. Shape of junction of veins r and 2RS in fore wing: distinctly but not strongly angled.

**Male.** As in female but with darker legs and narrower mediotergite 1.

#### Molecular data.

Sequences in BOLD: 3, barcode compliant sequences: 3.

#### Biology/ecology.

Gregarious (Fig. 217). Host: Pyralidae, *Macalla niveorufa* DHJ02

#### Distribution.

Costa Rica, ACG.

#### Etymology.

We dedicate this species to Luis Canales in recognition of his diligent efforts for the ACG Sector Marino.

### Apanteles luiscantillanoi

Fernández-Triana sp. n.

http://zoobank.org/328C924C-D107-4737-8045-EDDAF16F48DB

http://species-id.net/wiki/Apanteles_luiscantillanoi

Figs 28 , 230

Apanteles Rodriguez100. Smith et al. (2008). Interim name provided by the authors.

#### Type locality.

COSTA RICA, Alajuela, ACG, Sector Pitilla, Sendero Nacho, 710m, 10.98445, -85.42481.

#### Holotype.

♀ in CNC. Specimen labels: 1. DHJPAR0038334. 2. COSTA RICA, Guanacaste, ACG, Sector Pitilla, Sendero Nacho, 27.i.2010, 10.98445°N, -85.42481°W, 710m, DHJPAR0038334. 3. Voucher: D.H.Janzen & W.Hallwachs, DB: http://janzen.sas.upenn.edu, Area de Conservación Guanacaste, COSTA RICA, 10-SRNP-30404.

#### Paratypes.

2 ♀ (CNC, NMNH). COSTA RICA, ACG database codes: DHJPAR0038362, DHJPAR0038363.

#### Description.

**Female.** Body color: body mostly dark except for some sternites which may be pale. Antenna color: scape, pedicel, and flagellum dark. Coxae color (pro-, meso-, metacoxa): dark, dark, dark. Femora color (pro-, meso-, metafemur): anteriorly dark/posteriorly pale, dark, dark. Tibiae color (pro-, meso-, metatibia): pale, anteriorly pale/posteriorly dark, anteriorly pale/posteriorly dark. Tegula and humeral complex color: both dark. Pterostigma color: mostly pale and/or transparent, with thin dark borders. Fore wing veins color: partially pigmented (a few veins may be dark but most are pale). Antenna length/body length: antenna about as long as body (head to apex of metasoma); if slightly shorter, at least extending beyond anterior 0.7 metasoma length. Body in lateral view: not distinctly flattened dorso–ventrally. Body length (head to apex of metasoma): 2.3–2.4 mm, rarely 2.1–2.2 mm or 2.5–2.6 mm. Fore wing length: 2.3–2.4 mm or 2.5–2.6 mm. Ocular–ocellar line/posterior ocellus diameter: 2.0–2.2. Interocellar distance/posterior ocellus diameter: 1.7–1.9. Antennal flagellomerus 2 length/width: 2.9–3.1. Antennal flagellomerus 14 length/width: 1.4–1.6. Length of flagellomerus 2/length of flagellomerus 14: 2.3–2.5. Tarsal claws: simple. Metafemur length/width: 2.8–2.9. Metatibia inner spur length/metabasitarsus length: 0.4–0.5. Anteromesoscutum: mostly with deep, dense punctures (separated by less than 2.0 × its maximum diameter). Mesoscutellar disc: mostly smooth. Number of pits in scutoscutellar sulcus: 9 or 10. Maximum height of mesoscutellum lunules/maximum height of lateral face of mesoscutellum: 0.6–0.7. Propodeum areola: completely defined by carinae, including transverse carina extending to spiracle. Propodeum background sculpture: partly sculptured, especially on anterior 0.5. Mediotergite 1 length/width at posterior margin: 2.0–2.2. Mediotergite 1 shape: more or less parallel–sided. Mediotergite 1 sculpture: mostly sculptured, excavated area centrally with transverse striation inside and/or a polished knob centrally on posterior margin of mediotergite. Mediotergite 2 width at posterior margin/length: 3.6–3.9 or 4.0–4.3. Mediotergite 2 sculpture: mostly smooth. Outer margin of hypopygium: with a wide, medially folded, transparent, semi–desclerotized area; usually with 4 or more pleats. Ovipositor thickness: about same width throughout its length. Ovipositor sheaths length/metatibial length: 1.0–1.1, rarely 0.8–0.9. Length of fore wing veins r/2RS: 1.4–1.6. Length of fore wing veins 2RS/2M: 1.1–1.3. Length of fore wing veins 2M/(RS+M)b: 0.7–0.8. Pterostigma length/width: 3.6 or more. Point of insertion of vein r in pterostigma: clearly beyond half way point length of pterostigma. Angle of vein r with fore wing anterior margin: clearly outwards, inclined towards fore wing apex. Shape of junction of veins r and 2RS in fore wing: distinctly but not strongly angled.

**Male.** Unknown.

#### Molecular data.

Sequences in BOLD: 11, barcode compliant sequences: 11.

#### Biology/ecology.

Solitary (Fig. 230). Hosts: Crambidae, *Diacme* BioLep02, *Undulambia* Solis02, *Neurophyseta completalis*.

#### Distribution.

Costa Rica, ACG.

#### Etymology.

We dedicate this species to Luis Cantillo in recognition of his diligent efforts for the ACG Programa de Protección e Incendios.

### Apanteles luisgarciai

Fernández-Triana sp. n.

http://zoobank.org/DF628AD6-83A4-4191-B43B-F8EF2E039ED3

http://species-id.net/wiki/Apanteles_luisgarciai

Figs 47 , 240

#### Type locality.

COSTA RICA, Guanacaste, ACG, Sector San Cristobal, Tajo Angeles, 540m, 10.86472, -85.41531.

#### Holotype.

♀ in CNC. Specimen labels: 1. DHJPAR0038250. 2. Voucher: D.H.Janzen & W.Hallwachs, DB: http://janzen.sas.upenn.edu, Area de Conservación Guanacaste, COSTA RICA, 09-SRNP-5512.

#### Description.

**Female.** Body color: body mostly dark except for some sternites which may be pale. Antenna color: scape, pedicel, and flagellum dark. Coxae color (pro-, meso-, metacoxa): dark, dark, dark. Femora color (pro-, meso-, metafemur): anteriorly dark/posteriorly pale, dark, dark. Tibiae color (pro-, meso-, metatibia): pale, pale, anteriorly pale/posteriorly dark. Tegula and humeral complex color: tegula pale, humeral complex half pale/half dark. Pterostigma color: mostly pale and/or transparent, with thin dark borders. Fore wing veins color: partially pigmented (a few veins may be dark but most are pale). Antenna length/body length: antenna about as long as body (head to apex of metasoma); if slightly shorter, at least extending beyond anterior 0.7 metasoma length. Body in lateral view: not distinctly flattened dorso–ventrally. Body length (head to apex of metasoma): 2.9–3.0 mm. Fore wing length: 2.9–3.0 mm. Ocular–ocellar line/posterior ocellus diameter: 2.6 or more. Interocellar distance/posterior ocellus diameter: 1.7–1.9. Antennal flagellomerus 2 length/width: 2.9–3.1. Antennal flagellomerus 14 length/width: 1.7–1.9. Length of flagellomerus 2/length of flagellomerus 14: 2.0–2.2. Tarsal claws: with single basal spine–like seta (?). Metafemur length/width: 3.2–3.3. Metatibia inner spur length/metabasitarsus length: 0.4–0.5. Anteromesoscutum: mostly with deep, dense punctures (separated by less than 2.0 × its maximum diameter). Mesoscutellar disc: mostly smooth. Number of pits in scutoscutellar sulcus: 9 or 10. Maximum height of mesoscutellum lunules/maximum height of lateral face of mesoscutellum: 0.6–0.7. Propodeum areola: completely defined by carinae, including transverse carina extending to spiracle. Propodeum background sculpture: partly sculptured, especially on anterior 0.5. Mediotergite 1 length/width at posterior margin: 2.6–2.8. Mediotergite 1 shape: mostly parallel–sided for 0.5–0.7 of its length, then narrowing posteriorly so mediotergite anterior width >1.1 × posterior width. Mediotergite 1 sculpture: mostly sculptured, excavated area centrally with transverse striation inside and/or a polished knob centrally on posterior margin of mediotergite. Mediotergite 2 width at posterior margin/length: 1.6–1.9. Mediotergite 2 sculpture: mostly smooth. Outer margin of hypopygium: with a wide, medially folded, transparent, semi–desclerotized area; usually with 4 or more pleats. Ovipositor thickness: about same width throughout its length. Ovipositor sheaths length/metatibial length: 1.6–1.7. Length of fore wing veins r/2RS: 1.4–1.6. Length of fore wing veins 2RS/2M: 1.7–1.8. Length of fore wing veins 2M/(RS+M)b: 0.7–0.8. Pterostigma length/width: 3.6 or more. Point of insertion of vein r in pterostigma: clearly beyond half way point length of pterostigma. Angle of vein r with fore wing anterior margin: more or less perpendicular to fore wing margin. Shape of junction of veins r and 2RS in fore wing: distinctly but not strongly angled.

**Male.** Unknown.

#### Molecular data.

Sequences in BOLD: 6, barcode compliant sequences: 6.

#### Biology/ecology.

Solitary (Fig. 240). Hosts: Gelechiidae, *Anacampsis* Janzen301.

#### Distribution.

Costa Rica, ACG.

#### Etymology.

We dedicate this species to Luis García in recognition of his diligent efforts for the ACG Programa de Ecoturismo.

### Apanteles luisgaritai

Fernández-Triana sp. n.

http://zoobank.org/6BF0CA6A-60EE-4069-9EB9-1FAD93C22022

http://species-id.net/wiki/Apanteles_luisgaritai

Figs 139 , 295

Apanteles Rodriguez82 (Smith et al. 2006). Interim name provided by the authors.

#### Type locality.

COSTA RICA, Alajuela, ACG, Sector San Cristobal, Rio Blanco Abajo, 500m, 10.90037, -85.37254.

#### Holotype.

♀ in CNC. Specimen labels: 1. COSTA RICA, Alajuela, ACG, Sector San Cristobal, Rio Blanco Abajo, 18.vi.2007, 500m, 10.90037, -85.37254, DHJPAR0020137.

#### Paratypes.

5 ♀, 2 ♂ (CNC, NMNH). COSTA RICA: ACG database codes: DHJPAR0005163, DHJPAR0039012, DHJPAR0039035, DHJPAR0039739, DHJPAR0039742, DHJPAR0039749, DHJPAR0039770.

#### Description.

**Female.** Body color: head pale, mesosoma extensively pale (anteromesoscutum and scutellar disc). Antenna color: scape, pedicel, and flagellum dark. Coxae color (pro-, meso-, metacoxa): pale, pale, partially pale/partially dark. Femora color (pro-, meso-, metafemur): mostly dark but anterior 0.2 pale, mostly dark but anterior 0.2 pale, anteriorly pale/posteriorly dark. Tibiae color (pro-, meso-, metatibia): mostly dark but anterior 0.2 pale, mostly dark but anterior 0.2 pale, anteriorly pale/posteriorly dark. Tegula and humeral complex color: both pale. Pterostigma color: dark. Fore wing veins color: mostly dark (a few veins may be unpigmented). Antenna length/body length: antenna about as long as body (head to apex of metasoma); if slightly shorter, at least extending beyond anterior 0.7 metasoma length. Body in lateral view: not distinctly flattened dorso–ventrally. Body length (head to apex of metasoma): 3.3–3.4 mm, 3.5–3.6 mm, rarely 3.1–3.2 mm. Fore wing length: 3.5–3.6 mm or 3.7–3.8 mm. Ocular–ocellar line/posterior ocellus diameter: 2.0–2.2. Interocellar distance/posterior ocellus diameter: 1.4–1.6. Antennal flagellomerus 2 length/width: 2.3–2.5. Antennal flagellomerus 14 length/width: 1.4–1.6. Length of flagellomerus 2/length of flagellomerus 14: 2.0–2.2. Tarsal claws: with single basal spine–like seta. Metafemur length/width: 3.0–3.1. Metatibia inner spur length/metabasitarsus length: 0.4–0.5. Anteromesoscutum: mostly with shallow, sparse punctures (separated by more than 2.0 × its maximum diameter). Mesoscutellar disc: mostly smooth. Number of pits in scutoscutellar sulcus: 5 or 6. Maximum height of mesoscutellum lunules/maximum height of lateral face of mesoscutellum: 0.2–0.3. Propodeum areola: completely defined by carinae, including transverse carina extending to spiracle. Propodeum background sculpture: mostly sculptured. Mediotergite 1 length/width at posterior margin: 3.5–3.7. Mediotergite 1 shape: mostly parallel–sided for 0.5–0.7 of its length, then narrowing posteriorly so mediotergite anterior width >1.1 × posterior width. Mediotergite 1 sculpture: mostly sculptured, excavated area centrally with transverse striation inside and/or a polished knob centrally on posterior margin of mediotergite. Mediotergite 2 width at posterior margin/length: 3.2–3.5. Mediotergite 2 sculpture: mostly smooth. Outer margin of hypopygium: with a wide, medially folded, transparent, semi–desclerotized area; usually with 4 or more pleats. Ovipositor thickness: about same width throughout its length. Ovipositor sheaths length/metatibial length: 0.8–0.9. Length of fore wing veins r/2RS: 2.0–2.2. Length of fore wing veins 2RS/2M: 1.1–1.3. Length of fore wing veins 2M/(RS+M)b: 0.7–0.8. Pterostigma length/width: 3.6 or more. Point of insertion of vein r in pterostigma: clearly beyond half way point length of pterostigma. Angle of vein r with fore wing anterior margin: clearly outwards, inclined towards fore wing apex. Shape of junction of veins r and 2RS in fore wing: distinctly but not strongly angled.

**Male.** As in female.

#### Molecular data.

Sequences in BOLD: 16, barcode compliant sequences: 15.

#### Biology/ecology.

Solitary (Fig. 295). Hosts: Crambidae, *Desmia octomaculalis*, *Desmia* benealisDHJ02, *Desmia* BioLep02, *Desmia* BioLep06, *Desmia* ploralisDHJ03, *Desmia* Solis19, *Trichaea pilicornis*.

#### Distribution.

Costa Rica, ACG.

#### Comments.

This species is characterized by its orange head and mesosternum, metasoma fully black, and propodeal areola closed basally and with transverse carinae reaching spiracles.

#### Etymology.

We dedicate this species to Luis Garita in recognition of his diligent efforts for the ACG Programa de Sectores.

### Apanteles luishernandezi

Fernández-Triana sp. n.

http://zoobank.org/6F6D1CF0-4880-4118-A308-201AE32721B0

http://species-id.net/wiki/Apanteles_luishernandezi

Figs 115 , 281

#### Type locality.

COSTA RICA, Guanacaste, ACG, Sector Del Oro, Rio Chon, 320m, 11.04118, -85.44170.

#### Holotype.

♀ in CNC. Specimen labels: 1. DHJPAR0031085. 2. COSTA RICA, Guanacaste, ACG, Sector Del Oro, Rio Chon, 1.iii.2008, 11.04118 N, -85.44170 W, 320m, DHJPAR0031085.

#### Paratypes.

2 ♀, 1 ♂ (CNC). COSTA RICA, ACG database codes: DHJPAR0031085, DHJPAR0031092, DHJPAR0031093.

#### Description.

**Female.** Body color: head dark, mesosoma dark with parts of axillar complex pale, metasoma with some mediotergites, most laterotergites, sternites, and/or hypopygium pale. Antenna color: scape, pedicel, and flagellum dark. Coxae color (pro-, meso-, metacoxa): pale, pale, partially pale/partially dark. Femora color (pro-, meso-, metafemur): pale, pale, mostly dark but with pale spot antero–ventrally. Tibiae color (pro-, meso-, metatibia): pale, pale, anteriorly pale/posteriorly dark. Tegula and humeral complex color: both pale. Pterostigma color: dark. Fore wing veins color: mostly dark (a few veins may be unpigmented). Antenna length/body length: antenna about as long as body (head to apex of metasoma); if slightly shorter, at least extending beyond anterior 0.7 metasoma length. Body in lateral view: not distinctly flattened dorso–ventrally. Body length (head to apex of metasoma): 3.1–3.2 mm or 3.3–3.4 mm. Fore wing length: 3.5–3.6 mm or 3.7–3.8 mm. Ocular–ocellar line/posterior ocellus diameter: 2.0–2.2. Interocellar distance/posterior ocellus diameter: 2.0–2.2. Antennal flagellomerus 2 length/width: 2.6–2.8. Antennal flagellomerus 14 length/width: 1.7–1.9. Length of flagellomerus 2/length of flagellomerus 14: 2.0–2.2. Tarsal claws: with single basal spine–like seta. Metafemur length/width: 3.0–3.1. Metatibia inner spur length/metabasitarsus length: 0.4–0.5. Anteromesoscutum: mostly with deep, dense punctures (separated by less than 2.0 × its maximum diameter). Mesoscutellar disc: mostly punctured. Number of pits in scutoscutellar sulcus: 9 or 10. Maximum height of mesoscutellum lunules/maximum height of lateral face of mesoscutellum: 0.4–0.5. Propodeum areola: completely defined by carinae, including transverse carina extending to spiracle. Propodeum background sculpture: mostly sculptured. Mediotergite 1 length/width at posterior margin: 2.3–2.5. Mediotergite 1 shape: mostly parallel–sided for 0.5–0.7 of its length, then narrowing posteriorly so mediotergite anterior width >1.1 × posterior width. Mediotergite 1 sculpture: mostly sculptured, excavated area centrally with transverse striation inside and/or a polished knob centrally on posterior margin of mediotergite. Mediotergite 2 width at posterior margin/length: 3.2–3.5. Mediotergite 2 sculpture: mostly smooth. Outer margin of hypopygium: with a wide, medially folded, transparent, semi–desclerotized area; usually with 4 or more pleats. Ovipositor thickness: about same width throughout its length. Ovipositor sheaths length/metatibial length: 0.4–0.5. Length of fore wing veins r/2RS: 2.3 or more. Length of fore wing veins 2RS/2M: 1.1–1.3. Length of fore wing veins 2M/(RS+M)b: 0.7–0.8. Pterostigma length/width: 3.1–3.5. Point of insertion of vein r in pterostigma: about half way point length of pterostigma. Angle of vein r with fore wing anterior margin: clearly outwards, inclined towards fore wing apex. Shape of junction of veins r and 2RS in fore wing: distinctly but not strongly angled.

**Male.** Similar to female, with darker metafemur and metatibia.

#### Molecular data.

Sequences in BOLD: 4, barcode compliant sequences: 3.

#### Biology/ecology.

Solitary (Fig. 281). Host: Crambidae, *Piletosoma thialis*.

#### Distribution.

Costa Rica, ACG.

#### Etymology.

We dedicate this species to Luis Hernández in recognition of his diligent efforts for the ACG Programa de Seguridad.

### Apanteles luislopezi

Fernández-Triana sp. n.

http://zoobank.org/2C05F004-FC97-4D84-9076-28B288472F00

http://species-id.net/wiki/Apanteles_luislopezi

Figs 16 , 218

Apanteles Rodriguez48. Smith et al. (2008). Interim name provided by the authors.

#### Type locality.

COSTA RICA, Alajuela, ACG, Sector San Cristobal, Rio Areno, 460m, 10.91407, -85.38174.

#### Holotype.

♀ in CNC. Specimen labels: 1. Costa Rica: Alajuela, ACG, Sector San Cristobal, Rio Areno, 04.v.2009, 460m, 10.91407, -85.38174, 09-SRNP-2437.

#### Paratypes.

29 ♀, 34 ♂ (BMNH, CNC, INBIO, INHS, NMNH). COSTA RICA, ACG database codes: DHJPAR0020672, DHJPAR0035384, DHJPAR0039727, 00-SRNP-18136, 06-SRNP-55804, 09-SRNP-2437, 09-SRNP-75129, 09-SRNP-75216, 11-SRNP-55878.

#### Description.

**Female.** Body color: body mostly dark except for some sternites which may be pale. Antenna color: scape, pedicel, and flagellum dark. Coxae color (pro-, meso-, metacoxa): dark, dark, dark. Femora color (pro-, meso-, metafemur): anteriorly dark/posteriorly pale, dark, dark. Tibiae color (pro-, meso-, metatibia): pale, pale, mostly dark but anterior 0.2 or less pale. Tegula and humeral complex color: tegula pale, humeral complex half pale/half dark. Pterostigma color: mostly pale and/or transparent, with thin dark borders. Fore wing veins color: partially pigmented (a few veins may be dark but most are pale). Antenna length/body length: antenna about as long as body (head to apex of metasoma); if slightly shorter, at least extending beyond anterior 0.7 metasoma length. Body in lateral view: not distinctly flattened dorso–ventrally. Body length (head to apex of metasoma): 2.7–2.8 mm, 2.9–3.0 mm, rarely 2.3–2.4 mm. Fore wing length: 2.9–3.0 mm, 3.1–3.2 mm, rarely 2.3–2.4 mm or 2.5–2.6 mm. Ocular–ocellar line/posterior ocellus diameter: 2.3–2.5 or 2.6 or more. Interocellar distance/posterior ocellus diameter: 2.0–2.2, rarely 2.3–2.5. Antennal flagellomerus 2 length/width: 2.9–3.1. Antennal flagellomerus 14 length/width: 1.4–1.6. Length of flagellomerus 2/length of flagellomerus 14: 2.0–2.2. Tarsal claws: with single basal spine–like seta. Metafemur length/width: 3.0–3.1. Metatibia inner spur length/metabasitarsus length: 0.4–0.5. Anteromesoscutum: mostly with deep, dense punctures (separated by less than 2.0 × its maximum diameter). Mesoscutellar disc: with punctures near margins, central part mostly smooth. Number of pits in scutoscutellar sulcus: 7 or 8. Maximum height of mesoscutellum lunules/maximum height of lateral face of mesoscutellum: 0.6–0.7. Propodeum areola: completely defined by carinae, including transverse carina extending to spiracle. Propodeum background sculpture: mostly sculptured. Mediotergite 1 length/width at posterior margin: 2.0–2.2. Mediotergite 1 shape: slightly widening from anterior margin to 0.7–0.8 mediotergite length (where maximum width is reached), then narrowing towards posterior margin. Mediotergite 1 sculpture: mostly sculptured, excavated area centrally with transverse striation inside and/or a polished knob centrally on posterior margin of mediotergite. Mediotergite 2 width at posterior margin/length: 3.2–3.5. Mediotergite 2 sculpture: with some sculpture, mostly near posterior margin. Outer margin of hypopygium: with a wide, medially folded, transparent, semi–desclerotized area; usually with 4 or more pleats. Ovipositor thickness: about same width throughout its length. Ovipositor sheaths length/metatibial length: 1.2–1.3 or 1.4–1.5. Length of fore wing veins r/2RS: 2.3 or more. Length of fore wing veins 2RS/2M: 1.1–1.3. Length of fore wing veins 2M/(RS+M)b: 0.5–0.6. Pterostigma length/width: 3.6 or more. Point of insertion of vein r in pterostigma: clearly beyond half way point length of pterostigma. Angle of vein r with fore wing anterior margin: clearly outwards, inclined towards fore wing apex. Shape of junction of veins r and 2RS in fore wing: distinctly but not strongly angled.

**Male.** As in female.

#### Molecular data.

Sequences in BOLD: 17, barcode compliant sequences: 17.

#### Biology/ecology.

Gregarious (Fig. 218). Host: Elachistidae, *Anadasmus* Janzen25, *Cerconota* Janzen104.

#### Distribution.

Costa Rica, ACG.

#### Etymology.

We dedicate this species to Luis López in recognition of his diligent efforts for the ACG Programa de Seguridad.

### Apanteles luisvargasi

Fernández-Triana sp. n.

http://zoobank.org/976880BD-A308-43D8-B5AF-FB8B885E1848

http://species-id.net/wiki/Apanteles_luisvargasi

Fig. 140

Apanteles Rodriguez99 (Smith et al. 2006). Interim name provided by the authors.

#### Type locality.

COSTA RICA, Guanacaste, ACG, Sector Santa Rosa, Bosque San Emilio, 300m, 10.84389, -85.61384.

#### Holotype.

♀ in CNC. Specimen labels: 1. Costa Rica: Guanacaste, ACG, Sector Santa Rosa, Bosque San Emilio, 19 May 2001, gusaneros. 2. 01-SRNP-12432, Dichogama colotha, On Capparis indica. 3. DHJPAR0005269.

#### Paratypes.

4 ♂ (CNC, NMNH). COSTA RICA, ACG database codes: DHJPAR0005277, DHJPAR0005278, DHJPAR0005281, DHJPAR0012478.

#### Description.

**Female.** Body color: body mostly dark except for some sternites which may be pale. Antenna color: scape, pedicel, and flagellum dark. Coxae color (pro-, meso-, metacoxa): dark, dark, dark. Femora color (pro-, meso-, metafemur): pale, anteriorly dark/posteriorly pale, dark. Tibiae color (pro-, meso-, metatibia): pale, pale, anteriorly pale/posteriorly dark. Tegula and humeral complex color: tegula pale, humeral complex half pale/half dark. Pterostigma color: mostly pale and/or transparent, with thin dark borders. Fore wing veins color: mostly white or entirely transparent. Antenna length/body length: antenna about as long as body (head to apex of metasoma); if slightly shorter, at least extending beyond anterior 0.7 metasoma length. Body in lateral view: not distinctly flattened dorso–ventrally. Body length (head to apex of metasoma): 3.1–3.2 mm. Fore wing length: 3.1–3.2 mm. Ocular–ocellar line/posterior ocellus diameter: 1.7–1.9. Interocellar distance/posterior ocellus diameter: 2.0–2.2. Antennal flagellomerus 2 length/width: 2.0–2.2. Antennal flagellomerus 14 length/width: 1.1–1.3. Length of flagellomerus 2/length of flagellomerus 14: 2.0–2.2. Tarsal claws: with single basal spine–like seta (?). Anteromesoscutum: mostly with deep, dense punctures (separated by less than 2.0 × its maximum diameter). Mesoscutellar disc: mostly smooth. Number of pits in scutoscutellar sulcus: 9 or 10. Maximum height of mesoscutellum lunules/maximum height of lateral face of mesoscutellum: 0.6–0.7. Propodeum areola: completely defined by carinae, including transverse carina extending to spiracle. Propodeum background sculpture: partly sculptured, especially on anterior 0.5. Mediotergite 1 length/width at posterior margin: 1.4–1.6. Mediotergite 1 shape: slightly widening from anterior margin to 0.7–0.8 mediotergite length (where maximum width is reached), then narrowing towards posterior margin. Mediotergite 1 sculpture: mostly sculptured, excavated area centrally with transverse striation inside and/or a polished knob centrally on posterior margin of mediotergite. Mediotergite 2 width at posterior margin/length: 4.4–4.7. Mediotergite 2 sculpture: mostly smooth. Outer margin of hypopygium: with a wide, medially folded, transparent, semi–desclerotized area; usually with 4 or more pleats. Ovipositor thickness: about same width throughout its length. Length of fore wing veins r/2RS: 2.0–2.2. Length of fore wing veins 2RS/2M: 1.4–1.6. Length of fore wing veins 2M/(RS+M)b: 0.5–0.6. Pterostigma length/width: 2.6–3.0. Point of insertion of vein r in pterostigma: clearly beyond half way point length of pterostigma. Angle of vein r with fore wing anterior margin: clearly outwards, inclined towards fore wing apex. Shape of junction of veins r and 2RS in fore wing: distinctly but not strongly angled.

**Male.** As in female, with slender mediotergite 1.

#### Molecular data.

Sequences in BOLD: 5, barcode compliant sequences: 5.

#### Biology/ecology.

Solitary. Host: Crambidae, *Dichogama colotha*.

#### Distribution.

Costa Rica, ACG.

#### Comments.

This is the only *Apanteles* species attacking *Dichogama* in ACG. The species is characterized by glossa elongate and mediotergite 1 with strong longitudinal striation, a unique combination of characters among Mesoamerican *Apanteles*.

#### Etymology.

We dedicate this species to Luis Vargas in recognition of his diligent efforts for the ACG Programa de Ecoturismo.

### Apanteles luzmariaromeroae

Fernández-Triana sp. n.

http://zoobank.org/C2FB463C-ABF6-4005-ACA4-2E53B73BF14B

http://species-id.net/wiki/Apanteles_luzmariaromeroae

Fig. 194

Apanteles Rodriguez38 (Smith et al. 2006). Interim name provided by the authors.

#### Type locality.

COSTA RICA, Guanacaste, ACG, Sector Cacao, Estación Cacao, 1150m, 10.92691, -85.46822.

#### Holotype.

♀ in CNC. Specimen labels: 1. DHJPAR0005310.

#### Paratypes.

20 ♀, 12 ♂ (BMNH, CNC, INBIO, INHS, NMNH). COSTA RICA, ACG database codes: DHJPAR0001563, DHJPAR0002978, DHJPAR0005203, DHJPAR0005216.

#### Description.

**Female.** Metatibia color (outer face): entirely or mostly (>0.7 metatibia length) dark brown to black, with yellow to white coloration usually restricted to anterior 0.2 or less. Fore wing veins color: veins C+Sc+R and R1 with brown coloration restricted narrowly to borders, interior area of those veins and pterostigma (and sometimes veins r, 2RS and 2M) transparent or white; other veins mostly transparent. Antenna length/body length: antenna about as long as body (head to apex of metasoma); if slightly shorter, at least extending beyond anterior 0.7 metasoma length. Body length (head to apex of metasoma): 2.1–2.2 mm or 2.3–2.4 mm. Fore wing length: 2.3–2.4 mm or 2.5–2.6 mm. Metafemur length/width: 2.8–2.9, 3.0–3.1 or 3.2–3.3. Mediotergite 1 length/width at posterior margin: 2.7–2.8. Mediotergite 1 maximum width/width at posterior margin: 1.6–1.7. Ovipositor sheaths length/metafemur length: 1.2 or 1.3. Ovipositor sheaths length/metatibia length: 1.0 or 1.1.

#### Molecular data.

Sequences in BOLD: 5, barcode compliant sequences: 3.

#### Biology/ecology.

Gregarious. Host: Hesperiidae, *Codatractus imalena*.

#### Distribution.

Costa Rica, ACG.

#### Etymology.

We dedicate this species to Luz María Romero in recognition of his unwavering support for conservation in Costa Rica, for the founding of ACG, and for the administration of Costa Rica’s Paz con la Naturaleza initiative.

### Apanteles manuelarayai

Fernández-Triana sp. n.

http://zoobank.org/D8ED91F2-0B25-4A20-9BB3-AB32D907C927

http://species-id.net/wiki/Apanteles_manuelarayai

Figs 17 , 219

#### Type locality.

COSTA RICA, Alajuela, ACG, Sector Pitilla, Estación Quica, 470m, 10.99697, -85.39666.

#### Holotype.

♀ in CNC. Specimen labels: 1. DHJPAR0038109. 2. COSTA RICA, Guanacaste, ACG, Sector Pitilla, Estación Quica, 20.viii.2009, 10.99697°N, -85.39666°W, 470m, DHJPAR0038109. 3. Voucher: D.H.Janzen & W.Hallwachs, DB: http://janzen.sas.upenn.edu, Area de Conservación Guanacaste, COSTA RICA, 09-SRNP-72674.

#### Paratypes.

4 ♀ (CNC, NMNH). COSTA RICA: Guanacaste, ACG database code: 09-SRNP-72674.

#### Description.

**Female.** Body color: body mostly dark except for some sternites which may be pale. Antenna color: scape, pedicel, and flagellum dark. Coxae color (pro-, meso-, metacoxa): dark, dark, dark. Femora color (pro-, meso-, metafemur): anteriorly dark/posteriorly pale, dark, dark. Tibiae color (pro-, meso-, metatibia): pale, pale, mostly dark but anterior 0.2 or less pale. Tegula and humeral complex color: tegula pale, humeral complex half pale/half dark. Pterostigma color: mostly pale and/or transparent, with thin dark borders. Fore wing veins color: partially pigmented (a few veins may be dark but most are pale). Antenna length/body length: antenna about as long as body (head to apex of metasoma); if slightly shorter, at least extending beyond anterior 0.7 metasoma length. Body in lateral view: not distinctly flattened dorso–ventrally. Body length (head to apex of metasoma): 2.5–2.6 mm, rarely 2.7–2.8 mm. Fore wing length: 2.9–3.0 mm. Ocular–ocellar line/posterior ocellus diameter: 2.3–2.5. Interocellar distance/posterior ocellus diameter: 1.7–1.9. Antennal flagellomerus 2 length/width: 2.9–3.1. Antennal flagellomerus 14 length/width: 1.7–1.9. Length of flagellomerus 2/length of flagellomerus 14: 2.0–2.2. Tarsal claws: simple. Metafemur length/width: 2.8–2.9. Metatibia inner spur length/metabasitarsus length: 0.4–0.5. Anteromesoscutum: mostly with deep, dense punctures (separated by less than 2.0 × its maximum diameter). Mesoscutellar disc: with punctures near margins, central part mostly smooth. Number of pits in scutoscutellar sulcus: 7 or 8. Maximum height of mesoscutellum lunules/maximum height of lateral face of mesoscutellum: 0.4–0.5. Propodeum areola: completely defined by carinae, including transverse carina extending to spiracle. Propodeum background sculpture: mostly sculptured. Mediotergite 1 length/width at posterior margin: 2.0–2.2. Mediotergite 1 shape: slightly widening from anterior margin to 0.7–0.8 mediotergite length (where maximum width is reached), then narrowing towards posterior margin. Mediotergite 1 sculpture: mostly sculptured, excavated area centrally with transverse striation inside and/or a polished knob centrally on posterior margin of mediotergite. Mediotergite 2 width at posterior margin/length: 2.8–3.1. Mediotergite 2 sculpture: mostly smooth. Outer margin of hypopygium: with a wide, medially folded, transparent, semi–desclerotized area; usually with 4 or more pleats. Ovipositor thickness: anterior width at most 2.0 × posterior width (beyond ovipositor constriction). Ovipositor sheaths length/metatibial length: 1.0–1.1. Length of fore wing veins r/2RS: 1.7–1.9. Length of fore wing veins 2RS/2M: 1.1–1.3. Length of fore wing veins 2M/(RS+M)b: 0.7–0.8. Pterostigma length/width: 3.6 or more. Point of insertion of vein r in pterostigma: clearly beyond half way point length of pterostigma. Angle of vein r with fore wing anterior margin: clearly outwards, inclined towards fore wing apex. Shape of junction of veins r and 2RS in fore wing: distinctly but not strongly angled.

**Male.** Unknown.

#### Molecular data.

Sequences in BOLD: 13, barcode compliant sequences: 13.

#### Biology/ecology.

Gregarious (Fig. 219). Host: Elachistidae, *Stenoma* sp. 09-SRNP-72674.

#### Distribution.

Costa Rica, ACG.

#### Etymology.

We dedicate this species to Manuel Araya in recognition of his diligent efforts for the ACG Programa de Sectores.

### Apanteles manuelpereirai

Fernánez-Triana sp. n.

http://zoobank.org/78C365B3-9F4D-4C58-8A51-E55E5C258AAA

http://species-id.net/wiki/Apanteles_manuelpereirai

Figs 61 , 254

Apanteles Rodriguez09 (Smith et al. 2006). Interim name provided by the authors.

#### Type locality.

COSTA RICA, Guanacaste, ACG, Sector Pitilla, Pasmompa, 440m, 11.01926, -85.40997.

#### Holotype.

♀ in CNC. Specimen labels: 1. COSTA RICA, Guanacaste, ACG, Sector Pitilla, Pasmompa, 28.vi.2004, 440m, 11.01926, -85.40997, 04-SRNP-33763. 2. DHJPAR0003041.

#### Paratypes.

24 ♀, 15 ♂ (BMNH, CNC, INBIO, INHS, NMNH). COSTA RICA: ACG database codes: 98-SRNP-4293, 01-SRNP-5181, 01-SRNP-9182, 02-SRNP-4541, 02-SRNP-5615, 02-SRNP-6229, 03-SRNP-5963, 03-SRNP-7197, 03-SRNP-20163, 03-SRNP-20799, 03-SRNP-20933, 03-SRNP-21562, 03-SRNP-21736, 03-SRNP-21892, 04-SRNP-33763.

#### Description.

**Female.** Body color: head and mesosoma mostly dark, metasoma with some tergites and/or most of sternites pale. Antenna color: scape, pedicel, and flagellum pale, rarely scape, pedicel, and flagellum dark. Coxae color (pro-, meso-, metacoxa): pale, pale, dark or pale, pale, pale. Femora color (pro-, meso-, metafemur): pale, pale, mostly pale but posterior 0.2 or less dark. Tibiae color (pro-, meso-, metatibia): pale, pale, mostly pale but with posterior 0.2 or less dark. Tegula and humeral complex color: both pale. Pterostigma color: dark. Fore wing veins color: mostly dark (a few veins may be unpigmented). Antenna length/body length: antenna shorter than body (head to apex of metasoma), not extending beyond anterior 0.7 metasoma length. Body in lateral view: not distinctly flattened dorso–ventrally. Body length (head to apex of metasoma): 2.7–2.8 mm, 2.9–3.0 mm, rarely 3.1–3.2 mm. Fore wing length: 2.9–3.0 mm, 3.1–3.2 mm, rarely 2.7–2.8 mm. Ocular–ocellar line/posterior ocellus diameter: 2.0–2.2. Interocellar distance/posterior ocellus diameter: 1.4–1.6. Antennal flagellomerus 2 length/width: 2.3–2.5. Antennal flagellomerus 14 length/width: 1.4–1.6. Length of flagellomerus 2/length of flagellomerus 14: 2.0–2.2. Tarsal claws: with single basal spine–like seta. Metafemur length/width: 2.8–2.9. Metatibia inner spur length/metabasitarsus length: 0.4–0.5. Anteromesoscutum: mostly with deep, dense punctures (separated by less than 2.0 × its maximum diameter). Mesoscutellar disc: mostly punctured. Number of pits in scutoscutellar sulcus: 7 or 8. Maximum height of mesoscutellum lunules/maximum height of lateral face of mesoscutellum: 0.6–0.7. Propodeum areola: completely defined by carinae, including transverse carina extending to spiracle. Propodeum background sculpture: partly sculptured, especially on anterior 0.5. Mediotergite 1 length/width at posterior margin: 2.6–2.8. Mediotergite 1 shape: mostly parallel–sided for 0.5–0.7 of its length, then narrowing posteriorly so mediotergite anterior width >1.1 × posterior width, rarely slightly widening from anterior margin to 0.7–0.8 mediotergite length (where maximum width is reached), then narrowing towards posterior margin. Mediotergite 1 sculpture: with some sculpture near lateral margins and/or posterior 0.2–0.4 of mediotergite. Mediotergite 2 width at posterior margin/length: 3.6–3.9. Mediotergite 2 sculpture: mostly smooth. Outer margin of hypopygium: inflexible (without a folded, transparent, semi–desclerotized area); with no pleats visible. Ovipositor thickness: anterior width 3.0–5.0 × posterior width (beyond ovipositor constriction). Ovipositor sheaths length/metatibial length: 0.8–0.9, rarely 0.6–0.7. Length of fore wing veins r/2RS: 1.7–1.9. Length of fore wing veins 2RS/2M: 1.4–1.6. Length of fore wing veins 2M/(RS+M)b: 0.7–0.8. Pterostigma length/width: 3.1–3.5. Point of insertion of vein r in pterostigma: clearly beyond half way point length of pterostigma. Angle of vein r with fore wing anterior margin: more or less perpendicular to fore wing margin. Shape of junction of veins r and 2RS in fore wing: distinctly but not strongly angled.

**Male.** Similar to females except for darker legs and metasoma.

#### Molecular data.

Sequences in BOLD: 42, barcode compliant sequences: 38.

#### Biology/ecology.

Gregarious (Fig. 254). Host: Hesperiidae, *Telemiades antiope*.

#### Distribution.

Costa Rica, ACG.

#### Etymology.

We dedicate this species to Manuel Pereira recognition of his diligent efforts for the ACG Programa de Parataxónomos and Estación Biológica Cacao.

### Apanteles manuelriosi

Fernández-Triana sp. n.

http://zoobank.org/13875EE8-1EBD-4747-98AF-B96A8D05DC96

http://species-id.net/wiki/Apanteles_manuelriosi

Figs 88 , 267

Apanteles Rodriguez60 (Smith et al. 2006). Interim name provided by the authors.

#### Type locality.

COSTA RICA, Guanacaste, Sector Pitilla, Loaiciga, 445m, 11.01983, -85.41342.

#### Holotype.

♀ in CNC. Specimen labels: 1. COSTA RICA: Guanacaste, ACG, Sector Pitilla, Loaiciga, 8.vi.2004, 445m, 11.01983, -85.41342. 2. DHJPAR0002914. 3. Voucher: D.H.Janzen & W.Hallwachs, DB: http://janzen.sas.upenn.edu, Area de Conservación Guanacaste, COSTA RICA, 04-SRNP-33152.

#### Paratypes.

7 ♀, 2 ♂ (BMNH, CNC, INBIO, INHS, NMNH). COSTA RICA, ACG database codes: 97-SRNP-5757, 02-SRNP-3735, 04-SRNP-56294, 05-SRNP-33209, 06-SRNP-31198, 06-SRNP-32523, 07-SRNP-33461, 08-SRNP-65992, 08-SRNP-70494.

#### Description.

**Female.** Body color: body mostly dark except for some sternites which may be pale. Antenna color: scape, pedicel, and flagellum dark. Coxae color (pro-, meso-, metacoxa): dark, dark, dark. Femora color (pro-, meso-, metafemur): pale, pale, pale. Tibiae color (pro-, meso-, metatibia): pale, pale, mostly pale but with posterior 0.2 or less dark. Tegula and humeral complex color: tegula pale, humeral complex dark. Pterostigma color: dark. Fore wing veins color: mostly dark (a few veins may be unpigmented). Antenna length/body length: antenna about as long as body (head to apex of metasoma); if slightly shorter, at least extending beyond anterior 0.7 metasoma length. Body in lateral view: not distinctly flattened dorso–ventrally. Body length (head to apex of metasoma): 4.0 mm or more. Fore wing length: 4.0 mm or more. Ocular–ocellar line/posterior ocellus diameter: 2.0–2.2. Interocellar distance/posterior ocellus diameter: 2.0–2.2. Antennal flagellomerus 2 length/width: 2.3–2.5, 2.6–2.8, rarely 2.0–2.2. Antennal flagellomerus 14 length/width: 2.6–2.9. Length of flagellomerus 2/length of flagellomerus 14: 1.1–1.3. Tarsal claws: pectinate. Metafemur length/width: 3.2–3.3. Metatibia inner spur length/metabasitarsus length: 0.6–0.7. Anteromesoscutum: mostly with deep, dense punctures (separated by less than 2.0 × its maximum diameter). Mesoscutellar disc: mostly punctured. Number of pits in scutoscutellar sulcus: 7 or 8 or 9 or 10. Maximum height of mesoscutellum lunules/maximum height of lateral face of mesoscutellum: 0.4–0.5. Propodeum areola: completely defined by carinae, including transverse carina extending to spiracle. Propodeum background sculpture: mostly sculptured. Mediotergite 1 length/width at posterior margin: 1.7–1.9. Mediotergite 1 shape: more or less parallel–sided. Mediotergite 1 sculpture: mostly smooth. Mediotergite 2 width at posterior margin/length: 3.6–3.9. Mediotergite 2 sculpture: mostly smooth. Outer margin of hypopygium: with a wide, medially folded, transparent, semi–desclerotized area; usually with 4 or more pleats. Ovipositor thickness: anterior width 3.0–5.0 × posterior width (beyond ovipositor constriction). Ovipositor sheaths length/metatibial length: 0.8–0.9. Length of fore wing veins r/2RS: 2.3 or more. Length of fore wing veins 2RS/2M: 1.7–1.8. Length of fore wing veins 2M/(RS+M)b: 0.5–0.6. Pterostigma length/width: 3.1–3.5. Point of insertion of vein r in pterostigma: clearly beyond half way point length of pterostigma. Angle of vein r with fore wing anterior margin: clearly outwards, inclined towards fore wing apex. Shape of junction of veins r and 2RS in fore wing: distinctly but not strongly angled.

**Male.** The pterostigma and most of veins in fore wing are transparent, while the metasomal terga tend to be lighter in color.

#### Molecular data.

Sequences in BOLD: 22, barcode compliant sequences: 20.

#### Biology/ecology.

Solitary (Fig. 267). Host: Hesperiidae, *Quadrus cerialis*.

#### Distribution.

Costa Rica, ACG.

#### Etymology.

We dedicate this species to Manuel Ríos in recognition of his diligent efforts for the ACG Programa de Parataxónomos and Estación Biológica Pitilla of ACG.

### Apanteles manuelzumbadoi

Fernández-Triana sp. n.

http://zoobank.org/FE13C222-ABCC-48CF-90E9-94B5CE53BF5D

http://species-id.net/wiki/Apanteles_manuelzumbadoi

Figs 190 , 319

Apanteles Rodriguez26 (Smith et al. 2006). Interim name provided by the authors.

#### Type locality.

COSTA RICA, Guanacaste, ACG, Sector Santa Rosa, Vado Cuajiniquil, 5m, 10.94041, -85.68043.

#### Holotype.

♀ in CNC. Specimen labels: 1. DHJPAR0013684. 2. COSTA RICA: Guanacaste: Area de Conservación Guancaste: Santa Rosa, Vado Cuajiniquil, 01/25/1992: gusaneros. 3. 92-SRNP-251, Telemiades fides, Inga vera.

#### Paratypes.

186 ♀, 78 ♂ (BMNH, CNC, INBIO, INHS, NMNH). COSTA RICA, ACG database codes: See Appendix 2 for detailed label data.

#### Description.

**Female.** Metatibia color (outer face): entirely or mostly (>0.7 metatibia length) dark brown to black, with yellow to white coloration usually restricted to anterior 0.2 or less, rarely with extended pale coloration (light yellow to orange–yellow), ranging from 0.4 to almost entire metatibia length. Fore wing veins color: veins C+Sc+R and R1 mostly brown; usually veins r, 2RS, 2M, (RS+M)b, 1CU, 2Cua, and 1m–cu partially brown; interior area of other veins, and at least part of pterostigma, usually light brown or yellowish–white, rarely veins C+Sc+R and R1 with brown coloration restricted narrowly to borders, interior area of those veins and pterostigma (and sometimes veins r, 2RS and 2M) transparent or white; other veins mostly transparent. Antenna length/body length: antenna about as long as body (head to apex of metasoma); if slightly shorter, at least extending beyond anterior 0.7 metasoma length. Body length (head to apex of metasoma): 2.1–2.2 mm or 2.3–2.4 mm. Fore wing length: 2.3–2.4 mm or 2.5–2.6 mm. Metafemur length/width: 2.8–2.9 or 3.0–3.1. Mediotergite 1 length/width at posterior margin: 2.3–2.4, rarely 2.5–2.6. Mediotergite 1 maximum width/width at posterior margin: 1.2–1.3 or 1.4–1.5. Ovipositor sheaths length/metafemur length: 1.0. Ovipositor sheaths length/metatibia length: 0.8 or 0.9.

#### Molecular data.

Sequences in BOLD: 204, barcode compliant sequences: 189.

#### Biology/ecology.

Gregarious (Fig. 319). Host: Hesperiidae, *Telemiades fides*.

#### Distribution.

Costa Rica, ACG.

#### Etymology.

We dedicate this species to Manuel Zumbado in recognition of his diligent efforts for the ACG Programa de Parataxónomos, administration and Diptera curatorial taxonomy for INBio, Costa Rica’s Instituto Nacional de Biodiversidad, and for ACG.

### Apanteles marcobustosi

Fernández-Triana sp. n.

http://zoobank.org/436D5B40-1813-4219-94D1-9BCE3BA2F36F

http://species-id.net/wiki/Apanteles_marcobustosi

Figs 123 , 288

Apanteles Rodriguez34 (Smith et al. 2006). Interim name provided by the authors.

#### Type locality.

COSTA RICA, Guanacaste, ACG, Sector del Oro, Chon, 11.04788, -85.45266.

#### Holotype.

♀ in CNC. Specimen labels: 1. COSTA RICA, Guanacaste, ACG, Sector del Oro, Chon, 26.xi.2004, 280m, 11.04788, -85.45266, 04-SRNP-26690.

#### Paratypes.

35 ♀, 2 ♂ (BMNH, CNC, INBIO, INHS, NMNH). COSTA RICA, Alajuela, ACG database codes: 99-SRNP-5544, 99-SRNP-5547, 01-SRNP-5523, 04-SRNP-26690.

#### Description.

**Female.** Body color: body mostly dark except for some sternites which may be pale. Antenna color: scape, pedicel, and flagellum pale. Coxae color (pro-, meso-, metacoxa): dark, dark, dark. Femora color (pro-, meso-, metafemur): dark, dark, dark or anteriorly dark/posteriorly pale, dark, dark. Tibiae color (pro-, meso-, metatibia): pale, pale, anteriorly pale/posteriorly dark. Tegula and humeral complex color: both pale. Pterostigma color: dark. Fore wing veins color: mostly dark (a few veins may be unpigmented). Antenna length/body length: antenna shorter than body (head to apex of metasoma), not extending beyond anterior 0.7 metasoma length or antenna very short, barely or not extending beyond mesosoma length. Body in lateral view: distinctly flattened dorso–ventrally. Body length (head to apex of metasoma): 2.0 mm or less, 2.1–2.2 mm, rarely 2.3–2.4 mm. Fore wing length: 2.0 mm or less, 2.1–2.2 mm or 2.3–2.4 mm. Ocular–ocellar line/posterior ocellus diameter: 2.6 or more. Interocellar distance/posterior ocellus diameter: 1.4–1.6. Antennal flagellomerus 2 length/width: 1.7–1.9. Antennal flagellomerus 14 length/width: 1.0 or less. Length of flagellomerus 2/length of flagellomerus 14: 2.0–2.2. Tarsal claws: simple. Metafemur length/width: 2.8–2.9. Metatibia inner spur length/metabasitarsus length: 0.6–0.7. Anteromesoscutum: mostly smooth or with shallow sparse punctures, except for anterior 0.3 where it has deeper and/or denser punctures. Mesoscutellar disc: mostly smooth. Number of pits in scutoscutellar sulcus: 9 or 10 or 11 or 12. Maximum height of mesoscutellum lunules/maximum height of lateral face of mesoscutellum: 0.4–0.5, rarely 0.2–0.3. Propodeum areola: completely defined by carinae, but only partial or absent transverse carina. Propodeum background sculpture: mostly sculptured. Mediotergite 1 length/width at posterior margin: 2.6–2.8. Mediotergite 1 shape: mostly parallel–sided for 0.5–0.7 of its length, then narrowing posteriorly so mediotergite anterior width >1.1 × posterior width. Mediotergite 1 sculpture: with some sculpture near lateral margins and/or posterior 0.2–0.4 of mediotergite. Mediotergite 2 width at posterior margin/length: 3.2–3.5. Mediotergite 2 sculpture: mostly smooth. Outer margin of hypopygium: with a medially folded, transparent, semi–desclerotized area; with 0–3 pleats visible. Ovipositor thickness: anterior width at most 2.0 × posterior width (beyond ovipositor constriction). Ovipositor sheaths length/metatibial length: 0.4–0.5. Length of fore wing veins r/2RS: 1.4–1.6. Length of fore wing veins 2RS/2M: 0.9–1.0. Length of fore wing veins 2M/(RS+M)b: 1.1–1.3. Pterostigma length/width: 2.6–3.0. Point of insertion of vein r in pterostigma: about half way point length of pterostigma. Angle of vein r with fore wing anterior margin: more or less perpendicular to fore wing margin. Shape of junction of veins r and 2RS in fore wing: strongly angulated, sometimes with a knob.

**Male.** As in female, but with long antenna.

#### Molecular data.

Sequences in BOLD: 8, barcode compliant sequences: 8.

#### Biology/ecology.

Gregarious (Fig. 288). Host: Crambidae, *Pantographa expansalis*, *Phostria mapetalis*.

#### Distribution.

Costa Rica, ACG.

#### Etymology.

We dedicate this species to Marco Bustos in recognition of his diligent efforts for the ACG Programa de Ecoturismo.

### Apanteles marcogonzalezi

Fernández-Triana sp. n.

http://zoobank.org/CCF94897-A5DC-4279-A19E-D6C0CA81A58F

http://species-id.net/wiki/Apanteles_marcogonzalezi

Fig. 141

#### Type locality.

COSTA RICA, Alajuela, ACG, Sector San Cristobal, Bosque Trampa Malaise, 815m, 10.86280, -85.38460.

#### Holotype.

♀ in CNC. Specimen labels: 1. DHJPAR0027678. 2. San Gerardo: MT San Cristobal, Date: 5-11 Apr-2008.

#### Paratypes.

1 ♀, 3 ♂ (CNC, NMNH). COSTA RICA, ACG database codes: DHJPAR0026176, DHJPAR0026787, DHJPAR0027705, DHJPAR0027589.

#### Description.

**Female.** Body color: head dark, mesosoma dark with parts of axillar complex pale, metasoma with some mediotergites, most laterotergites, sternites, and/or hypopygium pale. Antenna color: scape, pedicel, and flagellum dark. Coxae color (pro-, meso-, metacoxa): pale, pale, pale. Femora color (pro-, meso-, metafemur): pale, anteriorly dark/posteriorly pale, anteriorly dark/posteriorly pale. Tibiae color (pro-, meso-, metatibia): pale, pale, anteriorly pale/posteriorly dark. Tegula and humeral complex color: both pale. Pterostigma color: dark with pale spot at base. Fore wing veins color: mostly dark (a few veins may be unpigmented). Antenna length/body length: antenna about as long as body (head to apex of metasoma); if slightly shorter, at least extending beyond anterior 0.7 metasoma length. Body in lateral view: not distinctly flattened dorso–ventrally. Body length (head to apex of metasoma): 2.9–3.0 mm or 3.1–3.2 mm. Fore wing length: 3.1–3.2 mm or 3.3–3.4 mm. Ocular–ocellar line/posterior ocellus diameter: 2.0–2.2. Interocellar distance/posterior ocellus diameter: 1.4–1.6. Antennal flagellomerus 2 length/width: 2.6–2.8. Antennal flagellomerus 14 length/width: 1.1–1.3. Length of flagellomerus 2/length of flagellomerus 14: 2.3–2.5. Tarsal claws: simple. Metafemur length/width: 3.2–3.3. Metatibia inner spur length/metabasitarsus length: 0.4–0.5. Anteromesoscutum: mostly with shallow, sparse punctures (separated by more than 2.0 × its maximum diameter). Mesoscutellar disc: with a few sparse punctures. Number of pits in scutoscutellar sulcus: 5 or 6. Maximum height of mesoscutellum lunules/maximum height of lateral face of mesoscutellum: 0.4–0.5. Propodeum areola: completely defined by carinae, including transverse carina extending to spiracle. Propodeum background sculpture: mostly sculptured. Mediotergite 1 length/width at posterior margin: 4.1 or more. Mediotergite 1 shape: clearly narrowing towards posterior margin. Mediotergite 1 sculpture: mostly sculptured, excavated area centrally with transverse striation inside and/or a polished knob centrally on posterior margin of mediotergite. Mediotergite 2 width at posterior margin/length: 2.8–3.1 or 3.2–3.5. Mediotergite 2 sculpture: mostly smooth. Outer margin of hypopygium: with a wide, medially folded, transparent, semi–desclerotized area; usually with 4 or more pleats. Ovipositor thickness: anterior width at most 2.0 × posterior width (beyond ovipositor constriction). Ovipositor sheaths length/metatibial length: 0.4–0.5. Length of fore wing veins r/2RS: 1.7–1.9. Length of fore wing veins 2RS/2M: 1.1–1.3. Length of fore wing veins 2M/(RS+M)b: 0.9–1.0. Pterostigma length/width: 2.6–3.0. Point of insertion of vein r in pterostigma: about half way point length of pterostigma. Angle of vein r with fore wing anterior margin: clearly outwards, inclined towards fore wing apex. Shape of junction of veins r and 2RS in fore wing: distinctly but not strongly angled.

**Male.** As in female.

#### Molecular data.

Sequences in BOLD: 16, barcode compliant sequences: 16.

#### Biology/ecology.

Solitary. Host: Crambidae, *Ategumia* Solis01.

#### Distribution.

Costa Rica, ACG.

#### Comments.

This species is characterized by relatively long mediotergite 1 (its length 4.5 × its width at apex), extensive yellow-orange coloration (including tegula and humeral complex, parts of the axillar complex, most of laterotergites 1-4, all sternites, and hypopygium), and ovipositor sheaths shorter than half metatibia length. Molecular data also supports this species as a very divergent one.

#### Etymology.

We dedicate this species to Marco González in recognition of his diligent efforts for the ACG Programa de Educacion.

### Apanteles marcovenicioi

Fernández-Triana sp. n.

http://zoobank.org/5834E3BB-5B7F-4B24-93E2-101EA95E6B46

http://species-id.net/wiki/Apanteles_marcovenicioi

Fig. 191

Apanteles Rodriguez95 (Smith et al. 2006). Interim name provided by the authors.

#### Type locality.

COSTA RICA, Guanacaste, ACG, Sector Del Oro, Camino Mangos, 480m, 11.00766, -85.47926.

#### Holotype.

♀ in CNC. Specimen labels: 1. DHJPAR0002695. 2. COSTA RICA, Guanacaste, ACG, Sector Del Oro, Camino Mangos, 11.vii.2003, 480m, 11.00766, -85.47926, 03-SRNP-16760.

#### Paratypes.

♀ (CNC). COSTA RICA: Guanacaste, ACG database code: 03-SRNP-16760

#### Description.

**Female.** Metatibia color (outer face): entirely or mostly (>0.7 metatibia length) dark brown to black, with yellow to white coloration usually restricted to anterior 0.2 or less. Fore wing veins color: veins C+Sc+R and R1 with brown coloration restricted narrowly to borders, interior area of those veins and pterostigma (and sometimes veins r, 2RS and 2M) transparent or white; other veins mostly transparent. Antenna length/body length: antenna about as long as body (head to apex of metasoma); if slightly shorter, at least extending beyond anterior 0.7 metasoma length. Body length (head to apex of metasoma): 2.1–2.2 mm. Fore wing length: 2.3–2.4 mm. Metafemur length/width: 2.8–2.9. Mediotergite 1 length/width at posterior margin: 2.5–2.6. Mediotergite 1 maximum width/width at posterior margin: 1.4–1.5. Ovipositor sheaths length/metafemur length: 1.3. Ovipositor sheaths length/metatibia length: 1.0.

#### Molecular data.

Sequences in BOLD: 1, barcode compliant sequences: 1.

#### Biology/ecology.

Gregarious. Hosts: Hesperiidae, *Astraptes talus*.

#### Distribution.

Costa Rica, ACG.

#### Etymology.

We dedicate this species to Marco Venicio in recognition of his diligent efforts to support the founding stages of ACG by Direccion Forestal of MINAE, Costa Rica’s Ministerio del Ambiente y Energía.

### Apanteles mariachavarriae

Fernández-Triana sp. n.

http://zoobank.org/28572D1D-9DD0-4772-BE33-6D4053487469

http://species-id.net/wiki/Apanteles_mariachavarriae

Figs 165 , 320

Apanteles Rodriguez29 (Smith et al. 2006). Interim name provided by the authors.

#### Type locality.

COSTA RICA, Guanacaste, ACG, Sector Santa Rosa, Vado Rio Calera, 10m, 10.80274, -85.67423.

#### Holotype.

♀ in CNC. Specimen labels: 1. DHJPAR0005295. 2. COSTA RICA, Guanacaste, Area de Conservacion Guanacaste: Sector Santa Rosa: Vado Rio Calera, 07/09/1997: Harry Ramirez. 3. 97-SRNP-3256, Urbanus same as 97-3251, Poaceae sp. 13516.

#### Paratypes.

27 ♀, 10 ♂ (BMNH, CNC, INBIO, INHS, NMNH). COSTA RICA, ACG database codes: DHJPAR0001674, DHJPAR0001680, DHJPAR0005295, DHJPAR0013679, 97-SRNP-3252, 01-SRNP-17370.

#### Description.

**Female.** Metatibia color (outer face): with extended pale coloration (light yellow to orange–yellow), ranging from 0.4 to almost entire metatibia length. Fore wing veins color: veins C+Sc+R and R1 with brown coloration restricted narrowly to borders, interior area of those veins and pterostigma (and sometimes veins r, 2RS and 2M) transparent or white; other veins mostly transparent. Antenna length/body length: antenna about as long as body (head to apex of metasoma); if slightly shorter, at least extending beyond anterior 0.7 metasoma length. Body length (head to apex of metasoma): 2.3–2.4 mm or 2.5–2.6 mm. Fore wing length: 2.5–2.6 mm. Metafemur length/width: 2.8–2.9, 3.0–3.1, 3.2–3.3, rarely 3.4–3.5. Mediotergite 1 length/width at posterior margin: 2.1–2.2. Mediotergite 1 maximum width/width at posterior margin: 1.4–1.5. Ovipositor sheaths length/metafemur length: 0.7 or 0.8. Ovipositor sheaths length/metatibia length: 0.6 or 0.7.

#### Molecular data.

Sequences in BOLD: 7, barcode compliant sequences: 5.

#### Biology/ecology.

Gregarious (Fig. 320). Host: Hesperiidae, *Urbanus teleus*.

#### Distribution.

Costa Rica, ACG.

#### Etymology.

We dedicate this species to Maria Marta Chavarria in recognition of her diligent efforts for the ACG Programa de Parataxónomos, Programa Marino, and Programa de Biosensibilizacion, and for INBio, Costa Rica’s Instituto Nacional de Biodiversidad.

### Apanteles mariaguevarae

Fernández-Triana sp. n.

http://zoobank.org/6FAC51A1-3A40-4169-BED7-16AF1946DAFB

http://species-id.net/wiki/Apanteles_mariaguevarae

Fig. 109

#### Type locality.

COSTA RICA, Guanacaste, ACG, Sector Santa Rosa, Bosque San Emilio, 300m, 10.84389, -85.61384.

#### Holotype.

♀ in CNC. Specimen labels: 1. DHJPAR0012537. 2. COSTA RICA, Guanacaste, ACG, Sector Santa Rosa, Bosque San Emilio, 300m, 10.84389°N, -85.61384°W, 7.xii.1998, DHJPAR0012537. 3. 36-23 Nov 98-SE.

#### Paratypes.

1 ♂ (CNC). COSTA RICA: Guanacaste, ACG database code: DHJPAR0038045.

#### Description.

**Female.** Body color: body mostly dark except for some sternites which may be pale. Antenna color: scape, pedicel, and flagellum dark. Coxae color (pro-, meso-, metacoxa): dark, dark, dark. Femora color (pro-, meso-, metafemur): pale, anteriorly dark/posteriorly pale, dark. Tibiae color (pro-, meso-, metatibia): pale, pale, anteriorly pale/posteriorly dark. Tegula and humeral complex color: tegula dark, humeral complex pale. Pterostigma color: dark with pale spot at base. Fore wing veins color: mostly dark (a few veins may be unpigmented). Antenna length/body length: antenna about as long as body (head to apex of metasoma); if slightly shorter, at least extending beyond anterior 0.7 metasoma length. Body in lateral view: not distinctly flattened dorso–ventrally. Body length (head to apex of metasoma): 2.0 mm or less. Fore wing length: 2.0 mm or less. Ocular–ocellar line/posterior ocellus diameter: 2.0–2.2. Interocellar distance/posterior ocellus diameter: 1.7–1.9. Antennal flagellomerus 2 length/width: 2.9–3.1. Antennal flagellomerus 14 length/width: 2.0–2.2. Length of flagellomerus 2/length of flagellomerus 14: 1.7–1.9. Tarsal claws: with single basal spine–like seta. Metafemur length/width: 3.0–3.1. Metatibia inner spur length/metabasitarsus length: 0.4–0.5. Anteromesoscutum: mostly with shallow, dense punctures (separated by less than 2.0 × its maximum diameter). Mesoscutellar disc: mostly smooth. Number of pits in scutoscutellar sulcus: 7 or 8. Maximum height of mesoscutellum lunules/maximum height of lateral face of mesoscutellum: 0.4–0.5. Propodeum areola: completely defined by carinae, including transverse carina extending to spiracle. Propodeum background sculpture: partly sculptured, especially on anterior 0.5. Mediotergite 1 length/width at posterior margin: 3.2–3.4. Mediotergite 1 shape: clearly narrowing towards posterior margin. Mediotergite 1 sculpture: with some sculpture near lateral margins and/or posterior 0.2–0.4 of mediotergite. Mediotergite 2 width at posterior margin/length: 3.6–3.9. Mediotergite 2 sculpture: mostly smooth. Outer margin of hypopygium: with a medially folded, transparent, semi–desclerotized area; with 0–3 pleats visible. Ovipositor thickness: anterior width 3.0–5.0 × posterior width (beyond ovipositor constriction). Ovipositor sheaths length/metatibial length: 0.4–0.5. Length of fore wing veins r/2RS: 1.0 or less. Length of fore wing veins 2RS/2M: 1.4–1.6. Length of fore wing veins 2M/(RS+M)b: 0.7–0.8. Pterostigma length/width: 2.6–3.0. Point of insertion of vein r in pterostigma: about half way point length of pterostigma. Angle of vein r with fore wing anterior margin: clearly outwards, inclined towards fore wing apex. Shape of junction of veins r and 2RS in fore wing: distinctly but not strongly angled.

**Male.** The only specimen available for study is in poor condition.

#### Molecular data.

Sequences in BOLD: 2, barcode compliant sequences: 2.

#### Biology/ecology.

Malaise Trapped.

#### Distribution.

Costa Rica, ACG.

#### Etymology.

We dedicate this species to María Guevara in recognition of her diligent efforts in assisting ACG operations of the Programa de Administracion.

### Apanteles marialuisariasae

Fernández-Triana sp. n.

http://zoobank.org/F0B62D8A-1F61-46CD-959B-434311FD5089

http://species-id.net/wiki/Apanteles_marialuisariasae

Figs 142 , 296

#### Type locality.

COSTA RICA, Alajuela, ACG, Sector San Cristobal, Bosque Trampa Malaise, 815m, 10.86280, -85.38460.

#### Holotype.

♀ in CNC. Specimen labels: 1. DHJPAR0027680. 2. San Gerardo: MT San Cristobal, Date: 11-17 Apr. 2008.

#### Description.

**Female.** Body color: body mostly dark except for some sternites which may be pale. Antenna color: scape, pedicel, and flagellum dark. Coxae color (pro-, meso-, metacoxa): dark, dark, dark. Femora color (pro-, meso-, metafemur): pale, pale, mostly pale but posterior 0.2 or less dark. Tibiae color (pro-, meso-, metatibia): pale, pale, mostly pale but with posterior 0.2 or less dark. Tegula and humeral complex color: tegula pale, humeral complex half pale/half dark. Pterostigma color: dark. Fore wing veins color: mostly dark (a few veins may be unpigmented). Antenna length/body length: antenna about as long as body (head to apex of metasoma); if slightly shorter, at least extending beyond anterior 0.7 metasoma length. Body in lateral view: not distinctly flattened dorso–ventrally. Body length (head to apex of metasoma): 2.9–3.0 mm. Fore wing length: 3.3–3.4 mm. Ocular–ocellar line/posterior ocellus diameter: 2.3–2.5. Interocellar distance/posterior ocellus diameter: 1.7–1.9. Antennal flagellomerus 2 length/width: 2.6–2.8. Antennal flagellomerus 14 length/width: 1.7–1.9. Length of flagellomerus 2/length of flagellomerus 14: 2.0–2.2. Tarsal claws: with single basal spine–like seta. Metafemur length/width: 3.4–3.5. Metatibia inner spur length/metabasitarsus length: 0.6–0.7. Anteromesoscutum: mostly with deep, dense punctures (separated by less than 2.0 × its maximum diameter). Mesoscutellar disc: mostly smooth. Number of pits in scutoscutellar sulcus: 9 or 10. Maximum height of mesoscutellum lunules/maximum height of lateral face of mesoscutellum: 0.4–0.5. Propodeum areola: completely defined by carinae, including transverse carina extending to spiracle. Propodeum background sculpture: partly sculptured, especially on anterior 0.5. Mediotergite 1 length/width at posterior margin: 1.4–1.6. Mediotergite 1 shape: more or less parallel–sided. Mediotergite 1 sculpture: mostly sculptured, excavated area centrally with transverse striation inside and/or a polished knob centrally on posterior margin of mediotergite. Mediotergite 2 width at posterior margin/length: 3.2–3.5. Mediotergite 2 sculpture: mostly smooth, with weak sculpture on anterior margin. Outer margin of hypopygium: with a medially folded, transparent, semi–desclerotized area; with 0–3 pleats visible. Ovipositor thickness: anterior width at most 2.0 × posterior width (beyond ovipositor constriction). Ovipositor sheaths length/metatibial length: 0.4–0.5. Length of fore wing veins r/2RS: 2.3 or more. Length of fore wing veins 2RS/2M: 1.4–1.6. Length of fore wing veins 2M/(RS+M)b: 0.5–0.6. Pterostigma length/width: 3.1–3.5. Point of insertion of vein r in pterostigma: clearly beyond half way point length of pterostigma. Angle of vein r with fore wing anterior margin: more or less perpendicular to fore wing margin. Shape of junction of veins r and 2RS in fore wing: distinctly but not strongly angled.

**Male.** Unknown.

#### Molecular data.

Sequences in BOLD: 2, barcode compliant sequences: 2.

#### Biology/ecology.

Solitary (Fig. 296). Host: Elachistidae, elachJanzen01 Janzen185.

#### Distribution.

Costa Rica, ACG.

#### Comments.

This species is characterized by hypopygium with a median folded, transparent, semi-desclerotized area with 1–3 pleats visible; ovipositor thin (thinner than width of median flagellomerus), and with basal width <2.0 × its apical width after constriction; T1 mostly sculptured and 1.4 × as long as wide at posterior margin; and body length 3.3 mm.

#### Etymology.

We dedicate this species to María Luisa Arias in recognition of her diligent efforts for the ACG Centro de Investigaciones y Estaciones.

### Apanteles mariamendezae

Fernández-Triana sp. n.

http://zoobank.org/C6E04933-50A4-44F8-BF49-A19A1220E953

http://species-id.net/wiki/Apanteles_mariamendezae

Fig. 143

#### Type locality.

COSTA RICA, Alajuela, ACG, Sector Pitilla, Estación Pitilla, 675m, 10.98931, -85.42581.

#### Holotype.

♀ in CNC. Specimen labels: 1. DHJPAR0042469. 2. COSTA RICA, Alajuela, ACG, Sector Pitilla, Estación Pitilla, 4.iii.2011, 10.98931°N, -85.42581°W, 675m, DHJPAR0042469. 3. Voucher: D.H.Janzen & W.Hallwachs, DB: http://janzen.sas.upenn.edu, Area de Conservación Guanacaste, COSTA RICA, 11-SRNP-30706.

#### Description.

**Female.** Body color: body mostly dark except for some sternites which may be pale. Antenna color: scape, pedicel, and flagellum dark. Coxae color (pro-, meso-, metacoxa): pale, dark, dark. Femora color (pro-, meso-, metafemur): pale, anteriorly dark/posteriorly pale, mostly dark but anterior 0.2 or less pale. Tibiae color (pro-, meso-, metatibia): pale, pale, anteriorly pale/posteriorly dark. Tegula and humeral complex color: tegula dark, humeral complex half pale/half dark. Pterostigma color: dark with pale spot at base. Fore wing veins color: mostly dark (a few veins may be unpigmented). Antenna length/body length: antenna shorter than body (head to apex of metasoma), not extending beyond anterior 0.7 metasoma length. Body in lateral view: not distinctly flattened dorso–ventrally. Body length (head to apex of metasoma): 3.1–3.2 mm. Fore wing length: 2.9–3.0 mm. Ocular–ocellar line/posterior ocellus diameter: 1.7–1.9. Interocellar distance/posterior ocellus diameter: 1.7–1.9. Antennal flagellomerus 2 length/width: 2.6–2.8. Antennal flagellomerus 14 length/width: 1.1–1.3. Length of flagellomerus 2/length of flagellomerus 14: 2.3–2.5. Tarsal claws: simple. Metafemur length/width: 3.2–3.3 or 3.4–3.5. Metatibia inner spur length/metabasitarsus length: 0.4–0.5. Anteromesoscutum: mostly with deep, dense punctures (separated by less than 2.0 × its maximum diameter). Mesoscutellar disc: mostly punctured. Number of pits in scutoscutellar sulcus: 7 or 8 or 9 or 10. Maximum height of mesoscutellum lunules/maximum height of lateral face of mesoscutellum: 0.4–0.5. Propodeum areola: completely defined by carinae, including transverse carina extending to spiracle. Propodeum background sculpture: partly sculptured, especially on anterior 0.5. Mediotergite 1 length/width at posterior margin: 4.1 or more. Mediotergite 1 shape: mostly parallel–sided for 0.5–0.7 of its length, then narrowing posteriorly so mediotergite anterior width >1.1 × posterior width. Mediotergite 1 sculpture: mostly sculptured, excavated area centrally with transverse striation inside and/or a polished knob centrally on posterior margin of mediotergite. Mediotergite 2 width at posterior margin/length: 3.6–3.9. Mediotergite 2 sculpture: with some sculpture, mostly near posterior margin. Outer margin of hypopygium: with a wide, medially folded, transparent, semi–desclerotized area; usually with 4 or more pleats. Ovipositor thickness: about same width throughout its length. Ovipositor sheaths length/metatibial length: 1.0–1.1. Length of fore wing veins r/2RS: 1.7–1.9. Length of fore wing veins 2RS/2M: 1.4–1.6. Length of fore wing veins 2M/(RS+M)b: 0.7–0.8. Pterostigma length/width: 2.6–3.0. Point of insertion of vein r in pterostigma: clearly beyond half way point length of pterostigma. Angle of vein r with fore wing anterior margin: clearly outwards, inclined towards fore wing apex. Shape of junction of veins r and 2RS in fore wing: distinctly but not strongly angled.

**Male.** Unknown.

#### Molecular data.

Sequences in BOLD: 1, barcode compliant sequences: 1.

#### Biology/ecology.

Solitary. Hosts: Elachistidae, elachJanzen01 10-SRNP-30313.

#### Distribution.

Costa Rica, ACG.

#### Comments.

This species is characterized by the combination of glossa elongate, pterostigma brown, and ovipositor about the same width from base to apex.

#### Etymology.

We dedicate this species to María Mendez in recognition of her efforts for the ACG Comedor in Santa Rosa.

### Apanteles marianopereirai

Fernández-Triana sp. n.

http://zoobank.org/5038A467-6A8A-4FC5-82E3-A0BFAFCA1584

http://species-id.net/wiki/Apanteles_marianopereirai

Fig. 62

Apanteles Rodriguez30 (Smith et al. 2006). Interim name provided by the authors.

#### Type locality.

COSTA RICA, Alajuela, ACG, Sector San Cristobal, Melina Bufalo, 560m, 10.88400, -85.38600.

#### Holotype.

♀ in CNC. Specimen labels: 1. COSTA RICA, Alajuela, ACG, Sector San Cristobal, Melina Bufalo, 01.iv.1998, 560m, 10.88400, -85.38600, DHJPAR0001565.

#### Paratypes.

20 ♀, 4 ♂ (BMNH, CNC, INBIO, INHS, NMNH). COSTA RICA: Alajuela, ACG database codes: 98-SRNP-6602.

#### Description.

**Female.** Body color: body mostly dark except for some sternites which may be pale. Antenna color: scape, pedicel, and flagellum dark or scape, pedicel, and flagellum pale. Coxae color (pro-, meso-, metacoxa): dark, dark, dark, rarely pale, dark, dark. Femora color (pro-, meso-, metafemur): anteriorly dark/posteriorly pale, dark, dark. Tibiae color (pro-, meso-, metatibia): pale, anteriorly pale/posteriorly dark, anteriorly pale/posteriorly dark. Tegula and humeral complex color: tegula pale, humeral complex half pale/half dark. Pterostigma color: mostly pale and/or transparent, with thin dark borders. Fore wing veins color: mostly white or entirely transparent. Antenna length/body length: antenna about as long as body (head to apex of metasoma); if slightly shorter, at least extending beyond anterior 0.7 metasoma length. Body in lateral view: not distinctly flattened dorso–ventrally. Body length (head to apex of metasoma): 2.3–2.4 mm, rarely 2.1–2.2 mm. Fore wing length: 2.3–2.4 mm or 2.5–2.6 mm. Ocular–ocellar line/posterior ocellus diameter: 2.6 or more. Interocellar distance/posterior ocellus diameter: 2.0–2.2. Antennal flagellomerus 2 length/width: 2.9–3.1. Antennal flagellomerus 14 length/width: 1.7–1.9. Length of flagellomerus 2/length of flagellomerus 14: 1.7–1.9. Tarsal claws: with single basal spine–like seta. Metafemur length/width: 3.4–3.5. Metatibia inner spur length/metabasitarsus length: 0.6–0.7. Anteromesoscutum: mostly with deep, dense punctures (separated by less than 2.0 × its maximum diameter). Mesoscutellar disc: with punctures near margins, central part mostly smooth. Number of pits in scutoscutellar sulcus: 5 or 6 or 7 or 8. Maximum height of mesoscutellum lunules/maximum height of lateral face of mesoscutellum: 0.4–0.5. Propodeum areola: completely defined by carinae, including transverse carina extending to spiracle. Propodeum background sculpture: partly sculptured, especially on anterior 0.5. Mediotergite 1 length/width at posterior margin: 3.2–3.4. Mediotergite 1 shape: mostly parallel–sided for 0.5–0.7 of its length, then narrowing posteriorly so mediotergite anterior width >1.1 × posterior width. Mediotergite 1 sculpture: with some sculpture near lateral margins and/or posterior 0.2–0.4 of mediotergite. Mediotergite 2 width at posterior margin/length: 3.6–3.9. Mediotergite 2 sculpture: mostly smooth. Outer margin of hypopygium: inflexible (without a folded, transparent, semi–desclerotized area); with no pleats visible. Ovipositor thickness: anterior width at most 2.0 × posterior width (beyond ovipositor constriction) or anterior width 3.0–5.0 × posterior width (beyond ovipositor constriction) (?). Ovipositor sheaths length/metatibial length: 0.6–0.7 or 0.8–0.9. Length of fore wing veins r/2RS: 1.7–1.9. Length of fore wing veins 2RS/2M: 1.1–1.3. Length of fore wing veins 2M/(RS+M)b: 0.7–0.8. Pterostigma length/width: 3.6 or more. Point of insertion of vein r in pterostigma: about half way point length of pterostigma. Angle of vein r with fore wing anterior margin: more or less perpendicular to fore wing margin. Shape of junction of veins r and 2RS in fore wing: distinctly but not strongly angled.

**Male.** Similar to female.

#### Molecular data.

Sequences in BOLD: 1, barcode compliant sequences: 0 (the only available sequence is 419 bp).

#### Biology/ecology.

Gregarious. Host: Hesperiidae, *Polythrix kanshul*.

#### Distribution.

Costa Rica, ACG.

#### Comments.

Despite the single lot of specimens available (98-SRNP-6601 was parasitized by the same species but rearing maltreatment killed the wasps in their cocoons), the distinctive wasp morphology, distinctive host, and distinctive, though short, barcode make it clear that this is a species different from all others considered here.

#### Etymology.

We dedicate this species to Mariano Pereira recognition of his diligent efforts for the ACG Programa de Parataxónomos and Estación Biológica La Perla of Sector Mundo Nuevo.

### Apanteles mariatorrentesae

Fernández-Triana sp. n.

http://zoobank.org/7E27B624-A30F-4C34-84BA-FEA0C36748E3

http://species-id.net/wiki/Apanteles_mariatorrentesae

Fig. 29

#### Type locality.

COSTA RICA, Alajuela, ACG, Sector Rincon Rain Forest, Puente Rio Negro, 340m, 10.90376, -85.30274.

#### Holotype.

♀ in CNC. Specimen labels: 1. DHJPAR0043137. 2. COSTA RICA, Guanacaste, ACG, Sector Rincon Rain Forest, Puente Rio Negro, 27.iii.2011, 10.90376, -85.30274, 340m, DHJPAR0043137. 3. Voucher: D.H.Janzen & W.Hallwachs, DB: http://janzen.sas.upenn.edu, Area de Conservación Guanacaste, COSTA RICA, 11-SRNP-41455.

#### Paratypes.

1 ♀ (NMNH). COSTA RICA, ACG database codes: DHJPAR0043139.

#### Description.

**Female.** Body color: body mostly dark except for some sternites which may be pale. Antenna color: scape, pedicel, and flagellum dark. Coxae color (pro-, meso-, metacoxa): dark, dark, dark. Femora color (pro-, meso-, metafemur): anteriorly dark/posteriorly pale, dark, dark. Tibiae color (pro-, meso-, metatibia): pale, pale, anteriorly pale/posteriorly dark. Tegula and humeral complex color: tegula pale, humeral complex half pale/half dark. Pterostigma color: mostly pale and/or transparent, with thin dark borders. Fore wing veins color: mostly white or entirely transparent. Antenna length/body length: antenna about as long as body (head to apex of metasoma); if slightly shorter, at least extending beyond anterior 0.7 metasoma length. Body in lateral view: not distinctly flattened dorso–ventrally. Body length (head to apex of metasoma): 2.5–2.6 mm. Fore wing length: 2.7–2.8 mm. Ocular–ocellar line/posterior ocellus diameter: 2.0–2.2. Interocellar distance/posterior ocellus diameter: 2.0–2.2. Antennal flagellomerus 2 length/width: 2.9–3.1. Antennal flagellomerus 14 length/width: 1.4–1.6. Length of flagellomerus 2/length of flagellomerus 14: 2.0–2.2. Tarsal claws: with single basal spine–like seta. Metafemur length/width: 3.2–3.3. Metatibia inner spur length/metabasitarsus length: 0.4–0.5. Anteromesoscutum: mostly with deep, dense punctures (separated by less than 2.0 × its maximum diameter). Mesoscutellar disc: with a few sparse punctures. Number of pits in scutoscutellar sulcus: 11 or 12. Maximum height of mesoscutellum lunules/maximum height of lateral face of mesoscutellum: 0.6–0.7. Propodeum areola: completely defined by carinae, including transverse carina extending to spiracle. Propodeum background sculpture: partly sculptured, especially on anterior 0.5. Mediotergite 1 length/width at posterior margin: 2.0–2.2. Mediotergite 1 shape: mostly parallel–sided for 0.5–0.7 of its length, then narrowing posteriorly so mediotergite anterior width >1.1 × posterior width. Mediotergite 1 sculpture: mostly sculptured, excavated area centrally with transverse striation inside and/or a polished knob centrally on posterior margin of mediotergite. Mediotergite 2 width at posterior margin/length: 3.6–3.9. Mediotergite 2 sculpture: mostly smooth. Outer margin of hypopygium: with a wide, medially folded, transparent, semi–desclerotized area; usually with 4 or more pleats. Ovipositor thickness: about same width throughout its length. Ovipositor sheaths length/metatibial length: 1.0–1.1. Length of fore wing veins r/2RS: 2.3 or more. Length of fore wing veins 2RS/2M: 1.4–1.6. Length of fore wing veins 2M/(RS+M)b: 0.5–0.6. Pterostigma length/width: 3.1–3.5. Point of insertion of vein r in pterostigma: about half way point length of pterostigma. Angle of vein r with fore wing anterior margin: more or less perpendicular to fore wing margin. Shape of junction of veins r and 2RS in fore wing: distinctly but not strongly angled.

**Male.** Unknown.

#### Molecular data.

Sequences in BOLD: 6, barcode compliant sequences: 6.

#### Biology/ecology.

Solitary. Hosts: Crambidae, *Leucochromodes* BioLep314, *Asturodes fimbriauralis* DHJ01.

#### Distribution.

Costa Rica, ACG.

#### Etymology.

We dedicate this species to María Torrentes in recognition of her diligent efforts for the ACG Programa del Comedor Santa Rosa.

### Apanteles marisolarroyoae

Fernández-Triana sp. n.

http://zoobank.org/3ADA9966-C370-47E8-BE2F-86B46BB67B95

http://species-id.net/wiki/Apanteles_marisolarroyoae

Figs 86 , 265

Apanteles Rodriguez170. Smith et al. (2008). Interim name provided by the authors.

#### Type locality.

COSTA RICA, Alajuela, ACG, Sector Rincon Rain Forest, Camino Albergue Oscar, 560m, 10.87741, -85.32363.

#### Holotype.

♀ in CNC. Specimen labels: 1. Costa Rica: Alajuela, ACG, Sector Rincon Rain Forest, Puente Rio Negro, 21.iv.2010, 340m, 10.90376, -85.30274, 10-SRNP-41503.

#### Paratypes.

7 ♀ (BMNH, CNC, INBIO, INHS, NMNH). COSTA RICA, ACG database codes: 10-SRNP-41503.

#### Description.

**Female.** Body color: body mostly dark except for some sternites which may be pale. Antenna color: scape, pedicel, and flagellum dark. Coxae color (pro-, meso-, metacoxa): dark, dark, dark. Femora color (pro-, meso-, metafemur): anteriorly dark/posteriorly pale, dark, dark. Tibiae color (pro-, meso-, metatibia): pale, pale, mostly dark but anterior 0.2 or less pale. Tegula and humeral complex color: tegula pale, humeral complex half pale/half dark. Pterostigma color: mostly pale and/or transparent, with thin dark borders. Fore wing veins color: partially pigmented (a few veins may be dark but most are pale). Antenna length/body length: antenna shorter than body (head to apex of metasoma), not extending beyond anterior 0.7 metasoma length. Body in lateral view: not distinctly flattened dorso–ventrally. Body length (head to apex of metasoma): 3.1–3.2 mm or 3.3–3.4 mm. Fore wing length: 3.1–3.2 mm or 3.3–3.4 mm. Ocular–ocellar line/posterior ocellus diameter: 2.3–2.5. Interocellar distance/posterior ocellus diameter: 1.7–1.9. Antennal flagellomerus 2 length/width: 2.6–2.8. Antennal flagellomerus 14 length/width: 1.1–1.3. Length of flagellomerus 2/length of flagellomerus 14: 2.3–2.5. Tarsal claws: with single basal spine–like seta. Metafemur length/width: 3.2–3.3. Metatibia inner spur length/metabasitarsus length: 0.6–0.7. Anteromesoscutum: mostly with deep, dense punctures (separated by less than 2.0 × its maximum diameter). Mesoscutellar disc: with punctures near margins, central part mostly smooth. Number of pits in scutoscutellar sulcus: 7 or 8. Maximum height of mesoscutellum lunules/maximum height of lateral face of mesoscutellum: 0.4–0.5. Propodeum areola: completely defined by carinae, including transverse carina extending to spiracle. Propodeum background sculpture: mostly sculptured. Mediotergite 1 length/width at posterior margin: 2.3–2.5. Mediotergite 1 shape: mostly parallel–sided for 0.5–0.7 of its length, then narrowing posteriorly so mediotergite anterior width >1.1 × posterior width. Mediotergite 1 sculpture: mostly sculptured, excavated area centrally with transverse striation inside and/or a polished knob centrally on posterior margin of mediotergite. Mediotergite 2 width at posterior margin/length: 2.4–2.7. Mediotergite 2 sculpture: with some sculpture, mostly near posterior margin. Outer margin of hypopygium: with a wide, medially folded, transparent, semi–desclerotized area; usually with 4 or more pleats. Ovipositor thickness: about same width throughout its length. Ovipositor sheaths length/metatibial length: 1.4–1.5. Length of fore wing veins r/2RS: 1.7–1.9. Length of fore wing veins 2RS/2M: 1.1–1.3. Length of fore wing veins 2M/(RS+M)b: 0.7–0.8. Pterostigma length/width: 3.6 or more. Point of insertion of vein r in pterostigma: clearly beyond half way point length of pterostigma. Angle of vein r with fore wing anterior margin: clearly outwards, inclined towards fore wing apex. Shape of junction of veins r and 2RS in fore wing: distinctly but not strongly angled, rarely evenly curved.

**Male.** Unknown.

#### Molecular data.

Sequences in BOLD: 4, barcode compliant sequences: 2.

#### Biology/ecology.

Solitary (Fig. 265). Hosts: Elachistidae, three undetermined species.

#### Distribution.

Costa Rica, ACG.

#### Comments.

This species is placed in the *bienvenidachavarriae* species-group based on morphological and host similarities (barcoding clusters this species apart from the other two).

#### Etymology.

We dedicate this species to Marisol Arroyo for her diligent efforts for the ACG Sector Marino.

### Apanteles marisolnavarroae

Fernández-Triana sp. n.

http://zoobank.org/CC9289B5-ADCA-4A4F-91C5-7A4E45C9FBD6

http://species-id.net/wiki/Apanteles_marisolnavarroae

Figs 144 , 297

#### Type locality.

COSTA RICA, Guanacaste, ACG, Sector San Cristobal, Tajo Angeles, 540m, 10.86472, -85.41531.

#### Holotype.

♀ in CNC. Specimen labels: 1. DHJPAR0041984. 2. COSTA RICA, Guanacaste, ACG, Sector San Cristobal, Tajo Angeles, 10.86472°N, -85.41531°W, 540m, DHJPAR0041984. 3. Voucher: D.H.Janzen & W.Hallwachs, DB: http://janzen.sas.upenn.edu, Area de Conservación Guanacaste, COSTA RICA, 10-SRNP-6737.

#### Paratypes.

1 ♂ (CNC). COSTA RICA: Guanacaste, ACG database code: DHJPAR0039474.

#### Description.

**Female.** Body color: body mostly dark except for some sternites which may be pale. Antenna color: scape, pedicel, and flagellum dark. Coxae color (pro-, meso-, metacoxa): dark, dark, dark. Femora color (pro-, meso-, metafemur): pale, dark, dark. Tibiae color (pro-, meso-, metatibia): pale, pale, anteriorly pale/posteriorly dark. Tegula and humeral complex color: tegula pale, humeral complex half pale/half dark. Pterostigma color: dark with pale spot at base. Fore wing veins color: mostly dark (a few veins may be unpigmented). Antenna length/body length: antenna shorter than body (head to apex of metasoma), not extending beyond anterior 0.7 metasoma length. Body in lateral view: not distinctly flattened dorso–ventrally. Body length (head to apex of metasoma): 3.7–3.8 mm. Fore wing length: 3.5–3.6 mm. Ocular–ocellar line/posterior ocellus diameter: 2.0–2.2. Interocellar distance/posterior ocellus diameter: 1.7–1.9. Antennal flagellomerus 2 length/width: 3.2 or more. Antennal flagellomerus 14 length/width: 1.4–1.6. Length of flagellomerus 2/length of flagellomerus 14: 2.3–2.5. Tarsal claws: with single basal spine–like seta. Metafemur length/width: 3.2–3.3. Metatibia inner spur length/metabasitarsus length: 0.4–0.5. Anteromesoscutum: mostly with deep, dense punctures (separated by less than 2.0 × its maximum diameter). Mesoscutellar disc: mostly punctured. Number of pits in scutoscutellar sulcus: 9 or 10. Maximum height of mesoscutellum lunules/maximum height of lateral face of mesoscutellum: 0.4–0.5. Propodeum areola: completely defined by carinae, including transverse carina extending to spiracle. Propodeum background sculpture: partly sculptured, especially on anterior 0.5. Mediotergite 1 length/width at posterior margin: 2.3–2.5. Mediotergite 1 shape: mostly parallel–sided for 0.5–0.7 of its length, then narrowing posteriorly so mediotergite anterior width >1.1 × posterior width. Mediotergite 1 sculpture: mostly sculptured, excavated area centrally with transverse striation inside and/or a polished knob centrally on posterior margin of mediotergite. Mediotergite 2 width at posterior margin/length: 2.8–3.1. Mediotergite 2 sculpture: mostly smooth. Outer margin of hypopygium: with a wide, medially folded, transparent, semi–desclerotized area; usually with 4 or more pleats. Ovipositor thickness: about same width throughout its length. Ovipositor sheaths length/metatibial length: 1.0–1.1. Length of fore wing veins r/2RS: 1.7–1.9. Length of fore wing veins 2RS/2M: 1.4–1.6. Length of fore wing veins 2M/(RS+M)b: 0.5–0.6. Pterostigma length/width: 2.6–3.0. Point of insertion of vein r in pterostigma: clearly beyond half way point length of pterostigma. Angle of vein r with fore wing anterior margin: clearly outwards, inclined towards fore wing apex. Shape of junction of veins r and 2RS in fore wing: distinctly but not strongly angled.

**Male.** As in female, but pterostigma is mostly transparent, with thin brown borders.

#### Molecular data.

Sequences in BOLD: 2, barcode compliant sequences: 2.

#### Biology/ecology.

Solitary (Fig. 297). Hosts: Pyralidae, epipaJanzen01 Janzen18, epipaJanzen01 Janzen178.

#### Distribution.

Costa Rica, ACG.

#### Etymology.

We dedicate this species to Marisol Navarro in recognition of her diligent efforts for the ACG Sector Marino.

### Apanteles marvinmendozai

Fernández-Triana sp. n.

http://zoobank.org/CD48D952-8F05-4D35-88B4-5D384B9883C7

http://species-id.net/wiki/Apanteles_marvinmendozai

Figs 48 , 241

#### Type locality.

COSTA RICA, Guanacaste, ACG, Sector San Cristobal, Tajo Angeles, 540m, 10.86472, -85.41531.

#### Holotype.

♀ in CNC. Specimen labels: 1. DHJPAR0041608. 2. COSTA RICA, Guanacaste, ACG, Sector San Cristobal, Tajo Angeles, 25.x.2010, 10.86472 N, -85.41531 W, 540m, DHJPAR0041608. 3. Voucher: D.H.Janzen & W.Hallwachs, DB: http://janzen.sas.upenn.edu, Area de Conservación Guanacaste, COSTA RICA, 10-SRNP-6252.

#### Description.

**Female.** Body color: body mostly dark except for some sternites which may be pale. Antenna color: scape, pedicel, and flagellum dark. Coxae color (pro-, meso-, metacoxa): dark, dark, dark. Femora color (pro-, meso-, metafemur): anteriorly dark/posteriorly pale, dark, dark. Tibiae color (pro-, meso-, metatibia): pale, pale, anteriorly pale/posteriorly dark. Tegula and humeral complex color: tegula pale, humeral complex half pale/half dark. Pterostigma color: mostly pale and/or transparent, with thin dark borders. Fore wing veins color: partially pigmented (a few veins may be dark but most are pale). Antenna length/body length: antenna about as long as body (head to apex of metasoma); if slightly shorter, at least extending beyond anterior 0.7 metasoma length. Body in lateral view: not distinctly flattened dorso–ventrally. Body length (head to apex of metasoma): 3.3–3.4 mm. Fore wing length: 3.3–3.4 mm. Ocular–ocellar line/posterior ocellus diameter: 2.6 or more. Interocellar distance/posterior ocellus diameter: 2.0–2.2. Antennal flagellomerus 2 length/width: 2.6–2.8. Antennal flagellomerus 14 length/width: 1.4–1.6. Length of flagellomerus 2/length of flagellomerus 14: 2.0–2.2. Tarsal claws: with single basal spine–like seta. Metafemur length/width: 3.0–3.1. Metatibia inner spur length/metabasitarsus length: 0.4–0.5. Anteromesoscutum: mostly with deep, dense punctures (separated by less than 2.0 × its maximum diameter). Mesoscutellar disc: mostly smooth. Number of pits in scutoscutellar sulcus: 11 or 12. Maximum height of mesoscutellum lunules/maximum height of lateral face of mesoscutellum: 0.6–0.7. Propodeum areola: completely defined by carinae, including transverse carina extending to spiracle. Propodeum background sculpture: mostly sculptured. Mediotergite 1 length/width at posterior margin: 2.3–2.5. Mediotergite 1 shape: more or less parallel–sided. Mediotergite 1 sculpture: mostly sculptured, excavated area centrally with transverse striation inside and/or a polished knob centrally on posterior margin of mediotergite. Mediotergite 2 width at posterior margin/length: 1.6–1.9. Mediotergite 2 sculpture: mostly smooth. Outer margin of hypopygium: with a wide, medially folded, transparent, semi–desclerotized area; usually with 4 or more pleats. Ovipositor thickness: about same width throughout its length. Ovipositor sheaths length/metatibial length: 1.8–1.9. Length of fore wing veins r/2RS: 2.3 or more. Length of fore wing veins 2RS/2M: 1.1–1.3. Length of fore wing veins 2M/(RS+M)b: 0.7–0.8. Pterostigma length/width: 3.1–3.5. Point of insertion of vein r in pterostigma: clearly beyond half way point length of pterostigma. Angle of vein r with fore wing anterior margin: clearly outwards, inclined towards fore wing apex. Shape of junction of veins r and 2RS in fore wing: distinctly but not strongly angled.

**Male.** Unknown.

#### Molecular data.

Sequences in BOLD: 3, barcode compliant sequences: 3.

#### Biology/ecology.

Solitary (Fig. 241). Hosts: Elachistidae, three species of *Antaeotricha*.

#### Distribution.

Costa Rica, ACG.

#### Etymology.

We dedicate this species to Marvin Mendoza in recognition of his diligent efforts as and ACG driver for all Programs.

### Apanteles mauriciogurdiani

Fernández-Triana sp. n.

http://zoobank.org/BDC3DD70-A3FD-497A-A305-C3739FAAAEBB

http://species-id.net/wiki/Apanteles_mauriciogurdiani

Figs 70 , 260

#### Type locality.

COSTA RICA, Alajuela, ACG, Sector Rincon Rain Forest, San Lucas, 320m, 10.91847, -85.30338.

#### Holotype.

♀ in CNC. Specimen labels: 1. DHJPAR0041802. 2. COSTA RICA, Alajuela, ACG, Sector Rincon Rain Forest, San Lucas, 28.xi.2010, 10.91847°N, -85.30338°W, 320m, DHJPAR0041802.

#### Paratypes.

16 ♀ (BMNH, CNC, INBIO, INHS, NMNH). COSTA RICA: Guanacaste, ACG database code: DHJPAR0041802.

#### Description.

**Female.** Body color: body mostly dark except for some sternites which may be pale. Antenna color: scape, pedicel, and flagellum dark. Coxae color (pro-, meso-, metacoxa): pale, dark, dark. Femora color (pro-, meso-, metafemur): pale, pale, dark. Tibiae color (pro-, meso-, metatibia): pale, pale, anteriorly pale/posteriorly dark. Tegula and humeral complex color: tegula pale, humeral complex half pale/half dark. Pterostigma color: dark with pale spot at base. Fore wing veins color: mostly dark (a few veins may be unpigmented). Antenna length/body length: antenna about as long as body (head to apex of metasoma); if slightly shorter, at least extending beyond anterior 0.7 metasoma length. Body in lateral view: not distinctly flattened dorso–ventrally. Body length (head to apex of metasoma): 2.3–2.4 mm or 2.5–2.6 mm. Fore wing length: 2.5–2.6 mm or 2.7–2.8 mm. Ocular–ocellar line/posterior ocellus diameter: 2.0–2.2. Interocellar distance/posterior ocellus diameter: 1.4–1.6. Antennal flagellomerus 2 length/width: 2.9–3.1. Antennal flagellomerus 14 length/width: 1.7–1.9. Length of flagellomerus 2/length of flagellomerus 14: 2.0–2.2. Tarsal claws: with single basal spine–like seta or with two basal spine–like setae (?). Metafemur length/width: 3.4–3.5. Metatibia inner spur length/metabasitarsus length: 0.4–0.5. Anteromesoscutum: mostly with deep, dense punctures (separated by less than 2.0 × its maximum diameter). Mesoscutellar disc: mostly smooth. Number of pits in scutoscutellar sulcus: 5 or 6 or 7 or 8. Maximum height of mesoscutellum lunules/maximum height of lateral face of mesoscutellum: 0.2–0.3. Propodeum areola: completely defined by carinae, including transverse carina extending to spiracle. Propodeum background sculpture: partly sculptured, especially on anterior 0.5. Mediotergite 1 length/width at posterior margin: 2.3–2.5. Mediotergite 1 shape: slightly widening from anterior margin to 0.7–0.8 mediotergite length (where maximum width is reached), then narrowing towards posterior margin. Mediotergite 1 sculpture: mostly sculptured, excavated area centrally with transverse striation inside and/or a polished knob centrally on posterior margin of mediotergite. Mediotergite 2 width at posterior margin/length: 3.2–3.5. Mediotergite 2 sculpture: mostly smooth, with weak sculpture on anterior margin. Outer margin of hypopygium: with a wide, medially folded, transparent, semi–desclerotized area; usually with 4 or more pleats. Ovipositor thickness: about same width throughout its length. Ovipositor sheaths length/metatibial length: 1.4–1.5. Length of fore wing veins r/2RS: 1.4–1.6. Length of fore wing veins 2RS/2M: 1.4–1.6. Length of fore wing veins 2M/(RS+M)b: 0.9–1.0. Pterostigma length/width: 3.6 or more. Point of insertion of vein r in pterostigma: clearly beyond half way point length of pterostigma. Angle of vein r with fore wing anterior margin: clearly outwards, inclined towards fore wing apex. Shape of junction of veins r and 2RS in fore wing: distinctly but not strongly angled.

**Male.** Unknown.

#### Molecular data.

Sequences in BOLD: 1, barcode compliant sequences: 1.

#### Biology/ecology.

Gregarious (Fig. 260). Host: Elachistidae, elachJanzen01 Janzen764.

#### Distribution.

Costa Rica, ACG.

#### Etymology.

We dedicate this species to Mauricio Gurdián in recognition of his diligent efforts for the ACG Programa de Contabilidad.

### Apanteles megastidis

Muesebeck, 1958

http://species-id.net/wiki/Apanteles_megastidis

Fig. 151

Apanteles megastidis Muesebeck, 1958: 445.

#### Type locality.

TRINIDAD: St. Augustine.

#### Holotype.

♀, NMNH (examined).

#### Description.

**Female.** Body color: body mostly dark except for some sternites which may be pale. Antenna color: scape, pedicel, and flagellum dark. Coxae color (pro-, meso-, metacoxa): dark, dark, dark. Femora color (pro-, meso-, metafemur): pale, pale, anteriorly pale/posteriorly dark. Tibiae color (pro-, meso-, metatibia): pale, pale, mostly pale but with posterior 0.2 or less dark. Tegula and humeral complex color: both pale. Pterostigma color: mostly pale and/or transparent, with thin dark borders. Fore wing veins color: mostly white or entirely transparent. Antenna length/body length: antenna about as long as body (head to apex of metasoma); if slightly shorter, at least extending beyond anterior 0.7 metasoma length. Body in lateral view: not distinctly flattened dorso–ventrally. Body length (head to apex of metasoma): 3.7–3.8 mm. Fore wing length: 4.0 mm or more. Ocular–ocellar line/posterior ocellus diameter: 2.0–2.2. Interocellar distance/posterior ocellus diameter: 1.7–1.9. Antennal flagellomerus 2 length/width: 2.9–3.1. Antennal flagellomerus 14 length/width: 1.4–1.6. Length of flagellomerus 2/length of flagellomerus 14: 2.0–2.2. Tarsal claws: simple. Metafemur length/width: 3.2–3.3. Metatibia inner spur length/metabasitarsus length: 0.4–0.5. Anteromesoscutum: mostly with deep, dense punctures (separated by less than 2.0 × its maximum diameter). Mesoscutellar disc: mostly smooth. Number of pits in scutoscutellar sulcus: 13 or 14. Maximum height of mesoscutellum lunules/maximum height of lateral face of mesoscutellum: 0.8 or more. Propodeum areola: completely defined by carinae, including transverse carina extending to spiracle. Propodeum background sculpture: mostly sculptured. Mediotergite 1 length/width at posterior margin: 1.1–1.3. Mediotergite 1 shape: more or less parallel–sided. Mediotergite 1 sculpture: mostly sculptured, excavated area centrally with transverse striation inside and/or a polished knob centrally on posterior margin of mediotergite. Mediotergite 2 width at posterior margin/length: 2.8–3.1. Mediotergite 2 sculpture: mostly smooth. Outer margin of hypopygium: with a wide, medially folded, transparent, semi–desclerotized area; usually with 4 or more pleats. Ovipositor thickness: about same width throughout its length. Ovipositor sheaths length/metatibial length: 1.4–1.5. Length of fore wing veins r/2RS: 1.4–1.6. Length of fore wing veins 2RS/2M: 1.7–1.8. Length of fore wing veins 2M/(RS+M)b: 0.5–0.6. Pterostigma length/width: 2.6–3.0. Point of insertion of vein r in pterostigma: clearly beyond half way point length of pterostigma. Angle of vein r with fore wing anterior margin: clearly outwards, inclined towards fore wing apex. Shape of junction of veins r and 2RS in fore wing: distinctly but not strongly angled.

**Male.** Esentially like female (Muesebeck 1958).

#### Molecular data.

No molecular data available for this species.

#### Biology/ecology.

Solitary, white cocoon about 6-7 mm long. Host: Crambidae, *Megastes* sp.

#### Distribution.

Trinidad and Tobago. There is no suggestion that this species occurs in ACG.

#### Comments.

This species is only known from the specimens studied by Muesebeck (1958) when describing the species.

### Apanteles megathymi

Riley, 1881

http://species-id.net/wiki/Apanteles_megathymi

Fig. 147

Apanteles megathymi Riley, 1881: 304.

#### Type locality.

UNITED STATES: “South Carolina”, locality not specified.

#### Holotype.

A series of 7 male specimens considered as cotypes, NMNH (examined).

#### Material Examined.

2 ♀, 2 ♂ (CNC). UNITED STATES: CA, San Diego, San Felipe Valley, ix-1938, W. P. Medlar, ex *Agathymus stephensi*.

#### Description.

**Female.** Body color: body mostly dark except for some sternites which may be pale. Antenna color: scape, pedicel, and flagellum dark. Coxae color (pro-, meso-, metacoxa): dark, dark, dark. Femora color (pro-, meso-, metafemur): pale, anteriorly dark/posteriorly pale, mostly dark but centrally pale. Tibiae color (pro-, meso-, metatibia): pale, pale, mostly pale but with posterior 0.2 or less dark. Tegula and humeral complex color: tegula pale, humeral complex half pale/half dark. Pterostigma color: mostly pale and/or transparent, with thin dark borders. Fore wing veins color: partially pigmented (a few veins may be dark but most are pale). Antenna length/body length: antenna about as long as body (head to apex of metasoma); if slightly shorter, at least extending beyond anterior 0.7 metasoma length. Body in lateral view: not distinctly flattened dorso–ventrally. Body length (head to apex of metasoma): 3.5–3.6 mm. Fore wing length: 3.7–3.8 mm. Ocular–ocellar line/posterior ocellus diameter: 1.4–1.6. Interocellar distance/posterior ocellus diameter: 1.7–1.9. Antennal flagellomerus 2 length/width: 2.6–2.8. Antennal flagellomerus 14 length/width: 2.0–2.2. Length of flagellomerus 2/length of flagellomerus 14: 1.7–1.9. Tarsal claws: simple. Metafemur length/width: 3.2–3.3. Metatibia inner spur length/metabasitarsus length: 0.4–0.5. Anteromesoscutum: mostly with deep, dense punctures (separated by less than 2.0 × its maximum diameter). Mesoscutellar disc: with a few sparse punctures. Number of pits in scutoscutellar sulcus: 7 or 8. Maximum height of mesoscutellum lunules/maximum height of lateral face of mesoscutellum: 0.6–0.7. Propodeum areola: completely defined by carinae, including transverse carina extending to spiracle. Propodeum background sculpture: mostly sculptured. Mediotergite 1 length/width at posterior margin: 2.3–2.5 or 2.6–2.8. Mediotergite 1 shape: more or less parallel–sided. Mediotergite 1 sculpture: mostly sculptured, excavated area centrally with transverse striation inside and/or a polished knob centrally on posterior margin of mediotergite. Mediotergite 2 width at posterior margin/length: 2.8–3.1. Mediotergite 2 sculpture: mostly smooth. Outer margin of hypopygium: with a wide, medially folded, transparent, semi–desclerotized area; usually with 4 or more pleats. Ovipositor thickness: about same width throughout its length. Ovipositor sheaths length/metatibial length: 1.4–1.5. Length of fore wing veins r/2RS: 1.0 or less. Length of fore wing veins 2RS/2M: 1.4–1.6. Length of fore wing veins 2M/(RS+M)b: 0.7–0.8. Pterostigma length/width: 2.6–3.0. Point of insertion of vein r in pterostigma: clearly beyond half way point length of pterostigma. Angle of vein r with fore wing anterior margin: clearly outwards, inclined towards fore wing apex. Shape of junction of veins r and 2RS in fore wing: strongly angulated, sometimes with a knob.

**Male.** The vein r in the fore wing tends to be longer, surpassing the length of vein 2RS. The mediotergite 2 is more trapezoidal (i.e., the ratio of its width at apex/medial length is lower than in females). The metafemur is fully dark brown to black.

#### Molecular data.

No molecular data available for this species.

#### Biology/ecology.

Gregarious, cocoons packed close together in the burrow of its stem-mining host (Muesebeck 1921). Hosts: Hesperiidae (*Agathymus stephensi*, *Megathymus colouradensis*, *Megathymus comstocki*, *Megathymus ursus*, *Megathymus yucae*).

#### Distribution.

Mexico, United States (AZ, CA, NC, SC). While Asparagaceae (formerly Agavaceae) does occur in Costa Rica and ACG, there is no suggestion that this species or its host caterpillars occur in Costa Rica or ACG.

#### Comments.

The description provided was mostly based on two female specimens from California deposited in the CNC. They were identified by Muesebeck after comparing with the type material. The specimens match well the short descriptions provided in previous papers (e.g., Riley 1881; Muesebeck 1921).

### Apanteles milenagutierrezae

Fernández-Triana sp. n.

http://zoobank.org/1B7973DB-A471-4457-BB58-D3D298359949

http://species-id.net/wiki/Apanteles_milenagutierrezae

Figs 116 , 282

#### Type locality.

COSTA RICA, Guanacaste, ACG, Sector Pitilla, Pasmompa, 440m, 11.01926, -85.40997.

#### Holotype.

♀ in CNC. Specimen labels: 1. Voucher: D.H.Janzen & W.Hallwachs, DB: http://janzen.sas.upenn.edu, Area de Conservación Guanacaste, COSTA RICA, 10-SRNP-30844. 2. DHJPAR0039048.

#### Paratypes.

13 ♀, 1#M (BMNH, CNC, INBIO, INHS, NMNH). COSTA RICA, ACG database codes: DHJPAR0039040, DHJPAR0039042, DHJPAR0039044, DHJPAR0039050, DHJPAR0039053, DHJPAR0039060, DHJPAR0039068, DHJPAR0039087, DHJPAR0039088, DHJPAR0039093, DHJPAR0039096, DHJPAR0039103, DHJPAR0039113, DHJPAR0039735.

#### Description.

**Female.** Body color: head dark, mesosoma dark with parts of axillar complex pale, metasoma with some mediotergites, most laterotergites, sternites, and/or hypopygium pale. Antenna color: scape, pedicel, and flagellum dark. Coxae color (pro-, meso-, metacoxa): pale, pale, pale. Femora color (pro-, meso-, metafemur): pale, pale, mostly dark but with pale spot antero–ventrally. Tibiae color (pro-, meso-, metatibia): pale, pale, anteriorly pale/posteriorly dark. Tegula and humeral complex color: both pale. Pterostigma color: dark. Fore wing veins color: mostly dark (a few veins may be unpigmented). Antenna length/body length: antenna shorter than body (head to apex of metasoma), not extending beyond anterior 0.7 metasoma length. Body in lateral view: not distinctly flattened dorso–ventrally. Body length (head to apex of metasoma): 3.5–3.6 mm, 3.7–3.8 mm, rarely 3.9–4.0 mm. Fore wing length: 3.5–3.6 mm. Ocular–ocellar line/posterior ocellus diameter: 2.3–2.5. Interocellar distance/posterior ocellus diameter: 2.0–2.2. Antennal flagellomerus 2 length/width: 2.3–2.5. Antennal flagellomerus 14 length/width: 1.4–1.6. Length of flagellomerus 2/length of flagellomerus 14: 2.0–2.2. Tarsal claws: with single basal spine–like seta. Metafemur length/width: 3.0–3.1. Metatibia inner spur length/metabasitarsus length: 0.4–0.5. Anteromesoscutum: mostly with deep, dense punctures (separated by less than 2.0 × its maximum diameter). Mesoscutellar disc: mostly punctured. Number of pits in scutoscutellar sulcus: 7 or 8. Maximum height of mesoscutellum lunules/maximum height of lateral face of mesoscutellum: 0.2–0.3. Propodeum areola: completely defined by carinae, including transverse carina extending to spiracle. Propodeum background sculpture: mostly sculptured. Mediotergite 1 length/width at posterior margin: 2.6–2.8. Mediotergite 1 shape: slightly widening from anterior margin to 0.7–0.8 mediotergite length (where maximum width is reached), then narrowing towards posterior margin. Mediotergite 1 sculpture: mostly sculptured, excavated area centrally with transverse striation inside and/or a polished knob centrally on posterior margin of mediotergite. Mediotergite 2 width at posterior margin/length: 2.8–3.1. Mediotergite 2 sculpture: mostly smooth. Outer margin of hypopygium: with a wide, medially folded, transparent, semi–desclerotized area; usually with 4 or more pleats. Ovipositor thickness: anterior width at most 2.0 × posterior width (beyond ovipositor constriction). Ovipositor sheaths length/metatibial length: 0.8–0.9. Length of fore wing veins r/2RS: 1.4–1.6. Length of fore wing veins 2RS/2M: 1.1–1.3. Length of fore wing veins 2M/(RS+M)b: 0.7–0.8. Pterostigma length/width: 3.1–3.5. Point of insertion of vein r in pterostigma: about half way point length of pterostigma. Angle of vein r with fore wing anterior margin: clearly outwards, inclined towards fore wing apex. Shape of junction of veins r and 2RS in fore wing: distinctly but not strongly angled.

**Male.** Darker coloration, especially all tergites and metacoxa (which is dark brown).

#### Molecular data.

Sequences in BOLD: 21, barcode compliant sequences: 21.

#### Biology/ecology.

Solitary (Fig. 282). Hosts: Crambidae, an undescribed species of Spilomelinae with interim name “spiloBioLep01 BioLep577”.

#### Distribution.

Costa Rica, ACG.

#### Etymology.

We dedicate this species to Milena Gutiérrez in recognition of her diligent efforts for the ACG Programa Forestal and Restoracion.

### Apanteles minorcarmonai

Fernández-Triana sp. n.

http://zoobank.org/70357693-8B93-42A5-9D8D-169AA1E28A46

http://species-id.net/wiki/Apanteles_minorcarmonai

Figs 166 , 321

Apanteles Rodriguez57 (Smith et al. 2006). Interim name provided by the authors.

#### Type locality.

COSTA RICA, Alajuela, ACG, Sector Rincon Rain Forest, Sendero Rincon, 430m, 10.8962, -85.27769.

#### Holotype.

♀ in CNC. Specimen labels: 1. COSTA RICA, Alajuela, ACG, Sector Rincon Rain Forest, Sendero Rincon, 23.xii.2004, 430m, 10.8962, -85.27769, DHJPAR0002173.

#### Paratypes.

20 ♀, 6 ♂ (BMNH, CNC, INBIO, INHS, NMNH). COSTA RICA: ACG database codes: DHJPAR0002173, 04-SRNP-41651.

#### Description.

**Female.** Metatibia color (outer face): entirely or mostly (>0.7 metatibia length) dark brown to black, with yellow to white coloration usually restricted to anterior 0.2 or less. Fore wing veins color: veins C+Sc+R and R1 with brown coloration restricted narrowly to borders, interior area of those veins and pterostigma (and sometimes veins r, 2RS and 2M) transparent or white; other veins mostly transparent. Antenna length/body length: antenna about as long as body (head to apex of metasoma); if slightly shorter, at least extending beyond anterior 0.7 metasoma length or antenna shorter than body (head to apex of metasoma), not extending beyond anterior 0.7 metasoma length. Body length (head to apex of metasoma): 2.3–2.4 mm, rarely 2.5–2.6 mm. Fore wing length: 2.5–2.6 mm. Metafemur length/width: 3.2–3.3. Mediotergite 1 length/width at posterior margin: 2.3–2.4. Mediotergite 1 maximum width/width at posterior margin: 1.2–1.3. Ovipositor sheaths length/metafemur length: 1.0. Ovipositor sheaths length/metatibia length: 0.8.

#### Molecular data.

Sequences in BOLD: 4, barcode compliant sequences: 4.

#### Biology/ecology.

Gregarious (Fig. 321). Hosts: Hesperiidae, *Aguna* Burns01.

#### Distribution.

Costa Rica, ACG.

#### Etymology.

We dedicate this species to Minor Carmona in recognition of his diligent efforts for the ACG Programa de Parataxónomos and Estación Biológica Brasilia and Estación Biológica Caribe.

### Apanteles minornavarroi

Fernández-Triana sp. n.

http://zoobank.org/17FC4925-7C0C-435F-85D6-DEF4322F3576

http://species-id.net/wiki/Apanteles_minornavarroi

Figs 50 , 243

Apanteles Rodriguez43 (Smith et al. 2006). Interim name provided by the authors.

#### Type locality.

COSTA RICA, Guanacaste, ACG, Sector Pitilla, Colocho, 375m, 11.02367, -85.41884.

#### Holotype.

♀ in CNC. Specimen labels: 1. DHJPAR0012758. 2. Voucher: D.H.Janzen & W.Hallwachs, DB: http://janzen.sas.upenn.edu, Area de Conservación Guanacaste, COSTA RICA, 07-SRNP-30510.

#### Paratypes.

14 ♀, 2 ♂ (BMNH, CNC, INBIO, INHS, NMNH). COSTA RICA, ACG database codes: DHJPAR0002326, DHJPAR0012755, DHJPAR0012756, DHJPAR0035516, DHJPAR0038313, DHJPAR0038331, DHJPAR0038335, DHJPAR0038339, DHJPAR0038341, DHJPAR0038358, DHJPAR0038361, DHJPAR0038364, DHJPAR0038365, DHJPAR0039061, DHJPAR0039783.

#### Description.

**Female.** Body color: body mostly dark except for some sternites which may be pale. Antenna color: scape, pedicel, and flagellum dark. Coxae color (pro-, meso-, metacoxa): dark, dark, dark. Femora color (pro-, meso-, metafemur): anteriorly dark/posteriorly pale, dark, dark. Tibiae color (pro-, meso-, metatibia): pale, pale, anteriorly pale/posteriorly dark. Tegula and humeral complex color: tegula pale, humeral complex half pale/half dark. Pterostigma color: mostly pale and/or transparent, with thin dark borders. Fore wing veins color: partially pigmented (a few veins may be dark but most are pale). Antenna length/body length: antenna about as long as body (head to apex of metasoma); if slightly shorter, at least extending beyond anterior 0.7 metasoma length. Body in lateral view: not distinctly flattened dorso–ventrally. Body length (head to apex of metasoma): 3.3–3.4 mm, 3.5–3.6 mm or 3.7–3.8 mm. Fore wing length: 3.3–3.4 mm, 3.5–3.6 mm or 3.7–3.8 mm. Ocular–ocellar line/posterior ocellus diameter: 2.3–2.5. Interocellar distance/posterior ocellus diameter: 1.7–1.9. Antennal flagellomerus 2 length/width: 2.3–2.5. Antennal flagellomerus 14 length/width: 1.7–1.9. Length of flagellomerus 2/length of flagellomerus 14: 2.0–2.2. Tarsal claws: with single basal spine–like seta. Metafemur length/width: 3.2–3.3. Metatibia inner spur length/metabasitarsus length: 0.4–0.5. Anteromesoscutum: mostly with deep, dense punctures (separated by less than 2.0 × its maximum diameter). Mesoscutellar disc: mostly smooth. Number of pits in scutoscutellar sulcus: 11 or 12. Maximum height of mesoscutellum lunules/maximum height of lateral face of mesoscutellum: 0.6–0.7. Propodeum areola: completely defined by carinae, but only partial or absent transverse carina. Propodeum background sculpture: mostly sculptured. Mediotergite 1 length/width at posterior margin: 2.0–2.2. Mediotergite 1 shape: more or less parallel–sided. Mediotergite 1 sculpture: mostly sculptured, excavated area centrally with transverse striation inside and/or a polished knob centrally on posterior margin of mediotergite. Mediotergite 2 width at posterior margin/length: 2.0–2.3. Mediotergite 2 sculpture: mostly smooth. Outer margin of hypopygium: with a wide, medially folded, transparent, semi–desclerotized area; usually with 4 or more pleats. Ovipositor thickness: about same width throughout its length. Ovipositor sheaths length/metatibial length: 1.8–1.9. Length of fore wing veins r/2RS: 2.3 or more. Length of fore wing veins 2RS/2M: 1.1–1.3. Length of fore wing veins 2M/(RS+M)b: 0.5–0.6. Pterostigma length/width: 3.1–3.5. Point of insertion of vein r in pterostigma: clearly beyond half way point length of pterostigma. Angle of vein r with fore wing anterior margin: clearly outwards, inclined towards fore wing apex. Shape of junction of veins r and 2RS in fore wing: distinctly but not strongly angled.

**Male.** Similar to female, except for mediotergite 2 slightly less quadrate, i.e., more transverse.

#### Molecular data.

Sequences in BOLD: 40, barcode compliant sequences: 40.

#### Biology/ecology.

Solitary (Fig. 243). Hosts: Elachistidae, *Antaeotricha rensalariana*, *Antaeotricha* Janzen146, elachjanzen01 Janzen120.

#### Distribution.

Costa Rica, ACG.

#### Etymology.

We dedicate this species to Minor Navarro in recognition of his diligent support for the ACG Programa de Sectores.

### Apanteles monicachavarriae

Fernández-Triana sp. n.

http://zoobank.org/DB05F252-27D5-4F69-BC2C-4C6A1046A137

http://species-id.net/wiki/Apanteles_monicachavarriae

Figs 148 , 299

#### Type locality.

COSTA RICA, Alajuela, ACG, Sector Rincon Rain Forest, San Lucas, 320m, 10.91847, -85.30338.

#### Holotype.

♀ in CNC. Specimen labels: 1. DHJPAR0039017. 2. Voucher: D.H.Janzen & W.Hallwachs, DB: http://janzen.sas.upenn.edu, Area de Conservación Guanacaste, COSTA RICA, 10-SRNP-40550.

#### Paratypes.

1 ♀, 1 ♂ (CNC). COSTA RICA, ACG database codes: DHJPAR0039023, DHJPAR0043032.

#### Description.

**Female.** Body color: body mostly dark except for some sternites which may be pale. Antenna color: scape, pedicel, and flagellum pale. Coxae color (pro-, meso-, metacoxa): pale, dark, dark. Femora color (pro-, meso-, metafemur): pale, dark, dark. Tibiae color (pro-, meso-, metatibia): pale, pale, mostly pale but with posterior 0.2 or less dark. Tegula and humeral complex color: both pale. Pterostigma color: mostly pale and/or transparent, with thin dark borders. Fore wing veins color: mostly white or entirely transparent. Antenna length/body length: antenna about as long as body (head to apex of metasoma); if slightly shorter, at least extending beyond anterior 0.7 metasoma length. Body in lateral view: not distinctly flattened dorso–ventrally. Body length (head to apex of metasoma): 3.1–3.2 mm. Fore wing length: 3.7–3.8 mm. Ocular–ocellar line/posterior ocellus diameter: 2.0–2.2. Interocellar distance/posterior ocellus diameter: 1.7–1.9. Antennal flagellomerus 2 length/width: 2.3–2.5. Antennal flagellomerus 14 length/width: 1.4–1.6. Length of flagellomerus 2/length of flagellomerus 14: 2.3–2.5. Tarsal claws: with single basal spine–like seta. Metafemur length/width: 2.8–2.9. Metatibia inner spur length/metabasitarsus length: 0.6–0.7. Anteromesoscutum: mostly with deep, dense punctures (separated by less than 2.0 × its maximum diameter). Mesoscutellar disc: with punctures near margins, central part mostly smooth. Number of pits in scutoscutellar sulcus: 11 or 12. Maximum height of mesoscutellum lunules/maximum height of lateral face of mesoscutellum: 0.6–0.7. Propodeum areola: completely defined by carinae, including transverse carina extending to spiracle. Propodeum background sculpture: partly sculptured, especially on anterior 0.5. Mediotergite 1 length/width at posterior margin: 1.4–1.6. Mediotergite 1 shape: more or less parallel–sided. Mediotergite 1 sculpture: mostly sculptured, excavated area centrally with transverse striation inside and/or a polished knob centrally on posterior margin of mediotergite. Mediotergite 2 width at posterior margin/length: 4.4–4.7. Mediotergite 2 sculpture: mostly smooth. Outer margin of hypopygium: with a wide, medially folded, transparent, semi–desclerotized area; usually with 4 or more pleats. Ovipositor thickness: about same width throughout its length (?). Ovipositor sheaths length/metatibial length: 1.0–1.1. Length of fore wing veins r/2RS: 1.7–1.9. Length of fore wing veins 2RS/2M: 1.1–1.3. Length of fore wing veins 2M/(RS+M)b: 0.9–1.0. Pterostigma length/width: 3.1–3.5. Point of insertion of vein r in pterostigma: clearly beyond half way point length of pterostigma. Angle of vein r with fore wing anterior margin: clearly outwards, inclined towards fore wing apex. Shape of junction of veins r and 2RS in fore wing: distinctly but not strongly angled.

**Male.** Like female but mediotergite 1 is comparatively narrower.

#### Molecular data.

Sequences in BOLD: 6, barcode compliant sequences: 6.

#### Biology/ecology.

Solitary (Fig. 299). Hosts: Pyralidae, chryBioLep01 BioLep803, chryBioLep01 BioLep506, chryJanzen01 Janzen165.

#### Distribution.

Costa Rica, ACG.

#### Comments.

This species is characterized by pterostigma fully transparent or mostly transparent with only thin brown borders, tegula and humeral complex yellow, all coxae dark brown to black, mediotergite 2 mostly smooth, and mediotergite 1 relatively wide (its length 1.5 × its width at posterior margin). It is supported by the Bayesian molecular analysis as divergent from other species, although the data suggests it might be related to the *glenriverai* group (Fig. 1). However, we have not placed *Apanteles monicachavarriae* within the *glenriverai* group because of the morphological differences, although future studies may change this situation.

#### Etymology.

We dedicate this species to Mónica Chavarría in recognition of her diligent efforts for the ACG Liberia office.

### Apanteles oscarchavezi

Fernández-Triana sp. n.

http://zoobank.org/FEC95685-635B-4AB6-8FA7-11B958F835E7

http://species-id.net/wiki/Apanteles_oscarchavezi

Fig. 149

#### Type locality.

COSTA RICA, Alajuela, Sector San Cristobal, Estación San Gerardo, 575m, 10.88009, -85.38887.

#### Holotype.

♀ in CNC. Specimen labels: 1. San Gerardo: Est. San Gerardo, Date: 1 Mar-15 May 08. 2. DHJPAR0026271.

#### Paratypes.

2 ♀, 5 ♂ (CNC). COSTA RICA, Alajuela, ACG database codes: DHJPAR0012743, DHJPAR0013191, DHJPAR0013424, DHJPAR0013542, DHJPAR0013637, DHJPAR0024664, DHJPAR0026280.

#### Description.

**Female.** Body color: body mostly dark except for some sternites which may be pale. Antenna color: scape, pedicel, and flagellum dark. Coxae color (pro-, meso-, metacoxa): pale, pale, dark. Femora color (pro-, meso-, metafemur): pale, pale, mostly pale but posterior 0.2 or less dark. Tibiae color (pro-, meso-, metatibia): pale, pale, mostly pale but with posterior 0.2 or less dark. Tegula and humeral complex color: both pale. Pterostigma color: dark. Fore wing veins color: mostly dark (a few veins may be unpigmented). Antenna length/body length: antenna about as long as body (head to apex of metasoma); if slightly shorter, at least extending beyond anterior 0.7 metasoma length. Body in lateral view: not distinctly flattened dorso–ventrally. Body length (head to apex of metasoma): 2.5–2.6 mm or 2.7–2.8 mm. Fore wing length: 2.5–2.6 mm or 2.7–2.8 mm. Ocular–ocellar line/posterior ocellus diameter: 2.0–2.2. Interocellar distance/posterior ocellus diameter: 1.7–1.9. Antennal flagellomerus 2 length/width: 2.6–2.8. Antennal flagellomerus 14 length/width: 1.0 or less. Length of flagellomerus 2/length of flagellomerus 14: 2.6–2.8. Tarsal claws: with single basal spine–like seta. Metafemur length/width: 3.0–3.1. Metatibia inner spur length/metabasitarsus length: 0.4–0.5. Anteromesoscutum: mostly with deep, dense punctures (separated by less than 2.0 × its maximum diameter). Mesoscutellar disc: with punctures near margins, central part mostly smooth. Number of pits in scutoscutellar sulcus: 9 or 10. Maximum height of mesoscutellum lunules/maximum height of lateral face of mesoscutellum: 0.4–0.5. Propodeum areola: completely defined by carinae, including transverse carina extending to spiracle. Propodeum background sculpture: mostly sculptured. Mediotergite 1 length/width at posterior margin: 2.3–2.5. Mediotergite 1 shape: more or less parallel–sided. Mediotergite 1 sculpture: mostly sculptured, excavated area centrally with transverse striation inside and/or a polished knob centrally on posterior margin of mediotergite. Mediotergite 2 width at posterior margin/length: 3.6–3.9. Mediotergite 2 sculpture: with some sculpture, mostly near posterior margin. Outer margin of hypopygium: with a medially folded, transparent, semi–desclerotized area; with 0–3 pleats visible. Ovipositor thickness: anterior width at most 2.0 × posterior width (beyond ovipositor constriction). Ovipositor sheaths length/metatibial length: 0.6–0.7. Length of fore wing veins r/2RS: 1.4–1.6. Length of fore wing veins 2RS/2M: 1.1–1.3. Length of fore wing veins 2M/(RS+M)b: 0.7–0.8. Pterostigma length/width: 2.6–3.0. Point of insertion of vein r in pterostigma: clearly before half way point length of pterostigma. Angle of vein r with fore wing anterior margin: clearly outwards, inclined towards fore wing apex. Shape of junction of veins r and 2RS in fore wing: distinctly but not strongly angled.

**Male.** As in female.

#### Molecular data.

Sequences in BOLD: 49 (32 from Mexico, 17 from ACG), barcode compliant sequences: 49.

#### Biology/ecology.

Malaise-trapped.

#### Distribution.

Costa Rica, ACG; Mexico, State of Jalisco.

#### Comments.

This species is characterized by hypopygium with a median folded, transparent, semi-desclerotized area with 1–3 pleats visible; ovipositor thin (thinner than width of median flagellomerus), and with basal width <2.0 × its apical width after constriction; tarsal claws with one basal spine-like seta; mediotergite 1 length 2.3 × its width; and mediotergite 2 with some sculpture near its posterior margin. The molecular data supports this species as divergent (Fig. 1).

#### Etymology.

We dedicate this species to Oscar Chávez in recognition of his diligent efforts as ACG chofer.

### Apanteles osvaldoespinozai

Fernández-Triana sp. n.

http://zoobank.org/40483CDB-1514-4F90-972D-0568267E675D

http://species-id.net/wiki/Apanteles_osvaldoespinozai

Figs 63 , 255

Apanteles Rodriguez06 (Smith et al. 2006). Interim name provided by the authors.

#### Type locality.

COSTA RICA, Guanacaste, ACG, Sector San Cristobal, Quebrada Cementerio, 700m, 10.87124, -85.38749.

#### Holotype.

♀ in CNC. Specimen labels: 1. COSTA RICA, Guanacaste, ACG, Sector San Cristobal, Quebrada Cementerio, 23 Sept. 1999, Gloria Sihezar. 2. 99-SRNP-13043, *Astraptes* on *Senna papillosa*. 3. DHJPAR0004020.

#### Paratypes.

159 ♀, 81 ♂ (BMNH, CNC, INBIO, INHS, NMNH). COSTA RICA: ACG database codes: See Appendix 2 for detailed label data.

#### Description.

**Female.** Body color: body mostly dark except for some sternites which may be pale. Antenna color: scape, pedicel, and flagellum dark. Coxae color (pro-, meso-, metacoxa): pale, dark, dark. Femora color (pro-, meso-, metafemur): pale, pale, mostly pale but posterior 0.2 or less dark. Tibiae color (pro-, meso-, metatibia): pale, pale, mostly pale but with posterior 0.2 or less dark. Tegula and humeral complex color: tegula pale, humeral complex half pale/half dark. Pterostigma color: mostly dark, with small pale area centrally. Fore wing veins color: mostly dark (a few veins may be unpigmented). Antenna length/body length: antenna about as long as body (head to apex of metasoma); if slightly shorter, at least extending beyond anterior 0.7 metasoma length. Body in lateral view: not distinctly flattened dorso–ventrally. Body length (head to apex of metasoma): 2.9–3.0 mm, 3.1–3.2 mm, rarely 3.3–3.4 mm. Fore wing length: 3.1–3.2 mm, 3.3–3.4 mm, rarely 2.9–3.0 mm or 3.5–3.6 mm. Ocular–ocellar line/posterior ocellus diameter: 2.0–2.2. Interocellar distance/posterior ocellus diameter: 1.4–1.6. Antennal flagellomerus 2 length/width: 2.6–2.8. Antennal flagellomerus 14 length/width: 1.7–1.9. Length of flagellomerus 2/length of flagellomerus 14: 2.0–2.2. Tarsal claws: with single basal spine–like seta or with two basal spine–like setae. Metafemur length/width: 3.0–3.1. Metatibia inner spur length/metabasitarsus length: 0.6–0.7. Anteromesoscutum: mostly with deep, dense punctures (separated by less than 2.0 × its maximum diameter). Mesoscutellar disc: with punctures near margins, central part mostly smooth. Number of pits in scutoscutellar sulcus: 7 or 8. Maximum height of mesoscutellum lunules/maximum height of lateral face of mesoscutellum: 0.6–0.7. Propodeum areola: completely defined by carinae, including transverse carina extending to spiracle. Propodeum background sculpture: partly sculptured, especially on anterior 0.5. Mediotergite 1 length/width at posterior margin: 3.2–3.4. Mediotergite 1 shape: more or less parallel–sided. Mediotergite 1 sculpture: with some sculpture near lateral margins and/or posterior 0.2–0.4 of mediotergite. Mediotergite 2 width at posterior margin/length: 3.2–3.5. Mediotergite 2 sculpture: mostly smooth. Outer margin of hypopygium: inflexible (without a folded, transparent, semi–desclerotized area); with no pleats visible. Ovipositor thickness: anterior width 3.0–5.0 × posterior width (beyond ovipositor constriction). Ovipositor sheaths length/metatibial length: 0.6–0.7. Length of fore wing veins r/2RS: 2.3 or more. Length of fore wing veins 2RS/2M: 0.9–1.0. Length of fore wing veins 2M/(RS+M)b: 0.9–1.0. Pterostigma length/width: 3.6 or more. Point of insertion of vein r in pterostigma: about half way point length of pterostigma. Angle of vein r with fore wing anterior margin: more or less perpendicular to fore wing margin. Shape of junction of veins r and 2RS in fore wing: distinctly but not strongly angled.

**Male.** Similar to female, but with darker coloration, especially on hind legs.

#### Molecular data.

Sequences in BOLD: 116, barcode compliant sequences: 100.

#### Biology/ecology.

Gregarious (Fig. 255). Hosts: Hesperiidae, *Astraptes obstupefactus*, *Astraptes*, *augeas, Astraptes inflatio, Astraptes fruticibus, Astraptes viracocha, Astraptes augeas*.

#### Distribution.

Costa Rica, ACG.

#### Etymology.

We dedicate this species to Osvaldo Espinoza recognition of his diligent efforts for the ACG Programa de Parataxónomos and Estación Biológica San Gerardo.

### Apanteles pablotranai

Fernández-Triana sp. n.

http://zoobank.org/361F965A-80CA-4CD3-92AF-BF22E9CBF61F

http://species-id.net/wiki/Apanteles_pablotranai

Fig. 126

#### Type locality.

COSTA RICA, Alajuela, ACG, Sector San Cristobal, Bosque Trampa Malaise, 815m, 10.86280, -85.38460.

#### Holotype.

♀ in CNC. Specimen labels: 1. DHJPAR0027654. 2. San Gerardo, MT, San Cristobal, 24-30 January 2008.

#### Description.

**Female.** Body color: body mostly dark except for some sternites which may be pale. Antenna color: scape, pedicel, and flagellum dark. Coxae color (pro-, meso-, metacoxa): dark, dark, dark. Femora color (pro-, meso-, metafemur): anteriorly dark/posteriorly pale, dark, dark. Tibiae color (pro-, meso-, metatibia): pale, pale, anteriorly pale/posteriorly dark. Tegula and humeral complex color: both dark. Pterostigma color: dark. Fore wing veins color: mostly dark (a few veins may be unpigmented). Antenna length/body length: antenna about as long as body (head to apex of metasoma); if slightly shorter, at least extending beyond anterior 0.7 metasoma length. Body in lateral view: not distinctly flattened dorso–ventrally. Body length (head to apex of metasoma): 2.5–2.6 mm. Fore wing length: 2.5–2.6 mm. Ocular–ocellar line/posterior ocellus diameter: 2.3–2.5. Interocellar distance/posterior ocellus diameter: 1.4–1.6. Antennal flagellomerus 2 length/width: 3.2 or more. Antennal flagellomerus 14 length/width: 1.1–1.3. Length of flagellomerus 2/length of flagellomerus 14: 2.6–2.8. Tarsal claws: with single basal spine–like seta. Metafemur length/width: 3.0–3.1. Metatibia inner spur length/metabasitarsus length: 0.4–0.5. Anteromesoscutum: mostly with deep, dense punctures (separated by less than 2.0 × its maximum diameter). Mesoscutellar disc: with punctures near margins, central part mostly smooth. Number of pits in scutoscutellar sulcus: 11 or 12. Maximum height of mesoscutellum lunules/maximum height of lateral face of mesoscutellum: 0.4–0.5. Propodeum areola: completely defined by carinae, including transverse carina extending to spiracle. Propodeum background sculpture: partly sculptured, especially on anterior 0.5. Mediotergite 1 length/width at posterior margin: 1.1–1.3. Mediotergite 1 shape: clearly widening towards posterior margin. Mediotergite 1 sculpture: mostly sculptured, excavated area centrally with transverse striation inside and/or a polished knob centrally on posterior margin of mediotergite. Mediotergite 2 width at posterior margin/length: 4.4–4.7. Mediotergite 2 sculpture: more or less fully sculptured, with longitudinal striation. Outer margin of hypopygium: with a wide, medially folded, transparent, semi–desclerotized area; usually with 4 or more pleats. Ovipositor thickness: anterior width at most 2.0 × posterior width (beyond ovipositor constriction). Ovipositor sheaths length/metatibial length: 1.2–1.3. Length of fore wing veins r/2RS: 1.1–1.3. Length of fore wing veins 2RS/2M: 1.1–1.3. Length of fore wing veins 2M/(RS+M)b: 0.9–1.0. Pterostigma length/width: 3.1–3.5. Point of insertion of vein r in pterostigma: about half way point length of pterostigma. Angle of vein r with fore wing anterior margin: more or less perpendicular to fore wing margin. Shape of junction of veins r and 2RS in fore wing: distinctly but not strongly angled.

**Male.** Unknown.

#### Molecular data.

Sequences in BOLD: 1, barcode compliant sequences: 1.

#### Biology/ecology.

Malaise-trapped.

#### Distribution.

Costa Rica, ACG.

#### Etymology.

We dedicate this species to Pablo Trana in recognition of his diligent efforts for the ACG Programa de Sectores.

### Apanteles pabloumanai

Fernández-Triana sp. n.

http://zoobank.org/3F5AE9AA-AC5C-427D-A8F5-9D1AF4AB2FFE

http://species-id.net/wiki/Apanteles_pabloumanai

Figs 193 , 322

Apanteles Rodriguez19 (Smith et al. 2006). Interim name provided by the authors.

#### Type locality.

COSTA RICA, Guanacaste, ACG, Sector Santa Rosa, Bosque San Emilio, 300m, 10.84389, -85.61384.

#### Holotype.

♀ in CNC. Specimen labels: 1. DHJPAR0002686.

#### Paratypes.

12 ♀, 4 ♂ (BMNH, CNC, INBIO, INHS, NMNH). COSTA RICA: ACG database codes: DHJPAR0001607, 99-SRNP-895.

#### Description.

**Female.** Metatibia color (outer face): entirely or mostly (>0.7 metatibia length) dark brown to black, with yellow to white coloration usually restricted to anterior 0.2 or less. Fore wing veins color: veins C+Sc+R and R1 mostly brown; usually veins r, 2RS, 2M, (RS+M)b, 1CU, 2Cua, and 1m–cu partially brown; interior area of other veins, and at least part of pterostigma, usually light brown or yellowish–white. Antenna length/body length: antenna about as long as body (head to apex of metasoma); if slightly shorter, at least extending beyond anterior 0.7 metasoma length. Body length (head to apex of metasoma): 2.3–2.4 mm, 2.5–2.6 mm, rarely 2.1–2.2 mm. Fore wing length: 2.5–2.6 mm or 2.7–2.8 mm. Metafemur length/width: 2.8–2.9 or 3.0–3.1. Mediotergite 1 length/width at posterior margin: 2.3–2.4. Mediotergite 1 maximum width/width at posterior margin: 1.4–1.5. Ovipositor sheaths length/metafemur length: 0.9 or 1.0. Ovipositor sheaths length/metatibia length: 0.8 or 0.9.

#### Molecular data.

Sequences in BOLD: 5, barcode compliant sequences: 4.

#### Biology/ecology.

Gregarious (Fig. 322). Host: Hesperiidae, *Calliades zeutus*.

#### Distribution.

Costa Rica, ACG.

#### Etymology.

We dedicate this species to Pablo Umana in recognition of his diligent efforts for the ACG Programa de Parataxónomos and for Estación Biológica Caribe of Sector Rincon Rain Forest, ACG.

### Apanteles pablovasquezi

Fernández-Triana sp. n.

http://zoobank.org/94C5AD3D-A87A-4C7B-9ED7-AC07D51A4AF5

http://species-id.net/wiki/Apanteles_pablovasquezi

Figs 121 , 287

#### Type locality.

COSTA RICA, Guanacaste, ACG, Sector San Cristobal, Tajo Angeles, 540m, 10.86472, -85.41531.

#### Holotype.

♀ in CNC. Specimen labels: 1. DHJPAR0041619.

#### Paratypes.

5 ♀, 1 ♂ (BMNH, CNC, NMNH). COSTA RICA, ACG database codes: DHJPAR0041619.

#### Description.

**Female.** Body color: body mostly dark except for some sternites which may be pale. Antenna color: scape, pedicel, and flagellum dark. Coxae color (pro-, meso-, metacoxa): dark, dark, dark. Femora color (pro-, meso-, metafemur): anteriorly dark/posteriorly pale, dark, dark. Tibiae color (pro-, meso-, metatibia): pale, pale, mostly dark but anterior 0.2 or less pale. Tegula and humeral complex color: tegula pale, humeral complex half pale/half dark. Pterostigma color: mostly pale and/or transparent, with thin dark borders. Fore wing veins color: mostly white or entirely transparent. Antenna length/body length: antenna shorter than body (head to apex of metasoma), not extending beyond anterior 0.7 metasoma length. Body in lateral view: not distinctly flattened dorso–ventrally. Body length (head to apex of metasoma): 2.1–2.2 mm or 2.3–2.4 mm. Fore wing length: 2.5–2.6 mm. Ocular–ocellar line/posterior ocellus diameter: 2.6 or more. Interocellar distance/posterior ocellus diameter: 2.0–2.2. Antennal flagellomerus 2 length/width: 2.6–2.8. Antennal flagellomerus 14 length/width: 1.1–1.3. Length of flagellomerus 2/length of flagellomerus 14: 2.0–2.2. Tarsal claws: simple. Metafemur length/width: 3.2–3.3. Metatibia inner spur length/metabasitarsus length: 0.4–0.5. Anteromesoscutum: mostly with deep, dense punctures (separated by less than 2.0 × its maximum diameter). Mesoscutellar disc: with punctures near margins, central part mostly smooth. Number of pits in scutoscutellar sulcus: 9 or 10. Maximum height of mesoscutellum lunules/maximum height of lateral face of mesoscutellum: 0.4–0.5. Propodeum areola: completely defined by carinae, including transverse carina extending to spiracle. Propodeum background sculpture: partly sculptured, especially on anterior 0.5. Mediotergite 1 length/width at posterior margin: 2.3–2.5. Mediotergite 1 shape: slightly widening from anterior margin to 0.7–0.8 mediotergite length (where maximum width is reached), then narrowing towards posterior margin. Mediotergite 1 sculpture: with some sculpture near lateral margins and/or posterior 0.2–0.4 of mediotergite. Mediotergite 2 width at posterior margin/length: 4.0–4.3. Mediotergite 2 sculpture: mostly smooth. Outer margin of hypopygium: with a wide, medially folded, transparent, semi–desclerotized area; usually with 4 or more pleats. Ovipositor thickness: anterior width at most 2.0 × posterior width (beyond ovipositor constriction). Ovipositor sheaths length/metatibial length: 0.6–0.7. Length of fore wing veins r/2RS: 2.3 or more. Length of fore wing veins 2RS/2M: 1.1–1.3. Length of fore wing veins 2M/(RS+M)b: 0.7–0.8. Pterostigma length/width: 2.6–3.0. Point of insertion of vein r in pterostigma: about half way point length of pterostigma. Angle of vein r with fore wing anterior margin: clearly outwards, inclined towards fore wing apex. Shape of junction of veins r and 2RS in fore wing: evenly curved.

**Male.** Similar to female, but with darker coloration in hind legs (especially metatibia) and narrower mediotergite 1.

#### Molecular data.

Sequences in BOLD: 1, barcode compliant sequences: 1.

#### Biology/ecology.

Gregarious (Fig. 287). Host: Pyralidae, an undescribed species with the interim name epipajanzen01 Janzen09 in the ACG database.

#### Distribution.

Costa Rica, ACG.

#### Etymology.

We dedicate this species to Pablo Vásquez in recognition of his diligent efforts for the ACG Programa de Educacion Biológica.

### Apanteles paranthrenidis

Muesebeck, 1921

http://species-id.net/wiki/Apanteles_paranthrenidis

Fig. 152

Apanteles paranthrenidis Muesebeck, 1921: 506.

#### Type locality.

UNITED STATES: California, Los Angeles County, locality not specified.

#### Holotype.

♀, NMNH (examined).

#### Material Examined.

1 ♀ (CNC), USA: PA, Rochville, coll. W. S. Fisch; 1 ♀, 1 ♂ (CNC), USA, 30.x.1903, coll. E. P. Felt; 1 ♀ (CNC), USA: NC, Bertie County near Cahaba, 2.vi.1976.

#### Description.

**Female.** Body color: body mostly dark except for some sternites which may be pale. Antenna color: scape, pedicel, and flagellum dark. Coxae color (pro-, meso-, metacoxa): pale, pale, dark or pale, pale, partially pale/partially dark. Femora color (pro-, meso-, metafemur): pale, pale, pale. Tibiae color (pro-, meso-, metatibia): pale, pale, pale. Tegula and humeral complex color: tegula pale, humeral complex half pale/half dark. Pterostigma color: mostly pale and/or transparent, with thin dark borders. Fore wing veins color: mostly white or entirely transparent. Antenna length/body length: antenna about as long as body (head to apex of metasoma); if slightly shorter, at least extending beyond anterior 0.7 metasoma length. Body in lateral view: not distinctly flattened dorso–ventrally. Body length (head to apex of metasoma): 3.7–3.8 mm. Fore wing length: 3.9–4.0 mm. Ocular–ocellar line/posterior ocellus diameter: 2.0–2.2. Interocellar distance/posterior ocellus diameter: 1.7–1.9. Antennal flagellomerus 2 length/width: 2.0–2.2. Antennal flagellomerus 14 length/width: 1.7–1.9. Length of flagellomerus 2/length of flagellomerus 14: 1.7–1.9. Tarsal claws: simple. Metafemur length/width: 3.2–3.3. Metatibia inner spur length/metabasitarsus length: 0.4–0.5 or 0.6–0.7. Anteromesoscutum: mostly with deep, dense punctures (separated by less than 2.0 × its maximum diameter). Mesoscutellar disc: with punctures near margins, central part mostly smooth. Number of pits in scutoscutellar sulcus: 7 or 8 or 9 or 10. Maximum height of mesoscutellum lunules/maximum height of lateral face of mesoscutellum: 0.8 or more. Propodeum areola: completely defined by carinae, including transverse carina extending to spiracle. Propodeum background sculpture: mostly sculptured. Mediotergite 1 length/width at posterior margin: 1.1–1.3 or 1.4–1.6. Mediotergite 1 shape: clearly widening towards posterior margin. Mediotergite 1 sculpture: mostly sculptured, excavated area centrally with transverse striation inside and/or a polished knob centrally on posterior margin of mediotergite. Mediotergite 2 width at posterior margin/length: 3.6–3.9 or 4.0–4.3. Mediotergite 2 sculpture: with some sculpture, mostly near posterior margin. Outer margin of hypopygium: with a wide, medially folded, transparent, semi–desclerotized area; usually with 4 or more pleats. Ovipositor thickness: about same width throughout its length. Ovipositor sheaths length/metatibial length: 1.4–1.5. Length of fore wing veins r/2RS: 2.3 or more. Length of fore wing veins 2RS/2M: 1.1–1.3. Length of fore wing veins 2M/(RS+M)b: 0.7–0.8. Pterostigma length/width: 3.1–3.5. Point of insertion of vein r in pterostigma: clearly beyond half way point length of pterostigma. Angle of vein r with fore wing anterior margin: clearly outwards, inclined towards fore wing apex. Shape of junction of veins r and 2RS in fore wing: strongly angulated, sometimes with a knob.

#### Molecular data.

No molecular data available for this species.

#### Biology/ecology.

Gregarious; coccons large, white, not imbedded in a mass of silk but formed in the burrows of the host (Muesebeck 1921). Hosts: Noctuidae, *Helicoverpa zea* (miner in ears of corn), Sesiidae, *Paranthrene asilipennis*, *Paranthrene dolli*, *Paranthrene robiniae* (miners in stems of unknown host plant).

#### Distribution.

Mexico, United States (CA, DC, FL, MS, NY, OK, PA, here recorded for the first time from NC). There is no suggestion that this species occurs in ACG.

#### Comments.

Because of the holotype is missing both fore wings and antenna, the morphological characters related to those body parts were coded in the Lucid database from the female specimens deposited in the CNC.

### Apanteles paulaixcamparijae

Fernández-Triana sp. n.

http://zoobank.org/E5146AAA-CF6E-4984-9F17-29B91CAA121E

http://species-id.net/wiki/Apanteles_paulaixcamparijae

Figs 18 , 220

Apanteles Rodriguez169. Smith et al. (2008). Interim name provided by the authors.

#### Type locality.

COSTA RICA, Guanacaste, ACG, Sector Cacao, Sendero a Maritza, 570m, 10.95727, -85.49514.

#### Holotype.

♀ in CNC. Specimen labels: 1. Costa Rica: Guanacaste, ACG, Sector Cacao, Sendero a Maritza, 23.viii.2006, 570m, 10.95727, -85.49514, 06-SRNP-36069.

#### Paratypes.

10 ♀, 6 ♂ (BMNH, CNC, INBIO, INHS, NMNH). COSTA RICA, ACG database codes: DHJPAR0012298, DHJPAR0038032, 06-SRNP-36062.

#### Description.

**Female.** Body color: body mostly dark except for some sternites which may be pale. Antenna color: scape, pedicel, and flagellum dark. Coxae color (pro-, meso-, metacoxa): dark, dark, dark. Femora color (pro-, meso-, metafemur): anteriorly dark/posteriorly pale, dark, dark. Tibiae color (pro-, meso-, metatibia): pale, pale, anteriorly pale/posteriorly dark. Tegula and humeral complex color: tegula pale, humeral complex half pale/half dark. Pterostigma color: mostly pale and/or transparent, with thin dark borders. Fore wing veins color: partially pigmented (a few veins may be dark but most are pale). Antenna length/body length: antenna about as long as body (head to apex of metasoma); if slightly shorter, at least extending beyond anterior 0.7 metasoma length. Body in lateral view: not distinctly flattened dorso–ventrally. Body length (head to apex of metasoma): 2.5–2.6 mm, 2.7–2.8 mm, rarely 2.9–3.0 mm. Fore wing length: 2.7–2.8 mm, 2.9–3.0 mm, rarely 3.1–3.2 mm. Ocular–ocellar line/posterior ocellus diameter: 2.3–2.5. Interocellar distance/posterior ocellus diameter: 1.7–1.9. Antennal flagellomerus 2 length/width: 2.9–3.1. Antennal flagellomerus 14 length/width: 1.7–1.9. Length of flagellomerus 2/length of flagellomerus 14: 2.0–2.2. Tarsal claws: with single basal spine–like seta. Metafemur length/width: 3.0–3.1. Metatibia inner spur length/metabasitarsus length: 0.4–0.5. Anteromesoscutum: mostly with deep, dense punctures (separated by less than 2.0 × its maximum diameter). Mesoscutellar disc: mostly punctured. Number of pits in scutoscutellar sulcus: 9 or 10. Maximum height of mesoscutellum lunules/maximum height of lateral face of mesoscutellum: 0.6–0.7. Propodeum areola: completely defined by carinae, including transverse carina extending to spiracle. Propodeum background sculpture: mostly sculptured. Mediotergite 1 length/width at posterior margin: 1.7–1.9. Mediotergite 1 shape: more or less parallel–sided. Mediotergite 1 sculpture: mostly sculptured, excavated area centrally with transverse striation inside and/or a polished knob centrally on posterior margin of mediotergite. Mediotergite 2 width at posterior margin/length: 3.2–3.5. Mediotergite 2 sculpture: with some sculpture, mostly near posterior margin. Outer margin of hypopygium: with a wide, medially folded, transparent, semi–desclerotized area; usually with 4 or more pleats. Ovipositor thickness: about same width throughout its length. Ovipositor sheaths length/metatibial length: 1.2–1.3. Length of fore wing veins r/2RS: 1.4–1.6. Length of fore wing veins 2RS/2M: 0.9–1.0. Length of fore wing veins 2M/(RS+M)b: 0.9–1.0. Pterostigma length/width: 3.1–3.5. Point of insertion of vein r in pterostigma: clearly beyond half way point length of pterostigma. Angle of vein r with fore wing anterior margin: more or less perpendicular to fore wing margin. Shape of junction of veins r and 2RS in fore wing: distinctly but not strongly angled.

**Male.** As in female, except for darker legs and narrower mediotergite 1.

#### Molecular data.

Sequences in BOLD: 3, barcode compliant sequences: 3.

#### Biology/ecology.

Gregarious (Fig. 220). Host: Elachistidae, *Stenoma patens* DHJ06, *Stenoma* BioLep30.

#### Distribution.

Costa Rica, ACG.

#### Etymology.

We dedicate this species to Paula Ixcamparij in recognition of her diligent efforts for the ACG Subregional Liberia.

### Apanteles petronariosae

Fernández-Triana sp. n.

http://zoobank.org/BFDB4BFE-543A-4DCE-AB8D-BCD8C6EE9E02

http://species-id.net/wiki/Apanteles_petronariosae

Figs 89 , 268

Apanteles Rodriguez61 (Smith et al. 2006). Interim name provided by the authors.

#### Type locality.

COSTA RICA, Alajuela, Sector Rincon Rain Forest, Sendero Tucán, 410m, 10.90424, -85.2712.

#### Holotype.

♀ in CNC. Specimen labels: 1. COSTA RICA: Alajuela, ACG, Sector Rincon Rain Forest, Sendero Tucan, 14.xi.2006, 410m, 10.90424, -85.2712, 06-SRNP-44315. 2. DHJPAR0012799. 3. Voucher: D.H.Janzen & W.Hallwachs, DB: http://janzen.sas.upenn.edu, Area de Conservación Guanacaste, COSTA RICA, 06-SRNP-44315.

#### Paratypes.

6 ♀ (CNC, NMNH, BMNH). Costa Rica: Alajuela, ACG database codes: 02-SRNP-6035, 06-SRNP-30165, 06-SRNP-32791, 09-SRNP-71961, 09-SRNP-72872, 10-SRNP-70644.

#### Description.

**Female.** Body color: body mostly dark except for some sternites which may be pale. Antenna color: scape, pedicel, and flagellum dark. Coxae color (pro-, meso-, metacoxa): dark, dark, dark. Femora color (pro-, meso-, metafemur): pale, pale, pale. Tibiae color (pro-, meso-, metatibia): pale, pale, mostly pale but with posterior 0.2 or less dark. Tegula and humeral complex color: tegula pale, humeral complex dark. Pterostigma color: dark. Fore wing veins color: mostly dark (a few veins may be unpigmented). Antenna length/body length: antenna about as long as body (head to apex of metasoma); if slightly shorter, at least extending beyond anterior 0.7 metasoma length. Body in lateral view: not distinctly flattened dorso–ventrally. Body length (head to apex of metasoma): 4.0 mm or more. Fore wing length: 4.0 mm or more. Ocular–ocellar line/posterior ocellus diameter: 1.7–1.9. Interocellar distance/posterior ocellus diameter: 1.7–1.9. Antennal flagellomerus 2 length/width: 2.3–2.5, 2.6–2.8, rarely 2.0–2.2 or 2.9–3.1. Antennal flagellomerus 14 length/width: 2.3–2.5 or 2.6–2.9. Length of flagellomerus 2/length of flagellomerus 14: 1.1–1.3. Tarsal claws: pectinate. Metafemur length/width: 3.0–3.1. Metatibia inner spur length/metabasitarsus length: 0.6–0.7. Anteromesoscutum: mostly with deep, dense punctures (separated by less than 2.0 × its maximum diameter). Mesoscutellar disc: with punctures near margins, central part mostly smooth. Number of pits in scutoscutellar sulcus: 7 or 8, rarely 5 or 6. Maximum height of mesoscutellum lunules/maximum height of lateral face of mesoscutellum: 0.6–0.7. Propodeum areola: completely defined by carinae, including transverse carina extending to spiracle. Propodeum background sculpture: mostly sculptured. Mediotergite 1 length/width at posterior margin: 2.0–2.2. Mediotergite 1 shape: more or less parallel–sided. Mediotergite 1 sculpture: with some sculpture near lateral margins and/or posterior 0.2–0.4 of mediotergite. Mediotergite 2 width at posterior margin/length: 4.0–4.3. Mediotergite 2 sculpture: with some sculpture, mostly near posterior margin. Outer margin of hypopygium: with a wide, medially folded, transparent, semi–desclerotized area; usually with 4 or more pleats. Ovipositor thickness: anterior width 3.0–5.0 × posterior width (beyond ovipositor constriction). Ovipositor sheaths length/metatibial length: 0.8–0.9. Length of fore wing veins r/2RS: 2.3 or more. Length of fore wing veins 2RS/2M: 1.4–1.6. Length of fore wing veins 2M/(RS+M)b: 0.7–0.8. Pterostigma length/width: 3.1–3.5. Point of insertion of vein r in pterostigma: clearly beyond half way point length of pterostigma. Angle of vein r with fore wing anterior margin: clearly outwards, inclined towards fore wing apex. Shape of junction of veins r and 2RS in fore wing: distinctly but not strongly angled.

**Male.** Unknown.

#### Molecular data.

Sequences in BOLD: 12, barcode compliant sequences: 11.

#### Biology/ecology.

Solitary (Fig. 268). Hosts: Hesperiidae, *Ouleus dilla baru*.

#### Distribution.

Costa Rica, ACG.

#### Etymology.

We dedicate this species to Petrona Ríos in recognition of her diligent efforts for the ACG Programa de Parataxónomos and Estación Biológica Pitilla of ACG.

### Apanteles randallgarciai

Fernández-Triana sp. n.

http://zoobank.org/E9F8CA09-9EF0-435B-AE3D-53D38F144D1B

http://species-id.net/wiki/Apanteles_randallgarciai

Figs 195 , 323

Apanteles Rodriguez36 (Smith et al. 2006). Interim name provided by the authors.

#### Type locality.

COSTA RICA, Guanacaste, ACG, Potrerillos, Rio Azufrado, 95m, 10.81224, -85.54438.

#### Holotype.

♀ in CNC. Specimen labels: 1. DHJPAR0003989.

#### Paratypes.

64 ♀, 45 ♂ (BMNH, CNC, INBIO, INHS, NMNH). COSTA RICA: ACG database codes: See Appendix 2 for detailed label data.

#### Description.

**Female.** Metatibia color (outer face): entirely or mostly (>0.7 metatibia length) dark brown to black, with yellow to white coloration usually restricted to anterior 0.2 or less. Fore wing veins color: veins C+Sc+R and R1 with brown coloration restricted narrowly to borders, interior area of those veins and pterostigma (and sometimes veins r, 2RS and 2M) transparent or white; other veins mostly transparent. Antenna length/body length: antenna about as long as body (head to apex of metasoma); if slightly shorter, at least extending beyond anterior 0.7 metasoma length. Body length (head to apex of metasoma): 2.1–2.2 mm or 2.3–2.4 mm. Fore wing length: 2.3–2.4 mm. Metafemur length/width: 2.6–2.7 or 2.8–2.9. Mediotergite 1 length/width at posterior margin: 2.1–2.2. Mediotergite 1 maximum width/width at posterior margin: 1.4–1.5. Ovipositor sheaths length/metafemur length: 0.8 or 0.9. Ovipositor sheaths length/metatibia length: 0.7 or 0.8.

#### Molecular data.

Sequences in BOLD: 34, barcode compliant sequences: 28.

#### Biology/ecology.

Gregarious (Fig. 323). Hosts: Hesperiidae, *Phocides belus*, *Phocides pigmalion* DHJ02, *Phocides* Warren01.

#### Distribution.

Costa Rica, ACG.

#### Etymology.

We dedicate this species to Randall García in recognition of his key role in the founding of ACG and subsequent diligent efforts for the administration of INBio, Costa Rica’s Instituto Nacional de Biodiversidad.

### Apanteles randallmartinezi

Fernández-Triana sp. n.

http://zoobank.org/974C43B7-E8A3-416E-A02E-8856B12D3141

http://species-id.net/wiki/Apanteles_randallmartinezi

Figs 145 , 298

#### Type locality.

COSTA RICA, Alajuela, ACG, Sector Rincon Rain Forest, Quebrada Escondida, 420m, 10.89928, -85.27486.

#### Holotype.

♀ in CNC. Specimen labels: 1. DHJPAR0038254. 2. Voucher: D.H.Janzen & W.Hallwachs, DB: http://janzen.sas.upenn.edu, Area de Conservación Guanacaste, COSTA RICA, 09-SRNP-42777.

#### Paratypes.

1 ♀ (CNC). COSTA RICA: Guanacaste, ACG database code: DHJPAR0038256.

#### Description.

**Female.** Body color: body mostly dark except for some sternites which may be pale. Antenna color: scape, pedicel, and flagellum dark. Coxae color (pro-, meso-, metacoxa): pale, dark, dark. Femora color (pro-, meso-, metafemur): pale, anteriorly dark/posteriorly pale, mostly dark but anterior 0.2 or less pale. Tibiae color (pro-, meso-, metatibia): pale, pale, mostly pale but with posterior 0.2 or less dark. Tegula and humeral complex color: tegula pale, humeral complex half pale/half dark. Pterostigma color: mostly dark, with small pale area centrally. Fore wing veins color: partially pigmented (a few veins may be dark but most are pale). Antenna length/body length: antenna about as long as body (head to apex of metasoma); if slightly shorter, at least extending beyond anterior 0.7 metasoma length. Body in lateral view: not distinctly flattened dorso–ventrally. Body length (head to apex of metasoma): 3.3–3.4 mm or 3.7–3.8 mm. Fore wing length: 3.3–3.4 mm or 3.5–3.6 mm. Ocular–ocellar line/posterior ocellus diameter: 2.0–2.2. Interocellar distance/posterior ocellus diameter: 1.7–1.9. Antennal flagellomerus 2 length/width: 2.9–3.1. Antennal flagellomerus 14 length/width: 1.4–1.6. Length of flagellomerus 2/length of flagellomerus 14: 2.3–2.5. Tarsal claws: simple (?). Metafemur length/width: 3.4–3.5. Metatibia inner spur length/metabasitarsus length: 0.4–0.5. Anteromesoscutum: mostly with deep, dense punctures (separated by less than 2.0 × its maximum diameter). Mesoscutellar disc: mostly punctured. Number of pits in scutoscutellar sulcus: 7 or 8. Maximum height of mesoscutellum lunules/maximum height of lateral face of mesoscutellum: 0.4–0.5. Propodeum areola: completely defined by carinae, including transverse carina extending to spiracle. Propodeum background sculpture: partly sculptured, especially on anterior 0.5. Mediotergite 1 length/width at posterior margin: 2.0–2.2. Mediotergite 1 shape: slightly widening from anterior margin to 0.7–0.8 mediotergite length (where maximum width is reached), then narrowing towards posterior margin. Mediotergite 1 sculpture: mostly sculptured, excavated area centrally with transverse striation inside and/or a polished knob centrally on posterior margin of mediotergite. Mediotergite 2 width at posterior margin/length: 4.0–4.3. Mediotergite 2 sculpture: with some sculpture, mostly near posterior margin. Outer margin of hypopygium: with a wide, medially folded, transparent, semi–desclerotized area; usually with 4 or more pleats. Ovipositor thickness: anterior width at most 2.0 × posterior width (beyond ovipositor constriction). Ovipositor sheaths length/metatibial length: 1.0–1.1. Length of fore wing veins r/2RS: 2.3 or more. Length of fore wing veins 2RS/2M: 1.7–1.8. Length of fore wing veins 2M/(RS+M)b: 0.5–0.6. Pterostigma length/width: 3.1–3.5. Point of insertion of vein r in pterostigma: clearly beyond half way point length of pterostigma. Angle of vein r with fore wing anterior margin: clearly outwards, inclined towards fore wing apex. Shape of junction of veins r and 2RS in fore wing: distinctly but not strongly angled.

**Male.** Unknown.

#### Molecular data.

Sequences in BOLD: 5, barcode compliant sequences: 5.

#### Biology/ecology.

Solitary (Fig. 298). Host: Pyralidae, *Tancoa crinita* DHJ04, *Tancoa crinita* DHJ03.

#### Distribution.

Costa Rica, ACG.

#### Etymology.

We dedicate this species to Randall Martínez in recognition of his diligent efforts for the ACG Programa de Sectores.

### Apanteles raulacevedoi

Fernández-Triana sp. n.

http://zoobank.org/AAB83378-6D17-4A75-9EF4-79BBD9C51C43

http://species-id.net/wiki/Apanteles_raulacevedoi

Figs 81 , 263

Apanteles Rodriguez49. Smith et al. (2008). Interim name provided by the authors.

#### Type locality.

COSTA RICA, Alajuela, ACG, Sector San Cristobal, Potrero Argentina, 520m, 10.89021, -85.38803.

#### Holotype.

♀ in CNC. Specimen labels: 1. DHJPAR0039746. 2. Voucher: D.H.Janzen & W.Hallwachs, DB: http://janzen.sas.upenn.edu, Area de Conservación Guanacaste, COSTA RICA, 09-SRNP-5283.

#### Paratypes.

8 ♀, 1 ♂ (BMNH, CNC, INBIO, INHS, NMNH). COSTA RICA, ACG database codes: DHJPAR0013118, DHJPAR0024681, DHJPAR0024706, DHJPAR0034179, DHJPAR0035491, DHJPAR0038305, DHJPAR0038311, DHJPAR0038312, DHJPAR0039034.

#### Description.

**Female.** Body color: head dark, mesosoma dark with parts of axillar complex pale, metasoma with some mediotergites, most laterotergites, sternites, and/or hypopygium pale. Antenna color: scape, pedicel, and flagellum pale. Coxae color (pro-, meso-, metacoxa): pale, pale, pale. Femora color (pro-, meso-, metafemur): pale, pale, pale. Tibiae color (pro-, meso-, metatibia): pale, pale, pale. Tegula and humeral complex color: both pale. Pterostigma color: dark. Fore wing veins color: mostly dark (a few veins may be unpigmented). Antenna length/body length: antenna about as long as body (head to apex of metasoma); if slightly shorter, at least extending beyond anterior 0.7 metasoma length. Body in lateral view: not distinctly flattened dorso–ventrally. Body length (head to apex of metasoma): 2.7–2.8 mm or 2.9–3.0 mm. Fore wing length: 2.9–3.0 mm or 3.1–3.2 mm. Ocular–ocellar line/posterior ocellus diameter: 2.0–2.2. Interocellar distance/posterior ocellus diameter: 1.7–1.9. Antennal flagellomerus 2 length/width: 2.3–2.5. Antennal flagellomerus 14 length/width: 1.1–1.3. Length of flagellomerus 2/length of flagellomerus 14: 2.3–2.5. Tarsal claws: with single basal spine–like seta. Metafemur length/width: 2.8–2.9. Metatibia inner spur length/metabasitarsus length: 0.4–0.5. Anteromesoscutum: mostly smooth or with shallow sparse punctures, except for anterior 0.3 where it has deeper and/or denser punctures. Mesoscutellar disc: mostly smooth. Number of pits in scutoscutellar sulcus: 5 or 6 or 7 or 8. Maximum height of mesoscutellum lunules/maximum height of lateral face of mesoscutellum: 0.4–0.5. Propodeum areola: completely defined by carinae, including transverse carina extending to spiracle. Propodeum background sculpture: mostly sculptured. Mediotergite 1 length/width at posterior margin: 2.3–2.5. Mediotergite 1 shape: mostly parallel–sided for 0.5–0.7 of its length, then narrowing posteriorly so mediotergite anterior width >1.1 × posterior width. Mediotergite 1 sculpture: mostly sculptured, excavated area centrally with transverse striation inside and/or a polished knob centrally on posterior margin of mediotergite. Mediotergite 2 width at posterior margin/length: 3.6–3.9. Mediotergite 2 sculpture: mostly smooth. Outer margin of hypopygium: with a wide, medially folded, transparent, semi–desclerotized area; usually with 4 or more pleats. Ovipositor thickness: about same width throughout its length. Ovipositor sheaths length/metatibial length: 0.8–0.9. Length of fore wing veins r/2RS: 1.4–1.6. Length of fore wing veins 2RS/2M: 1.4–1.6. Length of fore wing veins 2M/(RS+M)b: 0.7–0.8. Pterostigma length/width: 3.1–3.5. Point of insertion of vein r in pterostigma: about half way point length of pterostigma. Angle of vein r with fore wing anterior margin: clearly outwards, inclined towards fore wing apex. Shape of junction of veins r and 2RS in fore wing: strongly angulated, sometimes with a knob.

**Male.** As in female, but with metacoxae dark brown and tergites darker in coloration.

#### Molecular data.

Sequences in BOLD: 51, barcode compliant sequences: 51, haplotypes: 2–3.

#### Biology/ecology.

Solitary (Fig. 263). Hosts: 25 assorted species of Crambidae, Elachistidae, Gelechiidae and many malaise-trapped; holotype is reared from Gelechiidae, gelJanzen01 Janzen235.

#### Distribution.

Costa Rica, ACG.

#### Comments.

There are some minor differences among specimens with respect to barcode clustering, morphology, and hosts, but all females in good condition belong to one morphotype; we predict that once this species is better understood, there will be a complex of species included under this name.

#### Etymology.

We dedicate this species to Raúl Acevedo in recognition of his diligent efforts for the ACG Programa de Seguridad.

### Apanteles raulsolorsanoi

Fernández-Triana sp. n.

http://zoobank.org/B2E08A17-6E4D-4599-92FC-74A495391C24

http://species-id.net/wiki/Apanteles_raulsolorsanoi

Fig. 196

Apanteles Rodriguez77 (Smith et al. 2006). Interim name provided by the authors.

#### Type locality.

COSTA RICA, Guanacaste, ACG, Sector Santa Rosa, Bosque San Emilio, 300m, 10.84389, -85.61384.

#### Holotype.

♀ in CNC. Specimen labels: 1. DHJPAR0001573.

#### Paratypes.

29 ♀, 2 ♂ (BMNH, CNC, INBIO, INHS, NMNH). COSTA RICA: ACG database codes: DHJPAR0002702.

#### Description.

**Female.** Metatibia color (outer face): entirely or mostly (>0.7 metatibia length) dark brown to black, with yellow to white coloration usually restricted to anterior 0.2 or less. Fore wing veins color: veins C+Sc+R and R1 with brown coloration restricted narrowly to borders, interior area of those veins and pterostigma (and sometimes veins r, 2RS and 2M) transparent or white; other veins mostly transparent. Antenna length/body length: antenna about as long as body (head to apex of metasoma); if slightly shorter, at least extending beyond anterior 0.7 metasoma length. Body length (head to apex of metasoma): 2.3–2.4 mm. Fore wing length: 2.5–2.6 mm. Metafemur length/width: 2.8–2.9 or 3.0–3.1. Mediotergite 1 length/width at posterior margin: 2.1–2.2, rarely 2.3–2.4. Mediotergite 1 maximum width/width at posterior margin: 1.4–1.5, rarely 1.6–1.7. Ovipositor sheaths length/metafemur length: 0.8, 0.9 or 1.0. Ovipositor sheaths length/metatibia length: 0.7 or 0.8.

#### Molecular data.

Sequences in BOLD: 1, barcode compliant sequences: 1.

#### Biology/ecology.

Gregarious. Host: Hesperiidae, *Narcosius helen*.

#### Distribution.

Costa Rica, ACG.

#### Etymology.

We dedicate this species to Raúl Solorsano in recognition of his support for the founding of ACG and unwavering support for conservation in Costa Rica.

### Apanteles rhomboidalis

(Ashmead, 1900)

http://species-id.net/wiki/Apanteles_rhomboidalis

Fig. 102

Urogaster rhomboidalis Ashmead, 1900: 290.
Apanteles rhomboidalis (Ashmead). Transferred by Szépligeti (1904: 111).

#### Type locality.

ST. VINCENT, Lesser Antilles.

#### Holotype.

♀, BMNH (examined).

#### Description.

**Female.** Body color: body mostly dark except for some sternites which may be pale. Antenna color: scape, pedicel, and flagellum pale. Coxae color (pro-, meso-, metacoxa): pale, pale, partially pale/partially dark. Femora color (pro-, meso-, metafemur): pale, pale, anteriorly pale/posteriorly dark. Tibiae color (pro-, meso-, metatibia): pale, pale, anteriorly pale/posteriorly dark. Tegula and humeral complex color: both pale. Pterostigma color: mostly dark, with small pale area centrally. Fore wing veins color: mostly dark (a few veins may be unpigmented). Antenna length/body length: antenna about as long as body (head to apex of metasoma); if slightly shorter, at least extending beyond anterior 0.7 metasoma length. Body in lateral view: not distinctly flattened dorso–ventrally. Body length (head to apex of metasoma): 2.1–2.2 mm. Fore wing length: 2.1–2.2 mm. Ocular–ocellar line/posterior ocellus diameter: 1.7–1.9. Interocellar distance/posterior ocellus diameter: 2.0–2.2. Antennal flagellomerus 2 length/width: 2.6–2.8. Antennal flagellomerus 14 length/width: 1.1–1.3. Length of flagellomerus 2/length of flagellomerus 14: 2.3–2.5. Tarsal claws: simple. Metafemur length/width: 3.2–3.3. Metatibia inner spur length/metabasitarsus length: 0.4–0.5. Anteromesoscutum: mostly with shallow, dense punctures (separated by less than 2.0 × its maximum diameter). Mesoscutellar disc: mostly smooth. Number of pits in scutoscutellar sulcus: 11 or 12. Maximum height of mesoscutellum lunules/maximum height of lateral face of mesoscutellum: 0.4–0.5. Propodeum areola: partially defined by carinae on posterior 0.3–0.5 of its length, widely open anteriorly. Propodeum background sculpture: mostly sculptured. Mediotergite 1 length/width at posterior margin: 2.6–2.8. Mediotergite 1 shape: mostly parallel–sided for 0.5–0.7 of its length, then narrowing posteriorly so mediotergite anterior width >1.1 × posterior width. Mediotergite 1 sculpture: mostly sculptured, excavated area centrally with transverse striation inside and/or a polished knob centrally on posterior margin of mediotergite. Mediotergite 2 width at posterior margin/length: 3.6–3.9. Mediotergite 2 sculpture: more or less fully sculptured, with longitudinal striation. Outer margin of hypopygium: with a wide, medially folded, transparent, semi–desclerotized area; usually with 4 or more pleats. Ovipositor thickness: about same width throughout its length. Ovipositor sheaths length/metatibial length: 1.4–1.5. Length of fore wing veins r/2RS: 1.1–1.3. Length of fore wing veins 2RS/2M: 1.4–1.6. Length of fore wing veins 2M/(RS+M)b: 0.9–1.0. Pterostigma length/width: 2.6–3.0. Point of insertion of vein r in pterostigma: clearly beyond half way point length of pterostigma. Angle of vein r with fore wing anterior margin: more or less perpendicular to fore wing margin. Shape of junction of veins r and 2RS in fore wing: distinctly but not strongly angled.

#### Molecular data.

No molecular data available for this species.

#### Biology/ecology.

Unknown.

#### Distribution.

St. Vincent. There is no suggestion that this species occurs in ACG or even Costa Rica.

### Apanteles ricardocaleroi

Fernández-Triana sp. n.

http://zoobank.org/B93A5FA0-6A38-4F51-8C75-B023585F22DF

http://species-id.net/wiki/Apanteles_ricardocaleroi

Figs 198 , 324

Apanteles Rodriguez21 (Smith et al. 2006). Interim name provided by the authors.

#### Type locality.

COSTA RICA, Guanacaste, ACG, Sector Santa Rosa, Alacran, 260m, 10.89249, -85.60336.

#### Holotype.

♀ in CNC. Specimen labels: 1. DHJPAR0001646.

#### Paratypes.

12 ♀, 4 ♂ (BMNH, CNC, INBIO, INHS, NMNH). COSTA RICA: ACG database codes: DHJPAR0001646, 03-SRNP-8250.

#### Description.

**Female.** Metatibia color (outer face): entirely or mostly (>0.7 metatibia length) dark brown to black, with yellow to white coloration usually restricted to anterior 0.2 or less. Fore wing veins color: veins C+Sc+R and R1 with brown coloration restricted narrowly to borders, interior area of those veins and pterostigma (and sometimes veins r, 2RS and 2M) transparent or white; other veins mostly transparent. Antenna length/body length: antenna about as long as body (head to apex of metasoma); if slightly shorter, at least extending beyond anterior 0.7 metasoma length. Body length (head to apex of metasoma): 2.0 mm or less. Fore wing length: 2.1–2.2 mm, rarely 2.3–2.4 mm. Metafemur length/width: 3.0–3.1 or 3.2–3.3. Mediotergite 1 length/width at posterior margin: 2.3–2.4, 2.5–2.6 or 2.7–2.8. Mediotergite 1 maximum width/width at posterior margin: 1.2–1.3 or 1.4–1.5. Ovipositor sheaths length/metafemur length: 0.8, 0.9, 1.0 or 1.1. Ovipositor sheaths length/metatibia length: 0.7, 0.8 or 0.9.

#### Molecular data.

Sequences in BOLD: 6, barcode compliant sequences: 5.

#### Biology/ecology.

Gregarious (Fig. 324). Hosts: Hesperiidae, *Aguna asander*, *Aguna panama*, *Aguna arunce hypozonius*.

#### Distribution.

Costa Rica, ACG.

#### Etymology.

We dedicate this species to Ricardo Calero in recognition of his diligent efforts for the ACG Programa de Parataxónomos and Estación Biológica Quica of ACG.

### Apanteles robertmontanoi

Fernández-Triana sp. n.

http://zoobank.org/A0696DC4-6D5C-4075-84FD-391B5A98DDC9

http://species-id.net/wiki/Apanteles_robertmontanoi

Fig. 154

#### Type locality.

COSTA RICA, Alajuela, ACG, Sector San Cristobal, Rio Blanco Abajo, 500m, 10.90037, -85.37254.

#### Holotype.

♀ in CNC. Specimen labels: 1. San Gerardo, Rio Blanco Abajo, 21-27 August 2007. 2. DHJPAR0024890.

#### Paratypes.

1 ♀ (CNC). COSTA RICA: Guanacaste, ACG database code: DHJPAR0025089.

#### Description.

**Female.** Body color: body mostly dark except for some sternites which may be pale. Antenna color: scape, pedicel, and flagellum dark. Coxae color (pro-, meso-, metacoxa): pale, pale, partially pale/partially dark. Femora color (pro-, meso-, metafemur): pale, pale, anteriorly pale/posteriorly dark. Tibiae color (pro-, meso-, metatibia): pale, pale, mostly dark but anterior 0.2 or less pale. Tegula and humeral complex color: both pale. Pterostigma color: dark. Fore wing veins color: mostly dark (a few veins may be unpigmented). Antenna length/body length: antenna about as long as body (head to apex of metasoma); if slightly shorter, at least extending beyond anterior 0.7 metasoma length. Body in lateral view: not distinctly flattened dorso–ventrally. Body length (head to apex of metasoma): 2.3–2.4 mm. Fore wing length: 2.3–2.4 mm. Ocular–ocellar line/posterior ocellus diameter: 2.0–2.2. Interocellar distance/posterior ocellus diameter: 1.7–1.9. Antennal flagellomerus 2 length/width: 2.9–3.1. Antennal flagellomerus 14 length/width: 1.1–1.3. Length of flagellomerus 2/length of flagellomerus 14: 2.6–2.8. Tarsal claws: simple. Metafemur length/width: 3.2–3.3. Metatibia inner spur length/metabasitarsus length: 0.4–0.5. Anteromesoscutum: mostly with deep, dense punctures (separated by less than 2.0 × its maximum diameter). Mesoscutellar disc: mostly punctured. Number of pits in scutoscutellar sulcus: 5 or 6. Maximum height of mesoscutellum lunules/maximum height of lateral face of mesoscutellum: 0.4–0.5. Propodeum areola: completely defined by carinae, including transverse carina extending to spiracle. Propodeum background sculpture: mostly sculptured. Mediotergite 1 length/width at posterior margin: 4.1 or more. Mediotergite 1 shape: mostly parallel–sided for 0.5–0.7 of its length, then narrowing posteriorly so mediotergite anterior width >1.1 × posterior width. Mediotergite 1 sculpture: mostly sculptured, excavated area centrally with transverse striation inside and/or a polished knob centrally on posterior margin of mediotergite. Mediotergite 2 width at posterior margin/length: 3.2–3.5. Mediotergite 2 sculpture: with some sculpture, mostly near posterior margin. Outer margin of hypopygium: with a wide, medially folded, transparent, semi–desclerotized area; usually with 4 or more pleats. Ovipositor thickness: about same width throughout its length. Ovipositor sheaths length/metatibial length: 1.0–1.1. Length of fore wing veins r/2RS: 1.4–1.6. Length of fore wing veins 2RS/2M: 1.1–1.3. Length of fore wing veins 2M/(RS+M)b: 0.9–1.0. Pterostigma length/width: 3.1–3.5. Point of insertion of vein r in pterostigma: about half way point length of pterostigma. Angle of vein r with fore wing anterior margin: more or less perpendicular to fore wing margin. Shape of junction of veins r and 2RS in fore wing: distinctly but not strongly angled.

**Male.** Unknown.

#### Molecular data.

Sequences in BOLD: 2, barcode compliant sequences: 2.

#### Biology/ecology.

Malaise-trapped.

#### Distribution.

Costa Rica, ACG.

#### Comments.

This species is characterized by mediotergite 1 length more than 4.5 × its posterior width; vannal lobe straight and setose (with slightly shorter and sparser setae centrally); metacoxa and metafemur partially yellow and partially dark brown. Molecular data supports the species as divergent (Fig. 1). *Apanteles robertmontanoi* illustrates the present difficulties of separating the genera *Apanteles* and *Dolichogenidea*. Most of its morphological characters are those of a typical *Apanteles* – including punctures near the posterior margin of the anteromesoscutum, that tend to fuse with each other, sensu Mason’s (1981) concept of the genus – and the molecular analysis also clusters the species with many other Mesoamerican species of *Apanteles*. However the vannal lobe is closer to (but slightly different) from that of *Dolichogenidea* species. Solving the limits (or lack thereof) between those two genera is beyond the scope of this paper, thus we describe the species as *Apanteles* for the time being.

#### Etymology.

We dedicate this species to Robert Montano in recognition of his diligent efforts for the ACG Programa de Sectores.

### Apanteles robertoespinozai

Fernández-Triana sp. n.

http://zoobank.org/D462DD40-CF30-41A2-AF4A-143083B26B78

http://species-id.net/wiki/Apanteles_robertoespinozai

Figs 98 , 275

Apanteles Rodriguez14 (Smith et al. 2006). Interim name provided by the authors.

#### Type locality.

COSTA RICA, Alajuela, ACG, Sector San Cristobal, Rio Blanco Abajo, 500 meters, 10.90037, -85.37254.

#### Holotype.

♀ in CNC. Specimen labels: 1. DHJPAR0001549.

#### Paratypes.

29 ♀, 5 ♂ (BMNH, CNC, INBIO, INHS, NMNH). COSTA RICA, ACG database codes: DHJPAR0005005, 01-SRNP-3458, 04-SRNP-31136.

#### Description.

**Female.** Body color: body mostly dark except for some sternites which may be pale. Antenna color: scape and/or pedicel pale, flagellum dark. Coxae color (pro-, meso-, metacoxa): pale, pale, partially pale/partially dark. Femora color (pro-, meso-, metafemur): pale, pale, pale or pale, pale, mostly pale but with dark area dorsally. Tibiae color (pro-, meso-, metatibia): pale, pale, anteriorly pale/posteriorly dark. Tegula and humeral complex color: both pale. Pterostigma color: dark with pale spot at base. Fore wing veins color: mostly dark (a few veins may be unpigmented). Antenna length/body length: antenna about as long as body (head to apex of metasoma); if slightly shorter, at least extending beyond anterior 0.7 metasoma length. Body in lateral view: not distinctly flattened dorso–ventrally. Body length (head to apex of metasoma): 2.3–2.4 mm. Fore wing length: 2.5–2.6 mm. Ocular–ocellar line/posterior ocellus diameter: 2.6 or more. Interocellar distance/posterior ocellus diameter: 1.4–1.6. Antennal flagellomerus 2 length/width: 2.0–2.2. Antennal flagellomerus 14 length/width: 1.1–1.3. Length of flagellomerus 2/length of flagellomerus 14: 1.7–1.9. Tarsal claws: simple. Metafemur length/width: 2.8–2.9. Metatibia inner spur length/metabasitarsus length: 0.4–0.5. Anteromesoscutum: mostly with shallow, sparse punctures (separated by more than 2.0 × its maximum diameter). Mesoscutellar disc: mostly smooth. Number of pits in scutoscutellar sulcus: 9 or 10. Maximum height of mesoscutellum lunules/maximum height of lateral face of mesoscutellum: 0.2–0.3. Propodeum areola: completely defined by carinae, including transverse carina extending to spiracle. Propodeum background sculpture: partly sculptured, especially on anterior 0.5. Mediotergite 1 length/width at posterior margin: 4.1 or more. Mediotergite 1 shape: clearly narrowing towards posterior margin. Mediotergite 1 sculpture: more or less fully sculptured with longitudinal striation. Mediotergite 2 width at posterior margin/length: 3.2–3.5. Mediotergite 2 sculpture: mostly smooth. Outer margin of hypopygium: inflexible (without a folded, transparent, semi–desclerotized area); with no pleats visible. Ovipositor thickness: anterior width at most 2.0 × posterior width (beyond ovipositor constriction). Ovipositor sheaths length/metatibial length: 0.4–0.5. Length of fore wing veins r/2RS: 1.1–1.3. Length of fore wing veins 2RS/2M: 0.9–1.0. Length of fore wing veins 2M/(RS+M)b: 1.1–1.3. Pterostigma length/width: 3.6 or more. Point of insertion of vein r in pterostigma: about half way point length of pterostigma. Angle of vein r with fore wing anterior margin: clearly outwards, inclined towards fore wing apex. Shape of junction of veins r and 2RS in fore wing: strongly angulated, sometimes with a knob.

**Male.** As in female but with darker metacoxae and metafemur.

#### Molecular data.

Sequences in BOLD: 22, barcode compliant sequences: 22.

#### Biology/ecology.

Gregarious (Fig. 275). Hosts: Crambidae, four species of *Phostria*, *Desmia* Solis19, *Syllepte* Solis21.

#### Distribution.

Costa Rica, ACG.

#### Etymology.

We dedicate this species to Roberto Espinoza in recognition of his diligent efforts for the ACG Programa de Parataxónomos and the plant inventory of ACG.

### Apanteles robertovargasi

Fernández-Triana sp. n.

http://zoobank.org/A0071E4E-4301-4311-BF88-FBEA13420382

http://species-id.net/wiki/Apanteles_robertovargasi

Fig. 103

#### Type locality.

COSTA RICA, Alajuela, ACG, Sector San Cristobal, Rio Blanco Abajo, 500m, 10.90037, -85.37254.

#### Holotype.

♀ in CNC. Specimen labels: 1. DHJPAR0026474. 2. San Gerardo, Rio Blanco Abajo, 24-30 Jan 2008.

#### Description.

**Female.** Body color: body mostly dark except for some sternites which may be pale. Antenna color: scape, pedicel, and flagellum pale. Coxae color (pro-, meso-, metacoxa): pale, pale, partially pale/partially dark. Femora color (pro-, meso-, metafemur): pale, pale, pale. Tibiae color (pro-, meso-, metatibia): pale, pale, pale. Tegula and humeral complex color: both pale. Pterostigma color: dark. Fore wing veins color: mostly dark (a few veins may be unpigmented). Antenna length/body length: antenna shorter than body (head to apex of metasoma), not extending beyond anterior 0.7 metasoma length. Body in lateral view: not distinctly flattened dorso–ventrally. Body length (head to apex of metasoma): 3.3–3.4 mm. Fore wing length: 3.5–3.6 mm. Ocular–ocellar line/posterior ocellus diameter: 1.7–1.9. Interocellar distance/posterior ocellus diameter: 1.7–1.9. Antennal flagellomerus 2 length/width: 2.6–2.8. Antennal flagellomerus 14 length/width: 1.1–1.3. Length of flagellomerus 2/length of flagellomerus 14: 2.6–2.8. Tarsal claws: simple. Metafemur length/width: 2.8–2.9. Metatibia inner spur length/metabasitarsus length: 0.6–0.7. Anteromesoscutum: mostly with deep, dense punctures (separated by less than 2.0 × its maximum diameter). Mesoscutellar disc: with punctures near margins, central part mostly smooth. Number of pits in scutoscutellar sulcus: 9 or 10. Maximum height of mesoscutellum lunules/maximum height of lateral face of mesoscutellum: 0.4–0.5. Propodeum areola: completely defined by carinae, including transverse carina extending to spiracle. Propodeum background sculpture: mostly sculptured. Mediotergite 1 length/width at posterior margin: 1.1–1.3. Mediotergite 1 shape: more or less parallel–sided. Mediotergite 1 sculpture: mostly sculptured, excavated area centrally with transverse striation inside and/or a polished knob centrally on posterior margin of mediotergite. Mediotergite 2 width at posterior margin/length: 3.2–3.5. Mediotergite 2 sculpture: more or less fully sculptured, with longitudinal striation. Outer margin of hypopygium: with a wide, medially folded, transparent, semi–desclerotized area; usually with 4 or more pleats. Ovipositor thickness: about same width throughout its length. Ovipositor sheaths length/metatibial length: 1.0–1.1. Length of fore wing veins r/2RS: 1.7–1.9. Length of fore wing veins 2RS/2M: 1.1–1.3. Length of fore wing veins 2M/(RS+M)b: 0.9–1.0. Pterostigma length/width: 2.1–2.5. Point of insertion of vein r in pterostigma: clearly beyond half way point length of pterostigma. Angle of vein r with fore wing anterior margin: clearly outwards, inclined towards fore wing apex. Shape of junction of veins r and 2RS in fore wing: distinctly but not strongly angled.

**Male.** Unknown.

#### Molecular data.

Sequences in BOLD: 1, barcode compliant sequences: 1.

#### Biology/ecology.

Malaise-trapped.

#### Distribution.

Costa Rica, ACG.

#### Etymology.

We dedicate this species to Roberto Vargas in recognition of his diligent efforts for the ACG Programa de Educacion Biológica.

### Apanteles rodrigogamezi

Fernández-Triana sp. n.

http://zoobank.org/306151B4-4BA9-40CF-886C-67331FAA780E

http://species-id.net/wiki/Apanteles_rodrigogamezi

Fig. 199

Apanteles Rodriguez35 (Smith et al. 2006). Interim name provided by the authors.

#### Type locality.

COSTA RICA, Guanacaste, ACG, Sector Del Oro, Meteorologico, 590m, 11.00199, -85.46166.

#### Holotype.

♀ in CNC. Specimen labels: 1. DHJPAR0001554.

#### Paratypes.

13 ♀, 4 ♂ (BMNH, CNC, INBIO, INHS, NMNH). COSTA RICA: ACG database code: DHJPAR0001554.

#### Description.

**Female.** Metatibia color (outer face): entirely or mostly (>0.7 metatibia length) dark brown to black, with yellow to white coloration usually restricted to anterior 0.2 or less. Fore wing veins color: veins C+Sc+R and R1 with brown coloration restricted narrowly to borders, interior area of those veins and pterostigma (and sometimes veins r, 2RS and 2M) transparent or white; other veins mostly transparent. Antenna length/body length: antenna about as long as body (head to apex of metasoma); if slightly shorter, at least extending beyond anterior 0.7 metasoma length. Body length (head to apex of metasoma): 2.5–2.6 mm. Fore wing length: 2.5–2.6 mm or 2.7–2.8 mm. Metafemur length/width: 2.8–2.9, 3.0–3.1, rarely 3.2–3.3. Mediotergite 1 length/width at posterior margin: 2.9 or more, rarely 2.7–2.8. Mediotergite 1 maximum width/width at posterior margin: 1.6–1.7 or 1.8–1.9. Ovipositor sheaths length/metafemur length: 1.0. Ovipositor sheaths length/metatibia length: 0.8 or 0.9.

#### Molecular data.

Sequences in BOLD: 2, barcode compliant sequences: 1.

#### Biology/ecology.

Gregarious. Hosts: Hesperiidae, *Bungalotis diophorus*.

#### Distribution.

Costa Rica, ACG.

#### Etymology.

We dedicate this species to Rodrigo Gámez in recognition of his enormous efforts in support of founding ACG, and founding and directing INBio, Costa Rica’s Instituto Nacional de Biodiveristy.

### Apanteles rogerblancoi

Fernández-Triana sp. n.

http://zoobank.org/C6E11B11-6B8B-464B-81BB-DF02997480F4

http://species-id.net/wiki/Apanteles_rogerblancoi

Fig. 155

#### Type locality.

COSTA RICA, Guanacaste, ACG, Sector Santa Rosa, Bosque San Emilio, 300m, 10.84389, -85.61384.

#### Holotype.

♀ in CNC. Specimen labels: 1. DHJPAR0024724.

#### Description.

**Female.** Body color: body mostly dark except for some sternites which may be pale. Antenna color: scape and/or pedicel pale, flagellum dark. Coxae color (pro-, meso-, metacoxa): pale, pale, dark. Femora color (pro-, meso-, metafemur): pale, pale, mostly pale but posterior 0.2 or less dark. Tibiae color (pro-, meso-, metatibia): pale, pale, mostly pale but with posterior 0.2 or less dark. Tegula and humeral complex color: both pale. Pterostigma color: dark with pale spot at base. Fore wing veins color: mostly dark (a few veins may be unpigmented). Antenna length/body length: antenna about as long as body (head to apex of metasoma); if slightly shorter, at least extending beyond anterior 0.7 metasoma length. Body in lateral view: not distinctly flattened dorso–ventrally. Body length (head to apex of metasoma): 2.7–2.8 mm. Fore wing length: 2.9–3.0 mm. Ocular–ocellar line/posterior ocellus diameter: 1.7–1.9. Interocellar distance/posterior ocellus diameter: 2.0–2.2. Antennal flagellomerus 2 length/width: 2.3–2.5. Antennal flagellomerus 14 length/width: 1.1–1.3. Length of flagellomerus 2/length of flagellomerus 14: 2.6–2.8. Tarsal claws: with single basal spine–like seta (?). Metafemur length/width: 3.0–3.1. Metatibia inner spur length/metabasitarsus length: 0.6–0.7. Anteromesoscutum: mostly with deep, dense punctures (separated by less than 2.0 × its maximum diameter). Mesoscutellar disc: mostly punctured. Number of pits in scutoscutellar sulcus: 9 or 10. Maximum height of mesoscutellum lunules/maximum height of lateral face of mesoscutellum: 0.4–0.5. Propodeum areola: completely defined by carinae, including transverse carina extending to spiracle. Propodeum background sculpture: mostly sculptured. Mediotergite 1 length/width at posterior margin: 2.0–2.2. Mediotergite 1 shape: mostly parallel–sided for 0.5–0.7 of its length, then narrowing posteriorly so mediotergite anterior width >1.1 × posterior width. Mediotergite 1 sculpture: mostly sculptured, excavated area centrally with transverse striation inside and/or a polished knob centrally on posterior margin of mediotergite. Mediotergite 2 width at posterior margin/length: 4.8 or more. Mediotergite 2 sculpture: with some sculpture, mostly near posterior margin. Outer margin of hypopygium: with a wide, medially folded, transparent, semi–desclerotized area; usually with 4 or more pleats. Ovipositor thickness: about same width throughout its length. Ovipositor sheaths length/metatibial length: 1.0–1.1. Length of fore wing veins r/2RS: 2.0–2.2. Length of fore wing veins 2RS/2M: 1.4–1.6. Length of fore wing veins 2M/(RS+M)b: 0.5–0.6. Pterostigma length/width: 2.6–3.0. Point of insertion of vein r in pterostigma: clearly beyond half way point length of pterostigma. Angle of vein r with fore wing anterior margin: clearly outwards, inclined towards fore wing apex. Shape of junction of veins r and 2RS in fore wing: distinctly but not strongly angled.

**Male.** Unknown.

#### Molecular data.

Sequences in BOLD: 6, barcode compliant sequences: 6.

#### Biology/ecology.

Malaise-trapped.

#### Distribution.

Costa Rica, ACG.

#### Comments.

This species is characterized by head fully black except for gena partially white, a unique feature among all known species of *Apanteles* worldwide. Molecular data also supports the species as divergent (Fig. 1).

#### Etymology.

We dedicate this species to Roger Blanco in recognition of his diligent efforts for the ACG Programa de Investigacion and ACG administration.

### Apanteles rolandoramosi

Fernández-Triana sp. n.

http://zoobank.org/E0586964-56B0-46D1-9686-CB2504175F27

http://species-id.net/wiki/Apanteles_rolandoramosi

Fig. 104

#### Type locality.

COSTA RICA, Guanacaste, ACG, Sector Santa Rosa, Bosque Humedo, 290m, 10.85145, -85.60801.

#### Holotype.

♀ in CNC. Specimen labels: 1. DHJPAR0013113. 2. Bosque Humedo, MT, 30.v-5.vi.2007, J. Rodriguez & A. R. Deans.

#### Paratypes.

3 ♀ (CNC, NMNH). COSTA RICA, ACG database codes: DHJPAR0012497, DHJPAR0012526, DHJPAR0012543.

#### Description.

**Female.** Body color: head dark, mesosoma dark with parts of axillar complex pale, metasoma with some mediotergites, most laterotergites, sternites, and/or hypopygium pale. Antenna color: scape and/or pedicel pale, flagellum dark. Coxae color (pro-, meso-, metacoxa): pale, pale, partially pale/partially dark. Femora color (pro-, meso-, metafemur): pale, pale, pale. Tibiae color (pro-, meso-, metatibia): pale, pale, pale. Tegula and humeral complex color: both pale. Pterostigma color: dark with pale spot at base. Fore wing veins color: mostly dark (a few veins may be unpigmented). Antenna length/body length: antenna shorter than body (head to apex of metasoma), not extending beyond anterior 0.7 metasoma length. Body in lateral view: not distinctly flattened dorso–ventrally. Body length (head to apex of metasoma): 2.5–2.6 mm or 2.7–2.8 mm. Fore wing length: 2.5–2.6 mm, 2.7–2.8 mm, rarely 2.9–3.0 mm. Ocular–ocellar line/posterior ocellus diameter: 2.0–2.2. Interocellar distance/posterior ocellus diameter: 1.7–1.9. Antennal flagellomerus 2 length/width: 2.0–2.2. Antennal flagellomerus 14 length/width: 1.1–1.3. Length of flagellomerus 2/length of flagellomerus 14: 2.0–2.2. Tarsal claws: with single basal spine–like seta. Metafemur length/width: 3.0–3.1. Metatibia inner spur length/metabasitarsus length: 0.4–0.5. Anteromesoscutum: mostly with deep, dense punctures (separated by less than 2.0 × its maximum diameter). Mesoscutellar disc: mostly punctured. Number of pits in scutoscutellar sulcus: 9 or 10. Maximum height of mesoscutellum lunules/maximum height of lateral face of mesoscutellum: 0.4–0.5. Propodeum areola: completely defined by carinae, including transverse carina extending to spiracle. Propodeum background sculpture: partly sculptured, especially on anterior 0.5. Mediotergite 1 length/width at posterior margin: 1.4–1.6. Mediotergite 1 shape: slightly widening from anterior margin to 0.7–0.8 mediotergite length (where maximum width is reached), then narrowing towards posterior margin. Mediotergite 1 sculpture: mostly sculptured, excavated area centrally with transverse striation inside and/or a polished knob centrally on posterior margin of mediotergite. Mediotergite 2 width at posterior margin/length: 3.6–3.9. Mediotergite 2 sculpture: more or less fully sculptured, with longitudinal striation. Outer margin of hypopygium: with a wide, medially folded, transparent, semi–desclerotized area; usually with 4 or more pleats. Ovipositor thickness: about same width throughout its length. Ovipositor sheaths length/metatibial length: 0.8–0.9 or 1.0–1.1. Length of fore wing veins r/2RS: 1.7–1.9. Length of fore wing veins 2RS/2M: 1.4–1.6. Length of fore wing veins 2M/(RS+M)b: 0.7–0.8. Pterostigma length/width: 2.6–3.0. Point of insertion of vein r in pterostigma: clearly beyond half way point length of pterostigma. Angle of vein r with fore wing anterior margin: clearly outwards, inclined towards fore wing apex. Shape of junction of veins r and 2RS in fore wing: distinctly but not strongly angled.

**Male.** Unknown.

#### Molecular data.

Sequences in BOLD: 5, barcode compliant sequences: 5, haplotypes: 1

#### Biology/ecology.

Malaise-trapped.

#### Distribution.

Costa Rica, ACG.

#### Etymology.

We dedicate this species to Rolando Ramos in recognition of his diligent efforts for the ACG Programa de Educacion Biológica.

### Apanteles rolandovegai

Fernández-Triana sp. n.

http://zoobank.org/C3748132-B1D3-44D1-BED8-C86DCDA669D7

http://species-id.net/wiki/Apanteles_rolandovegai

Figs 156 , 300

Apanteles Rodriguez115 (Smith et al. 2006). Interim name provided by the authors.

#### Type locality.

COSTA RICA, Guanacaste, ACG, Sector Santa Rosa, Bosque San Emilio, 300m, 10.84389, -85.61384.

#### Holotype.

♀ in CNC. Specimen labels: 1. COSTA RICA, Guanacaste, ACG, Sector Santa Rosa, Bosque San Emilio, 09.viii.1990, 300m, 10.84389, -85.61384, 90-SRNP-1863.

#### Paratypes.

8 ♀ (BMNH, CNC, INBIO, INHS, NMNH). COSTA RICA: ACG database codes: DHJPAR0038045.

#### Description.

**Female.** Body color: body mostly dark except for some sternites which may be pale. Antenna color: scape, pedicel, and flagellum pale (?). Coxae color (pro-, meso-, metacoxa): pale, pale, pale (?). Femora color (pro-, meso-, metafemur): pale, pale, dark (?). Tibiae color (pro-, meso-, metatibia): pale, pale, anteriorly pale/posteriorly dark. Tegula and humeral complex color: both pale. Pterostigma color: dark (?). Fore wing veins color: partially pigmented (a few veins may be dark but most are pale) (?). Antenna length/body length: antenna about as long as body (head to apex of metasoma); if slightly shorter, at least extending beyond anterior 0.7 metasoma length. Body in lateral view: not distinctly flattened dorso–ventrally. Body length (head to apex of metasoma): 2.1–2.2 mm. Fore wing length: 2.5–2.6 mm. Ocular–ocellar line/posterior ocellus diameter: 2.0–2.2. Interocellar distance/posterior ocellus diameter: 1.4–1.6. Antennal flagellomerus 2 length/width: 2.6–2.8. Antennal flagellomerus 14 length/width: 1.4–1.6. Length of flagellomerus 2/length of flagellomerus 14: 1.7–1.9. Tarsal claws: simple. Metafemur length/width: 2.8–2.9. Metatibia inner spur length/metabasitarsus length: 0.6–0.7. Anteromesoscutum: mostly with deep, dense punctures (separated by less than 2.0 × its maximum diameter). Mesoscutellar disc: mostly smooth. Number of pits in scutoscutellar sulcus: 7 or 8. Maximum height of mesoscutellum lunules/maximum height of lateral face of mesoscutellum: 0.6–0.7. Propodeum areola: completely defined by carinae, including transverse carina extending to spiracle. Propodeum background sculpture: partly sculptured, especially on anterior 0.5. Mediotergite 1 length/width at posterior margin: 1.7–1.9. Mediotergite 1 shape: slightly widening from anterior margin to 0.7–0.8 mediotergite length (where maximum width is reached), then narrowing towards posterior margin. Mediotergite 1 sculpture: mostly smooth. Mediotergite 2 width at posterior margin/length: 4.0–4.3. Mediotergite 2 sculpture: mostly smooth. Outer margin of hypopygium: with a medially folded, transparent, semi–desclerotized area; with 0–3 pleats visible (?). Ovipositor thickness: anterior width at most 2.0 × posterior width (beyond ovipositor constriction) (?). Ovipositor sheaths length/metatibial length: 0.8–0.9. Length of fore wing veins r/2RS: 1.7–1.9. Length of fore wing veins 2RS/2M: 1.1–1.3. Length of fore wing veins 2M/(RS+M)b: 0.7–0.8. Pterostigma length/width: 3.1–3.5. Point of insertion of vein r in pterostigma: clearly beyond half way point length of pterostigma. Angle of vein r with fore wing anterior margin: clearly outwards, inclined towards fore wing apex. Shape of junction of veins r and 2RS in fore wing: distinctly but not strongly angled.

**Male.** Unknown.

#### Molecular data.

Sequences in BOLD: 4, barcode compliant sequences: 4.

#### Biology/ecology.

Gregarious (Fig. 300). Hosts: Gelechiidae, four unidentified species.

#### Distribution.

Costa Rica, ACG.

#### Comments.

This species is characterized by mediotergite 1 completely smooth. Molecular data also supports the species as divergent (Fig. 1).

#### Etymology.

We dedicate this species to Rolando Vega in recognition of his diligent efforts for the ACG Programa de Educacion Biológica.

### Apanteles ronaldcastroi

Fernández-Triana sp. n.

http://zoobank.org/71D1C69C-1002-4446-8D5D-06E8A440372B

http://species-id.net/wiki/Apanteles_ronaldcastroi

Figs 117 , 283

#### Type locality.

COSTA RICA, Alajuela, ACG, Sector San Cristobal, Bosque Trampa Malaise, 815m, 10.86280, -85.38460.

#### Holotype.

♀ in CNC. Specimen labels: 1. San Gerardo: MT, San Cristobal, Date: 11–17 Apr-2008. 2. DHJPAR0027681.

#### Paratypes.

1 ♀ (CNC). COSTA RICA: Guanacaste, ACG database code: DHJPAR0038206.

#### Description.

**Female.** Body color: head dark, mesosoma dark with parts of axillar complex pale, metasoma with some mediotergites, most laterotergites, sternites, and/or hypopygium pale. Antenna color: scape and/or pedicel pale, flagellum dark. Coxae color (pro-, meso-, metacoxa): pale, pale, pale. Femora color (pro-, meso-, metafemur): pale, pale, anteriorly pale/posteriorly dark. Tibiae color (pro-, meso-, metatibia): pale, pale, anteriorly pale/posteriorly dark. Tegula and humeral complex color: both pale. Pterostigma color: dark. Fore wing veins color: mostly dark (a few veins may be unpigmented). Antenna length/body length: antenna about as long as body (head to apex of metasoma); if slightly shorter, at least extending beyond anterior 0.7 metasoma length. Body in lateral view: not distinctly flattened dorso–ventrally. Body length (head to apex of metasoma): 3.3–3.4 mm. Fore wing length: 3.5–3.6 mm. Ocular–ocellar line/posterior ocellus diameter: 1.7–1.9. Interocellar distance/posterior ocellus diameter: 1.7–1.9. Antennal flagellomerus 2 length/width: 2.0–2.2. Antennal flagellomerus 14 length/width: 1.4–1.6. Length of flagellomerus 2/length of flagellomerus 14: 2.3–2.5. Tarsal claws: with single basal spine–like seta. Metafemur length/width: 2.8–2.9. Metatibia inner spur length/metabasitarsus length: 0.4–0.5. Anteromesoscutum: mostly with deep, dense punctures (separated by less than 2.0 × its maximum diameter). Mesoscutellar disc: mostly punctured. Number of pits in scutoscutellar sulcus: 5 or 6. Maximum height of mesoscutellum lunules/maximum height of lateral face of mesoscutellum: 0.2–0.3. Propodeum areola: completely defined by carinae, including transverse carina extending to spiracle. Propodeum background sculpture: mostly sculptured. Mediotergite 1 length/width at posterior margin: 2.9–3.1. Mediotergite 1 shape: mostly parallel–sided for 0.5–0.7 of its length, then narrowing posteriorly so mediotergite anterior width >1.1 × posterior width. Mediotergite 1 sculpture: mostly sculptured, excavated area centrally with transverse striation inside and/or a polished knob centrally on posterior margin of mediotergite. Mediotergite 2 width at posterior margin/length: 2.4–2.7 or 2.8–3.1. Mediotergite 2 sculpture: with some sculpture, mostly near posterior margin. Outer margin of hypopygium: with a wide, medially folded, transparent, semi–desclerotized area; usually with 4 or more pleats. Ovipositor thickness: anterior width at most 2.0 × posterior width (beyond ovipositor constriction). Ovipositor sheaths length/metatibial length: 0.8–0.9 or 1.0–1.1. Length of fore wing veins r/2RS: 1.4–1.6. Length of fore wing veins 2RS/2M: 1.4–1.6. Length of fore wing veins 2M/(RS+M)b: 0.7–0.8. Pterostigma length/width: 3.1–3.5. Point of insertion of vein r in pterostigma: about half way point length of pterostigma. Angle of vein r with fore wing anterior margin: clearly outwards, inclined towards fore wing apex. Shape of junction of veins r and 2RS in fore wing: strongly angulated, sometimes with a knob.

**Male.** Unknown.

#### Molecular data.

Sequences in BOLD: 2, barcode compliant sequences: 2.

#### Biology/ecology.

Solitary (Fig. 283). Host: Crambidae. *Pilocrocis ramentalis*

#### Distribution.

Costa Rica, ACG.

#### Etymology.

We dedicate this species to Ronald Castro in recognition of his diligent efforts for the ACG Programa Forestal.

### Apanteles ronaldgutierrezi

Fernández-Triana sp. n.

http://zoobank.org/F3A9E5A1-4047-4E0A-8B74-46016797FE8C

http://species-id.net/wiki/Apanteles_ronaldgutierrezi

Figs 157 , 301

#### Type locality.

COSTA RICA, Guanacaste, ACG, Sector Pitilla, Colocho, 375m, 11.02367, -85.41884.

#### Holotype.

♀ in CNC. Specimen labels: 1. Voucher: D.H.Janzen & W.Hallwachs, DB: http://janzen.sas.upenn.edu, Area de Conservación Guanacaste, COSTA RICA, 09-SRNP-30308. 2. DHJPAR0035344.

#### Paratypes.

1 ♂ (CNC). COSTA RICA: Guanacaste, ACG database code: DHJPAR0034284.

#### Description.

**Female.** Body color: body mostly dark except for some sternites which may be pale. Antenna color: scape, pedicel, and flagellum dark. Coxae color (pro-, meso-, metacoxa): dark, dark, dark. Femora color (pro-, meso-, metafemur): pale, pale, mostly dark but with pale spot antero–ventrally. Tibiae color (pro-, meso-, metatibia): pale, pale, anteriorly pale/posteriorly dark. Tegula and humeral complex color: tegula pale, humeral complex dark. Pterostigma color: dark. Fore wing veins color: mostly dark (a few veins may be unpigmented). Antenna length/body length: antenna about as long as body (head to apex of metasoma); if slightly shorter, at least extending beyond anterior 0.7 metasoma length. Body in lateral view: not distinctly flattened dorso–ventrally. Body length (head to apex of metasoma): 2.5–2.6 mm. Fore wing length: 2.7–2.8 mm. Ocular–ocellar line/posterior ocellus diameter: 2.0–2.2. Interocellar distance/posterior ocellus diameter: 1.4–1.6. Antennal flagellomerus 2 length/width: 2.9–3.1. Antennal flagellomerus 14 length/width: 1.7–1.9. Length of flagellomerus 2/length of flagellomerus 14: 2.3–2.5. Tarsal claws: simple. Metafemur length/width: 3.4–3.5. Metatibia inner spur length/metabasitarsus length: 0.6–0.7. Anteromesoscutum: mostly with deep, dense punctures (separated by less than 2.0 × its maximum diameter). Mesoscutellar disc: with punctures near margins, central part mostly smooth. Number of pits in scutoscutellar sulcus: 7 or 8. Maximum height of mesoscutellum lunules/maximum height of lateral face of mesoscutellum: 0.4–0.5. Propodeum areola: completely defined by carinae, including transverse carina extending to spiracle. Propodeum background sculpture: mostly sculptured. Mediotergite 1 length/width at posterior margin: 2.0–2.2. Mediotergite 1 shape: slightly widening from anterior margin to 0.7–0.8 mediotergite length (where maximum width is reached), then narrowing towards posterior margin. Mediotergite 1 sculpture: mostly sculptured, excavated area centrally with transverse striation inside and/or a polished knob centrally on posterior margin of mediotergite. Mediotergite 2 width at posterior margin/length: 2.8–3.1. Mediotergite 2 sculpture: mostly smooth, with weak sculpture on anterior margin. Outer margin of hypopygium: with a wide, medially folded, transparent, semi–desclerotized area; usually with 4 or more pleats. Ovipositor thickness: about same width throughout its length. Ovipositor sheaths length/metatibial length: 0.6–0.7. Length of fore wing veins r/2RS: 1.7–1.9. Length of fore wing veins 2RS/2M: 1.4–1.6. Length of fore wing veins 2M/(RS+M)b: 0.7–0.8. Pterostigma length/width: 3.6 or more. Point of insertion of vein r in pterostigma: about half way point length of pterostigma. Angle of vein r with fore wing anterior margin: more or less perpendicular to fore wing margin. Shape of junction of veins r and 2RS in fore wing: distinctly but not strongly angled.

**Male.** As in female, except for a much less transverse mediotergite 2.

#### Molecular data.

Sequences in BOLD: 4, barcode compliant sequences: 4.

#### Biology/ecology.

Solitary (Fig. 301). Host: Choreutidae, *Zodia* Rota02.

#### Distribution.

Costa Rica, ACG.

#### Etymology.

We dedicate this species to Ronald Gutiérrez in recognition of his diligent efforts for the ACG Programa de Sectores.

### Apanteles ronaldmurilloi

Fernández-Triana sp. n.

http://zoobank.org/3657C609-7832-43A1-A976-7CABDB8B8345

http://species-id.net/wiki/Apanteles_ronaldmurilloi

Figs 19 , 221

Apanteles Rodriguez270. Interim name provided by the authors.

#### Type locality.

COSTA RICA, Guanacaste, ACG, Sector Pitilla, Pasmompa, 440m, 11.01926, -85.40997.

#### Holotype.

♀ in CNC. Specimen labels: 1. Costa Rica: Guanacaste, ACG, Sector Pitilla, Pasmompa, 16.ii.2007, 440m, 11.01926, -85.40997, DHJPAR0013020.

#### Paratypes.

13 ♀, 1 ♂ (BMNH, CNC, INBIO, INHS, NMNH). COSTA RICA, ACG database codes: DHJPAR0013020, 06-SRNP-58235, 07-SRNP-31204.

#### Description.

**Female.** Body color: body mostly dark except for some sternites which may be pale. Antenna color: scape, pedicel, and flagellum dark. Coxae color (pro-, meso-, metacoxa): dark, dark, dark. Femora color (pro-, meso-, metafemur): anteriorly dark/posteriorly pale, dark, dark. Tibiae color (pro-, meso-, metatibia): pale, pale, anteriorly pale/posteriorly dark. Tegula and humeral complex color: tegula pale, humeral complex half pale/half dark. Pterostigma color: mostly pale and/or transparent, with thin dark borders. Fore wing veins color: partially pigmented (a few veins may be dark but most are pale). Antenna length/body length: antenna about as long as body (head to apex of metasoma); if slightly shorter, at least extending beyond anterior 0.7 metasoma length. Body in lateral view: not distinctly flattened dorso–ventrally. Body length (head to apex of metasoma): 2.9–3.0 mm, 3.1–3.2 mm, rarely 2.5–2.6 mm. Fore wing length: 3.3–3.4 mm, rarely 2.5–2.6 mm. Ocular–ocellar line/posterior ocellus diameter: 2.0–2.2. Interocellar distance/posterior ocellus diameter: 1.7–1.9. Antennal flagellomerus 2 length/width: 2.6–2.8. Antennal flagellomerus 14 length/width: 1.4–1.6. Length of flagellomerus 2/length of flagellomerus 14: 2.0–2.2. Tarsal claws: with single basal spine–like seta. Metafemur length/width: 3.2–3.3. Metatibia inner spur length/metabasitarsus length: 0.4–0.5. Anteromesoscutum: mostly with deep, dense punctures (separated by less than 2.0 × its maximum diameter). Mesoscutellar disc: mostly punctured. Number of pits in scutoscutellar sulcus: 7 or 8. Maximum height of mesoscutellum lunules/maximum height of lateral face of mesoscutellum: 0.4–0.5. Propodeum areola: completely defined by carinae, including transverse carina extending to spiracle. Propodeum background sculpture: mostly sculptured. Mediotergite 1 length/width at posterior margin: 1.4–1.6. Mediotergite 1 shape: slightly widening from anterior margin to 0.7–0.8 mediotergite length (where maximum width is reached), then narrowing towards posterior margin. Mediotergite 1 sculpture: mostly sculptured, excavated area centrally with transverse striation inside and/or a polished knob centrally on posterior margin of mediotergite. Mediotergite 2 width at posterior margin/length: 3.2–3.5. Mediotergite 2 sculpture: mostly smooth. Outer margin of hypopygium: with a wide, medially folded, transparent, semi–desclerotized area; usually with 4 or more pleats. Ovipositor thickness: about same width throughout its length. Ovipositor sheaths length/metatibial length: 1.4–1.5, rarely 1.2–1.3. Length of fore wing veins r/2RS: 1.7–1.9. Length of fore wing veins 2RS/2M: 1.4–1.6. Length of fore wing veins 2M/(RS+M)b: 0.7–0.8. Pterostigma length/width: 3.6 or more. Point of insertion of vein r in pterostigma: clearly beyond half way point length of pterostigma. Angle of vein r with fore wing anterior margin: clearly outwards, inclined towards fore wing apex. Shape of junction of veins r and 2RS in fore wing: distinctly but not strongly angled.

**Male.** As in female but with darker legs and narrower mediotergite 1.

#### Molecular data.

Sequences in BOLD: 4, barcode compliant sequences: 4.

#### Biology/ecology.

Gregarious (Fig. 221). Host: Elachistidae, *Stenoma* Janzen07.

#### Distribution.

Costa Rica, ACG.

#### Etymology.

We dedicate this species to Ronald Murillo in recognition of his diligent efforts for the ACG Sector Marino.

### Apanteles ronaldnavarroi

Fernández-Triana sp. n.

http://zoobank.org/3ACB6DE2-C612-42F4-A50D-D9863E1A7A9D

http://species-id.net/wiki/Apanteles_ronaldnavarroi

Fig. 137

Apanteles Rodriguez53 (Smith et al. 2006). Interim name provided by the authors.

#### Type locality.

COSTA RICA, Guanacaste, ACG, Sector Santa Rosa, Casetilla, 250m, 10.87652, -85.58605.

#### Holotype.

♀ in CNC. Specimen labels: 1. COSTA RICA, Guanacaste, ACG, Sector Santa Rosa, Casetilla, 250m, 27.vii.1994, 10.87652, -85.58605, DHJPAR0005196.

#### Paratypes.

1 ♀, (CNC). COSTA RICA: Guanacaste, ACG database code: DHJPAR0002164.

#### Description.

**Female.** Body color: body mostly dark except for some sternites which may be pale. Antenna color: scape, pedicel, and flagellum dark. Coxae color (pro-, meso-, metacoxa): dark, dark, dark. Femora color (pro-, meso-, metafemur): anteriorly dark/posteriorly pale, dark, dark. Tibiae color (pro-, meso-, metatibia): pale, pale, anteriorly pale/posteriorly dark. Tegula and humeral complex color: both dark. Pterostigma color: dark with pale spot at base. Fore wing veins color: partially pigmented (a few veins may be dark but most are pale). Antenna length/body length: antenna about as long as body (head to apex of metasoma); if slightly shorter, at least extending beyond anterior 0.7 metasoma length. Body in lateral view: not distinctly flattened dorso–ventrally. Body length (head to apex of metasoma): 2.3–2.4 mm. Fore wing length: 2.3–2.4 mm. Ocular–ocellar line/posterior ocellus diameter: 2.0–2.2. Interocellar distance/posterior ocellus diameter: 1.7–1.9. Antennal flagellomerus 2 length/width: 3.2 or more. Antennal flagellomerus 14 length/width: 1.1–1.3. Length of flagellomerus 2/length of flagellomerus 14: 2.3–2.5. Tarsal claws: simple. Metafemur length/width: 2.8–2.9. Metatibia inner spur length/metabasitarsus length: 0.4–0.5. Anteromesoscutum: mostly with deep, dense punctures (separated by less than 2.0 × its maximum diameter). Mesoscutellar disc: mostly smooth. Number of pits in scutoscutellar sulcus: 7 or 8. Maximum height of mesoscutellum lunules/maximum height of lateral face of mesoscutellum: 0.6–0.7. Propodeum areola: completely defined by carinae, including transverse carina extending to spiracle. Propodeum background sculpture: partly sculptured, especially on anterior 0.5. Mediotergite 1 length/width at posterior margin: 2.0–2.2. Mediotergite 1 shape: slightly widening from anterior margin to 0.7–0.8 mediotergite length (where maximum width is reached), then narrowing towards posterior margin. Mediotergite 1 sculpture: more or less fully sculptured with longitudinal striation. Mediotergite 2 width at posterior margin/length: 4.4–4.7. Mediotergite 2 sculpture: mostly smooth. Outer margin of hypopygium: with a wide, medially folded, transparent, semi–desclerotized area; usually with 4 or more pleats. Ovipositor thickness: anterior width at most 2.0 × posterior width (beyond ovipositor constriction). Ovipositor sheaths length/metatibial length: 0.4–0.5. Length of fore wing veins r/2RS: 1.7–1.9. Length of fore wing veins 2RS/2M: 1.4–1.6. Length of fore wing veins 2M/(RS+M)b: 0.7–0.8. Pterostigma length/width: 2.6–3.0. Point of insertion of vein r in pterostigma: about half way point length of pterostigma. Angle of vein r with fore wing anterior margin: clearly outwards, inclined towards fore wing apex. Shape of junction of veins r and 2RS in fore wing: distinctly but not strongly angled.

**Male.** Unknown.

#### Molecular data.

Sequences in BOLD: 4, barcode compliant sequences: 2.

#### Biology/ecology.

Gregarious. Host: Riodinidae, *Calydna sturnula*.

#### Distribution.

Costa Rica, ACG.

#### Comments.

The humeral complex has a small area on outer margin that is slightly lighter in color than the rest, but we still consider it as fully brown, and thus it is coded as such.

#### Etymology.

We dedicate this species to Ronald Navarro in recognition of his diligent efforts for the ACG Programa de Sectores.

### Apanteles ronaldquirosi

Fernández-Triana sp. n.

http://zoobank.org/1937443C-793F-446D-B227-A981A08C0ADE

http://species-id.net/wiki/Apanteles_ronaldquirosi

Fig. 30

#### Type locality.

COSTA RICA, Alajuela, ACG, Sector Rincon Rain Forest, Camino Porvenir, 383m, 10.90383, -85.25964.

#### Holotype.

♀ in CNC. Specimen labels: 1. DHJPAR0043013. 2. COSTA RICA, Guanacaste, ACG, Sector Rincon Rain Forest, Camino Porvenir, 26.i.2011, 10.90383°N, -85.25964°W, 383m, DHJPAR0043013. 3. Voucher: D.H.Janzen & W.Hallwachs, DB: http://janzen.sas.upenn.edu, Area de Conservación Guanacaste, COSTA RICA, 11-SRNP-40491.

#### Paratypes.

1 ♀, 1 ♂ (CNC). COSTA RICA, ACG database codes: DHJPAR0042977, DHJPAR0045273.

#### Description.

**Female.** Body color: body mostly dark except for some sternites which may be pale. Antenna color: scape, pedicel, and flagellum dark. Coxae color (pro-, meso-, metacoxa): dark, dark, dark. Femora color (pro-, meso-, metafemur): anteriorly dark/posteriorly pale, dark, dark. Tibiae color (pro-, meso-, metatibia): pale, dark, dark. Tegula and humeral complex color: tegula dark, humeral complex half pale/half dark. Pterostigma color: mostly pale and/or transparent, with thin dark borders. Fore wing veins color: mostly dark (a few veins may be unpigmented). Antenna length/body length: antenna shorter than body (head to apex of metasoma), not extending beyond anterior 0.7 metasoma length. Body in lateral view: not distinctly flattened dorso–ventrally. Body length (head to apex of metasoma): 2.7–2.8 mm. Fore wing length: 2.7–2.8 mm. Ocular–ocellar line/posterior ocellus diameter: 2.3–2.5. Interocellar distance/posterior ocellus diameter: 2.0–2.2. Antennal flagellomerus 2 length/width: 1.7–1.9. Antennal flagellomerus 14 length/width: 1.4–1.6. Length of flagellomerus 2/length of flagellomerus 14: 1.7–1.9. Tarsal claws: with single basal spine–like seta. Metafemur length/width: 3.0–3.1. Anteromesoscutum: mostly with deep, dense punctures (separated by less than 2.0 × its maximum diameter). Mesoscutellar disc: mostly smooth. Number of pits in scutoscutellar sulcus: 7 or 8 or 9 or 10. Maximum height of mesoscutellum lunules/maximum height of lateral face of mesoscutellum: 0.6–0.7. Propodeum areola: completely defined by carinae, including transverse carina extending to spiracle. Propodeum background sculpture: partly sculptured, especially on anterior 0.5. Mediotergite 1 length/width at posterior margin: 2.0–2.2. Mediotergite 1 shape: more or less parallel–sided. Mediotergite 1 sculpture: mostly sculptured, excavated area centrally with transverse striation inside and/or a polished knob centrally on posterior margin of mediotergite. Mediotergite 2 width at posterior margin/length: 3.6–3.9, rarely 3.2–3.5. Mediotergite 2 sculpture: mostly smooth. Outer margin of hypopygium: with a wide, medially folded, transparent, semi–desclerotized area; usually with 4 or more pleats. Ovipositor thickness: anterior width at most 2.0 × posterior width (beyond ovipositor constriction). Ovipositor sheaths length/metatibial length: 0.8–0.9. Length of fore wing veins r/2RS: 2.3 or more. Length of fore wing veins 2RS/2M: 0.9–1.0. Length of fore wing veins 2M/(RS+M)b: 0.7–0.8. Pterostigma length/width: 3.1–3.5. Point of insertion of vein r in pterostigma: about half way point length of pterostigma. Angle of vein r with fore wing anterior margin: clearly outwards, inclined towards fore wing apex. Shape of junction of veins r and 2RS in fore wing: distinctly but not strongly angled.

**Male.** As in female.

#### Molecular data.

Sequences in BOLD: 9, barcode compliant sequences: 9.

#### Biology/ecology.

Solitary. Hosts: Crambidae, *Pilocrocis xanthozonalis*, *Palpusia plumipes*, *Leucochromodes melusinalis* DHJ01.

#### Distribution.

Costa Rica, ACG.

#### Etymology.

We dedicate this species to Ronald Quirós in recognition of his diligent efforts for the ACG Sector Marino.

### Apanteles ronaldzunigai

Fernández-Triana sp. n.

http://zoobank.org/BF4060A5-23B2-4A62-B358-B76B6CBF5ED5

http://species-id.net/wiki/Apanteles_ronaldzunigai

Fig. 200

Apanteles Rodriguez24 (Smith et al. 2006). Interim name provided by the authors.

#### Type locality.

COSTA RICA, Guanacaste, ACG, Sector Santa Rosa, Cafetal, 280m, 10.85827, -85.61089.

#### Holotype.

♀ in CNC. Specimen labels: 1. DHJPAR0005285. 2. COSTA RICA: Guanacaste: Area de Conservacion Guanacaste: Santa Rosa: Cafetal, 07/23/1992: gusaneros. 3. 92-SRNP-3778, Urbanus dorantes, Desmodium distortum.

#### Paratypes.

7 ♀, 8 ♂ (BMNH, CNC, INBIO, INHS, NMNH). COSTA RICA: ACG database code: 92-SRNP-3788, 92-SRNP-4386, 93-SRNP-6245.

#### Description.

**Female.** Metatibia color (outer face): with extended pale coloration (light yellow to orange–yellow), ranging from 0.4 to almost entire metatibia length. Fore wing veins color: veins C+Sc+R and R1 with brown coloration restricted narrowly to borders, interior area of those veins and pterostigma (and sometimes veins r, 2RS and 2M) transparent or white; other veins mostly transparent. Antenna length/body length: antenna about as long as body (head to apex of metasoma); if slightly shorter, at least extending beyond anterior 0.7 metasoma length. Body length (head to apex of metasoma): 2.1–2.2 mm or 2.3–2.4 mm. Fore wing length: 2.3–2.4 mm or 2.5–2.6 mm. Metafemur length/width: 2.8–2.9, 3.0–3.1 or 3.2–3.3. Mediotergite 1 length/width at posterior margin: 2.3–2.4. Mediotergite 1 maximum width/width at posterior margin: 1.4–1.5. Ovipositor sheaths length/metafemur length: 0.9 or 1.0. Ovipositor sheaths length/metatibia length: 0.7 or 0.8.

#### Molecular data.

Sequences in BOLD: 9 < in my NJ tree from bold, there are only three R24 specimens called that, and the others are parked in R23 barcode compliant sequences: 2.

#### Biology/ecology.

Gregarious. Hosts: Hesperiidae, *Astraptes alardus*, *Urbanus dorantes*.

#### Distribution.

Costa Rica, ACG.

#### Etymology.

We dedicate this species to Ronald Zúñiga in recognition of his diligent efforts for the ACG Programa de Parataxónomos, and Hymenoptera curatorial taxonomy for INBio, Costa Rica’s Instituto Nacional de Biodiversidad, and for ACG.

### Apanteles rosibelelizondoae

Fernández-Triana sp. n.

http://zoobank.org/D978C39A-73EF-4F83-8B49-0A18770373E7

http://species-id.net/wiki/Apanteles_rosibelelizondoae

Figs 159 , 302

Apanteles Rodriguez159 (Smith et al. 2006). Interim name provided by the authors.

#### Type locality.

COSTA RICA, Alajuela, ACG, Sector San Cristobal, Bosque Trampa Malaise, 815m, 10.86280, -85.38460.

#### Holotype.

♀ in CNC. Specimen labels: 1. San Gerardo: MT, San Cristobal, 18-24 Jan. 2008. 2. DHJPAR0027647.

#### Description.

**Female.** Body color: head dark, mesosoma dark with parts of axillar complex pale, metasoma with some mediotergites, most laterotergites, sternites, and/or hypopygium pale. Antenna color: scape, pedicel, and flagellum dark. Coxae color (pro-, meso-, metacoxa): pale, pale, pale. Femora color (pro-, meso-, metafemur): pale, pale, anteriorly pale/posteriorly dark. Tibiae color (pro-, meso-, metatibia): pale, pale, mostly pale but with posterior 0.2 or less dark. Tegula and humeral complex color: both pale. Pterostigma color: dark. Fore wing veins color: mostly dark (a few veins may be unpigmented). Antenna length/body length: antenna about as long as body (head to apex of metasoma); if slightly shorter, at least extending beyond anterior 0.7 metasoma length. Body in lateral view: not distinctly flattened dorso–ventrally. Body length (head to apex of metasoma): 2.7–2.8 mm. Fore wing length: 2.9–3.0 mm. Ocular–ocellar line/posterior ocellus diameter: 2.6 or more. Interocellar distance/posterior ocellus diameter: 1.4–1.6. Antennal flagellomerus 2 length/width: 2.9–3.1. Antennal flagellomerus 14 length/width: 1.1–1.3. Length of flagellomerus 2/length of flagellomerus 14: 2.0–2.2. Tarsal claws: simple. Metafemur length/width: 3.4–3.5. Metatibia inner spur length/metabasitarsus length: 0.4–0.5. Anteromesoscutum: mostly smooth or with shallow sparse punctures, except for anterior 0.3 where it has deeper and/or denser punctures. Mesoscutellar disc: mostly smooth. Number of pits in scutoscutellar sulcus: 3 or 4. Maximum height of mesoscutellum lunules/maximum height of lateral face of mesoscutellum: 0.4–0.5. Propodeum areola: completely defined by carinae, including transverse carina extending to spiracle. Propodeum background sculpture: mostly sculptured. Mediotergite 1 length/width at posterior margin: 2.9–3.1. Mediotergite 1 shape: clearly narrowing towards posterior margin. Mediotergite 1 sculpture: mostly sculptured, excavated area centrally with transverse striation inside and/or a polished knob centrally on posterior margin of mediotergite. Mediotergite 2 width at posterior margin/length: 2.8–3.1. Mediotergite 2 sculpture: more or less fully sculptured, with longitudinal striation. Outer margin of hypopygium: with a wide, medially folded, transparent, semi–desclerotized area; usually with 4 or more pleats. Ovipositor thickness: anterior width at most 2.0 × posterior width (beyond ovipositor constriction). Ovipositor sheaths length/metatibial length: 0.8–0.9. Length of fore wing veins r/2RS: 1.1–1.3. Length of fore wing veins 2RS/2M: 1.1–1.3. Length of fore wing veins 2M/(RS+M)b: 0.7–0.8. Pterostigma length/width: 3.1–3.5. Point of insertion of vein r in pterostigma: about half way point length of pterostigma. Angle of vein r with fore wing anterior margin: clearly outwards, inclined towards fore wing apex. Shape of junction of veins r and 2RS in fore wing: strongly angulated, sometimes with a knob.

**Male.** Unknown.

#### Molecular data.

Sequences in BOLD: 26, barcode compliant sequences: 25.

#### Biology/ecology.

Solitary (Fig. 302). Hosts: Crambidae, 11 species including *Microthyris prolongalis*, *Salbia haemorrhoidalis*, *Salbia cassidalis*, *Herpetogramma salbialis* Eulepte Janzen03, *Neurophyseta completalis*, and *Ategumia* lotanalisDHJ07; Tortricidae, one possible species, *Paramorbia* Brown001DHJ01.

#### Distribution.

Costa Rica, ACG.

#### Comments.

This species is characterized by mediotergite 2 fully sculptured with longitudinal striation, and extensive yellow-orange coloration (including tegula and humeral complex, parts of the axillar complex, most of laterotergites 1-4, all sternites, and hypopygium).

#### Etymology.

We dedicate this species to Rosibel Elizondo in recognition of her diligent efforts for the ACG Programa de Educacion Biológica.

### Apanteles rostermoragai

Fernández-Triana sp. n.

http://zoobank.org/66DF3C7E-89F4-4EE4-9879-F309A28DA470

http://species-id.net/wiki/Apanteles_rostermoragai

Figs 169 , 328

Apanteles Rodriguez96 (Smith et al. 2006). Interim name provided by the authors.

#### Type locality.

COSTA RICA, Guanacaste, ACG, Sector Pitilla, Pasmompa, 440m, 11.01926, -85.40997.

#### Holotype.

♀ in CNC. Specimen labels: 1. DHJPAR0004845. 2. COSTA RICA, Guanacaste, ACG, Sector Pitilla, Pasmompa, 21.xi.2005, 440m, 11.01926, -85.40997, 05-SRNP-34862.

#### Paratypes.

36 ♀, 3 ♂ (BMNH, CNC, INBIO, INHS, NMNH). COSTA RICA, ACG database codes: DHJPAR0030788, DHJPAR0025861, DHJPAR0030918.

#### Description.

**Female.** Metatibia color (outer face): with extended pale coloration (light yellow to orange–yellow), ranging from 0.4 to almost entire metatibia length. Fore wing veins color: veins C+Sc+R and R1 with brown coloration restricted narrowly to borders, interior area of those veins and pterostigma (and sometimes veins r, 2RS and 2M) transparent or white; other veins mostly transparent. Antenna length/body length: antenna about as long as body (head to apex of metasoma); if slightly shorter, at least extending beyond anterior 0.7 metasoma length. Body length (head to apex of metasoma): 2.1–2.2 mm, rarely 2.0 mm or less or 2.5–2.6 mm. Fore wing length: 2.1–2.2 mm, rarely 2.5–2.6 mm. Metafemur length/width: 3.2–3.3. Mediotergite 1 length/width at posterior margin: 1.9–2.0. Mediotergite 1 maximum width/width at posterior margin: 1.4–1.5. Ovipositor sheaths length/metafemur length: 0.9. Ovipositor sheaths length/metatibia length: 0.8.

#### Molecular data.

Sequences in BOLD: 8, barcode compliant sequences: 8.

#### Biology/ecology.

Gregarious (Fig. 328). Host: Hesperiidae, *Urbanus doryssus* DHJ02.

#### Distribution.

Costa Rica, ACG.

#### Etymology.

We dedicate this species to Roster Moraga in recognition of his diligent efforts for the ACG Programa de Parataxónomos and Estación Biológica Los Almendros in Sector Del Oro and Sector El Hacha of ACG.

### Apanteles ruthfrancoae

Fernández-Triana sp. n.

http://zoobank.org/41EA537F-9760-4098-AF45-001BA4F491A2

http://species-id.net/wiki/Apanteles_ruthfrancoae

Figs 64 , 256

Apanteles Rodriguez07 (Smith et al. 2006), in part. Interim name provided by the authors.

#### Type locality.

COSTA RICA, Guanacaste, ACG, Sector Cacao, Estación Gongora, 570m, 10.88700, -85.47443.

#### Holotype.

♀ in CNC. Specimen labels: 1. COSTA RICA, Guanacaste, ACG, Cacao, Estación Gongora, 07/27/1995: gusaneros. 2. 95-SRNP-7168, *Staphylus* prob. *vulgata*, Amaranthaceae 13207. 3. DHJPAR0005254.

#### Paratypes.

36 ♀, 30 ♂ (BMNH, CNC, INBIO, INHS, NMNH). COSTA RICA: ACG database codes: 95-SRNP-7163, 95-SRNP-7168, 95-SRNP-7169, 95-SRNP-7171, 96-SRNP-10698, 97-SRNP-3323, 97-SRNP-4093, 02-SRNP-29391, 02-SRNP-29405, 02-SRNP-32482, 04-SRNP-45194, 04-SRNP-45195, 04-SRNP-45196, 04-SRNP-45421, 04-SRNP-45422, 04-SRNP-45424, 04-SRNP-45425, 04-SRNP-45426, 04-SRNP-45428, 04-SRNP-45476, 04-SRNP-45478, 04-SRNP-45511, 04-SRNP-45538, 04-SRNP-45544, 04-SRNP-45545, 04-SRNP-45555, 04-SRNP-45601.

#### Description.

**Female.** Body color: body mostly dark except for some sternites which may be pale. Antenna color: scape, pedicel, and flagellum dark. Coxae color (pro-, meso-, metacoxa): pale, dark, dark. Femora color (pro-, meso-, metafemur): pale, pale, pale, rarely pale, pale, mostly pale but posterior 0.2 or less dark. Tibiae color (pro-, meso-, metatibia): pale, pale, pale, rarely pale, pale, mostly pale but with posterior 0.2 or less dark. Tegula and humeral complex color: both pale. Pterostigma color: dark with pale spot at base. Fore wing veins color: mostly dark (a few veins may be unpigmented). Antenna length/body length: antenna shorter than body (head to apex of metasoma), not extending beyond anterior 0.7 metasoma length. Body in lateral view: not distinctly flattened dorso–ventrally. Body length (head to apex of metasoma): 2.7–2.8 mm, 2.9–3.0 mm, rarely 2.5–2.6 mm. Fore wing length: 2.7–2.8 mm or 2.9–3.0 mm. Ocular–ocellar line/posterior ocellus diameter: 2.3–2.5. Interocellar distance/posterior ocellus diameter: 1.7–1.9. Antennal flagellomerus 2 length/width: 2.3–2.5. Antennal flagellomerus 14 length/width: 1.1–1.3. Length of flagellomerus 2/length of flagellomerus 14: 2.0–2.2. Tarsal claws: with single basal spine–like seta. Metafemur length/width: 2.6–2.7. Metatibia inner spur length/metabasitarsus length: 0.6–0.7. Anteromesoscutum: mostly with deep, dense punctures (separated by less than 2.0 × its maximum diameter). Mesoscutellar disc: mostly smooth. Number of pits in scutoscutellar sulcus: 9 or 10. Maximum height of mesoscutellum lunules/maximum height of lateral face of mesoscutellum: 0.6–0.7. Propodeum areola: completely defined by carinae, including transverse carina extending to spiracle. Propodeum background sculpture: mostly sculptured. Mediotergite 1 length/width at posterior margin: 3.8–4.0. Mediotergite 1 shape: mostly parallel–sided for 0.5–0.7 of its length, then narrowing posteriorly so mediotergite anterior width >1.1 × posterior width. Mediotergite 1 sculpture: mostly sculptured, excavated area centrally with transverse striation inside and/or a polished knob centrally on posterior margin of mediotergite. Mediotergite 2 width at posterior margin/length: 2.4–2.7. Mediotergite 2 sculpture: mostly smooth or with some sculpture, mostly near posterior margin. Outer margin of hypopygium: with a medially folded, transparent, semi–desclerotized area; with 0–3 pleats visible. Ovipositor thickness: anterior width 3.0–5.0 × posterior width (beyond ovipositor constriction). Ovipositor sheaths length/metatibial length: 0.8–0.9 or 1.0–1.1. Length of fore wing veins r/2RS: 1.4–1.6. Length of fore wing veins 2RS/2M: 1.4–1.6. Length of fore wing veins 2M/(RS+M)b: 0.5–0.6. Pterostigma length/width: 2.6–3.0. Point of insertion of vein r in pterostigma: clearly beyond half way point length of pterostigma. Angle of vein r with fore wing anterior margin: clearly outwards, inclined towards fore wing apex. Shape of junction of veins r and 2RS in fore wing: evenly curved.

**Male.** As in female, but darker coloured, especially on legs, and longer, narrower mediotergite 1.

#### Molecular data.

Sequences in BOLD: 34, barcode compliant sequences: 27, haplotypes: 2.

#### Biology/Ecology.

Gregarious (Fig. 256). Host: Hesperiidae: *Staphylus* Janzen03, *Staphylus caribbea*, *Staphylus vulgata*.

#### Distribution.

Costa Rica, ACG.

#### Comments.

This new name may actually represent two different species, one reared from *Staphylus* Janzen03 and *Staphylus vulgata*, and the other reared from *Staphylus caribbea*. DNA barcoding haplotype data suggests this hypothesis, just as barcode data suggested the separation of *Apanteles duniagarciae* from *Apanteles ruthfrancoae*, but we have not yet been able to study the specimens reared from *Staphylus caribbea* and thus cannot describe them here.

#### Etymology.

We dedicate this species to Ruth Franco in recognition of her diligent efforts for the ACG Parataxonomist Program and BioLep, the inventory of the adult ACG Lepidoptera.

### Apanteles samarshalli

Fernández-Triana, 2010: 18.

http://species-id.net/wiki/Apanteles_samarshalli

Fig. 205

Apanteles samarshalli Fernández-Triana (2010)

#### Type locality.

UNITED STATES, Florida, Monroe County, Key Largo, 25°5'11.4"N, 80°26'50.28"W.

#### Holotype.

♀ in CNC (examined).

#### Material Examined.

COSTA RICA: Guanacaste, ACG database code: DHJPAR0012514, DHJPAR0012520, DHJPAR0012616, DHJPAR0013026. CANADA, UNITED STATES: All specimens listed in Fernández-Triana (2010), plus additional specimens from the type locality (CNC).

#### Description.

**Female.** Body color: body mostly dark except for some sternites which may be pale. Antenna color: scape and/or pedicel pale, flagellum dark. Coxae color (pro-, meso-, metacoxa): pale, dark, dark. Femora color (pro-, meso-, metafemur): pale, pale, anteriorly pale/posteriorly dark. Tibiae color (pro-, meso-, metatibia): pale, pale, anteriorly pale/posteriorly dark. Tegula and humeral complex color: both pale. Pterostigma color: dark with pale spot at base. Fore wing veins color: mostly dark (a few veins may be unpigmented). Antenna length/body length: antenna very short, barely or not extending beyond mesosoma length. Body in lateral view: not distinctly flattened dorso–ventrally. Body length (head to apex of metasoma): 2.5–2.6 mm, 2.7–2.8 mm or 2.9–3.0 mm. Fore wing length: 2.3–2.4 mm or 2.5–2.6 mm. Ocular–ocellar line/posterior ocellus diameter: 1.7–1.9. Interocellar distance/posterior ocellus diameter: 1.7–1.9. Antennal flagellomerus 2 length/width: 1.4–1.6. Antennal flagellomerus 14 length/width: 1.0 or less. Length of flagellomerus 2/length of flagellomerus 14: 1.7–1.9. Tarsal claws: simple. Metafemur length/width: 2.6–2.7. Metatibia inner spur length/metabasitarsus length: 0.4–0.5. Anteromesoscutum: mostly with deep, dense punctures (separated by less than 2.0 × its maximum diameter). Mesoscutellar disc: with punctures near margins, central part mostly smooth. Number of pits in scutoscutellar sulcus: 11 or 12 or 13 or 14. Maximum height of mesoscutellum lunules/maximum height of lateral face of mesoscutellum: 0.4–0.5. Propodeum areola: weakly defined by central impression and few rugae or minute carinae arising from nucha. Propodeum background sculpture: mostly sculptured. Mediotergite 1 length/width at posterior margin: 1.4–1.6. Mediotergite 1 shape: clearly narrowing towards posterior margin. Mediotergite 1 sculpture: mostly sculptured, excavated area centrally with transverse striation inside and/or a polished knob centrally on posterior margin of mediotergite. Mediotergite 2 width at posterior margin/length: 2.8–3.1. Mediotergite 2 sculpture: mostly smooth. Outer margin of hypopygium: with a wide, medially folded, transparent, semi–desclerotized area; usually with 4 or more pleats. Ovipositor thickness: about same width throughout its length. Ovipositor sheaths length/metatibial length: 0.6–0.7. Length of fore wing veins r/2RS: 2.3 or more. Length of fore wing veins 2RS/2M: 0.8 or less. Length of fore wing veins 2M/(RS+M)b: 0.5–0.6. Pterostigma length/width: 2.6–3.0. Point of insertion of vein r in pterostigma: clearly beyond half way point length of pterostigma. Angle of vein r with fore wing anterior margin: more or less perpendicular to fore wing margin. Shape of junction of veins r and 2RS in fore wing: evenly curved.

**Male.** Unknown.

#### Molecular data.

Sequences in BOLD: 8 (4 from ACG), barcode compliant sequences: 4 (3 from ACG).

#### Biology/ecology.

Unknown, Malaise-trapped.

#### Distribution.

Canada (ON), Costa Rica (ACG), Mexico (Chamelas), United States (FL).

#### Comments.

Because of the unusual venation in the forewing and the lack of a propodeal areola, the generic position of this species has been in doubt since its description (Fernández-Triana 2010).

### Apanteles sergiocascantei

Fernández-Triana sp. n.

http://zoobank.org/BB2ABCA8-644B-4BC4-A994-B566B45A7F1E

http://species-id.net/wiki/Apanteles_sergiocascantei

Figs 161 , 303

Apanteles Rodriguez113. Smith et al. (2008). Interim name provided by the authors.

#### Type locality.

COSTA RICA, Guanacaste, ACG, Sector Pitilla, Pasmompa, 440m, 11.01926, -85.40997.

#### Holotype.

♀ in CNC. Specimen labels: 1. DHJPAR0004868. 2. Costa Rica: Guanacaste, ACG, Sector Pitilla, Pasmompa, 26.x.2006, 440m, 11.01926, -85.40997.

#### Paratypes.

2 ♀, 2 ♂ (BMNH, CNC, INBIO, INHS, NMNH). COSTA RICA, ACG database codes: DHJPAR0004873, DHJPAR0004875, DHJPAR0038314, DHJPAR0039033.

#### Description.

**Female.** Body color: body mostly dark except for some sternites which may be pale. Antenna color: scape, pedicel, and flagellum dark. Coxae color (pro-, meso-, metacoxa): pale, pale, pale. Femora color (pro-, meso-, metafemur): pale, pale, anteriorly pale/posteriorly dark. Tibiae color (pro-, meso-, metatibia): pale, pale, mostly pale but with posterior 0.2 or less dark. Tegula and humeral complex color: both dark. Pterostigma color: dark. Fore wing veins color: mostly dark (a few veins may be unpigmented). Antenna length/body length: antenna about as long as body (head to apex of metasoma); if slightly shorter, at least extending beyond anterior 0.7 metasoma length. Body in lateral view: not distinctly flattened dorso–ventrally. Body length (head to apex of metasoma): 2.3–2.4 mm, 2.5–2.6 mm or 2.7–2.8 mm. Fore wing length: 2.5–2.6 mm or 2.7–2.8 mm. Ocular–ocellar line/posterior ocellus diameter: 2.3–2.5. Interocellar distance/posterior ocellus diameter: 2.0–2.2. Antennal flagellomerus 2 length/width: 2.6–2.8. Antennal flagellomerus 14 length/width: 1.4–1.6. Length of flagellomerus 2/length of flagellomerus 14: 1.7–1.9. Tarsal claws: with single basal spine–like seta. Metafemur length/width: 3.2–3.3. Metatibia inner spur length/metabasitarsus length: 0.4–0.5. Anteromesoscutum: mostly with shallow, sparse punctures (separated by more than 2.0 × its maximum diameter). Mesoscutellar disc: mostly punctured. Number of pits in scutoscutellar sulcus: 5 or 6. Maximum height of mesoscutellum lunules/maximum height of lateral face of mesoscutellum: 0.4–0.5. Propodeum areola: completely defined by carinae, including transverse carina extending to spiracle. Propodeum background sculpture: mostly sculptured. Mediotergite 1 length/width at posterior margin: 3.5–3.7 or 3.8–4.0. Mediotergite 1 shape: mostly parallel–sided for 0.5–0.7 of its length, then narrowing posteriorly so mediotergite anterior width >1.1 × posterior width. Mediotergite 1 sculpture: with some sculpture near lateral margins and/or posterior 0.2–0.4 of mediotergite. Mediotergite 2 width at posterior margin/length: 3.6–3.9. Mediotergite 2 sculpture: mostly smooth. Outer margin of hypopygium: with a wide, medially folded, transparent, semi–desclerotized area; usually with 4 or more pleats. Ovipositor thickness: about same width throughout its length. Ovipositor sheaths length/metatibial length: 0.4–0.5. Length of fore wing veins r/2RS: 1.4–1.6. Length of fore wing veins 2RS/2M: 1.4–1.6. Length of fore wing veins 2M/(RS+M)b: 0.9–1.0. Pterostigma length/width: 2.6–3.0. Point of insertion of vein r in pterostigma: about half way point length of pterostigma. Angle of vein r with fore wing anterior margin: clearly outwards, inclined towards fore wing apex. Shape of junction of veins r and 2RS in fore wing: distinctly but not strongly angled.

**Male.** As in female, with metacoxa slightly darker dorsally.

#### Molecular data.

Sequences in BOLD: 9, barcode compliant sequences: 9.

#### Biology/ecology.

Solitary (Fig. 303). Host: Choreutidae, *Brenthia* Janzen04.

#### Distribution.

Costa Rica, ACG.

#### Comments.

The vannal lobe of the fore wing is more or less straight (*Apanteles*-like) but it is also fully setose – suggesting this species might better be placed within the genus *Dolichogenidea*. However, molecular data clusters the species with other *Apanteles*. The limits of *Apanteles* and *Dolichogenidea* have long been controversial (e.g., Mason 1981; Achterberg 2003; Fernández-Triana 2010) and solving that is beyond the scope of this paper. The species is characterized by all coxae yellow (but not extensive yellow coloration in rest of the body), very short ovipositor (0.4 × as long as metatibia length), and mediotergite 1 3.8–4.0 × as long as wide at posterior margin.

#### Etymology.

We dedicate this species to Sergio Cascante in recognition of his diligent efforts for the ACG Programa de Seguridad.

### Apanteles sergioriosi

Fernández-Triana sp. n.

http://zoobank.org/67AEFC42-7415-47FB-88DE-67B62BDE91CF

http://species-id.net/wiki/Apanteles_sergioriosi

Figs 201 , 325

Apanteles Rodriguez166 (Smith et al. 2006). Interim name provided by the authors.

#### Type locality.

COSTA RICA, Guanacaste, ACG, Sector Del Oro, Uncaria, 370m, 11.01752, -85.47411.

#### Holotype.

♀ in CNC. Specimen labels: 1. DHJPAR0012293. 2. COSTA RICA, Guanacaste, ACG, Sector Del Oro, Uncaria, 26.xii.2006, 370m, 11.01752, -85.47411, 06-SRNP-22608.

#### Paratypes.

4 ♀, 2 ♂ (CNC, NMNH). COSTA RICA: ACG database codes: 93-SRNP-5683, 93-SRNP-6294, 04-SRNP-45450.

#### Description.

**Female.** Metatibia color (outer face): with extended pale coloration (light yellow to orange–yellow), ranging from 0.4 to almost entire metatibia length. Fore wing veins color: veins C+Sc+R and R1 mostly brown; usually veins r, 2RS, 2M, (RS+M)b, 1CU, 2Cua, and 1m–cu partially brown; interior area of other veins, and at least part of pterostigma, usually light brown or yellowish–white. Antenna length/body length: antenna about as long as body (head to apex of metasoma); if slightly shorter, at least extending beyond anterior 0.7 metasoma length. Body length (head to apex of metasoma): 2.3–2.4 mm. Fore wing length: 2.5–2.6 mm. Metafemur length/width: 2.8–2.9 or 3.0–3.1. Mediotergite 1 length/width at posterior margin: 2.3–2.4. Mediotergite 1 maximum width/width at posterior margin: 1.4–1.5. Ovipositor sheaths length/metafemur length: 0.8 or 0.9. Ovipositor sheaths length/metatibia length: 0.7 or 0.8.

#### Molecular data.

Sequences in BOLD: 4, barcode compliant sequences: 4.

#### Biology/ecology.

Gregarious (Fig. 325). Hosts: Hesperiidae, *Urbanus simplicius*.

#### Distribution.

Costa Rica, ACG.

#### Etymology.

We dedicate this species to Sergio Ríos in recognition of his diligent efforts for the ACG Parataxonomist Program and BioLep, the inventory of the adult ACG Lepidoptera.

### Apanteles sigifredomarini

Fernández-Triana sp. n.

http://zoobank.org/C6E52C4E-5B63-40E4-87FA-C26B7E383600

http://species-id.net/wiki/Apanteles_sigifredomarini

Fig. 192

Apanteles Rodriguez39 (Smith et al. 2006). Interim name provided by the authors.

#### Type locality.

COSTA RICA, Guanacaste, ACG, Sector Cacao, Gongora Bananal, 600m, 10.88919, -85.47609.

#### Holotype.

♀ in CNC. Specimen labels: 1. COSTA RICA, Guanacaste, ACG, Sector Cacao, Gongora Bananal, 13.iii.1997, 600m, 10.88919, -85.47609, DHJPAR0001546.

#### Paratypes.

17 ♀, 6 ♂ (BMNH, CNC, INBIO, INHS, NMNH). COSTA RICA: ACG database codes: DHJPAR0005225.

#### Description.

**Female.** Metatibia color (outer face): with extended pale coloration (light yellow to orange–yellow), ranging from 0.4 to almost entire metatibia length. Fore wing veins color: veins C+Sc+R and R1 with brown coloration restricted narrowly to borders, interior area of those veins and pterostigma (and sometimes veins r, 2RS and 2M) transparent or white; other veins mostly transparent. Antenna length/body length: antenna about as long as body (head to apex of metasoma); if slightly shorter, at least extending beyond anterior 0.7 metasoma length. Body length (head to apex of metasoma): 2.1–2.2 mm or 2.3–2.4 mm. Fore wing length: 2.3–2.4 mm or 2.5–2.6 mm. Metafemur length/width: 2.8–2.9 or 3.0–3.1. Mediotergite 1 length/width at posterior margin: 2.3–2.4. Mediotergite 1 maximum width/width at posterior margin: 1.4–1.5. Ovipositor sheaths length/metafemur length: 1.0. Ovipositor sheaths length/metatibia length: 0.8.

#### Molecular data.

Sequences in BOLD: 2, barcode compliant sequences: 2.

#### Biology/ecology.

Gregarious. Host: Hesperiidae, *Astraptes anaphus annetta*.

#### Distribution.

Costa Rica, ACG.

#### Etymology.

We dedicate this species to Sigifredo Marin in recognition of his unwavering support and defense of the development of the Programa de Parataxónomos, and his key role as former director of ACG and the primary land negotiator for the Guanacaste Dry Forest Conservation Fund (http://www.gdfcf.org).

### Apanteles thurberiae

Muesebeck, 1921

http://species-id.net/wiki/Apanteles_thurberiae

Fig. 153

Apanteles thurberiae Muesebeck, 1921: 507.

#### Type locality.

UNITED STATES: Arizona, Santa Rita Mountains, Stone Cabin Canyon.

#### Holotype.

♀, NMNH (examined).

#### Material Examined.

1 ♀, 1 ♂, Paratypes (CNC). UNITED STATES: CA, Sabino Canyon, xi-1913, A.W. Morrill, ex bollworm on *Thurberia thespesioides*.

#### Description.

**Female.** Body color: body mostly dark except for some sternites which may be pale. Antenna color: scape, pedicel, and flagellum dark. Coxae color (pro-, meso-, metacoxa): pale, dark, dark. Femora color (pro-, meso-, metafemur): pale, anteriorly dark/posteriorly pale, dark. Tibiae color (pro-, meso-, metatibia): pale, pale, mostly pale but with posterior 0.2 or less dark. Tegula and humeral complex color: tegula pale, humeral complex half pale/half dark. Pterostigma color: mostly pale and/or transparent, with thin dark borders. Fore wing veins color: mostly white or entirely transparent. Antenna length/body length: antenna about as long as body (head to apex of metasoma); if slightly shorter, at least extending beyond anterior 0.7 metasoma length. Body in lateral view: not distinctly flattened dorso–ventrally. Body length (head to apex of metasoma): 3.5–3.6 mm. Fore wing length: 3.7–3.8 mm or 3.9–4.0 mm. Ocular–ocellar line/posterior ocellus diameter: 2.0–2.2. Interocellar distance/posterior ocellus diameter: 1.7–1.9. Antennal flagellomerus 2 length/width: 2.6–2.8. Antennal flagellomerus 14 length/width: 1.7–1.9. Length of flagellomerus 2/length of flagellomerus 14: 2.0–2.2. Tarsal claws: simple. Metafemur length/width: 2.8–2.9. Metatibia inner spur length/metabasitarsus length: 0.4–0.5. Anteromesoscutum: mostly with deep, dense punctures (separated by less than 2.0 × its maximum diameter). Mesoscutellar disc: mostly smooth. Number of pits in scutoscutellar sulcus: 9 or 10 or 11 or 12. Maximum height of mesoscutellum lunules/maximum height of lateral face of mesoscutellum: 0.6–0.7. Propodeum areola: completely defined by carinae, including transverse carina extending to spiracle. Propodeum background sculpture: mostly sculptured. Mediotergite 1 length/width at posterior margin: 1.1–1.3. Mediotergite 1 shape: clearly widening towards posterior margin. Mediotergite 1 sculpture: mostly sculptured, excavated area centrally with transverse striation inside and/or a polished knob centrally on posterior margin of mediotergite. Mediotergite 2 width at posterior margin/length: 3.2–3.5. Mediotergite 2 sculpture: with some sculpture, mostly near posterior margin. Outer margin of hypopygium: with a wide, medially folded, transparent, semi–desclerotized area; usually with 4 or more pleats. Ovipositor thickness: about same width throughout its length. Ovipositor sheaths length/metatibial length: 1.4–1.5. Length of fore wing veins r/2RS: 2.0–2.2. Length of fore wing veins 2RS/2M: 1.4–1.6. Length of fore wing veins 2M/(RS+M)b: 0.5–0.6. Pterostigma length/width: 3.1–3.5. Point of insertion of vein r in pterostigma: clearly beyond half way point length of pterostigma. Angle of vein r with fore wing anterior margin: clearly outwards, inclined towards fore wing apex. Shape of junction of veins r and 2RS in fore wing: distinctly but not strongly angled.

**Male.** Slight differences in the shape and sculpture of mediotergite 2.

#### Molecular data.

No molecular data available for this species.

#### Biology/ecology.

Solitary; white coccons formed in the bolls (Mueseback 1921). Hosts: Gelechiidae, *Pectinophora gossypiella*, Noctuidae, *Helicoverpa zea*, *Sacadodes pyralis*, *Thurberiphaga diffusa*.

#### Distribution.

Colombia, Nicaragua, Trinidad & Tobago, United States (AZ, TX), Venezuela. There is no suggestion that this species occurs in ACG.

#### Comments.

There are very few morphological differences between this species and *Apanteles esthercentenoae*; and future studies might find the latter species to be a synonym of *Apanteles*. *thurberiae*. However, we consider the differences in host families and tarsal claws of adult wasps (likely to be related to host selection, manipulation and parasitation), as well as rather elongate glossa of *Apanteles esthercentenoae*, to support the separation of these two species.

### Apanteles tiboshartae

Fernández-Triana sp. n.

http://zoobank.org/F3C04CD2-676F-4223-8BF7-33119231AE99

http://species-id.net/wiki/Apanteles_tiboshartae

Figs 49 , 242

#### Type locality.

COSTA RICA, Guanacaste, ACG, Sector San Cristobal, Tajo Angeles, 540m, 10.86472, -85.41531.

#### Holotype.

♀ in CNC. Specimen labels: 1. Voucher: D.H.Janzen & W.Hallwachs, DB: http://janzen.sas.upenn.edu, Area de Conservación Guanacaste, COSTA RICA, 09-SRNP-5496. 2. DHJPAR0038226.

#### Paratypes.

8 ♀, 2 ♂ (CNC, NMNH, BMNH). COSTA RICA, ACG database codes: DHJPAR0025764, DHJPAR0038228, DHJPAR0038230, DHJPAR0038252, DHJPAR0038257, DHJPAR0039743, DHJPAR0038347, DHJPAR0039058, DHJPAR0039747, DHJPAR0039778.

#### Description.

**Female.** Body color: body mostly dark except for some sternites which may be pale. Antenna color: scape, pedicel, and flagellum dark. Coxae color (pro-, meso-, metacoxa): dark, dark, dark. Femora color (pro-, meso-, metafemur): pale, anteriorly dark/posteriorly pale, mostly dark but anterior 0.2 or less pale. Tibiae color (pro-, meso-, metatibia): pale, pale, anteriorly pale/posteriorly dark. Tegula and humeral complex color: both pale. Pterostigma color: dark. Fore wing veins color: mostly dark (a few veins may be unpigmented). Antenna length/body length: antenna about as long as body (head to apex of metasoma); if slightly shorter, at least extending beyond anterior 0.7 metasoma length. Body in lateral view: not distinctly flattened dorso–ventrally. Body length (head to apex of metasoma): 3.5–3.6 mm, 3.7–3.8 mm or 3.9–4.0 mm. Fore wing length: 3.3–3.4 mm, 3.5–3.6 mm or 3.7–3.8 mm. Ocular–ocellar line/posterior ocellus diameter: 2.0–2.2. Interocellar distance/posterior ocellus diameter: 1.7–1.9. Antennal flagellomerus 2 length/width: 2.6–2.8. Antennal flagellomerus 14 length/width: 1.1–1.3. Length of flagellomerus 2/length of flagellomerus 14: 2.3–2.5. Tarsal claws: with two basal spine–like setae. Metafemur length/width: 3.0–3.1. Metatibia inner spur length/metabasitarsus length: 0.6–0.7. Anteromesoscutum: mostly with deep, dense punctures (separated by less than 2.0 × its maximum diameter). Mesoscutellar disc: mostly smooth. Number of pits in scutoscutellar sulcus: 9 or 10. Maximum height of mesoscutellum lunules/maximum height of lateral face of mesoscutellum: 0.4–0.5. Propodeum areola: completely defined by carinae, including transverse carina extending to spiracle. Propodeum background sculpture: mostly sculptured. Mediotergite 1 length/width at posterior margin: 2.0–2.2. Mediotergite 1 shape: mostly parallel–sided for 0.5–0.7 of its length, then narrowing posteriorly so mediotergite anterior width >1.1 × posterior width. Mediotergite 1 sculpture: mostly sculptured, excavated area centrally with transverse striation inside and/or a polished knob centrally on posterior margin of mediotergite. Mediotergite 2 width at posterior margin/length: 1.5 or less. Mediotergite 2 sculpture: mostly smooth. Outer margin of hypopygium: with a wide, medially folded, transparent, semi–desclerotized area; usually with 4 or more pleats. Ovipositor thickness: about same width throughout its length. Ovipositor sheaths length/metatibial length: 1.6–1.7 or 1.8–1.9. Length of fore wing veins r/2RS: 1.1–1.3. Length of fore wing veins 2RS/2M: 1.4–1.6. Length of fore wing veins 2M/(RS+M)b: 0.7–0.8. Pterostigma length/width: 3.6 or more. Point of insertion of vein r in pterostigma: clearly beyond half way point length of pterostigma. Angle of vein r with fore wing anterior margin: clearly outwards, inclined towards fore wing apex. Shape of junction of veins r and 2RS in fore wing: distinctly but not strongly angled.

**Male.** Similar to female, except for mediotergite 2 much less quadrate (i.e., much more transverse).

#### Molecular data.

Sequences in BOLD: 24, barcode compliant sequences: 24.

#### Biology/ecology.

Solitary (Fig. 242). Hosts: Elachistidae, at least 12 species of *Antaeotricha*, two species of *Stenoma*.

#### Distribution.

Costa Rica, ACG.

#### Etymology.

We dedicate this species to Ti Boshart in recognition of her diligent efforts for the ACG administration and Programa de Ecoturismo.

### Apanteles vannesabrenesae

Fernández-Triana sp. n.

http://zoobank.org/CE7AAB95-EDED-4D7F-B7E5-489D170579A4

http://species-id.net/wiki/Apanteles_vannesabrenesae

Figs 34 , 233

Apanteles Rodriguez122. Smith et al. (2008). Interim name provided by the authors.

#### Type locality.

COSTA RICA, Guanacaste, ACG, Sector Cacao, Sendero Derrumbe, 1220m, 10.92918, -85.46426.

#### Holotype.

♀ in CNC. Specimen labels: 1. Costa Rica: Guanacaste, ACG, Sector Cacao, Sendero Derrumbe, 06.vi.2006, 1220m, 10.92918, -85.46426, DHJPAR0012049.

#### Paratypes.

19 ♀, 5 ♂ (BMNH, CNC, INBIO, INHS, NMNH). COSTA RICA, ACG database codes: DHJPAR0012053, DHJPAR0013104, DHJPAR0013103, DHJPAR0013108, DHJPAR0013109, DHJPAR0013110, DHJPAR0013112, DHJPAR0013117, DHJPAR0013503, DHJPAR0034267.

#### Description.

**Female.** Body color: head and mesosoma mostly dark, metasoma with some tergites and/or most of sternites pale. Antenna color: scape, pedicel, and flagellum dark. Coxae color (pro-, meso-, metacoxa): pale, pale, partially pale/partially dark. Femora color (pro-, meso-, metafemur): pale, pale, mostly pale but with dark area dorsally. Tibiae color (pro-, meso-, metatibia): pale, pale, anteriorly pale/posteriorly dark. Tegula and humeral complex color: both pale. Pterostigma color: dark with pale spot at base. Fore wing veins color: mostly dark (a few veins may be unpigmented). Antenna length/body length: antenna about as long as body (head to apex of metasoma); if slightly shorter, at least extending beyond anterior 0.7 metasoma length. Body in lateral view: not distinctly flattened dorso–ventrally. Body length (head to apex of metasoma): 2.5–2.6 mm, 2.7–2.8 mm, 2.9–3.0 mm, rarely 3.1–3.2 mm. Fore wing length: 2.7–2.8 mm, 2.9–3.0 mm, 3.1–3.2 mm, rarely 3.3–3.4 mm or 3.5–3.6 mm. Ocular–ocellar line/posterior ocellus diameter: 2.3–2.5. Interocellar distance/posterior ocellus diameter: 1.7–1.9. Antennal flagellomerus 2 length/width: 2.6–2.8. Antennal flagellomerus 14 length/width: 1.7–1.9. Length of flagellomerus 2/length of flagellomerus 14: 2.0–2.2. Tarsal claws: simple. Metafemur length/width: 2.8–2.9. Metatibia inner spur length/metabasitarsus length: 0.4–0.5. Anteromesoscutum: mostly with deep, dense punctures (separated by less than 2.0 × its maximum diameter). Mesoscutellar disc: with a few sparse punctures, with punctures near margins, central part mostly smooth, rarely mostly punctured. Number of pits in scutoscutellar sulcus: 7 or 8, rarely 5 or 6. Maximum height of mesoscutellum lunules/maximum height of lateral face of mesoscutellum: 0.4–0.5. Propodeum areola: completely defined by carinae, including transverse carina extending to spiracle. Propodeum background sculpture: mostly sculptured. Mediotergite 1 length/width at posterior margin: 2.3–2.5. Mediotergite 1 shape: mostly parallel–sided for 0.5–0.7 of its length, then narrowing posteriorly so mediotergite anterior width >1.1 × posterior width. Mediotergite 1 sculpture: with some sculpture near lateral margins and/or posterior 0.2–0.4 of mediotergite. Mediotergite 2 width at posterior margin/length: 3.6–3.9. Mediotergite 2 sculpture: mostly smooth. Outer margin of hypopygium: with a wide, medially folded, transparent, semi–desclerotized area; usually with 4 or more pleats. Ovipositor thickness: about same width throughout its length. Ovipositor sheaths length/metatibial length: 0.6–0.7, rarely 0.4–0.5. Length of fore wing veins r/2RS: 1.4–1.6. Length of fore wing veins 2RS/2M: 0.9–1.0. Length of fore wing veins 2M/(RS+M)b: 1.1–1.3. Pterostigma length/width: 2.6–3.0. Point of insertion of vein r in pterostigma: about half way point length of pterostigma. Angle of vein r with fore wing anterior margin: more or less perpendicular to fore wing margin or clearly outwards, inclined towards fore wing apex. Shape of junction of veins r and 2RS in fore wing: strongly angulated, sometimes with a knob.

**Male.** As in female, but specimens tend to be slightly darker.

#### Molecular data.

Sequences in BOLD: 15, barcode compliant sequences: 15.

#### Biology/ecology.

Gregarious (Fig. 233). Hosts: Tortricidae, *Anacrusis nephrodes*, *Anacrusis ellensatterleeae*, *Anacrusis terrimccarthyae*.

#### Distribution.

Costa Rica, ACG.

#### Etymology.

We dedicate this species to Vannesa Brenes in recognition of her diligent efforts for the ACG Horizontes Forest Experiment Station.

### Apanteles victorbarrantesi

Fernández-Triana sp. n.

http://zoobank.org/2C47F5C5-152D-47FC-9527-A3EA512DECBF

http://species-id.net/wiki/Apanteles_victorbarrantesi

Fig. 82

#### Type locality.

COSTA RICA, Guanacaste, ACG, Sector Santa Rosa, Bosque San Emilio, 300m, 10.84389, -85.61384.

#### Holotype.

♀ in CNC. Specimen labels: 1. San Emilio #2, 15.ix.1999. 2. DHJPAR0013202.

#### Paratypes.

1 ♀, 2 ♂ (CNC, NMNH). COSTA RICA, ACG database codes: DHJPAR0013552, DHJPAR0024676, DHJPAR0024683.

#### Description.

**Female.** Body color: head dark, mesosoma dark with parts of axillar complex pale, metasoma with some mediotergites, most laterotergites, sternites, and/or hypopygium pale. Antenna color: scape and/or pedicel pale, flagellum dark. Coxae color (pro-, meso-, metacoxa): pale, pale, pale. Femora color (pro-, meso-, metafemur): pale, pale, mostly pale but posterior 0.2 or less dark. Tibiae color (pro-, meso-, metatibia): pale, pale, mostly pale but with posterior 0.2 or less dark. Tegula and humeral complex color: both pale. Pterostigma color: dark. Fore wing veins color: mostly dark (a few veins may be unpigmented). Antenna length/body length: antenna about as long as body (head to apex of metasoma); if slightly shorter, at least extending beyond anterior 0.7 metasoma length. Body in lateral view: not distinctly flattened dorso–ventrally. Body length (head to apex of metasoma): 2.3–2.4 mm. Fore wing length: 2.5–2.6 mm. Ocular–ocellar line/posterior ocellus diameter: 2.0–2.2. Interocellar distance/posterior ocellus diameter: 1.7–1.9. Antennal flagellomerus 2 length/width: 2.3–2.5. Antennal flagellomerus 14 length/width: 1.4–1.6. Length of flagellomerus 2/length of flagellomerus 14: 2.0–2.2. Tarsal claws: simple. Metafemur length/width: 3.0–3.1. Metatibia inner spur length/metabasitarsus length: 0.6–0.7. Anteromesoscutum: mostly with deep, dense punctures (separated by less than 2.0 × its maximum diameter). Mesoscutellar disc: mostly punctured. Number of pits in scutoscutellar sulcus: 7 or 8. Maximum height of mesoscutellum lunules/maximum height of lateral face of mesoscutellum: 0.4–0.5. Propodeum areola: completely defined by carinae, including transverse carina extending to spiracle. Propodeum background sculpture: mostly sculptured. Mediotergite 1 length/width at posterior margin: 2.0–2.2. Mediotergite 1 shape: more or less parallel–sided. Mediotergite 1 sculpture: mostly sculptured, excavated area centrally with transverse striation inside and/or a polished knob centrally on posterior margin of mediotergite. Mediotergite 2 width at posterior margin/length: 4.0–4.3. Mediotergite 2 sculpture: mostly smooth. Outer margin of hypopygium: with a wide, medially folded, transparent, semi–desclerotized area; usually with 4 or more pleats. Ovipositor thickness: about same width throughout its length. Ovipositor sheaths length/metatibial length: 0.6–0.7. Length of fore wing veins r/2RS: 1.7–1.9. Length of fore wing veins 2RS/2M: 1.4–1.6. Length of fore wing veins 2M/(RS+M)b: 0.7–0.8. Pterostigma length/width: 3.1–3.5. Point of insertion of vein r in pterostigma: about half way point length of pterostigma. Angle of vein r with fore wing anterior margin: clearly outwards, inclined towards fore wing apex. Shape of junction of veins r and 2RS in fore wing: distinctly but not strongly angled.

**Male.** As in female except for metacoxa which is darker – the brown area covers the anterior half of metacoxa, the rest is yellow.

#### Molecular data.

Sequences in BOLD: 4, barcode compliant sequences: 4.

#### Biology/ecology.

Malaise-trapped.

#### Distribution.

Costa Rica, ACG.

#### Etymology.

We dedicate this species to Victor Barrantes in recognition of his diligent efforts for the ACG Programa de Seguridad.

### Apanteles vulgaris

(Ashmead, 1900)

http://species-id.net/wiki/Apanteles_vulgaris

Fig. 162

Urogaster vulgaris Ashmead, 1900: 286.
Apanteles vulgaris (Ashmead, 1900). Transferred by Szépligeti (1904: 111).
Urogaster xanthopus Ashmead, 1900: 288.
Apanteles xanthopus (Ashmead, 1900). Transferred by Szépligeti (1904: 111). Synonymized by Mason 1981: 52.

#### Type locality.

ST. VINCENT, Lesser Antilles.

#### Holotype.

♂, BMNH (examined).

#### Material examined.

2 ♀, 1 ♂; St. Vincent Island, 1972, Malaise Trap (CNC).

#### Description.

**Female.** Body color: head dark, mesosoma dark with parts of axillar complex pale, metasoma with some mediotergites, most laterotergites, sternites, and/or hypopygium pale or head and mesosoma mostly dark, metasoma with some tergites and/or most of sternites pale. Antenna color: scape and/or pedicel pale, flagellum dark. Coxae color (pro-, meso-, metacoxa): pale, pale, pale. Femora color (pro-, meso-, metafemur): pale, pale, pale. Tibiae color (pro-, meso-, metatibia): pale, pale, pale. Tegula and humeral complex color: both pale. Pterostigma color: dark. Fore wing veins color: mostly dark (a few veins may be unpigmented). Antenna length/body length: antenna about as long as body (head to apex of metasoma); if slightly shorter, at least extending beyond anterior 0.7 metasoma length. Body in lateral view: not distinctly flattened dorso–ventrally. Body length (head to apex of metasoma): 2.9–3.0 mm or 3.1–3.2 mm. Fore wing length: 3.1–3.2 mm. Ocular–ocellar line/posterior ocellus diameter: 1.4–1.6. Interocellar distance/posterior ocellus diameter: 1.4–1.6. Antennal flagellomerus 2 length/width: 2.9–3.1. Antennal flagellomerus 14 length/width: 1.4–1.6. Length of flagellomerus 2/length of flagellomerus 14: 2.3–2.5. Tarsal claws: simple. Metafemur length/width: 3.0–3.1. Metatibia inner spur length/metabasitarsus length: 0.6–0.7. Anteromesoscutum: mostly with deep, dense punctures (separated by less than 2.0 × its maximum diameter). Mesoscutellar disc: mostly smooth. Number of pits in scutoscutellar sulcus: 7 or 8 or 9 or 10. Maximum height of mesoscutellum lunules/maximum height of lateral face of mesoscutellum: 0.4–0.5 or 0.6–0.7. Propodeum areola: completely defined by carinae, including transverse carina extending to spiracle. Propodeum background sculpture: partly sculptured, especially on anterior 0.5. Mediotergite 1 length/width at posterior margin: 1.7–1.9. Mediotergite 1 shape: more or less parallel–sided. Mediotergite 1 sculpture: with some sculpture near lateral margins and/or posterior 0.2–0.4 of mediotergite. Mediotergite 2 width at posterior margin/length: 3.6–3.9. Mediotergite 2 sculpture: mostly smooth. Outer margin of hypopygium: with a wide, medially folded, transparent, semi–desclerotized area; usually with 4 or more pleats. Ovipositor thickness: about same width throughout its length. Ovipositor sheaths length/metatibial length: 0.6–0.7. Length of fore wing veins r/2RS: 1.4–1.6. Length of fore wing veins 2RS/2M: 2.1 or more. Length of fore wing veins 2M/(RS+M)b: 0.7–0.8. Pterostigma length/width: 3.1–3.5. Point of insertion of vein r in pterostigma: clearly beyond half way point length of pterostigma. Angle of vein r with fore wing anterior margin: clearly inwards, inclined towards fore wing base. Shape of junction of veins r and 2RS in fore wing: distinctly but not strongly angled.

**Male.** Resembles the female, but metacoxa is fully brown and metasoma is much darker, mostly brown with only laterotergites 1 and 2 yellow.

#### Molecular data.

One male, collected in 1972 from St. Vincent Island, rendered a partial barcode of 164 bp, which is unique in the BOLD database.

#### Biology/ecology.

Solitary. Hosts: Crambidae (*Diatraea lineolata* and *Diatraea saccharalis*).

#### Distribution.

Argentina, Brazil, Grenada, Puerto Rico, St. Vincent, Uruguay. There is no suggestion that this species occurs in ACG.

#### Comments.

The description of female characters is based on two specimens collected in 1972 and deposited in the CNC. Because those females were collected in the type locality, altogether with a male that closely resembles the holotype of *Apanteles vulgaris*, it is assumed that they all are the same species. The species is characterized by extensive yellow-orange coloration, smooth mesoscutellar disc, and relatively broad mediotergite 1 (its length 1.8 × its width at posterior margin). The known hosts (boring Crambidae, *Diatraea* spp.), are unique among all Mesoamerican species with extensive yellow-orange coloration.

### Apanteles wadyobandoi

Fernández-Triana sp. n.

http://zoobank.org/19EC8B7A-F82F-4150-AE24-625AD93EC52B

http://species-id.net/wiki/Apanteles_wadyobandoi

Figs 197 , 326

Apanteles Rodriguez97 (Smith et al. 2006). Interim name provided by the authors.

#### Type locality.

COSTA RICA, Alajuela, ACG, Sector San Cristobal, Rio Blanco Abajo, 500m, 10.90037, -85.37254.

#### Holotype.

♀ in CNC. Specimen labels: 1. DHJPAR0001677. <<oops, we have another problem here. This is a 369 bp specimen that trees in A. R23, when as you are indicating, it is very likely to be R97 from its host, etc. Cannot be holotype and probably should not be a paratype either (all the 01-SRNP-2944 specimens should not be paratypes unless you can do that morphologically). Do I assume correctly that it is morphologically R97? If so, I can live with it being a paratype if you like. DHJPAR0002205 and DHJPAR0002704 have good barcodes, so either one of them can be the holotype. And if left in R97, I definitely have to move it out of R23 in my DB (I will leave it there for the moment until you have told me what to do). Looks like in the end this kind of process will remove all four of the R23 Urbanus (very good), though I should have it clear where you want them morphologically before doing that.

#### Paratypes.

2 ♀, 5 ♂ (CNC, NMNH). COSTA RICA: ACG database codes: DHJPAR0002205, DHJPAR0002704, 01-SRNP-2944.

#### Description.

**Female.** Metatibia color (outer face): with extended pale coloration (light yellow to orange–yellow), ranging from 0.4 to almost entire metatibia length. Fore wing veins color: veins C+Sc+R and R1 mostly brown; usually veins r, 2RS, 2M, (RS+M)b, 1CU, 2Cua, and 1m–cu partially brown; interior area of other veins, and at least part of pterostigma, usually light brown or yellowish–white. Antenna length/body length: antenna about as long as body (head to apex of metasoma); if slightly shorter, at least extending beyond anterior 0.7 metasoma length. Body length (head to apex of metasoma): 2.3–2.4 mm or 2.5–2.6 mm. Fore wing length: 2.5–2.6 mm or 2.7–2.8 mm. Metafemur length/width: 3.0–3.1. Mediotergite 1 length/width at posterior margin: 2.3–2.4. Mediotergite 1 maximum width/width at posterior margin: 1.4–1.5. Ovipositor sheaths length/metafemur length: 0.6. Ovipositor sheaths length/metatibia length: 0.5.

#### Molecular data.

Sequences in BOLD: 3, barcode compliant sequences: 2.

#### Biology/ecology.

Gregarious (Fig. 326). Hosts: Hesperiidae, *Urbanus dorantes*, *Urbanus teleus*.

#### Distribution.

Costa Rica, ACG.

#### Etymology.

We dedicate this species to Wady Obando in recognition of his diligent efforts for the ACG Programa de Sectores.

### Apanteles waldymedinai

Fernández-Triana sp. n.

http://zoobank.org/1A100843-B486-43C8-9AD7-388350729762

http://species-id.net/wiki/Apanteles_waldymedinai

Fig. 163

#### Type locality.

COSTA RICA, Alajuela, ACG, Sector San Cristobal, Rio Blanco Abajo, 500m, 10.90037, -85.37254.

#### Holotype.

♀ in CNC. Specimen labels: 1. DHJPAR0025256. 2. San Gerardo, Rio Blanco Abajo, Date: 1-7 Dec. 2007.

#### Paratypes.

1 ♂ (CNC). COSTA RICA, ACG database code: DHJPAR0026834.

#### Description.

**Female.** Body color: head pale, mesosoma extensively pale (anteromesoscutum and scutellar disc). Antenna color: scape, pedicel, and flagellum dark. Coxae color (pro-, meso-, metacoxa): pale, pale, partially pale/partially dark. Femora color (pro-, meso-, metafemur): pale, anteriorly dark/posteriorly pale, anteriorly dark/posteriorly pale. Tibiae color (pro-, meso-, metatibia): pale, anteriorly pale/posteriorly dark, anteriorly pale/posteriorly dark. Tegula and humeral complex color: both pale. Pterostigma color: dark. Fore wing veins color: mostly dark (a few veins may be unpigmented). Antenna length/body length: antenna about as long as body (head to apex of metasoma); if slightly shorter, at least extending beyond anterior 0.7 metasoma length. Body in lateral view: not distinctly flattened dorso–ventrally. Body length (head to apex of metasoma): 3.1–3.2 mm. Fore wing length: 3.3–3.4 mm. Ocular–ocellar line/posterior ocellus diameter: 2.0–2.2. Interocellar distance/posterior ocellus diameter: 1.7–1.9. Antennal flagellomerus 2 length/width: 2.9–3.1. Antennal flagellomerus 14 length/width: 1.4–1.6. Length of flagellomerus 2/length of flagellomerus 14: 2.3–2.5. Tarsal claws: pectinate. Metafemur length/width: 3.0–3.1. Anteromesoscutum: mostly with shallow, sparse punctures (separated by more than 2.0 × its maximum diameter). Mesoscutellar disc: mostly smooth. Number of pits in scutoscutellar sulcus: 7 or 8. Maximum height of mesoscutellum lunules/maximum height of lateral face of mesoscutellum: 0.4–0.5. Propodeum areola: completely defined by carinae, including transverse carina extending to spiracle. Propodeum background sculpture: mostly sculptured. Mediotergite 1 length/width at posterior margin: 1.7–1.9. Mediotergite 1 shape: mostly parallel–sided for 0.5–0.7 of its length, then narrowing posteriorly so mediotergite anterior width >1.1 × posterior width. Mediotergite 1 sculpture: mostly sculptured, excavated area centrally with transverse striation inside and/or a polished knob centrally on posterior margin of mediotergite. Mediotergite 2 width at posterior margin/length: 4.8 or more. Mediotergite 2 sculpture: mostly smooth. Outer margin of hypopygium: with a wide, medially folded, transparent, semi–desclerotized area; usually with 4 or more pleats. Ovipositor thickness: about same width throughout its length. Length of fore wing veins r/2RS: 1.1–1.3. Length of fore wing veins 2RS/2M: 1.4–1.6. Length of fore wing veins 2M/(RS+M)b: 0.9–1.0. Pterostigma length/width: 3.1–3.5. Point of insertion of vein r in pterostigma: about half way point length of pterostigma. Angle of vein r with fore wing anterior margin: clearly outwards, inclined towards fore wing apex. Shape of junction of veins r and 2RS in fore wing: strongly angulated, sometimes with a knob.

**Male.** Similar to female, except for mediotergite 1 narrowing towards posterior end.

#### Molecular data.

Sequences in BOLD: 2, barcode compliant sequences: 2.

#### Biology/ecology.

Malaise-trapped.

#### Distribution.

Costa Rica, ACG.

#### Comments.

This species is uniquely among all other Mesoamerican *Apanteles* by its orange head, most of mesopleuron and mesosternum dark brown to black, and pectinate claws.

#### Etymology.

We dedicate this species to Waldy Medina in recognition of his diligent efforts for the ACG Programa de GIS and mapping.

### Apanteles wilbertharayai

Fernández-Triana sp. n.

http://zoobank.org/848A13C9-31A8-4C96-95B4-7CB0BB3E0DC8

http://species-id.net/wiki/Apanteles_wilbertharayai

Figs 20 , 222

#### Type locality.

COSTA RICA, Alajuela, ACG, Sector San Cristobal, Quebrada Cementerio, 700m, 10.87124, -85.38749.

#### Holotype.

♀ in CNC. Specimen labels: 1. DHJPAR0039765. 2. Voucher: D.H.Janzen & W.Hallwachs, DB: http://janzen.sas.upenn.edu, Area de Conservación Guanacaste, COSTA RICA, 09-SRNP-3520.

#### Description.

**Female.** Body color: body mostly dark except for some sternites which may be pale. Antenna color: scape, pedicel, and flagellum dark. Coxae color (pro-, meso-, metacoxa): dark, dark, dark. Femora color (pro-, meso-, metafemur): anteriorly dark/posteriorly pale, dark, dark. Tibiae color (pro-, meso-, metatibia): pale, pale, anteriorly pale/posteriorly dark. Tegula and humeral complex color: tegula pale, humeral complex half pale/half dark. Pterostigma color: mostly pale and/or transparent, with thin dark borders. Fore wing veins color: mostly white or entirely transparent. Antenna length/body length: antenna about as long as body (head to apex of metasoma); if slightly shorter, at least extending beyond anterior 0.7 metasoma length. Body in lateral view: not distinctly flattened dorso–ventrally. Body length (head to apex of metasoma): 3.1–3.2 mm. Fore wing length: 3.3–3.4 mm. Ocular–ocellar line/posterior ocellus diameter: 2.3–2.5. Interocellar distance/posterior ocellus diameter: 2.3–2.5. Antennal flagellomerus 2 length/width: 2.6–2.8. Antennal flagellomerus 14 length/width: 1.4–1.6. Length of flagellomerus 2/length of flagellomerus 14: 2.0–2.2. Tarsal claws: simple. Metafemur length/width: 3.0–3.1. Metatibia inner spur length/metabasitarsus length: 0.4–0.5. Anteromesoscutum: mostly with shallow, dense punctures (separated by less than 2.0 × its maximum diameter). Mesoscutellar disc: with punctures near margins, central part mostly smooth. Number of pits in scutoscutellar sulcus: 9 or 10. Maximum height of mesoscutellum lunules/maximum height of lateral face of mesoscutellum: 0.6–0.7. Propodeum areola: completely defined by carinae, including transverse carina extending to spiracle. Propodeum background sculpture: mostly sculptured. Mediotergite 1 length/width at posterior margin: 1.4–1.6. Mediotergite 1 shape: clearly widening towards posterior margin. Mediotergite 1 sculpture: mostly sculptured, excavated area centrally with transverse striation inside and/or a polished knob centrally on posterior margin of mediotergite. Mediotergite 2 width at posterior margin/length: 4.4–4.7. Mediotergite 2 sculpture: with some sculpture, mostly near posterior margin. Outer margin of hypopygium: with a wide, medially folded, transparent, semi–desclerotized area; usually with 4 or more pleats. Ovipositor sheaths length/metatibial length: 1.4–1.5. Length of fore wing veins r/2RS: 2.3 or more. Length of fore wing veins 2RS/2M: 1.1–1.3. Length of fore wing veins 2M/(RS+M)b: 0.7–0.8. Pterostigma length/width: 3.1–3.5. Point of insertion of vein r in pterostigma: clearly beyond half way point length of pterostigma. Angle of vein r with fore wing anterior margin: more or less perpendicular to fore wing margin. Shape of junction of veins r and 2RS in fore wing: distinctly but not strongly angled.

**Male.** Unknown.

#### Molecular data.

Sequences in BOLD: 1, barcode compliant sequences: 1.

#### Biology/ecology.

Solitary (Fig. 222). Host: Elachistidae, *Anadasmus* Janzen28.

#### Distribution.

Costa Rica, ACG.

#### Etymology.

We dedicate this species to Wilberth Araya in recognition of his diligent efforts for the ACG Programa de Sectores.

### Apanteles williamcamposi

Fernández-Triana sp. n.

http://zoobank.org/283C2948-F696-4316-84C5-85127FE8D741

http://species-id.net/wiki/Apanteles_williamcamposi

Figs 95 , 274

#### Type locality.

COSTA RICA, Alajuela, ACG, Sector Pitilla, Leonel, 510m, 10.99637, -85.40195.

#### Holotype.

♀ in CNC. Specimen labels: 1. DHJPAR0039768.

#### Paratypes.

1 ♂ (CNC). COSTA RICA: Guanacaste, ACG database code: DHJPAR0039760.

#### Description.

**Female.** Body color: head dark, mesosoma dark with parts of axillar complex pale, metasoma with some mediotergites, most laterotergites, sternites, and/or hypopygium pale. Antenna color: scape and/or pedicel pale, flagellum dark. Coxae color (pro-, meso-, metacoxa): pale, pale, pale. Femora color (pro-, meso-, metafemur): pale, pale, pale. Tibiae color (pro-, meso-, metatibia): pale, pale, mostly pale but with posterior 0.2 or less dark. Tegula and humeral complex color: both pale. Pterostigma color: dark. Fore wing veins color: mostly dark (a few veins may be unpigmented). Antenna length/body length: antenna about as long as body (head to apex of metasoma); if slightly shorter, at least extending beyond anterior 0.7 metasoma length. Body in lateral view: not distinctly flattened dorso–ventrally. Body length (head to apex of metasoma): 3.1–3.2 mm. Fore wing length: 3.3–3.4 mm. Ocular–ocellar line/posterior ocellus diameter: 1.7–1.9. Interocellar distance/posterior ocellus diameter: 2.0–2.2. Antennal flagellomerus 2 length/width: 2.9–3.1. Antennal flagellomerus 14 length/width: 1.7–1.9. Length of flagellomerus 2/length of flagellomerus 14: 2.3–2.5. Tarsal claws: simple (?). Metafemur length/width: 2.8–2.9. Metatibia inner spur length/metabasitarsus length: 0.4–0.5. Anteromesoscutum: mostly with deep, dense punctures (separated by less than 2.0 × its maximum diameter). Mesoscutellar disc: with punctures near margins, central part mostly smooth. Number of pits in scutoscutellar sulcus: 5 or 6. Maximum height of mesoscutellum lunules/maximum height of lateral face of mesoscutellum: 0.2–0.3. Propodeum areola: completely defined by carinae, including transverse carina extending to spiracle. Propodeum background sculpture: mostly sculptured. Mediotergite 1 length/width at posterior margin: 2.9–3.1. Mediotergite 1 shape: clearly narrowing towards posterior margin. Mediotergite 1 sculpture: with some sculpture near lateral margins and/or posterior 0.2–0.4 of mediotergite. Mediotergite 2 width at posterior margin/length: 2.4–2.7. Mediotergite 2 sculpture: mostly smooth. Outer margin of hypopygium: with a wide, medially folded, transparent, semi–desclerotized area; usually with 4 or more pleats. Ovipositor thickness: about same width throughout its length. Ovipositor sheaths length/metatibial length: 0.4–0.5. Length of fore wing veins r/2RS: 1.4–1.6. Length of fore wing veins 2RS/2M: 1.9–2.0. Length of fore wing veins 2M/(RS+M)b: 0.5–0.6. Pterostigma length/width: 2.6–3.0. Point of insertion of vein r in pterostigma: about half way point length of pterostigma. Angle of vein r with fore wing anterior margin: clearly outwards, inclined towards fore wing apex. Shape of junction of veins r and 2RS in fore wing: distinctly but not strongly angled.

**Male.** As in female, but tergites dark brown.

#### Molecular data.

Sequences in BOLD: 2, barcode compliant sequences: 2.

#### Biology/ecology.

Solitary (Fig. 274). Host: Elachistidae, elachJanzen01 Janzen281.

#### Distribution.

Costa Rica, ACG.

#### Etymology.

We dedicate this species to William Campos in recognition of his diligent efforts for the ACG Programa Forestal.

### Apanteles yeissonchavesi

Fernández-Triana sp. n.

http://zoobank.org/5BCBBCE9-6CBC-4E0E-89D5-318ADDCCFB6C

http://species-id.net/wiki/Apanteles_yeissonchavesi

Fig. 100

#### Type locality.

COSTA RICA, Alajuela, ACG, Sector San Cristobal, Rio Blanco Abajo, 500m, 10.90037, -85.37254.

#### Holotype.

♀ in CNC. Specimen labels: 1. DHJPAR0026887. 2. San Gerardo, Rio Blanco Abajo, 30 Mar-5 Apr/2008.

#### Description.

**Female.** Body color: body mostly dark except for some sternites which may be pale. Antenna color: scape and/or pedicel pale, flagellum dark. Coxae color (pro-, meso-, metacoxa): pale, pale, pale. Femora color (pro-, meso-, metafemur): pale, pale, mostly pale but posterior 0.2 or less dark. Tibiae color (pro-, meso-, metatibia): pale, pale, mostly pale but with posterior 0.2 or less dark. Tegula and humeral complex color: tegula dark, humeral complex pale. Pterostigma color: dark. Fore wing veins color: mostly dark (a few veins may be unpigmented). Antenna length/body length: antenna about as long as body (head to apex of metasoma); if slightly shorter, at least extending beyond anterior 0.7 metasoma length. Body in lateral view: not distinctly flattened dorso–ventrally. Body length (head to apex of metasoma): 2.3–2.4 mm. Fore wing length: 2.5–2.6 mm. Ocular–ocellar line/posterior ocellus diameter: 2.0–2.2. Interocellar distance/posterior ocellus diameter: 1.7–1.9. Antennal flagellomerus 2 length/width: 2.6–2.8. Antennal flagellomerus 14 length/width: 1.4–1.6. Length of flagellomerus 2/length of flagellomerus 14: 2.0–2.2. Tarsal claws: simple. Metafemur length/width: 3.0–3.1. Metatibia inner spur length/metabasitarsus length: 0.4–0.5. Anteromesoscutum: mostly with deep, dense punctures (separated by less than 2.0 × its maximum diameter). Mesoscutellar disc: mostly punctured. Number of pits in scutoscutellar sulcus: 5 or 6. Maximum height of mesoscutellum lunules/maximum height of lateral face of mesoscutellum: 0.4–0.5. Propodeum areola: completely defined by carinae, including transverse carina extending to spiracle. Propodeum background sculpture: mostly sculptured. Mediotergite 1 length/width at posterior margin: 3.5–3.7. Mediotergite 1 shape: mostly parallel–sided for 0.5–0.7 of its length, then narrowing posteriorly so mediotergite anterior width >1.1 × posterior width. Mediotergite 1 sculpture: with some sculpture near lateral margins and/or posterior 0.2–0.4 of mediotergite. Mediotergite 2 width at posterior margin/length: 3.2–3.5. Mediotergite 2 sculpture: mostly smooth. Outer margin of hypopygium: with a medially folded, transparent, semi–desclerotized area; with 0–3 pleats visible. Ovipositor thickness: anterior width at most 2.0 × posterior width (beyond ovipositor constriction). Ovipositor sheaths length/metatibial length: 0.4–0.5. Length of fore wing veins r/2RS: 1.4–1.6. Length of fore wing veins 2RS/2M: 1.1–1.3. Length of fore wing veins 2M/(RS+M)b: 0.9–1.0. Pterostigma length/width: 3.1–3.5. Point of insertion of vein r in pterostigma: clearly beyond half way point length of pterostigma. Angle of vein r with fore wing anterior margin: clearly outwards, inclined towards fore wing apex. Shape of junction of veins r and 2RS in fore wing: strongly angulated, sometimes with a knob.

**Male.** Unknown.

#### Molecular data.

Sequences in BOLD: 6, barcode compliant sequences: 5.

#### Biology/ecology.

Malaise-trapped.

#### Distribution.

Costa Rica, ACG.

#### Etymology.

We dedicate this species to Yeisson Chávez in recognition of his diligent efforts for the ACG Programa de Computerizacion and Informatics.

### Apanteles yilbertalvaradoi

Fernández-Triana sp. n.

http://zoobank.org/54F00180-6557-4269-AC20-16A66411FB19

http://species-id.net/wiki/Apanteles_yilbertalvaradoi

Figs 31 , 231

Apanteles Rodriguez80 (Smith et al. 2006). Interim name provided by the authors.

#### Type locality.

COSTA RICA, Alajuela, ACG, Sector San Cristobal, Sendero Perdido, 620m, 10.8794, -85.38607.

#### Holotype.

♀ in CNC. Specimen labels: 1. Voucher: D.H.Janzen & W.Hallwachs, DB: http://janzen.sas.upenn.edu, Area de Conservación Guanacaste, COSTA RICA, 09-SRNP-1423. 2. DHJPAR0035358.

#### Paratypes.

1 ♀ (CNC, NMNH). COSTA RICA: Guanacaste, ACG database code: DHJPAR0038325.

#### Description.

**Female.** Body color: body mostly dark except for some sternites which may be pale. Antenna color: scape, pedicel, and flagellum dark. Coxae color (pro-, meso-, metacoxa): dark, dark, dark. Femora color (pro-, meso-, metafemur): anteriorly dark/posteriorly pale, dark, dark. Tibiae color (pro-, meso-, metatibia): pale, anteriorly pale/posteriorly dark, dark. Tegula and humeral complex color: tegula dark, humeral complex half pale/half dark. Pterostigma color: mostly pale and/or transparent, with thin dark borders. Fore wing veins color: partially pigmented (a few veins may be dark but most are pale). Antenna length/body length: antenna about as long as body (head to apex of metasoma); if slightly shorter, at least extending beyond anterior 0.7 metasoma length. Body in lateral view: not distinctly flattened dorso–ventrally. Body length (head to apex of metasoma): 2.5–2.6 mm. Fore wing length: 2.7–2.8 mm. Ocular–ocellar line/posterior ocellus diameter: 2.6 or more. Interocellar distance/posterior ocellus diameter: 2.3–2.5. Antennal flagellomerus 2 length/width: 2.6–2.8. Antennal flagellomerus 14 length/width: 1.4–1.6. Length of flagellomerus 2/length of flagellomerus 14: 2.0–2.2. Tarsal claws: simple. Metafemur length/width: 3.0–3.1. Metatibia inner spur length/metabasitarsus length: 0.4–0.5. Anteromesoscutum: mostly with deep, dense punctures (separated by less than 2.0 × its maximum diameter). Mesoscutellar disc: mostly smooth. Number of pits in scutoscutellar sulcus: 9 or 10. Maximum height of mesoscutellum lunules/maximum height of lateral face of mesoscutellum: 0.6–0.7. Propodeum areola: completely defined by carinae, including transverse carina extending to spiracle. Propodeum background sculpture: partly sculptured, especially on anterior 0.5. Mediotergite 1 length/width at posterior margin: 1.4–1.6. Mediotergite 1 shape: more or less parallel–sided. Mediotergite 1 sculpture: mostly sculptured, excavated area centrally with transverse striation inside and/or a polished knob centrally on posterior margin of mediotergite. Mediotergite 2 width at posterior margin/length: 4.8 or more. Mediotergite 2 sculpture: mostly smooth. Outer margin of hypopygium: with a wide, medially folded, transparent, semi–desclerotized area; usually with 4 or more pleats. Ovipositor thickness: about same width throughout its length. Ovipositor sheaths length/metatibial length: 1.0–1.1. Length of fore wing veins r/2RS: 1.4–1.6. Length of fore wing veins 2RS/2M: 1.4–1.6. Length of fore wing veins 2M/(RS+M)b: 0.5–0.6. Pterostigma length/width: 3.1–3.5. Point of insertion of vein r in pterostigma: about half way point length of pterostigma. Angle of vein r with fore wing anterior margin: more or less perpendicular to fore wing margin. Shape of junction of veins r and 2RS in fore wing: strongly angulated, sometimes with a knob.

**Male.** Unknown.

#### Molecular data.

Sequences in BOLD: 8, barcode compliant sequences: 8.

#### Biology/ecology.

Solitary (Fig. 231). Hosts: Tortricidae, *Episimus ortygia*.

#### Distribution.

Costa Rica, ACG.

#### Etymology.

We dedicate this species to Yilbert Alvarado in recognition of his diligent efforts for the ACG Programa de Seguridad.

### Apanteles yolandarojasae

Fernández-Triana sp. n.

http://zoobank.org/7A6C72FF-757B-4C6C-A674-1D59FF6A51BF

http://species-id.net/wiki/Apanteles_yolandarojasae

Figs 21 , 223

Apanteles Rodriguez168. Smith et al. (2008). Interim name provided by the authors.

#### Type locality.

COSTA RICA, Guanacaste, ACG, Sector Mundo Nuevo, Sendero Aguacate, 335m, 10.76901, -85.43465.

#### Holotype.

♀ in CNC. Specimen labels: 1. DHJPAR0012283. 2. Costa Rica: Guanacaste, ACG, Sector Mundo Nuevo, Sendero Aguacate, 14.ix.2006, 335m, 10.76901, -85.43465, 06-SRNP-58299.

#### Paratypes.

6 ♀, 6 ♂ (BMNH, CNC, INBIO, INHS, NMNH). COSTA RICA, ACG database codes: DHJPAR0012996.

#### Description.

**Female.** Body color: body mostly dark except for some sternites which may be pale. Antenna color: scape, pedicel, and flagellum dark. Coxae color (pro-, meso-, metacoxa): dark, dark, dark. Femora color (pro-, meso-, metafemur): anteriorly dark/posteriorly pale, dark, dark. Tibiae color (pro-, meso-, metatibia): pale, pale, dark. Tegula and humeral complex color: tegula pale, humeral complex half pale/half dark. Pterostigma color: mostly pale and/or transparent, with thin dark borders. Fore wing veins color: partially pigmented (a few veins may be dark but most are pale). Body in lateral view: not distinctly flattened dorso–ventrally. Body length (head to apex of metasoma): 2.1–2.2 mm, rarely 2.0 mm or less or 2.7–2.8 mm. Fore wing length: 2.3–2.4 mm, rarely 2.1–2.2 mm or 2.9–3.0 mm. Ocular–ocellar line/posterior ocellus diameter: 2.0–2.2. Interocellar distance/posterior ocellus diameter: 1.7–1.9. Antennal flagellomerus 2 length/width: 2.6–2.8. Antennal flagellomerus 14 length/width: 1.4–1.6. Length of flagellomerus 2/length of flagellomerus 14: 2.0–2.2. Tarsal claws: with single basal spine–like seta. Metafemur length/width: 2.6–2.7. Metatibia inner spur length/metabasitarsus length: 0.4–0.5. Anteromesoscutum: mostly with deep, dense punctures (separated by less than 2.0 × its maximum diameter). Mesoscutellar disc: with punctures near margins, central part mostly smooth. Number of pits in scutoscutellar sulcus: 7 or 8. Maximum height of mesoscutellum lunules/maximum height of lateral face of mesoscutellum: 0.4–0.5. Propodeum areola: completely defined by carinae, including transverse carina extending to spiracle. Propodeum background sculpture: mostly sculptured. Mediotergite 1 length/width at posterior margin: 2.0–2.2. Mediotergite 1 shape: more or less parallel–sided or mostly parallel–sided for 0.5–0.7 of its length, then narrowing posteriorly so mediotergite anterior width >1.1 × posterior width. Mediotergite 1 sculpture: mostly sculptured, excavated area centrally with transverse striation inside and/or a polished knob centrally on posterior margin of mediotergite. Mediotergite 2 width at posterior margin/length: 4.4–4.7. Mediotergite 2 sculpture: mostly smooth. Outer margin of hypopygium: with a wide, medially folded, transparent, semi–desclerotized area; usually with 4 or more pleats. Ovipositor thickness: about same width throughout its length. Ovipositor sheaths length/metatibial length: 1.0–1.1. Length of fore wing veins r/2RS: 1.7–1.9. Length of fore wing veins 2RS/2M: 1.1–1.3. Length of fore wing veins 2M/(RS+M)b: 0.7–0.8. Pterostigma length/width: 3.1–3.5. Point of insertion of vein r in pterostigma: clearly beyond half way point length of pterostigma. Angle of vein r with fore wing anterior margin: more or less perpendicular to fore wing margin. Shape of junction of veins r and 2RS in fore wing: distinctly but not strongly angled.

**Male.** As in female but with slightly narrower mediotergite 1.

#### Molecular data.

Sequences in BOLD: 8, barcode compliant sequences: 8.

#### Biology/ecology.

Gregarious (Fig. 223). Hosts: Pyralidae, *Epidelia damia*; Elachistidae, *Antaeotricha* Janzen90, *Antaeotricha* Janzen150, *Goniotermia latipennis*, elachJanzen01 Janze212.

#### Distribution.

Costa Rica, ACG.

#### Etymology.

We dedicate this species to Yolanda Rojas in recognition of her diligent efforts for the ACG Programa de Ecoturismo.

### Apanteles zeneidabolanosae

Fernández-Triana sp. n.

http://zoobank.org/1DC9A0A3-F69B-4C24-8134-5563DB765CF2

http://species-id.net/wiki/Apanteles_zeneidabolanosae

Figs 22 , 224

Apanteles Rodriguez158. Smith et al. (2008). Interim name provided by the authors.

#### Type locality.

COSTA RICA, Alajuela, ACG, Sector San Cristobal, Jardin Estrada, 722m, 10.86546, -85.39694.

#### Holotype.

♀ in CNC. Specimen labels: 1. DHJPAR0038295. 2. Voucher: D.H.Janzen & W.Hallwachs, DB: http://janzen.sas.upenn.edu, Area de Conservación Guanacaste, COSTA RICA, 10-SRNP-455.

#### Paratypes.

39 ♀, 10 ♂ (BMNH, CNC, INBIO, INHS, NMNH). COSTA RICA, ACG database codes: See Appendix 2 for detailed label data.

#### Description.

**Female.** Body color: body mostly dark except for some sternites which may be pale. Antenna color: scape, pedicel, and flagellum dark. Coxae color (pro-, meso-, metacoxa): dark, dark, dark. Femora color (pro-, meso-, metafemur): anteriorly dark/posteriorly pale, dark, dark. Tibiae color (pro-, meso-, metatibia): pale, pale, mostly dark but anterior 0.2 or less pale. Tegula and humeral complex color: tegula pale, humeral complex half pale/half dark. Pterostigma color: mostly pale and/or transparent, with thin dark borders. Fore wing veins color: partially pigmented (a few veins may be dark but most are pale). Antenna length/body length: antenna about as long as body (head to apex of metasoma); if slightly shorter, at least extending beyond anterior 0.7 metasoma length. Body in lateral view: not distinctly flattened dorso–ventrally. Body length (head to apex of metasoma): 2.9–3.0 mm or 3.1–3.2 mm. Fore wing length: 3.1–3.2 mm, 3.3–3.4 mm, rarely 2.9–3.0 mm. Ocular–ocellar line/posterior ocellus diameter: 2.0–2.2. Interocellar distance/posterior ocellus diameter: 1.4–1.6. Antennal flagellomerus 2 length/width: 2.0–2.2. Antennal flagellomerus 14 length/width: 1.4–1.6. Length of flagellomerus 2/length of flagellomerus 14: 2.0–2.2. Tarsal claws: simple. Metafemur length/width: 3.0–3.1. Metatibia inner spur length/metabasitarsus length: 0.6–0.7. Anteromesoscutum: mostly with deep, dense punctures (separated by less than 2.0 × its maximum diameter). Mesoscutellar disc: mostly smooth. Number of pits in scutoscutellar sulcus: 7 or 8. Maximum height of mesoscutellum lunules/maximum height of lateral face of mesoscutellum: 0.6–0.7. Propodeum areola: completely defined by carinae, but only partial or absent transverse carina. Propodeum background sculpture: mostly sculptured. Mediotergite 1 length/width at posterior margin: 2.0–2.2. Mediotergite 1 shape: more or less parallel–sided. Mediotergite 1 sculpture: mostly sculptured, excavated area centrally with transverse striation inside and/or a polished knob centrally on posterior margin of mediotergite. Mediotergite 2 width at posterior margin/length: 2.8–3.1. Mediotergite 2 sculpture: with some sculpture, mostly near posterior margin. Outer margin of hypopygium: with a wide, medially folded, transparent, semi–desclerotized area; usually with 4 or more pleats. Ovipositor thickness: about same width throughout its length. Ovipositor sheaths length/metatibial length: 1.4–1.5. Length of fore wing veins r/2RS: 1.1–1.3. Length of fore wing veins 2RS/2M: 1.4–1.6. Length of fore wing veins 2M/(RS+M)b: 0.9–1.0. Pterostigma length/width: 3.1–3.5. Point of insertion of vein r in pterostigma: clearly beyond half way point length of pterostigma. Angle of vein r with fore wing anterior margin: clearly outwards, inclined towards fore wing apex. Shape of junction of veins r and 2RS in fore wing: distinctly but not strongly angled.

**Male.** As in female.

#### Molecular data.

Sequences in BOLD: 7, barcode compliant sequences: 7.

#### Biology/ecology.

Gregarious (Fig. 224). Host: Elachistidae, *Lethata trochalosticta*.

#### Distribution.

Costa Rica, ACG.

#### Etymology.

We dedicate this species to Zeneida Bolaños in recognition of her diligent efforts for the ACG Office in Pocosol.

## Plates

**Figure 4.**
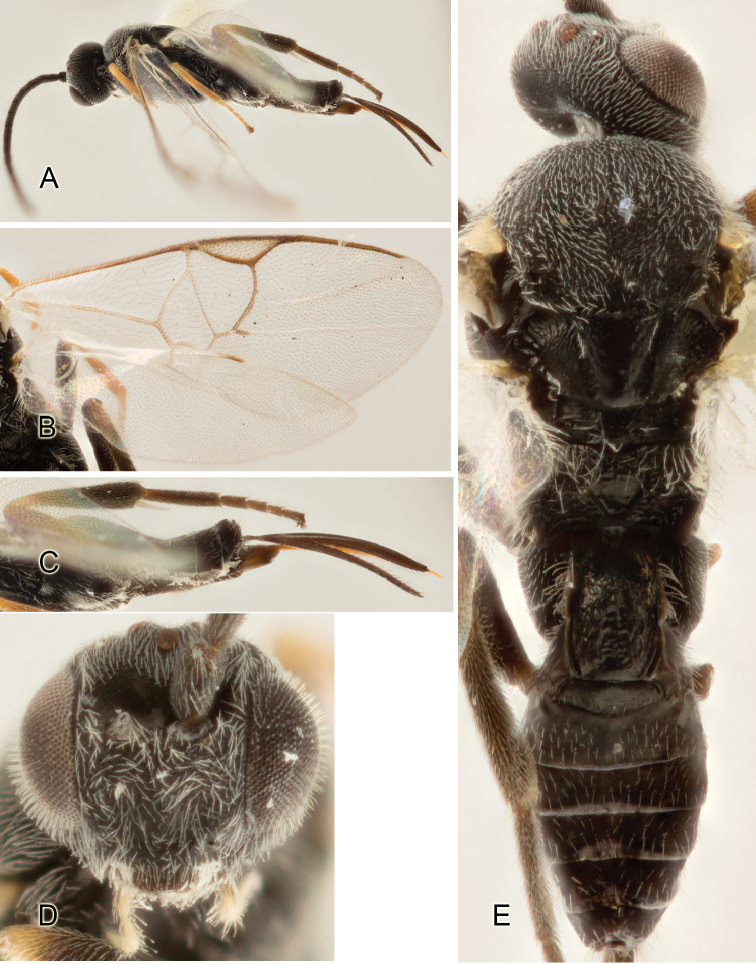
*Apanteles adelinamoralesae*. **A** Habitus, lateral view **B** Fore wing **C** Metasoma, lateral view **D** Head, frontal view **E** Head, meso- and metasoma, dorsal view.

**Figure 5.**
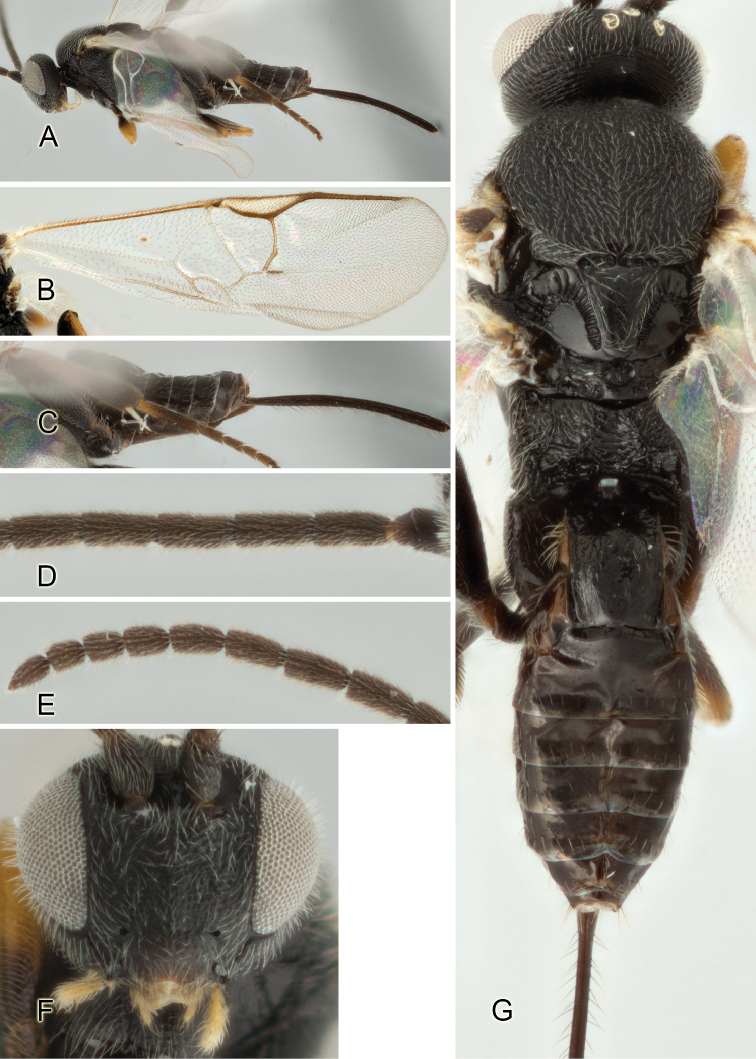
*Apanteles carloscastilloi*. **A** Habitus, lateral view **B** Fore wing **C** Metasoma, lateral view **D** Anterior half of antenna **E** Posterior half of antenna **F** Head, frontal view **G** Head, meso- and metasoma, dorsal view.

**Figure 6.**
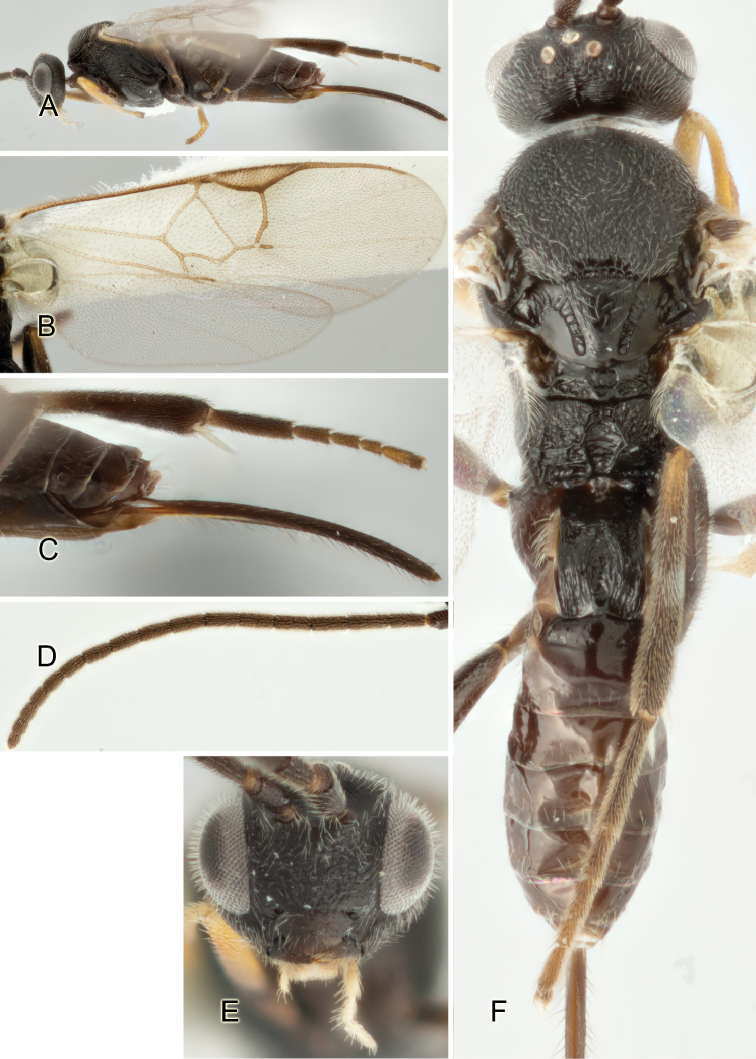
*Apanteles didiguadamuzi*. **A** Habitus, lateral view **B** Fore wing **C** Hypopygium and ovipositor sheats **D** Antenna **E** Head, frontal view **F** Head, meso- and metasoma, dorsal view.

**Figure 7.**
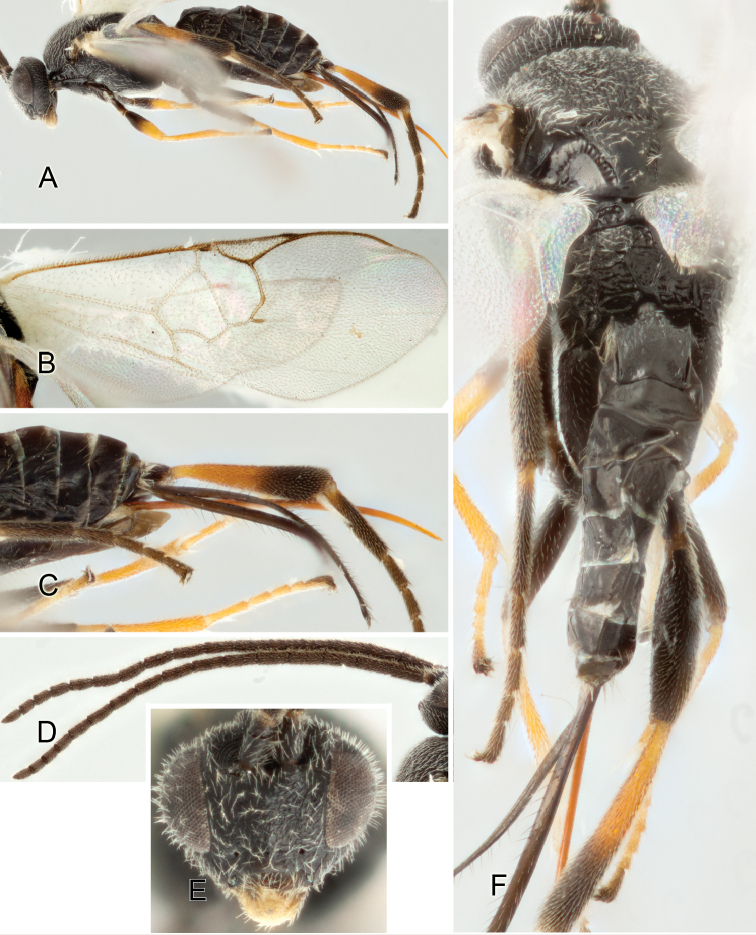
*Apanteles edgarjimenezi*. **A** Habitus, lateral view **B** Fore wing **C** Hypopygium and ovipositor sheats **D** Antenna **E** Head, frontal view **F** Head, meso- and metasoma, dorsal view.

**Figure 8.**
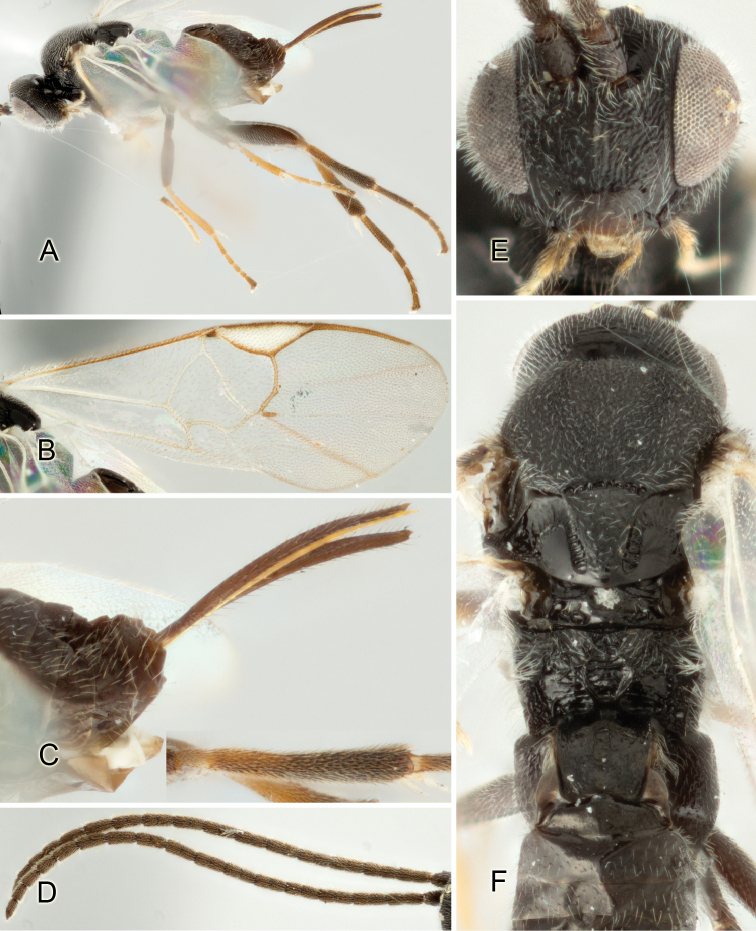
*Apanteles gerardosandovali*. **A** Habitus, lateral view **B** Fore wing **C** Hypopygium and ovipositor sheats **D** Antenna **E** Head, frontal view **F** Head, meso- and metasoma (partially), dorsal view.

**Figure 9.**
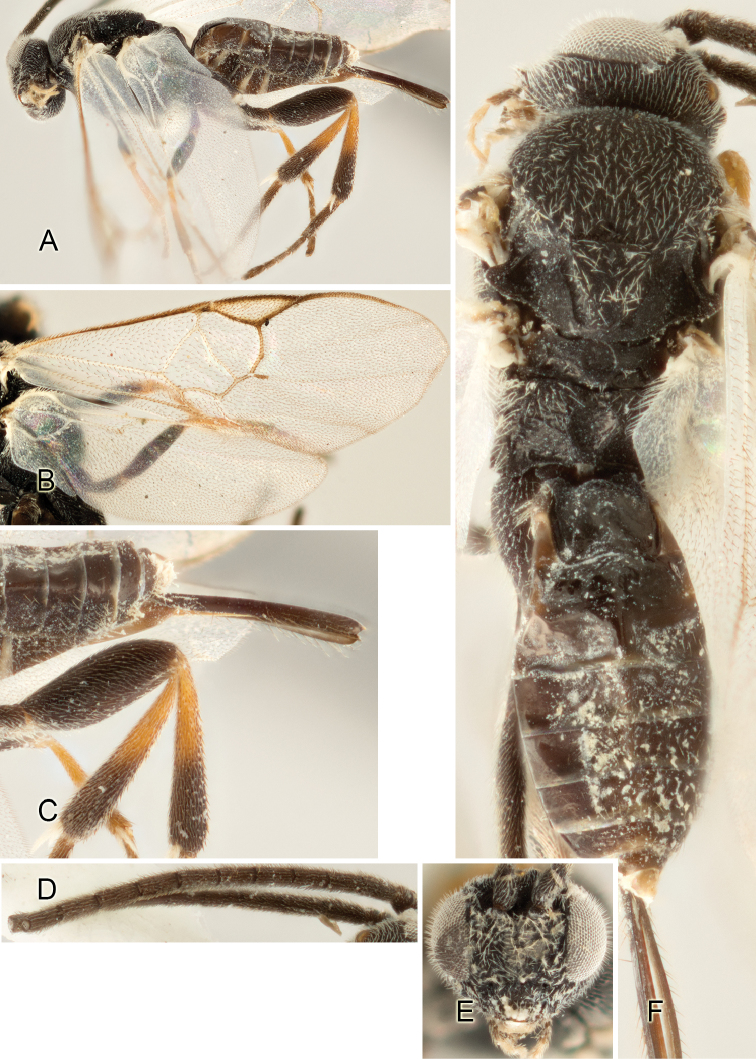
*Apanteles isaacbermudezi*. **A** Habitus, lateral view **B** Fore wing **C** Hypopygium and ovipositor sheats **D** Antenna **E** Head, frontal view **F** Head, meso- and metasoma, dorsal view.

**Figure 10.**
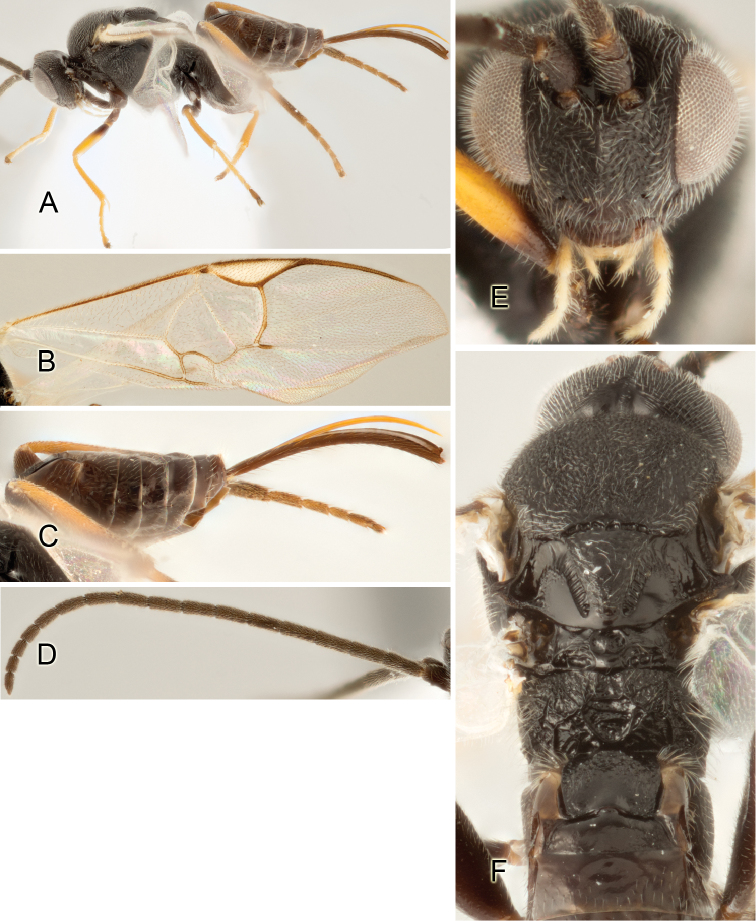
*Apanteles jorgecortesi*. **A** Habitus, lateral view **B** Fore wing **C** Hypopygium and ovipositor sheats **D** Antenna **E** Head, frontal view **F** Head, meso- and metasoma (partially), dorsal view.

**Figure 11.**
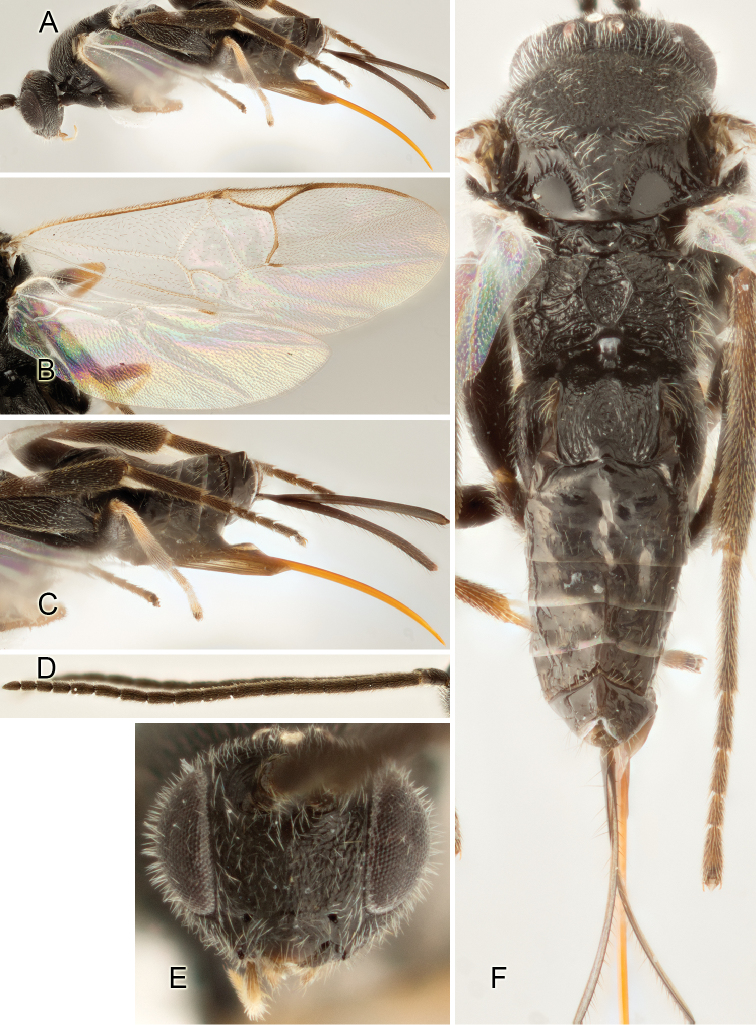
*Apanteles juanvictori*. **A** Habitus, lateral view **B** Fore wing **C** Hypopygium and ovipositor sheats **D** Antenna **E** Head, frontal view **F** Head, meso- and metasoma, dorsal view.

**Figure 12.**
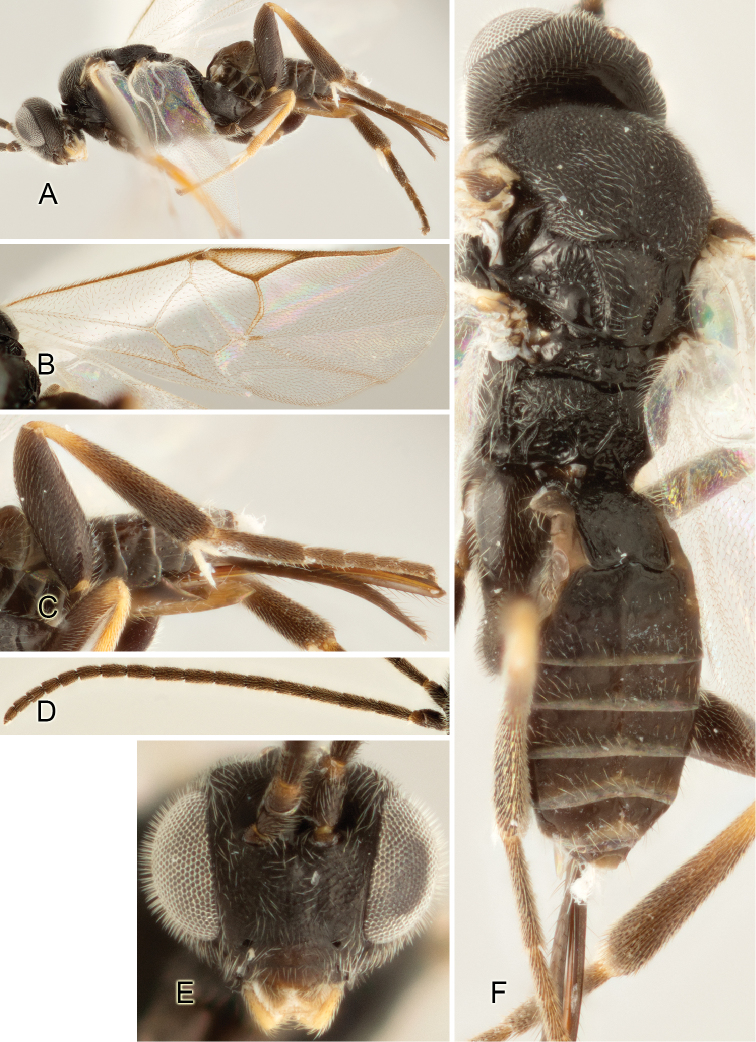
*Apanteles juniorlopezi*. **A** Habitus, lateral view **B** Fore wing **C** Hypopygium and ovipositor sheats **D** Antenna **E** Head, frontal view **F** Head, meso- and metasoma, dorsal view.

**Figure 13.**
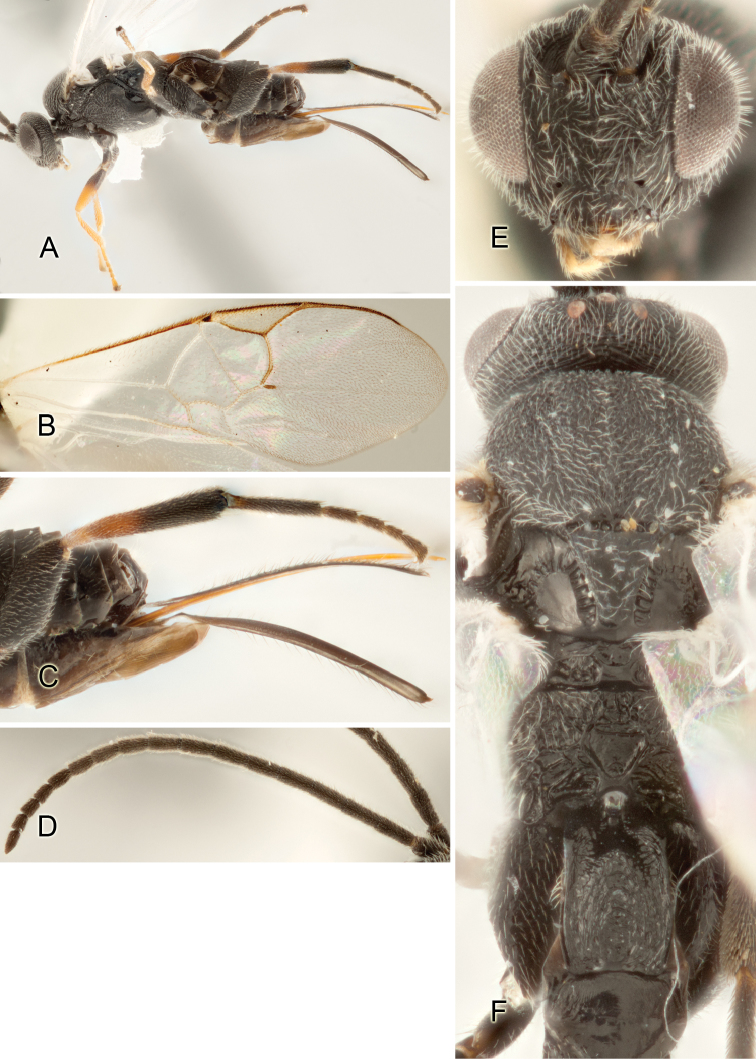
*Apanteles laurenmoralesae*. **A** Habitus, lateral view **B** Fore wing **C** Hypopygium and ovipositor sheats **D** Antenna **E** Head, frontal view **F** Head, meso- and metasoma (partially), dorsal view.

**Figure 14.**
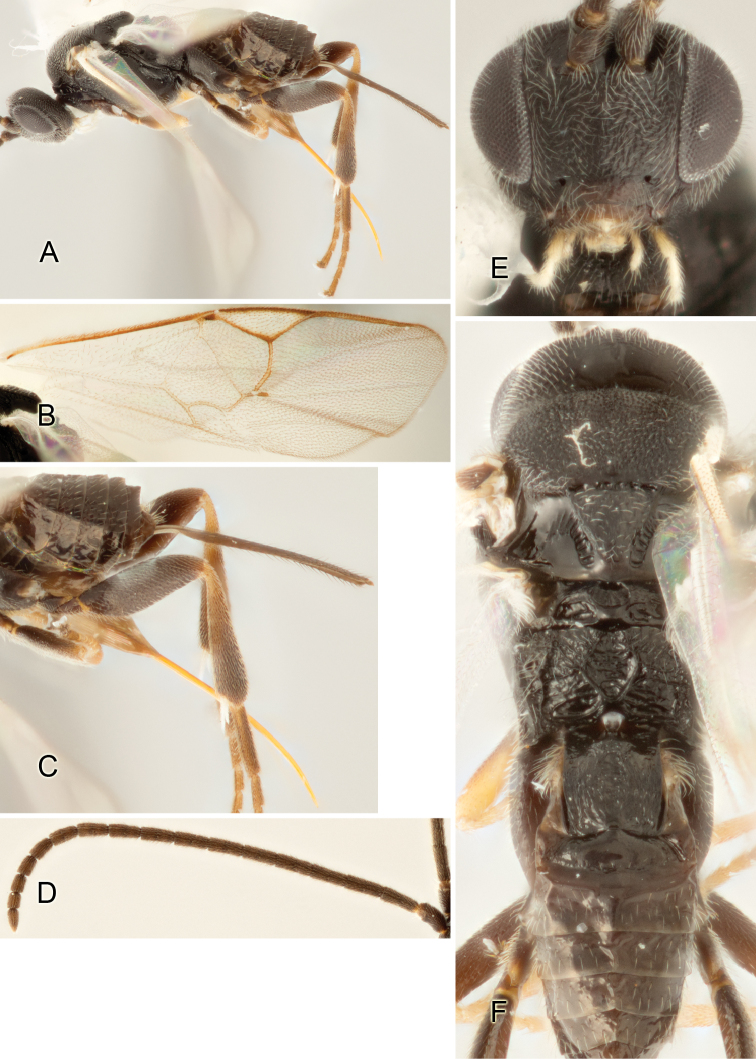
*Apanteles leninguadamuzi*. **A** Habitus, lateral view **B** Fore wing **C** Hypopygium and ovipositor sheats **D** Antenna **E** Head, frontal view **F** Head, meso- and metasoma, dorsal view.

**Figure 15.**
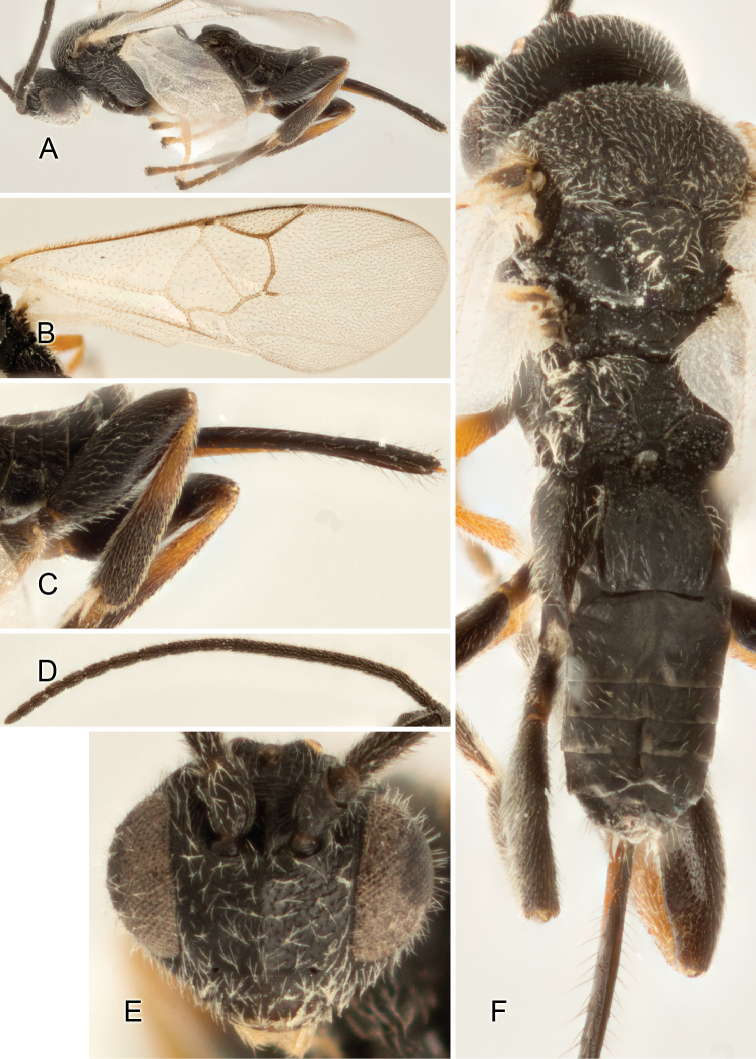
*Apanteles luiscanalesi*. **A** Habitus, lateral view **B** Fore wing **C** Hypopygium and ovipositor sheats **D** Antenna **E** Head, frontal view **F** Head, meso- and metasoma, dorsal view.

**Figure 16.**
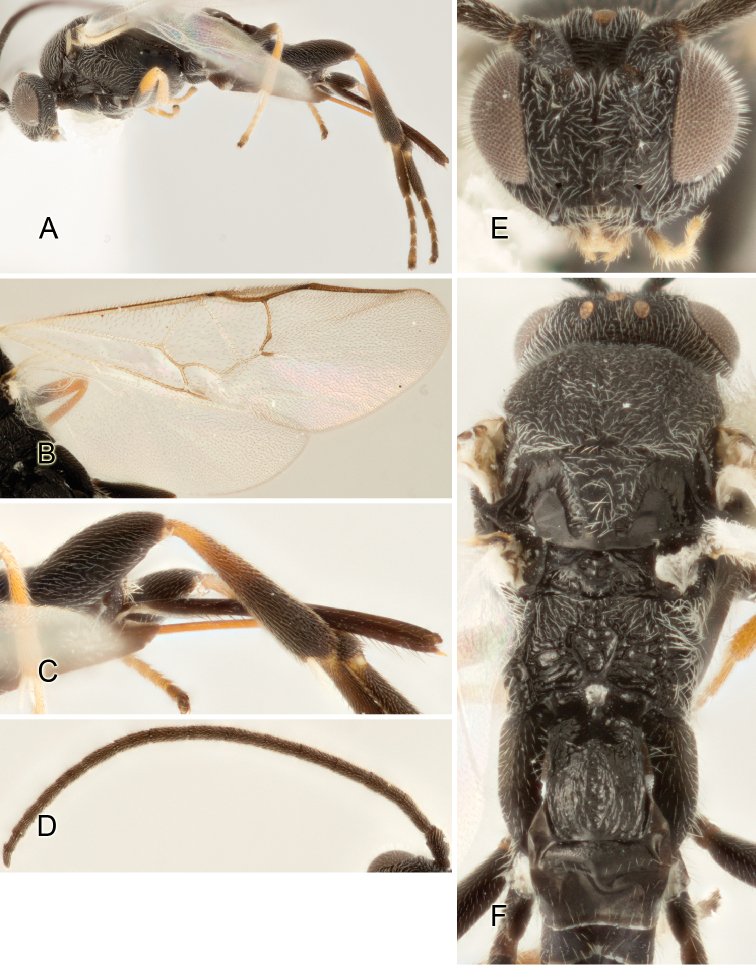
*Apanteles luislopezi*. **A** Habitus, lateral view **B** Fore wing **C** Hypopygium and ovipositor sheats **D** Antenna **E** Head, frontal view **F** Head, meso- and metasoma (partially), dorsal view.

**Figure 17.**
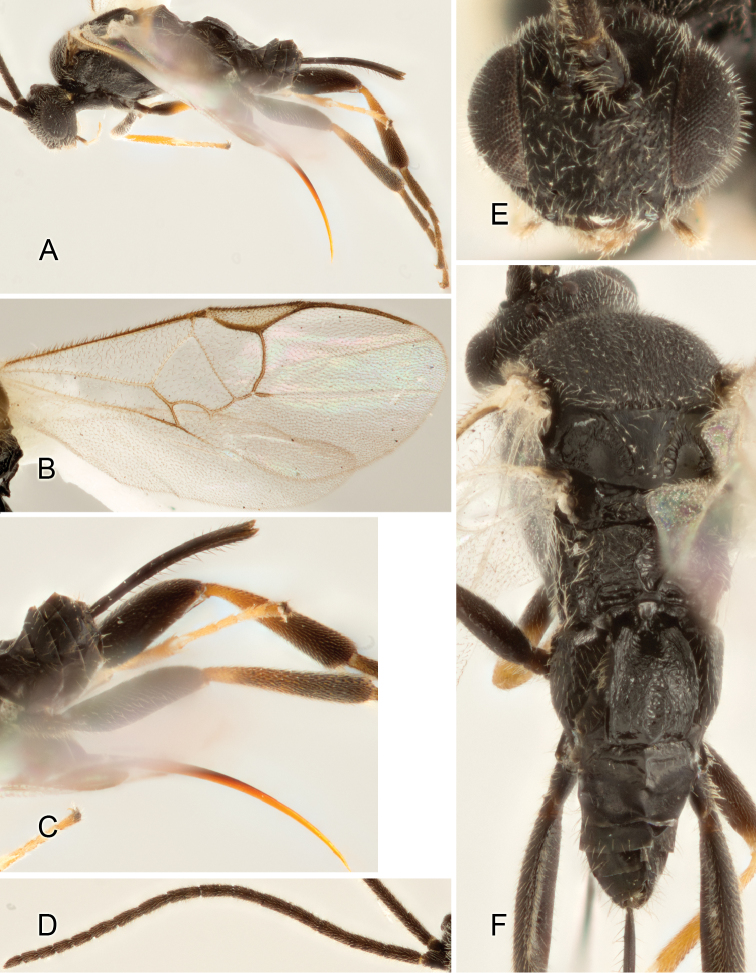
*Apanteles manuelarayai*. **A** Habitus, lateral view **B** Fore wing **C** Hypopygium and ovipositor sheats **D** Antenna **E** Head, frontal view **F** Head, meso- and metasoma, dorsal view.

**Figure 18.**
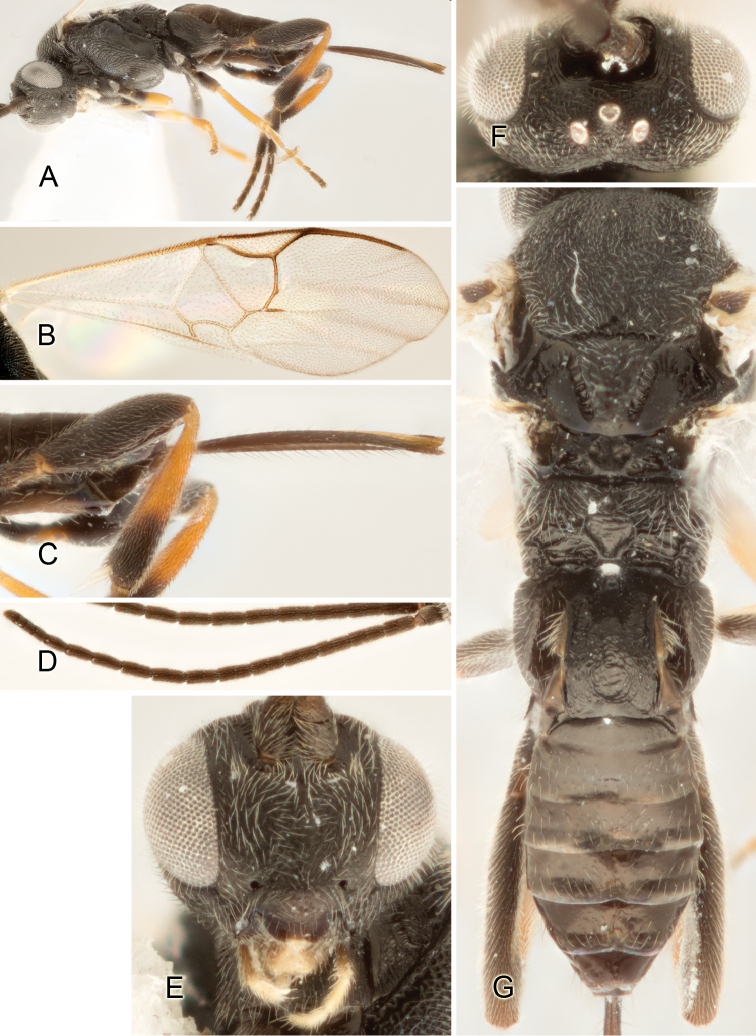
*Apanteles paulaixcamparijae*. **A** Habitus, lateral view **B** Fore wing **C** Hypopygium and ovipositor sheats **D** Antenna **E** Head, frontal view **F** Head, dorsal view **G** Meso- and metasoma, dorsal view.

**Figure 19.**
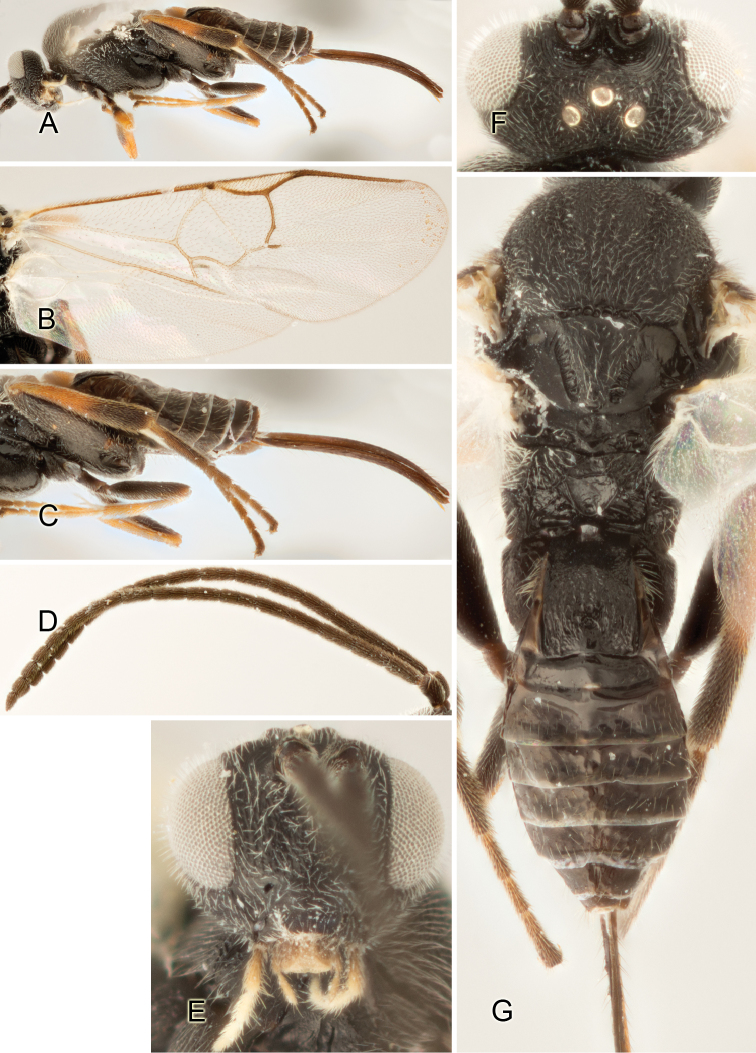
*Apanteles ronaldmurilloi*. **A** Habitus, lateral view **B** Fore wing **C** Hypopygium and ovipositor sheats **D** Antenna **E** Head, frontal view **F** Head, dorsal view **G** Meso- and metasoma, dorsal view.

**Figure 20.**
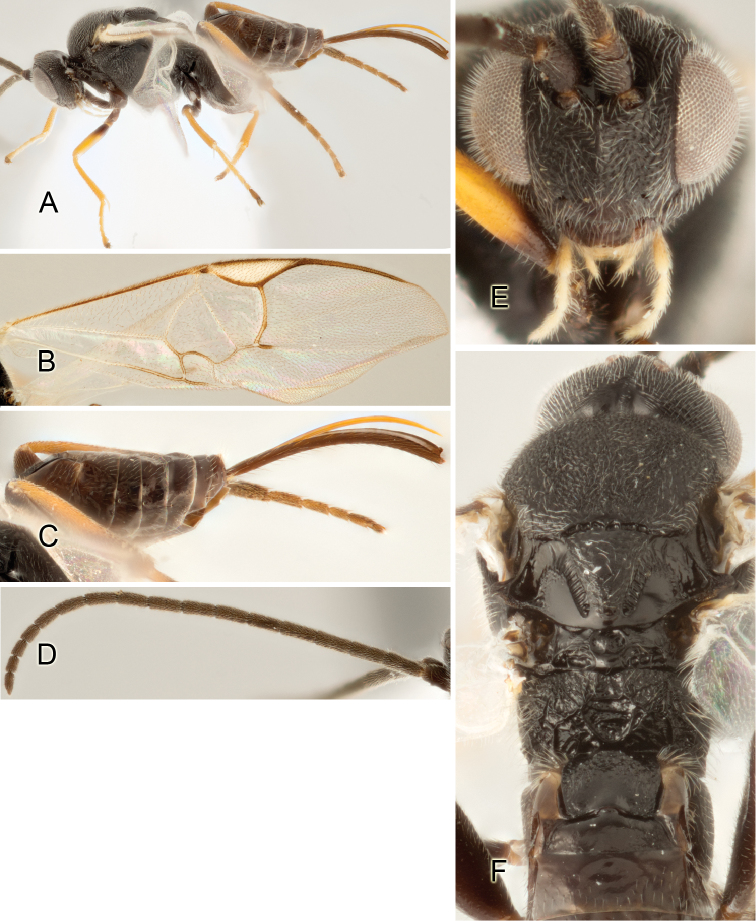
*Apanteles wilbertharayai*. **A** Habitus, lateral view **B** Fore wing **C** Hypopygium and ovipositor sheats **D** Antenna **E** Head, frontal view **F** Head, dorsal view **G** Meso- and metasoma, dorsal view.

**Figure 21.**
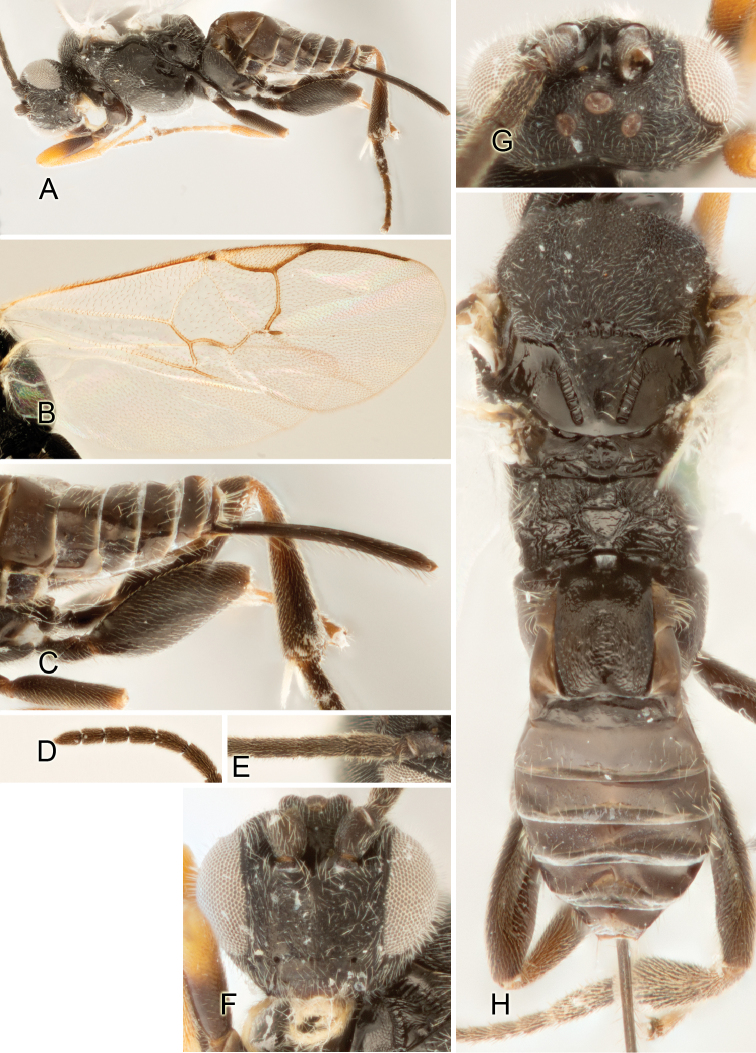
*Apanteles yolandarojasae*. **A** Habitus, lateral view **B** Fore wing **C** Hypopygium and ovipositor sheats **D** Antenna **E** Head, frontal view **F** Head, dorsal view **G** Meso- and metasoma, dorsal view.

**Figure 22.**
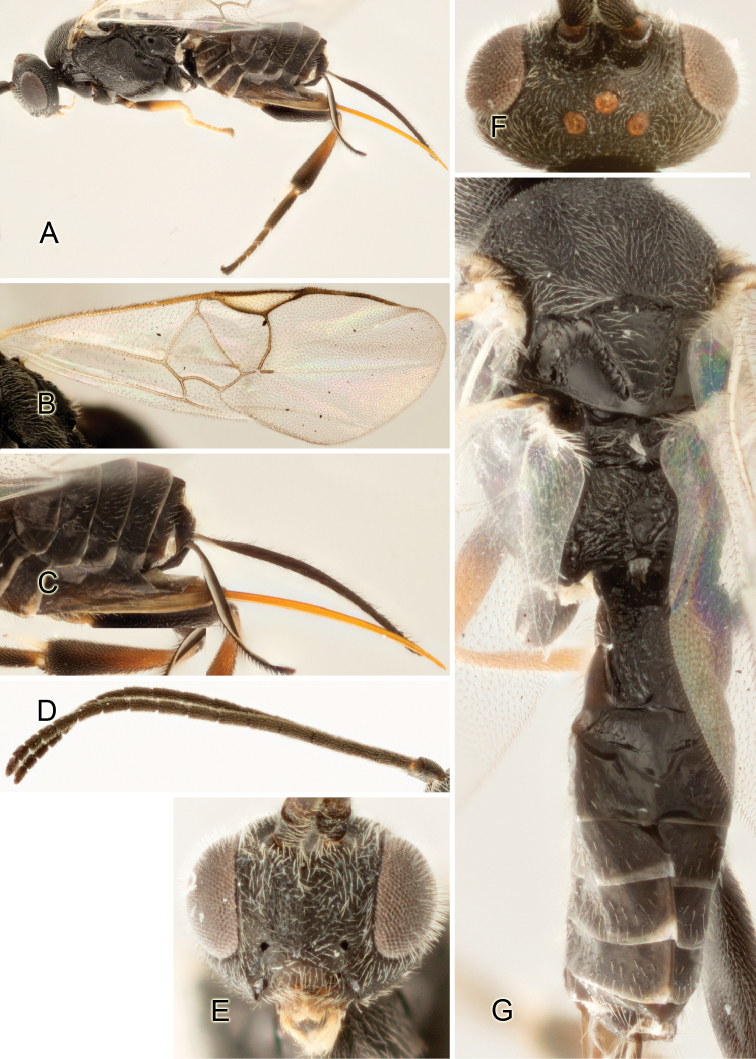
*Apanteles zeneidabolanosae*. **A** Habitus, lateral view **B** Fore wing **C** Hypopygium and ovipositor sheats **D** Antenna **E** Head, frontal view **F** Head, dorsal view **G** Meso- and metasoma, dorsal view.

**Figure 23.**
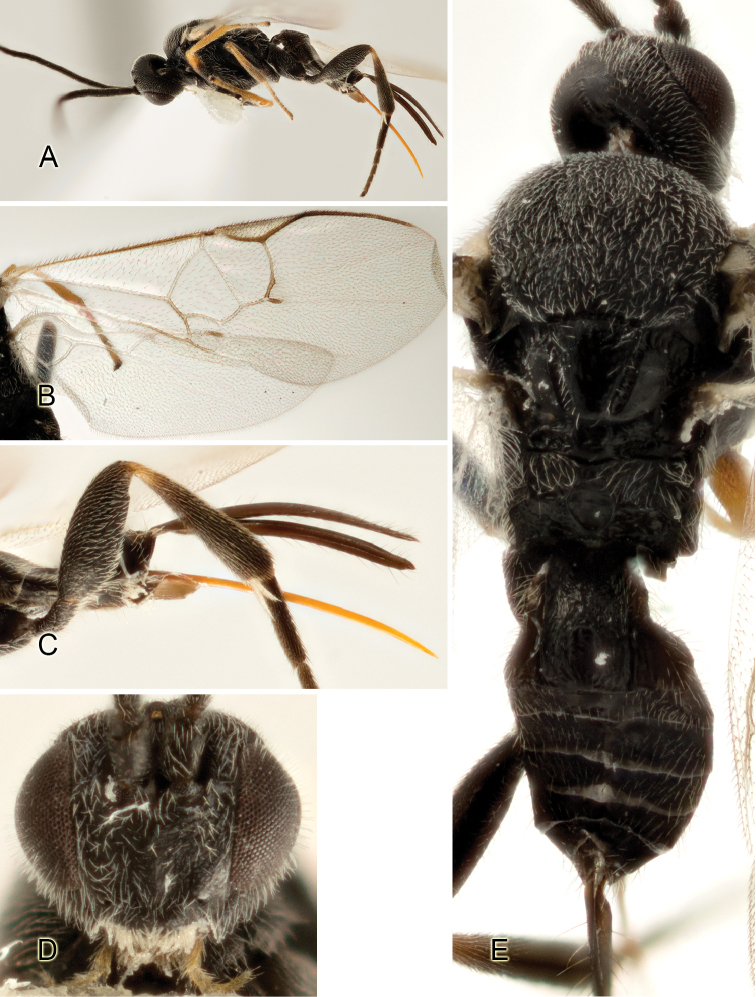
*Apanteles adrianachavarriae*. **A** Habitus, lateral view **B** Fore wing **C** Hypopygium and ovipositor sheats **D** Head, frontal view **E** Head, meso- and metasoma, dorsal view.

**Figure 24.**
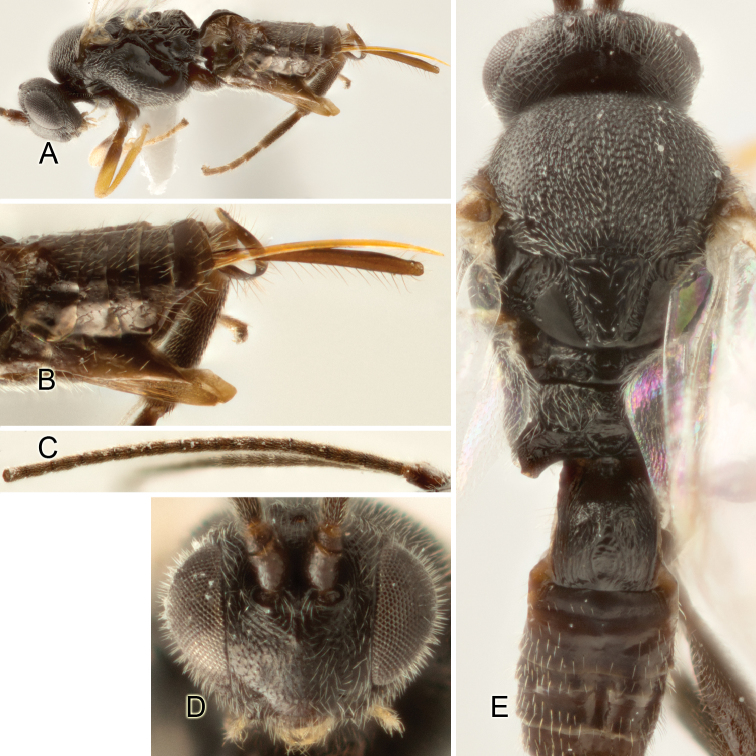
*Apanteles adrianguadamuzi*. **A** Habitus, lateral view **B** Hypopygium and ovipositor sheats **C** Antenna **D** Head, frontal view **E** Head, meso- and metasoma, dorsal view.

**Figure 25.**
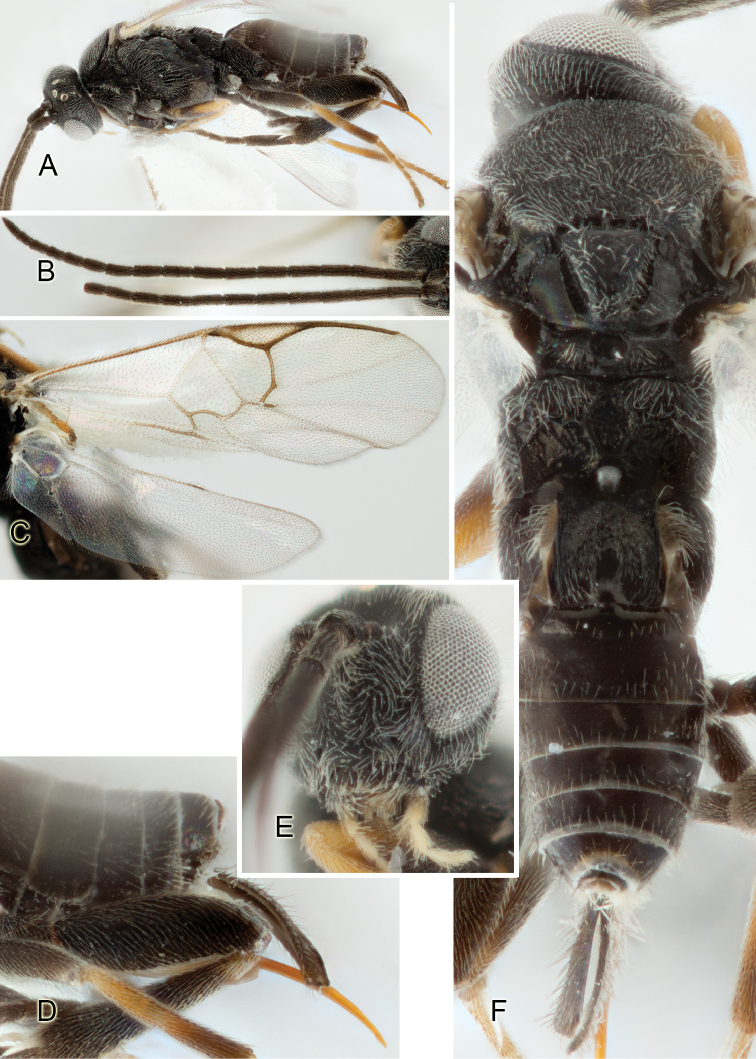
*Apanteles anamartinesae*. **A** Habitus, lateral view **B** Antenna **C** Fore wing **D** Hypopygium and ovipositor sheats **E** Head, frontal view **F** Head, meso- and metasoma, dorsal view.

**Figure 26.**
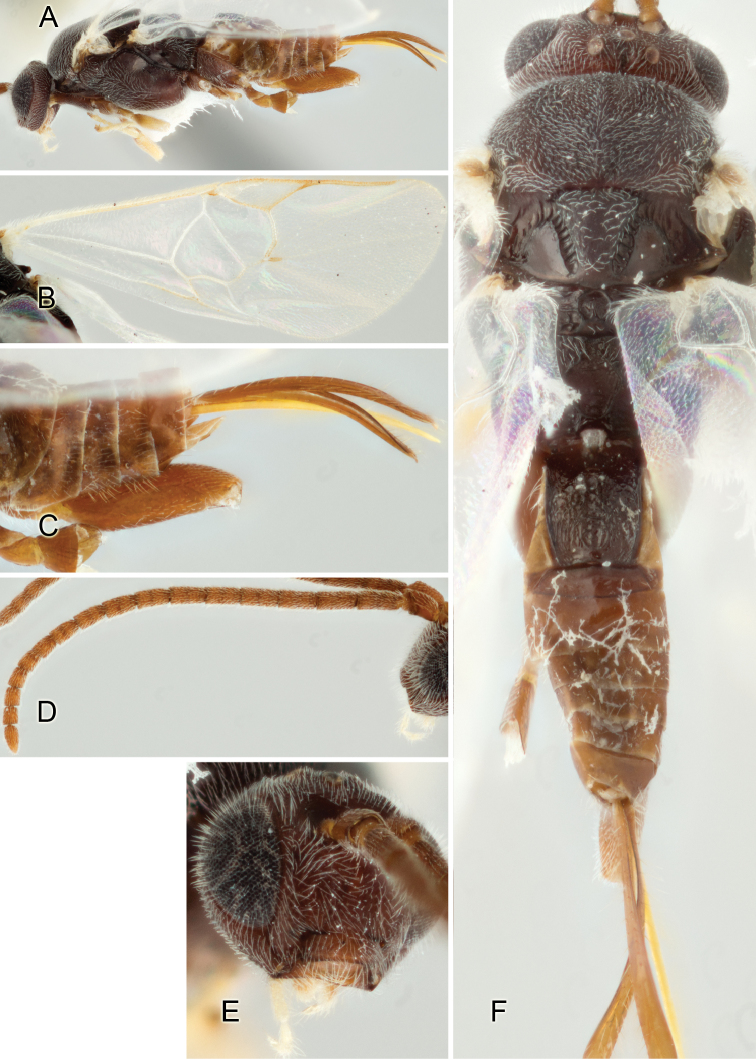
*Apanteles felipechavarriai*. **A** Habitus, lateral view **B** Fore wing **C** Hypopygium and ovipositor sheats **D** Antenna **E** Head, frontal view **F** Head, meso- and metasoma, dorsal view.

**Figure 27.**
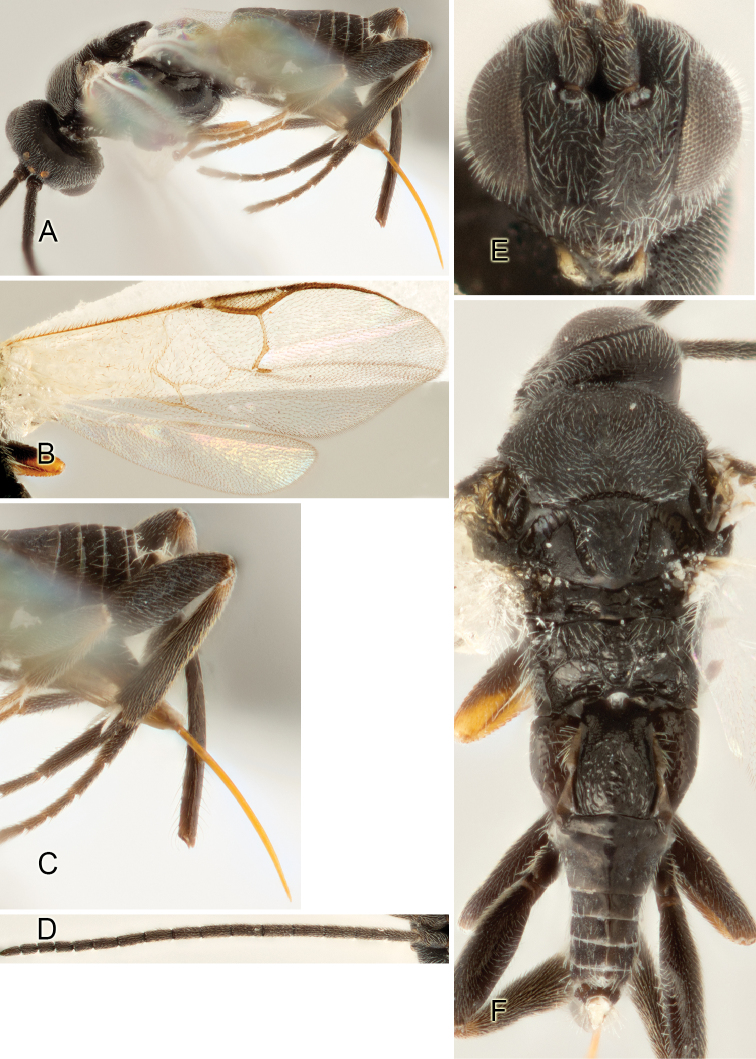
*Apanteles irenecarrilloi*. **A** Habitus, lateral view **B** Fore wing **C** Hypopygium and ovipositor sheats **D** Antenna **E** Head, frontal view **F** Head, meso- and metasoma, dorsal view.

**Figure 28.**
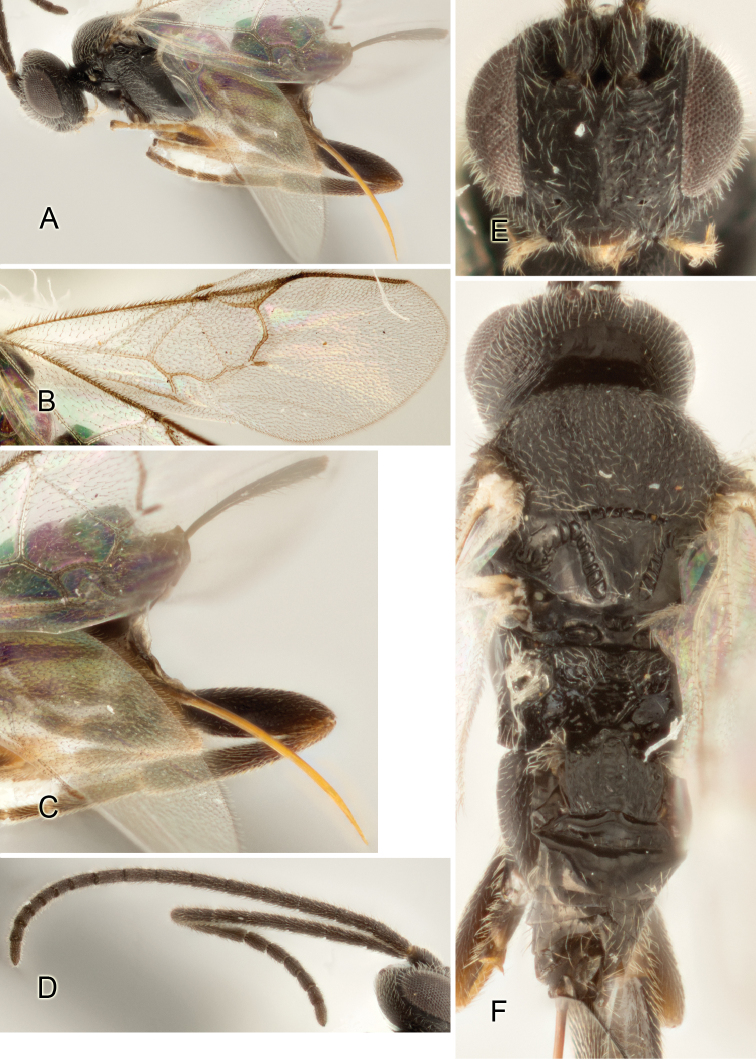
*Apanteles luiscantillanoi*. **A** Habitus, lateral view **B** Fore wing **C** Hypopygium and ovipositor sheats **D** Antenna **E** Head, frontal view **F** Head, meso- and metasoma, dorsal view.

**Figure 29.**
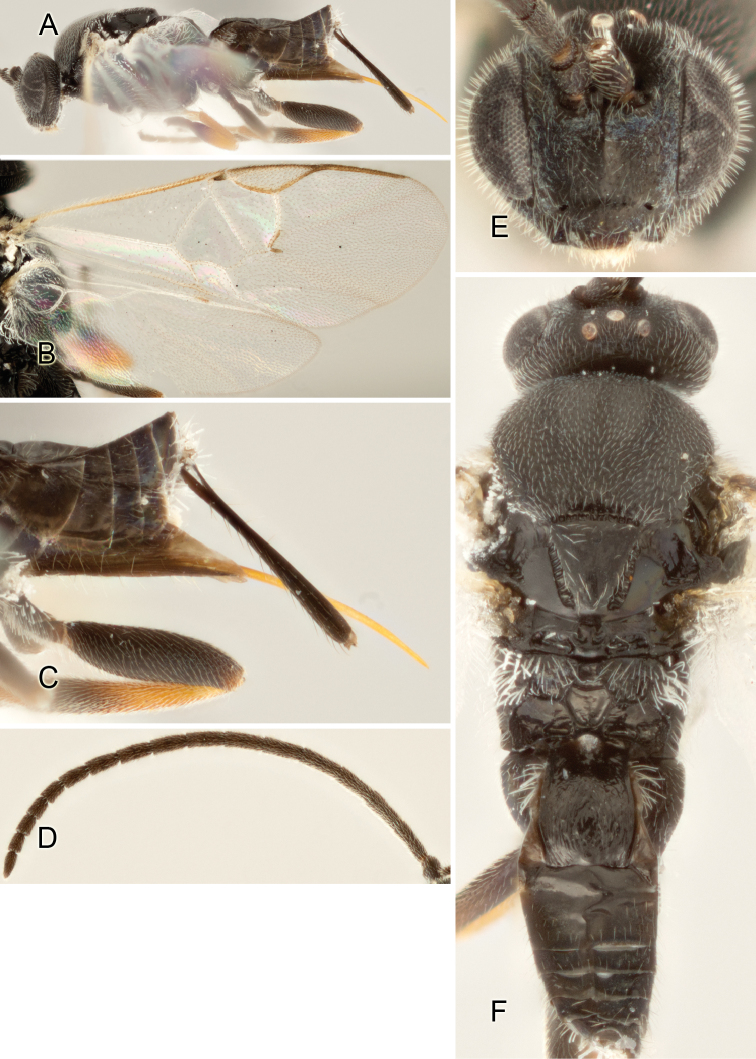
*Apanteles mariatorrentesae*. **A** Habitus, lateral view **B** Fore wing **C** Hypopygium and ovipositor sheats **D** Antenna **E** Head, frontal view **F** Head, meso- and metasoma, dorsal view.

**Figure 30.**
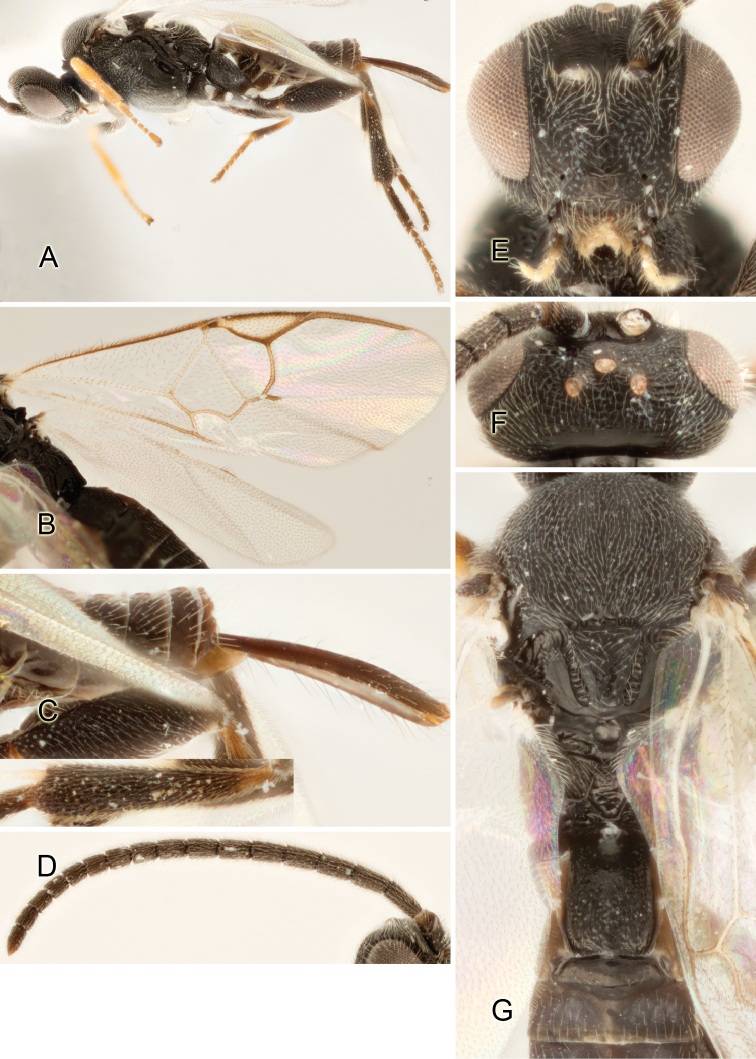
*Apanteles ronaldquirosi*. **A** Habitus, lateral view **B** Fore wing **C** Hypopygium and ovipositor sheats **D** Antenna **E** Head, frontal view **F** Head, dorsal view **G** Meso- and metasoma, dorsal view.

**Figure 31.**
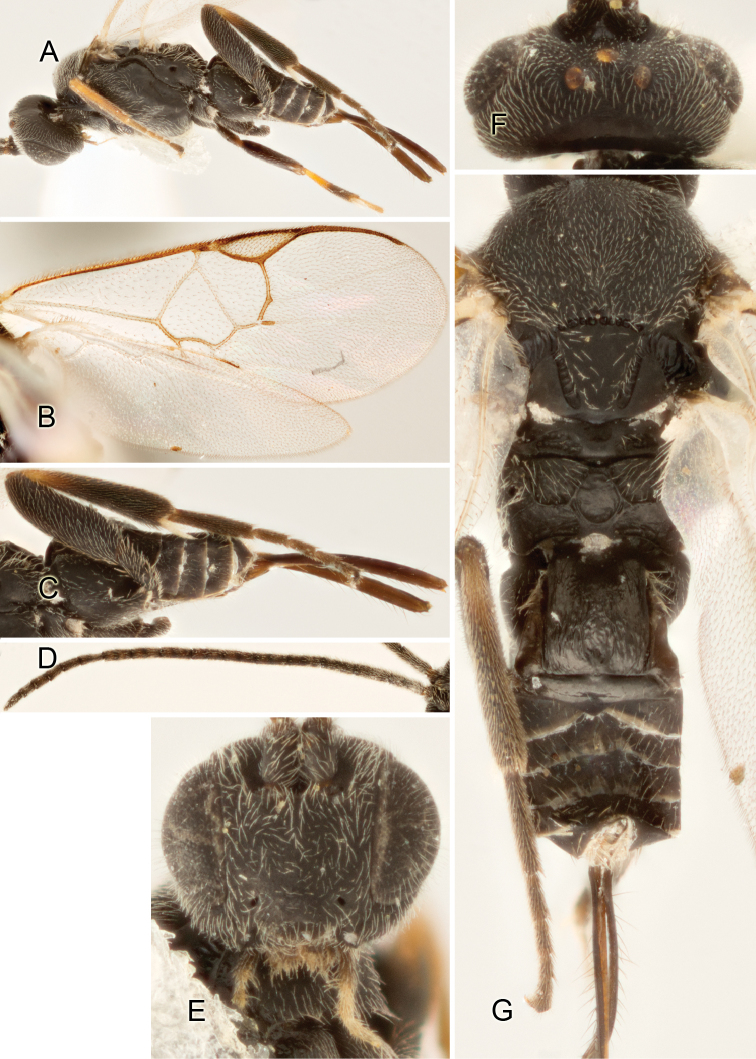
*Apanteles yilbertalvaradoi*. **A** Habitus, lateral view **B** Fore wing **C** Hypopygium and ovipositor sheats **D** Antenna **E** Head, frontal view **F** Head, dorsal view **G** Meso- and metasoma, dorsal view.

**Figure 32.**
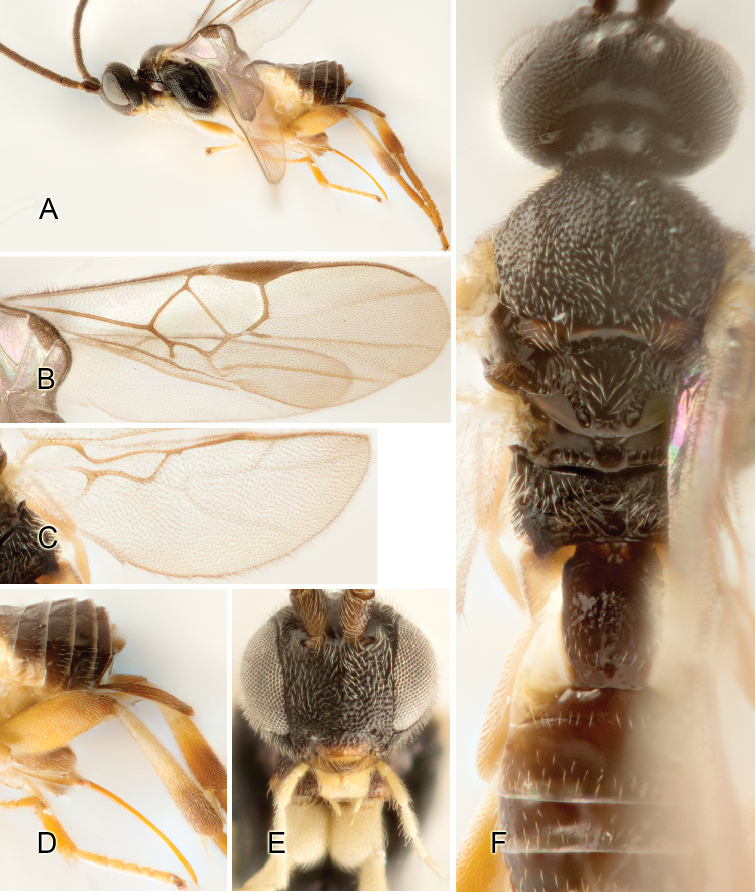
*Apanteles adrianaguilarae*. **A** Habitus, lateral view **B** Fore wing **C** Hind wing **D** Hypopygium and ovipositor sheats **E** Head, frontal view **F** Head, meso- and metasoma (partially), dorsal view.

**Figure 33.**
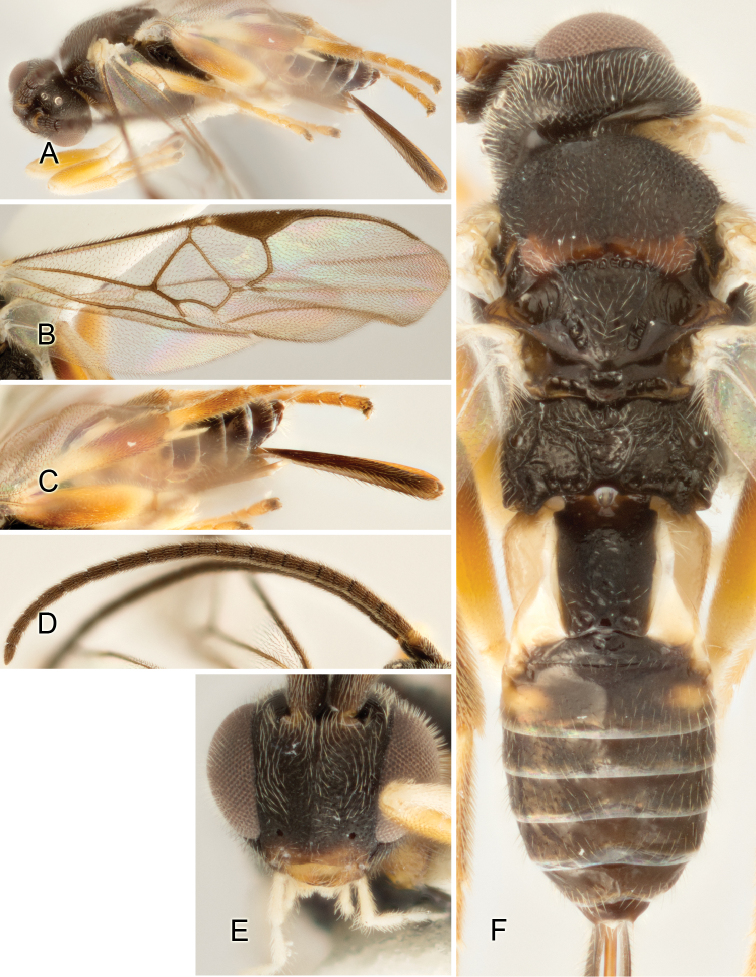
*Apanteles ivonnetranae*. **A** Habitus, lateral view **B** Fore wing **C** Hypopygium and ovipositor sheats **D** Antenna **E** Head, frontal view **F** Head, meso- and metasoma, dorsal view.

**Figure 34.**
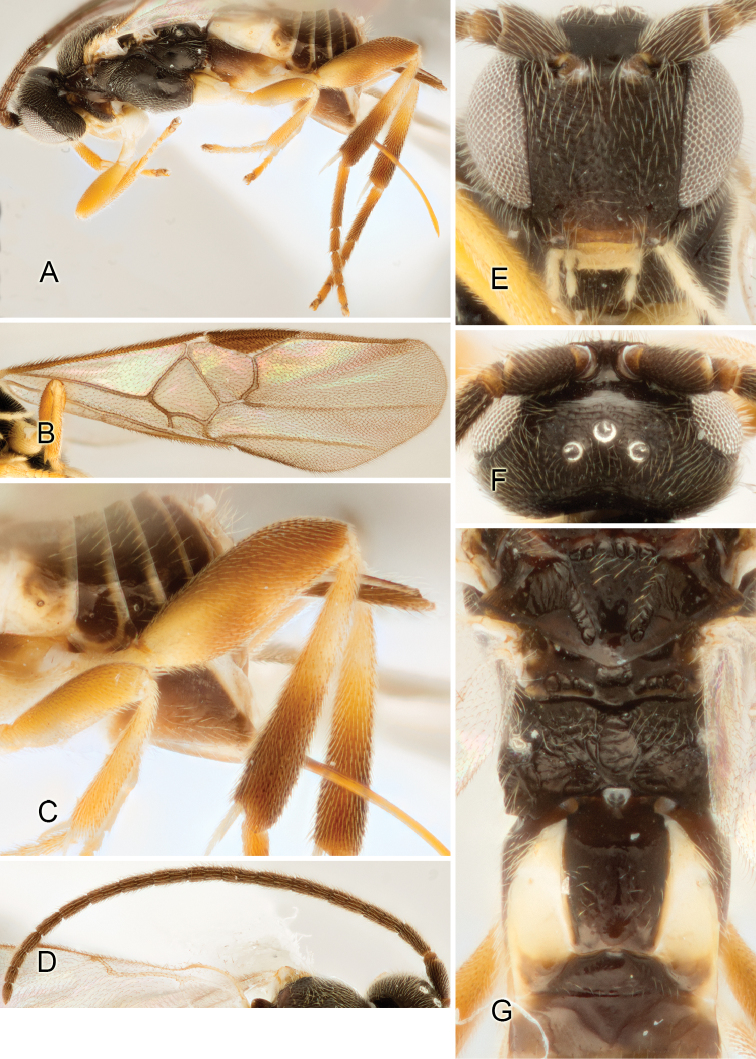
*Apanteles vannesabrenesae*. **A** Habitus, lateral view **B** Fore wing **C** Hypopygium and ovipositor sheats **D** Antenna **E** Head, frontal view **F** Head, dorsal view **G** Meso- and metasoma (partially), dorsal view.

**Figure 35.**
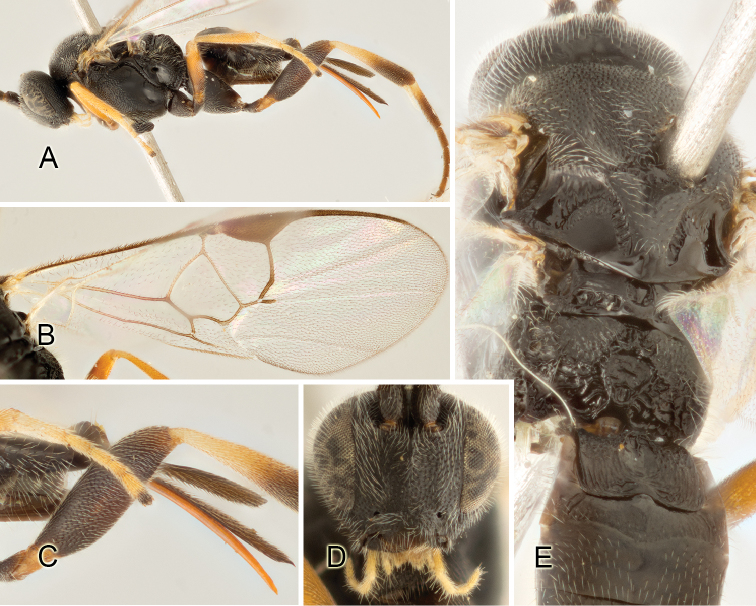
*Apanteles aichagirardae*. **A** Habitus, lateral view **B** Fore wing **C** Hypopygium and ovipositor sheats **D** Head, frontal view **E** Head, meso- and metasoma (partially), dorsal view.

**Figure 36.**
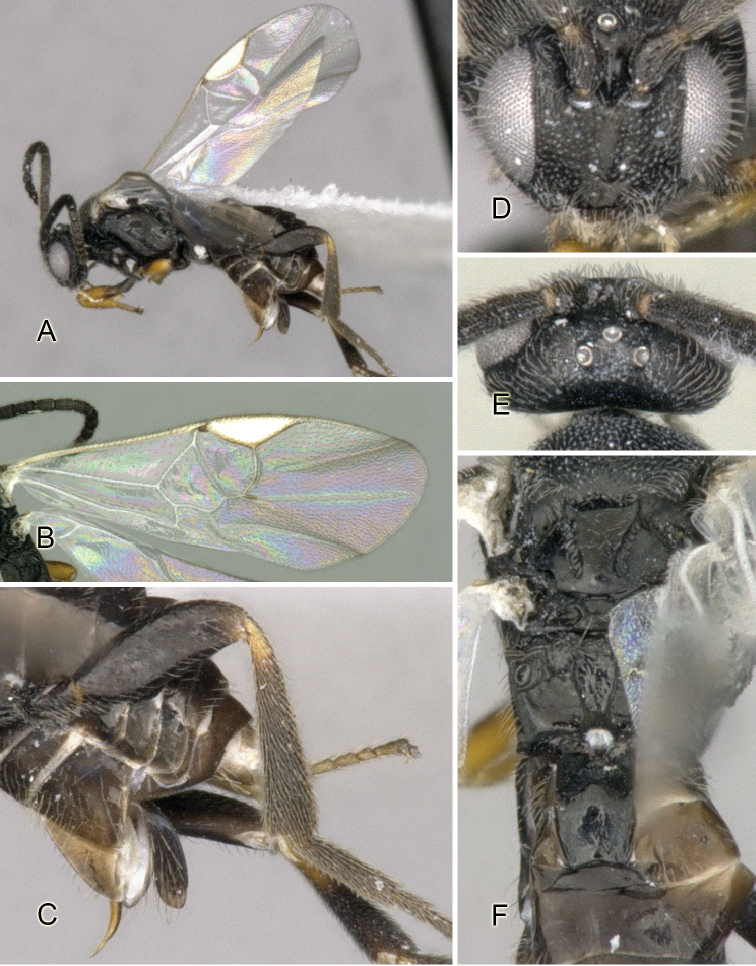
*Apanteles aidalopezae*. **A** Habitus, lateral view **B** Fore wing **C** Hypopygium and ovipositor sheats **D** Head, frontal view **E** Head, dorsal view **F** Meso- and metasoma (partially), dorsal view.

**Figure 37.**
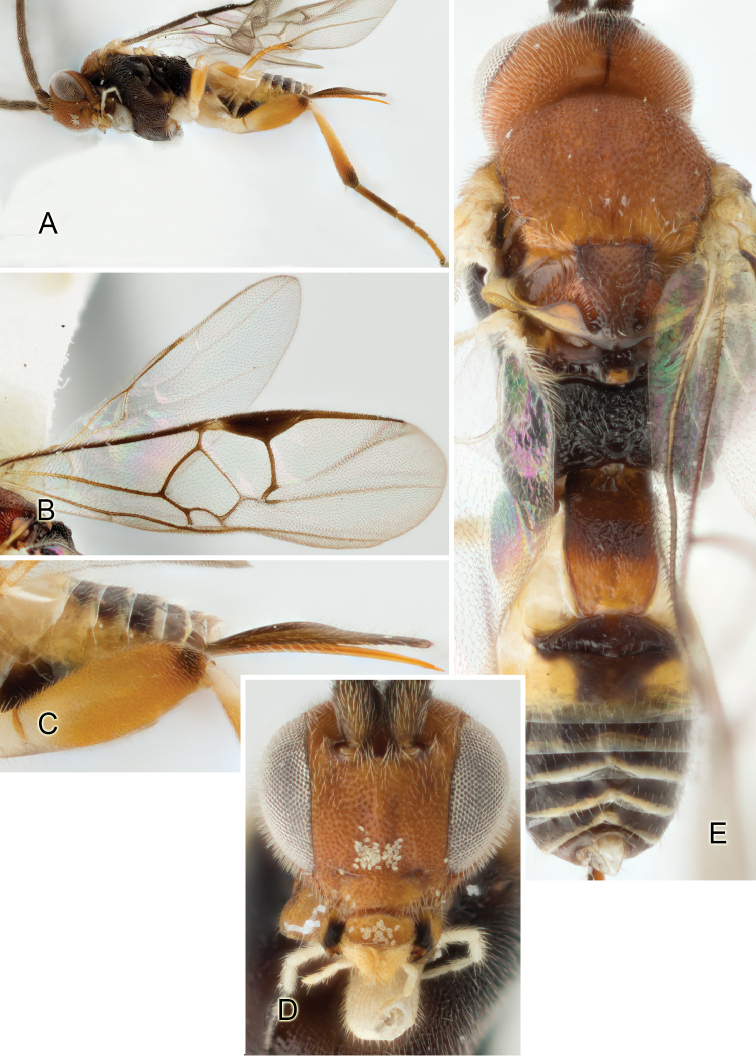
*Apanteles alejandromasisi*. **A** Habitus, lateral view **B** Fore wing **C** Hypopygium and ovipositor sheats **D** Head, frontal view **E** Head, meso- and metasoma, dorsal view.

**Figure 38.**
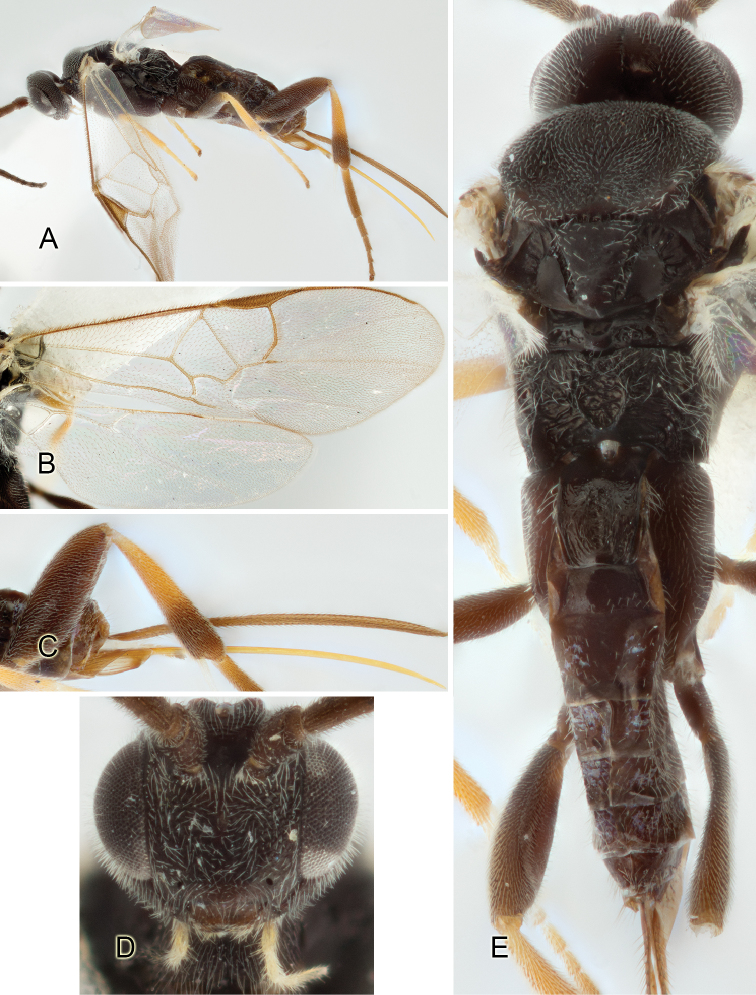
*Apanteles alejandromorai*. **A** Habitus, lateral view **B** Fore wing **C** Hypopygium and ovipositor sheats **D** Head, frontal view **E** Head, meso- and metasoma, dorsal view.

**Figure 39.**
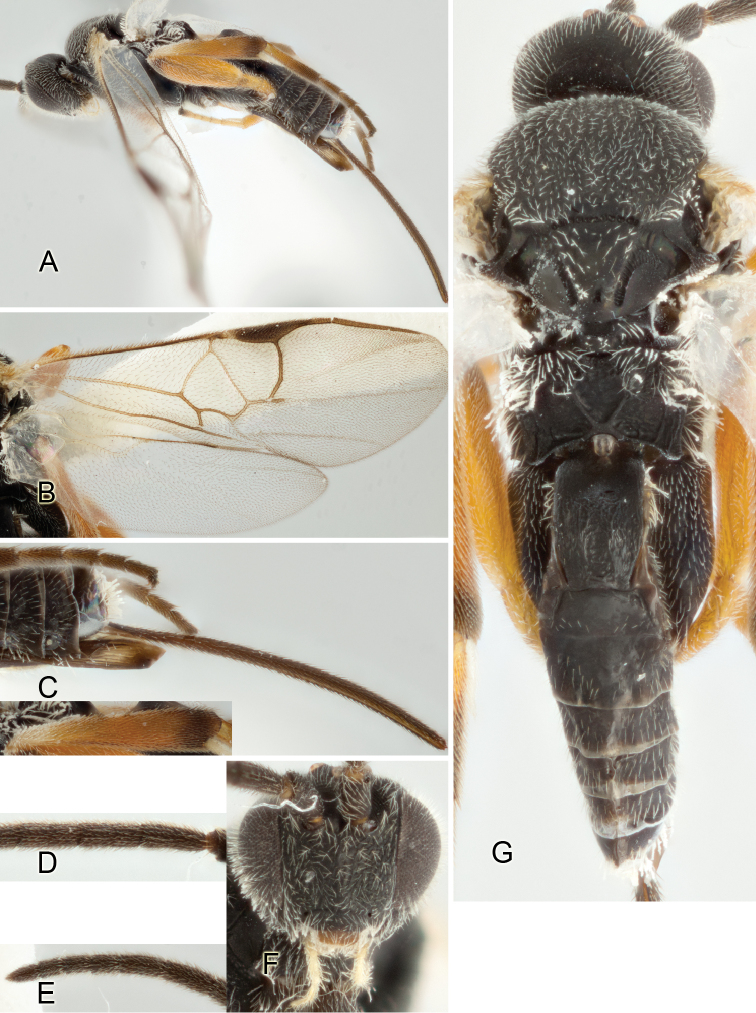
*Apanteles deifiliadavilae*. **A** Habitus, lateral view **B** Fore wing **C** Hypopygium and ovipositor sheats, with details of metatibia **D** Anterior half of antenna **E** Posterior half of antenna **F** Head, frontal view **G** Head, meso- and metasoma, dorsal view.

**Figure 40.**
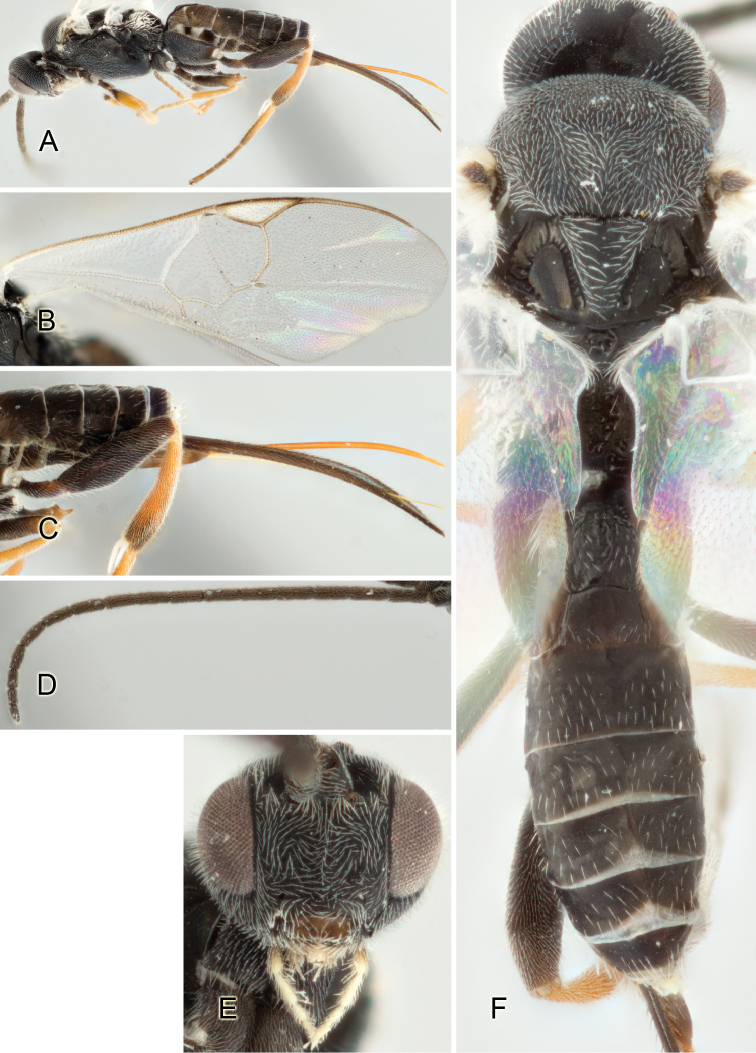
*Apanteles eulogiosequeirai*. **A** Habitus, lateral view **B** Fore wing **C** Hypopygium and ovipositor sheats **D** Antenna **E** Head, frontal view **F** Head, meso- and metasoma, dorsal view.

**Figure 41.**
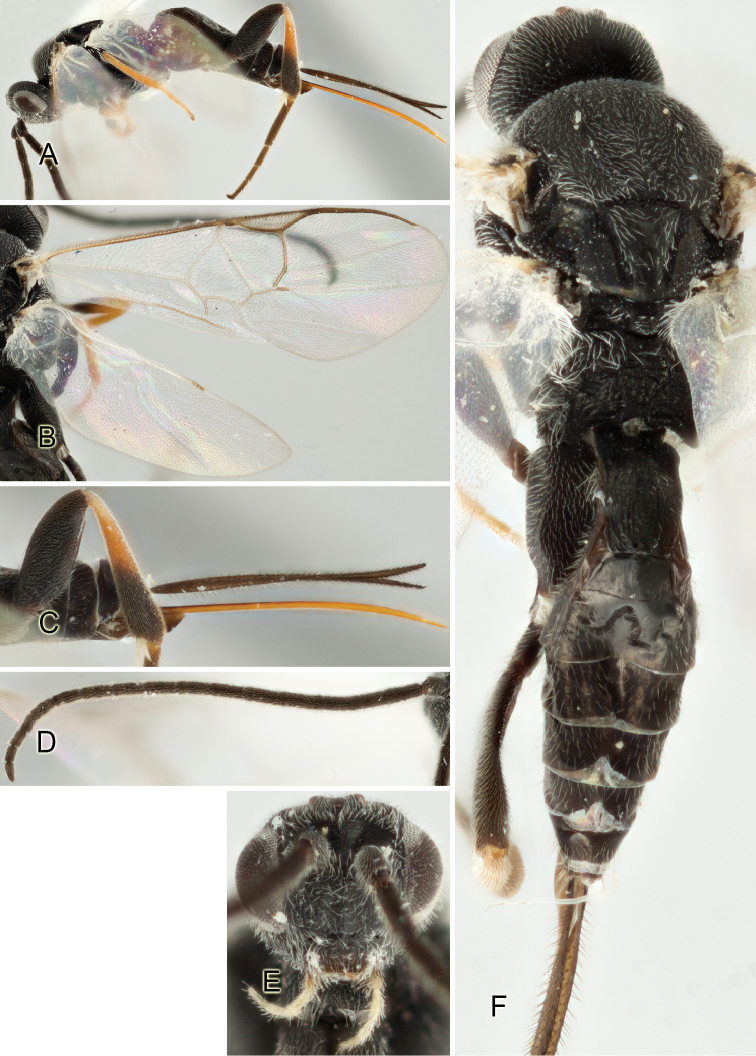
*Apanteles fernandochavarriai*. **A** Habitus, lateral view **B** Fore wing **C** Hypopygium and ovipositor sheats **D** Antenna **E** Head, frontal view **F** Head, dorsal view **G** Head, meso- and metasoma, dorsal view.

**Figure 42.**
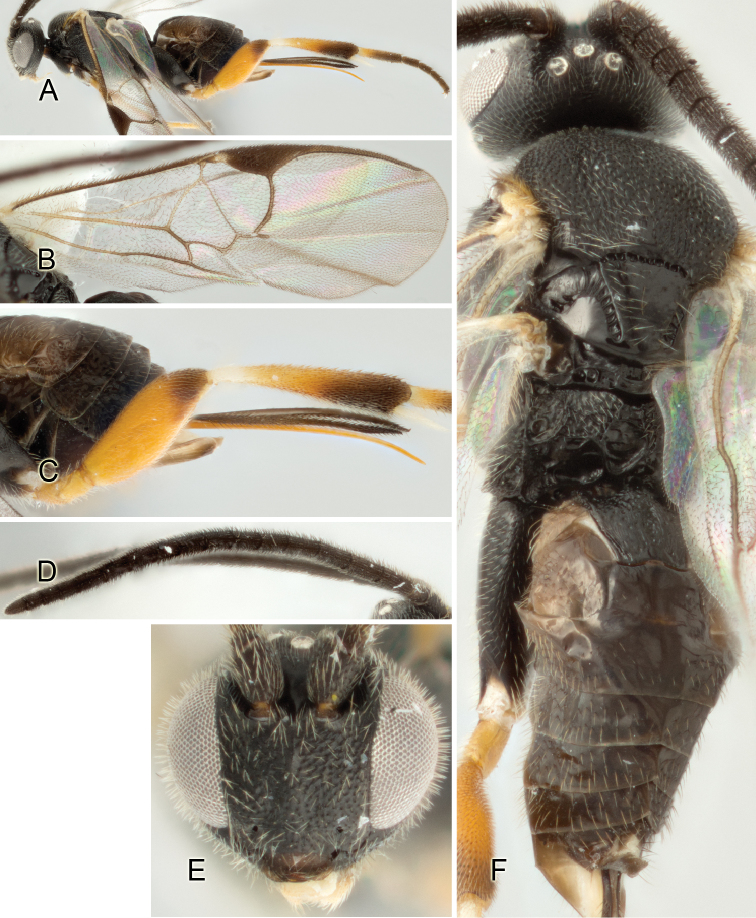
*Apanteles franciscoramirezi*. **A** Habitus, lateral view **B** Fore wing **C** Hypopygium and ovipositor sheats **D** Antenna **E** Head, frontal view **F** Head, meso- and metasoma, dorsal view.

**Figure 43.**
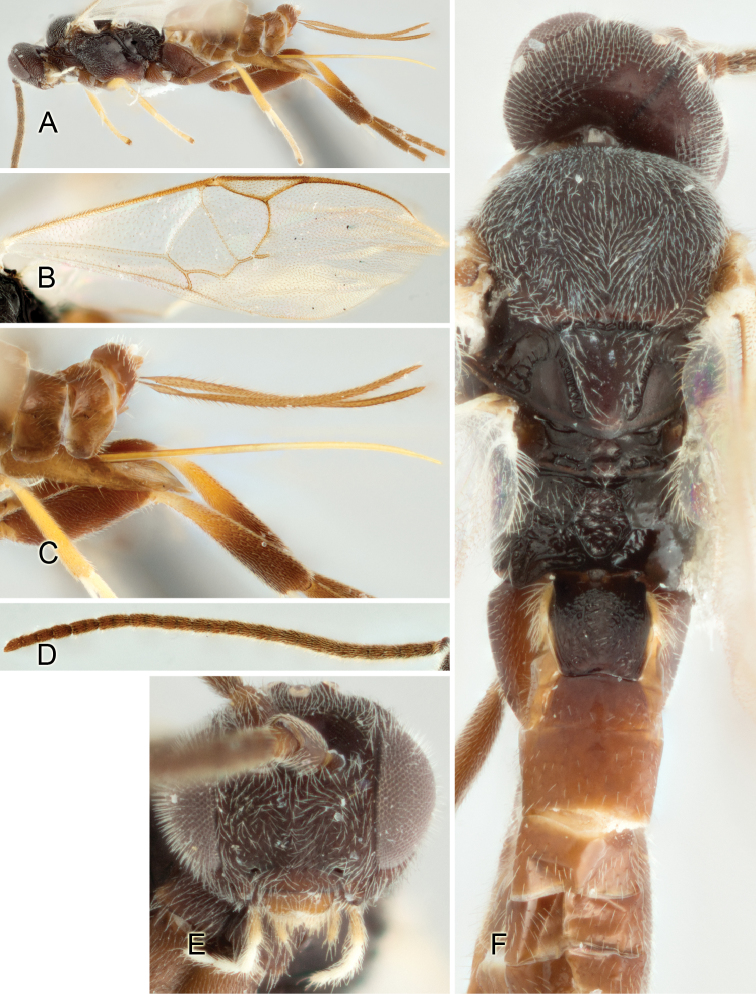
*Apanteles freddysalazari*. **A** Habitus, lateral view **B** Fore wing **C** Hypopygium and ovipositor sheats **D** Antenna **E** Head, frontal view **F** Head, meso- and metasoma (dorsally), dorsal view.

**Figure 44.**
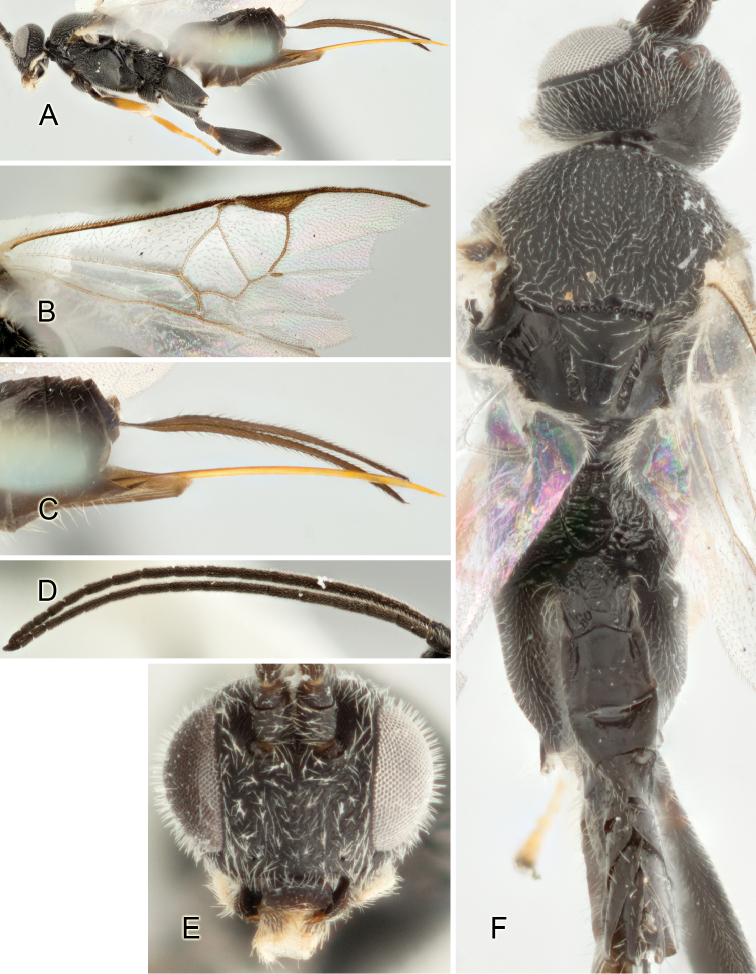
*Apanteles gabrielagutierrezae*. **A** Habitus, lateral view **B** Fore wing **C** Hypopygium and ovipositor sheats **D** Antenna **E** Head, frontal view **F** Head, meso- and metasoma, dorsal view.

**Figure 45.**
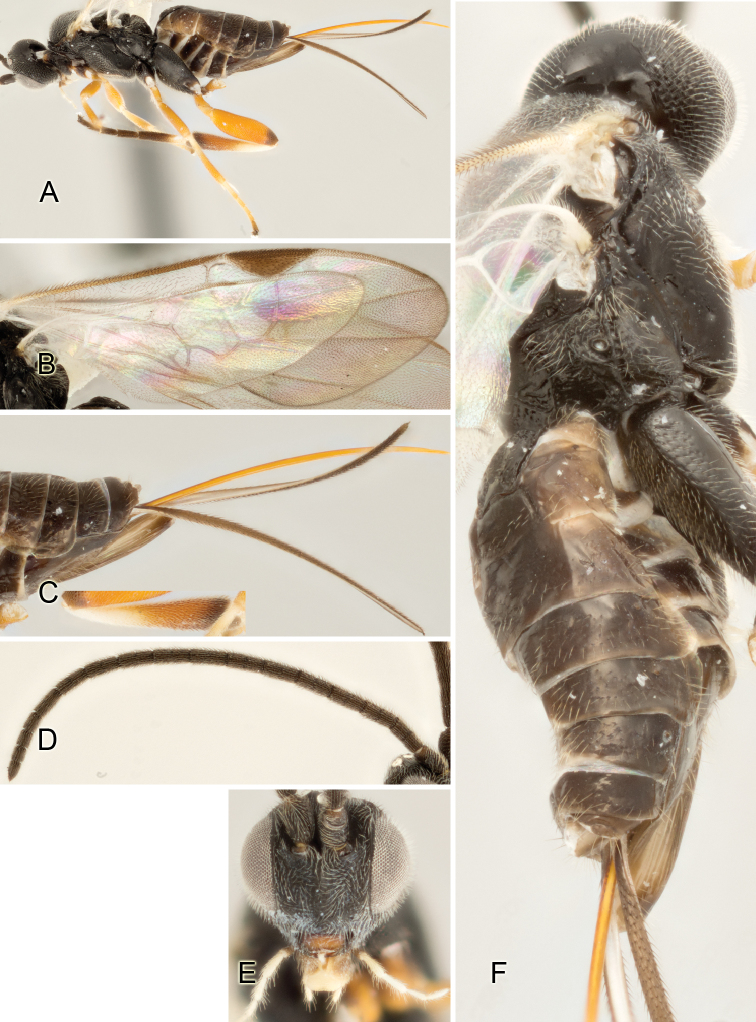
*Apanteles juancarrilloi*. **A** Habitus, lateral view **B** Fore wing **C** Hypopygium and ovipositor sheats **D** Antenna **E** Head, frontal view **F** Head, meso- and metasoma, dorsal view.

**Figure 46.**
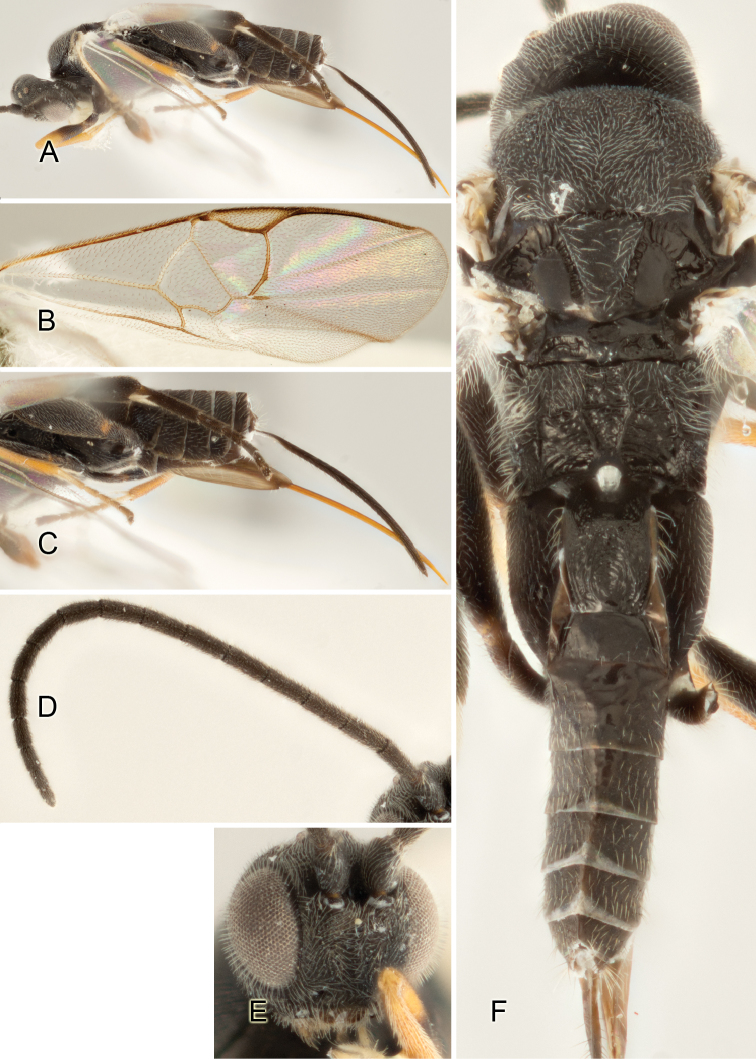
*Apanteles luisbrizuelai*. **A** Habitus, lateral view **B** Fore wing **C** Hypopygium and ovipositor sheats **D** Antenna **E** Head, frontal view **F** Head, meso- and metasoma, dorsal view.

**Figure 47.**
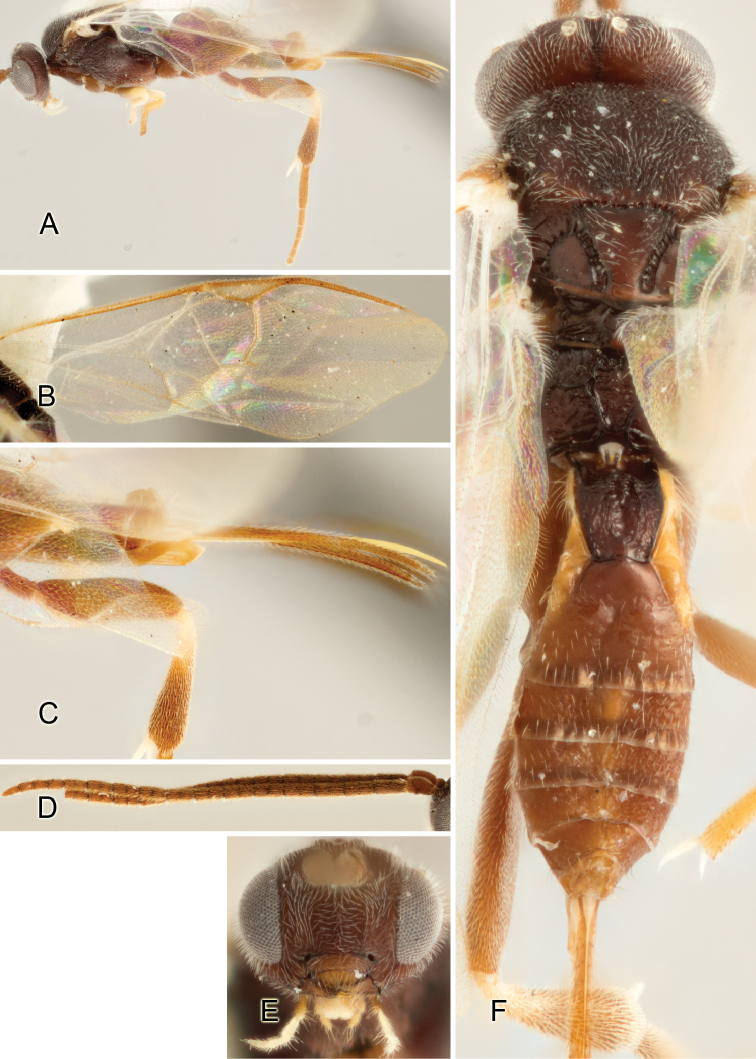
*Apanteles luisgarciai*. **A** Habitus, lateral view **B** Fore wing **C** Hypopygium and ovipositor sheats **D** Antenna **E** Head, frontal view **F** Head, meso- and metasoma, dorsal view.

**Figure 48.**
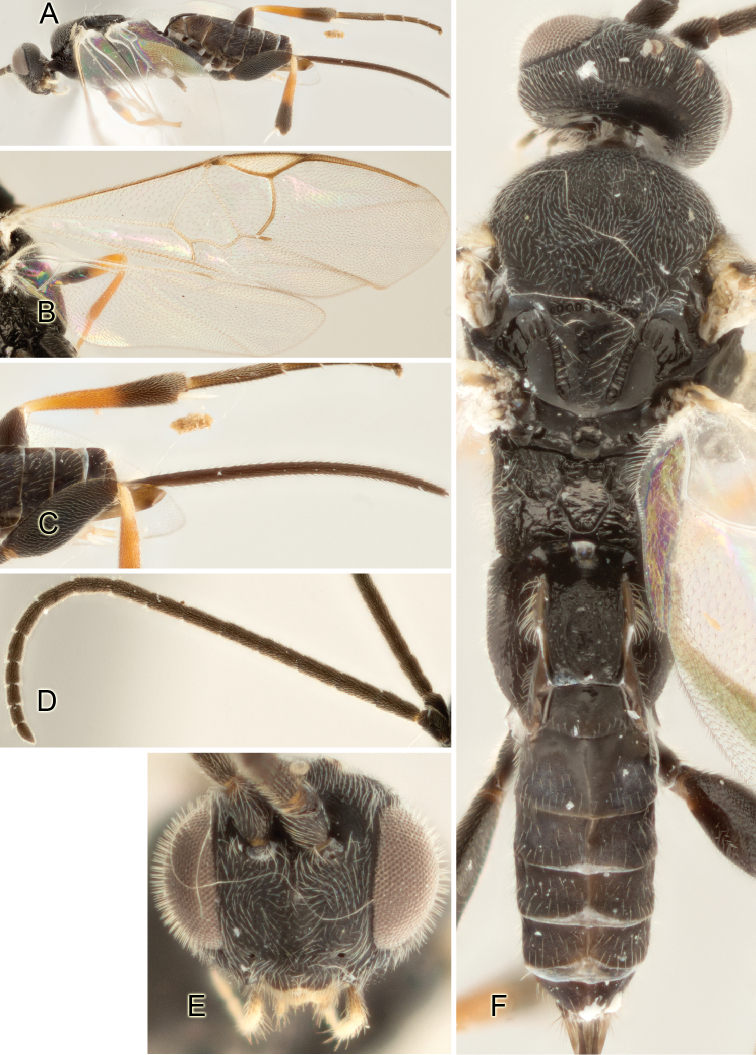
*Apanteles marvinmendozai*. **A** Habitus, lateral view **B** Fore wing **C** Hypopygium and ovipositor sheats **D** Antenna **E** Head, frontal view **F** Head, meso- and metasoma, dorsal view.

**Figure 49.**
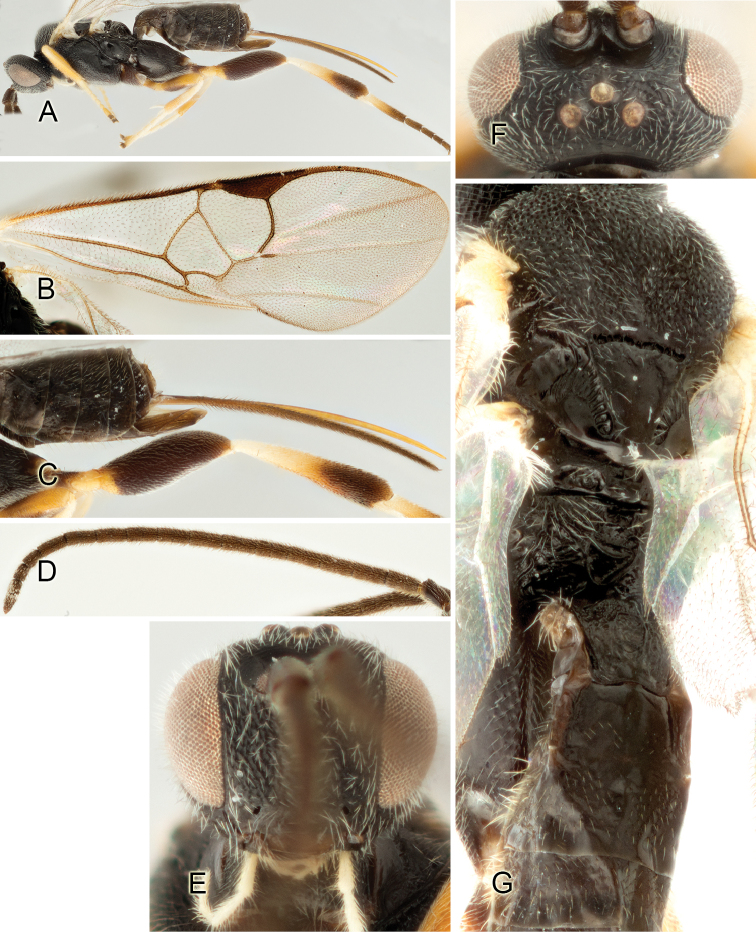
*Apanteles tiboshartae*. **A** Habitus, lateral view **B** Fore wing **C** Hypopygium and ovipositor sheats **D** Antenna **E** Head, frontal view **F** Head, dorsal view **G** Meso- and metasoma (partially), dorsal view.

**Figure 50.**
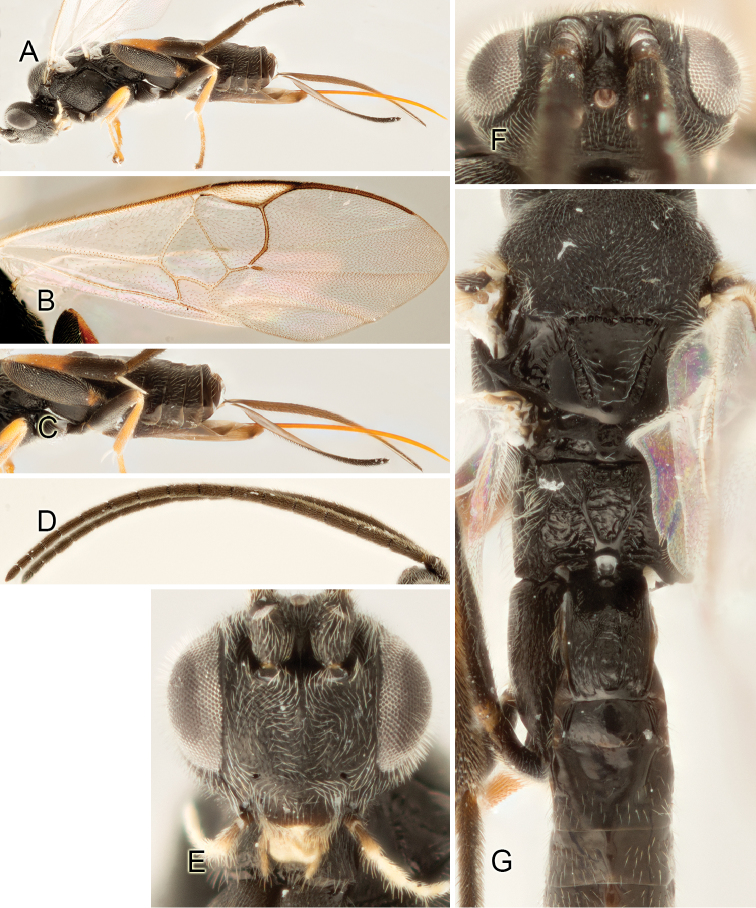
*Apanteles minornavarroi*. **A** Habitus, lateral view **B** Fore wing **C** Hypopygium and ovipositor sheats **D** Antenna **E** Head, frontal view **F** Head, dorsal view **G** Meso- and metasoma (partially), dorsal view.

**Figure 51.**
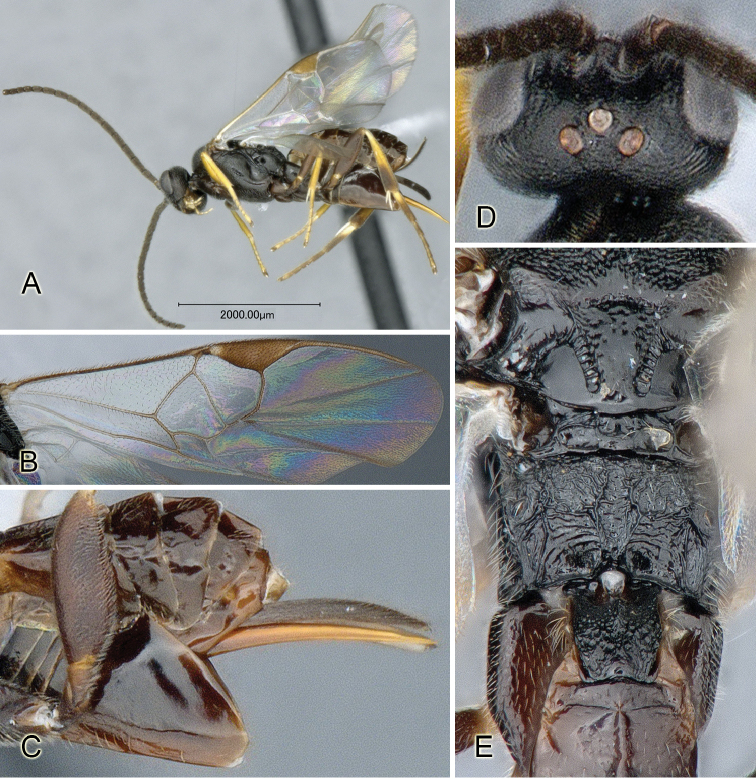
*Apanteles anabellecordobae*. **A** Habitus, lateral view **B** Fore wing **C** Hypopygium and ovipositor sheats **D** Head, frontal view **E** Meso- and metasoma (partially), dorsal view.

**Figure 52.**
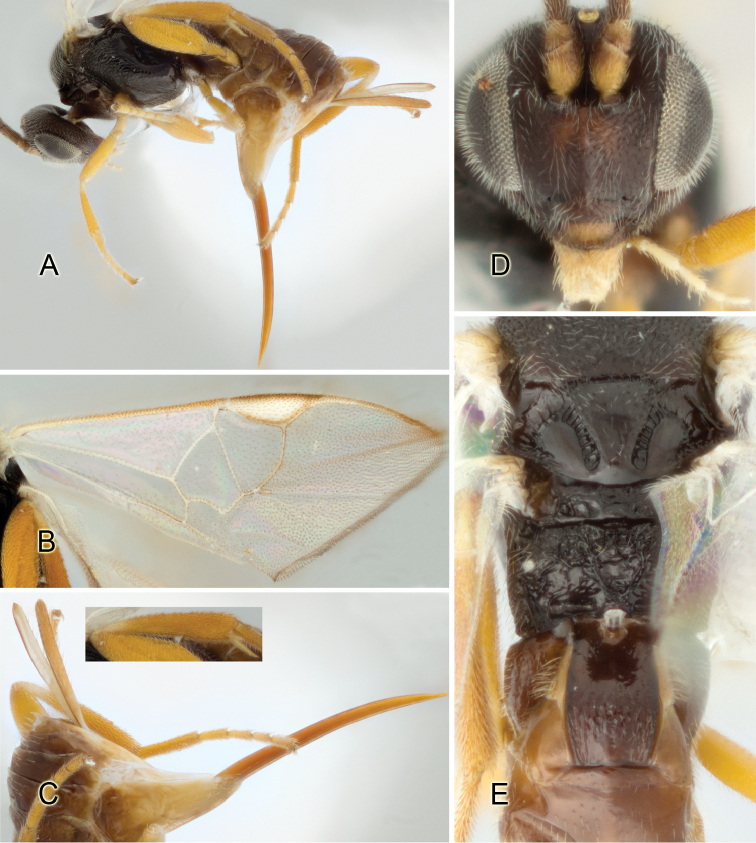
*Apanteles carolinacanoae*. **A** Habitus, lateral view **B** Fore wing **C** Hypopygium and ovipositor sheats, with details of metatibia **D** Head, frontal view **E** Meso- and metasoma (partially), dorsal view.

**Figure 53.**
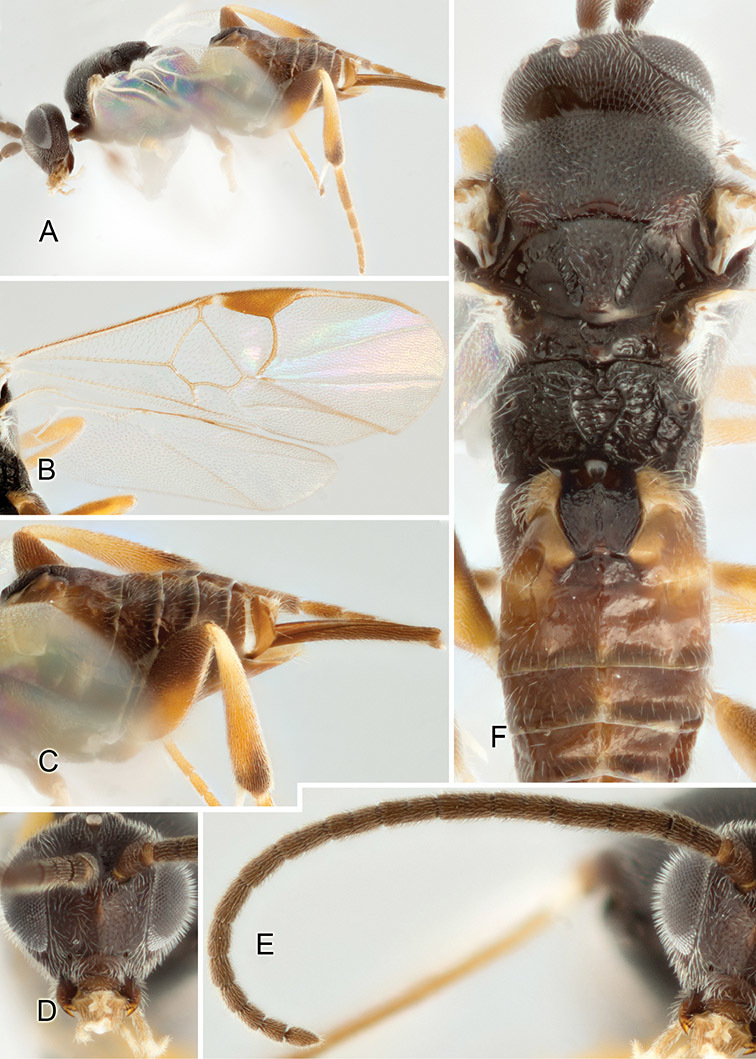
*Apanteles duniagarciae*. **A** Habitus, lateral view **B** Fore wing **C** Hypopygium and ovipositor sheats **D** Head, frontal view **E** Antenna **F** Head, meso- and metasoma (partially), dorsal view.

**Figure 54.**
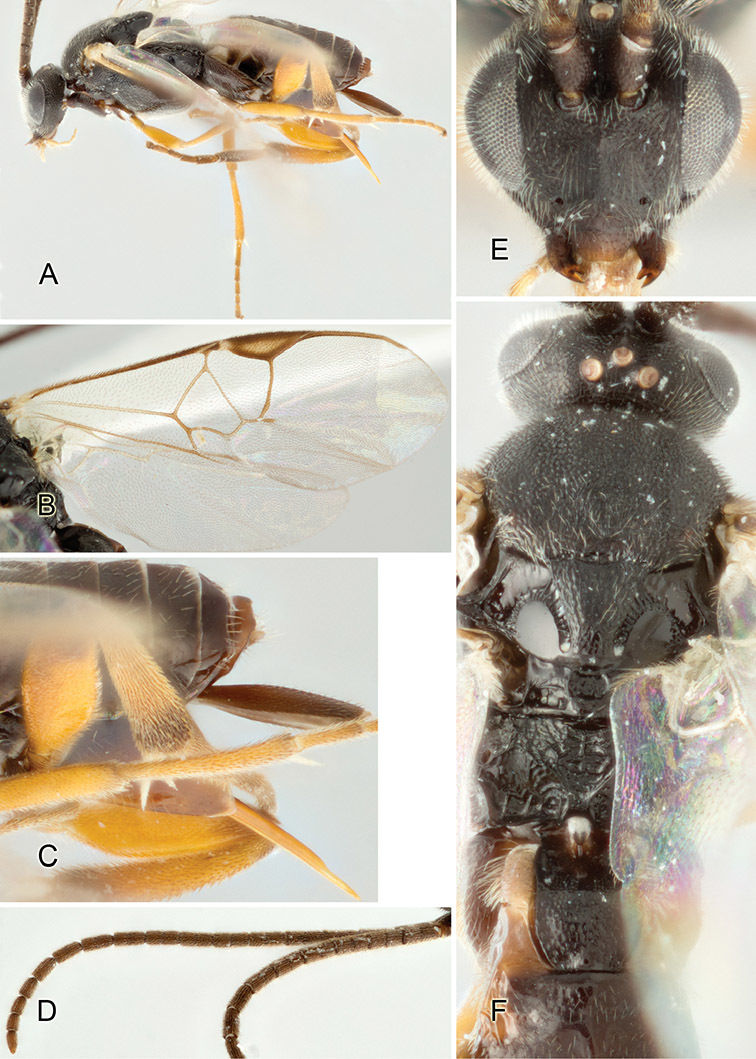
*Apanteles edwinapui*. **A** Habitus, lateral view **B** Fore wing **C** Hypopygium and ovipositor sheats **D** Antenna **E** Head, frontal view **F** Head, meso- and metasoma (partially), dorsal view.

**Figure 55.**
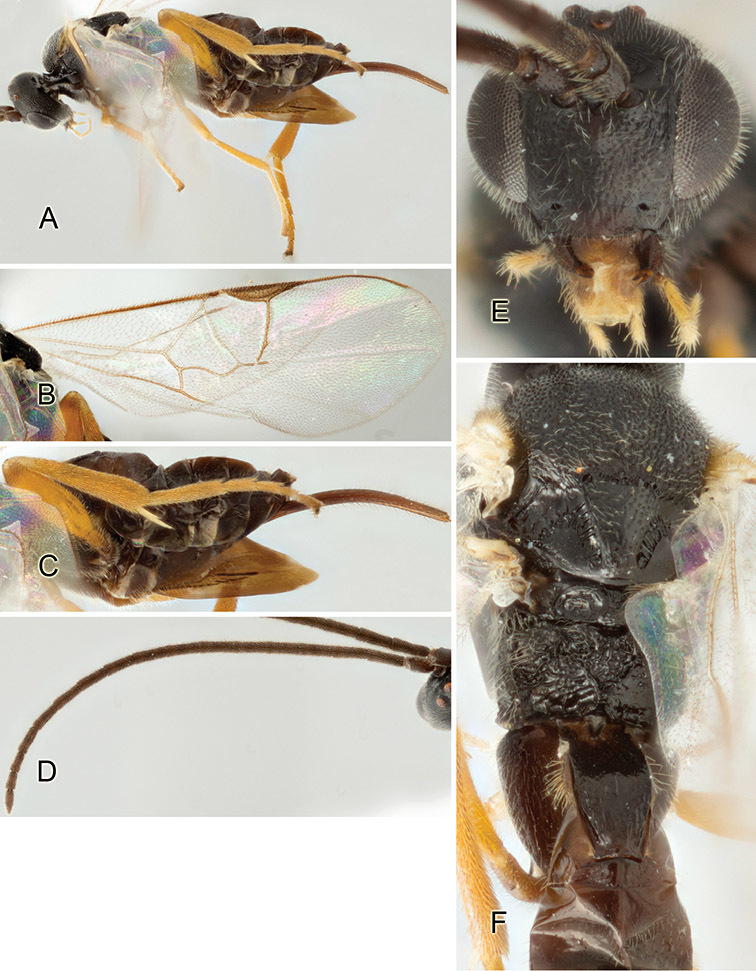
*Apanteles eldarayae*. **A** Habitus, lateral view **B** Fore wing **C** Hypopygium and ovipositor sheats **D** Antenna **E** Head, frontal view **F** Head, meso- and metasoma (partially), dorsal view.

**Figure 56.**
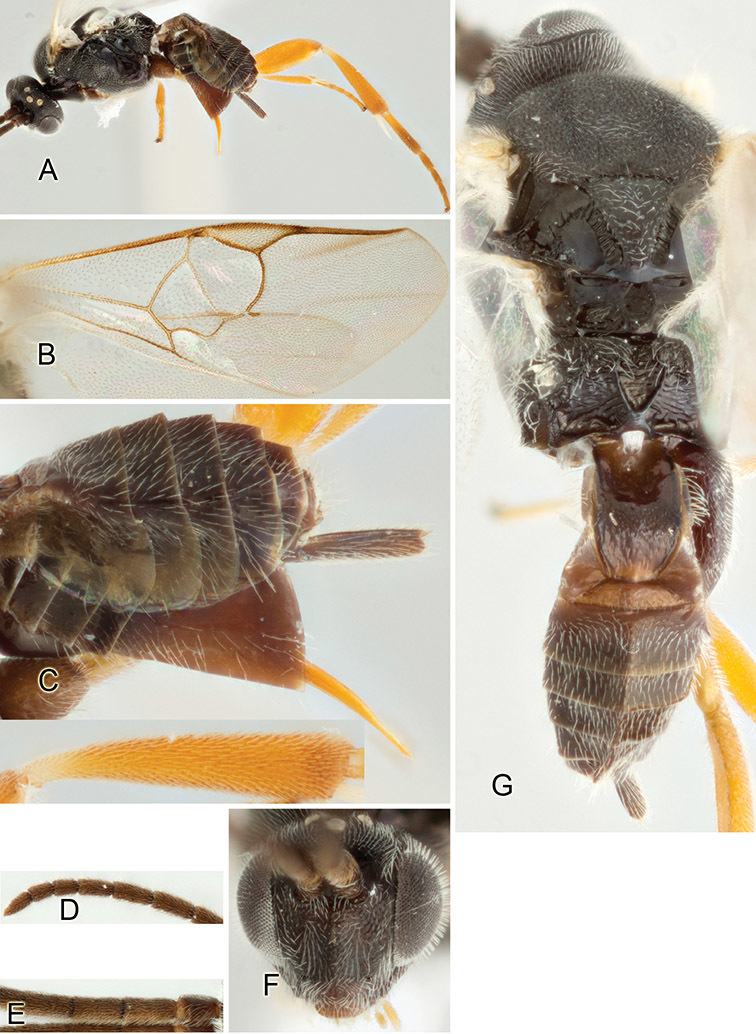
*Apanteles freddyquesadai*. **A** Habitus, lateral view **B** Fore wing **C** Hypopygium and ovipositor sheats, with details of metatibia **D** Anterior half of antenna **E** Posterior half of antenna **F** Head, frontal view **G** Head, meso- and metasoma, dorsal view.

**Figure 57.**
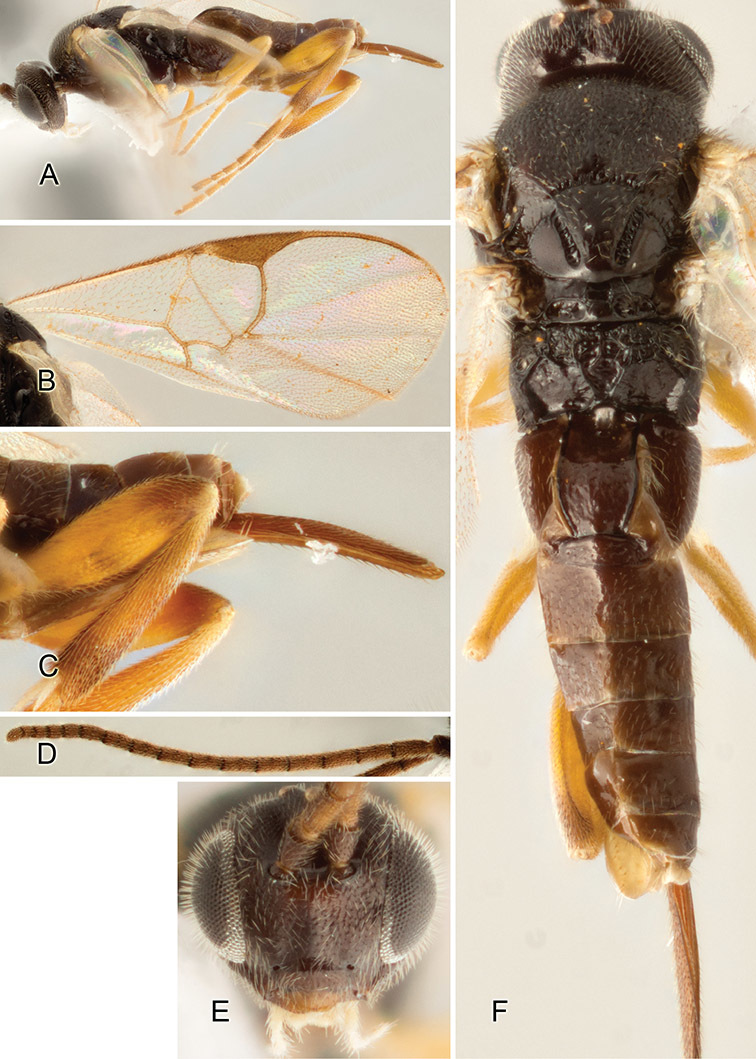
*Apanteles guillermopereirai*. **A** Habitus, lateral view **B** Fore wing **C** Hypopygium and ovipositor sheats **D** Antenna **E** Head, frontal view **F** Head, meso- and metasoma, dorsal view.

**Figure 58.**
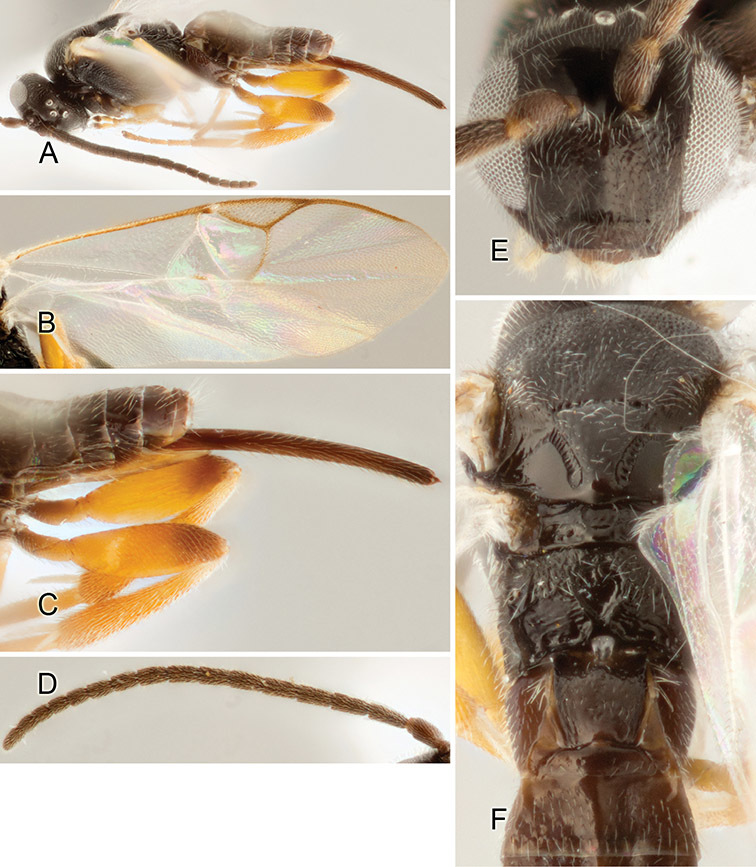
*Apanteles harryramirezi*. **A** Habitus, lateral view **B** Fore wing **C** Hypopygium and ovipositor sheats **D** Antenna **E** Head, frontal view **F** Head, meso- and metasoma (partially), dorsal view.

**Figure 59.**
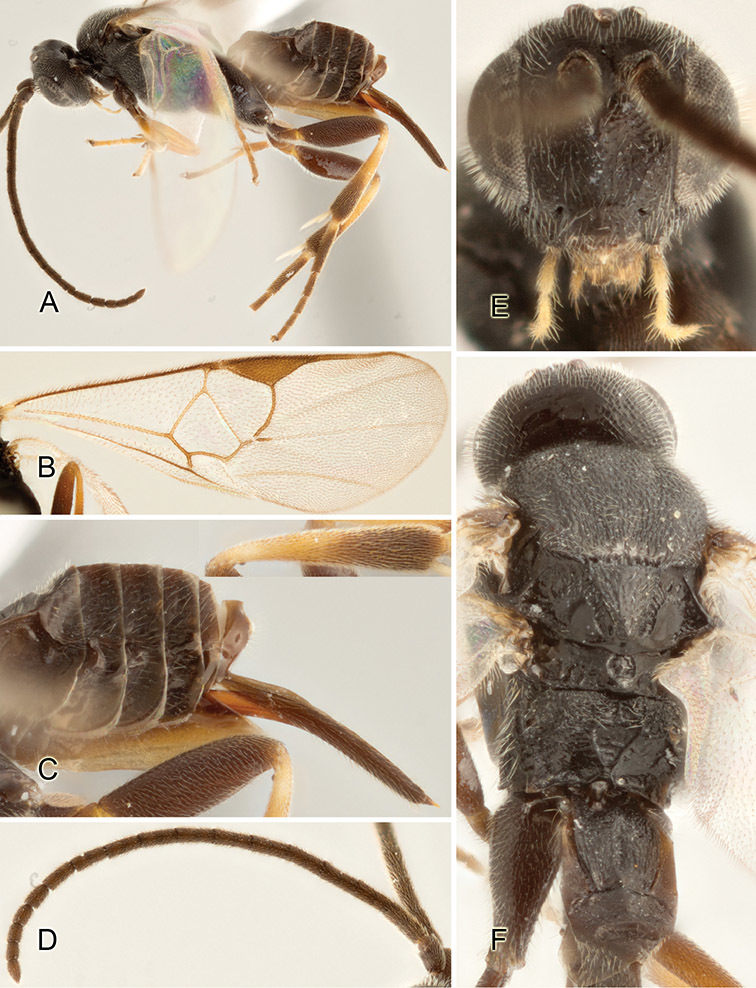
*Apanteles joseperezi*. **A** Habitus, lateral view **B** Fore wing **C** Hypopygium and ovipositor sheats **D** Antenna **E** Head, frontal view **F** Head, meso- and metasoma (partially), dorsal view.

**Figure 60.**
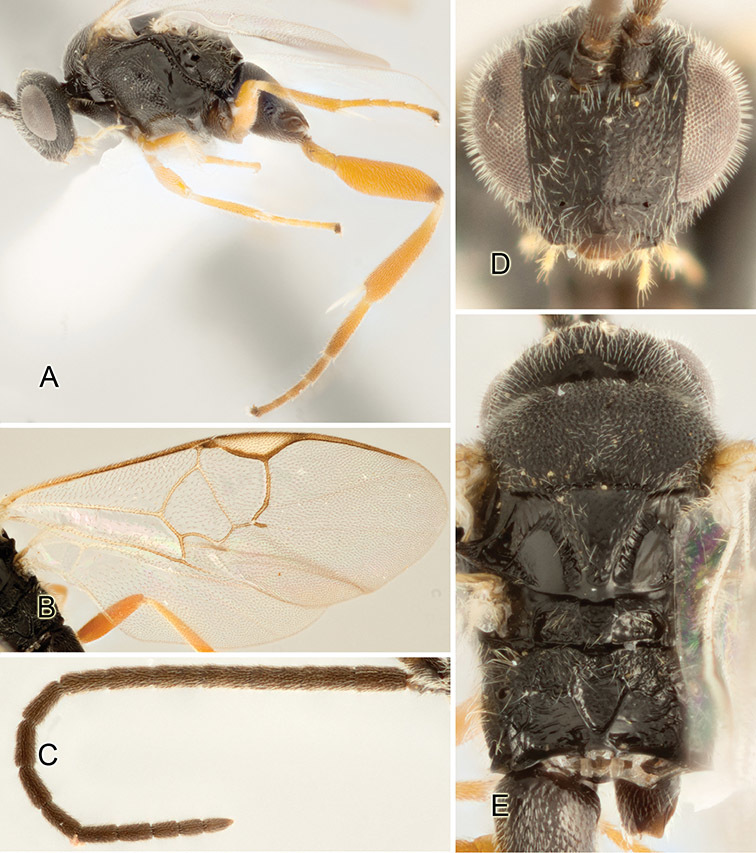
*Apanteles luciariosae*. **A** Habitus, lateral view **B** Fore wing **C** Antenna **D** Head, frontal view **E** Head, meso- and metasoma (partially), dorsal view.

**Figure 61.**
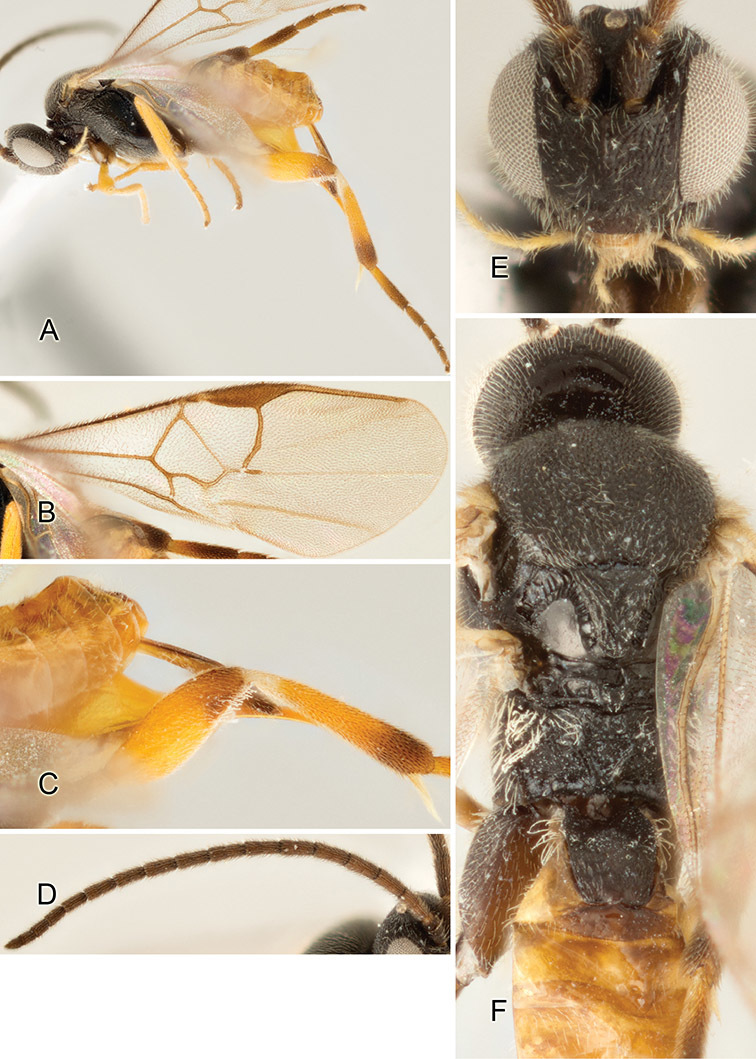
*Apanteles manuelpereirai*. **A** Habitus, lateral view **B** Fore wing **C** Hypopygium and ovipositor sheats **D** Antenna **E** Head, frontal view **F** Head, meso- and metasoma (partially), dorsal view.

**Figure 62.**
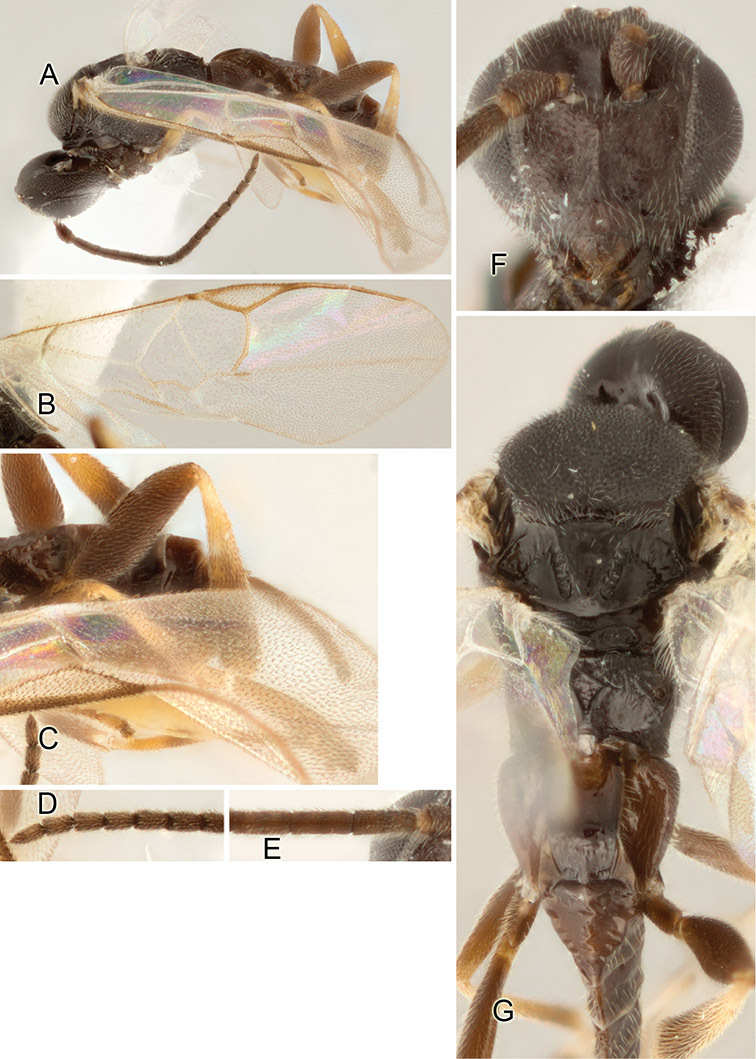
*Apanteles marianopereirai*. **A** Habitus, lateral view **B** Fore wing **C** Hypopygium and ovipositor sheats (partially) **D** Posterior half of antenna **E** Anterior half of antenna **F** Head, frontal view **G** Head, meso- and metasoma (partially), dorsal view.

**Figure 63.**
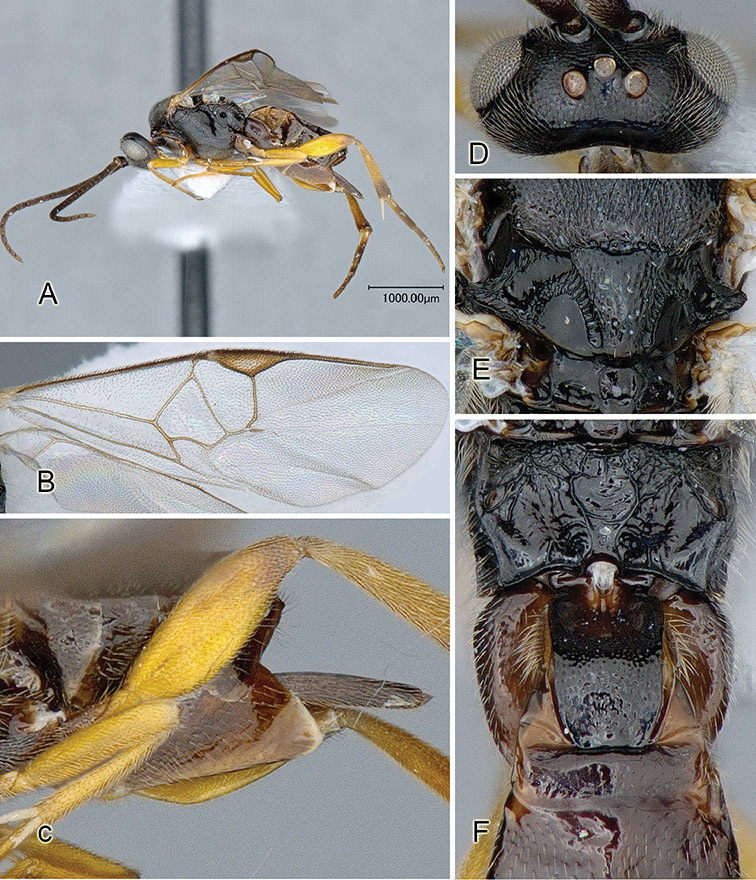
*Apanteles osvaldoespinozai*. **A** Habitus, lateral view **B** Fore wing **C** Hypopygium and ovipositor sheats **D** Head, dorsal view **E** Mesosoma (partially), dorsal view. Propodeum and metasoma (partially), dorsal view.

**Figure 64.**
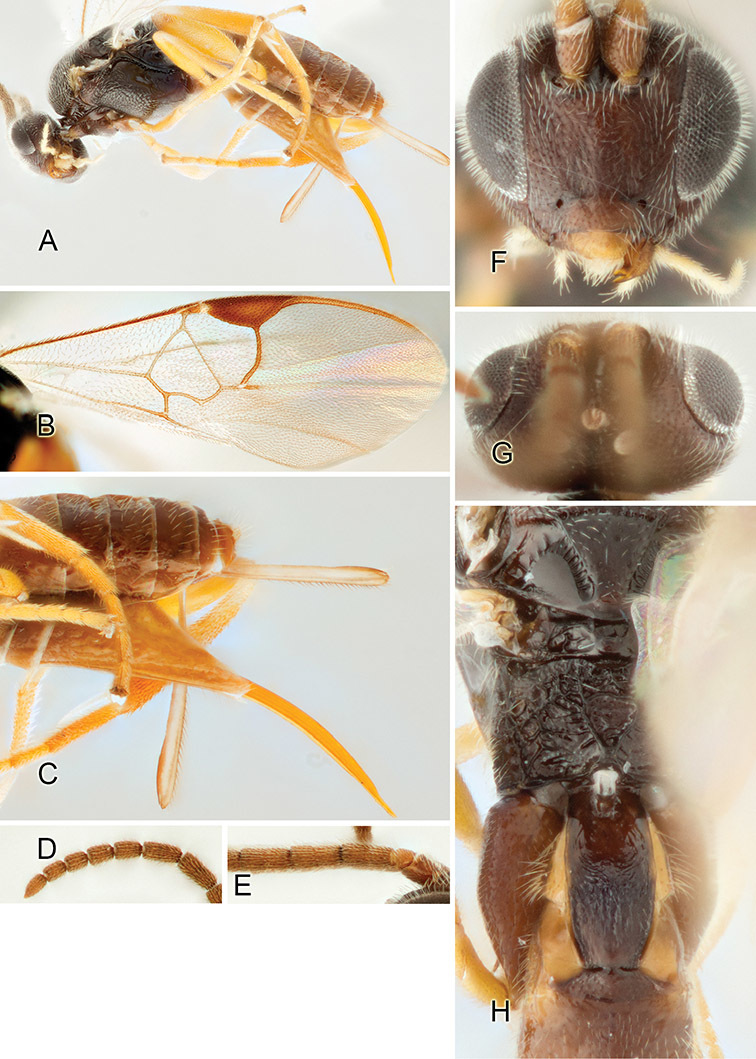
*Apanteles ruthfrancoae*. **A** Habitus, lateral view **B** Fore wing **C** Hypopygium and ovipositor sheats **D** Posterior half of antenna **E** Anterior half of antenna **F** Head, frontal view **G** Head, dorsal view **H** Meso- and metasoma (partially), dorsal view.

**Figure 65.**
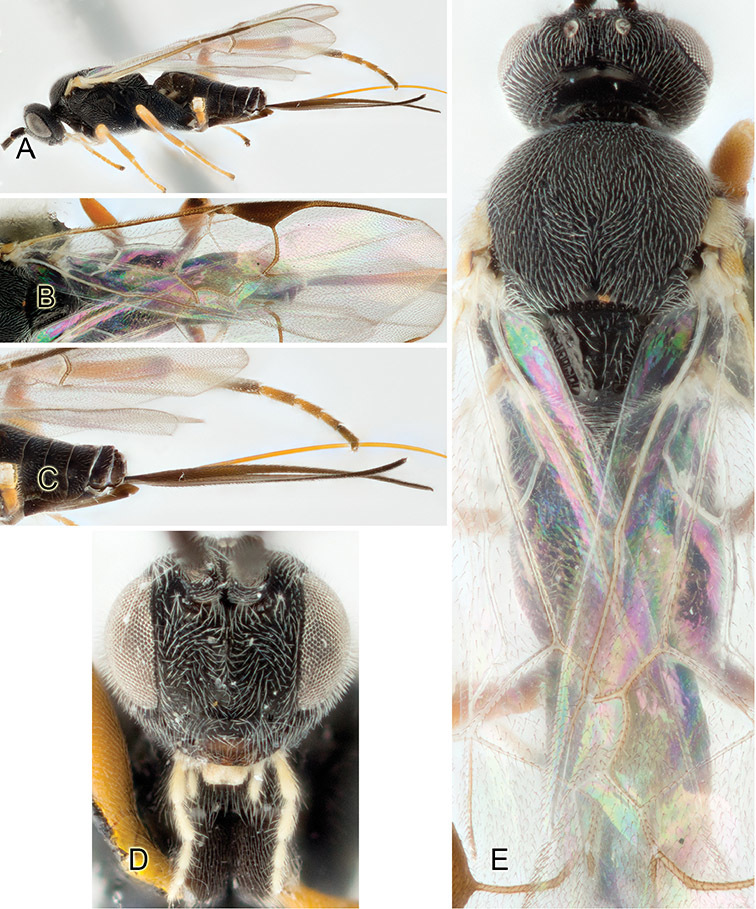
*Apanteles anamarencoae*. **A** Habitus, lateral view **B** Fore wing **C** Hypopygium and ovipositor sheats **D** Head, frontal view **E** Head, meso- and metasoma (partially), dorsal view.

**Figure 66.**
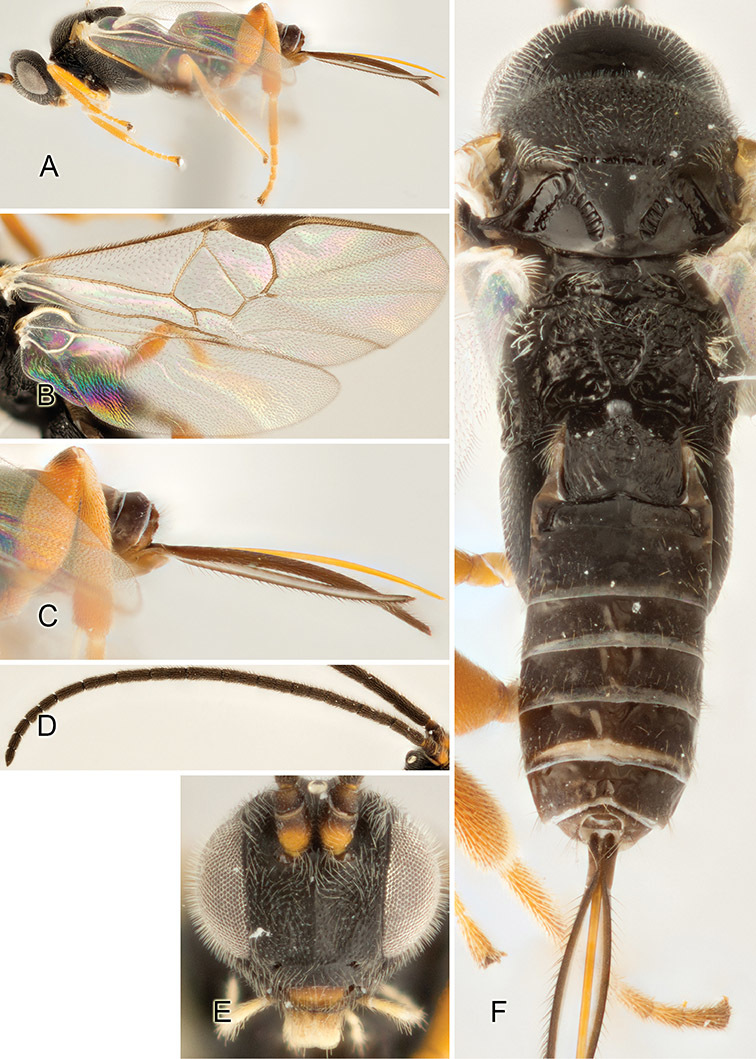
*Apanteles juanlopezi*. **A** Habitus, lateral view **B** Fore wing **C** Hypopygium and ovipositor sheats **D** Antenna **E** Head, frontal view **F** Head, meso- and metasoma, dorsal view.

**Figure 67.**
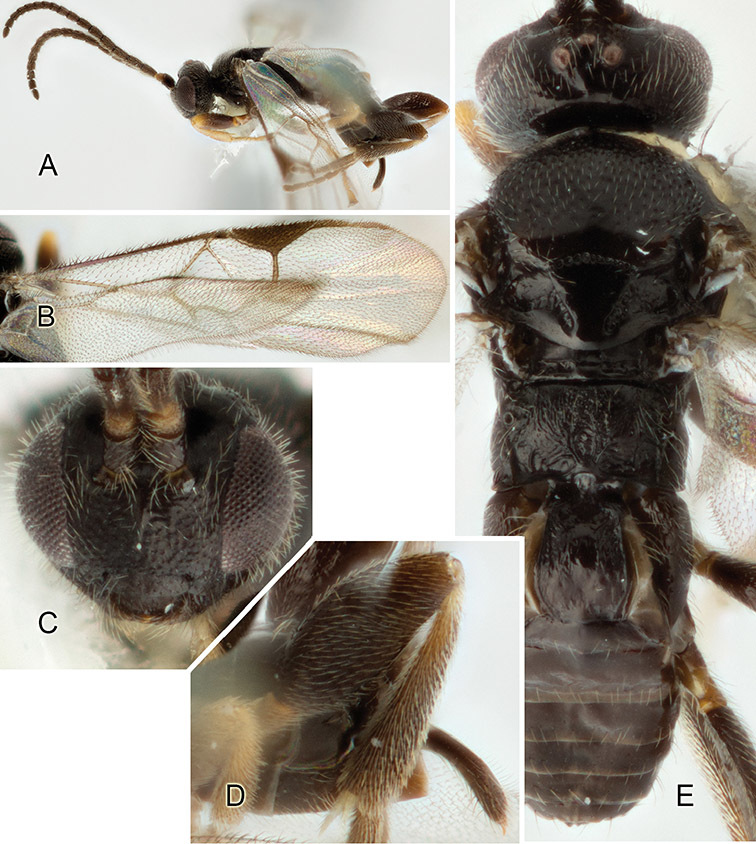
*Apanteles anapiedrae*. **A** Habitus, lateral view **B** Fore wing **C** Head, frontal view **D** Hypopygium and ovipositor sheats **E** Head, meso- and metasoma, dorsal view.

**Figure 68.**
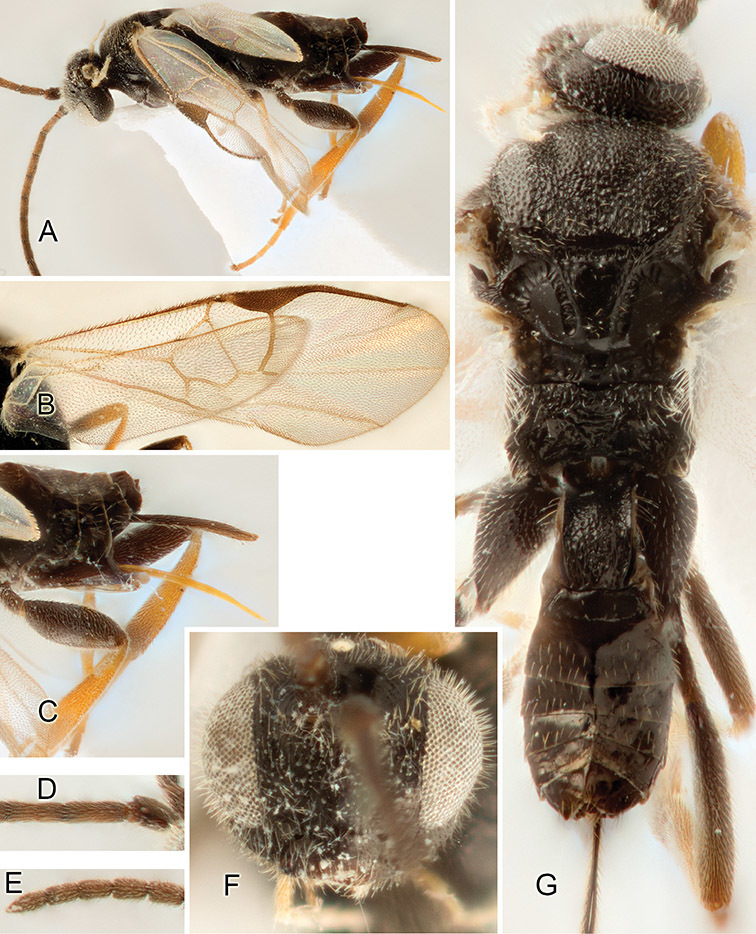
*Apanteles andreacalvoae*. **A** Habitus, lateral view **B** Fore wing **C** Hypopygium and ovipositor sheats **D** Anterior half of antenna **E** Posterior half of antenna **F** Head, frontal view **G** Head, meso- and metasoma, dorsal view.

**Figure 69.**
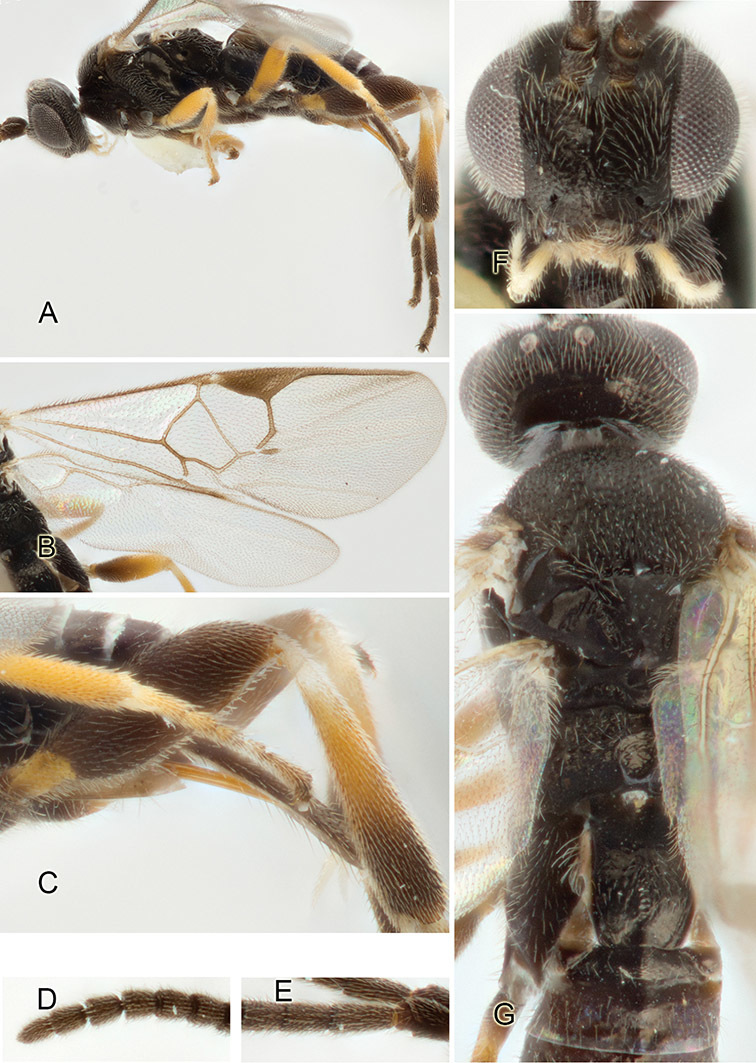
*Apanteles arielopezi*. **A** Habitus, lateral view **B** Fore wing **C** Hypopygium and ovipositor sheats **D** Anterior half of antenna **E** Posterior half of antenna **F** Head, frontal view **G** Head, meso- and metasoma (partially), dorsal view.

**Figure 70.**
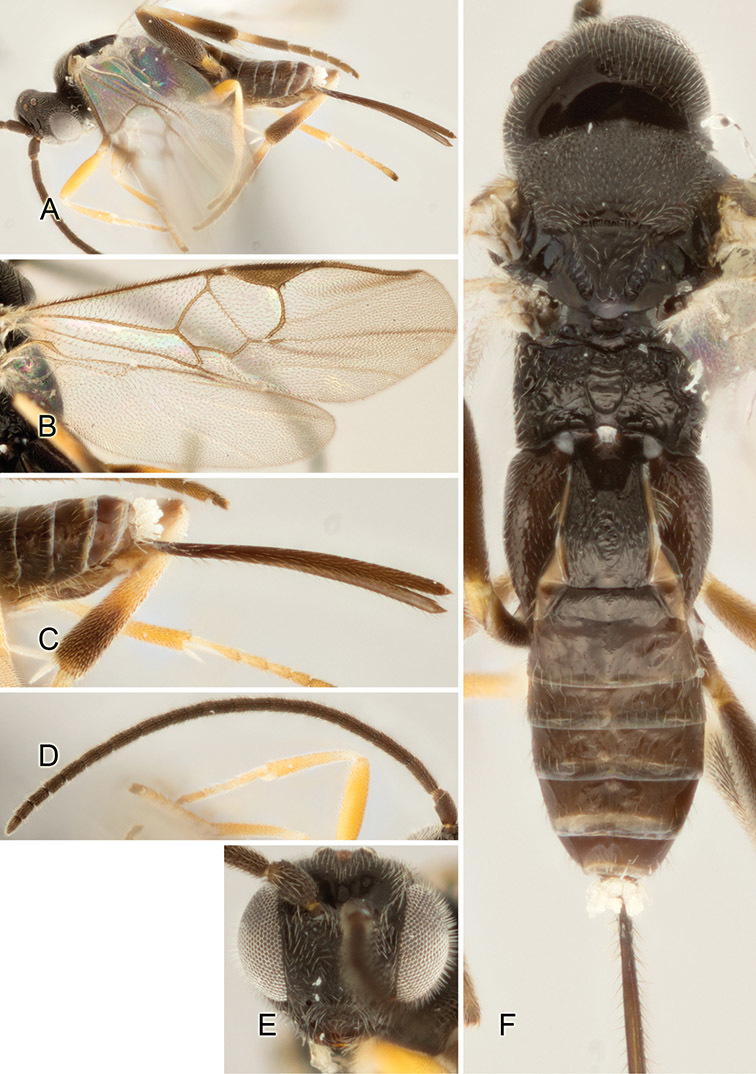
*Apanteles mauriciogurdiani*. **A** Habitus, lateral view **B** Fore wing **C** Hypopygium and ovipositor sheats **D** Antenna **E** Head, frontal view **F** Head, meso- and metasoma, dorsal view.

**Figure 71.**
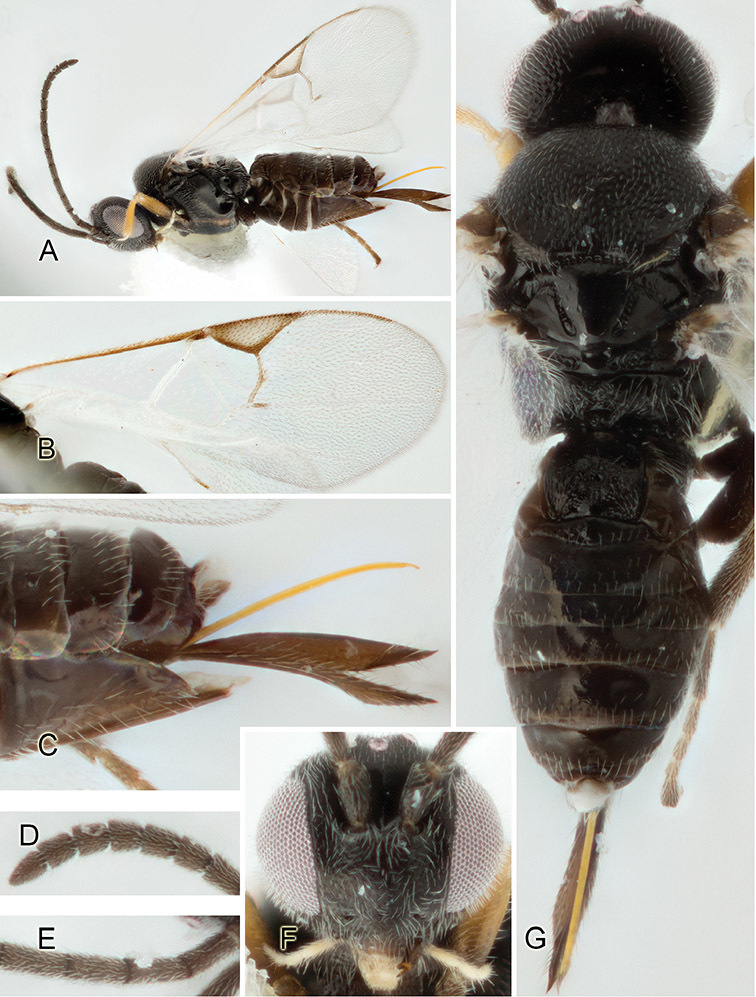
*Apanteles anariasae*. **A** Habitus, lateral view **B** Fore wing **C** Hypopygium and ovipositor sheats **D** Posterior half of antenna **E** Anterior half of antenna **F** Head, frontal view **G** Head, meso- and metasoma, dorsal view.

**Figure 72.**
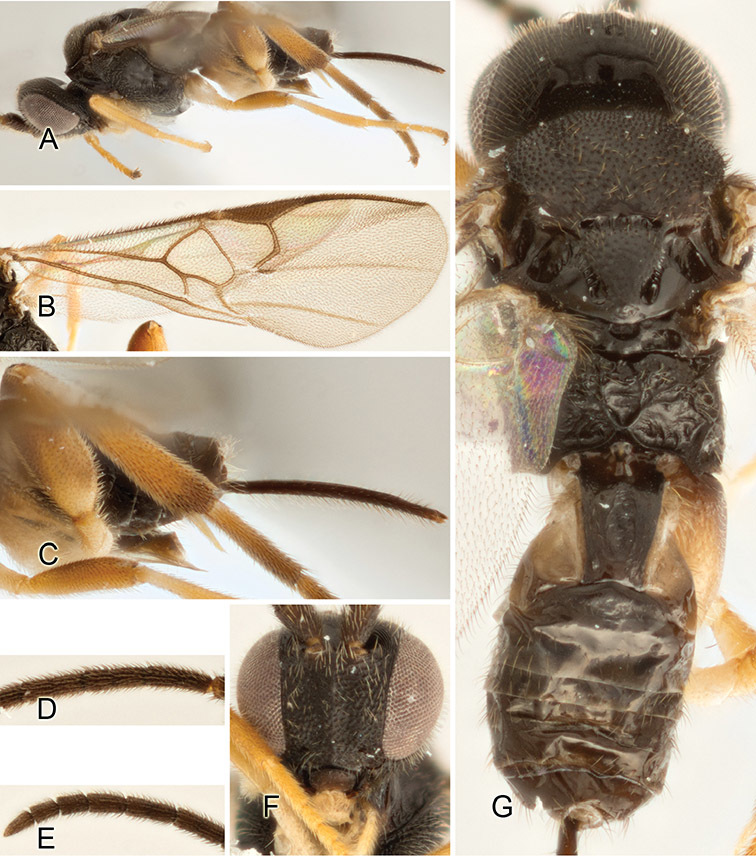
*Apanteles cristianalemani*. **A** Habitus, lateral view **B** Fore wing **C** Hypopygium and ovipositor sheats **D** Anterior half of antenna **E** Posterior half of antenna **F** Head, frontal view **G** Head, meso- and metasoma, dorsal view.

**Figure 73.**
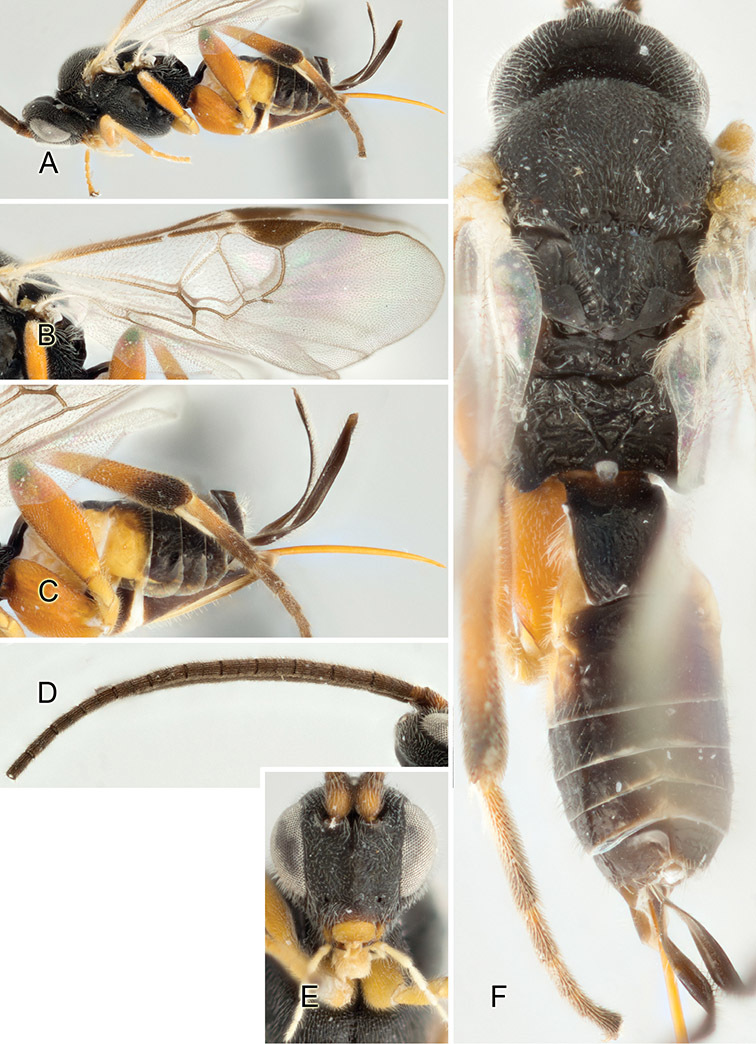
*Apanteles diegoalpizari*. **A** Habitus, lateral view **B** Fore wing **C** Hypopygium and ovipositor sheats **D** Antenna **E** Head, frontal view **F** Head, meso- and metasoma, dorsal view.

**Figure 74.**
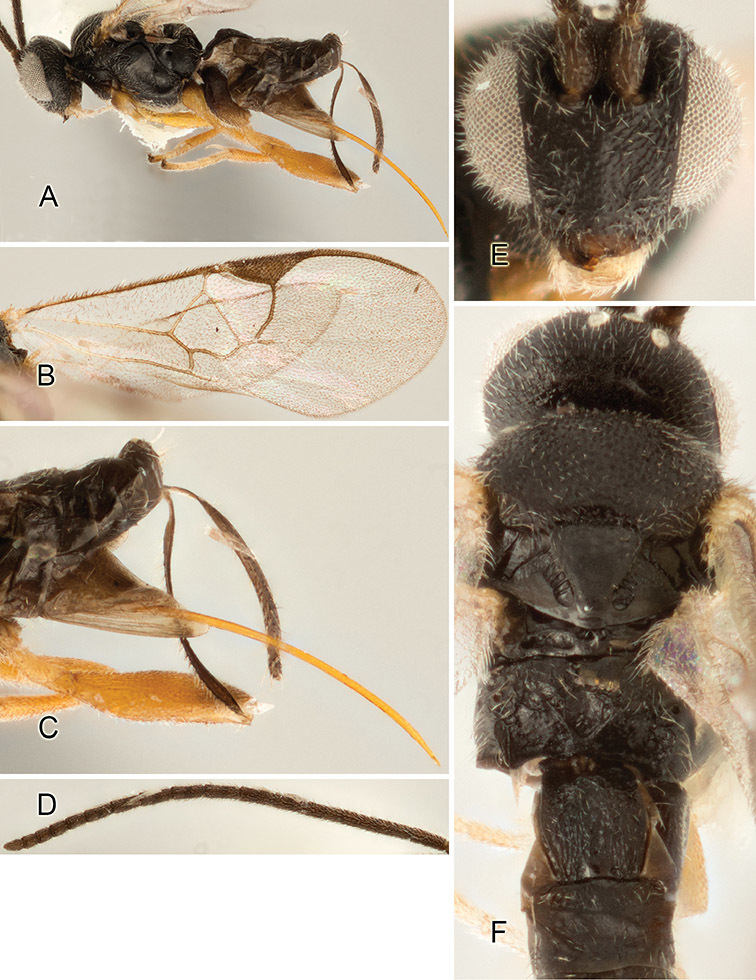
*Apanteles franciscopizarroi*. **A** Habitus, lateral view **B** Fore wing **C** Hypopygium and ovipositor sheats **D** Antenna **E** Head, frontal view **F** Head, meso- and metasoma, dorsal view.

**Figure 75.**
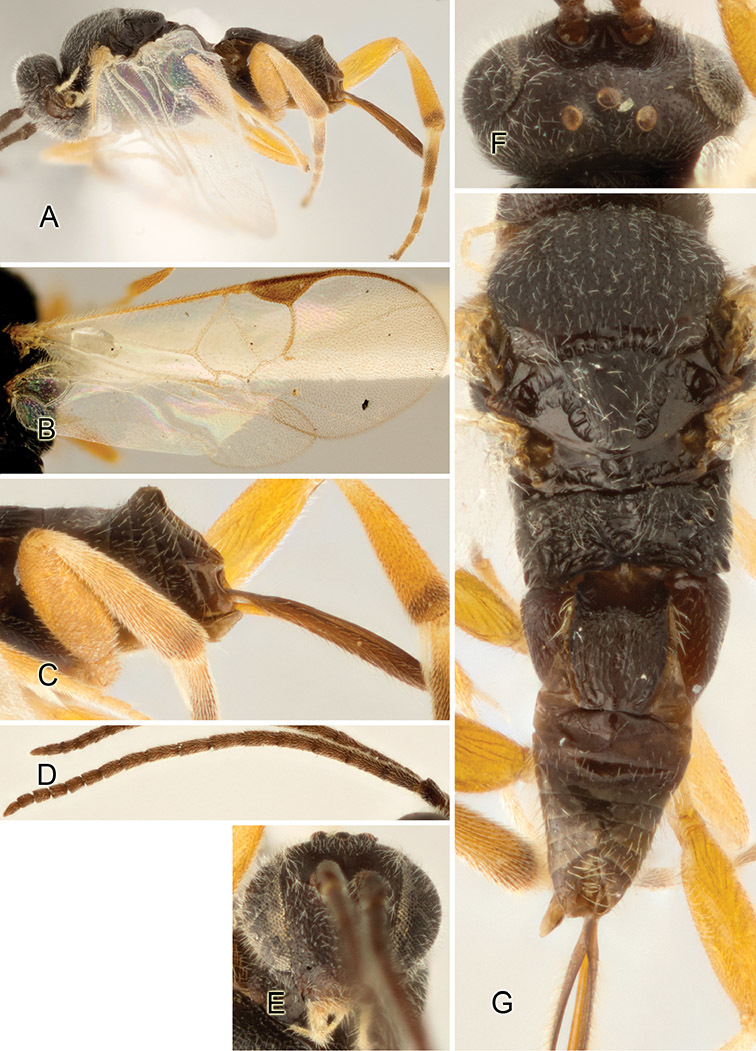
*Apanteles impiger*. **A** Habitus, lateral view **B** Fore wing **C** Hypopygium and ovipositor sheats **D** Antenna **E** Head, frontal view **F** Head, dorsal view **G** Meso- and metasoma, dorsal view.

**Figure 76.**
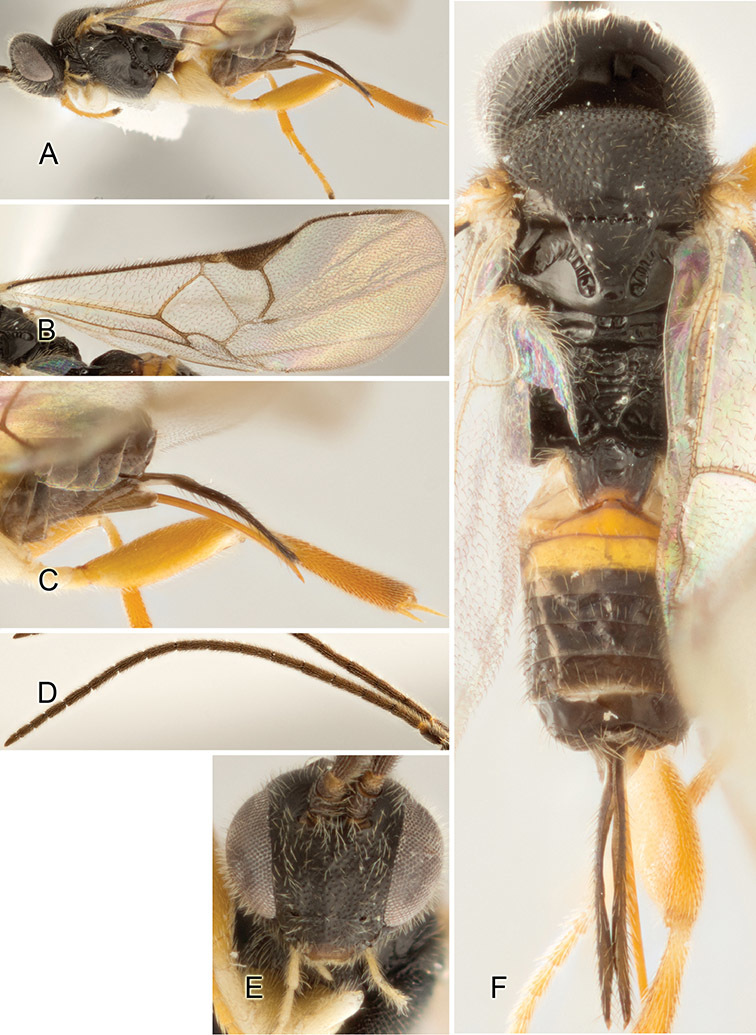
*Apanteles jairomoyai*. **A** Habitus, lateral view **B** Fore wing **C** Hypopygium and ovipositor sheats **D** Antenna **E** Head, frontal view **F** Head, meso- and metasoma, dorsal view.

**Figure 77.**
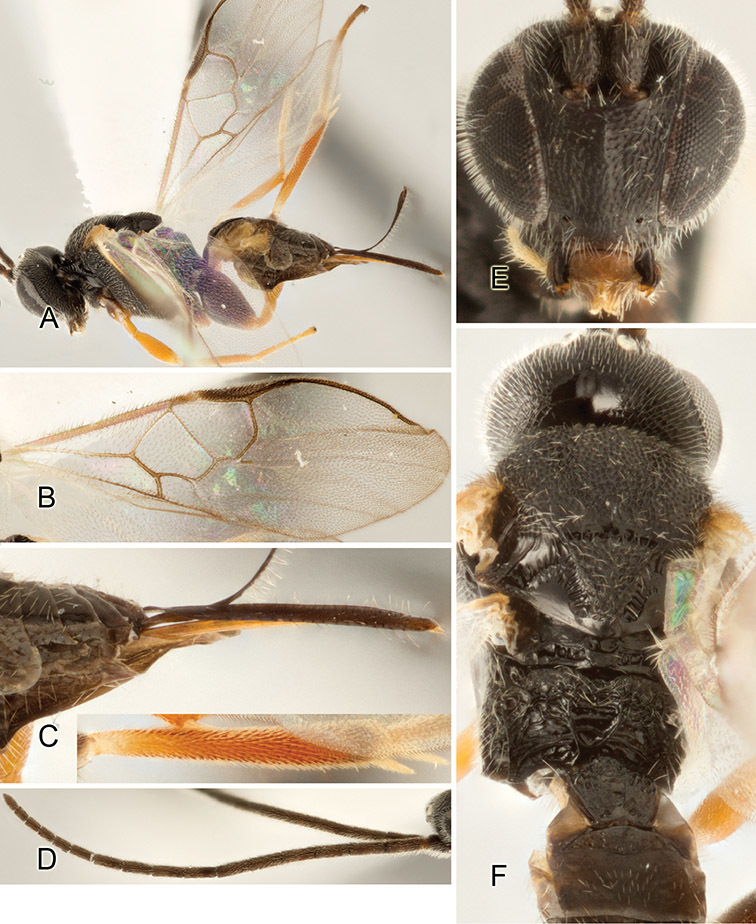
*Apanteles josejaramilloi*. **A** Habitus, lateral view **B** Fore wing **C** Hypopygium and ovipositor sheats, with details of metatibia **D** Antenna **E** Head, frontal view **F** Head, meso- and metasoma (partially), dorsal view.

**Figure 78.**
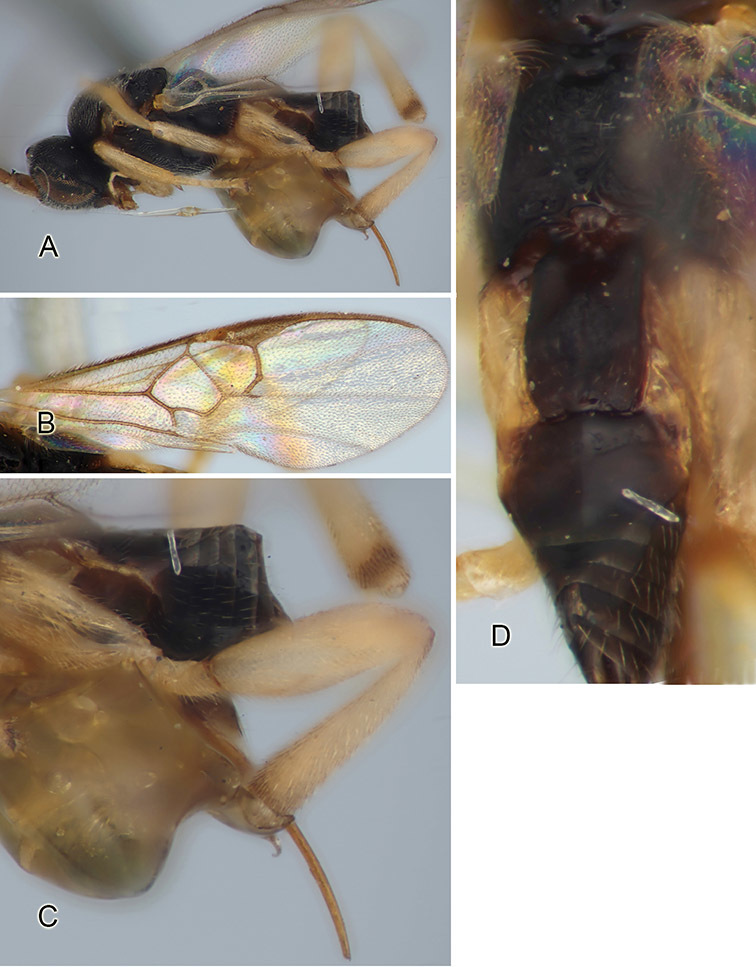
*Apanteles leucopus*. **A** Habitus, lateral view **B** Fore wing **C** Hypopygium and ovipositor sheats (partially) **D** Meso- and metasoma (partially), dorsal view.

**Figure 79.**
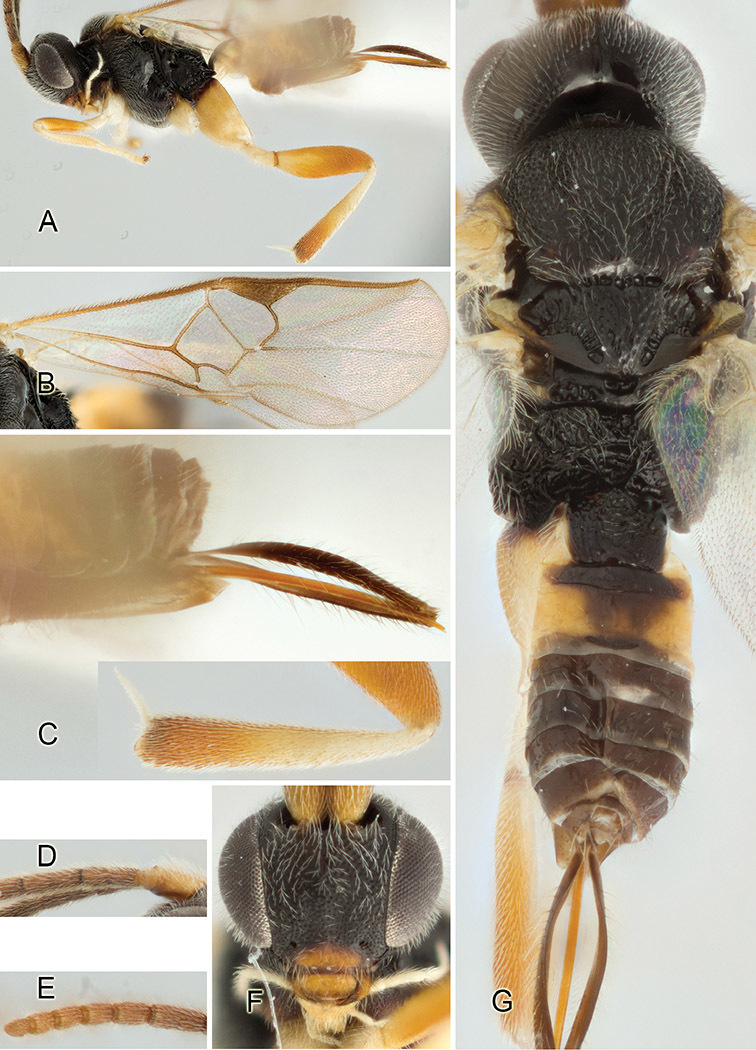
*Apanteles bernyapui*. **A** Habitus, lateral view **B** Fore wing **C** Hypopygium and ovipositor sheats, with details of metatibia **D** Anterior half of antenna **E** Posterior half of antenna **F** Head, frontal view **G** Head, meso- and metasoma, dorsal view.

**Figure 80.**
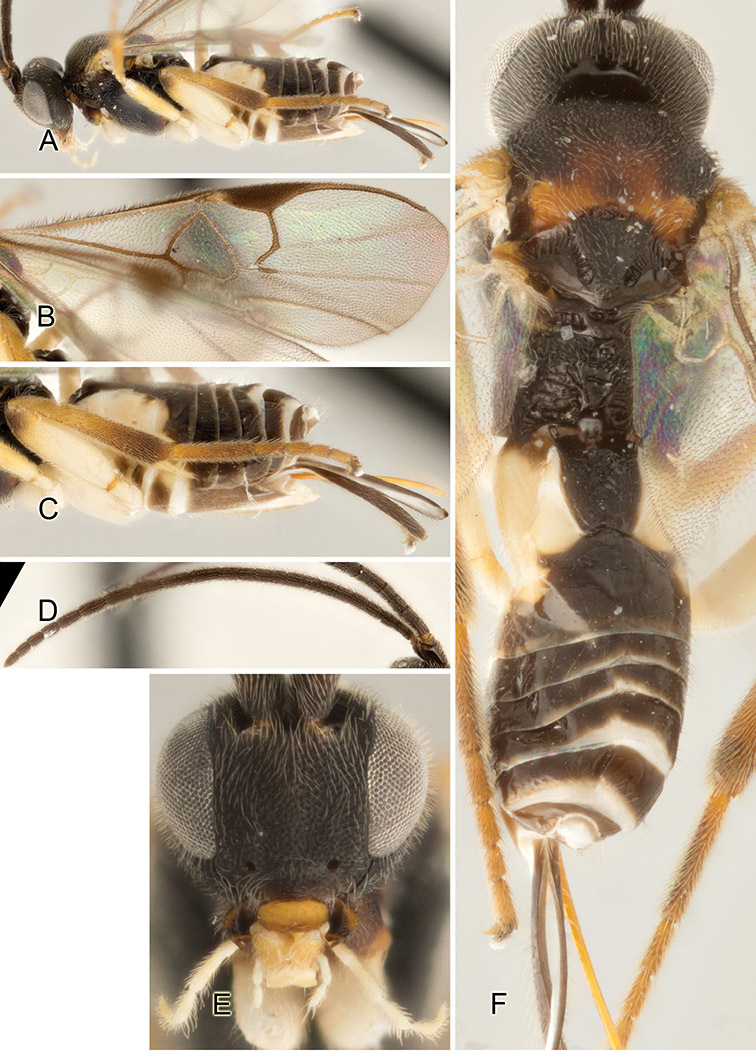
*Apanteles javiersihezari*. **A** Habitus, lateral view **B** Fore wing **C** Hypopygium and ovipositor sheats **D** Antenna **E** Head, frontal view **F** Head, meso- and metasoma, dorsal view.

**Figure 81.**
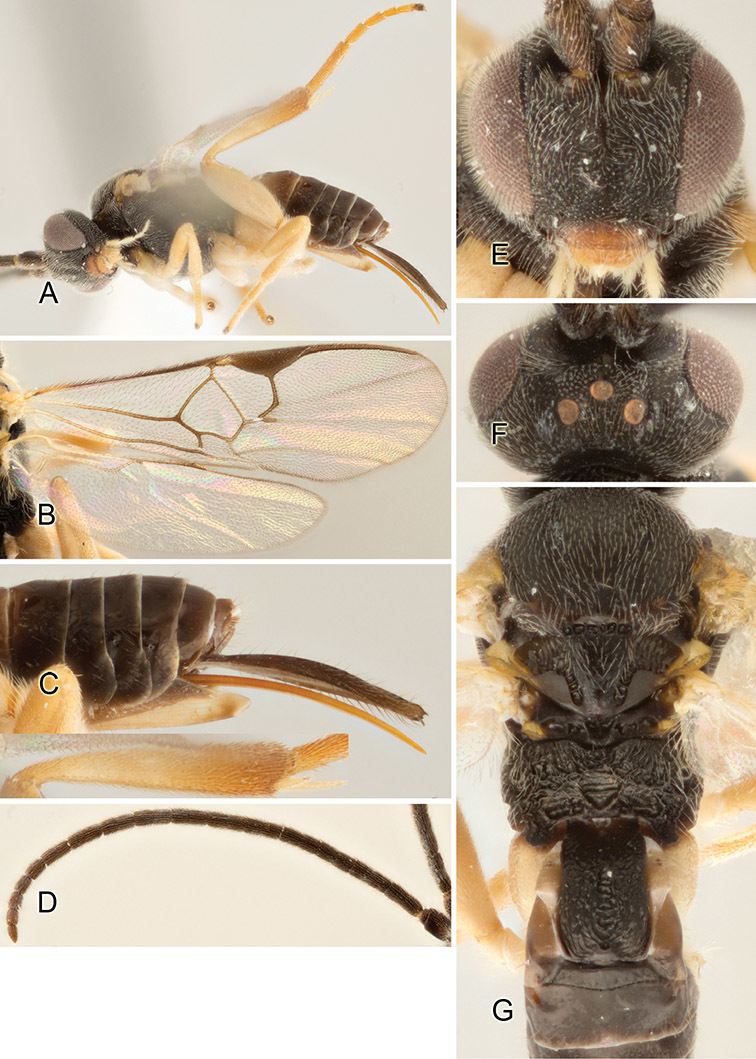
*Apanteles raulacevedoi*. **A** Habitus, lateral view **B** Fore wing **C** Hypopygium and ovipositor sheats **D** Antenna **E** Head, frontal view **F** Head, dorsal view **G** Meso- and metasoma (partially), dorsal view.

**Figure 82.**
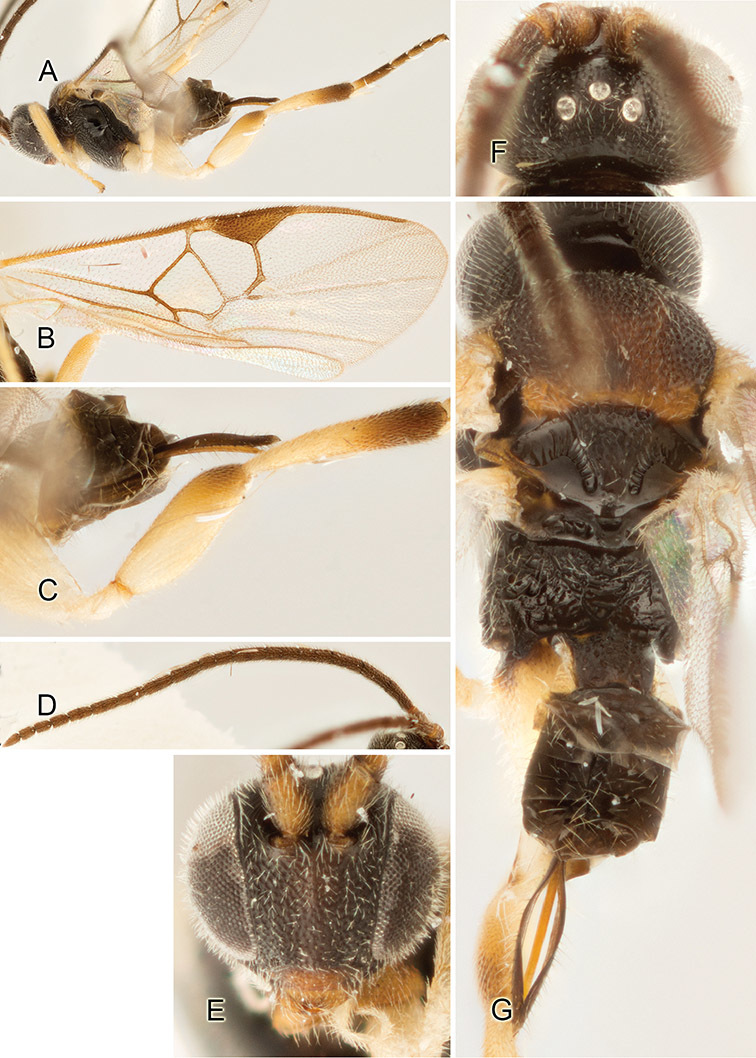
*Apanteles victorbarrantesi*. **A** Habitus, lateral view **B** Fore wing **C** Hypopygium and ovipositor sheats **D** Antenna **E** Head, frontal view **F** Head, dorsal view **G** Meso- and metasoma, dorsal view.

**Figure 83.**
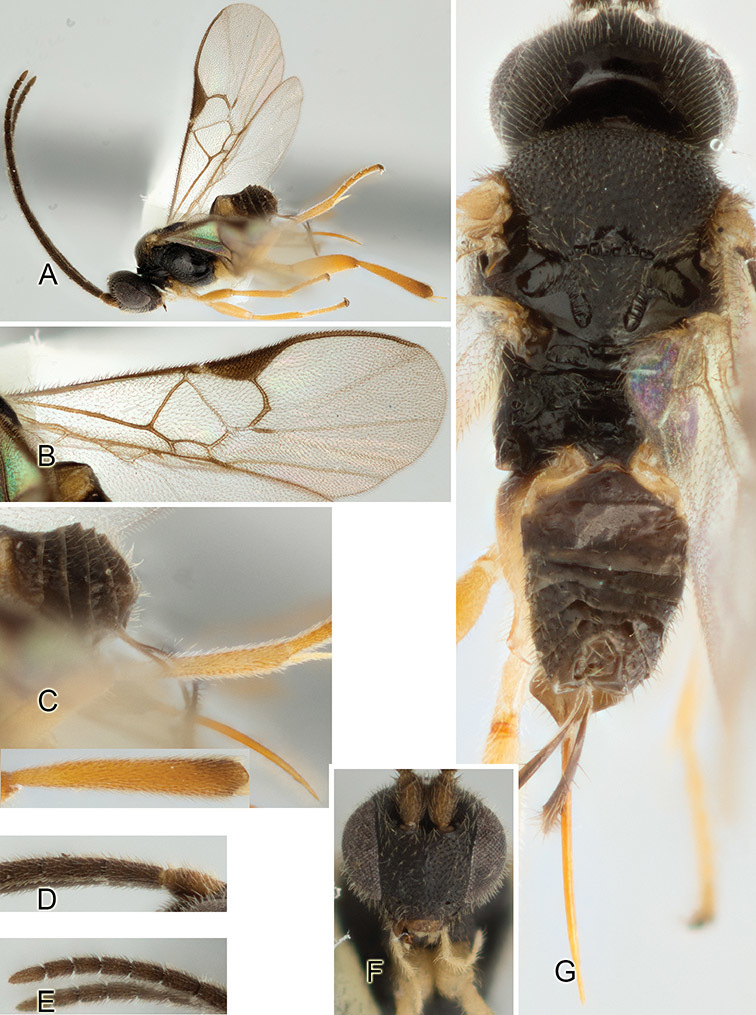
*Apanteles bettymarchenae*. **A** Habitus, lateral view **B** Fore wing **C** Hypopygium and ovipositor sheats, with details of metatibia **D** Anterior half of antenna **E** Posterior half of antenna **F** Head, frontal view **G** Head, meso- and metasoma, dorsal view.

**Figure 84.**
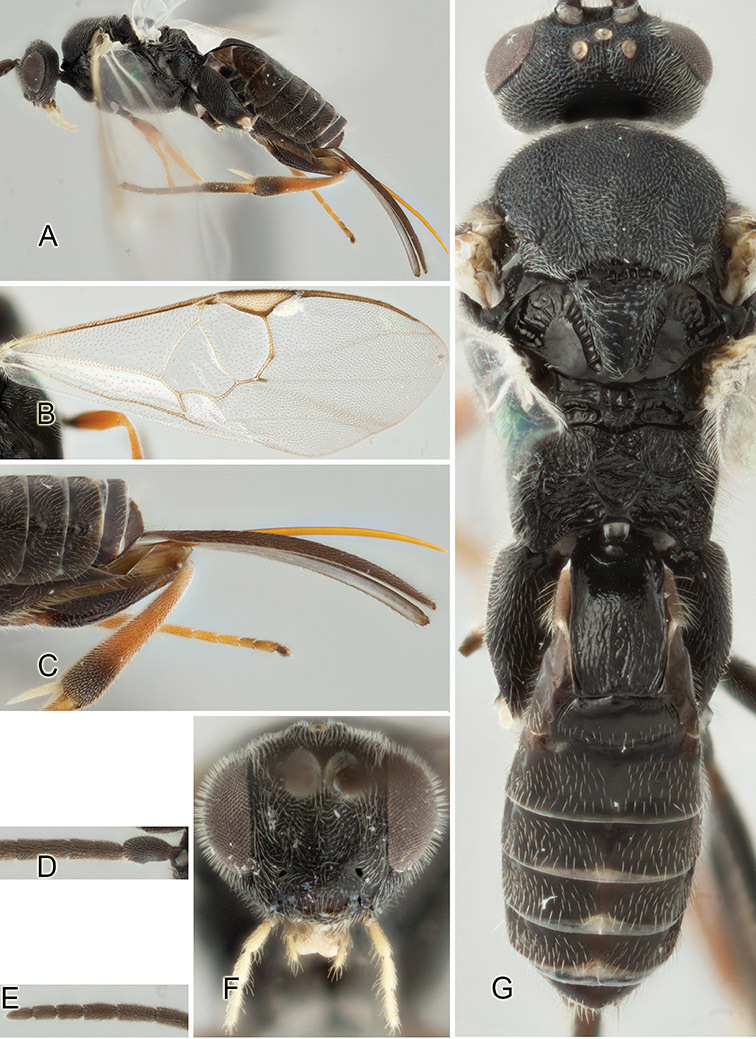
*Apanteles bienvenidachavarriae*. **A** Habitus, lateral view **B** Fore wing **C** Hypopygium and ovipositor sheats **D** Anterior half of antenna **E** Posterior half of antenna **F** Head, frontal view **G** Head, meso- and metasoma, dorsal view.

**Figure 85.**
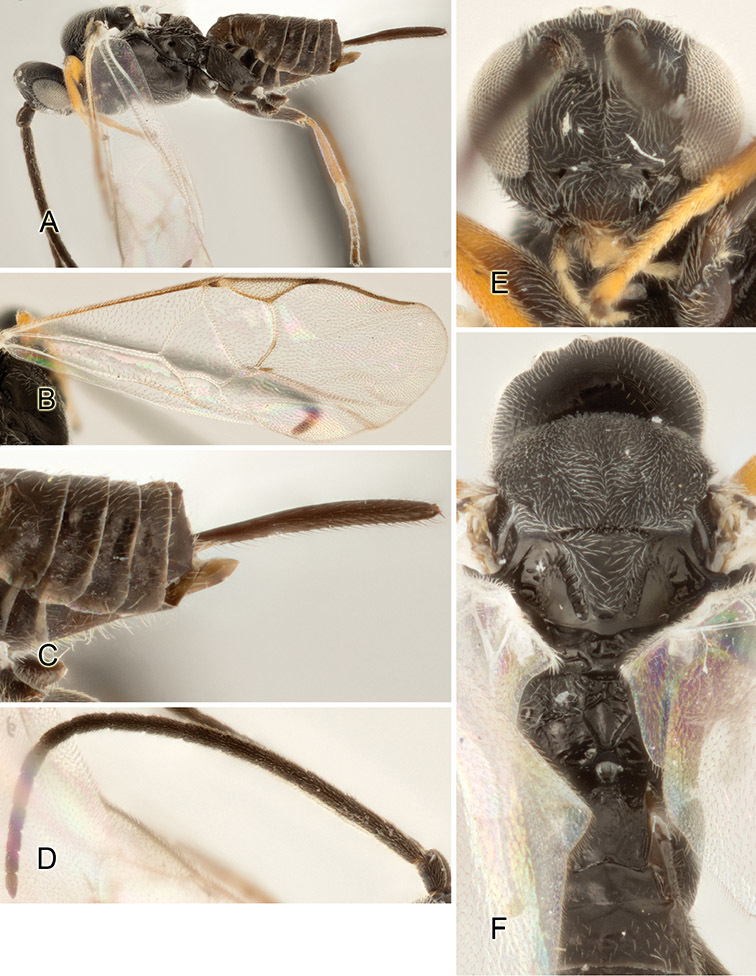
*Apanteles josecalvoi*. **A** Habitus, lateral view **B** Fore wing **C** Hypopygium and ovipositor sheats **D** Antenna **E** Head, frontal view **F** Head, meso- and metasoma (partially), dorsal view.

**Figure 86.**
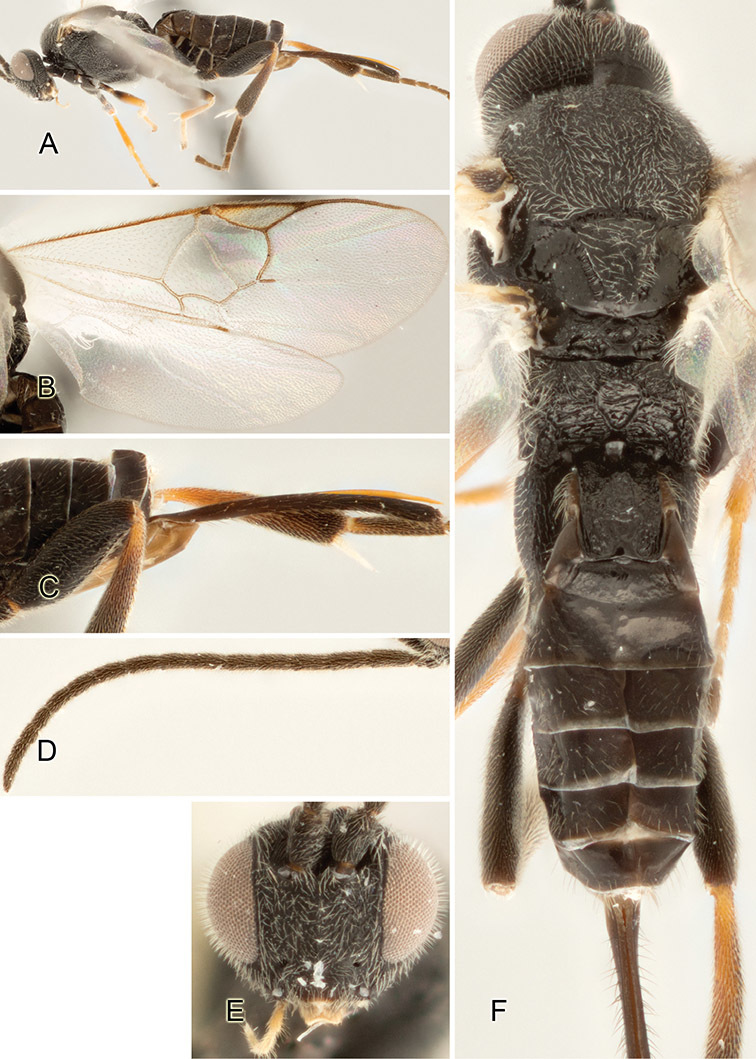
*Apanteles marisolarroyoae*. **A** Habitus, lateral view **B** Fore wing **C** Hypopygium and ovipositor sheats **D** Antenna **E** Head, frontal view **F** Head, meso- and metasoma, dorsal view.

**Figure 87.**
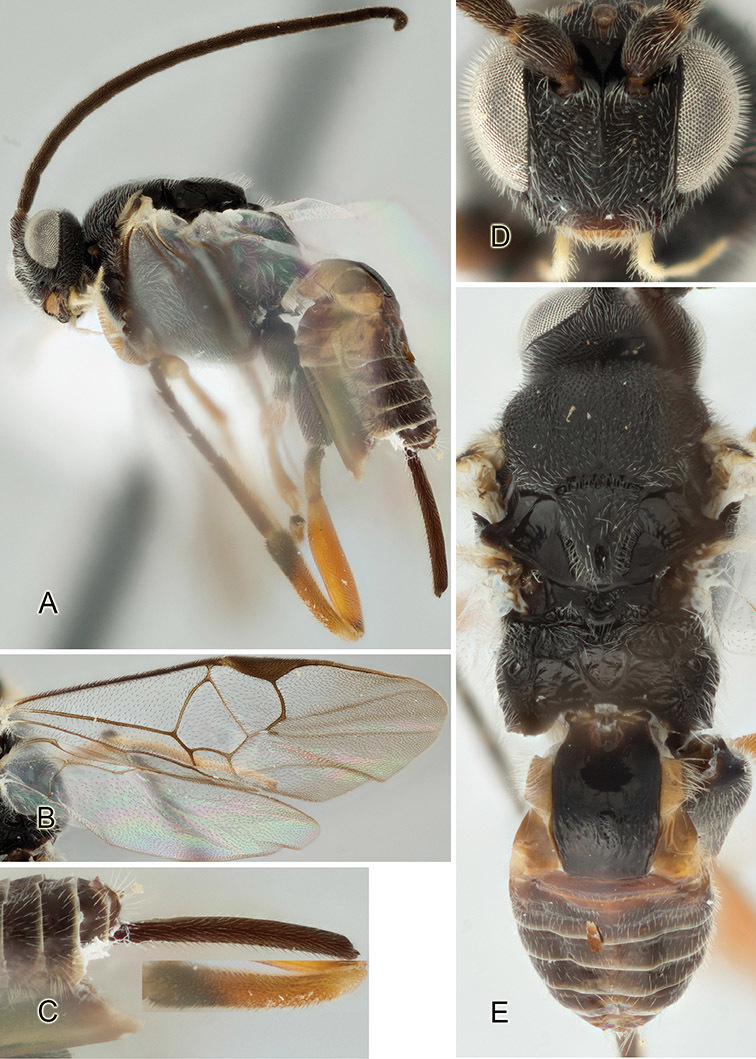
*Apanteles calixtomoragai*. **A** Habitus, lateral view **B** Fore wing **C** Hypopygium and ovipositor sheats, with details of metatibia **D** Head, frontal view **F** Head, meso- and metasoma, dorsal view.

**Figure 88.**
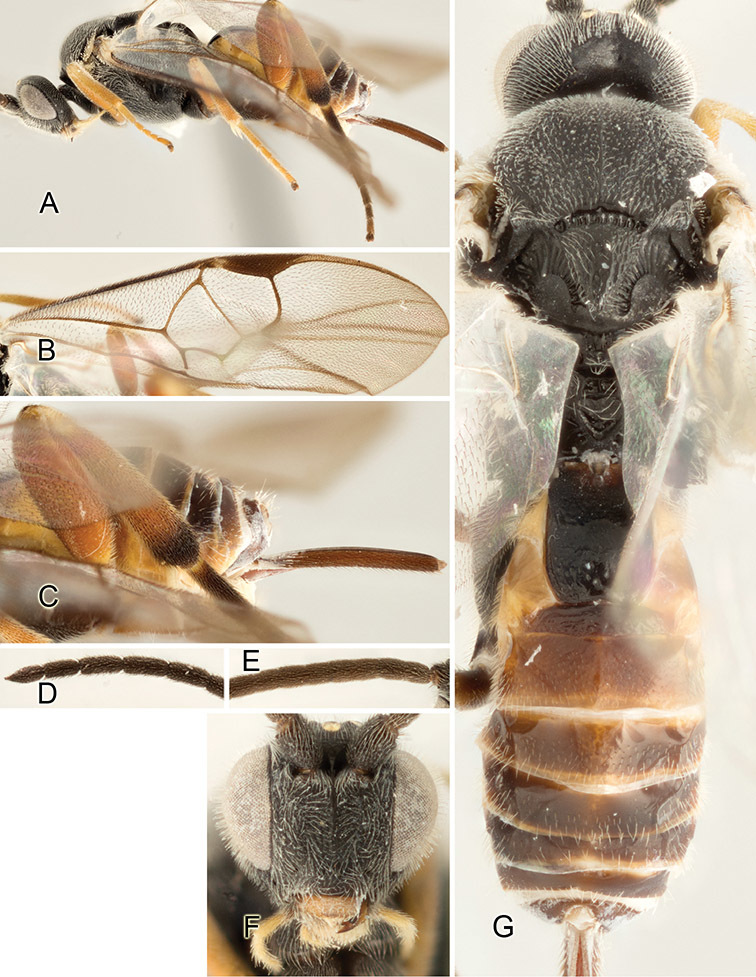
*Apanteles manuelriosi*. **A** Habitus, lateral view **B** Fore wing **C** Hypopygium and ovipositor sheats **D** Posterior half of antenna **E** Anterior half of antenna **F** Head, frontal view **G** Head, meso- and metasoma, dorsal view.

**Figure 89.**
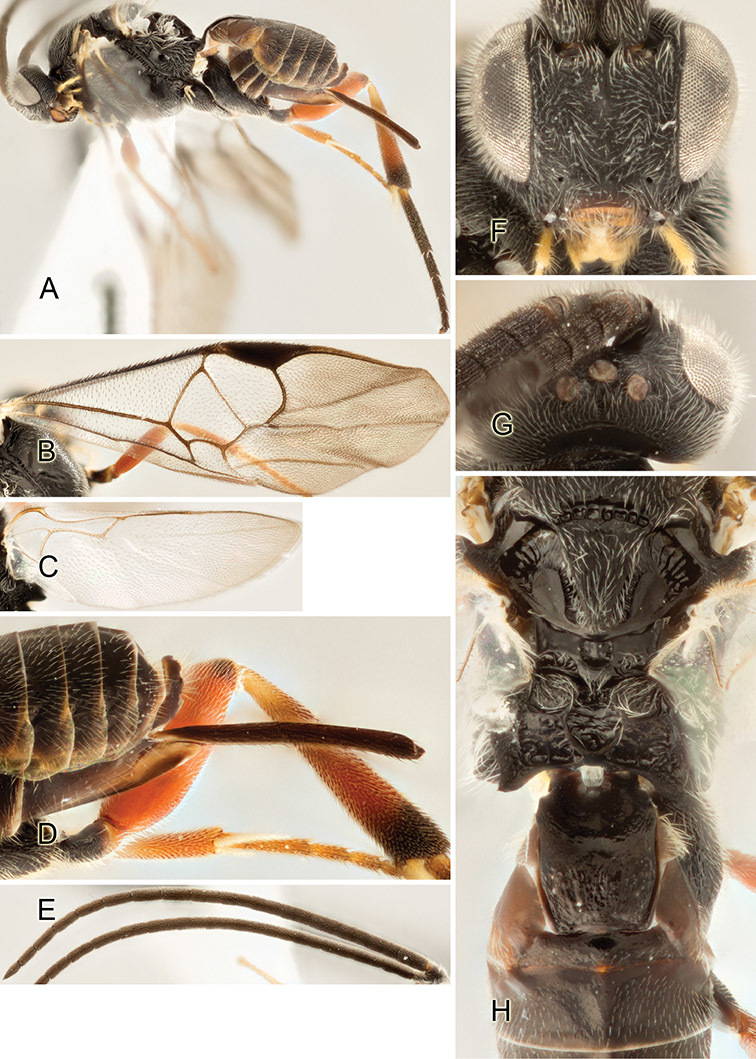
*Apanteles petronariosae*. **A** Habitus, lateral view **B** Fore wing **C** Hind wing **D** Hypopygium and ovipositor sheats **E** Antenna **F** Head, frontal view **G** Head, dorsal view **H** Meso- and metasoma (partially), dorsal view.

**Figure 90.**
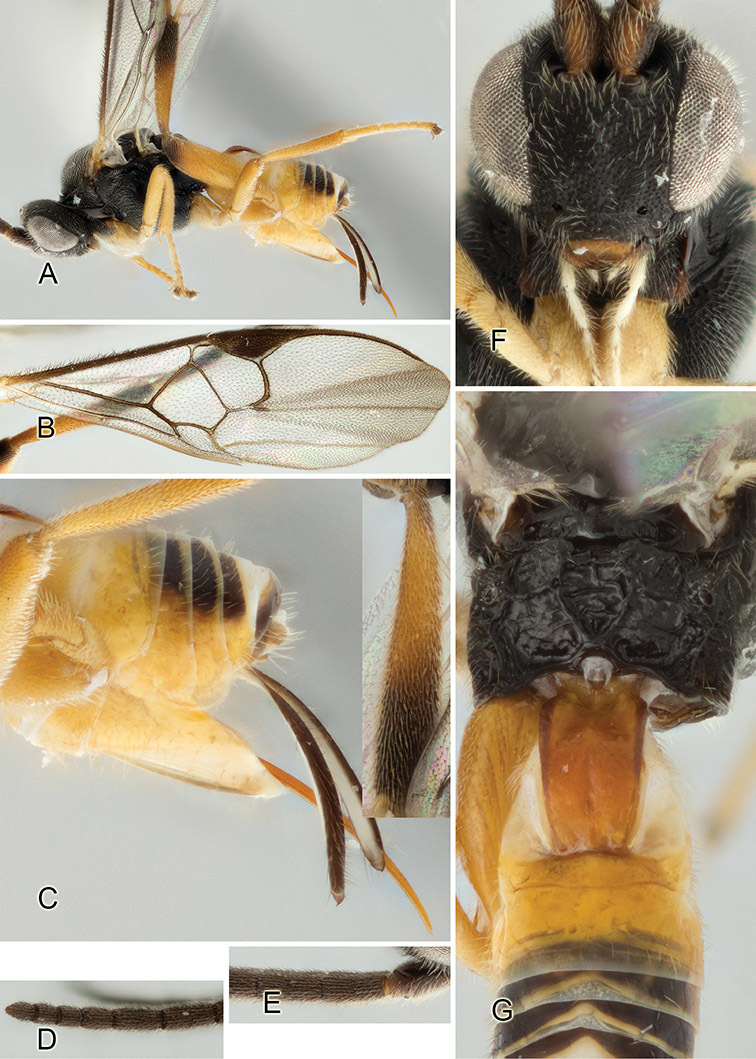
*Apanteles carlosguadamuzi*. **A** Habitus, lateral view **B** Fore wing **C** Hypopygium and ovipositor sheats, with details of metatibia **D** Posterior half of antenna **E** Anterior half of antenna **F** Head, frontal view **G** Meso- and metasoma (partially), dorsal view.

**Figure 91.**
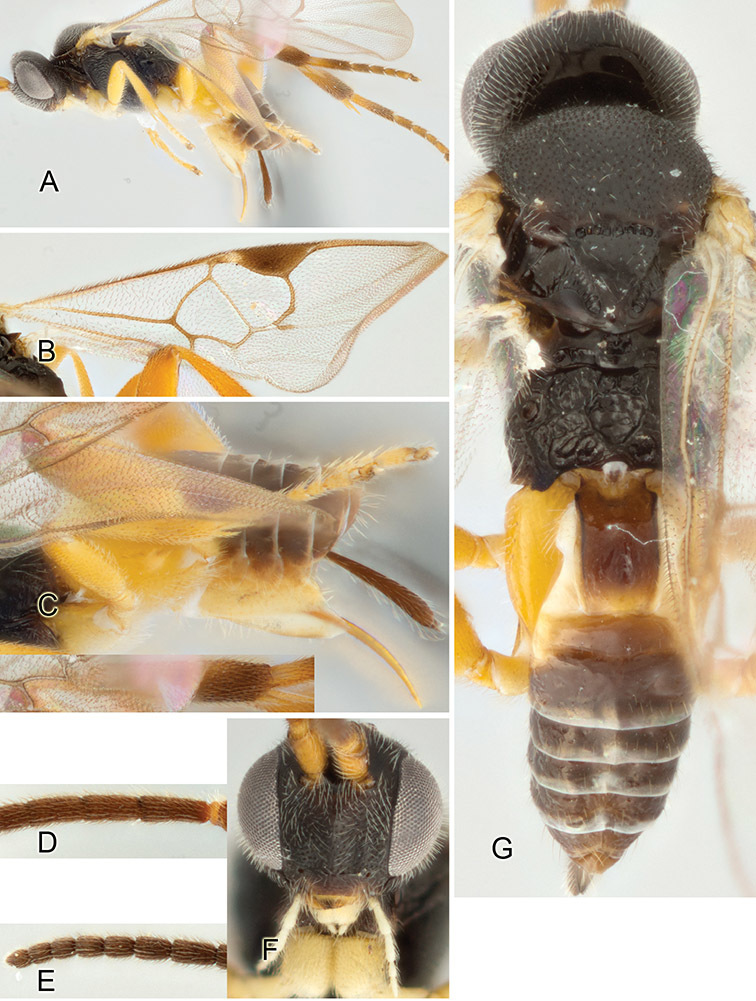
*Apanteles cinthiabarrantesae*. **A** Habitus, lateral view **B** Fore wing **C** Hypopygium and ovipositor sheats, with details of metatibia **D** Posterior half of antenna **E** Anterior half of antenna **F** Head, frontal view **G** Head, meso- and metasoma, dorsal view.

**Figure 92.**
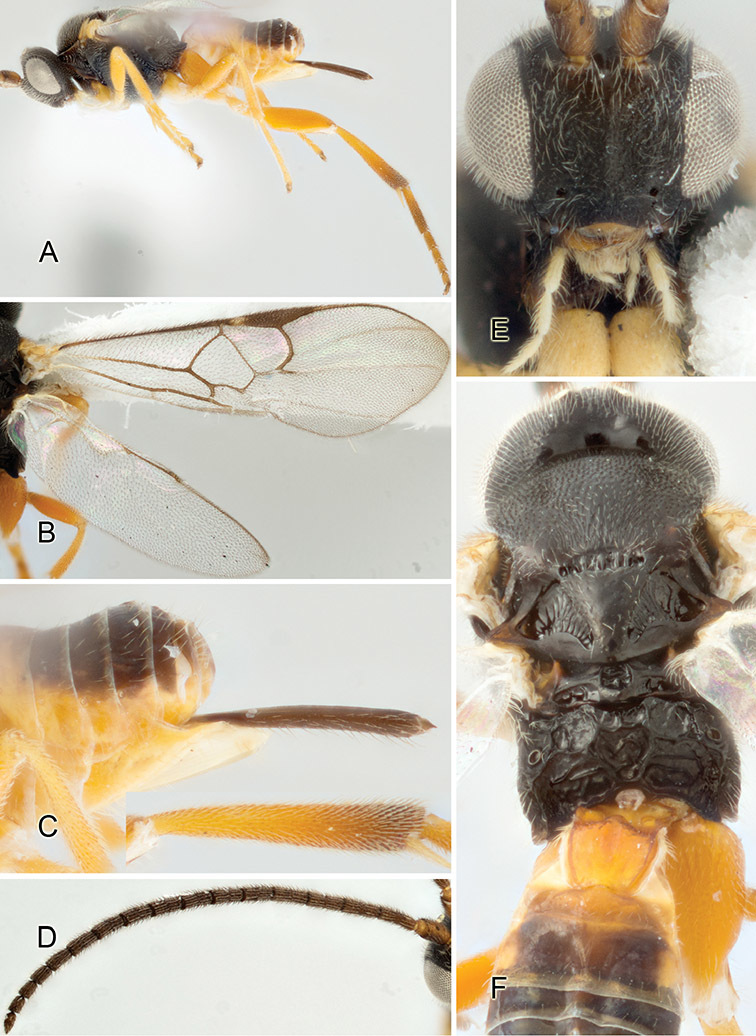
*Apanteles edithlopezae*. **A** Habitus, lateral view **B** Fore wing **C** Hypopygium and ovipositor sheats **D** Antenna **E** Head, frontal view **F** Head, meso- and metasoma (partially), dorsal view.

**Figure 93.**
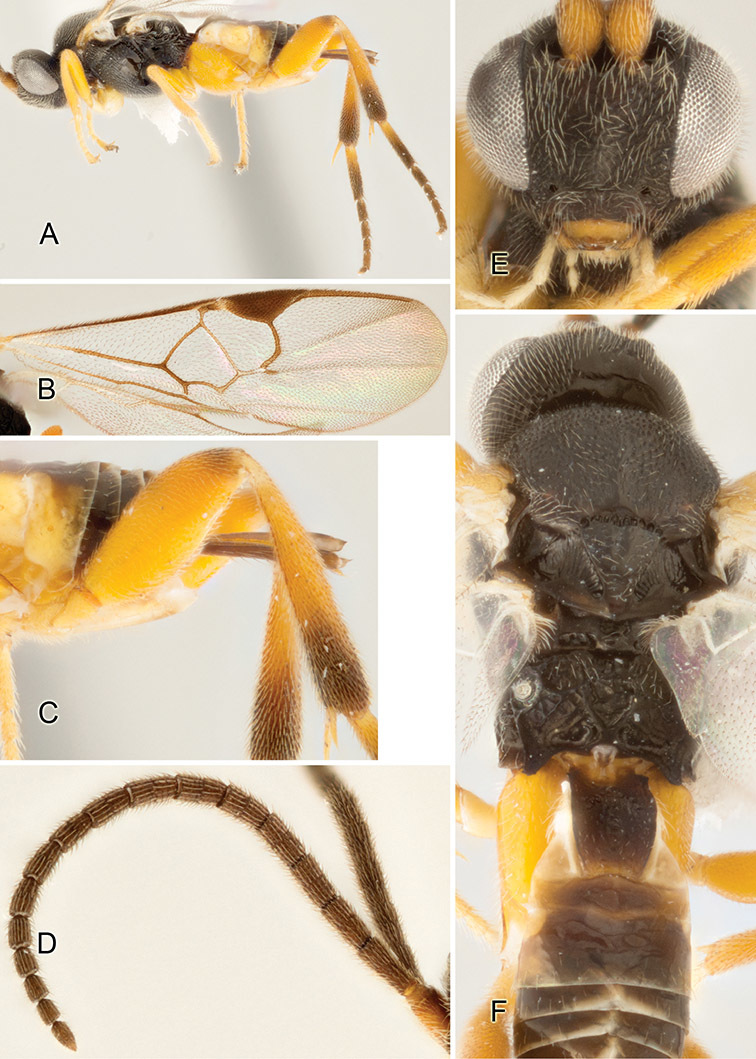
*Apanteles javiercontrerasi*. **A** Habitus, lateral view **B** Fore wing **C** Hypopygium and ovipositor sheats **D** Antenna **E** Head, frontal view **F** Head, meso- and metasoma (partially), dorsal view.

**Figure 94.**
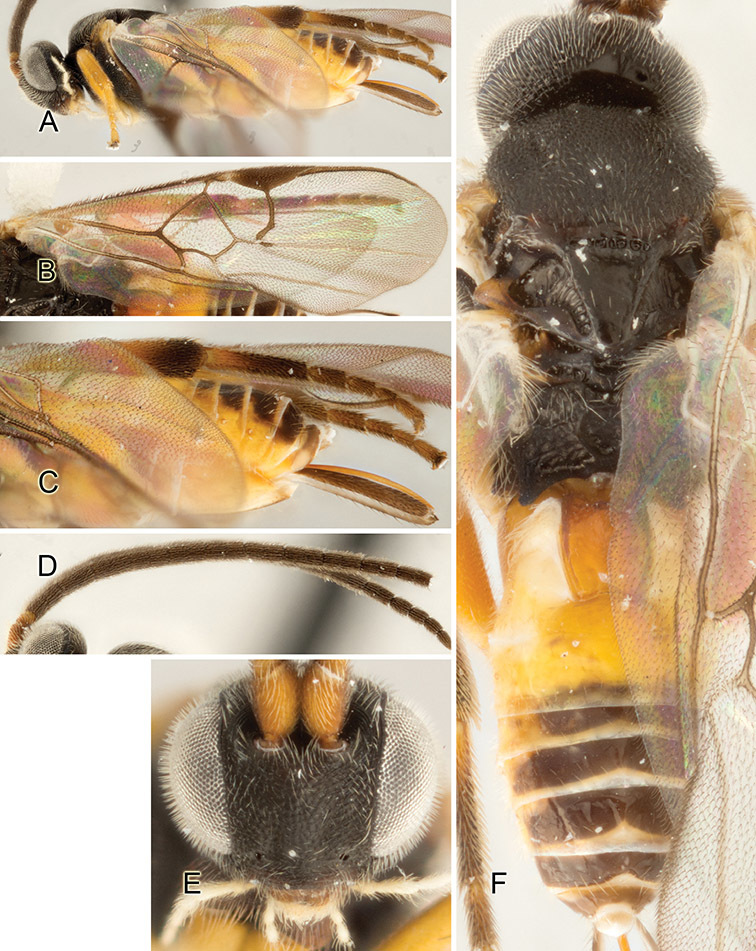
*Apanteles jesusbrenesi*. **A** Habitus, lateral view **B** Fore wing **C** Hypopygium and ovipositor sheats **D** Antenna **E** Head, frontal view **F** Head, meso- and metasoma, dorsal view.

**Figure 95.**
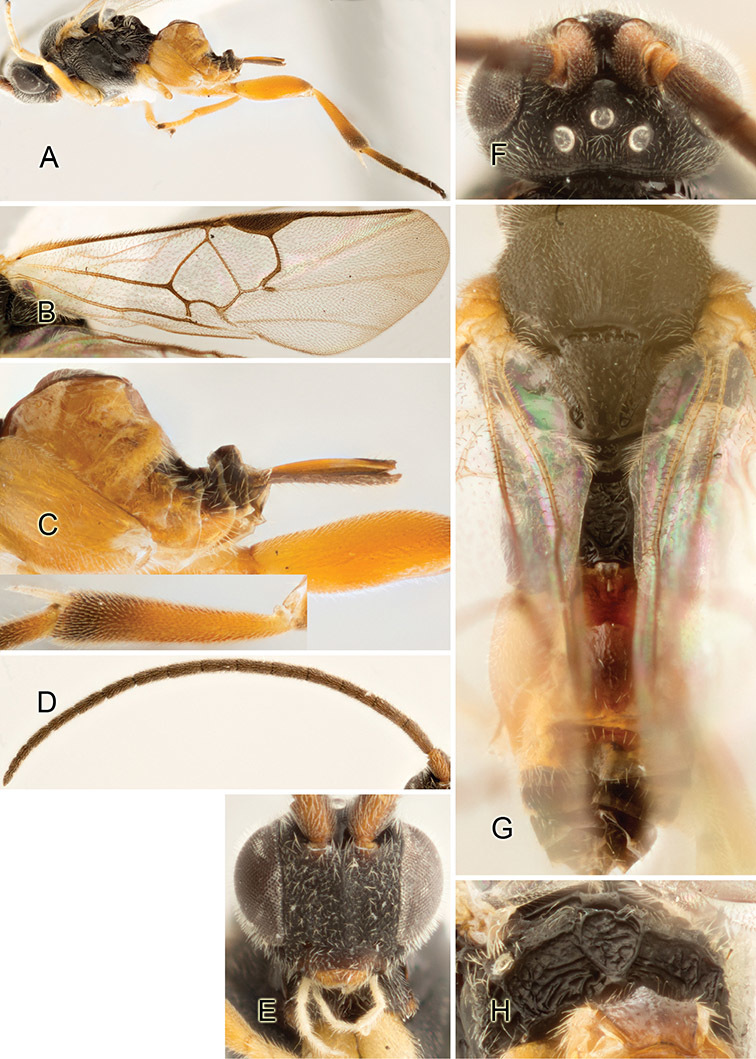
*Apanteles williamcamposi*. **A** Habitus, lateral view **B** Fore wing **C** Hypopygium and ovipositor sheats, with details of metatibia **D** Antenna **E** Head, frontal view **F** Head, dorsal view **G** Meso- and metasoma (partially), dorsal view **H** Propodeum.

**Figure 96.**
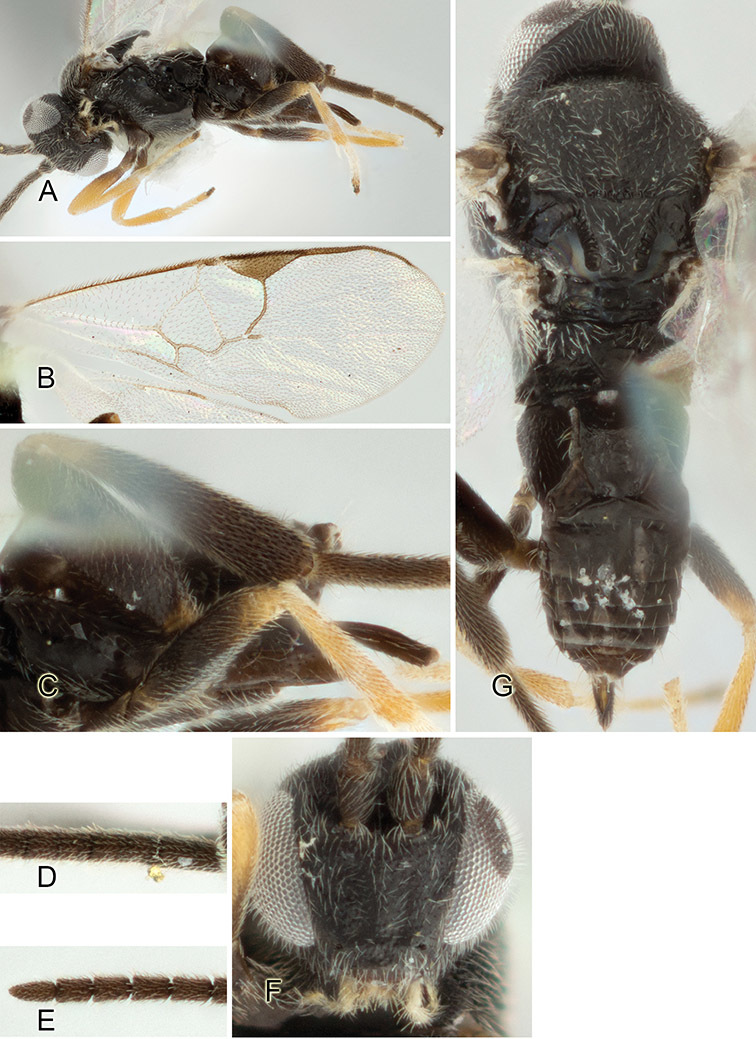
*Apanteles carlosrodriguezi*. **A** Habitus, lateral view **B** Fore wing **C** Hypopygium and ovipositor sheats **D** Anterior half of antenna **E** Posterior half of antenna **F** Head, frontal view **G** Head, meso- and metasoma, dorsal view.

**Figure 97.**
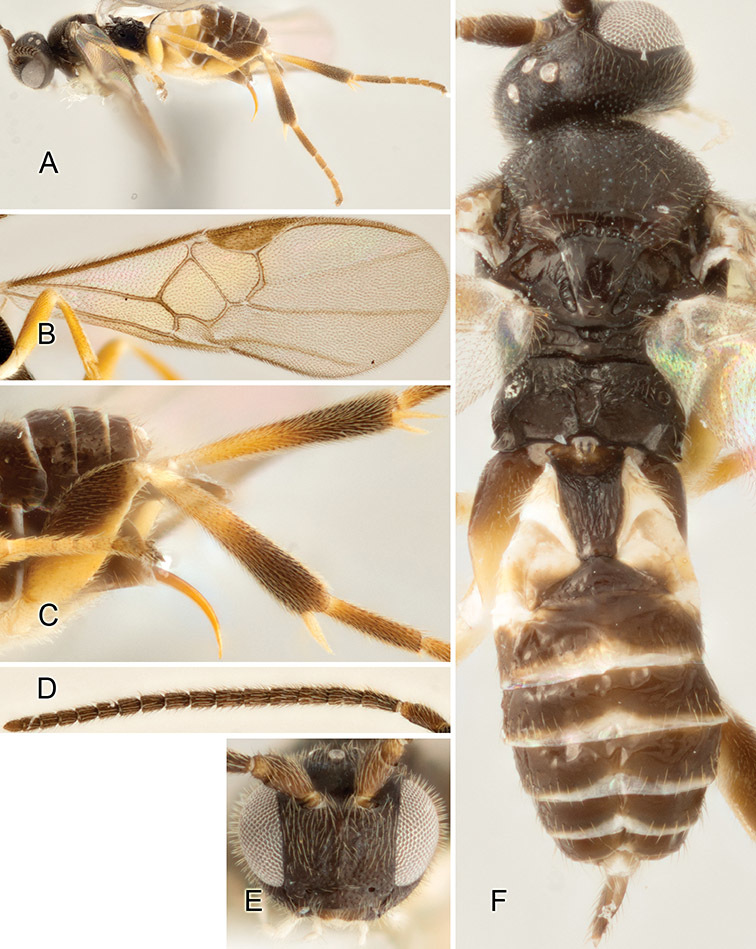
*Apanteles gloriasihezarae*. **A** Habitus, lateral view **B** Fore wing **C** Hypopygium and ovipositor sheats **D** Antenna **E** Head, frontal view **F** Head, meso- and metasoma, dorsal view.

**Figure 98.**
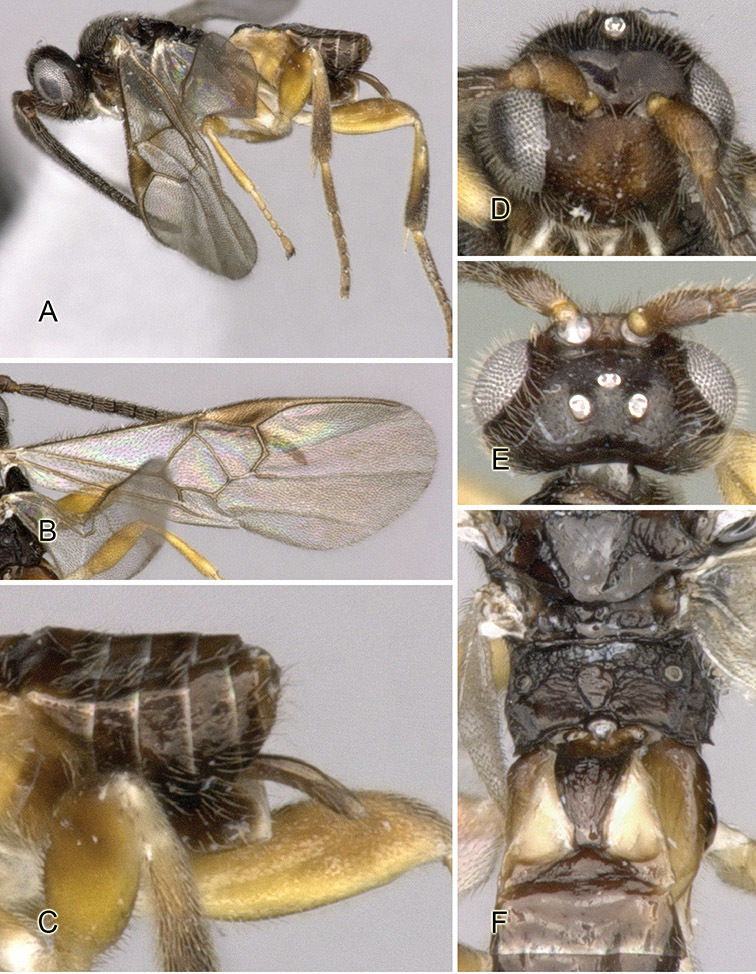
*Apanteles robertoespinozai*. **A** Habitus, lateral view **B** Fore wing **C** Hypopygium and ovipositor sheats **D** Head, frontal view **E** Head, dorsal view **F** Meso- and metasoma (partially), dorsal view.

**Figure 99.**
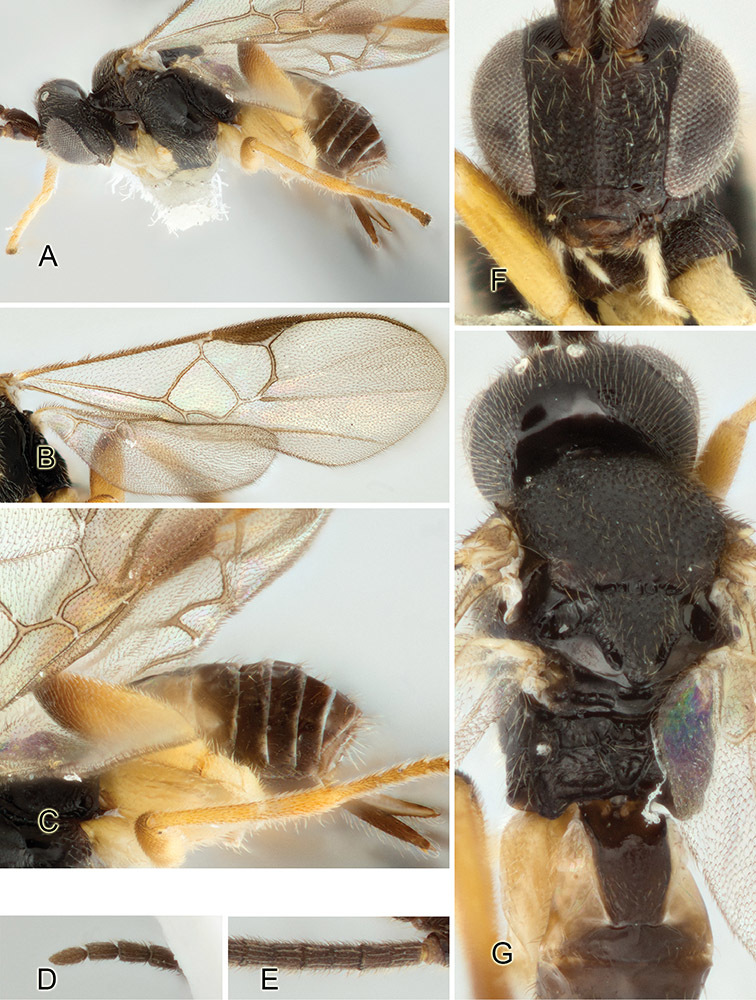
*Apanteles carloszunigai*. **A** Habitus, lateral view **B** Fore wing **C** Hypopygium and ovipositor sheats **D** Posterior half of antenna **E** Anterior half of antenna **F** Head, frontal view **G** Head, meso- and metasoma (partially), dorsal view.

**Figure 100.**
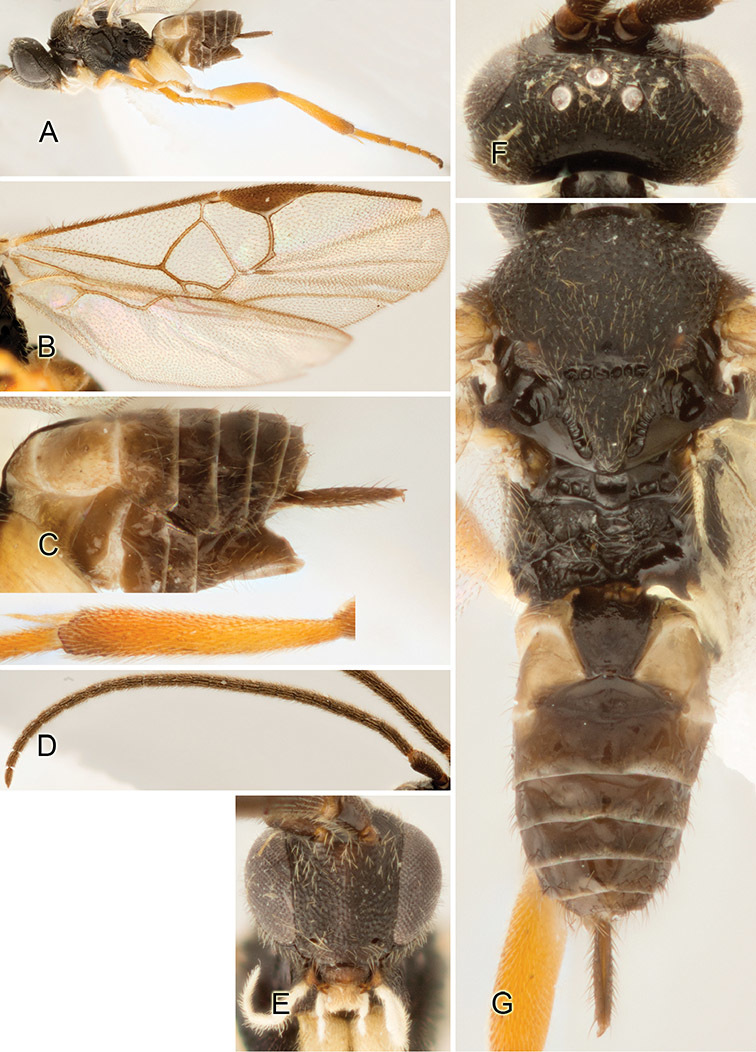
*Apanteles yeissonchavesi*. **A** Habitus, lateral view **B** Fore wing **C** Hypopygium and ovipositor sheats, with details of metatibia **D** Antenna **E** Head, frontal view **F** Head, dorsal view **G** Meso- and metasoma, dorsal view.

**Figure 101.**
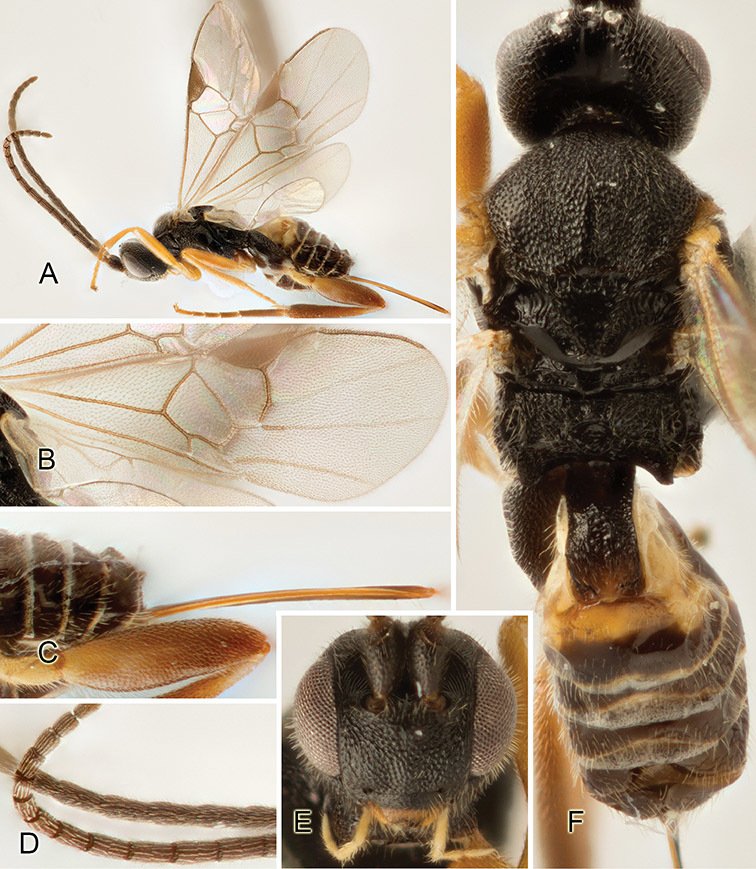
*Apanteles albanjimenezi*. **A** Habitus, lateral view **B** Fore wing **C** Hypopygium and ovipositor sheats **D** Antenna (partially) **E** Head, frontal view **F** Head, meso- and metasoma, dorsal view.

**Figure 102.**
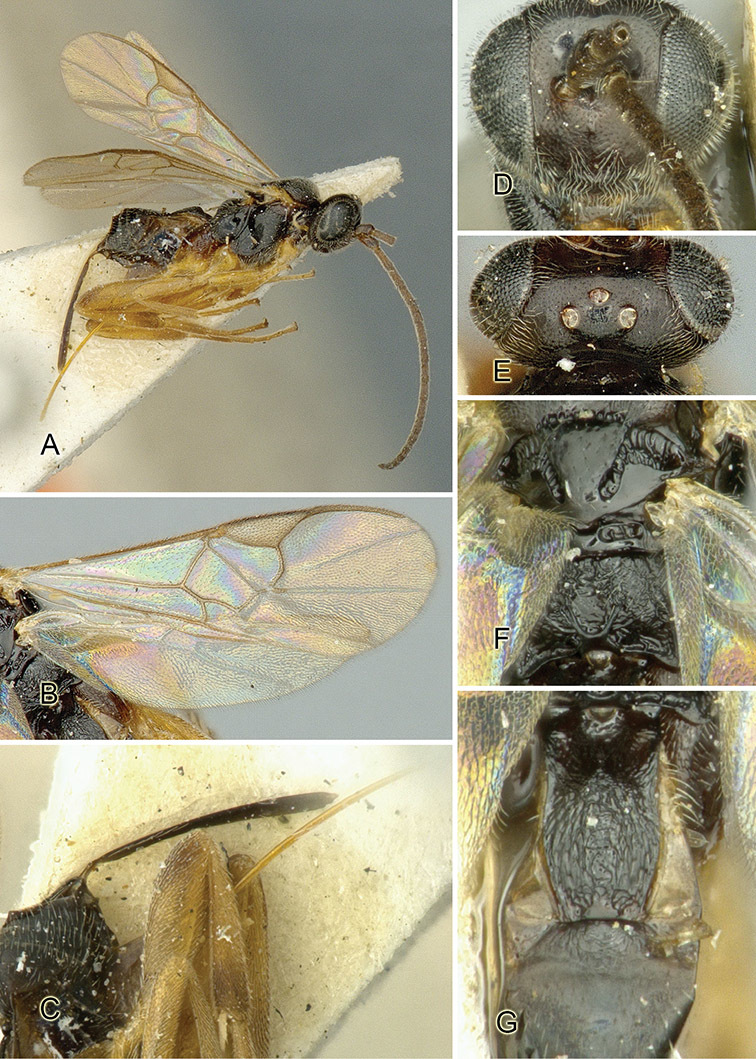
*Apanteles rhomboidalis*. **A** Habitus, lateral view **B** Fore wing **C** Hypopygium and ovipositor sheats **D** Head, frontal view **E** Head, dorsal view **F** Mesosome (partially) **G** Mediotergites 1–3, dorsal view.

**Figure 103.**
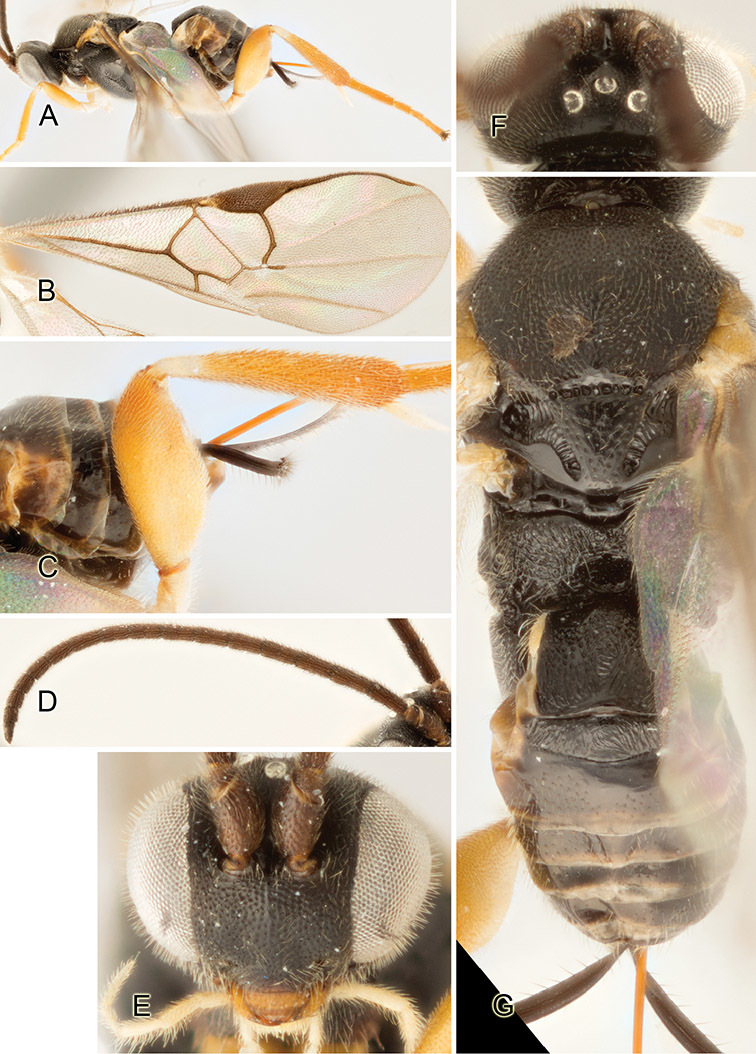
*Apanteles robertovargasi*. **A** Habitus, lateral view **B** Fore wing **C** Hypopygium and ovipositor sheats **D** Antenna **E** Head, frontal view **F** Head, dorsal view **G** Meso- and metasoma, dorsal view.

**Figure 104.**
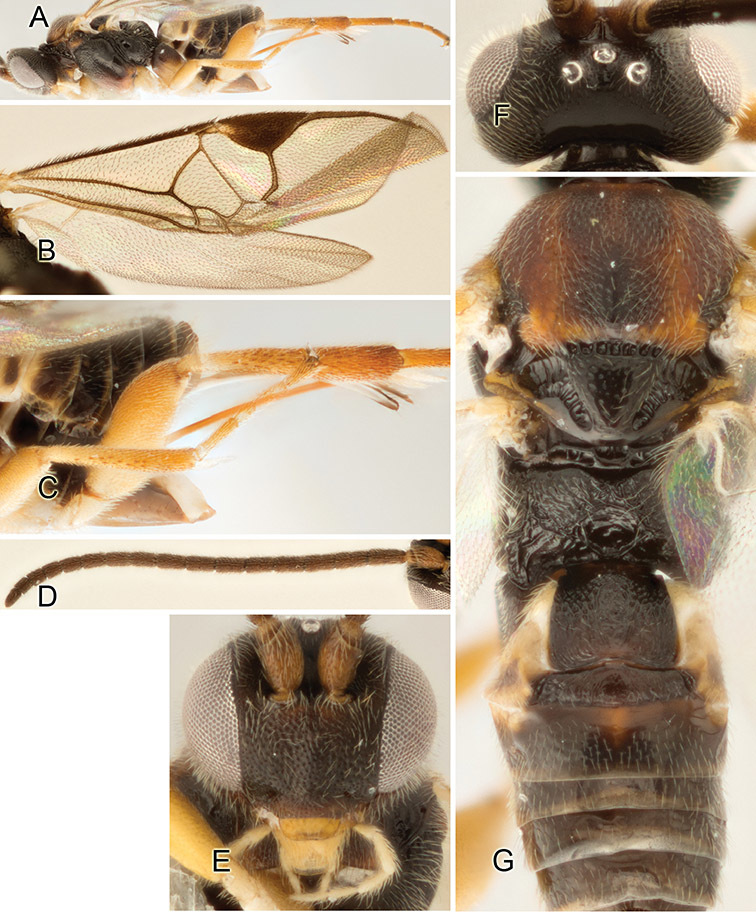
*Apanteles rolandoramosi*. **A** Habitus, lateral view **B** Fore wing **C** Hypopygium and ovipositor sheats **D** Antenna **E** Head, frontal view **F** Head, dorsal view **G** Meso- and metasoma (partially), dorsal view.

**Figure 105.**
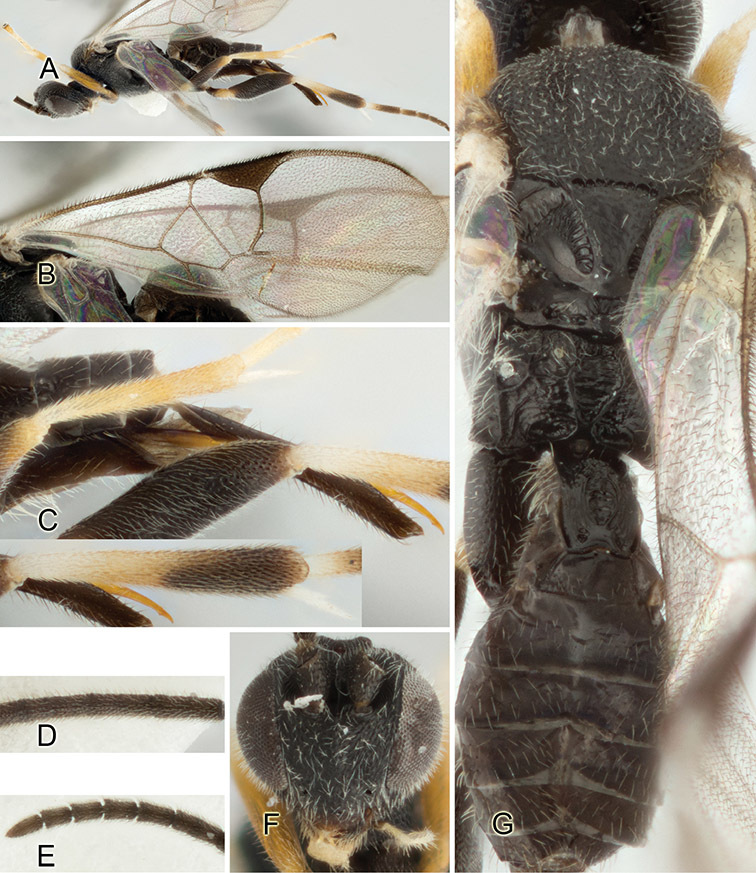
*Apanteles christianzunigai*. **A** Habitus, lateral view **B** Fore wing **C** Hypopygium and ovipositor sheats, with details of metatibia **D** Anterior half of antenna **E** Posterior half of antenna **F** Head, frontal view **G** Meso- and metasoma, dorsal view.

**Figure 106.**
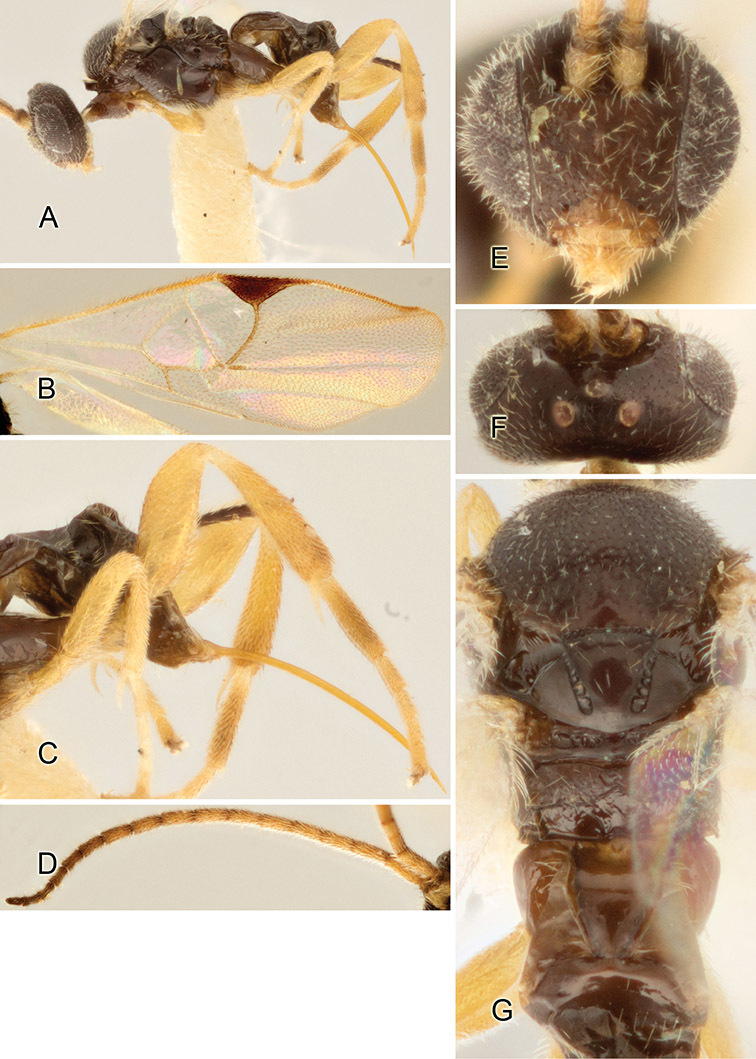
*Apanteles coffeellae*. **A** Habitus, lateral view **B** Fore wing **C** Hypopygium and ovipositor sheats **D** Antenna **E** Head, frontal view **F** Head, dorsal view **G** Meso- and metasoma (partially), dorsal view.

**Figure 107.**
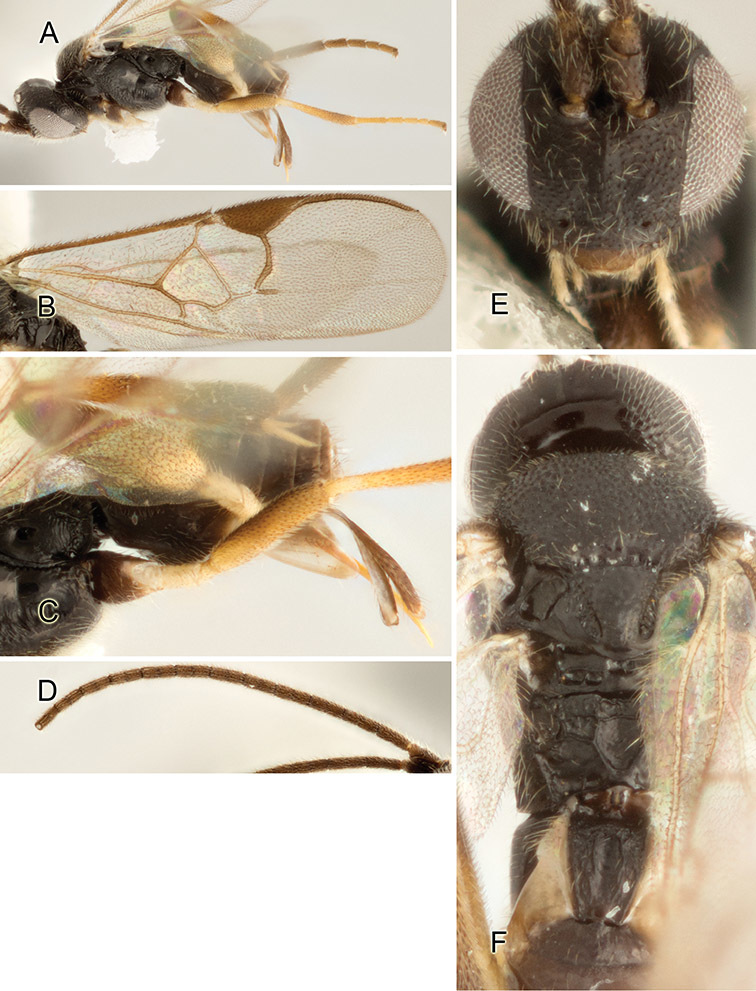
*Apanteles laurahuberae*. **A** Habitus, lateral view **B** Fore wing **C** Hypopygium and ovipositor sheats **D** Antenna **E** Head, frontal view **F** Head, meso- and metasoma (partially), dorsal view.

**Figure 108.**
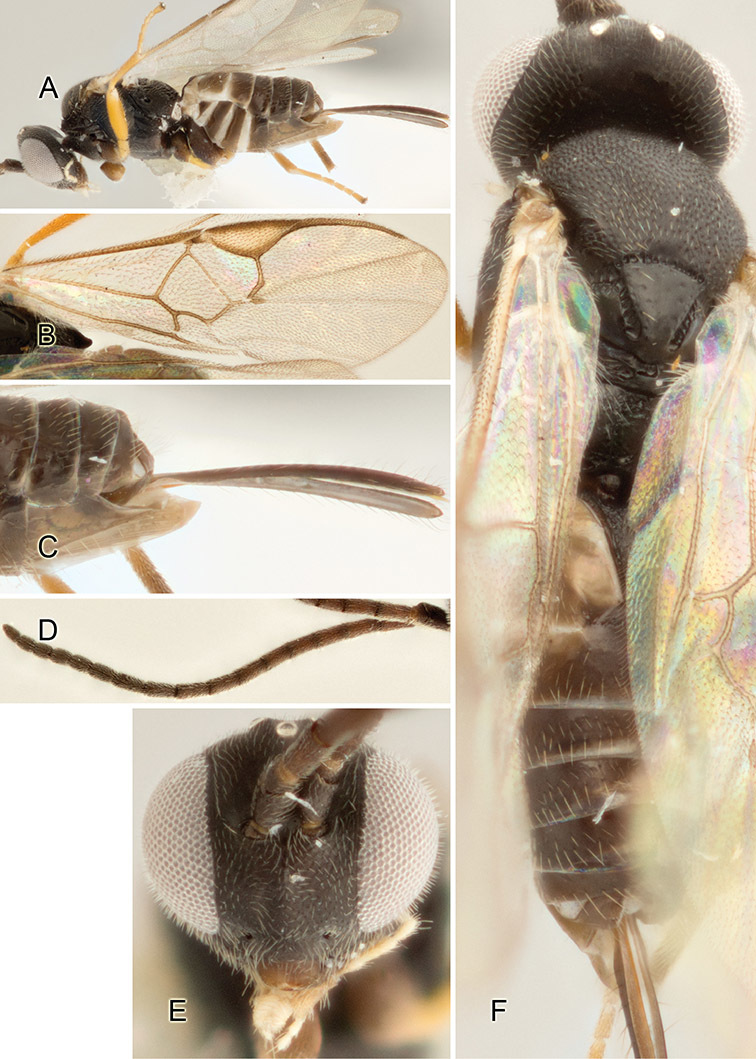
*Apanteles lisabearssae*. **A** Habitus, lateral view **B** Fore wing **C** Hypopygium and ovipositor sheats **D** Antenna **E** Head, frontal view **F** Head, meso- and metasoma, dorsal view.

**Figure 109.**
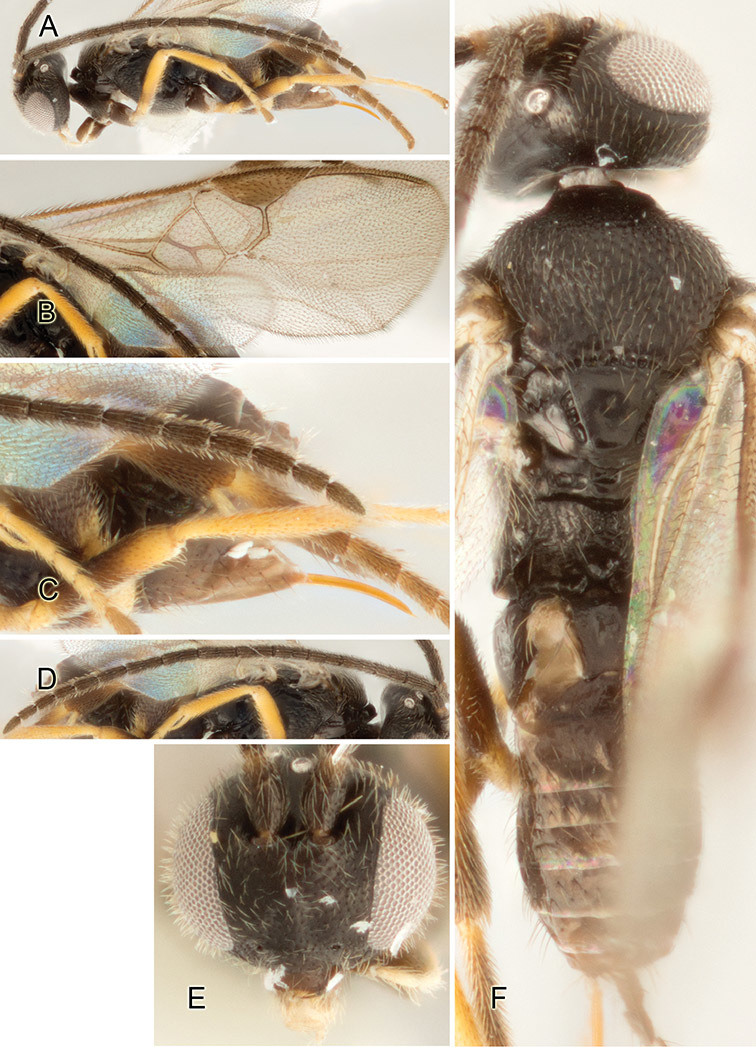
*Apanteles mariaguevarae*. **A** Habitus, lateral view **B** Fore wing **C** Hypopygium and ovipositor sheats **D** Antenna **E** Head, frontal view **F** Head, meso- and metasoma, dorsal view.

**Figure 110.**
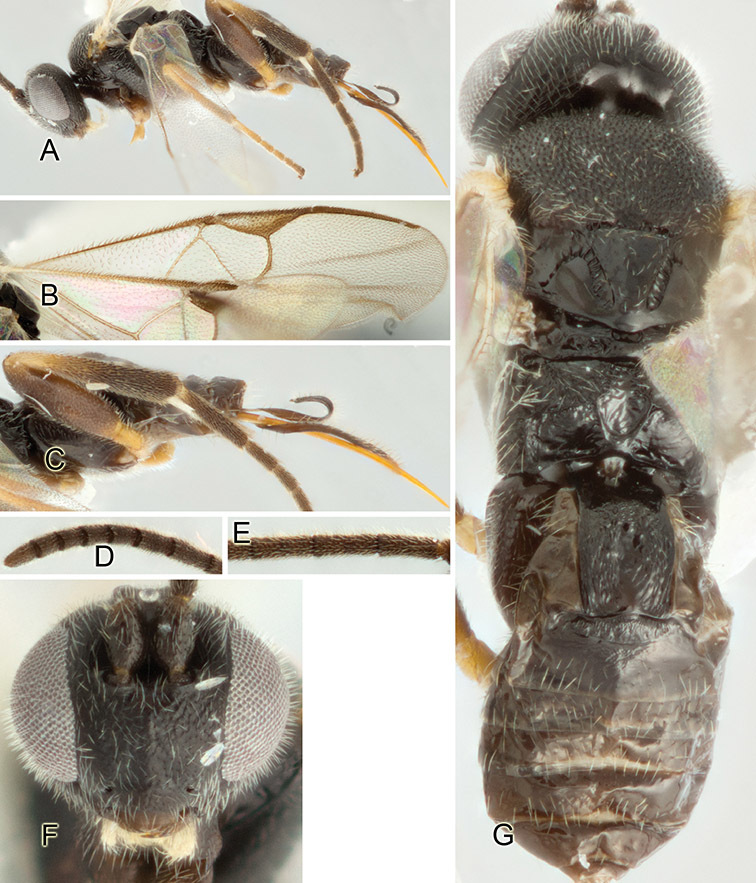
*Apanteles dickyui*. **A** Habitus, lateral view **B** Fore wing **C** Hypopygium and ovipositor sheats **D** Posterior half of antenna **E** Anterior half of antenna **F** Head, frontal view **G** Head, meso- and metasoma, dorsal view.

**Figure 111.**
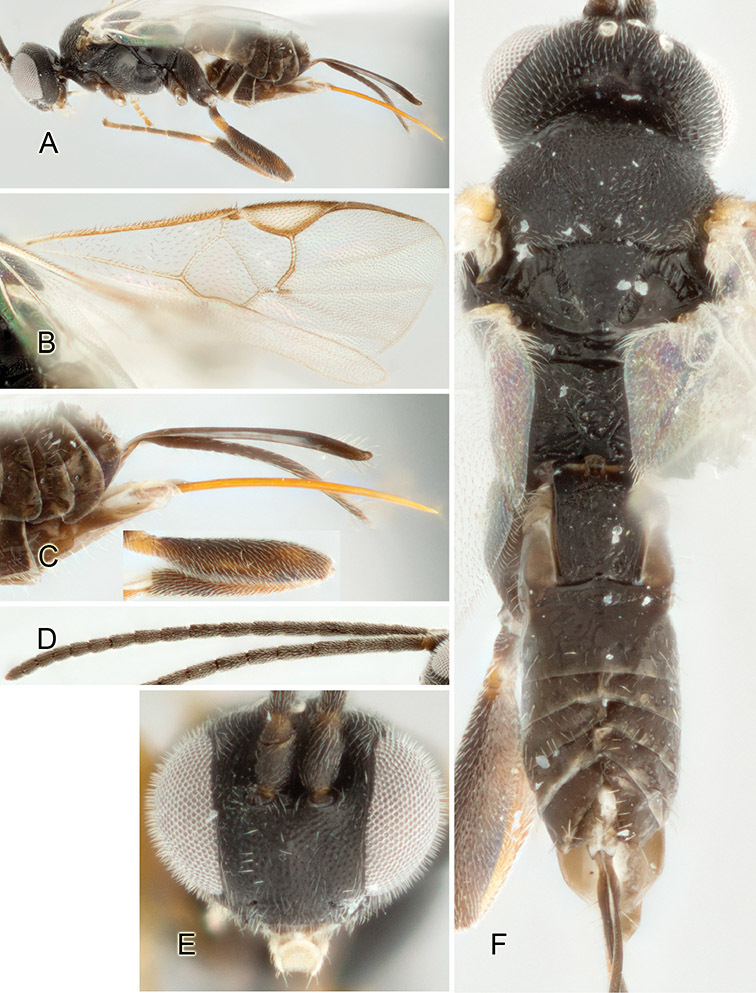
*Apanteles eduardoramirezi*. **A** Habitus, lateral view **B** Fore wing **C** Hypopygium and ovipositor sheats **D** Antenna **E** Head, frontal view **F** Head, meso- and metasoma, dorsal view.

**Figure 112.**
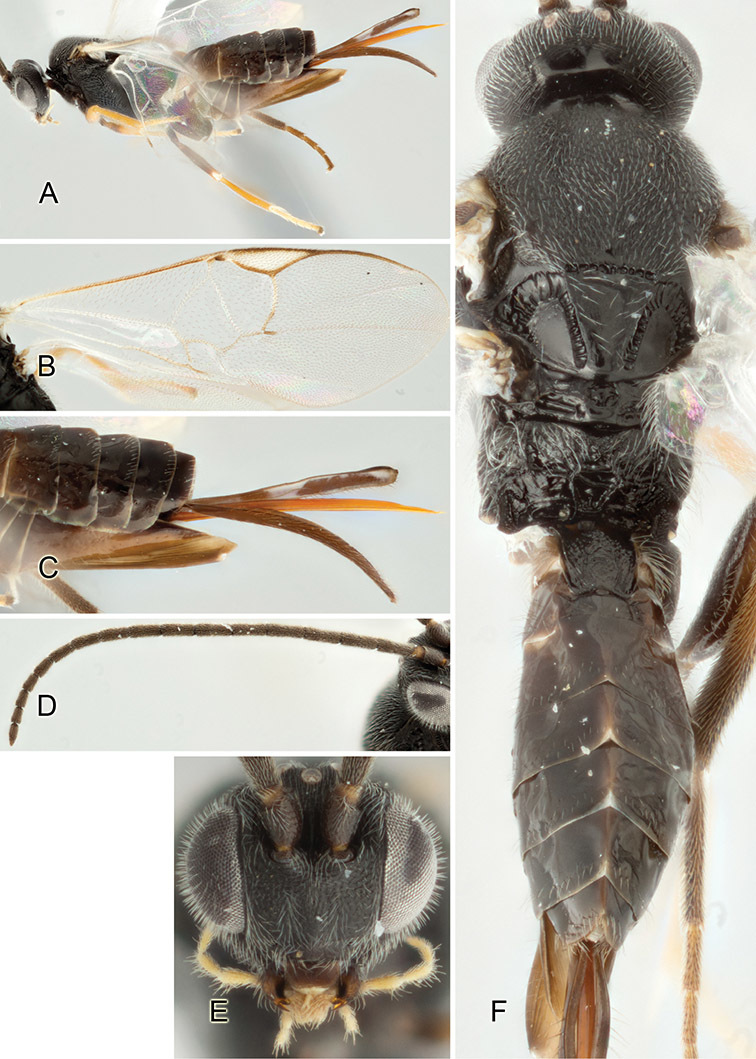
*Apanteles diegotorresi*. **A** Habitus, lateral view **B** Fore wing **C** Hypopygium and ovipositor sheats **D** Antenna **E** Head, frontal view **F** Head, meso- and metasoma, dorsal view.

**Figure 113.**
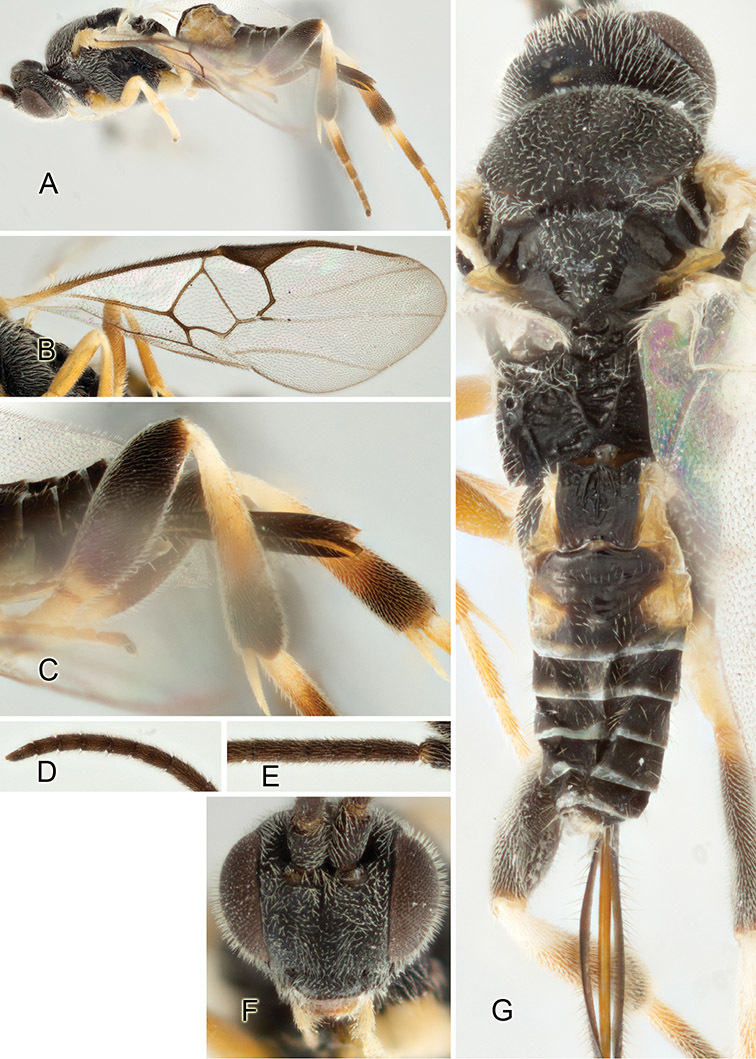
*Apanteles erickduartei*. **A** Habitus, lateral view **B** Fore wing **C** Hypopygium and ovipositor sheats **D** Posterior half of antenna **E** Anterior half of antenna **F** Head, frontal view **G** Head, meso- and metasoma, dorsal view.

**Figure 114.**
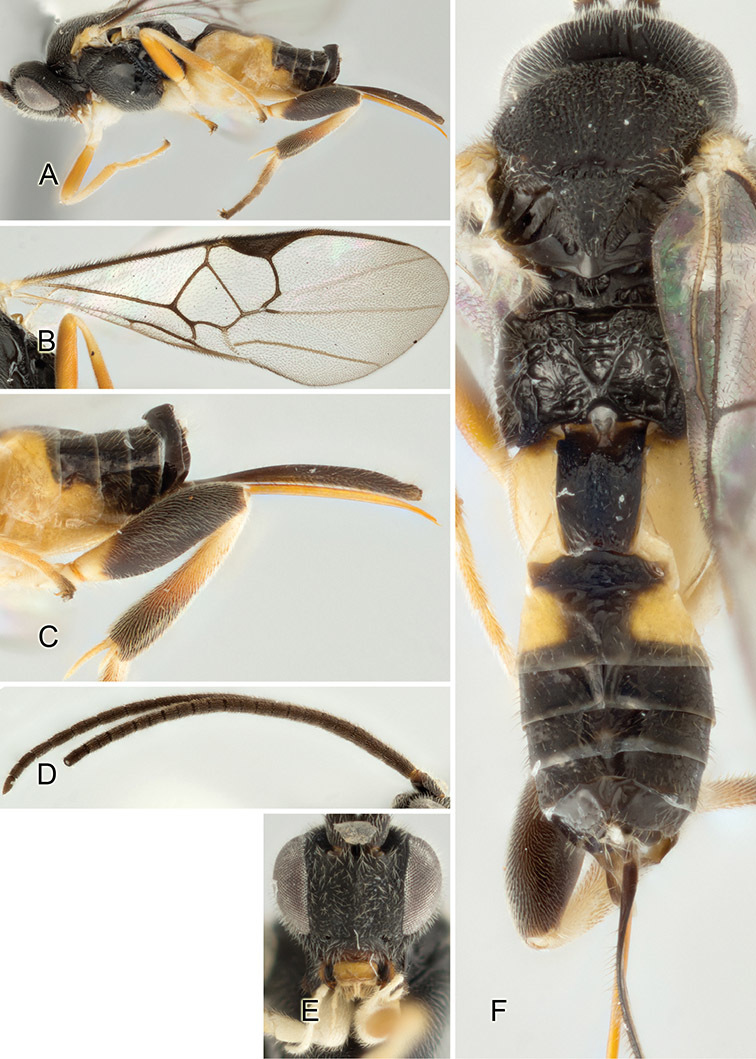
*Apanteles felixcarmonai*. **A** Habitus, lateral view **B** Fore wing **C** Hypopygium and ovipositor sheats **D** Antenna **E** Head, frontal view **F** Head, meso- and metasoma, dorsal view.

**Figure 115.**
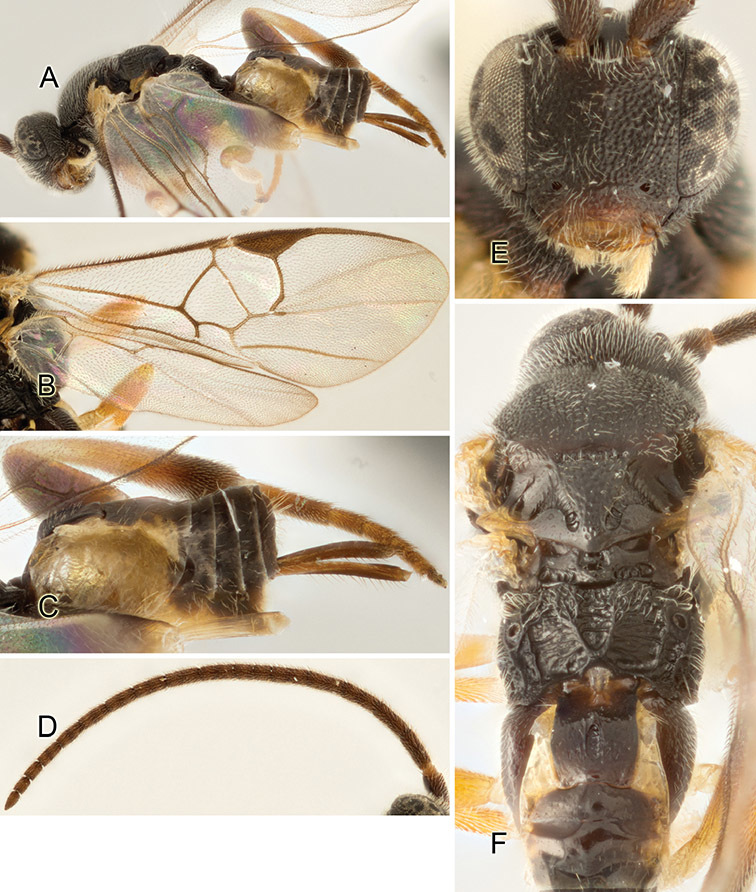
*Apanteles luishernandezi*. **A** Habitus, lateral view **B** Fore wing **C** Hypopygium and ovipositor sheats **D** Antenna **E** Head, frontal view **F** Head, meso- and metasoma (partially), dorsal view.

**Figure 116.**
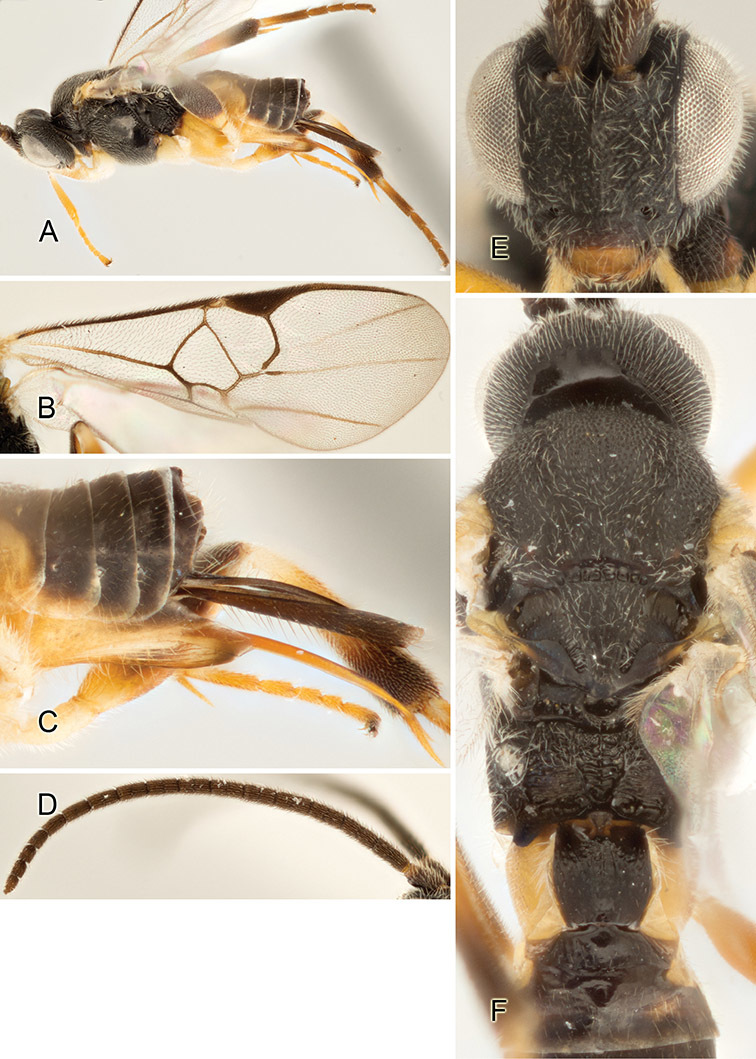
*Apanteles milenagutierrezae*. **A** Habitus, lateral view **B** Fore wing **C** Hypopygium and ovipositor sheats **D** Antenna **E** Head, frontal view **F** Head, meso- and metasoma (partially), dorsal view.

**Figure 117.**
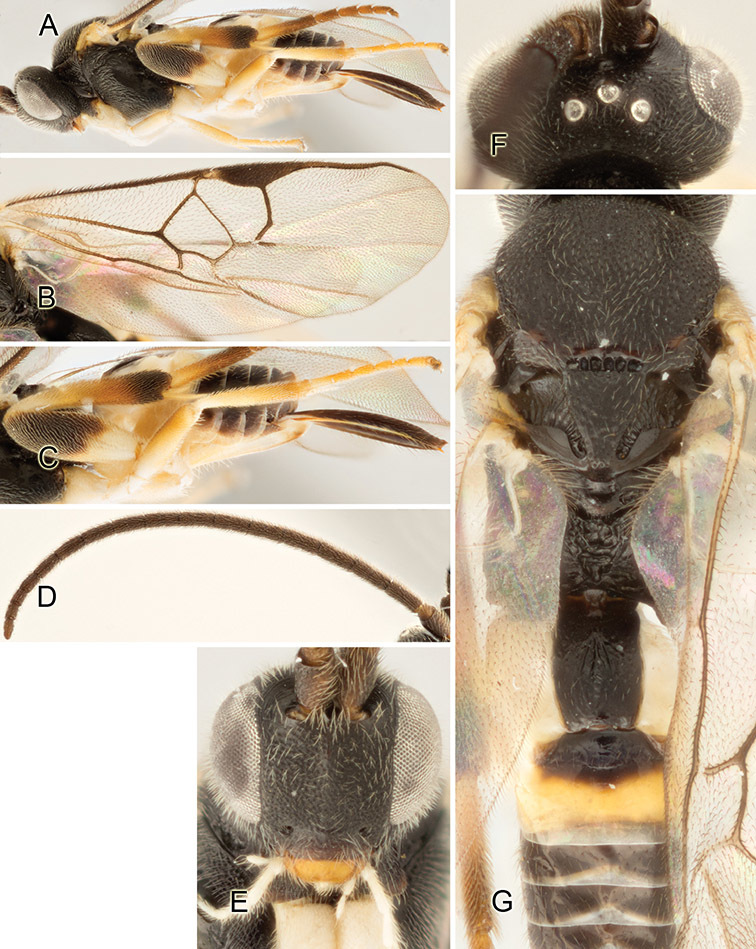
*Apanteles ronaldcastroi*. **A** Habitus, lateral view **B** Fore wing **C** Hypopygium and ovipositor sheats **D** Antenna **E** Head, frontal view **F** Head, dorsal view **G** Meso- and metasoma (partially), dorsal view.

**Figure 118.**
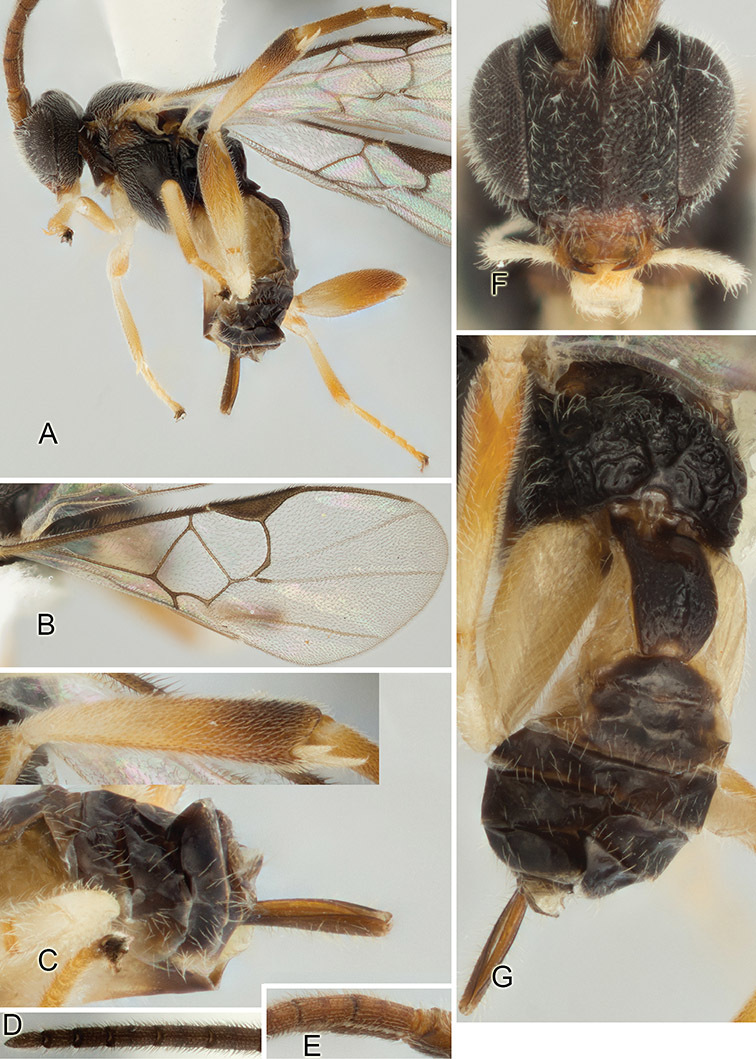
*Apanteles flormoralesae*. **A** Habitus, lateral view **B** Fore wing **C** Hypopygium and ovipositor sheats, with details of metatibia **D** Posterior half of antenna **E** Anterior half of antenna **F** Head, frontal view **G** Meso- and metasoma (partially), dorsal view.

**Figure 119.**
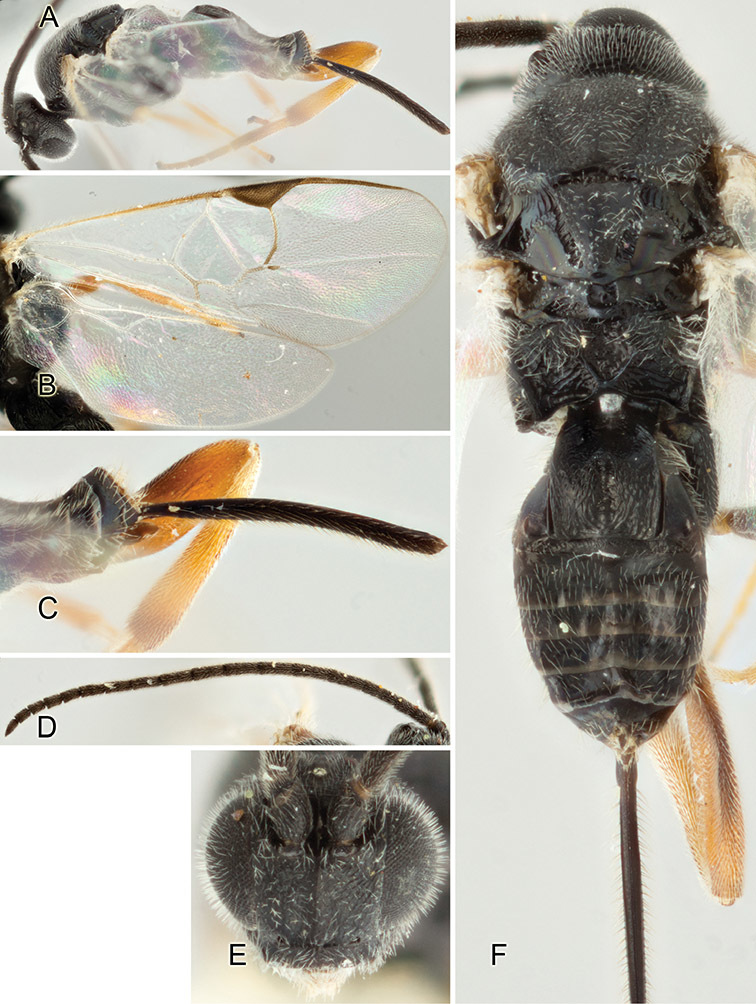
*Apanteles garygibsoni*. **A** Habitus, lateral view **B** Fore wing **C** Hypopygium and ovipositor sheats **D** Antenna **E** Head, frontal view **F** Head, meso- and metasoma, dorsal view.

**Figure 120.**
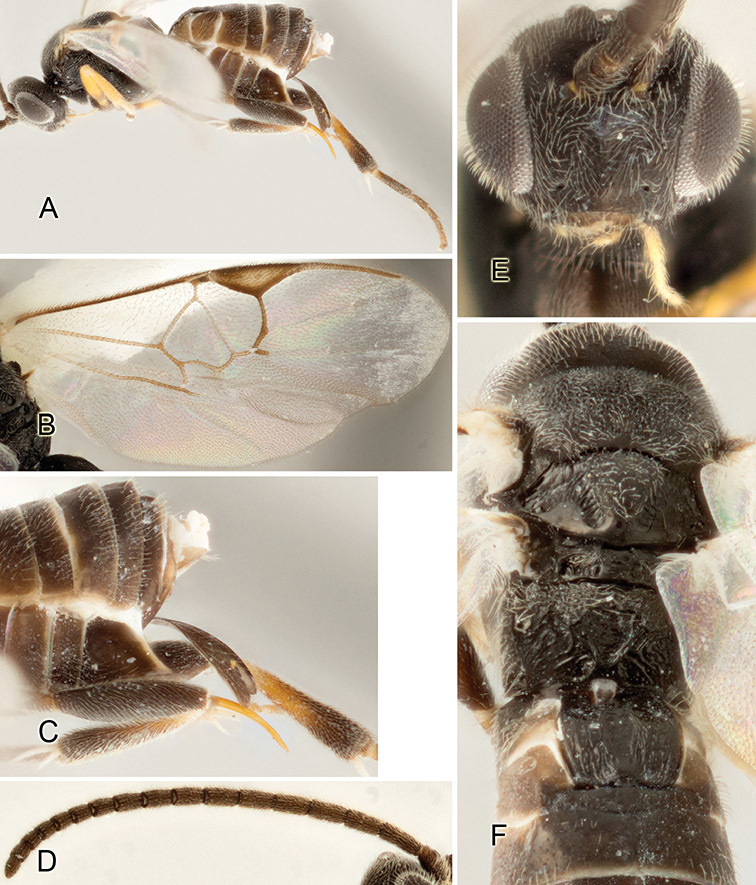
*Apanteles glenriverai*. **A** Habitus, lateral view **B** Fore wing **C** Hypopygium and ovipositor sheats **D** Antenna **E** Head, frontal view **F** Head, meso- and metasoma (partially), dorsal view.

**Figure 121.**
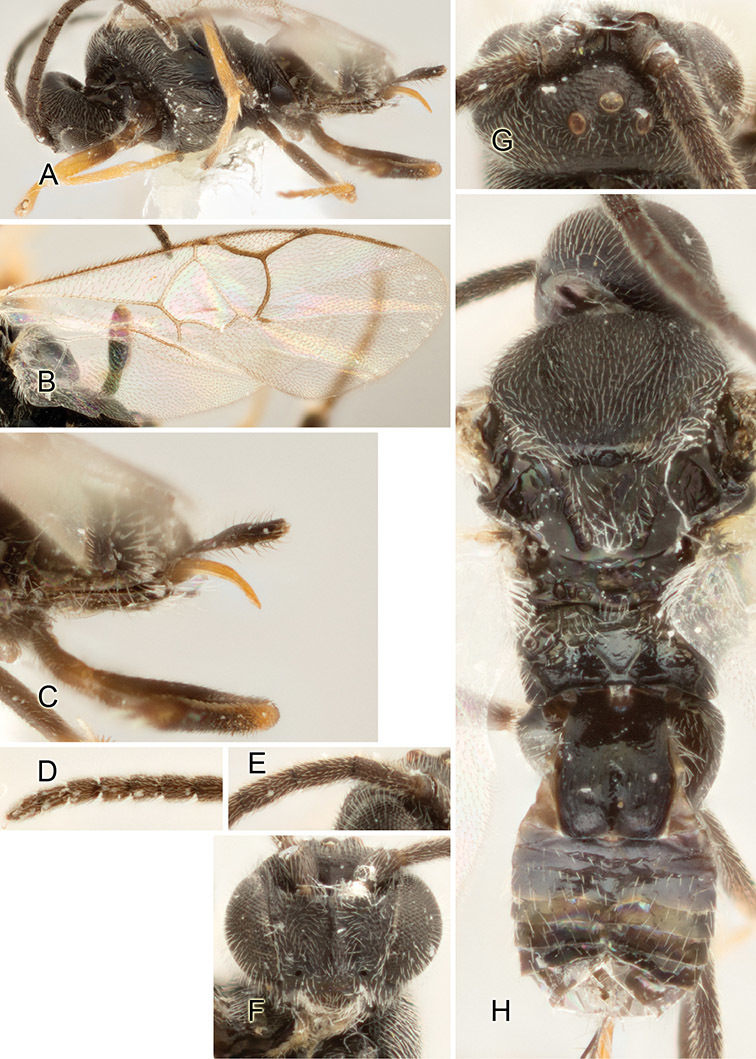
*Apanteles pablovasquezi*. **A** Habitus, lateral view **B** Fore wing **C** Hypopygium and ovipositor sheats **D** Posterior half of antenna **E** Anterior half of antenna **F** Head, frontal view **G** Head, dorsal view **H** Head, meso- and metasoma, dorsal view.

**Figure 122.**
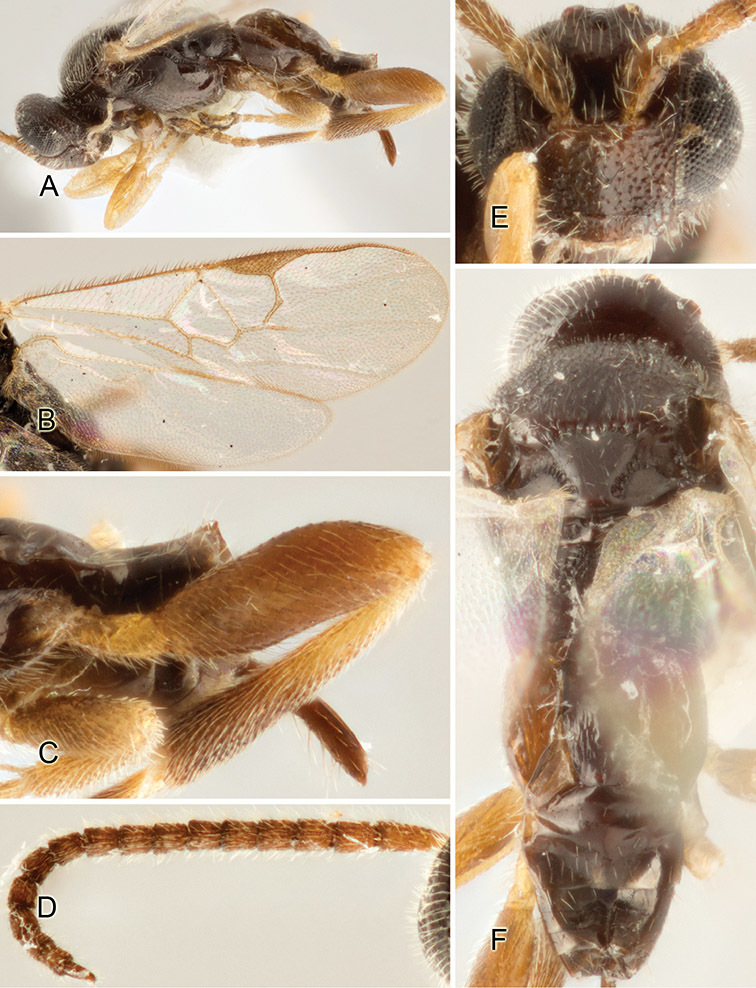
*Apanteles guadaluperodriguezae*. **A** Habitus, lateral view **B** Fore wing **C** Hypopygium and ovipositor sheats **D** Antenna **E** Head, frontal view **F** Head, meso- and metasoma, dorsal view.

**Figure 123.**
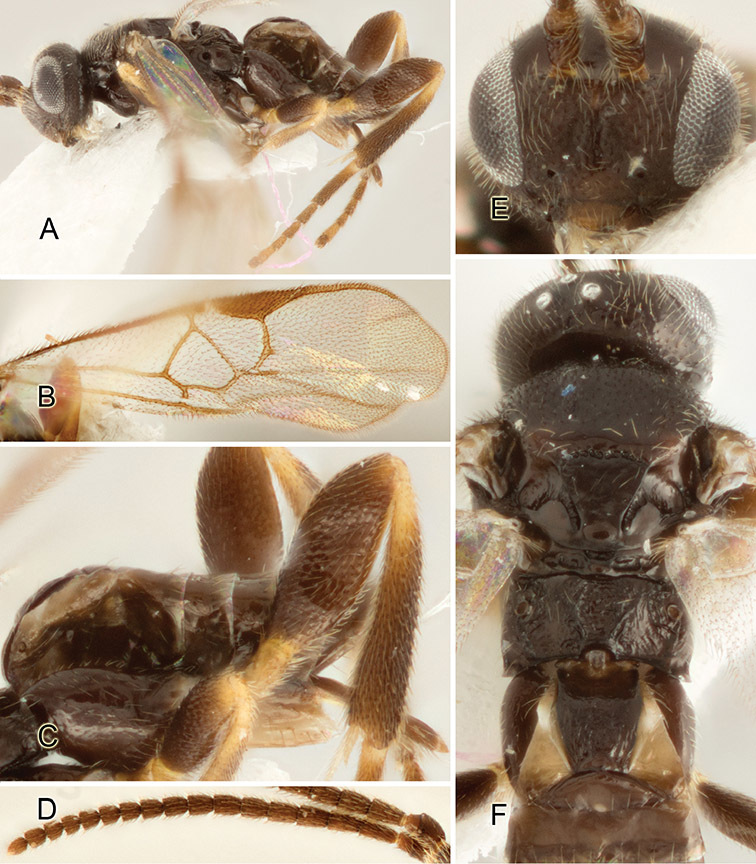
*Apanteles marcobustosi*. **A** Habitus, lateral view **B** Fore wing **C** Hypopygium and ovipositor sheats **D** Antenna **E** Head, frontal view **F** Head, meso- and metasoma (partially), dorsal view.

**Figure 124.**
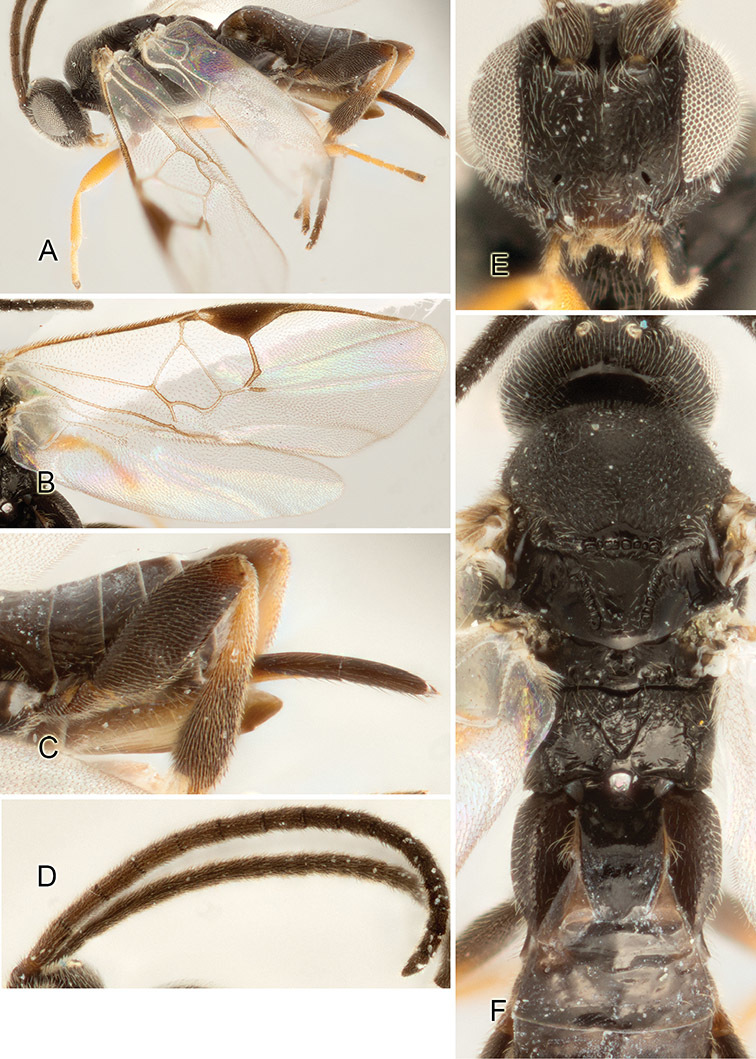
*Apanteles hectorsolisi*. **A** Habitus, lateral view **B** Fore wing **C** Hypopygium and ovipositor sheats **D** Antenna **E** Head, frontal view **F** Head, meso- and metasoma (partially), dorsal view.

**Figure 125.**
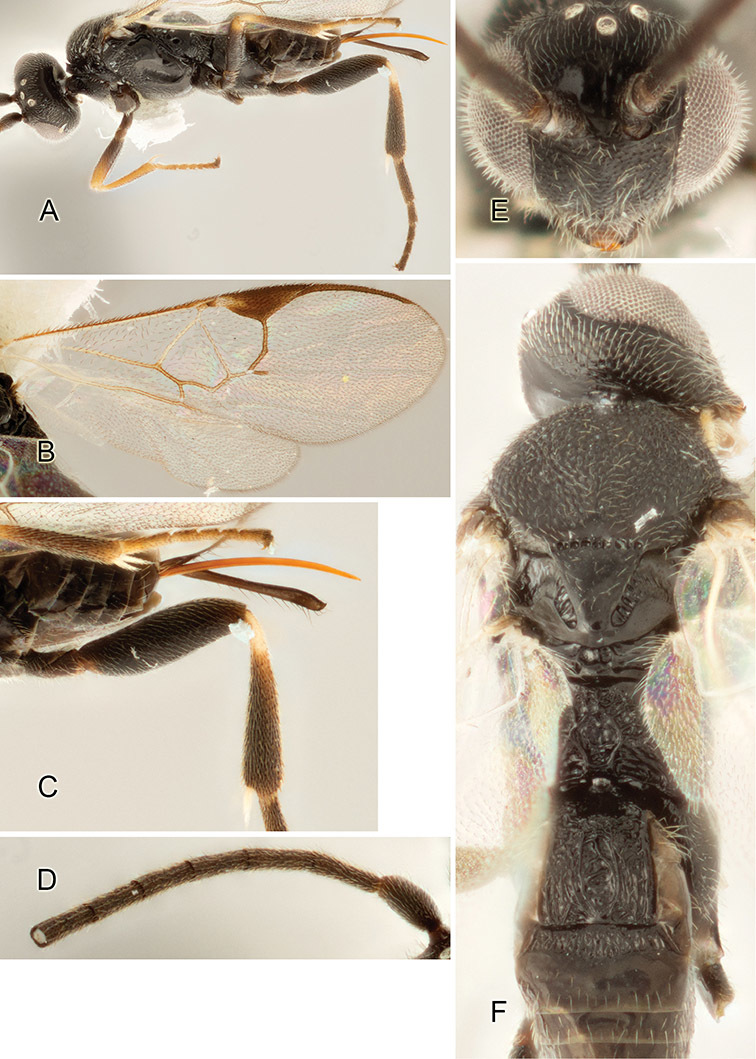
*Apanteles humbertolopezi*. **A** Habitus, lateral view **B** Fore wing **C** Hypopygium and ovipositor sheats **D** Antenna **E** Head, frontal view **F** Head, meso- and metasoma (partially), dorsal view.

**Figure 126.**
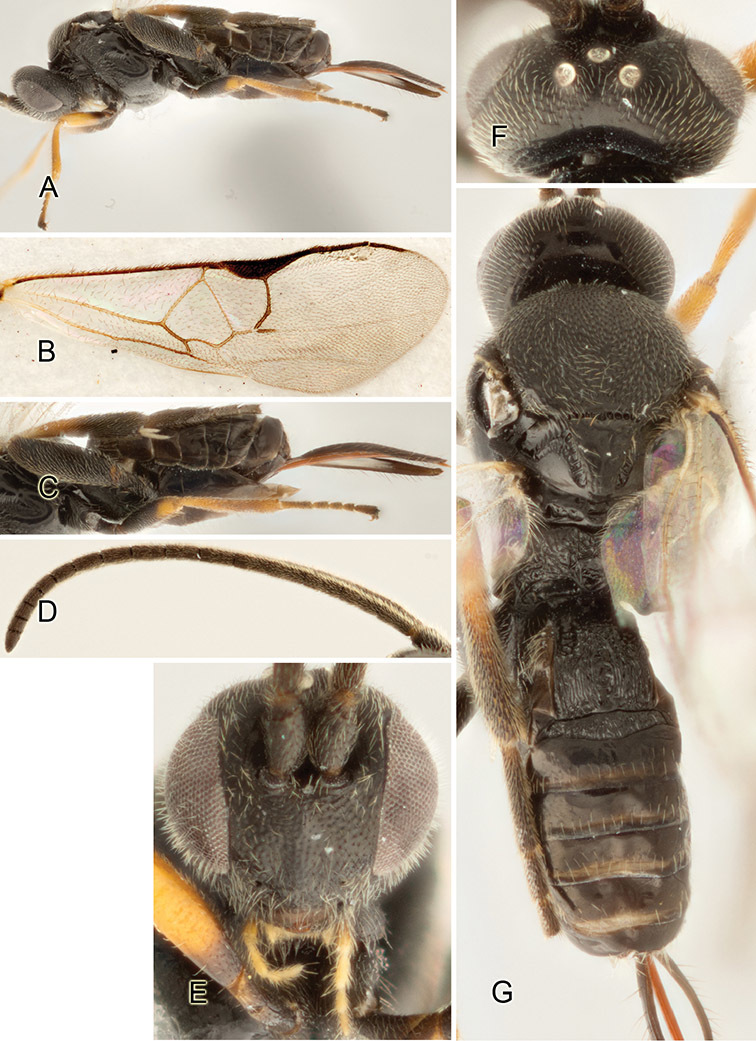
*Apanteles pablotranai*. **A** Habitus, lateral view **B** Fore wing **C** Hypopygium and ovipositor sheats **D** Antenna **E** Head, frontal view **F** Head, dorsal view **G** Head, meso- and metasoma, dorsal view.

**Figure 127.**
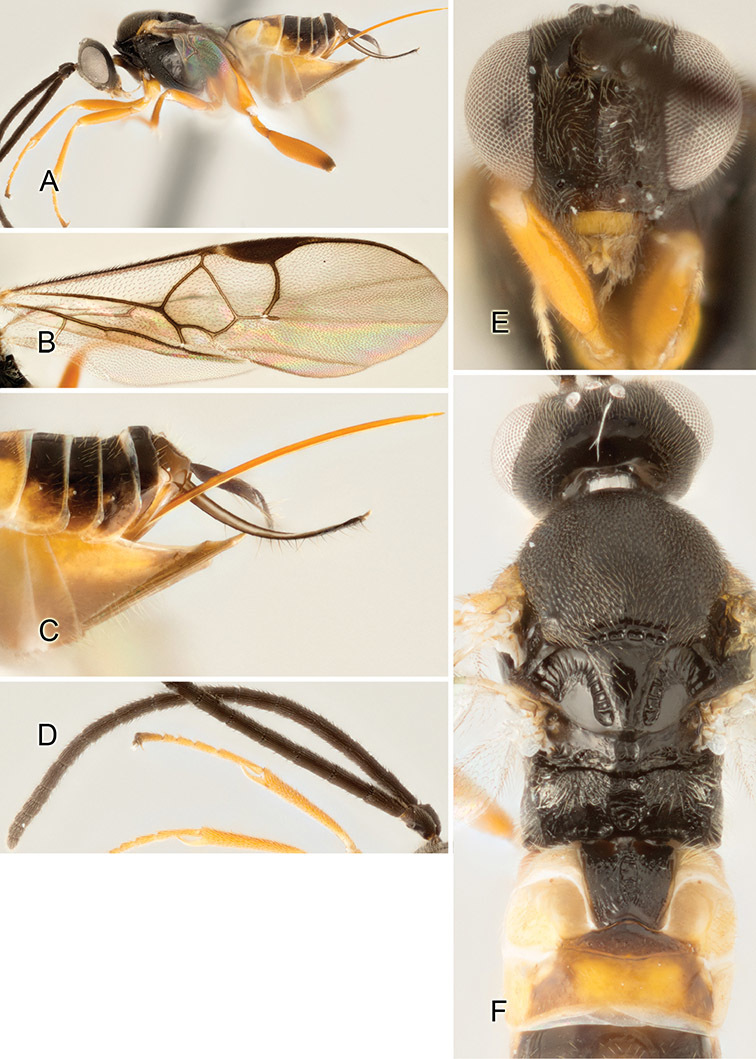
*Apanteles isidrochaconi*. **A** Habitus, lateral view **B** Fore wing **C** Hypopygium and ovipositor sheats **D** Antenna **E** Head, frontal view **F** Head, meso- and metasoma (partially), dorsal view.

**Figure 128.**
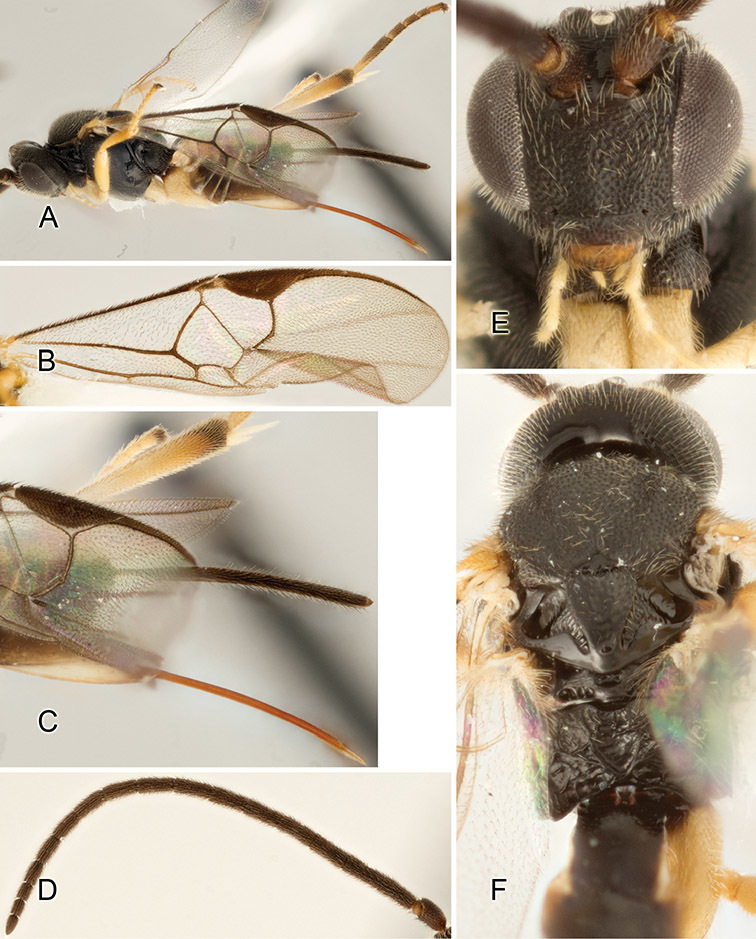
*Apanteles juanapui*. **A** Habitus, lateral view **B** Fore wing **C** Hypopygium and ovipositor sheats **D** Antenna **E** Head, frontal view **F** Head, meso- and metasoma (partially), dorsal view.

**Figure 129.**
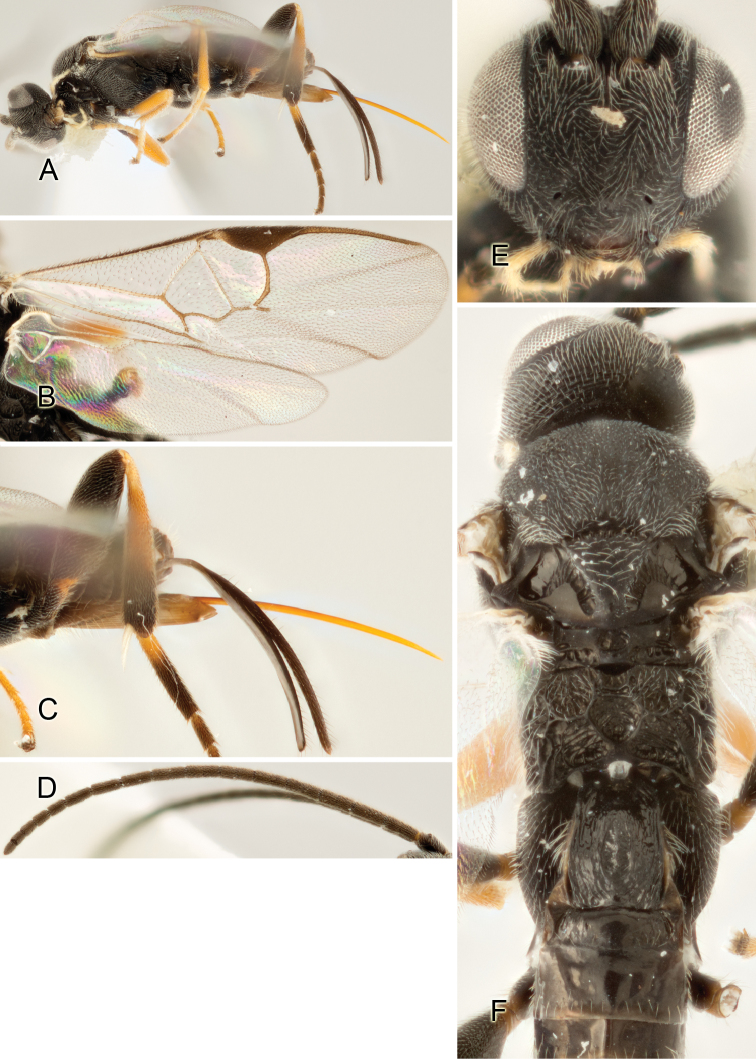
*Apanteles isidrovillegasi*. **A** Habitus, lateral view **B** Fore wing **C** Hypopygium and ovipositor sheats **D** Antenna **E** Head, frontal view **F** Head, meso- and metasoma (partially), dorsal view.

**Figure 130.**
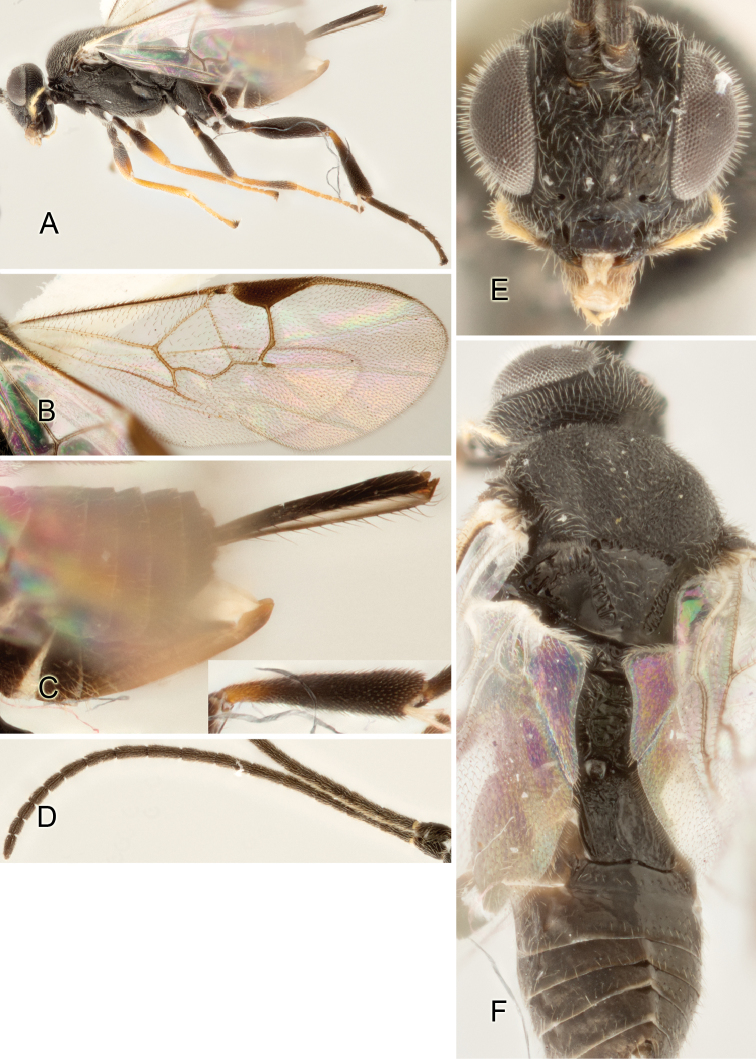
*Apanteles javierobandoi*. **A** Habitus, lateral view **B** Fore wing **C** Hypopygium and ovipositor sheats **D** Antenna **E** Head, frontal view **F** Head, meso- and metasoma (partially), dorsal view.

**Figure 131.**
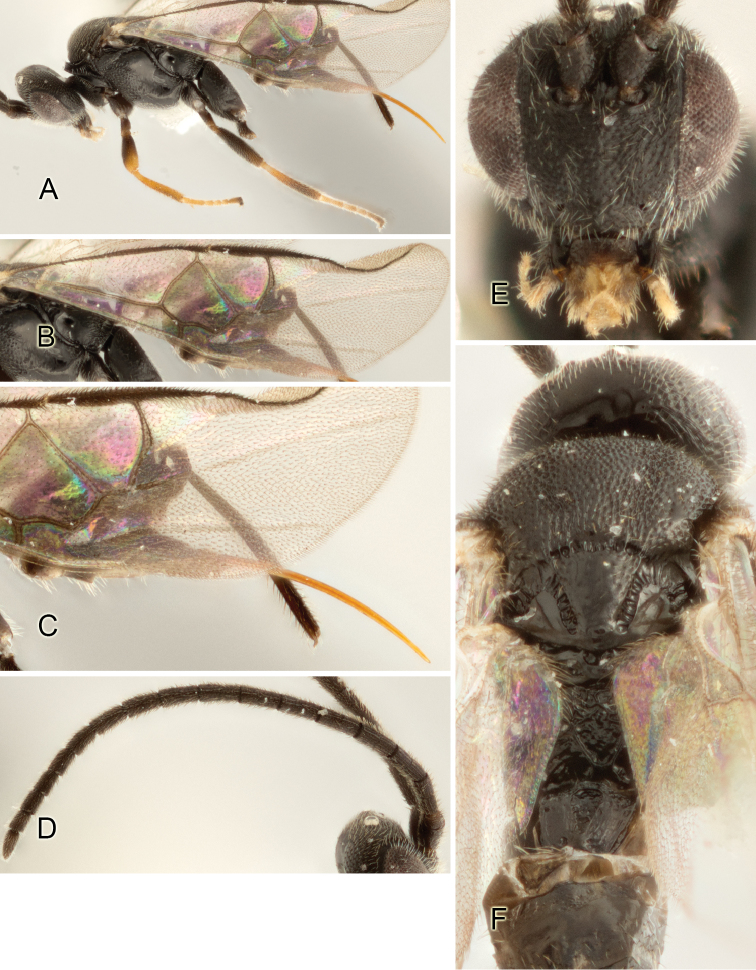
*Apanteles juangazoi*. **A** Habitus, lateral view **B** Fore wing **C** Hypopygium and ovipositor sheats **D** Antenna **E** Head, frontal view **F** Head, meso- and metasoma (partially), dorsal view.

**Figure 132.**
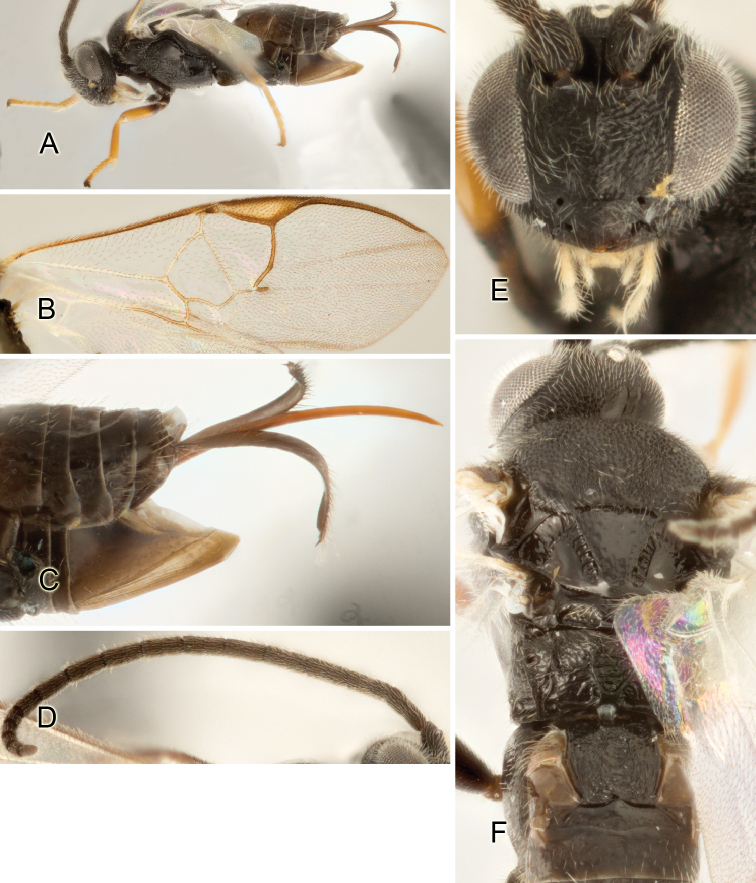
*Apanteles josediazi*. **A** Habitus, lateral view **B** Fore wing **C** Hypopygium and ovipositor sheats **D** Antenna **E** Head, frontal view **F** Head, meso- and metasoma (partially), dorsal view.

**Figure 133.**
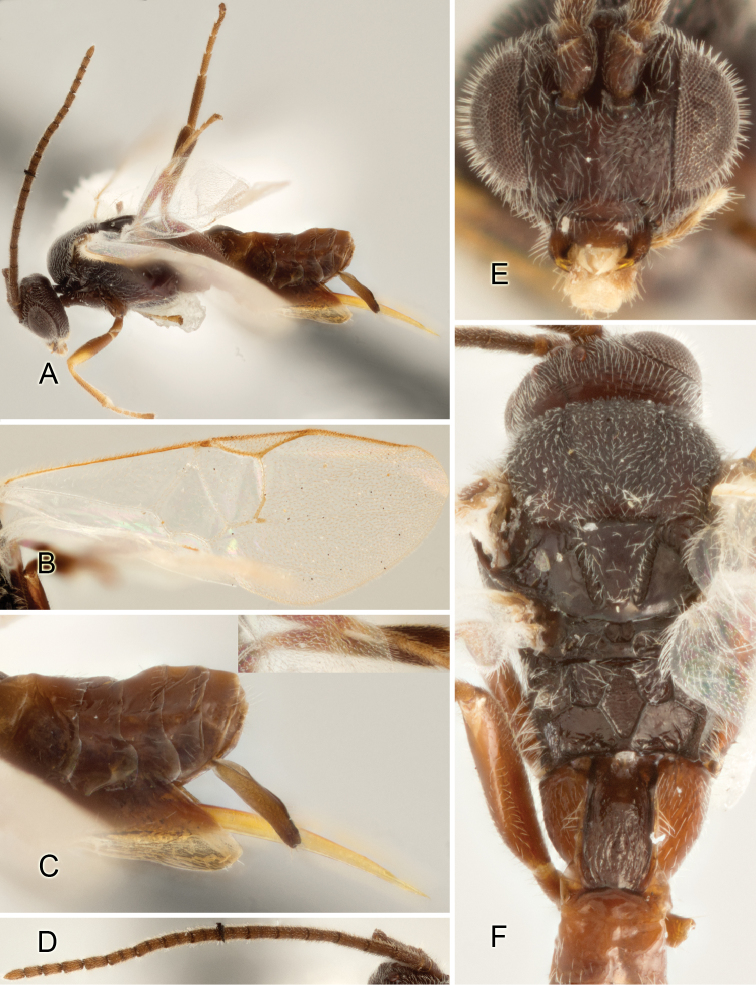
*Apanteles joserasi*. **A** Habitus, lateral view **B** Fore wing **C** Hypopygium and ovipositor sheats **D** Antenna **E** Head, frontal view **F** Head, meso- and metasoma (partially), dorsal view.

**Figure 134.**
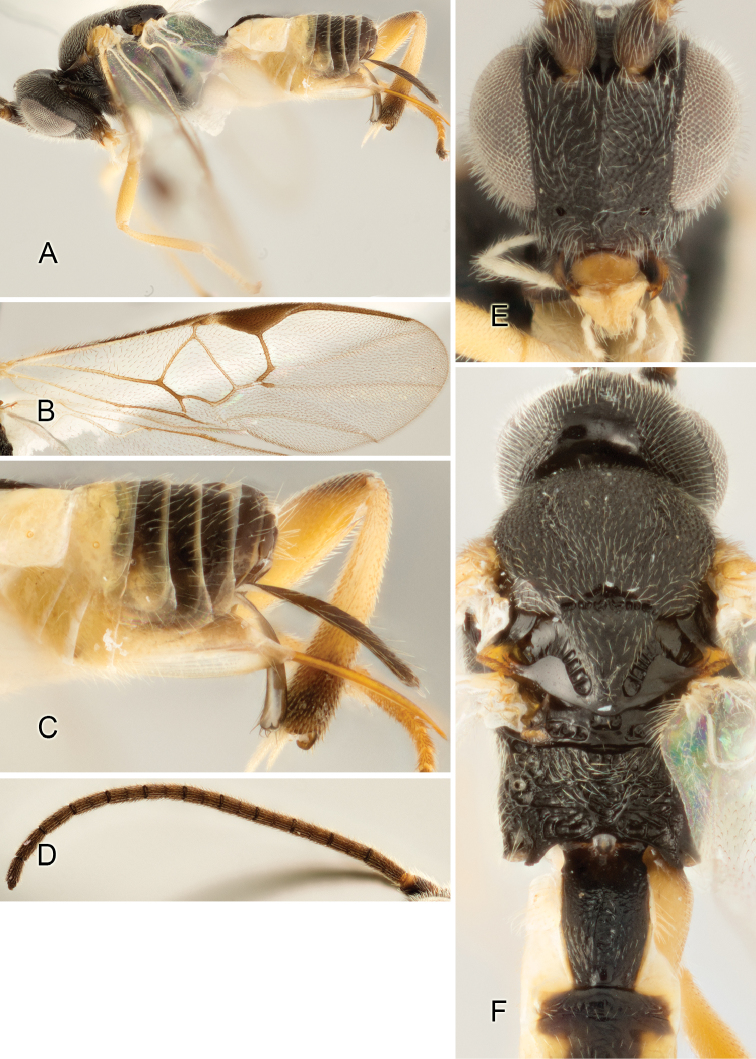
*Apanteles juanhernandezi*. **A** Habitus, lateral view **B** Fore wing **C** Hypopygium and ovipositor sheats **D** Antenna **E** Head, frontal view **F** Head, meso- and metasoma (partially), dorsal view.

**Figure 135.**
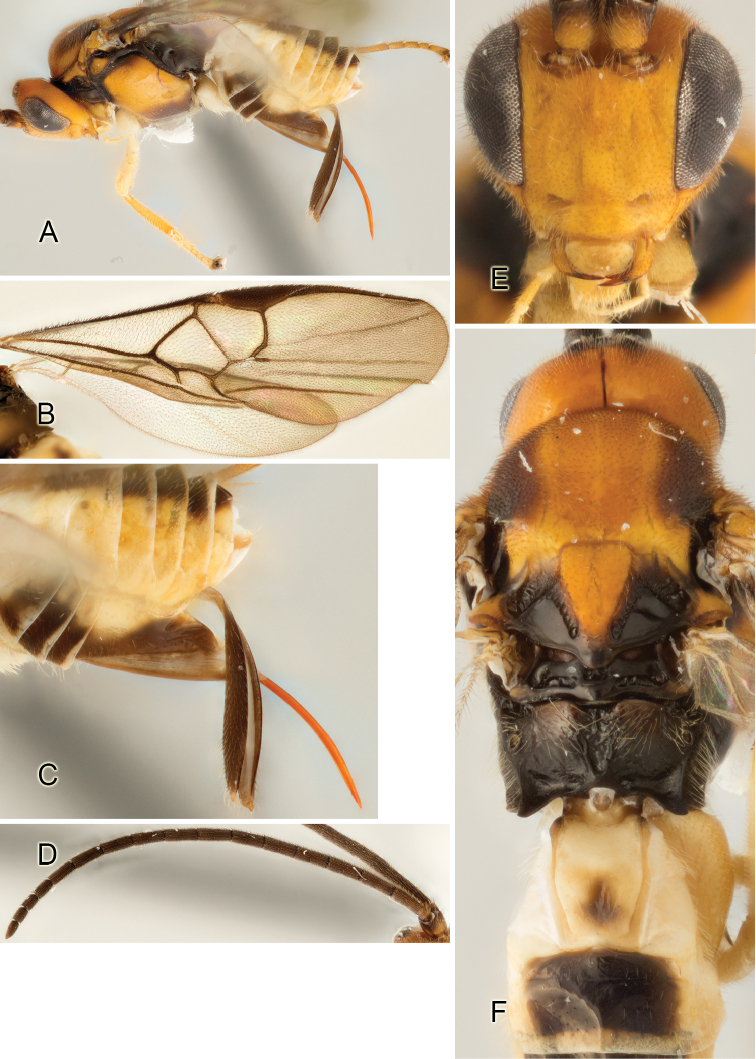
*Apanteles juliodiazi*. **A** Habitus, lateral view **B** Fore wing **C** Hypopygium and ovipositor sheats **D** Antenna **E** Head, frontal view **F** Head, meso- and metasoma (partially), dorsal view.

**Figure 136.**
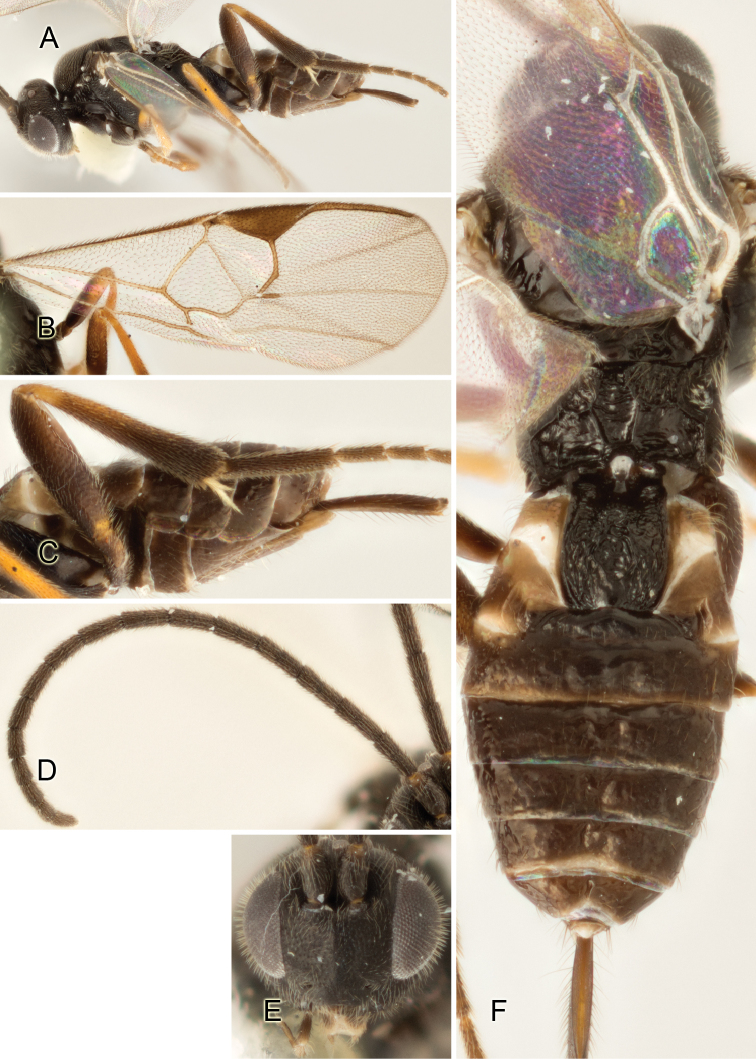
*Apanteles keineraragoni*. **A** Habitus, lateral view **B** Fore wing **C** Hypopygium and ovipositor sheats **D** Antenna **E** Head, frontal view **F** Head, meso- and metasoma, dorsal view.

**Figure 137.**
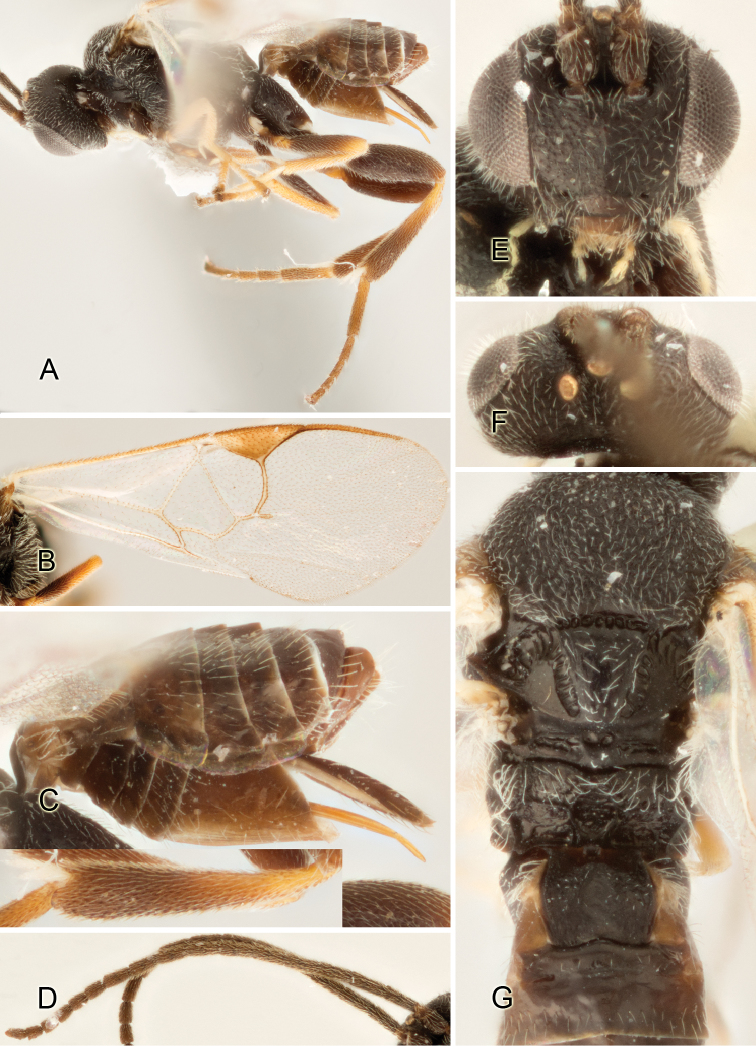
*Apanteles ronaldnavarroi*. **A** Habitus, lateral view **B** Fore wing **C** Hypopygium and ovipositor sheats **D** Antenna **E** Head, frontal view **F** Head, dorsal view **G** Meso- and metasoma (partially), dorsal view.

**Figure 138.**
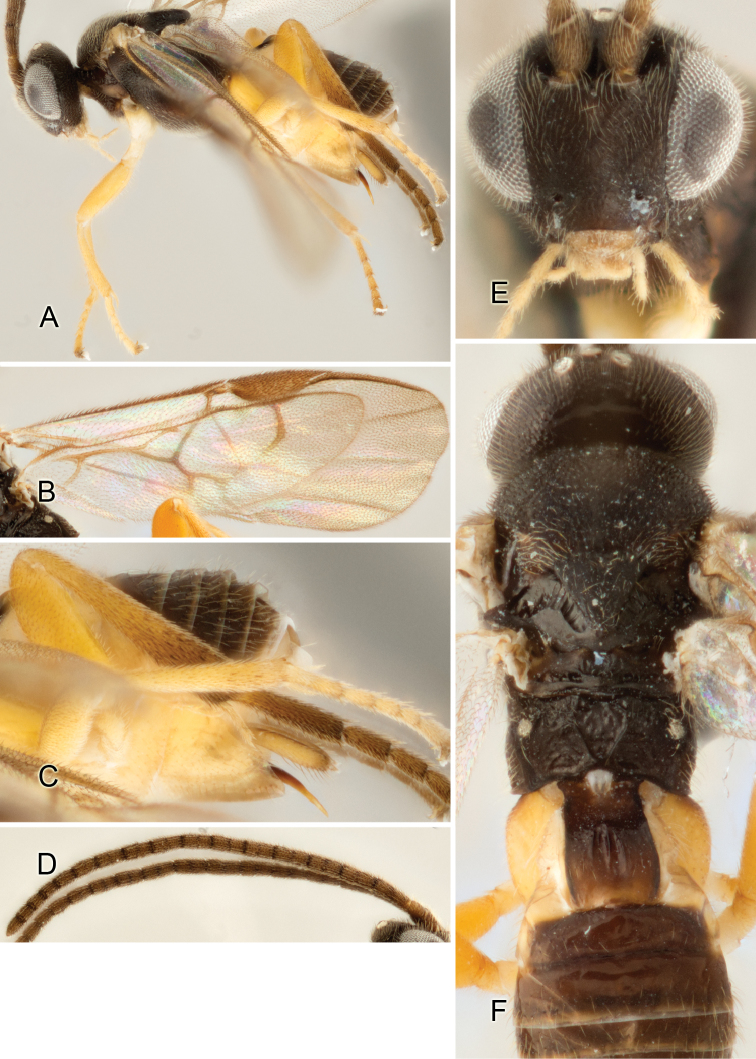
*Apanteles leonelgarayi*. **A** Habitus, lateral view **B** Fore wing **C** Hypopygium and ovipositor sheats **D** Antenna **E** Head, frontal view **F** Head, meso- and metasoma (partially), dorsal view.

**Figure 139.**
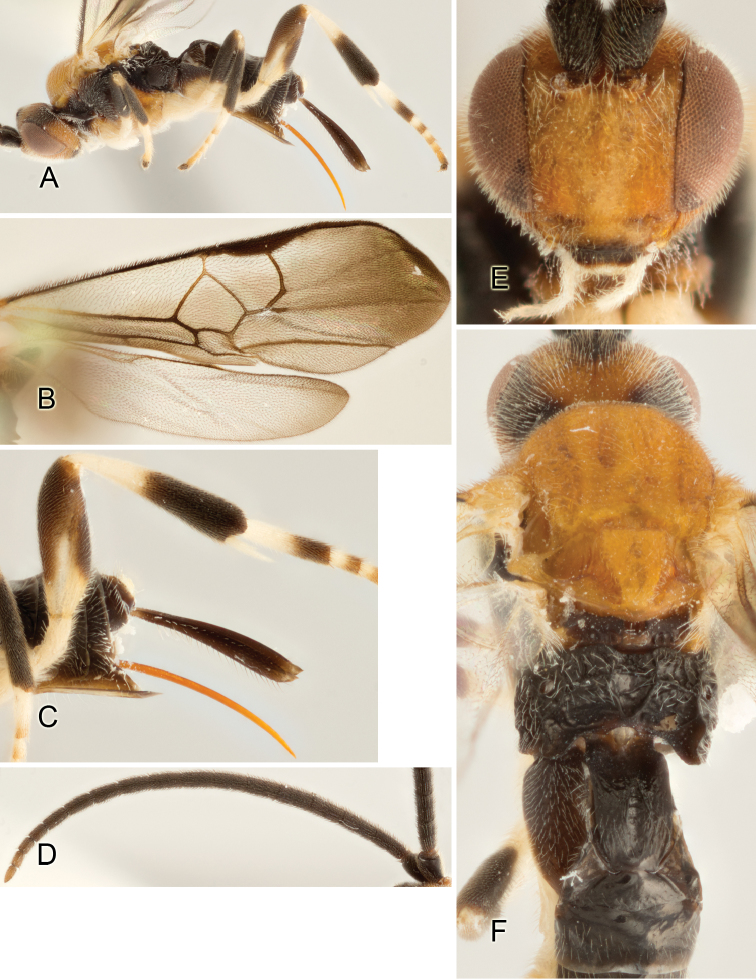
*Apanteles luisgaritai*. **A** Habitus, lateral view **B** Fore wing **C** Hypopygium and ovipositor sheats **D** Antenna **E** Head, frontal view **F** Head, meso- and metasoma (partially), dorsal view.

**Figure 140.**
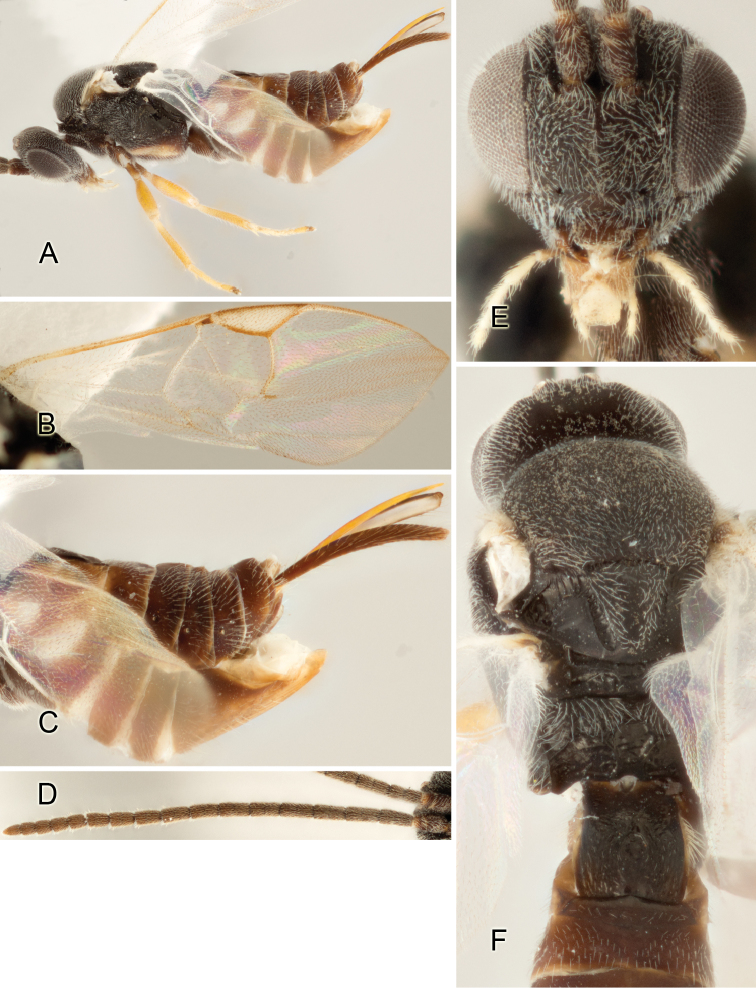
*Apanteles luisvargasi*. **A** Habitus, lateral view **B** Fore wing **C** Hypopygium and ovipositor sheats **D** Antenna **E** Head, frontal view **F** Head, meso- and metasoma (partially), dorsal view.

**Figure 141.**
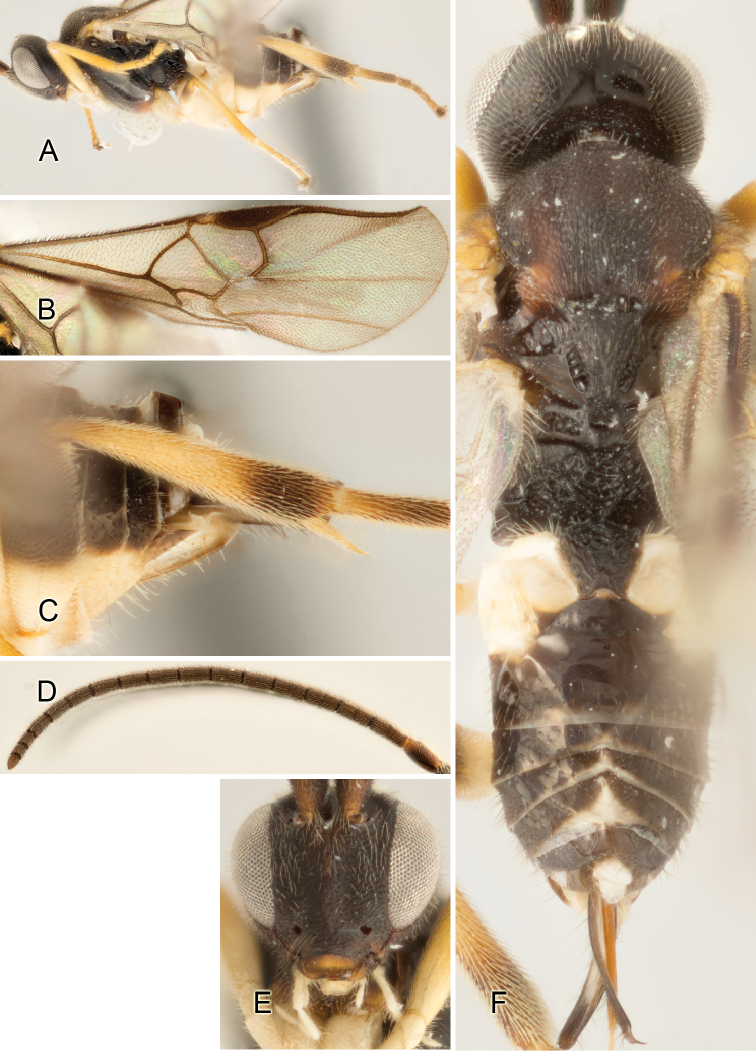
*Apanteles marcogonzalezi*. **A** Habitus, lateral view **B** Fore wing **C** Hypopygium and ovipositor sheats **D** Antenna **E** Head, frontal view **F** Head, meso- and metasoma, dorsal view.

**Figure 142.**
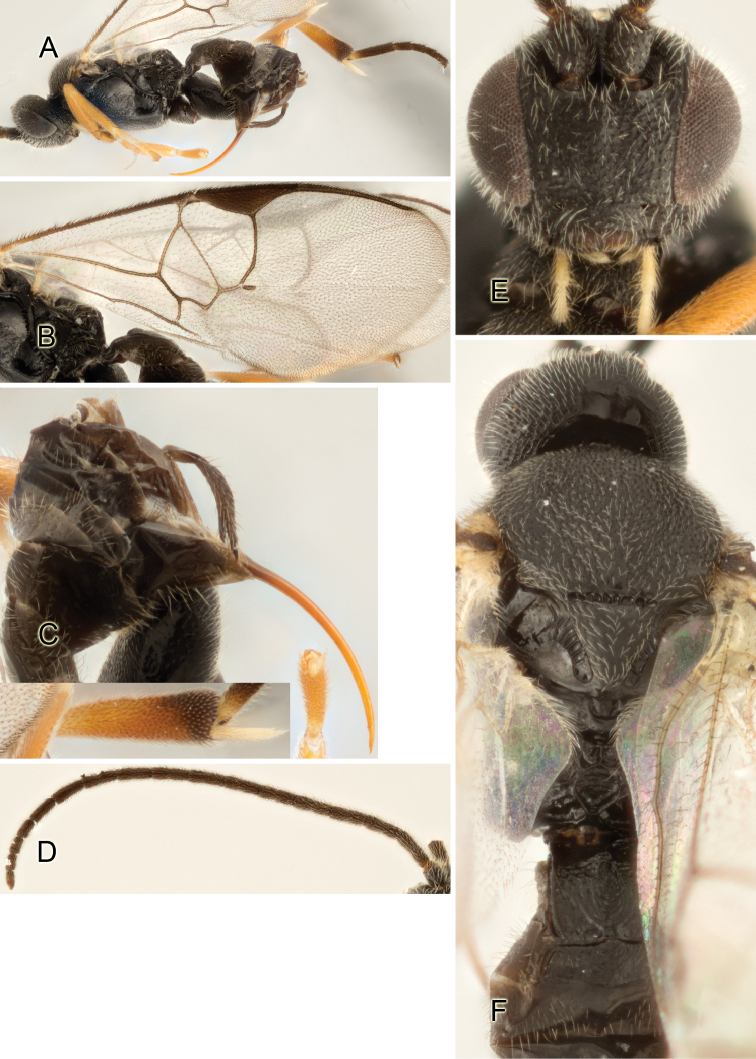
*Apanteles marialuisariasae*. **A** Habitus, lateral view **B** Fore wing **C** Hypopygium and ovipositor sheats, with details of metatibia **D** Antenna **E** Head, frontal view **F** Head, meso- and metasoma (partially), dorsal view.

**Figure 143.**
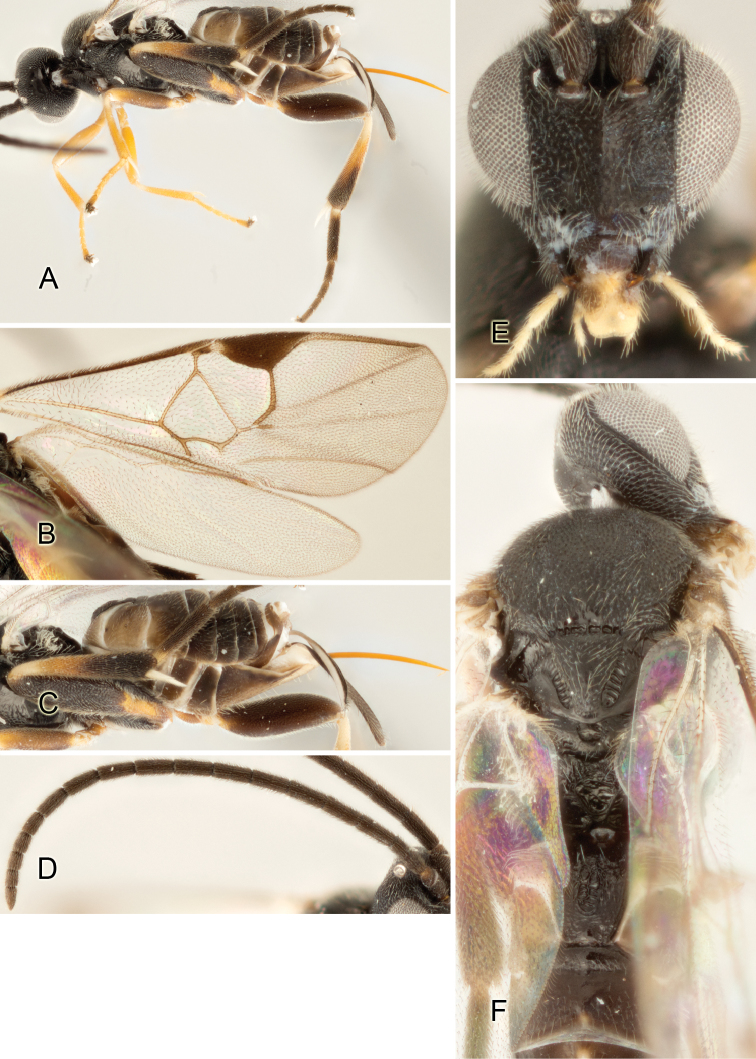
*Apanteles mariamendezae*. **A** Habitus, lateral view **B** Fore wing **C** Hypopygium and ovipositor sheats **D** Antenna **E** Head, frontal view **F** Head, meso- and metasoma (partially), dorsal view.

**Figure 144.**
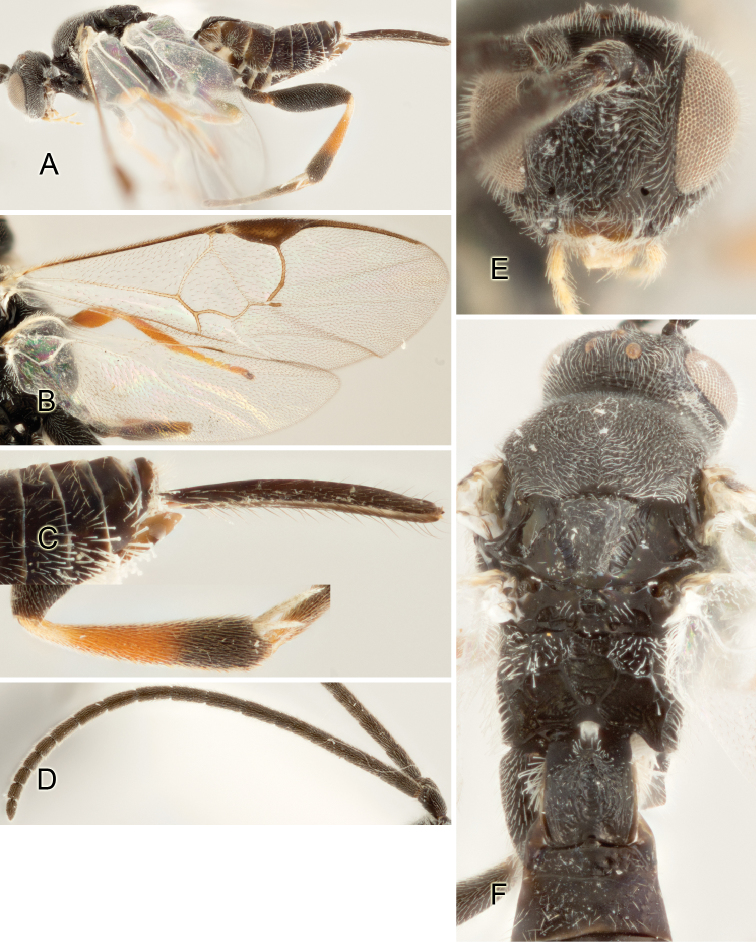
*Apanteles marisolnavarroae*. **A** Habitus, lateral view **B** Fore wing **C** Hypopygium and ovipositor sheats **D** Antenna **E** Head, frontal view **F** Head, meso- and metasoma (partially), dorsal view.

**Figure 145.**
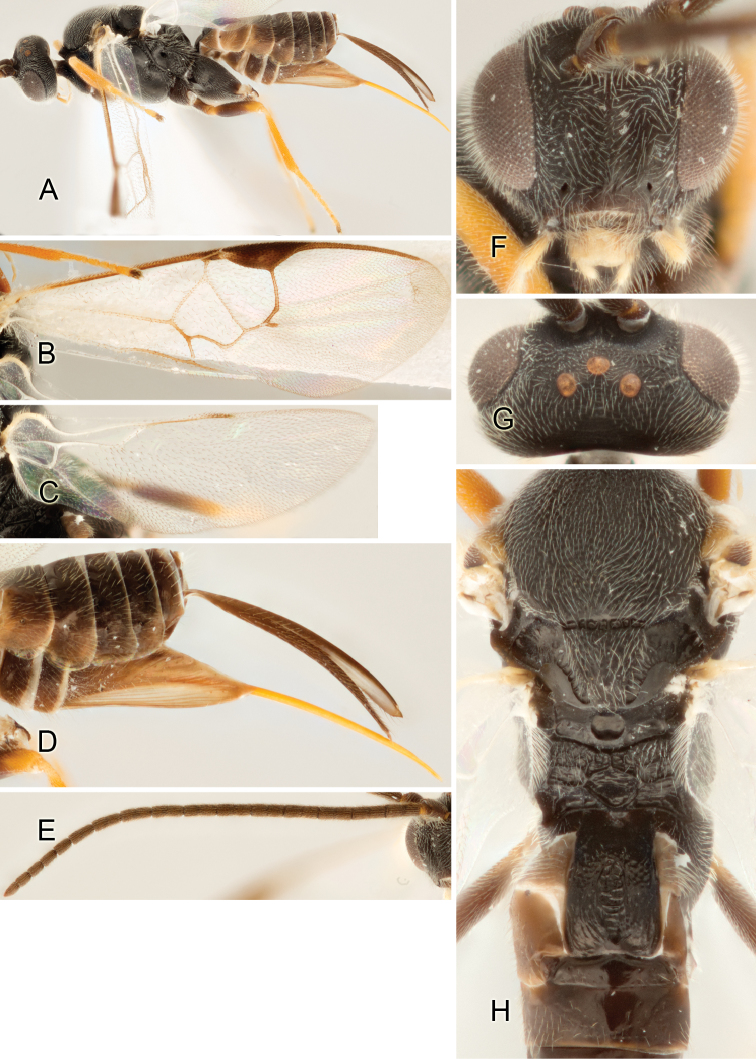
*Apanteles randallmartinezi*. **A** Habitus, lateral view **B** Fore wing **C** Hind wing **D** Hypopygium and ovipositor sheats **E** Antenna **F** Head, frontal view **G** Head, dorsal view **H** Meso- and metasoma (partially), dorsal view.

**Figure 146.**
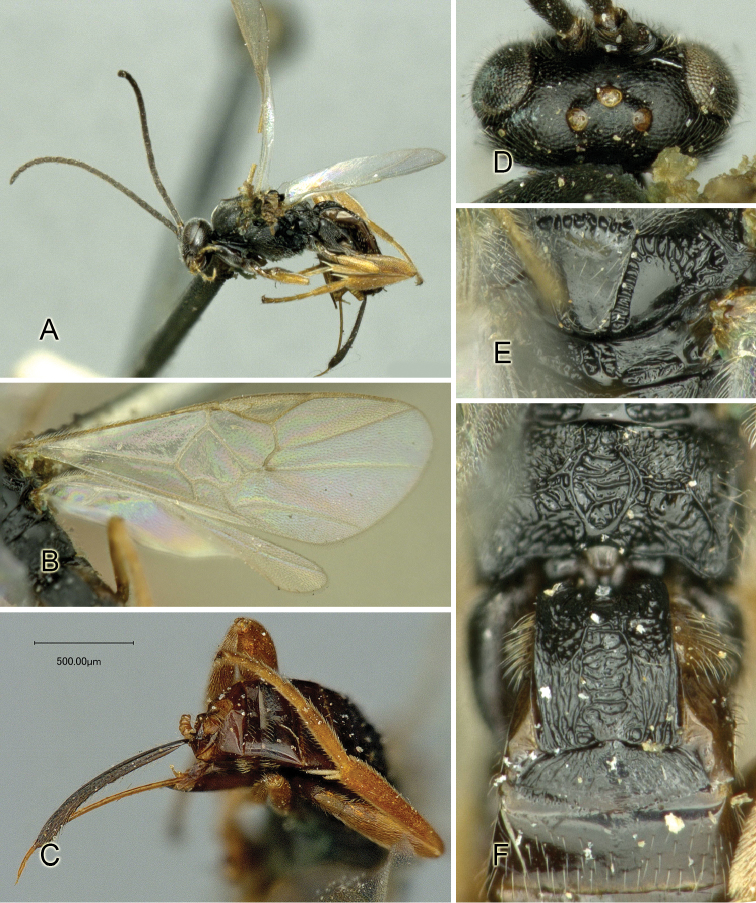
*Apanteles balthazari*. **A** Habitus, lateral view **B** Fore wing **C** Hypopygium and ovipositor sheats **D** Head, dorsal view **E** Mesosoma (partially), dorsal view **F** Propodeum and metasoma (partially), dorsal view.

**Figure 147.**
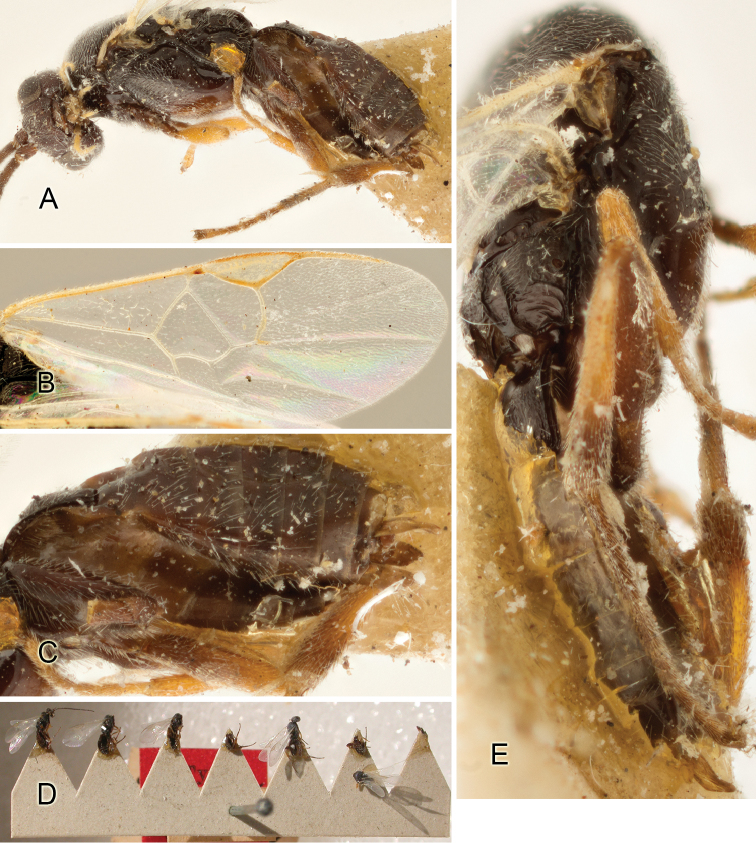
*Apanteles megathymi*. **A** Habitus, lateral view **B** Fore wing **C** Metasoma, lateral view **D** Syntypes **E** Meso- and metasoma, lateral view.

**Figure 148.**
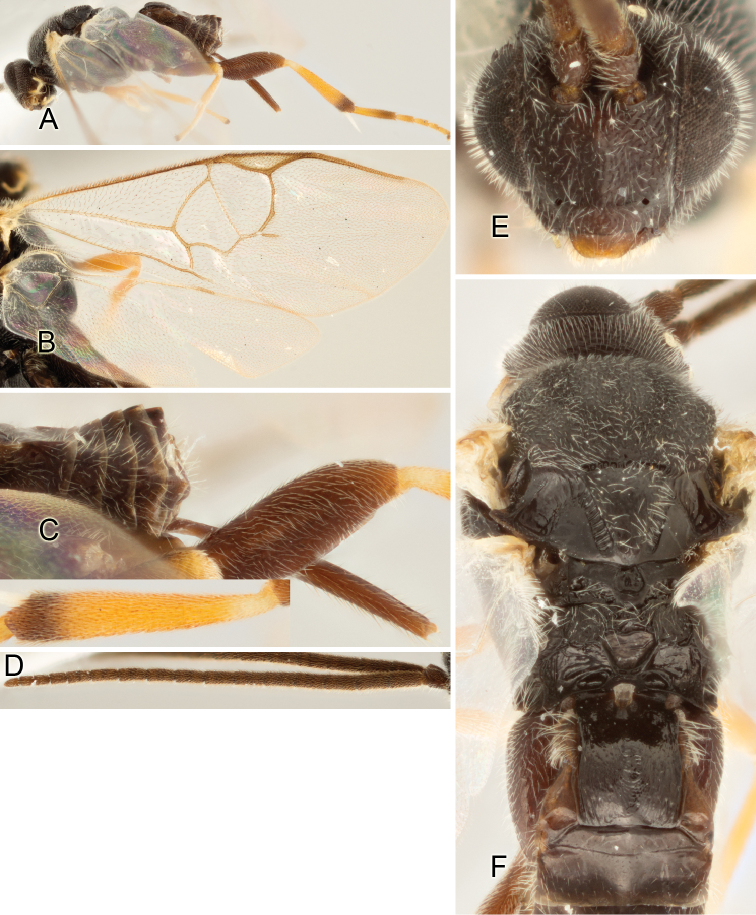
*Apanteles monicachavarriae*. **A** Habitus, lateral view **B** Fore wing **C** Hypopygium and ovipositor sheats **D** Antenna **E** Head, frontal view **F** Head, meso- and metasoma (partially), dorsal view.

**Figure 149.**
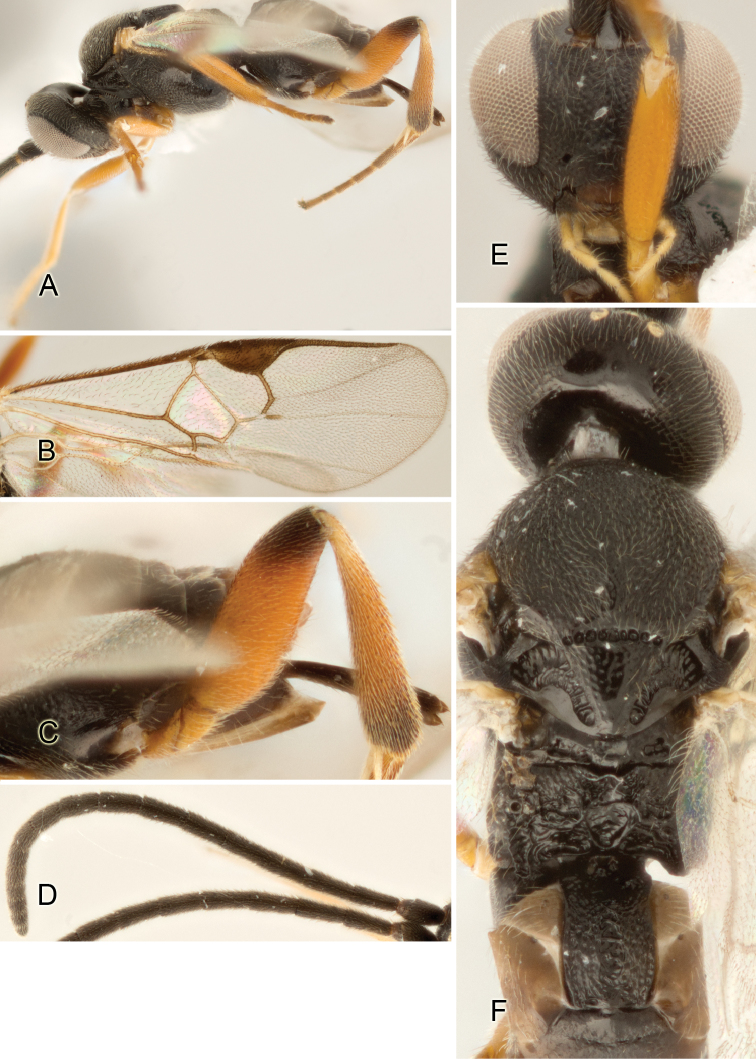
*Apanteles oscarchavezi*. **A** Habitus, lateral view **B** Fore wing **C** Hypopygium and ovipositor sheats **D** Antenna **E** Head, frontal view **F** Head, meso- and metasoma (partially), dorsal view.

**Figure 150.**
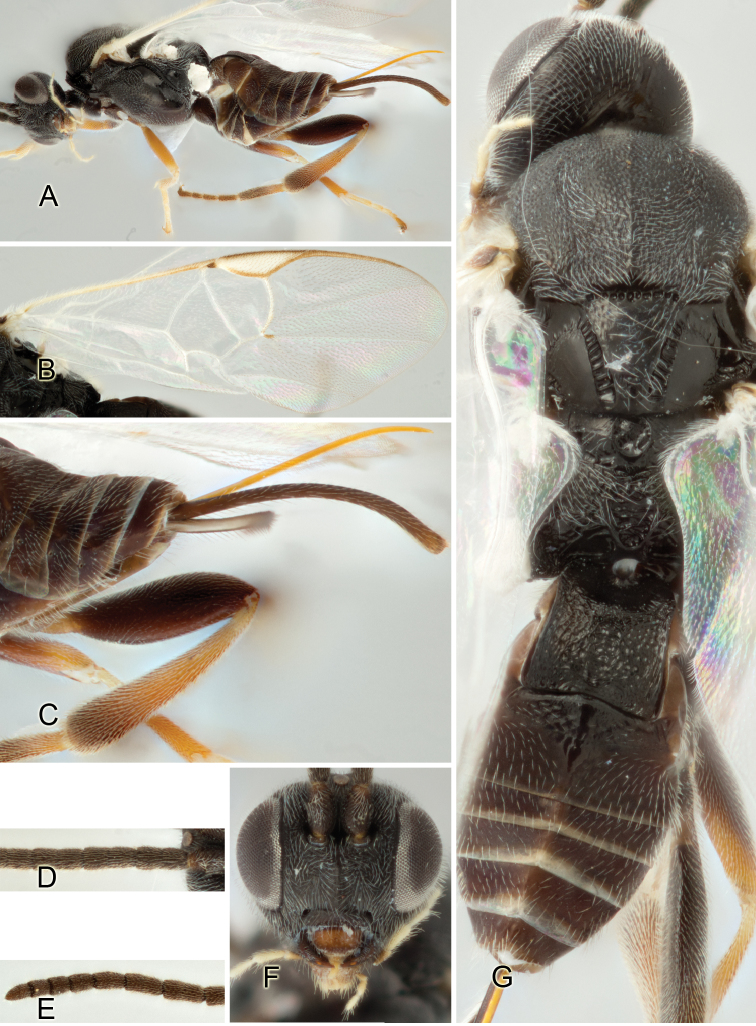
*Apanteles esthercentenoae*. **A** Habitus, lateral view **B** Fore wing **C** Hypopygium and ovipositor sheats **D** Anterior half of antenna **E** Posterior half of antenna **F** Head, frontal view **G** Head, meso- and metasoma, dorsal view.

**Figure 151.**
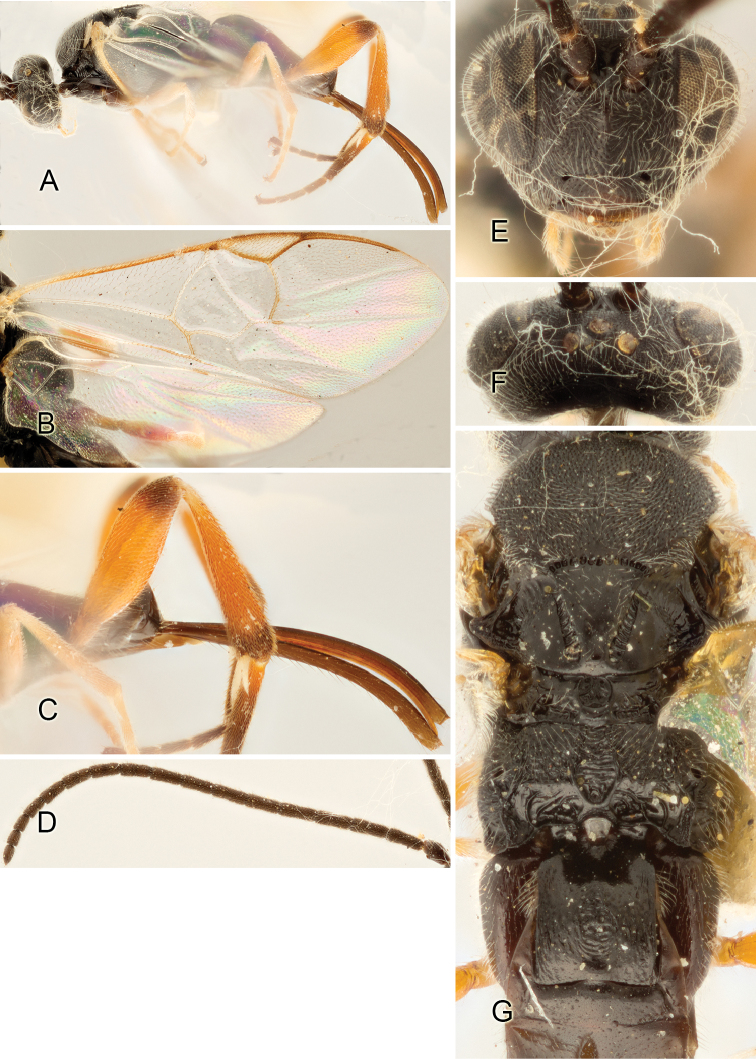
*Apanteles megastidis*. **A** Habitus, lateral view **B** Fore wing **C** Hypopygium and ovipositor sheats **D** Antenna **E** Head, frontal view **F** Head, dorsal view **G** Meso- and metasoma (partially), dorsal view.

**Figure 152.**
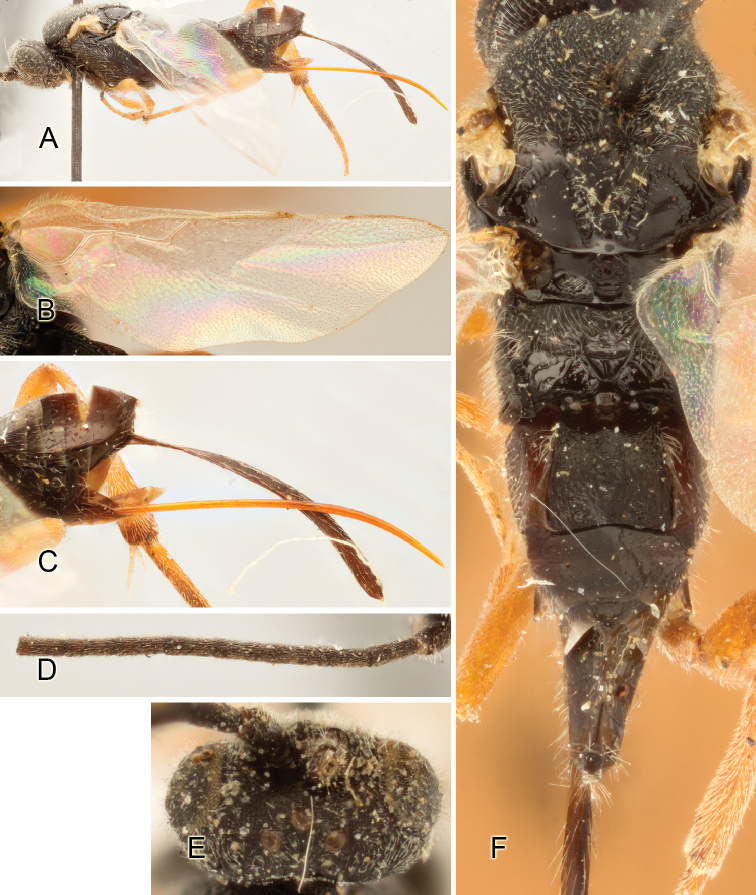
*Apanteles paranthrenidis*. **A** Habitus, lateral view **B** Hind wing **C** Hypopygium and ovipositor sheats **D** Antenna (partially) **E** Head, dorsal view **F** Head, meso- and metasoma (partially), dorsal view.

**Figure 153.**
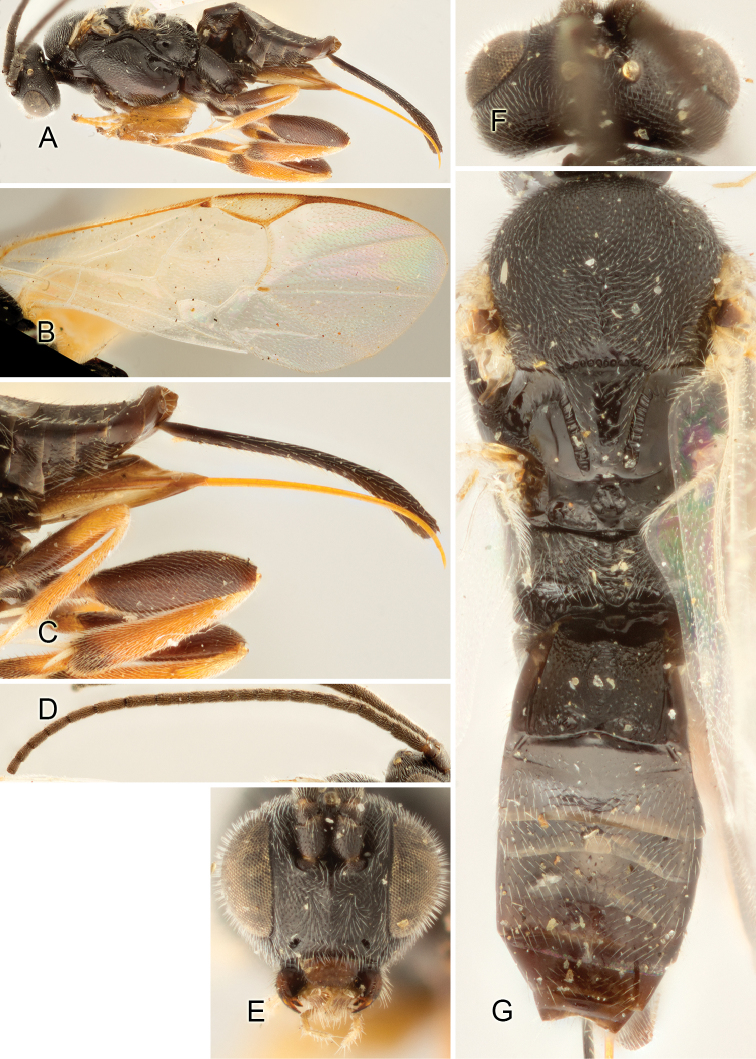
*Apanteles thurberiae*. **A** Habitus, lateral view **B** Fore wing **C** Hypopygium and ovipositor sheats **D** Antenna (partially) **E** Head, frontal view **F** Head, dorsal view **G** Meso- and metasoma, dorsal view.

**Figure 154.**
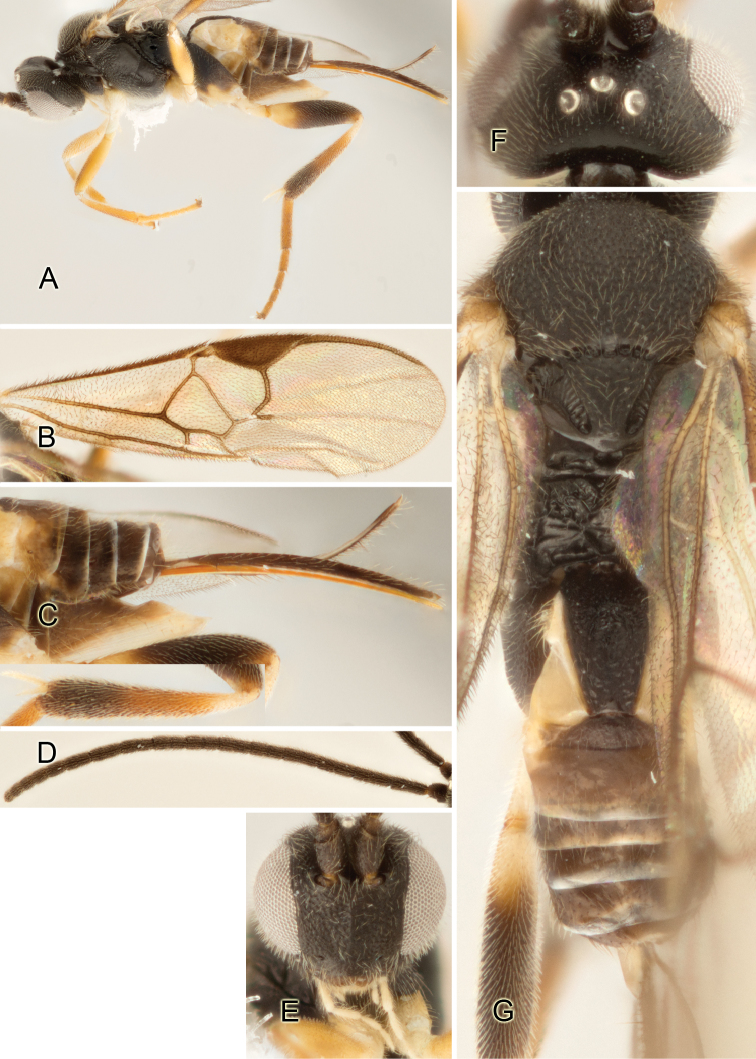
*Apanteles robertmontanoi*. **A** Habitus, lateral view **B** Fore wing **C** Hypopygium and ovipositor sheats **D** Antenna **E** Head, frontal view **F** Head, dorsal view **G** Meso- and metasoma, dorsal view.

**Figure 155.**
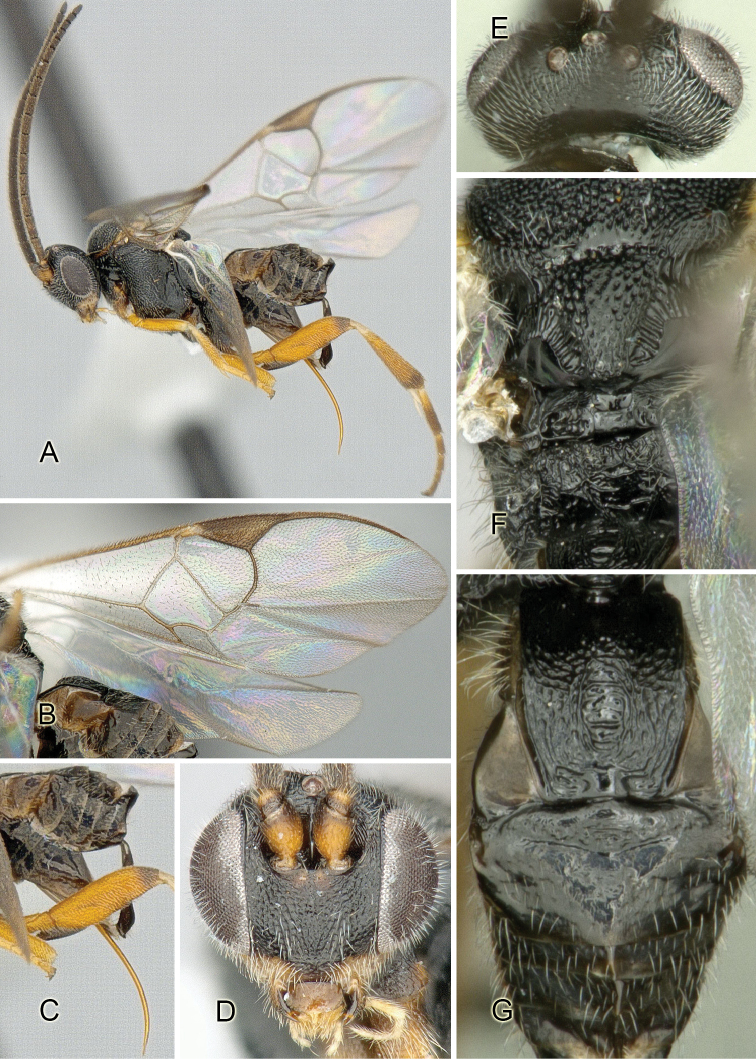
*Apanteles rogerblancoi*. **A** Habitus, lateral view **B** Fore wing **C** Hypopygium and ovipositor sheats **D** Head, frontal view **E** Head, dorsal view **F** Mesosoma (partially), dorsal view **G** Metasoma (partially), dorsal view.

**Figure 156.**
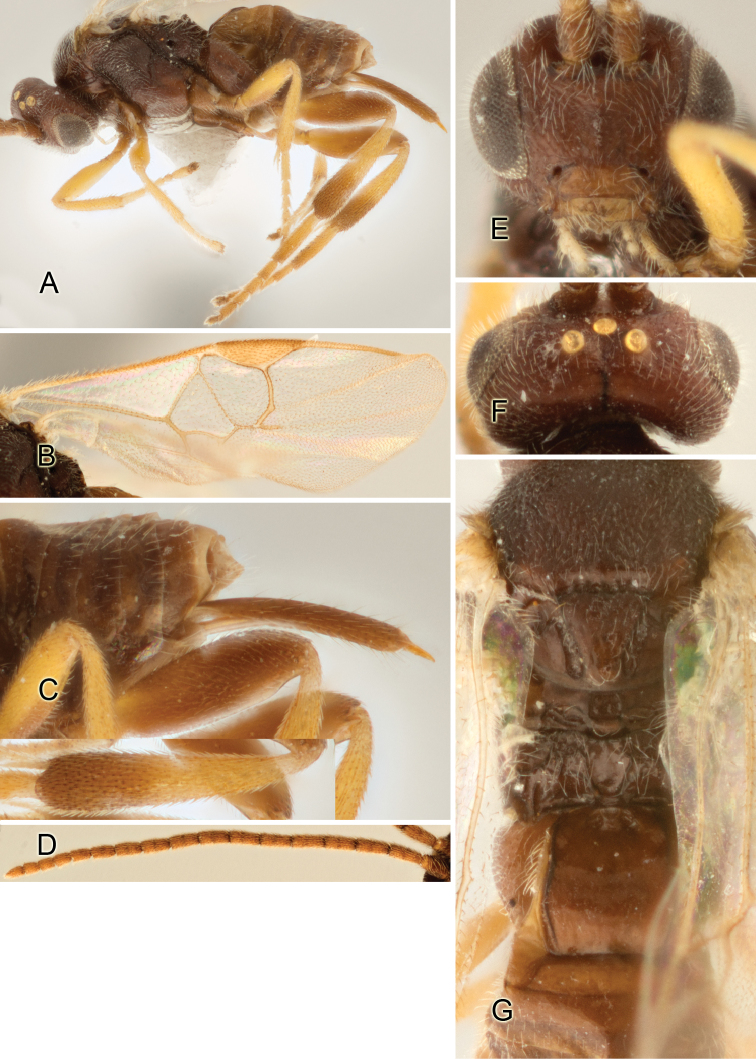
*Apanteles rolandovegai*. **A** Habitus, lateral view **B** Fore wing **C** Hypopygium and ovipositor sheats **D** Antenna **E** Head, frontal view **F** Head, dorsal view **G** Meso- and metasoma (partially), dorsal view.

**Figure 157.**
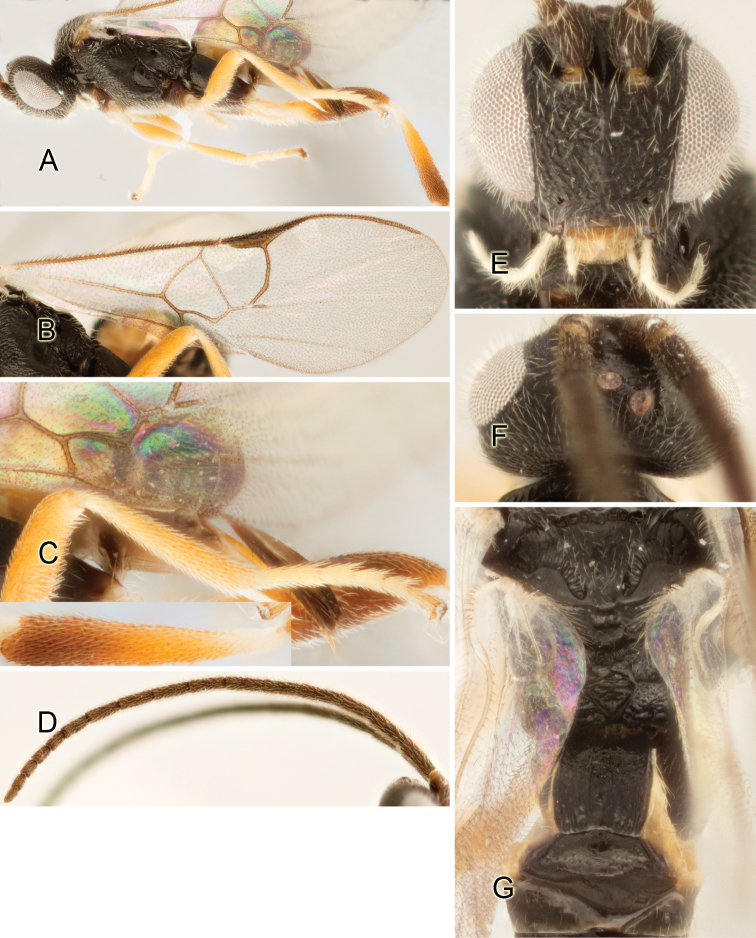
*Apanteles ronaldgutierrezi*. **A** Habitus, lateral view **B** Fore wing **C** Hypopygium and ovipositor sheats, with details of metatibia **D** Antenna **E** Head, frontal view **F** Head, dorsal view **G** Meso- and metasoma (partially), dorsal view.

**Figure 158.**
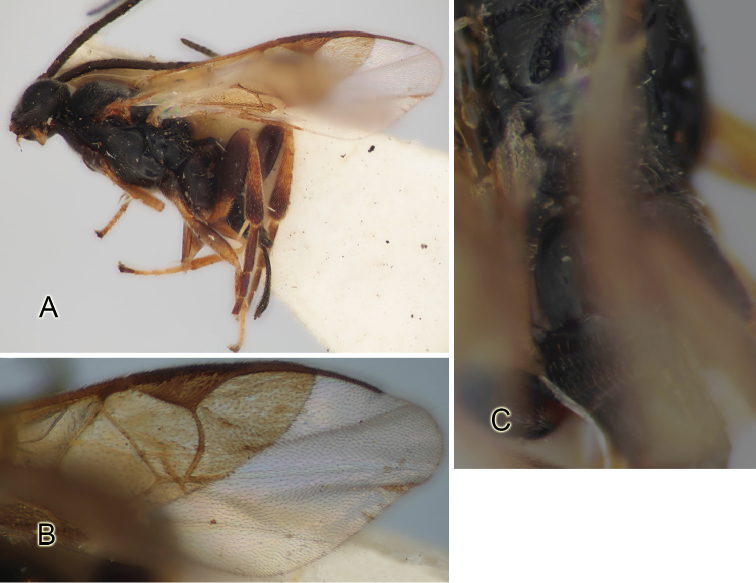
*Apanteles insularis*. **A** Habitus, lateral view **B** Fore wing **C** Meso- and metasoma (partially), dorsal view.

**Figure 159.**
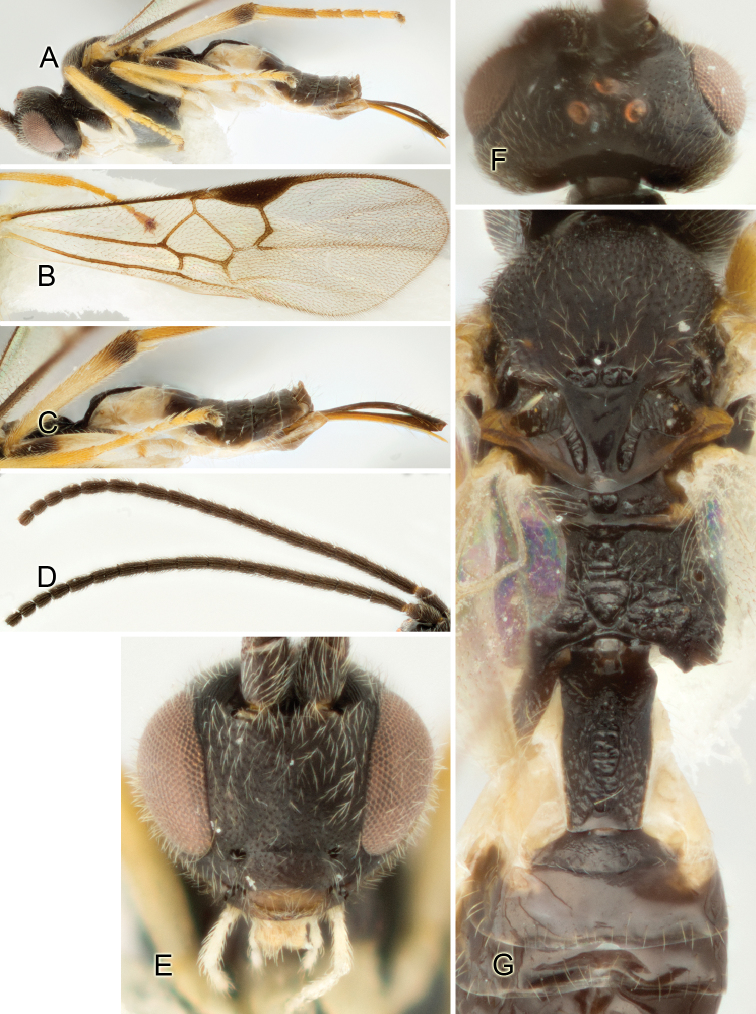
*Apanteles rosibelelizondoae*. **A** Habitus, lateral view **B** Fore wing **C** Hypopygium and ovipositor sheats **D** Antenna **E** Head, frontal view **F** Head, dorsal view **G** Meso- and metasoma (partially), dorsal view.

**Figure 160.**
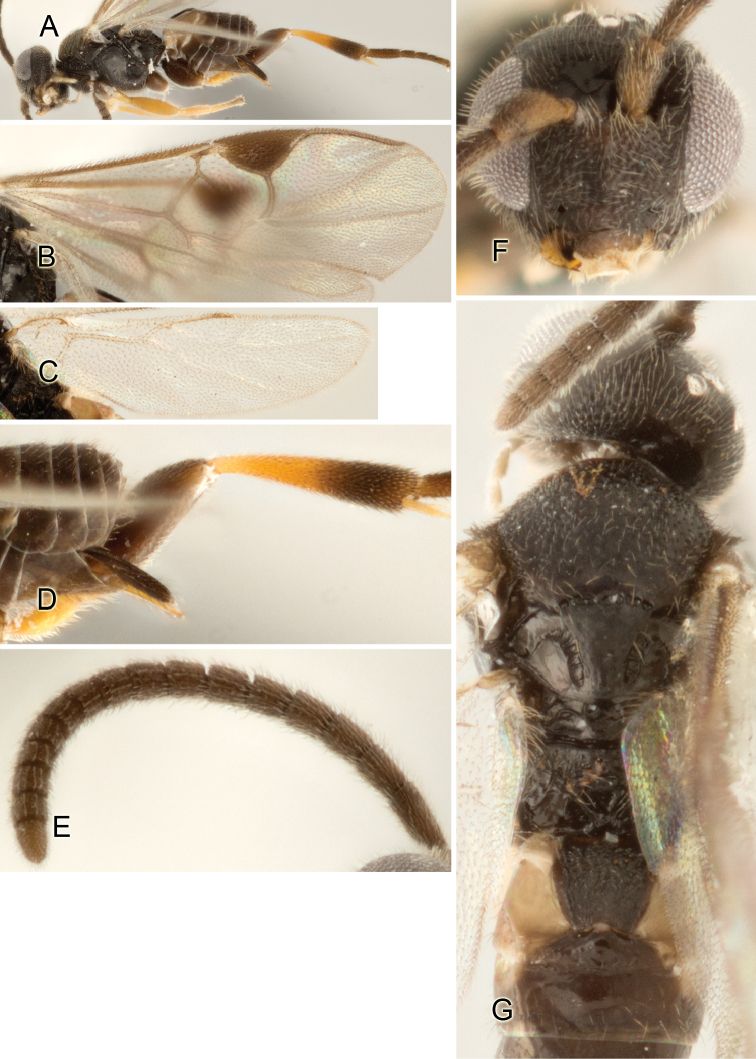
*Apanteles jimmychevezi*. **A** Habitus, lateral view **B** Fore wing **C** Hind wing **D** Hypopygium and ovipositor sheats **E** Antenna **F** Head, frontal view **G** Head, meso- and metasoma (partially), dorsal view.

**Figure 161.**
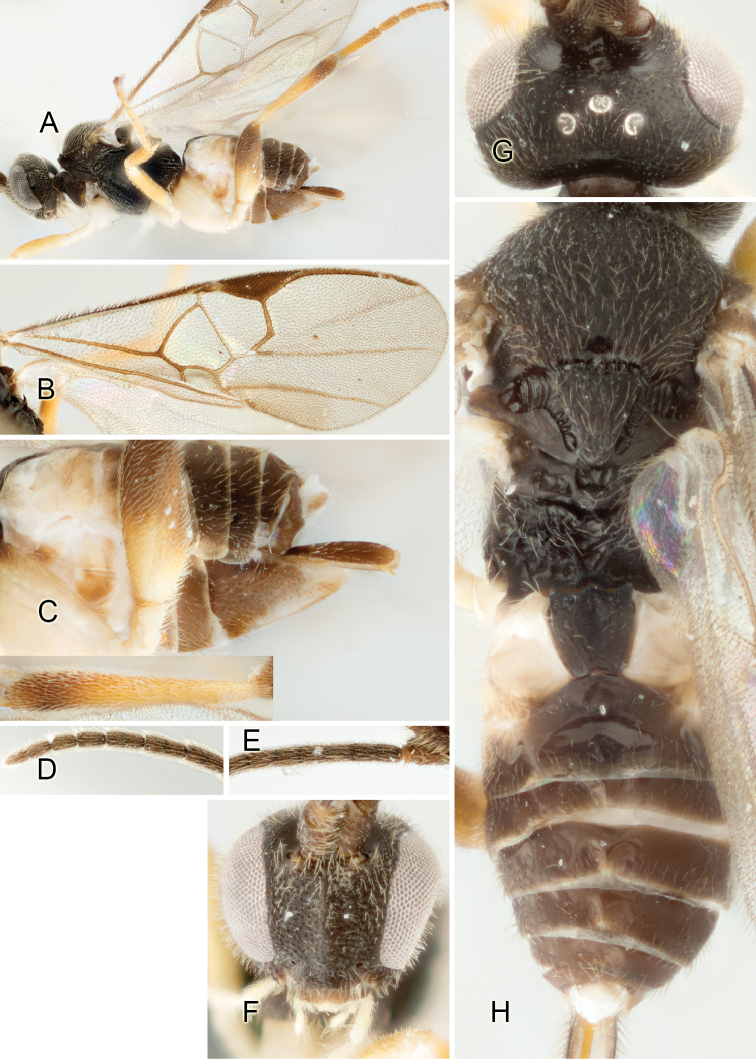
*Apanteles sergiocascantei*. **A** Habitus, lateral view **B** Fore wing **C** Hypopygium and ovipositor sheats, with details of metatibia **D** Anterior half of antenna **E** Posterior half of antenna **F** Head, frontal view **G** Head, dorsal view **H** Meso- and metasoma, dorsal view.

**Figure 162.**
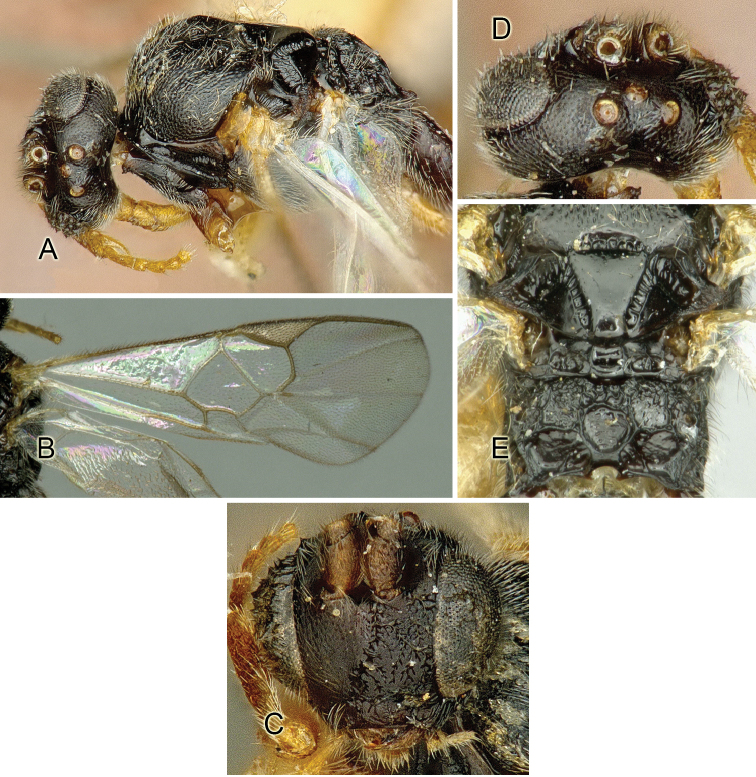
*Apanteles vulgaris*. **A** Head and mesosoma, lateral view **B** Fore wing **C** Head, frontal view **D** Head, dorsal view **E** Mesosoma (partially), dorsal view.

**Figure 163.**
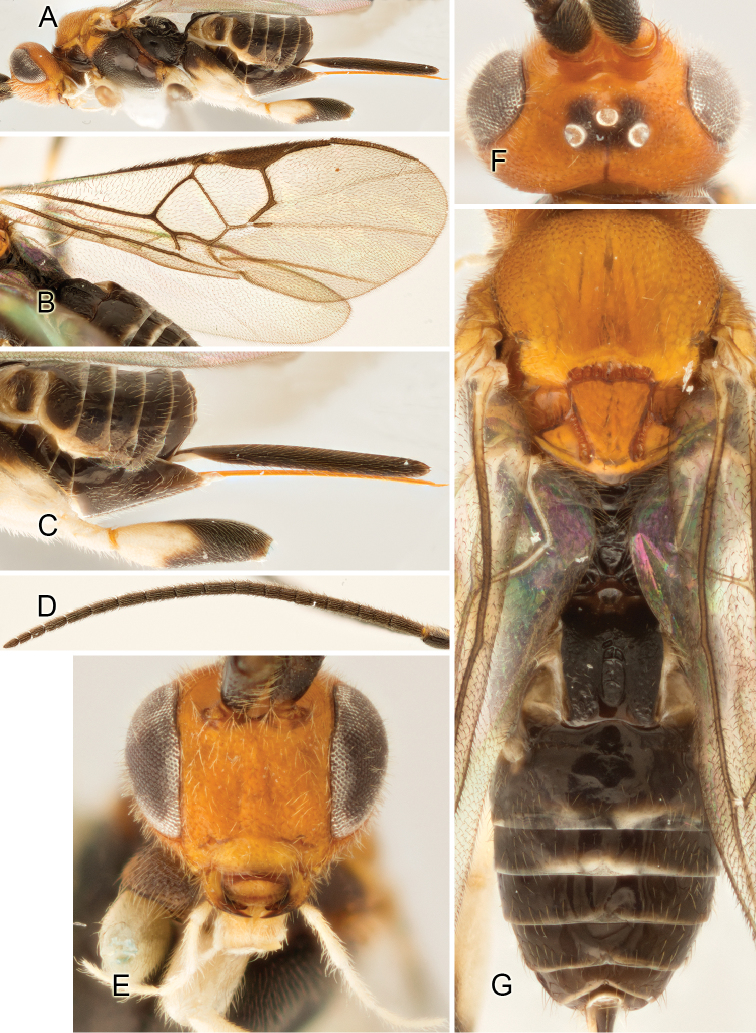
*Apanteles waldymedinai*. **A** Habitus, lateral view **B** Fore wing **C** Hypopygium and ovipositor sheats **D** Antenna **E** Head, frontal view **F** Head, dorsal view **G** Meso- and metasoma, dorsal view.

**Figure 164.**
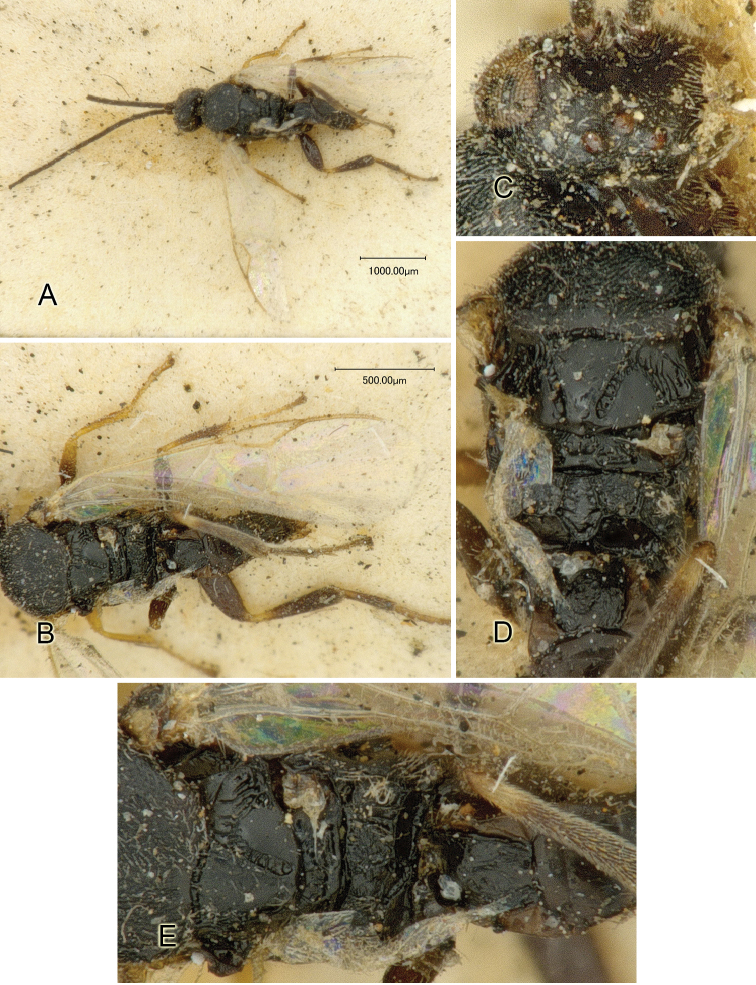
*Apanteles albinervis*. **A** Habitus, dorsal view **B** Fore wing **C** Head, dorsal view **D** and **E** Meso- and metasoma (partially), dorsal views.

**Figure 165.**
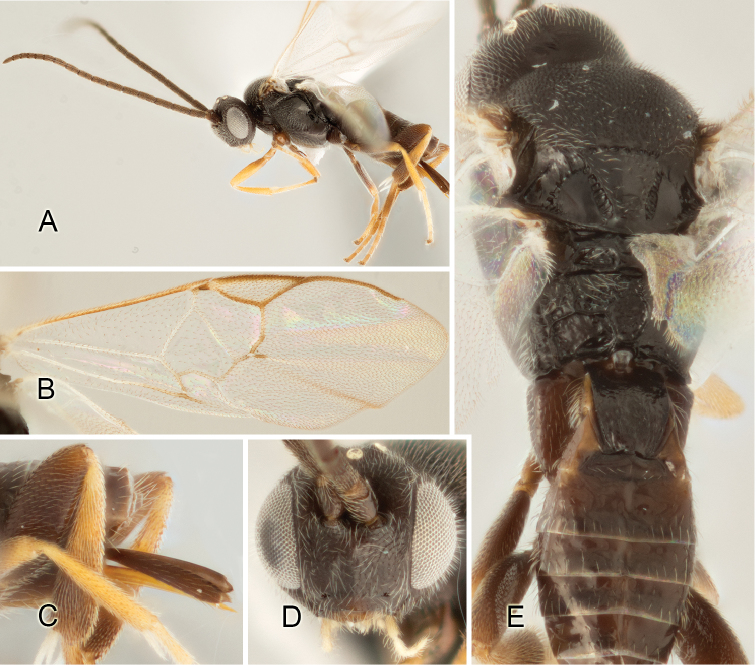
*Apanteles mariachavarriae*. **A** Habitus, lateral view **B** Fore wing **C** Hypopygium and ovipositor sheats **D** Head, frontal view **E** Head, meso- and metasoma (partially), dorsal view.

**Figure 166.**
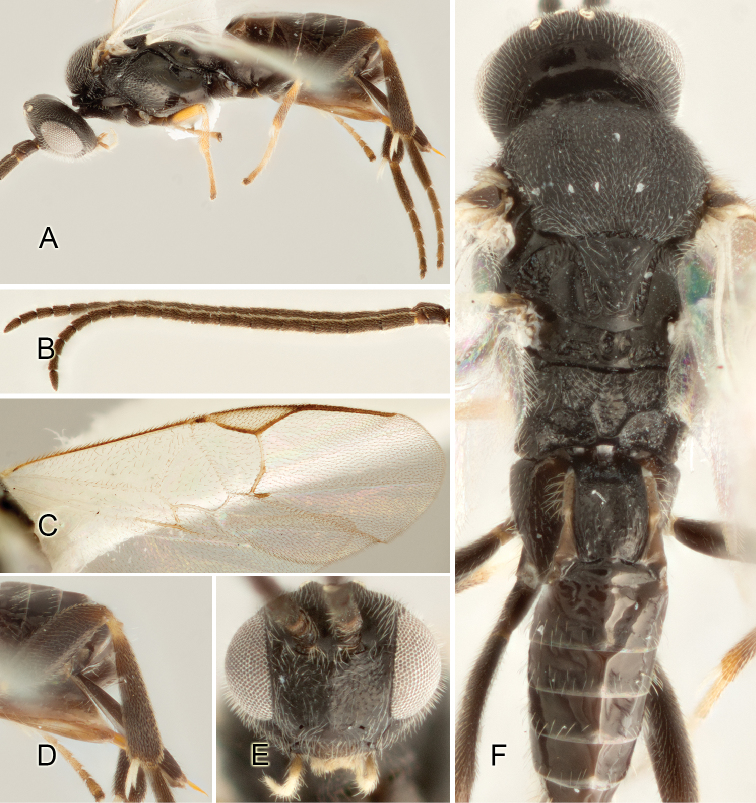
*Apanteles minorcarmonai*. **A** Habitus, lateral view **B** Antenna **C** Fore wing **D** Hypopygium and ovipositor sheats **E** Head, frontal view **F** Head, meso- and metasoma (partially), dorsal view.

**Figure 167.**
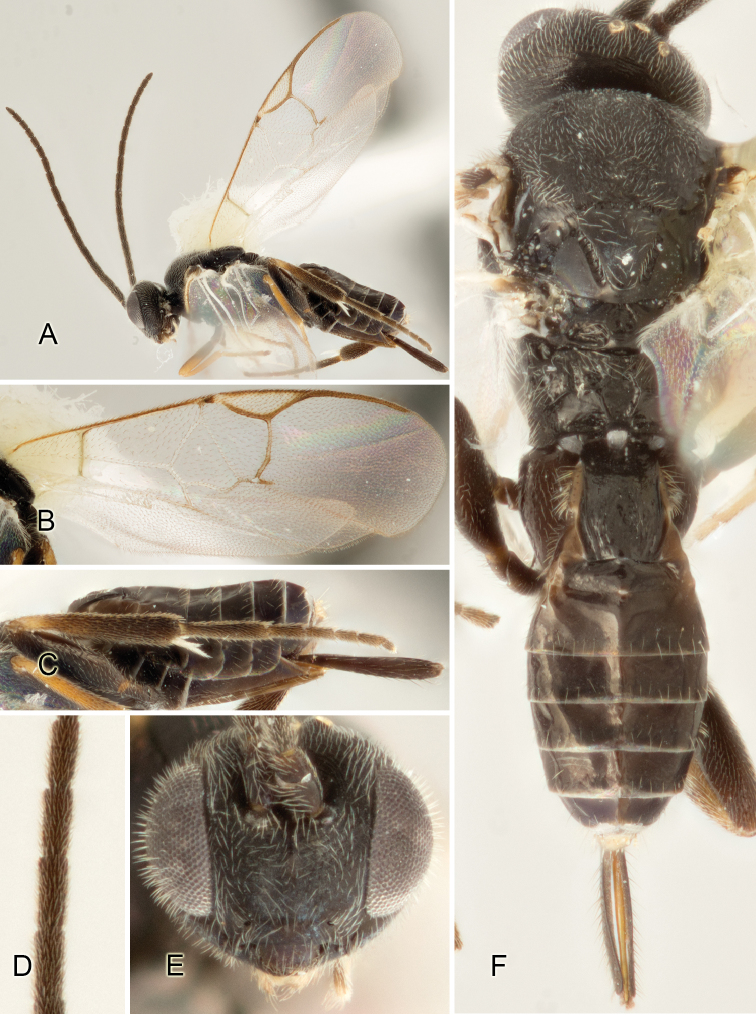
*Apanteles alvarougaldei*. **A** Habitus, lateral view **B** Fore wing **C** Hypopygium and ovipositor sheats **D** Antenna (partially) **E** Head, frontal view **F** Head, meso- and metasoma, dorsal view.

**Figure 168.**
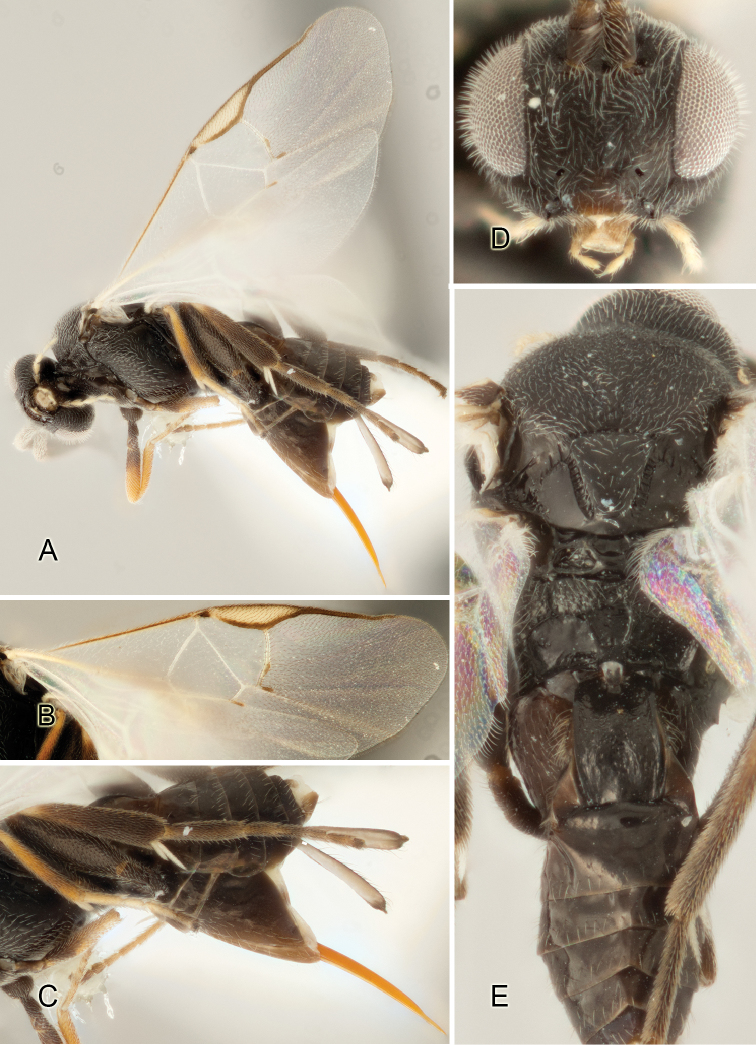
*Apanteles federicomatarritai*. **A** Habitus, lateral view **B** Fore wing **C** Hypopygium and ovipositor sheats **D** Head, frontal view **E** Head, meso- and metasoma (partially), dorsal view.

**Figure 169.**
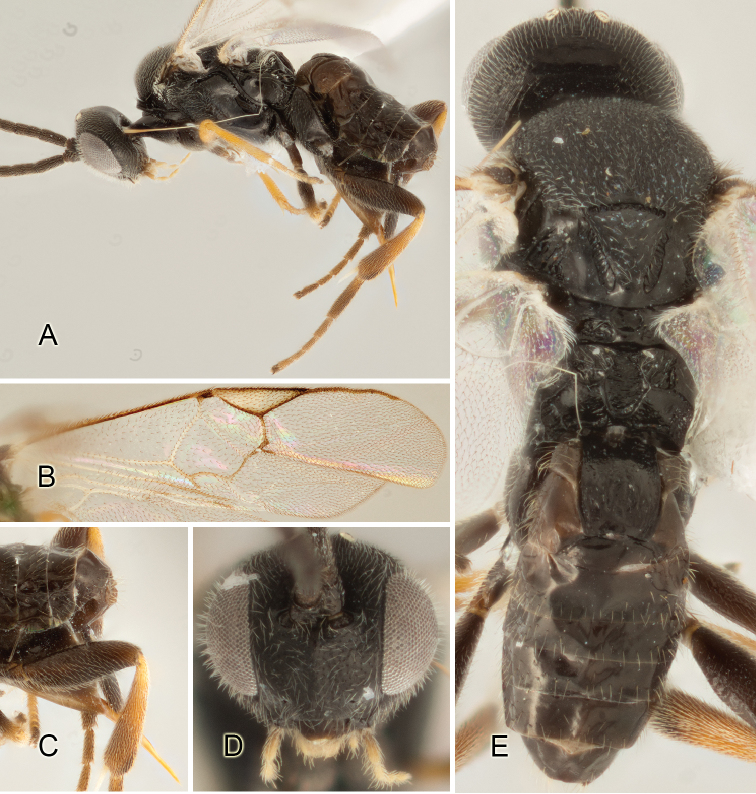
*Apanteles rostermoragai*. **A** Habitus, lateral view **B** Fore wing **C** Hypopygium and ovipositor sheats **D** Head, frontal view **E** Head, meso- and metasoma, dorsal view.

**Figure 170.**
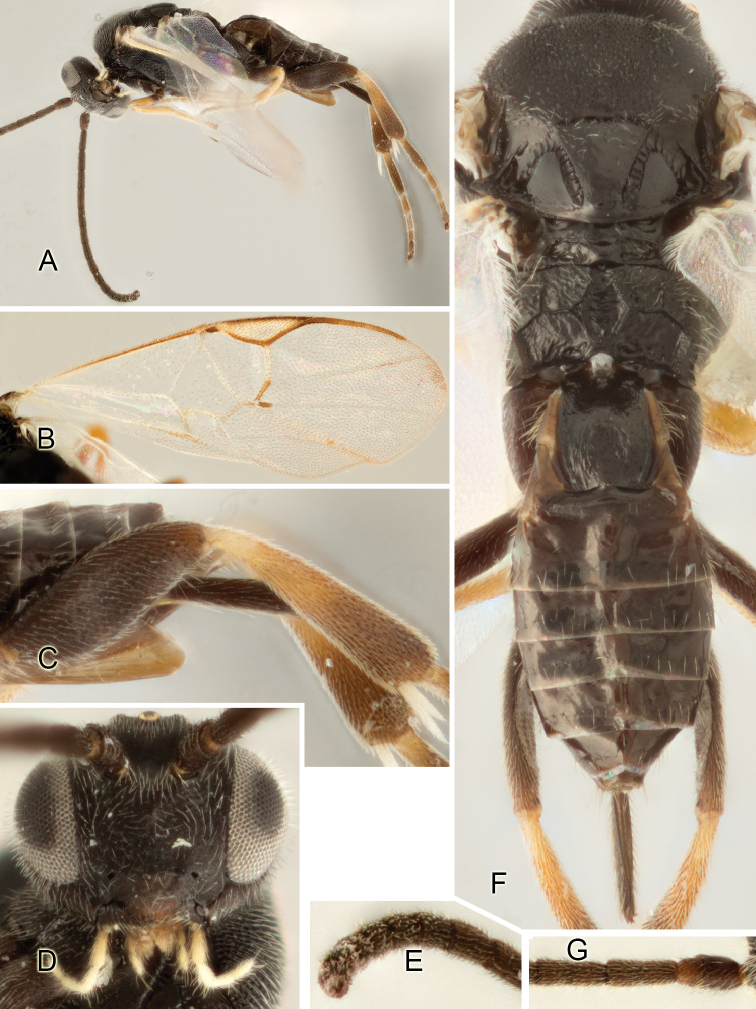
*Apanteles angelsolisi*. **A** Habitus, lateral view **B** Fore wing **C** Hypopygium and ovipositor sheats **D** Head, frontal view **E** Anterior half of antenna **F** Meso- and metasoma, dorsal view **G** Posterior half of antenna.

**Figure 171.**
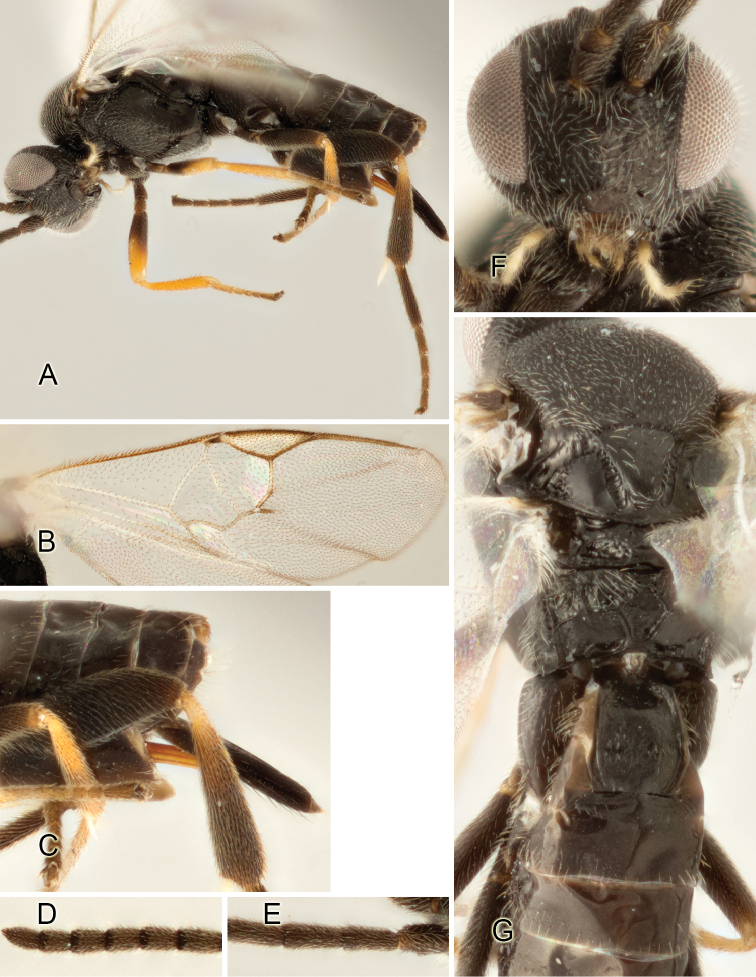
*Apanteles bernardoespinozai*. **A** Habitus, lateral view **B** Fore wing **C** Hypopygium and ovipositor sheats **D** Posterior half of antenna **E** Anterior half of antenna **F** Head, frontal view **G** Meso- and metasoma, dorsal view.

**Figure 172.**
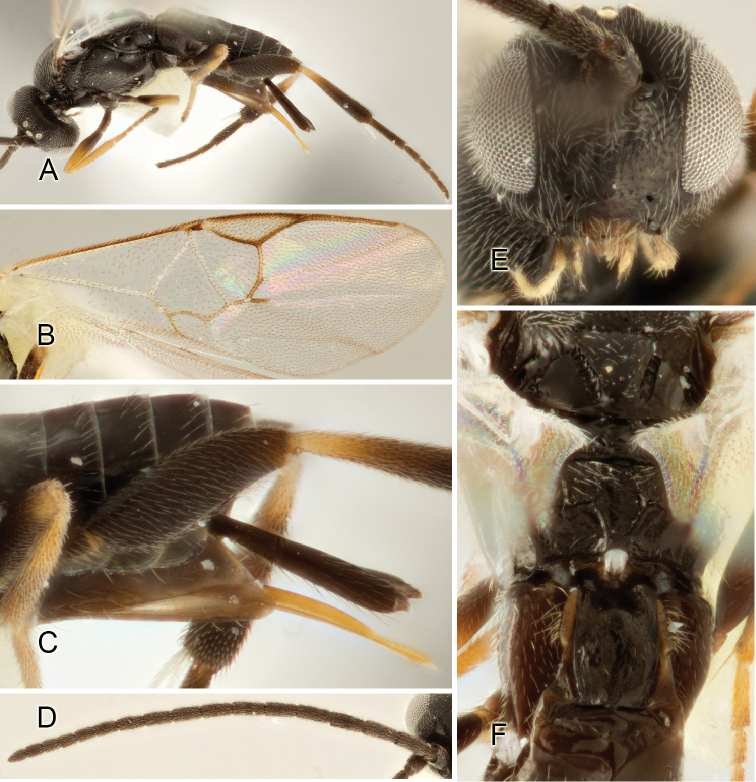
*Apanteles eliethcantillanoae*. **A** Habitus, lateral view **B** Fore wing **C** Hypopygium and ovipositor sheats **D** Antenna **E** Head, frontal view **F** Meso- and metasoma (partially), dorsal view.

**Figure 173.**
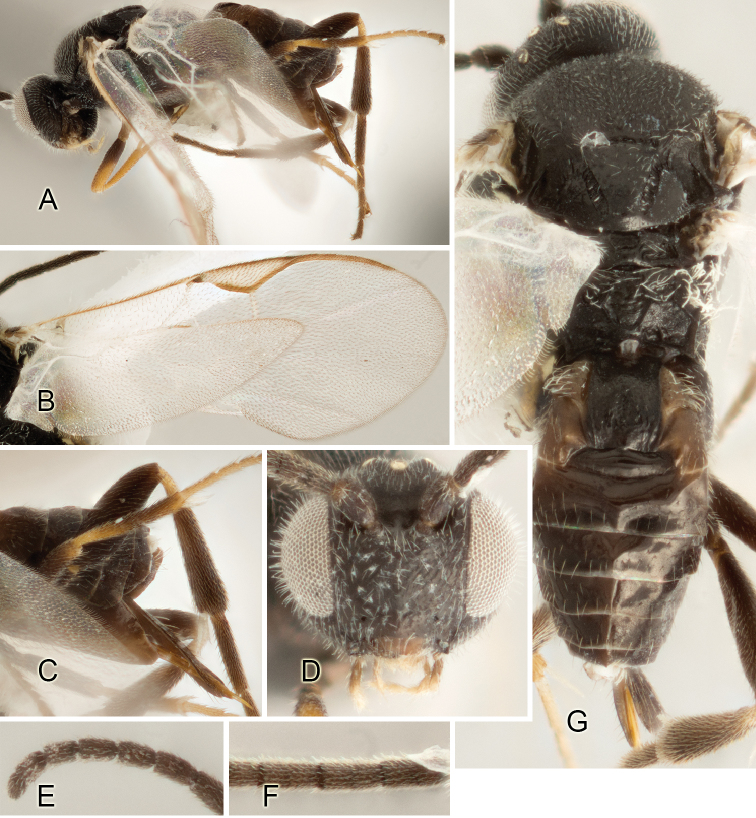
*Apanteles carlosviquezi*. **A** Habitus, lateral view **B** Fore wing **C** Hypopygium and ovipositor sheats **D** Head, frontal view **E** Anterior half of antenna **F** Posterior half of antenna **G** Head, meso- and metasoma, dorsal view.

**Figure 174.**
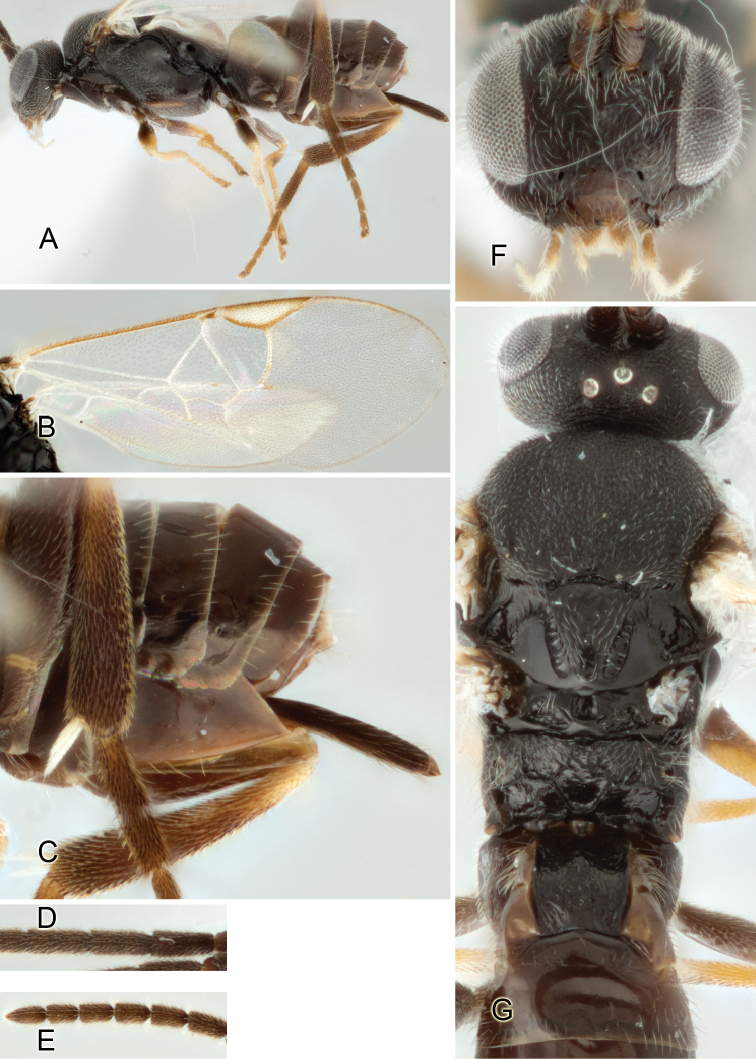
*Apanteles ciriloumanai*. **A** Habitus, lateral view **B** Fore wing **C** Hypopygium and ovipositor sheats **D** Anterior half of antenna **E** Posterior half of antenna **F** Head, frontal view **G** Head, meso- and metasoma (partially), dorsal view.

**Figure 175.**
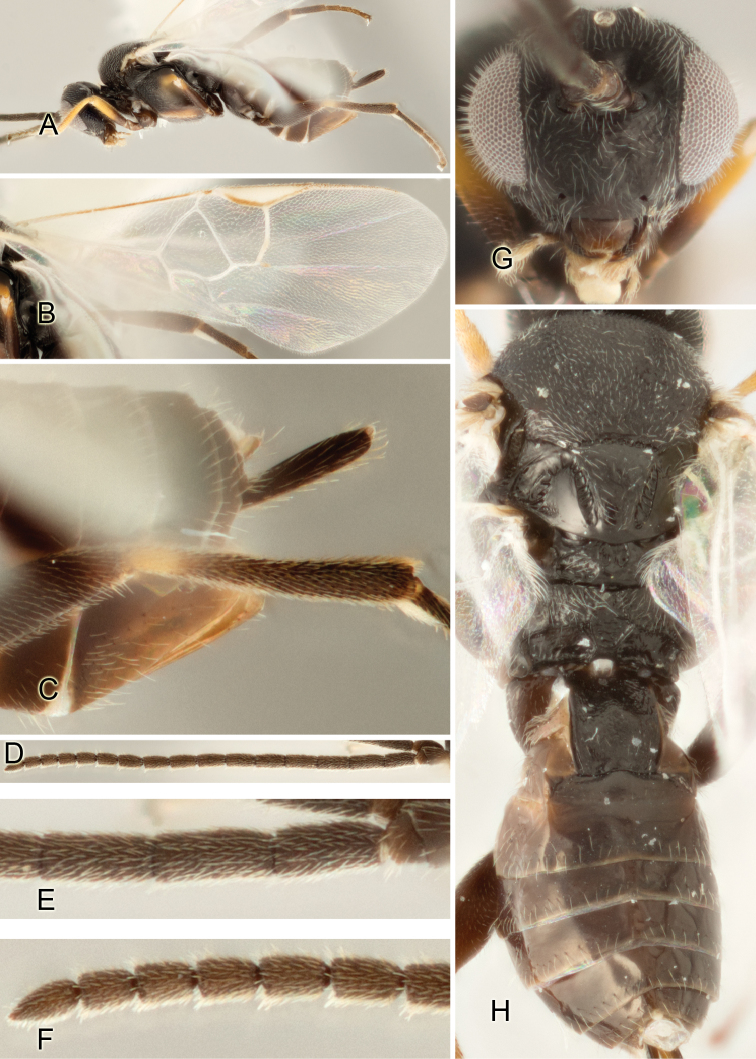
*Apanteles cynthiacorderoae*. **A** Habitus, lateral view **B** Fore wing **C** Hypopygium and ovipositor sheats **D** Antenna **E** Anterior half of antenna **F** Posterior half of antenna **G** Head, frontal view **H** Meso- and metasoma, dorsal view.

**Figure 176.**
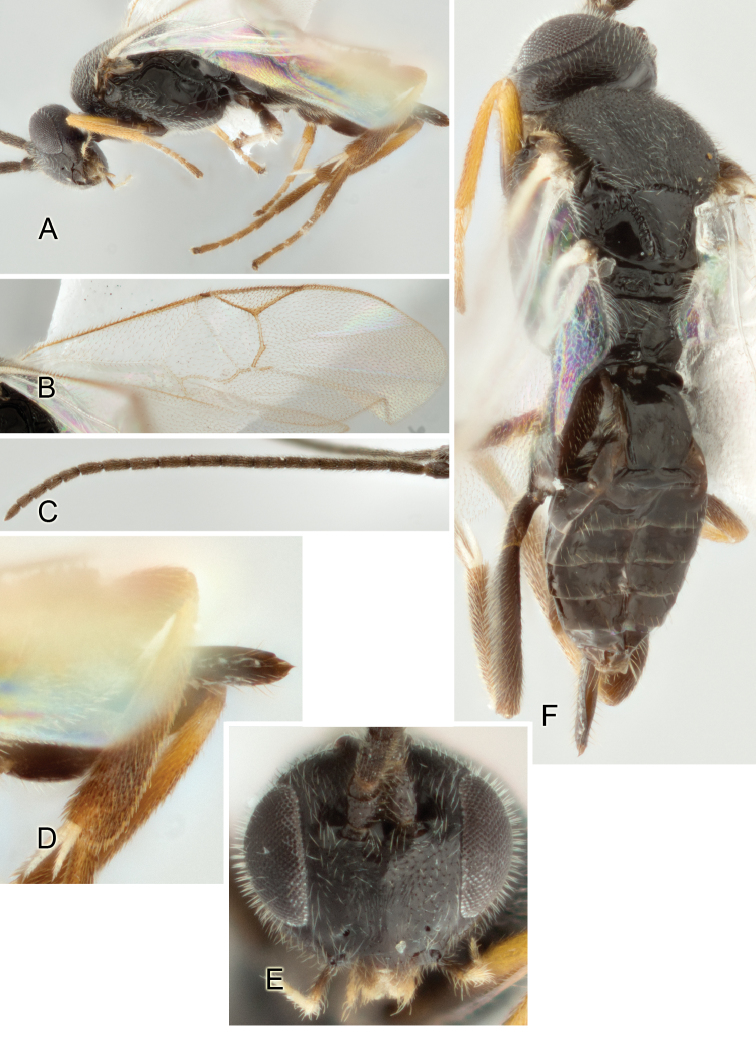
*Apanteles diniamartinezae*. **A** Habitus, lateral view **B** Fore wing **C** Antenna **D** Hypopygium and ovipositor sheats **E** Head, frontal view **F** Head, meso- and metasoma, dorsal view.

**Figure 177.**
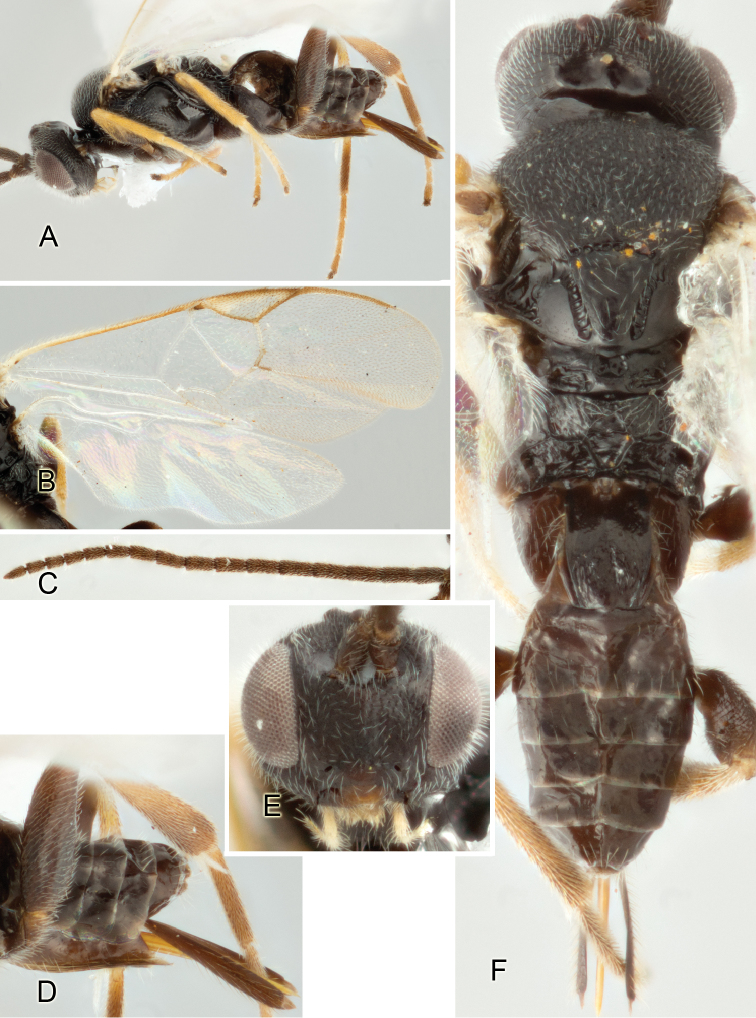
*Apanteles duvalierbricenoi*. **A** Habitus, lateral view **B** Fore wing **C** Antenna **D** Hypopygium and ovipositor sheats **E** Head, frontal view **F** Head, meso- and metasoma, dorsal view.

**Figure 178.**
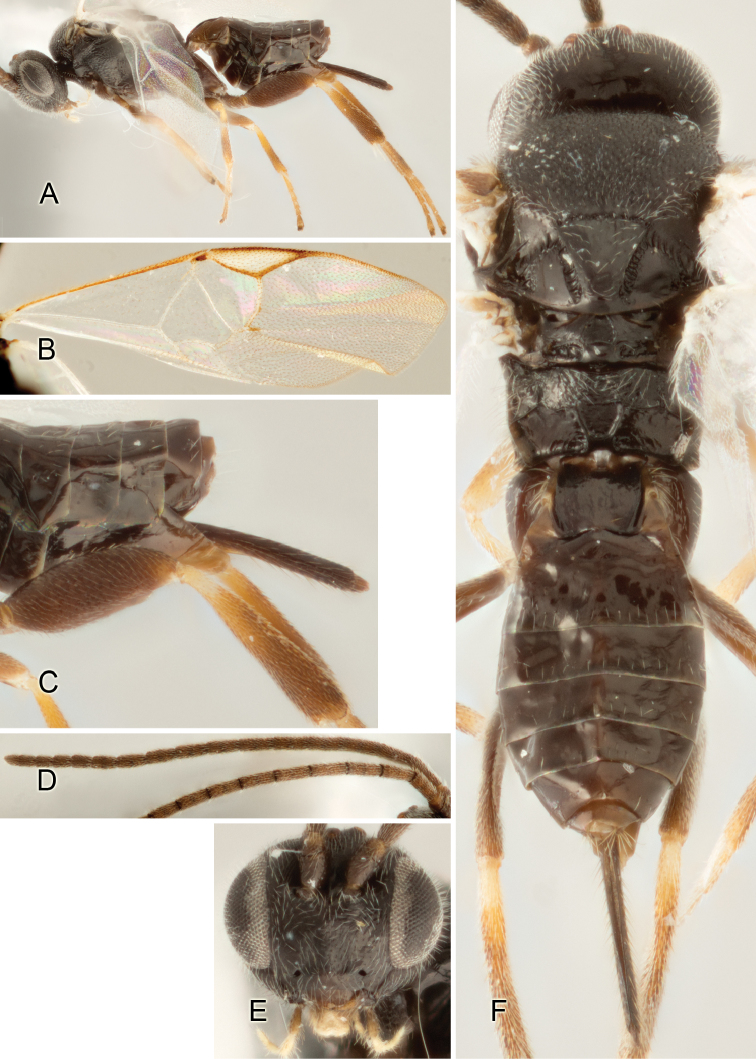
*Apanteles eugeniaphilipsae*. **A** Habitus, lateral view **B** Fore wing **C** Hypopygium and ovipositor sheats **D** Antenna **E** Head, frontal view **F** Head, meso- and metasoma, dorsal view.

**Figure 179.**
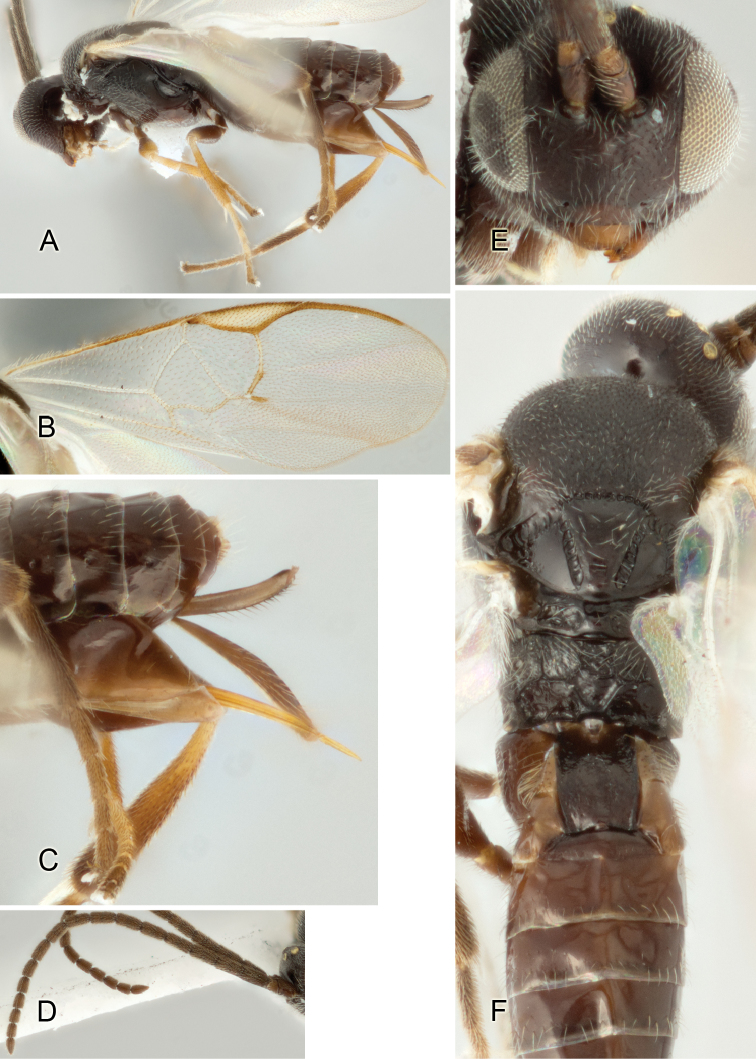
*Apanteles gerardobandoi*. **A** Habitus, lateral view **B** Fore wing **C** Hypopygium and ovipositor sheats **D** Antenna **E** Head, frontal view **F** Head, meso- and metasoma (partially), dorsal view.

**Figure 180.**
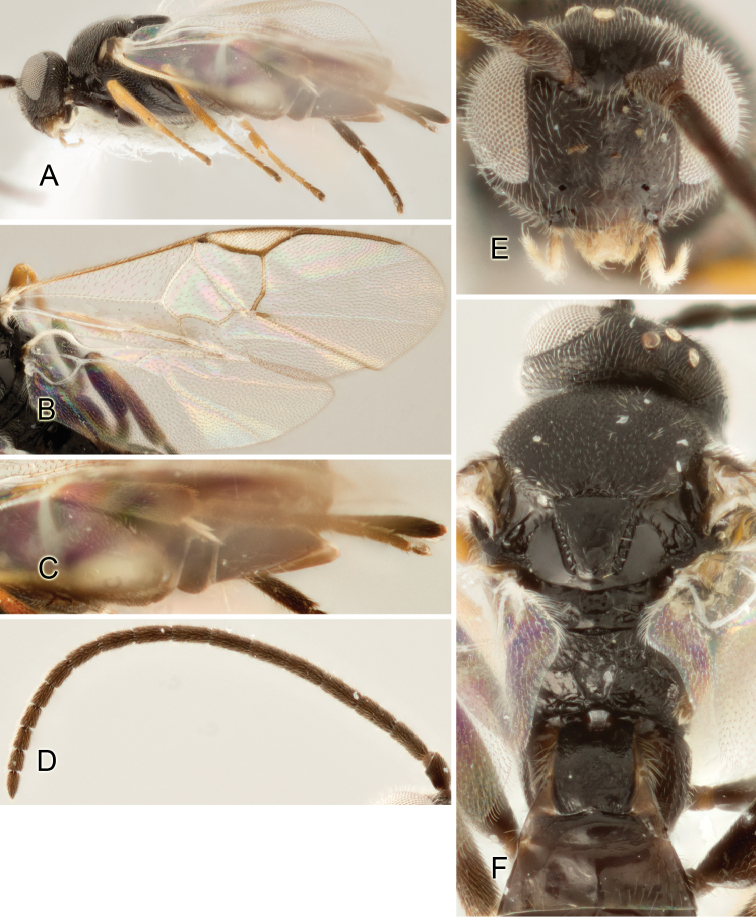
*Apanteles gladysrojasae*. **A** Habitus, lateral view **B** Fore wing **C** Hypopygium and ovipositor sheats **D** Antenna **E** Head, frontal view **F** Head, meso- and metasoma (partially), dorsal view.

**Figure 181.**
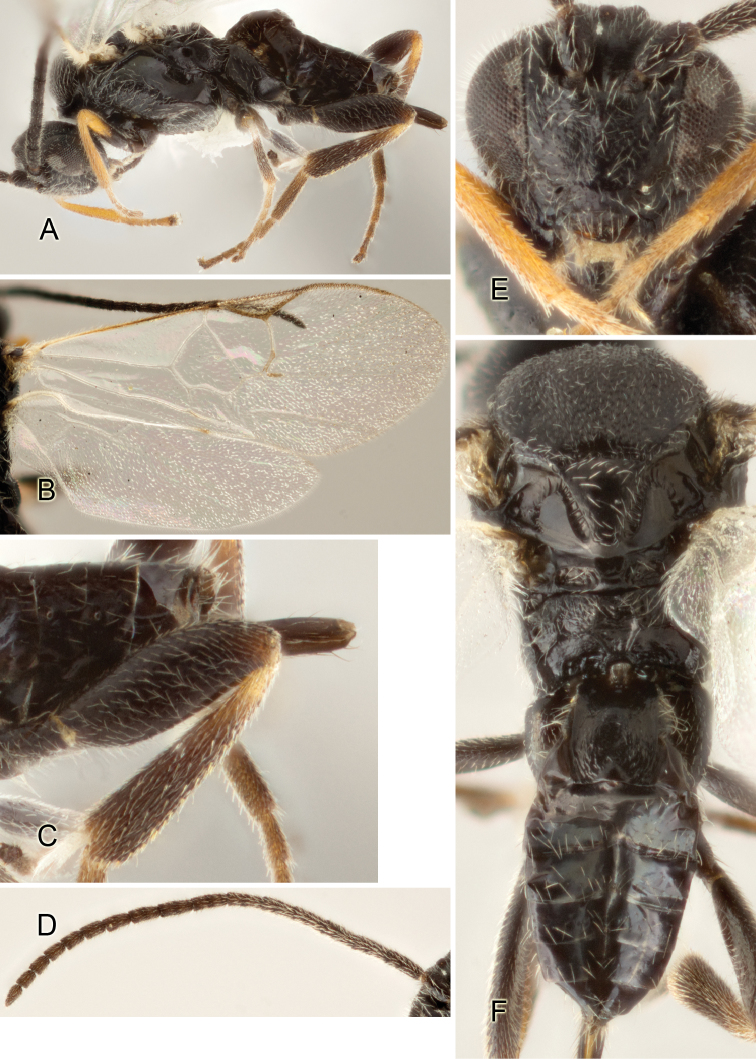
*Apanteles juanmatai*. **A** Habitus, lateral view **B** Fore wing **C** Hypopygium and ovipositor sheats **D** Antenna **E** Head, frontal view **F** Head, meso- and metasoma (partially), dorsal view.

**Figure 182.**
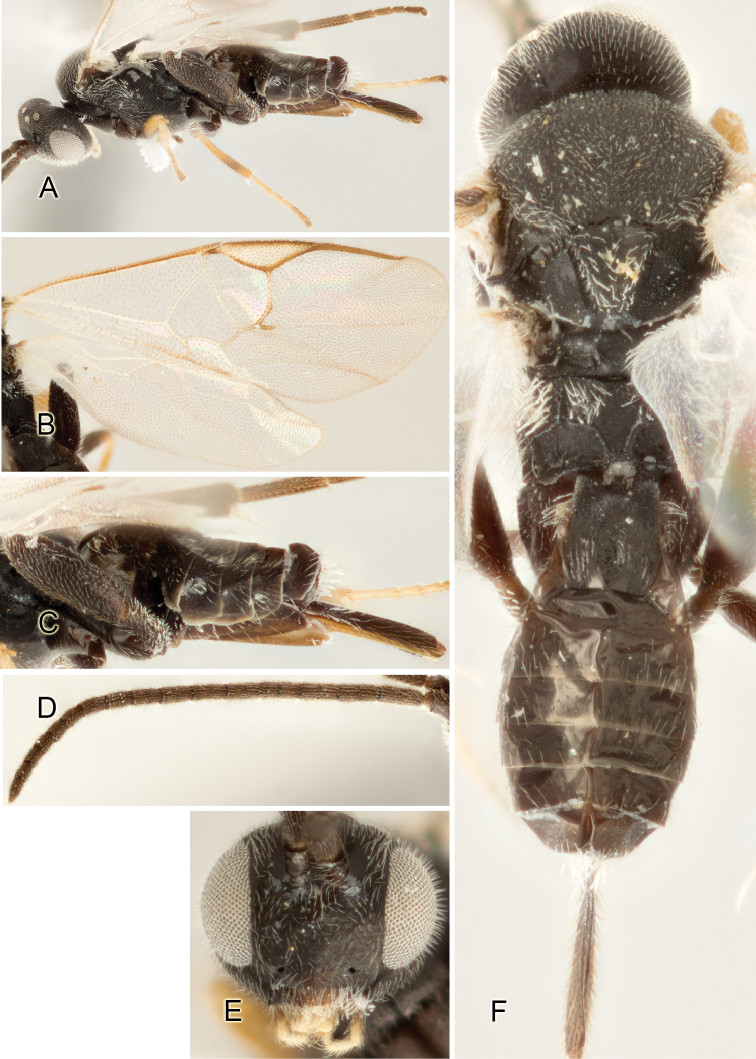
*Apanteles inesolisae*. **A** Habitus, lateral view **B** Fore wing **C** Hypopygium and ovipositor sheats **D** Antenna **E** Head, frontal view **F** Head, meso- and metasoma, dorsal view.

**Figure 183.**
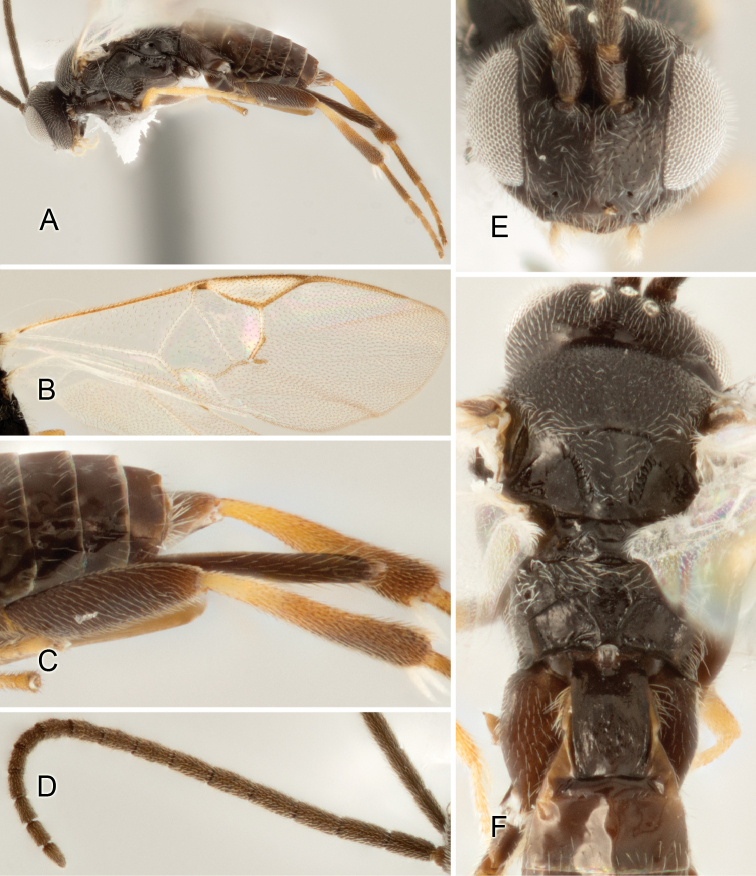
*Apanteles jesusugaldei*. **A** Habitus, lateral view **B** Fore wing **C** Hypopygium and ovipositor sheats **D** Antenna **E** Head, frontal view **F** Head, meso- and metasoma (partially), dorsal view.

**Figure 184.**
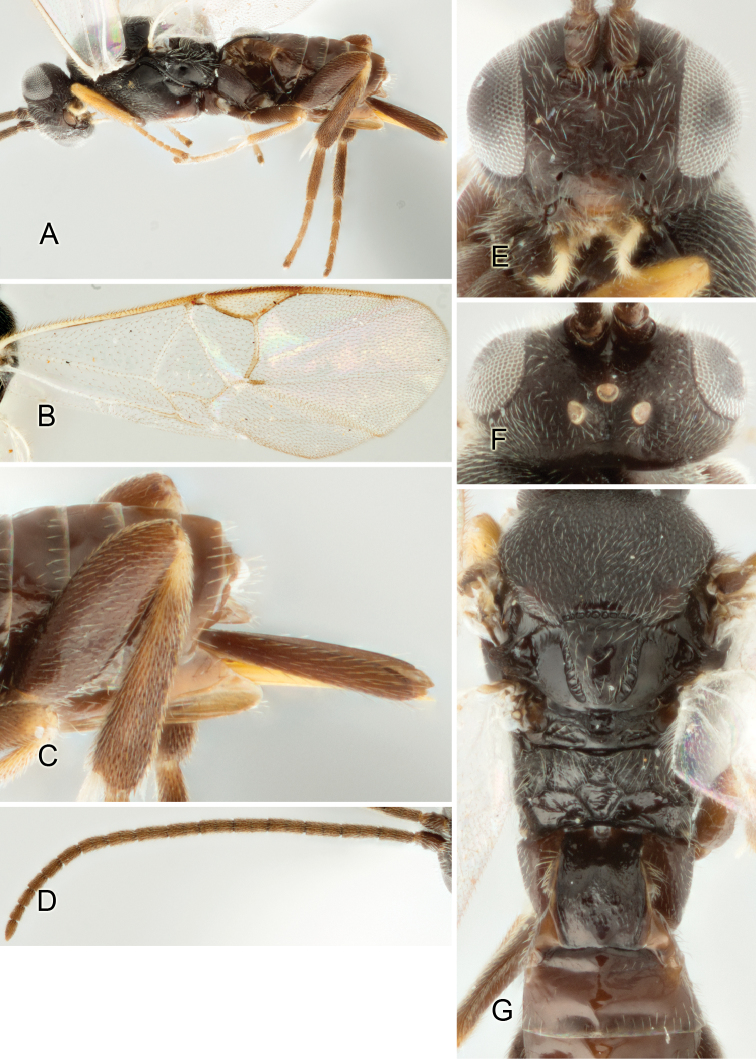
*Apanteles johanvargasi*. **A** Habitus, lateral view **B** Fore wing **C** Hypopygium and ovipositor sheats **D** Antenna **E** Head, frontal view **F** Head, dorsal view **G** Meso- and metasoma (partially), dorsal view.

**Figure 185.**
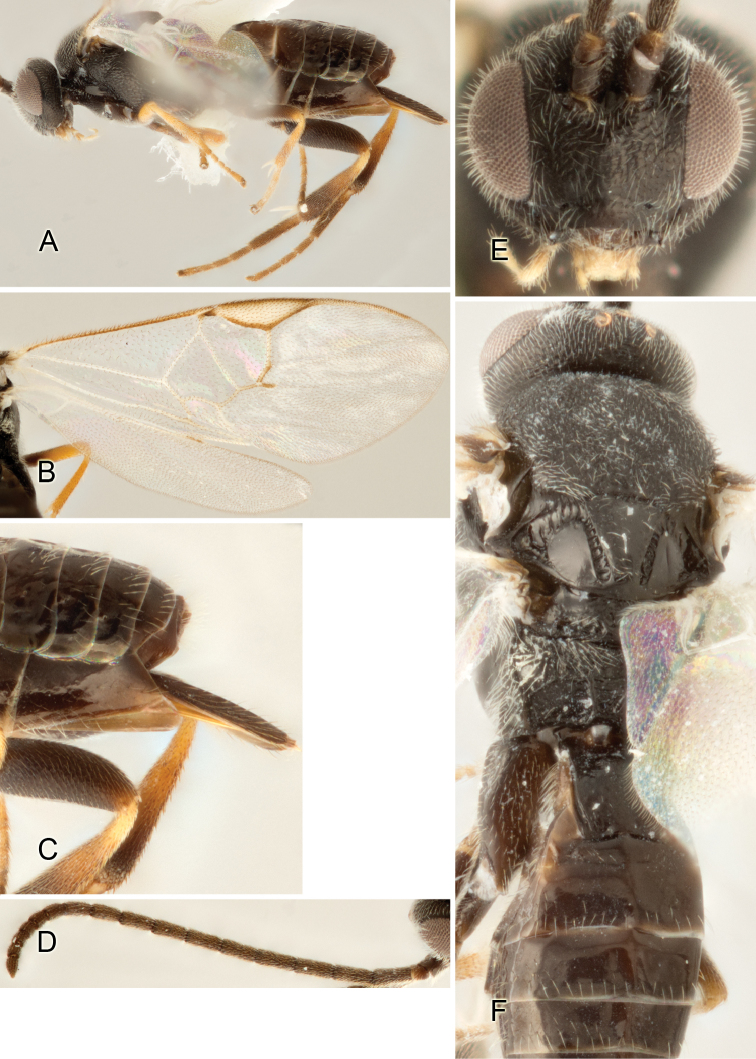
*Apanteles jorgehernandezi*. **A** Habitus, lateral view **B** Fore wing **C** Hypopygium and ovipositor sheats **D** Antenna **E** Head, frontal view **F** Head, meso- and metasoma (partially), dorsal view.

**Figure 186.**
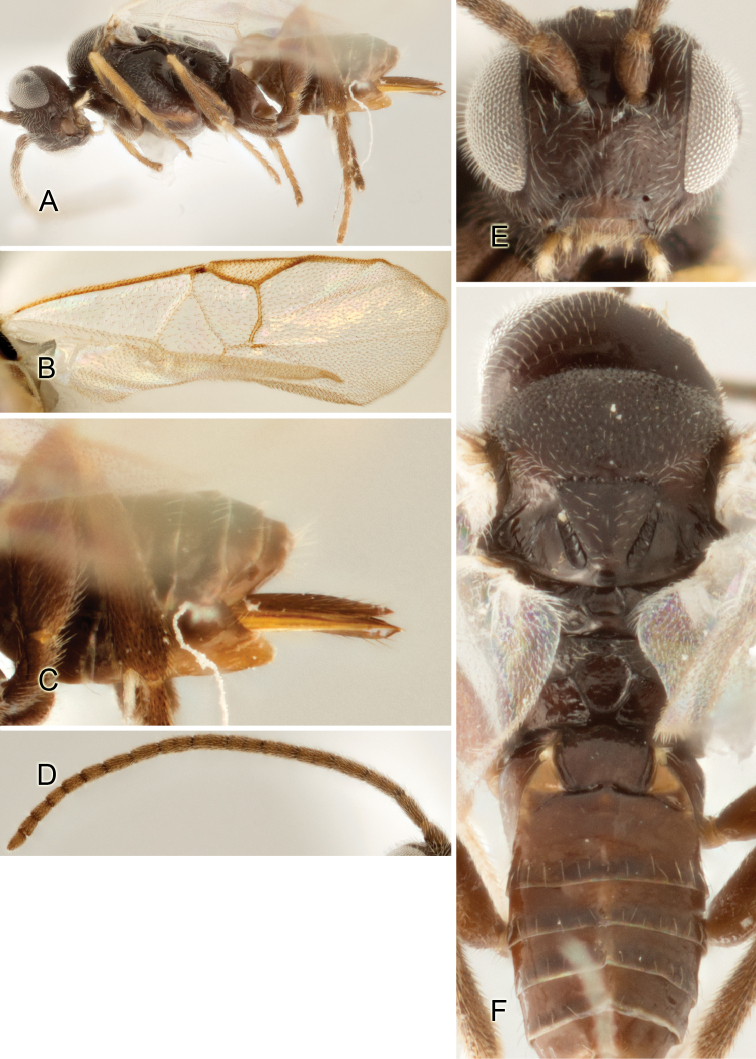
*Apanteles josecortesi*. **A** Habitus, lateral view **B** Fore wing **C** Hypopygium and ovipositor sheats **D** Antenna **E** Head, frontal view **F** Head, meso- and metasoma (partially), dorsal view.

**Figure 187.**
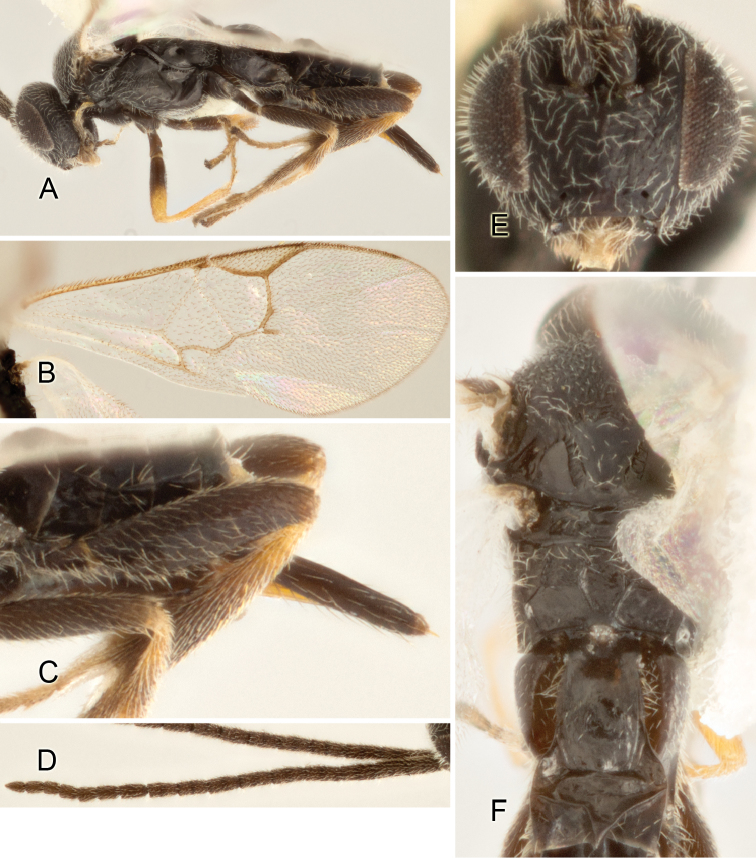
*Apanteles josemonteroi*. **A** Habitus, lateral view **B** Fore wing **C** Hypopygium and ovipositor sheats **D** Antenna **E** Head, frontal view **F** Head, meso- and metasoma (partially), dorsal view.

**Figure 188.**
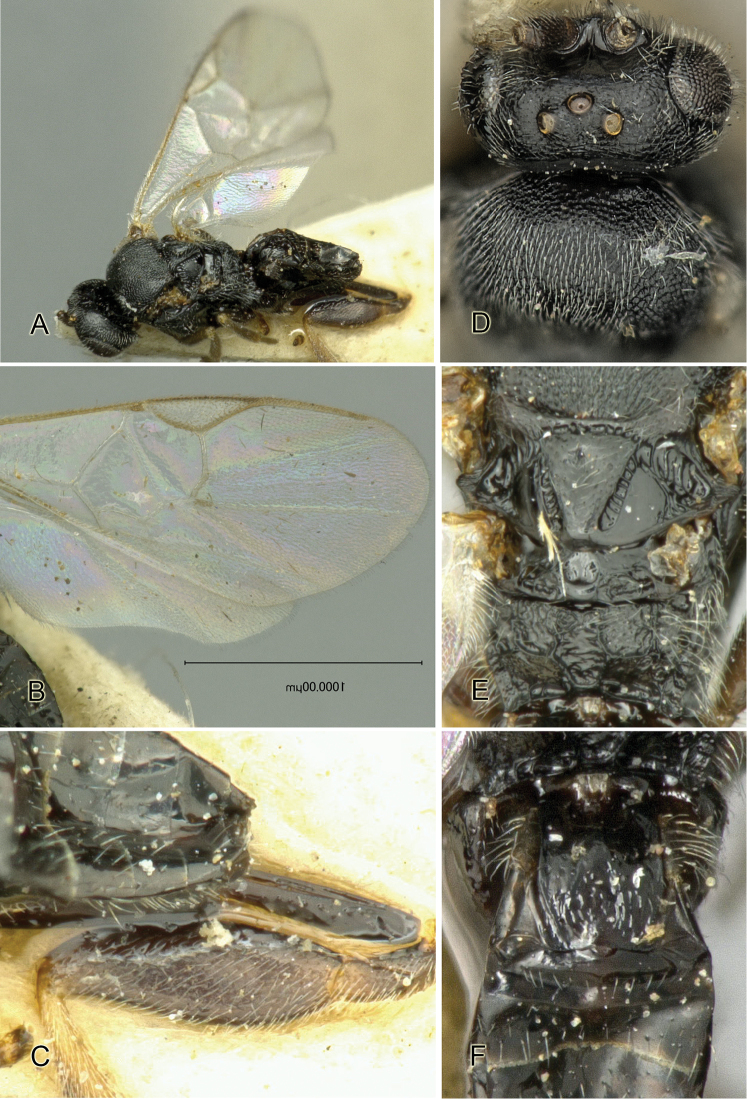
*Apanteles leucostigmus*. **A** Habitus, lateral view **B** Fore wing **C** Hypopygium and ovipositor sheats **D** Head and anteromesoscutum, dorsal view **E** Mesosoma (partially), dorsal view **F** Metasoma (partially), dorsal view.

**Figure 189.**
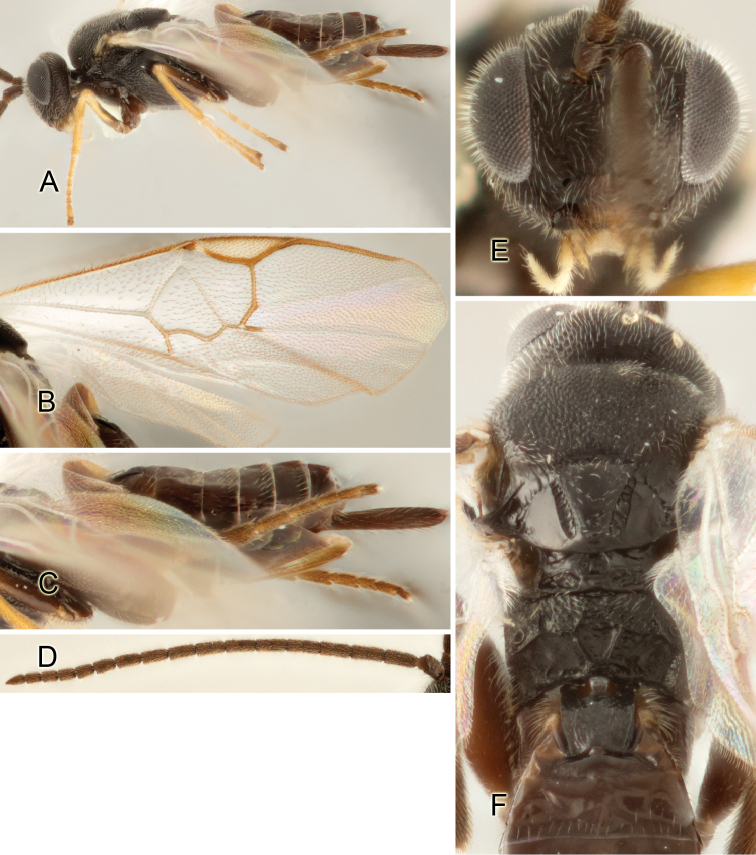
*Apanteles lilliammenae*. **A** Habitus, lateral view **B** Fore wing **C** Hypopygium and ovipositor sheats **D** Antenna **E** Head, frontal view **F** Head, meso- and metasoma (partially), dorsal view.

**Figure 190.**
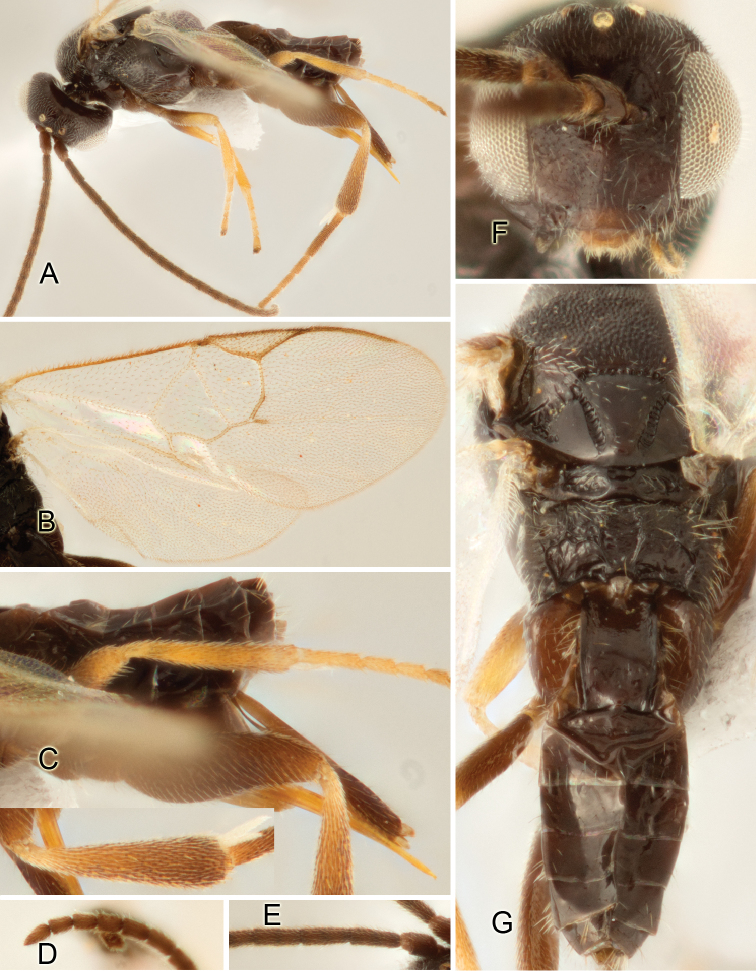
*Apanteles manuelzumbadoi*. **A** Habitus, lateral view **B** Fore wing **C** Hypopygium and ovipositor sheats, with details of metatibia **D** Posterior half of antenna **E** Anterior half of antenna **F** Head, frontal view **G** Meso- and metasoma, dorsal view.

**Figure 191.**
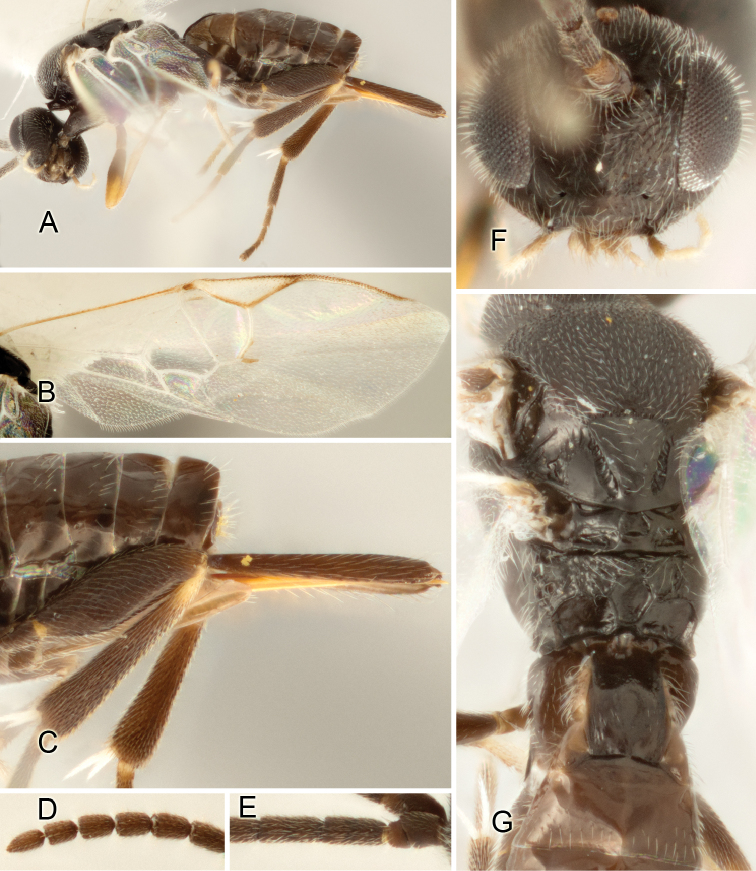
*Apanteles marcovenicioi*. **A** Habitus, lateral view **B** Fore wing **C** Hypopygium and ovipositor sheats **D** Posterior half of antenna **E** Anterior half of antenna **F** Head, frontal view **G** Meso- and metasoma (partially), dorsal view.

**Figure 192.**
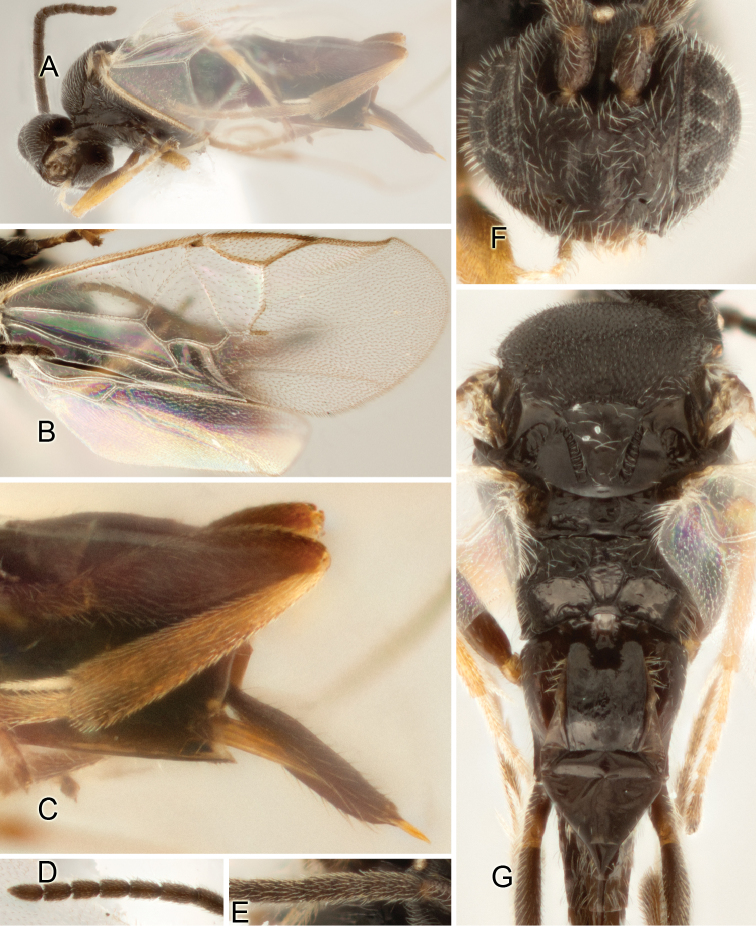
*Apanteles sigifredomarini*. **A** Habitus, lateral view **B** Fore wing **C** Hypopygium and ovipositor sheats **D** Posterior half of antenna **E** Anterior half of antenna **F** Head, frontal view **G** Meso- and metasoma (partially), dorsal view.

**Figure 193.**
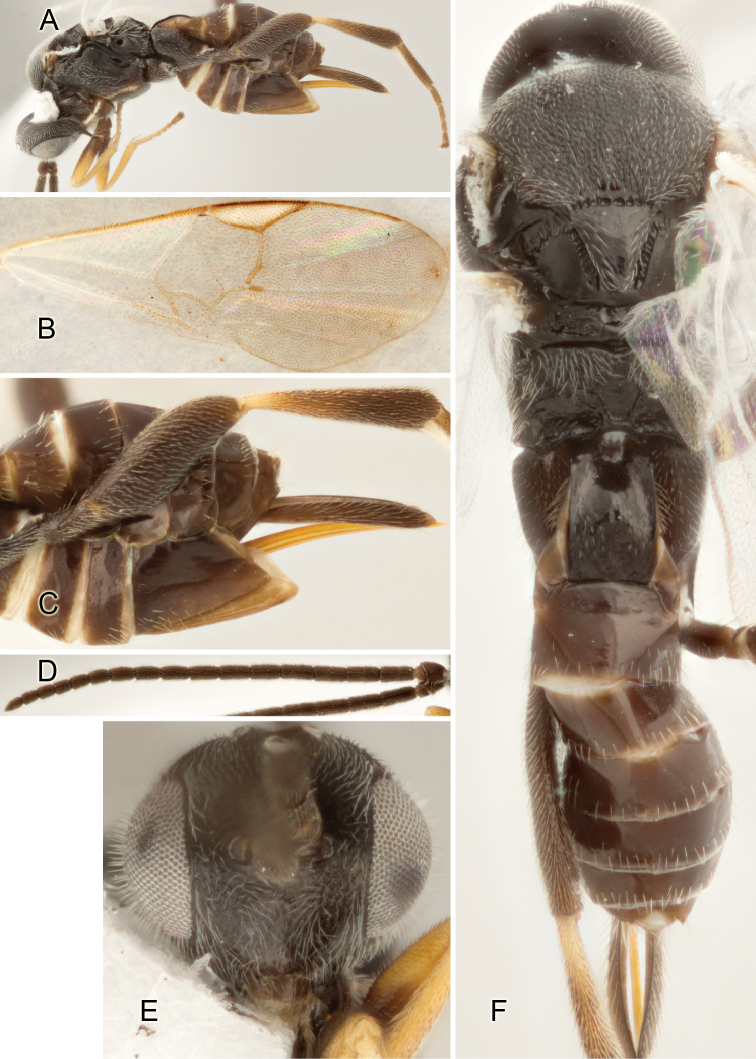
*Apanteles pabloumanai*. **A** Habitus, lateral view **B** Fore wing **C** Hypopygium and ovipositor sheats **D** Antenna **E** Head, frontal view **F** Head, meso- and metasoma, dorsal view.

**Figure 194.**
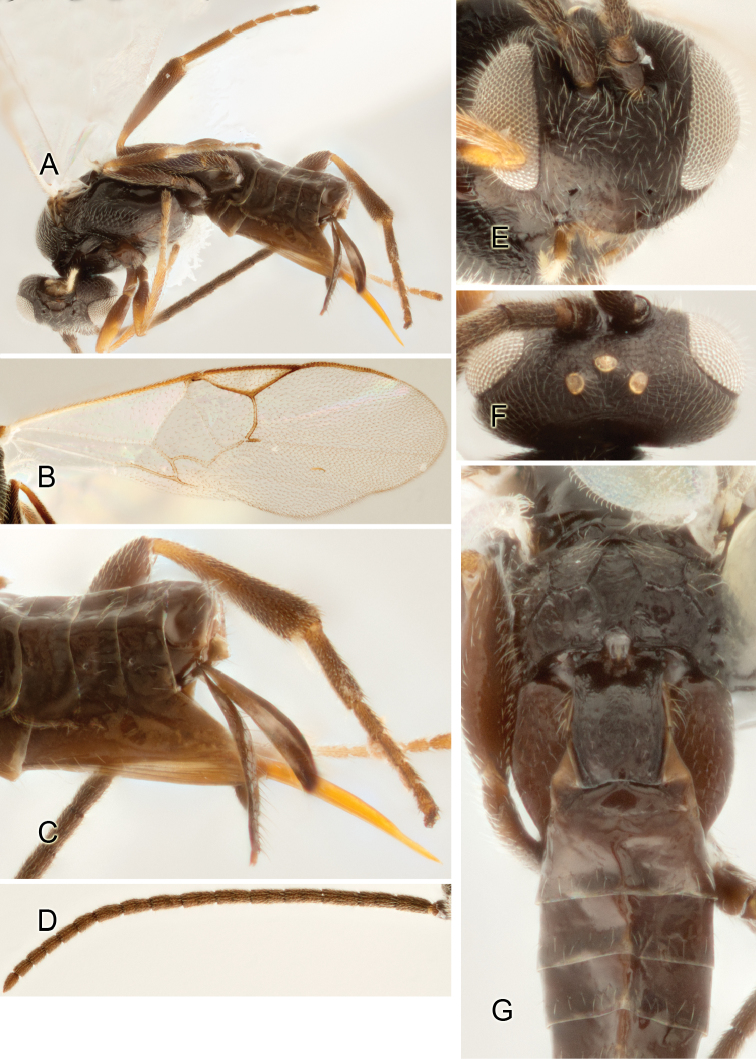
*Apanteles luzmariaromeroae*. **A** Habitus, lateral view **B** Fore wing **C** Hypopygium and ovipositor sheats **D** Antenna **E** Head, frontal view **F** Head, dorsal view **G** Meso- and metasoma (partially), dorsal view.

**Figure 195.**
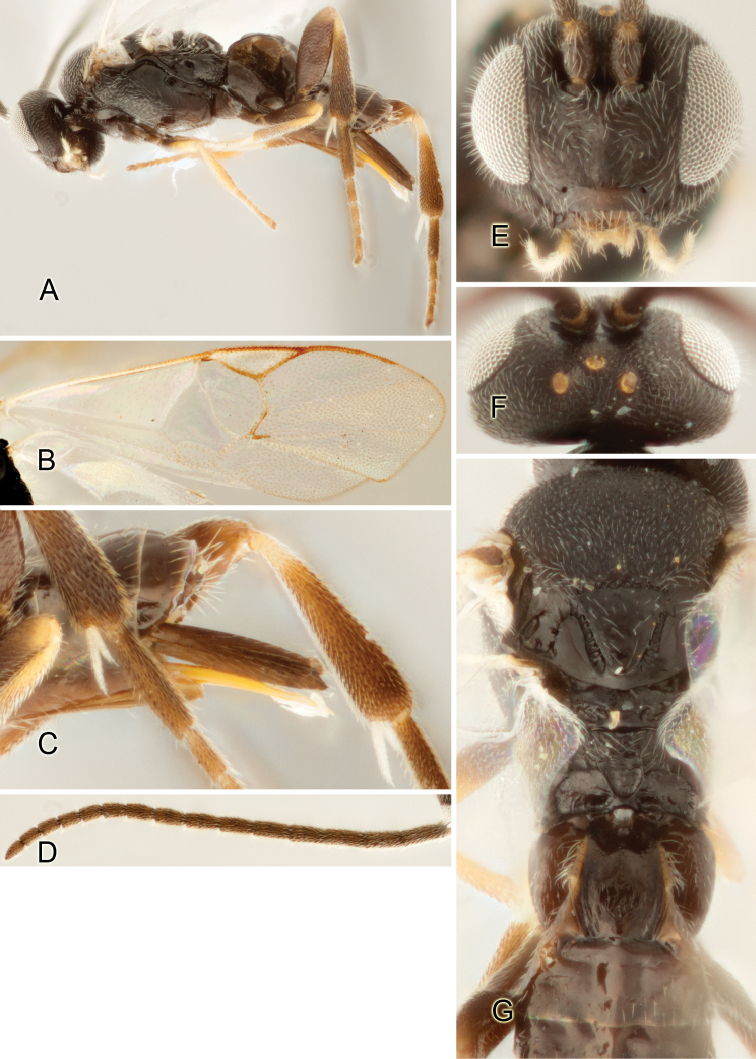
*Apanteles randallgarciai*. **A** Habitus, lateral view **B** Fore wing **C** Hypopygium and ovipositor sheats **D** Antenna **E** Head, frontal view **F** Head, dorsal view **G** Meso- and metasoma (partially), dorsal view.

**Figure 196.**
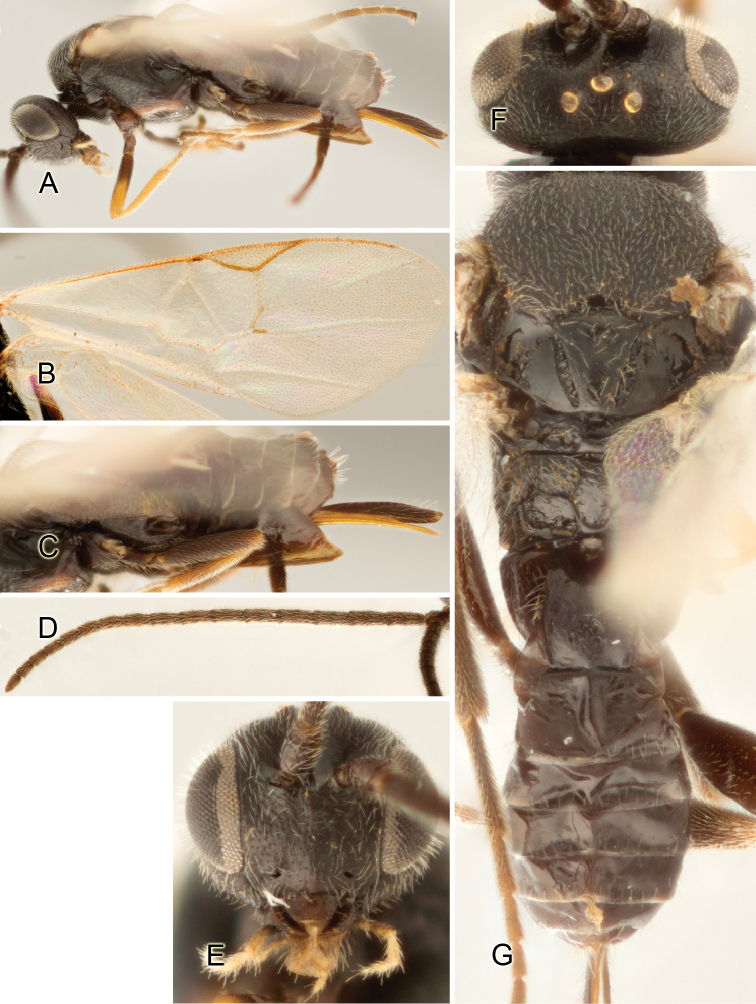
*Apanteles raulsolorsanoi*. **A** Habitus, lateral view **B** Fore wing **C** Hypopygium and ovipositor sheats **D** Antenna **E** Head, frontal view **F** Head, dorsal view **G** Meso- and metasoma, dorsal view.

**Figure 197.**
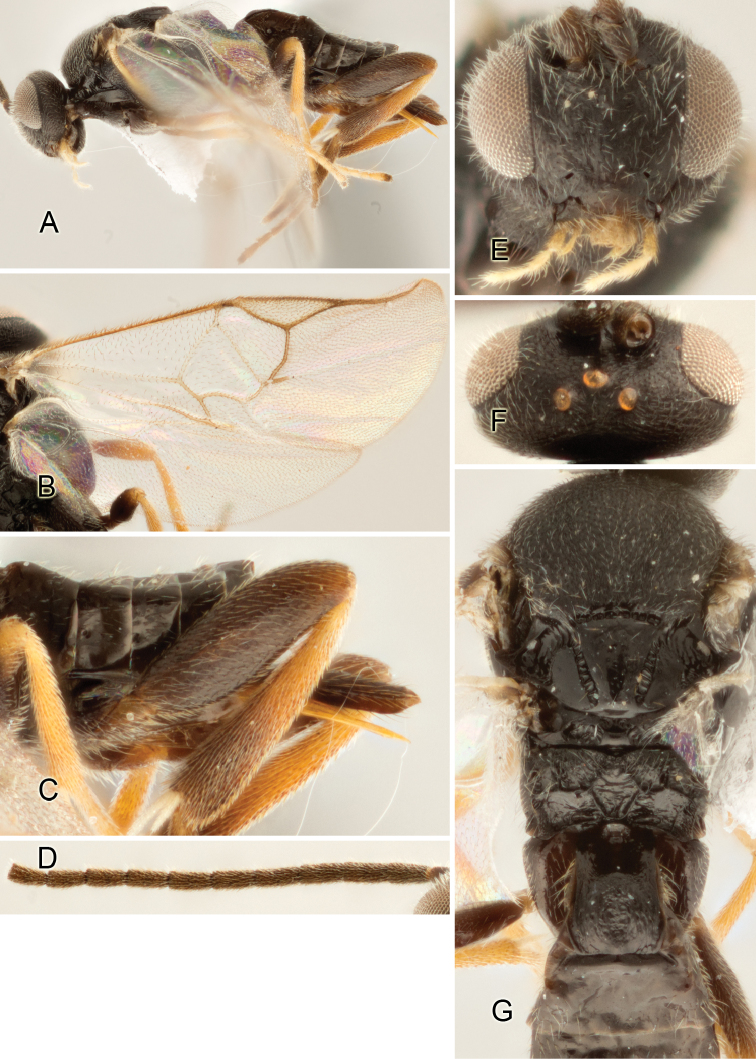
*Apanteles wadyobandoi*. **A** Habitus, lateral view **B** Fore wing **C** Hypopygium and ovipositor sheats **D** Antenna **E** Head, frontal view **F** Head, dorsal view **G** Meso- and metasoma (partially), dorsal view.

**Figure 198.**
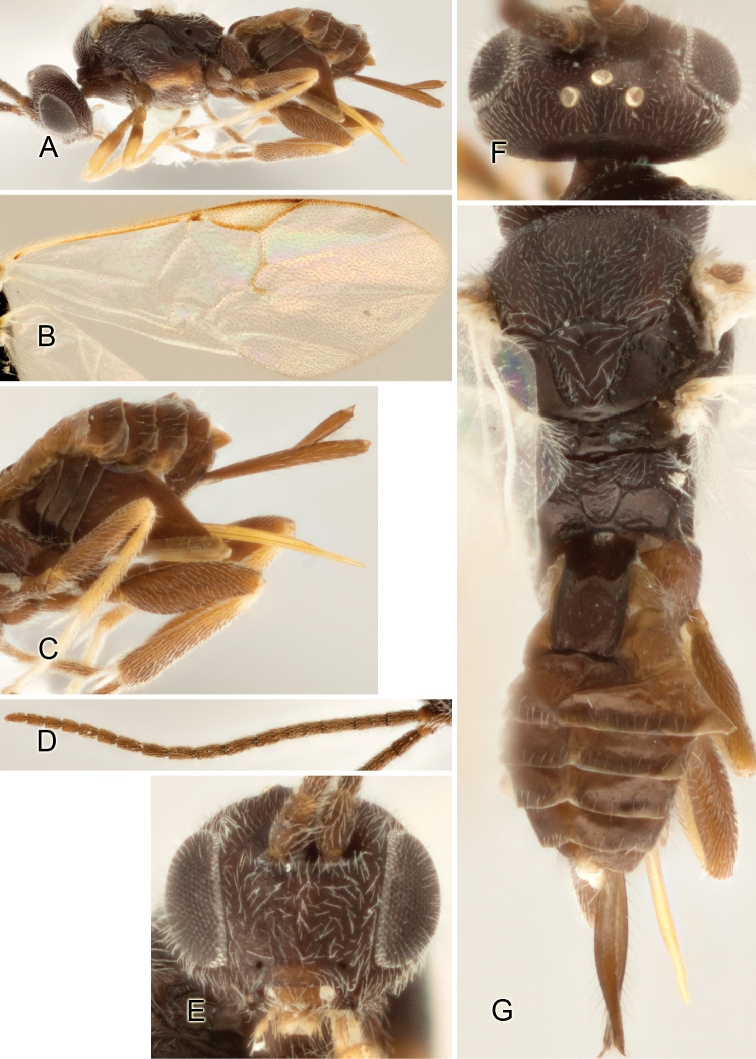
*Apanteles ricardocaleroi*. **A** Habitus, lateral view **B** Fore wing **C** Hypopygium and ovipositor sheats **D** Antenna **E** Head, frontal view **F** Head, dorsal view **G** Meso- and metasoma, dorsal view.

**Figure 199.**
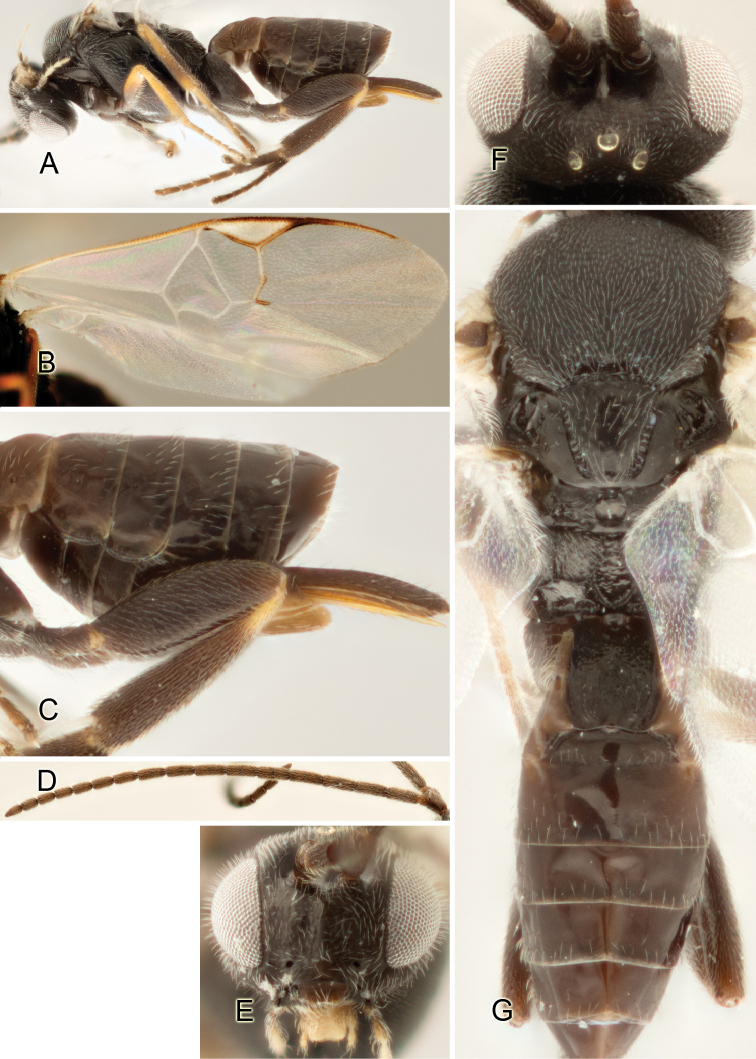
*Apanteles rodrigogamezi*. **A** Habitus, lateral view **B** Fore wing **C** Hypopygium and ovipositor sheats **D** Antenna **E** Head, frontal view **F** Head, dorsal view **G** Meso- and metasoma, dorsal view.

**Figure 200.**
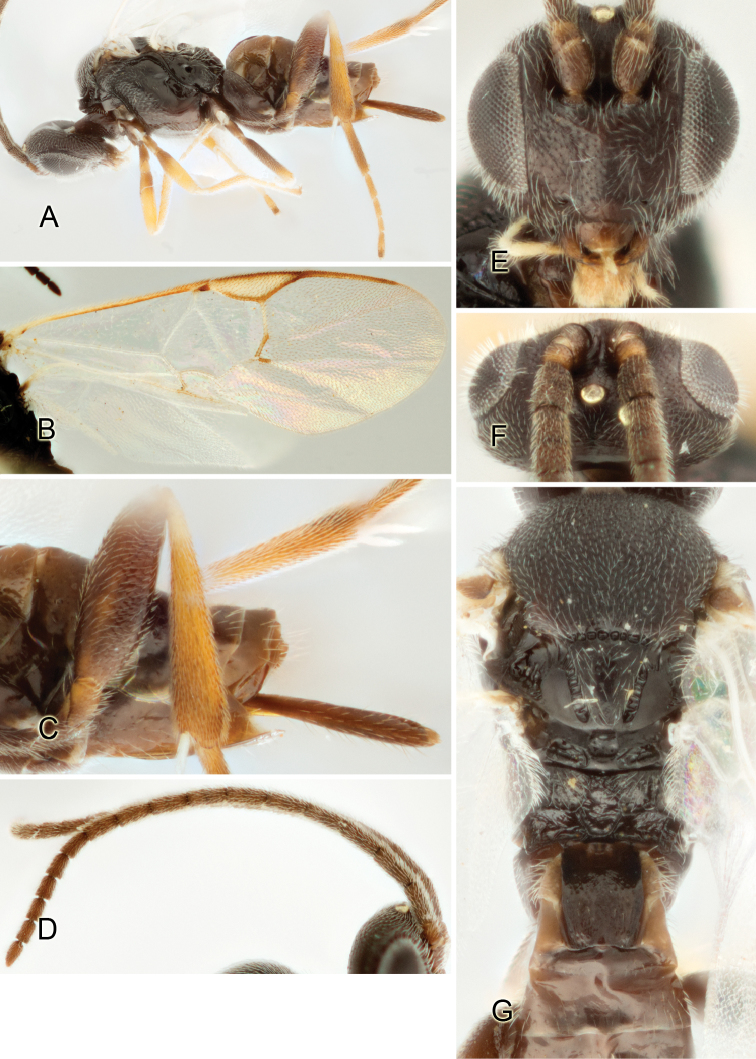
*Apanteles ronaldzunigai*. **A** Habitus, lateral view **B** Fore wing **C** Hypopygium and ovipositor sheats **D** Antenna **E** Head, frontal view **F** Head, dorsal view **G** Meso- and metasoma (partially), dorsal view.

**Figure 201.**
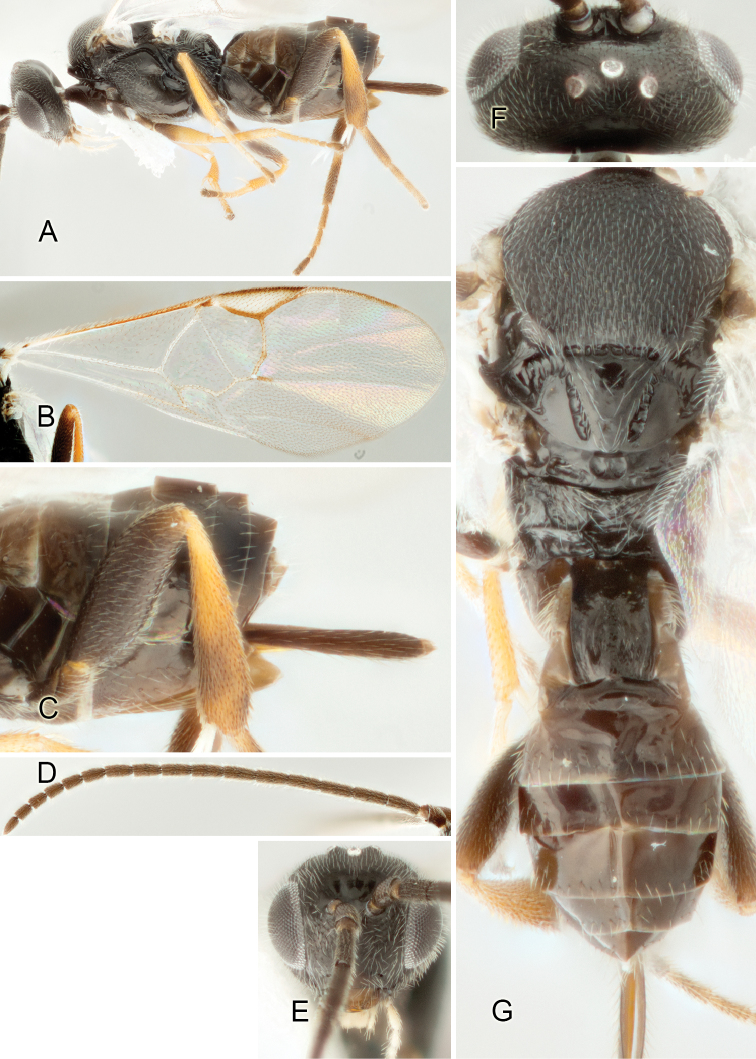
*Apanteles sergioriosi*. **A** Habitus, lateral view **B** Fore wing **C** Hypopygium and ovipositor sheats **D** Antenna **E** Head, frontal view **F** Head, dorsal view **G** Meso- and metasoma, dorsal view.

**Figure 202.**
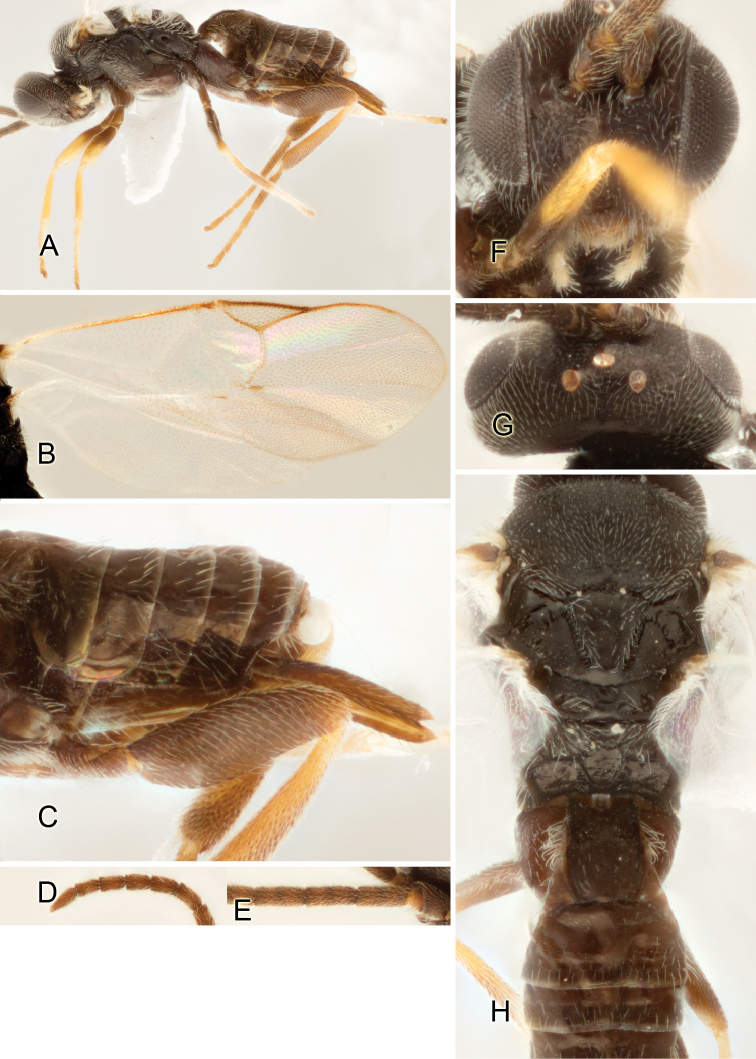
*Apanteles hazelcambroneroae*. **A** Habitus, lateral view **B** Fore wing **C** Hypopygium and ovipositor sheats **D** Posterior half of antenna **E** Anterior half of antenna **F** Head, frontal view **G** Head, dorsal view **H** Meso- and metasoma (partially), dorsal view.

**Figure 203.**
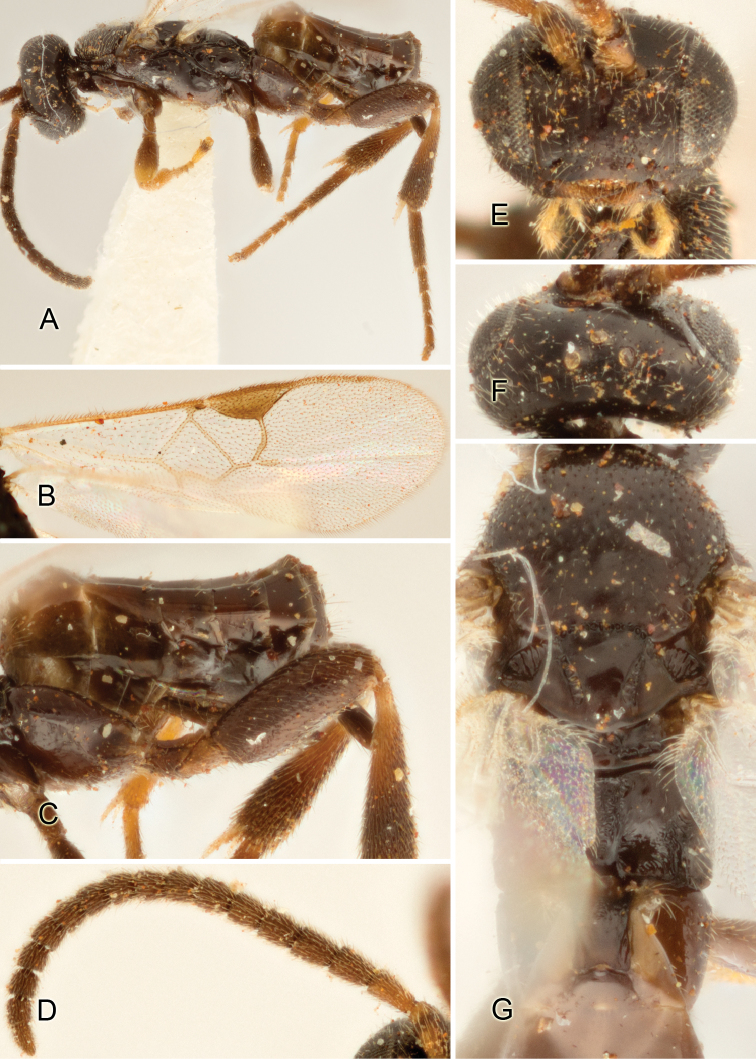
*Apanteles deplanatus*. **A** Habitus, lateral view **B** Fore wing **C** Metasoma, lateral view **D** Antenna **E** Head, frontal view **F** Head, dorsal view **G** Meso- and metasoma (partially), dorsal view.

**Figure 204.**
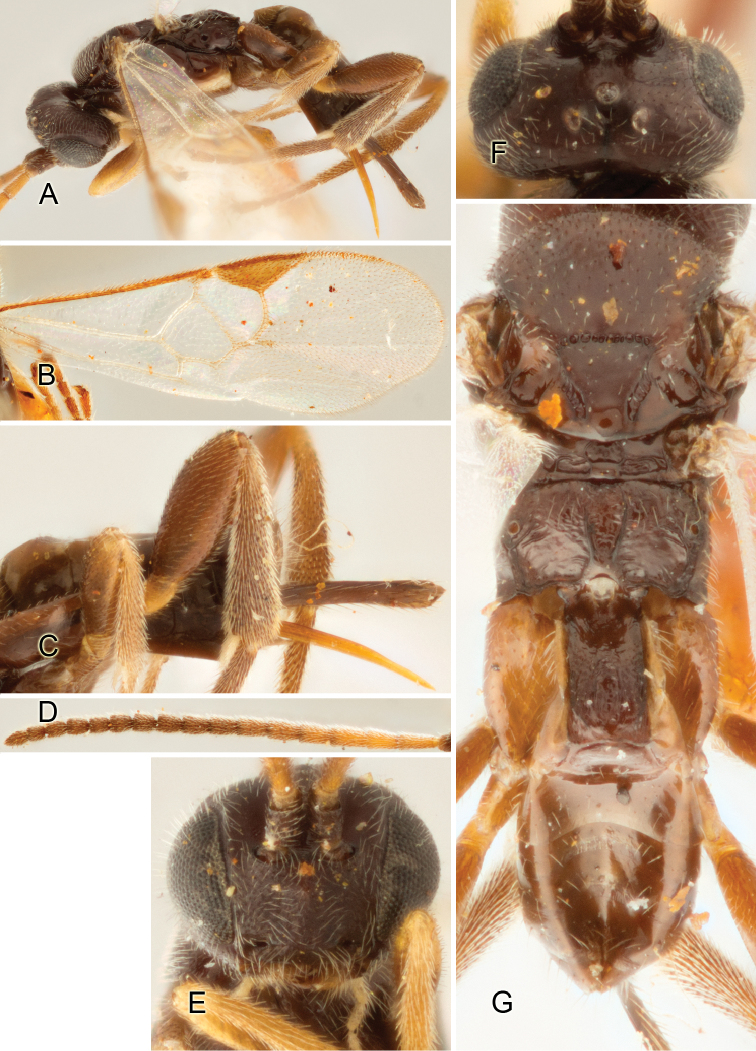
*Apanteles diatraeae*. **A** Habitus, lateral view **B** Fore wing **C** Hypopygium and ovipositor sheats **D** Antenna **E** Head, frontal view **F** Head, dorsal view **G** Meso- and metasoma, dorsal view.

**Figure 205.**
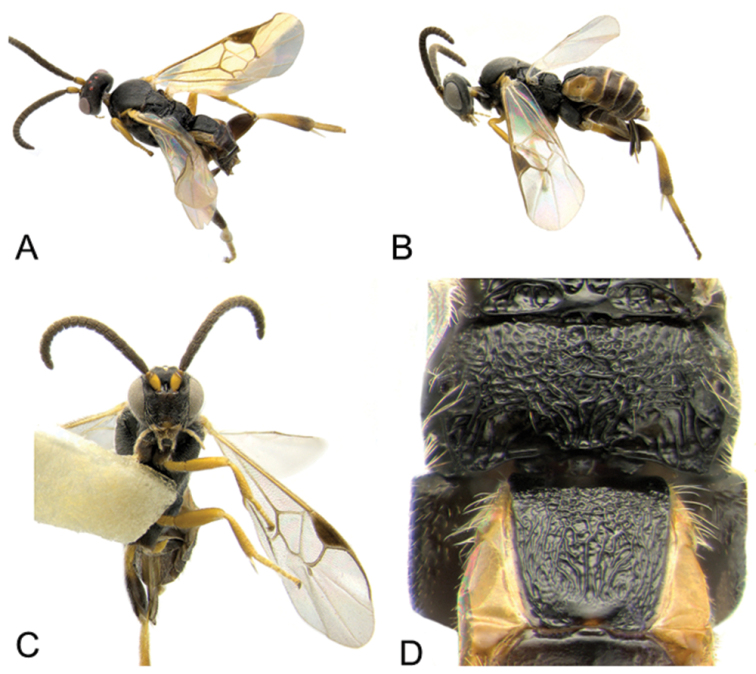
*Apanteles samarshalli*. **A** Habitus, lateral view **B** Habitus, lateral view **C** Habitus, ventral view **D** Propodeum and mediotergites 1-2, dorsal view.

**Figure 206.**
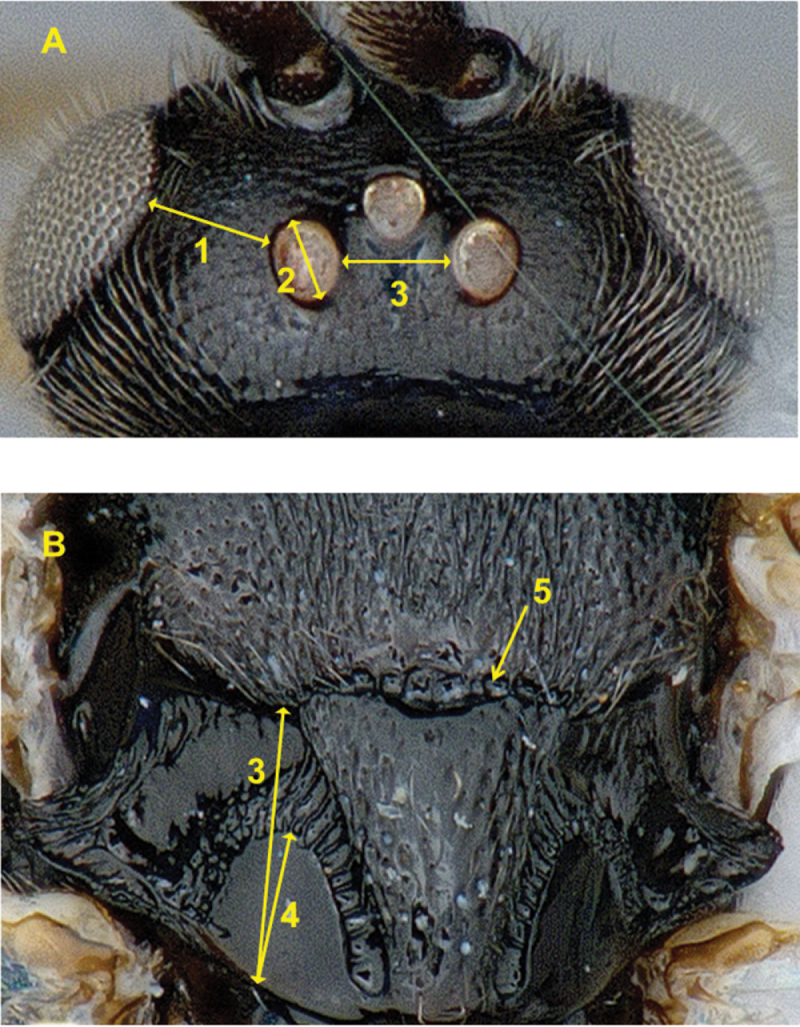
General morphology of *Apanteles* **A** Head, dorsal view. B-Mesosoma (in part), dorsal view. **1** Ocular–ocellar line **2** Posterior ocellus diameter **3** Interocellar distance **4** Maximum height of mesoscutellum lunules **5** Maximum height of lateral face of mesoscutellum **6** Scutoscutellar sulcus.

**Figure 207.**
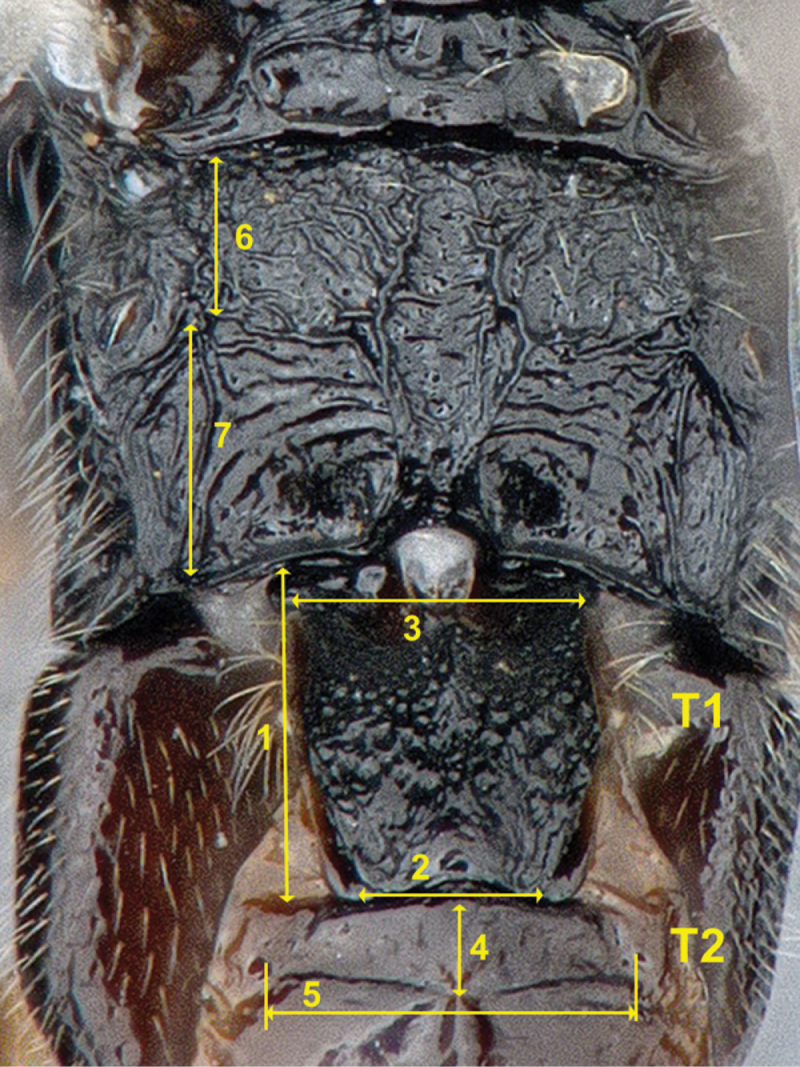
General morphology of *Apanteles*. **T1** Mediotergite **1** dorsal view **T2** Mediotergite 2, dorsal view **1** Mediotergite 1 length **2** Width at posterior margin **3** Width at anterior margin **4** Mediotergite 2 length **5** Mediotergite 2 width at posterior margin **6** Anterior half of proppdeum **7** Posterior half of propodeum.

**Figure 208.**
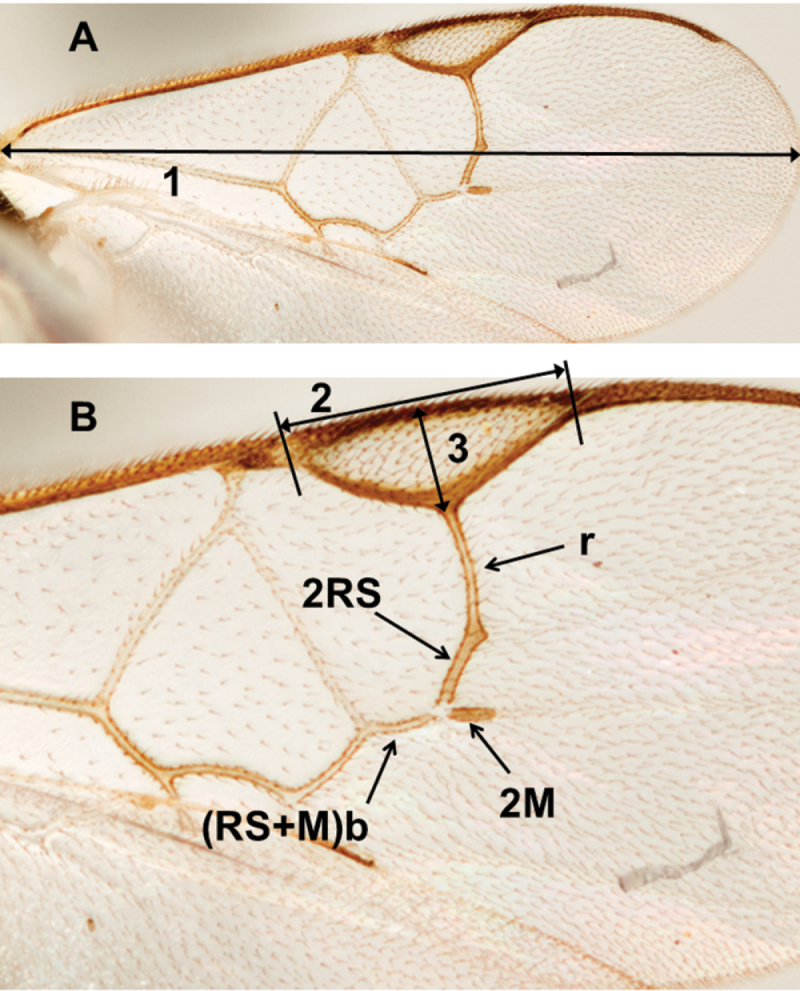
General morphology of *Apanteles* **A** Fore wing **B** Details of central area of fore wing. **1** Fore wing length **2** Pterostigma length **3** Pterostigma width. Fore wing veins showed with arrows.

**Figure 209.**
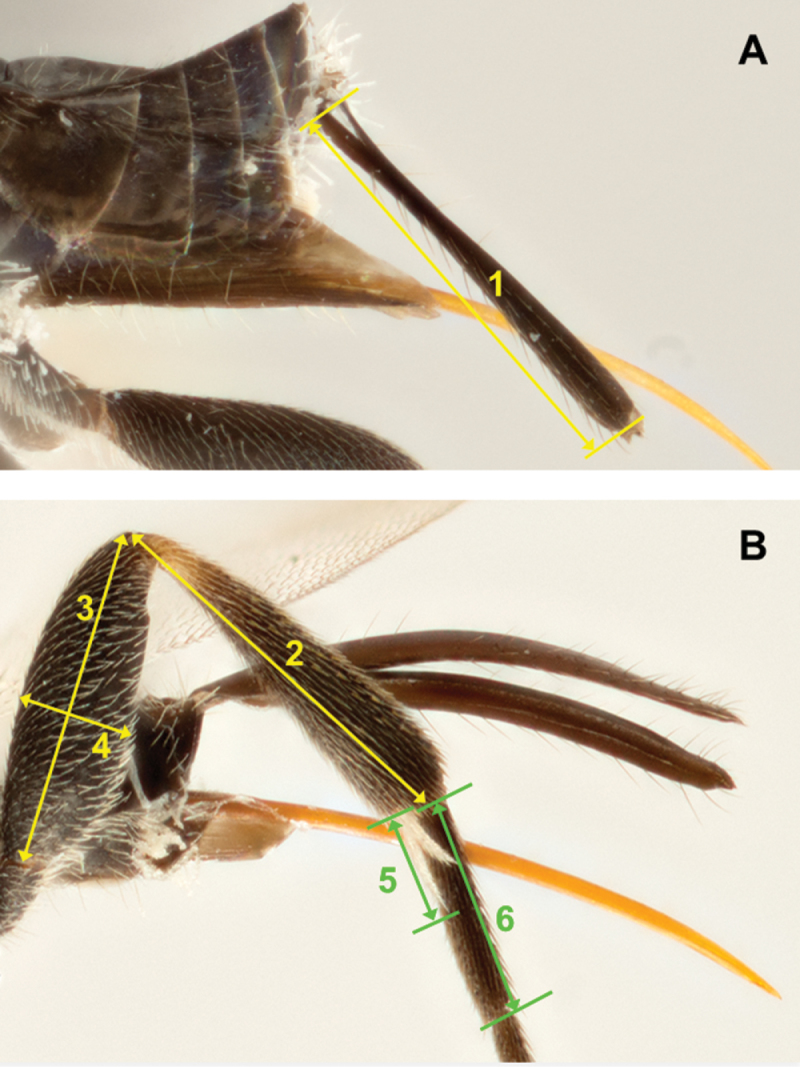
General morphology of *Apanteles* **A** Metasoma and ovipositor, lateral view **B** Details of hind leg, lateral view. **1** Ovipositor sheaths length **2** Metatibia length **3** Mesofemur length **4** Mesofemur width **5** Inner metatibia spur length **6** Metabasitarsus length.

**Figures 210–217.**
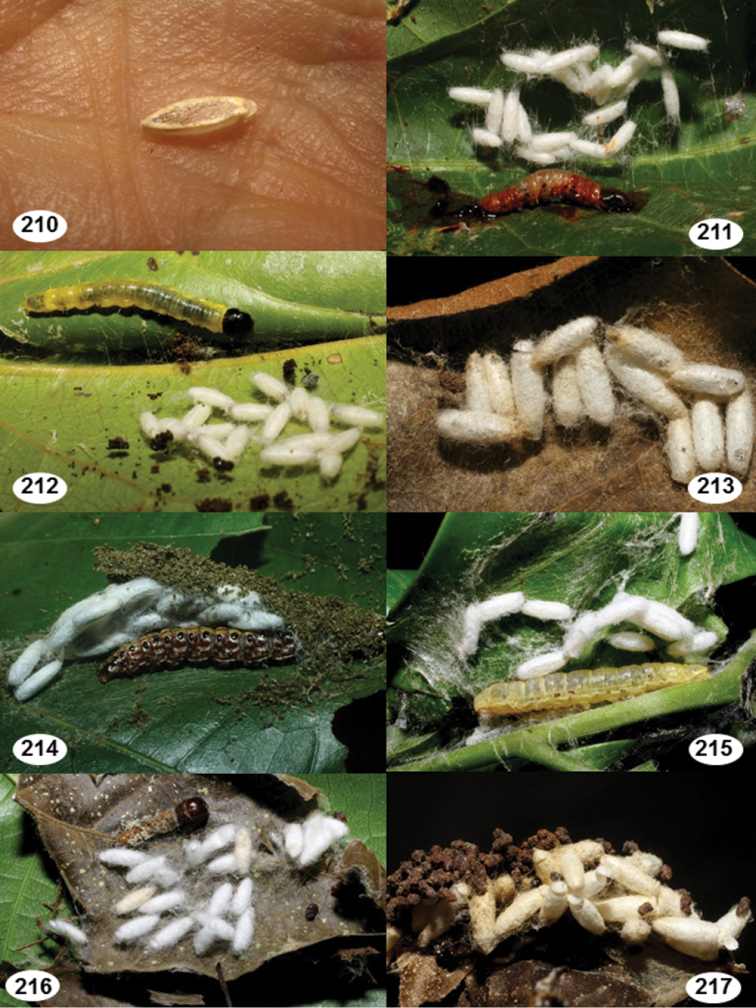
Cocoons of *Apanteles* species of Area de Conservación de Guanacaste. **210** *Apanteles adelinamoralesae* **211** *Apanteles carloscastilloi* **212** *Apanteles didiguadamuzi* **213** *Apanteles jorgecortesi* **214** *Apanteles juanvictori* **215** *Apanteles juniorlopezi* **216** *Apanteles leninguadamuzi* **217** *Apanteles luiscanalesi*.

**Figures 218–225.**
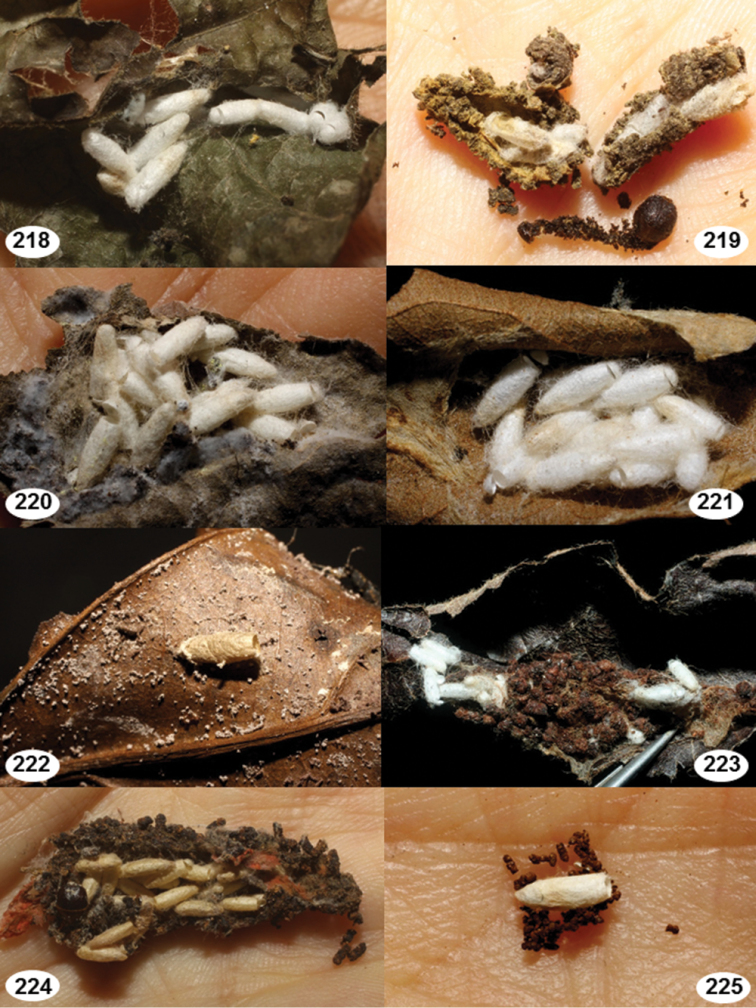
Cocoons of *Apanteles* species of Area de Conservación de Guanacaste. **218** *Apanteles luislopezi* **219** *Apanteles manuelarayai* **220** *Apanteles paulaixcamparijae* **221** *Apanteles ronaldmurilloi* **222** *Apanteles wilbertharayai* **223** *Apanteles yolandarojasae* **224** *Apanteles zeneidabolanosae* **225** *Apanteles adrianachavarriae*.

**Figures 226–233.**
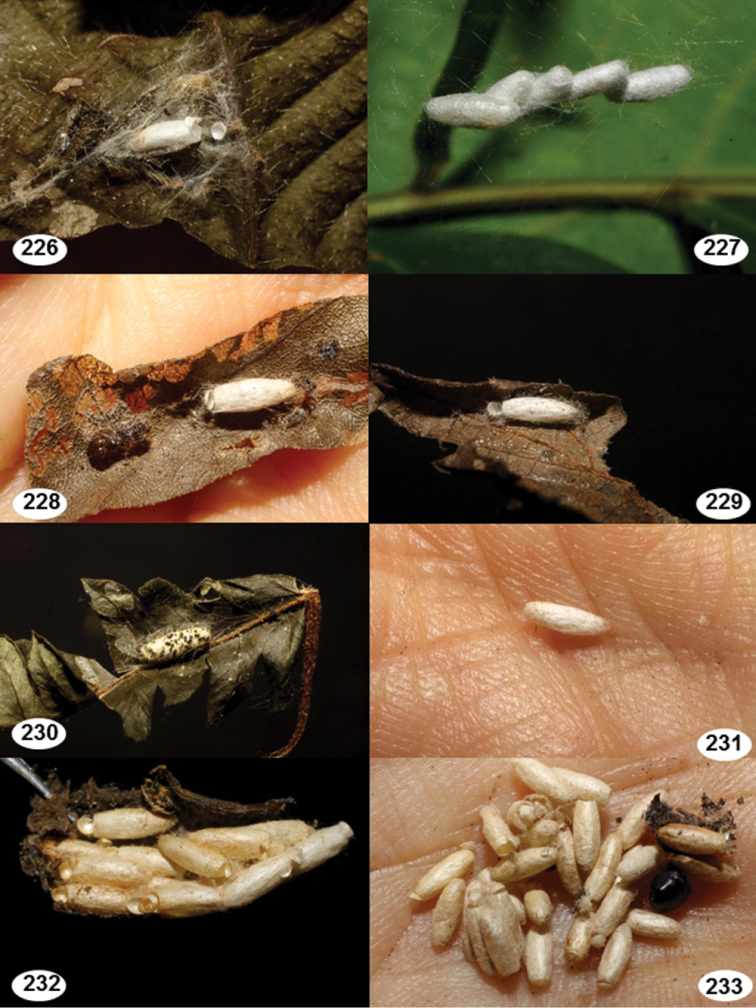
Cocoons of *Apanteles* species of Area de Conservación de Guanacaste. **226** *Apanteles adrianguadamuzi* **227** *Apanteles anamartinezae* **228** *Apanteles felipechavarriai* **229** *Apanteles irenecarrilloi* **230** *Apanteles luiscantillanoi* **231** *Apanteles yilbertalvaradoi* **232** *Apanteles adrianaguilarae* **233** *Apanteles vannesabrenesae*.

**Figures 234–241.**
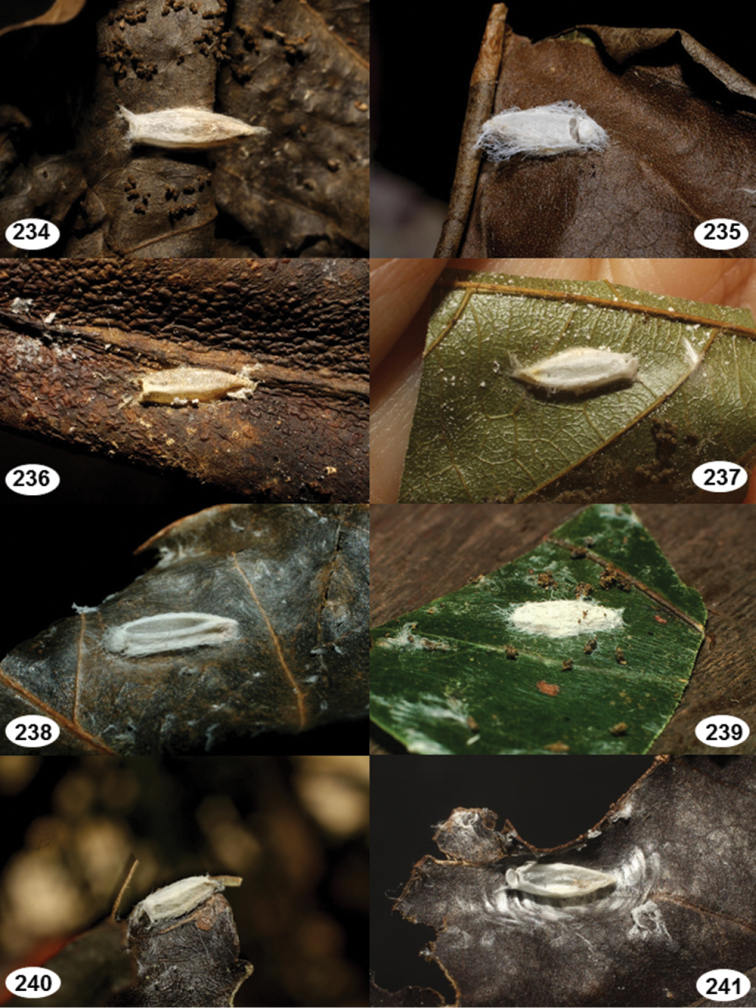
Cocoons of *Apanteles* species of Area de Conservación de Guanacaste. **234** *Apanteles alejandromorai* **235** *Apanteles deifiliadavilae* **236** *Apanteles eulogiosequeirai* **237** *Apanteles fernandochavarriai* **238** *Apanteles gabrielagutierrezae* **239** *Apanteles juancarrilloi* **240** *Apanteles luisgarciai* **241** *Apanteles marvinmendozai*.

**Figures 242–249.**
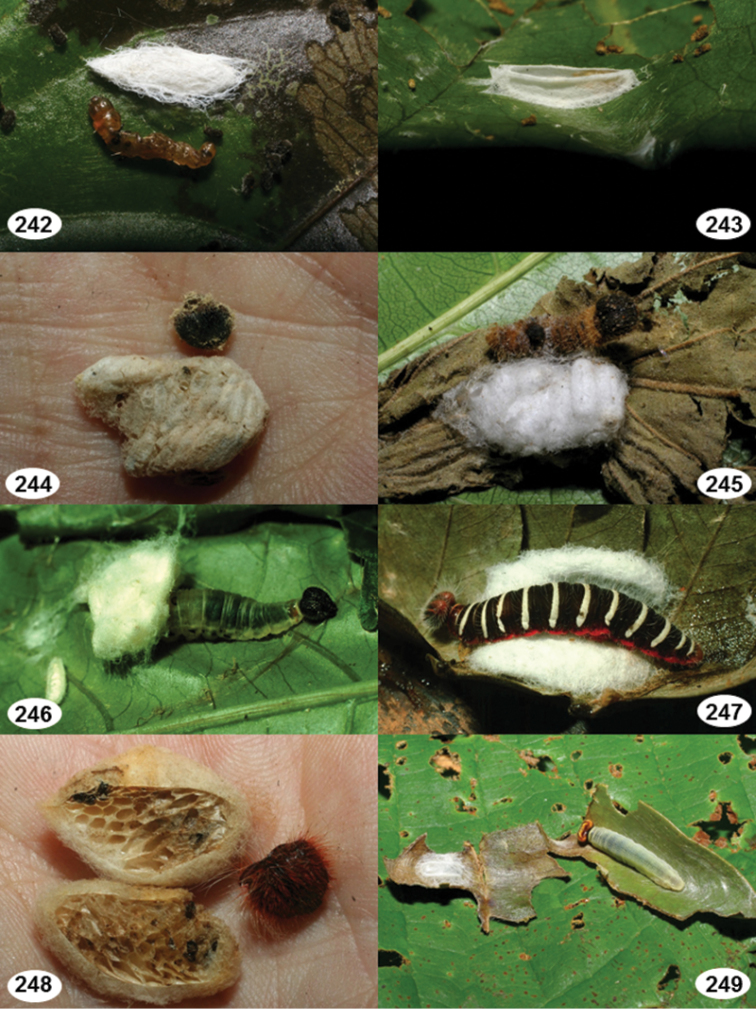
Cocoons of *Apanteles* species of Area de Conservación de Guanacaste. **242** *Apanteles tiboshartae* **243** *Apanteles minornavarroi* **244** *Apanteles anabellecordobae* **245** *Apanteles carolinacanoae* **246** *Apanteles duniagarciae* **247** *Apanteles edwinapui* **248** *Apanteles eldarayae* **249** *Apanteles freddyquesadai*.

**Figures 250–257.**
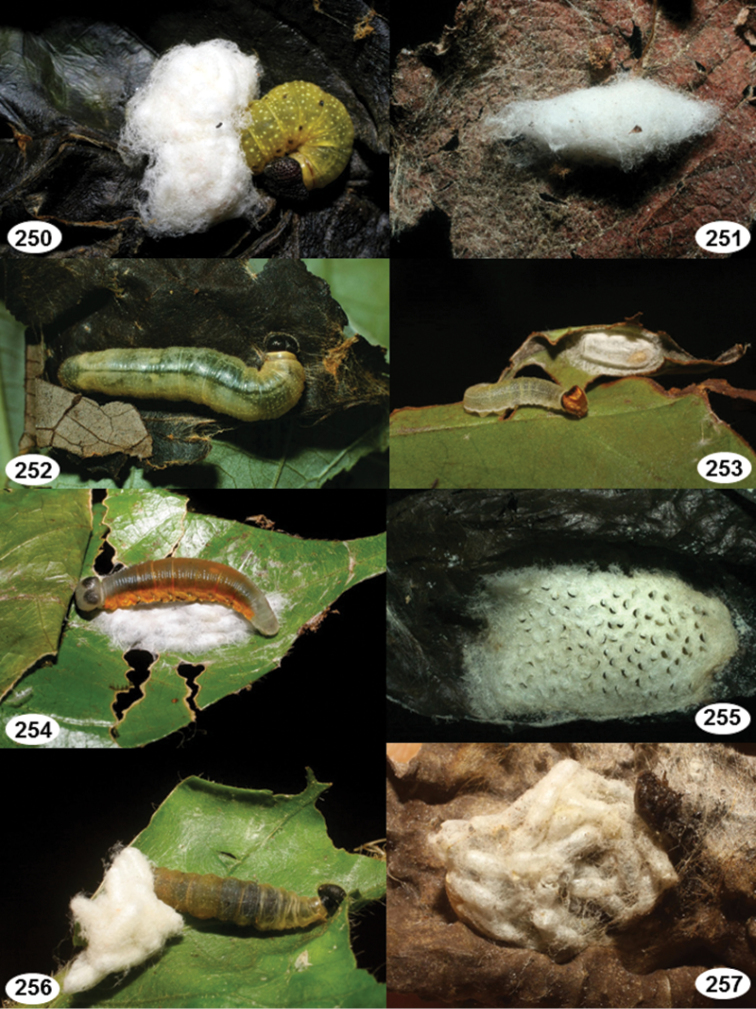
Cocoons of *Apanteles* species of Area de Conservación de Guanacaste. **250** *Apanteles guillermopereirai* **251** *Apanteles harryramirezi* **252** *Apanteles joseperezi* **253** *Apanteles luciariosae* **254** *Apanteles manuelpereirai* **255** *Apanteles osvaldoespinozai* **256** *Apanteles ruthfrancoae* **257** *Apanteles anapiedrae*.

**Figures 258–265.**
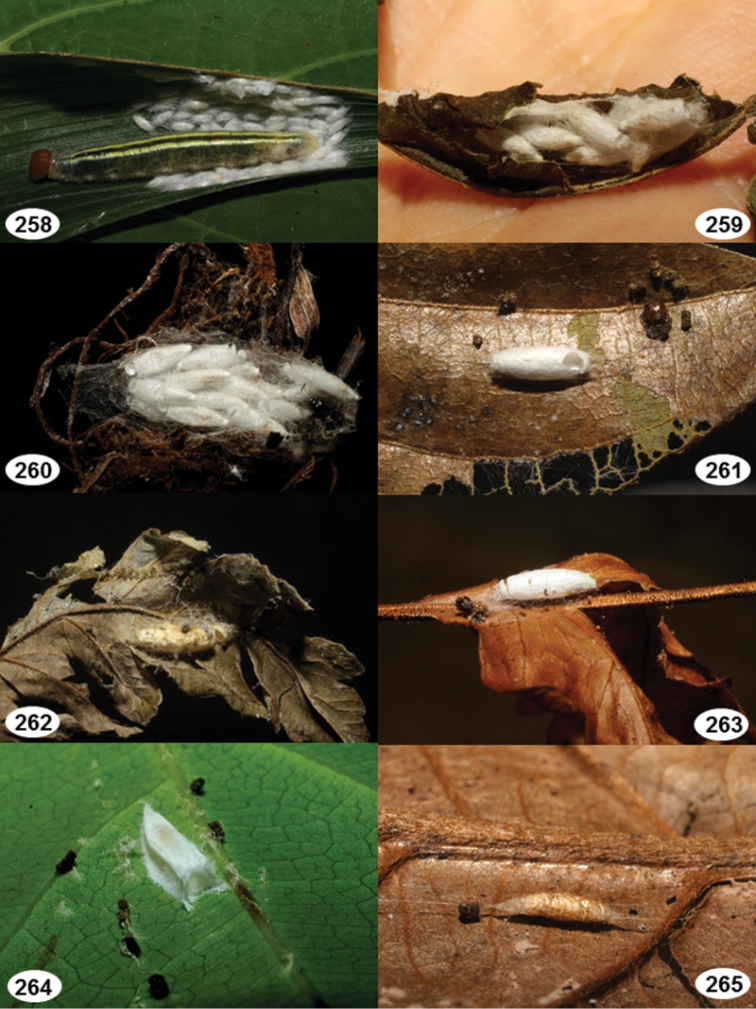
Cocoons of *Apanteles* species of Area de Conservación de Guanacaste. **258** *Apanteles andreacalvoae* **259** *Apanteles arielopezi* **260** *Apanteles mauriciogurdiani* **261** *Apanteles diegoalpizari* **262** *Apanteles javiersihezari* **263** *Apanteles raulacevedoi* **264** *Apanteles bienvenidachavarriae* **265** *Apanteles marisolarroyoae*.

**Figures 266–273.**
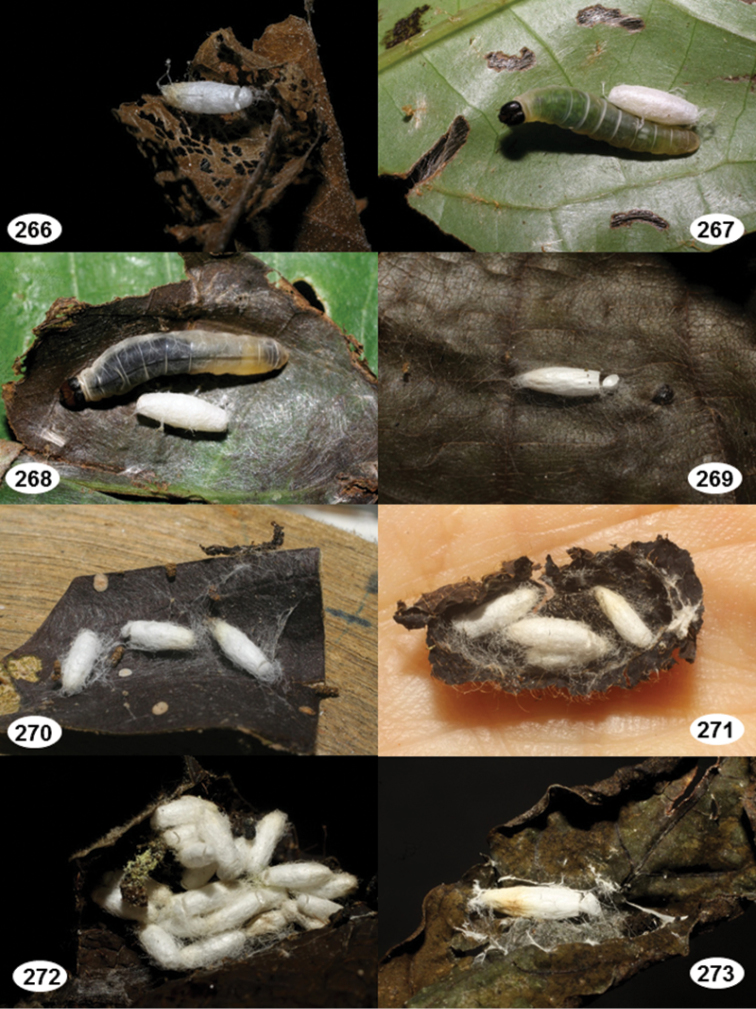
Cocoons of *Apanteles* species of Area de Conservación de Guanacaste. **266** *Apanteles calixtomoragai* **267** *Apanteles manuelriosi* **268** *Apanteles petronariosae* **269** *Apanteles carlosguadamuzi* **270** *Apanteles cinthiabarrantesae* **271** *Apanteles edithlopezae* **272** *Apanteles javiercontrerasi* **273** *Apanteles jesusbrenesi*.

**Figures 274–281.**
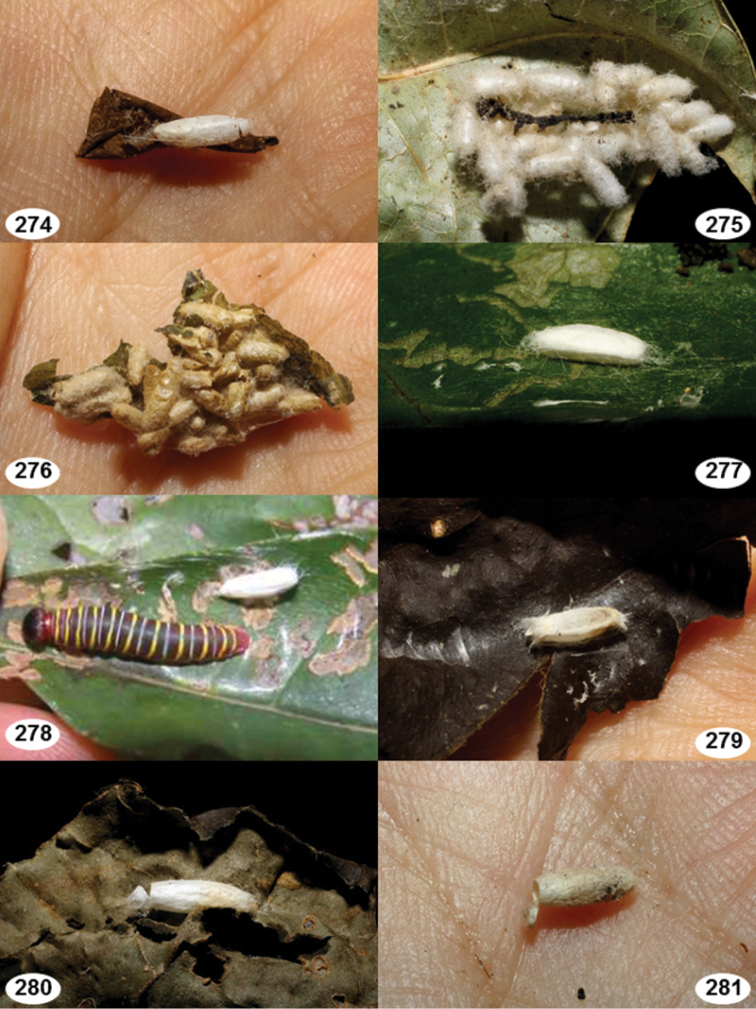
Cocoons of *Apanteles* species of Area de Conservación de Guanacaste. **274** *Apanteles williamcamposi* **275** *Apanteles carlosrodriguezi* **276** *Apanteles gloriasihezarae* **277** *Apanteles christianzunigai* **278** *Apanteles diegotorresi* **279** *Apanteles erickduartei* **280** *Apanteles felixcarmonai* **281** *Apanteles luishernandezi*.

**Figures 282–289.**
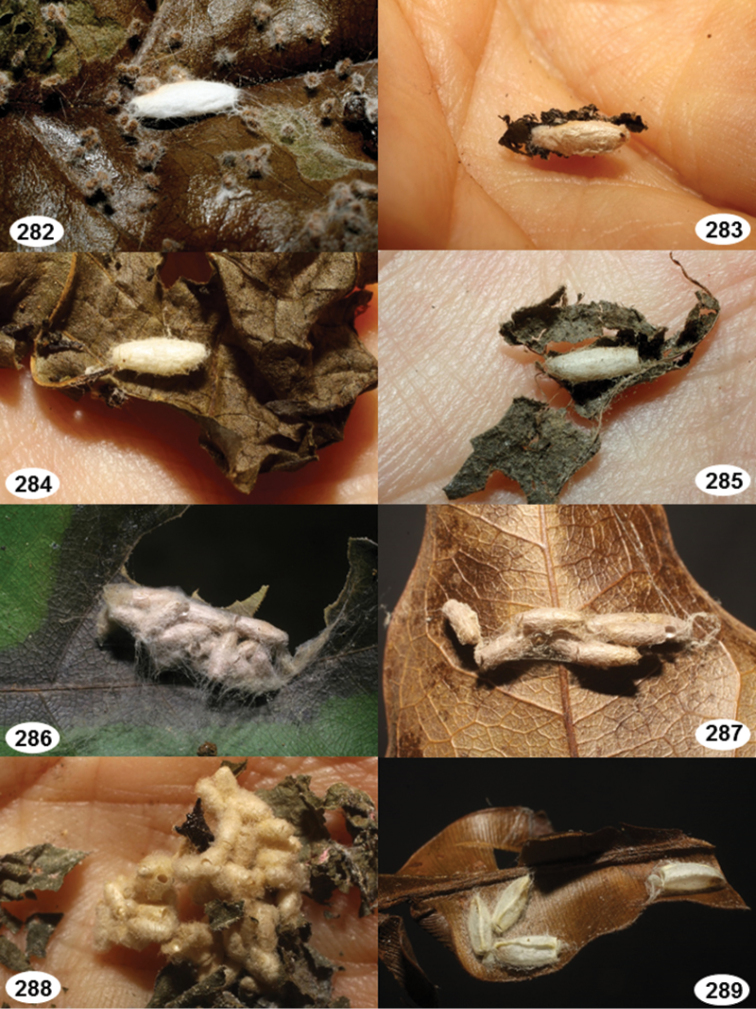
Cocoons of *Apanteles* species of Area de Conservación de Guanacaste. **282** *Apanteles milenagutierrezae* **283** *Apanteles ronaldcastroi* **284** *Apanteles flormoralesae* **285** *Apanteles garygibsoni* **286** *Apanteles glenriverai* **287** *Apanteles pablovasquezi* **288** *Apanteles marcobustosi* **289** *Apanteles hectorsolisi*.

**Figures 290–297.**
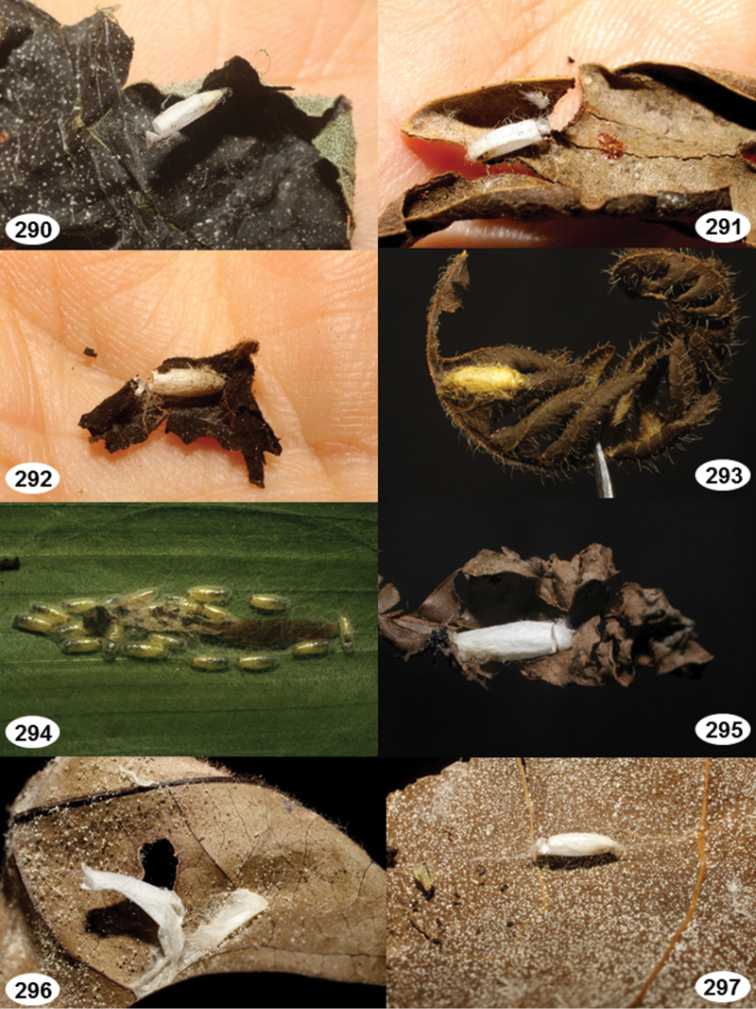
Cocoons of *Apanteles* species of Area de Conservación de Guanacaste. **290** *Apanteles isidrovillegasi* **291** *Apanteles joserasi* **292** *Apanteles juanhernandezi* **293** *Apanteles keineraragoni* **294** *Apanteles leonelgarayi* **295** *Apanteles luisgaritai* **296** *Apanteles marialuisariasae* **297** *Apanteles marisolnavarroae*.

**Figures 298–305.**
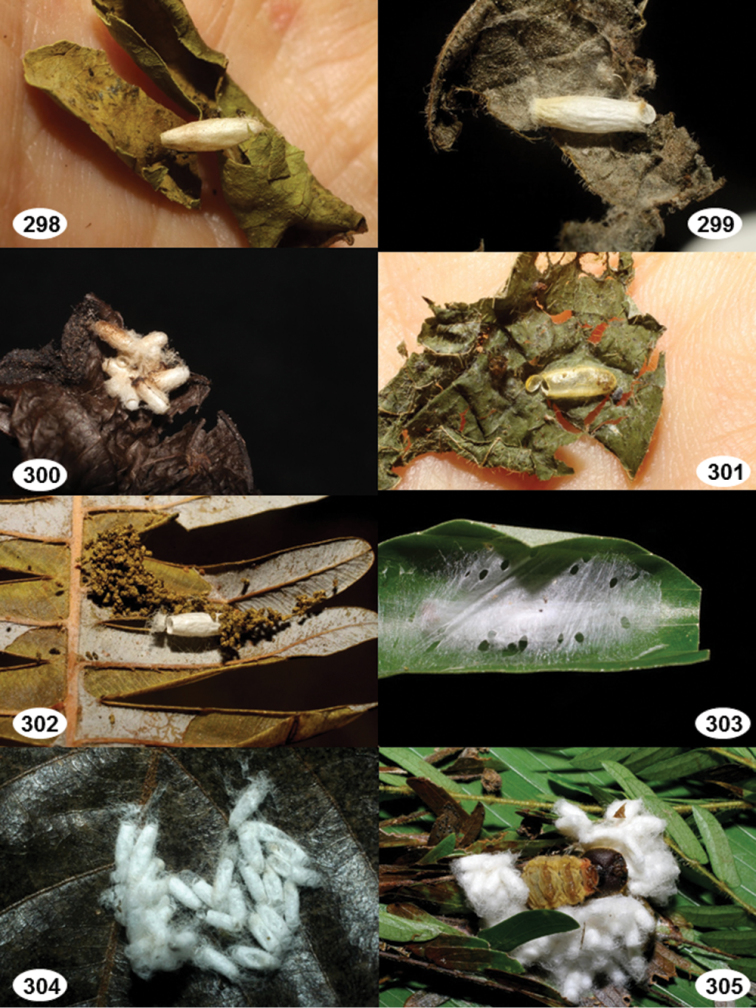
Cocoons of *Apanteles* species of Area de Conservación de Guanacaste. **298** *Apanteles randallmartinezi* **299** *Apanteles monicachavarriae* **300** *Apanteles rolandovegai* **301** *Apanteles ronaldgutierrezi* **302** *Apanteles rosibelelizondoae* **303** *Apanteles sergiocascantei* **304** *Apanteles alvarougaldei* **305** *Apanteles angelsolisi*.

**Figures 306–313.**
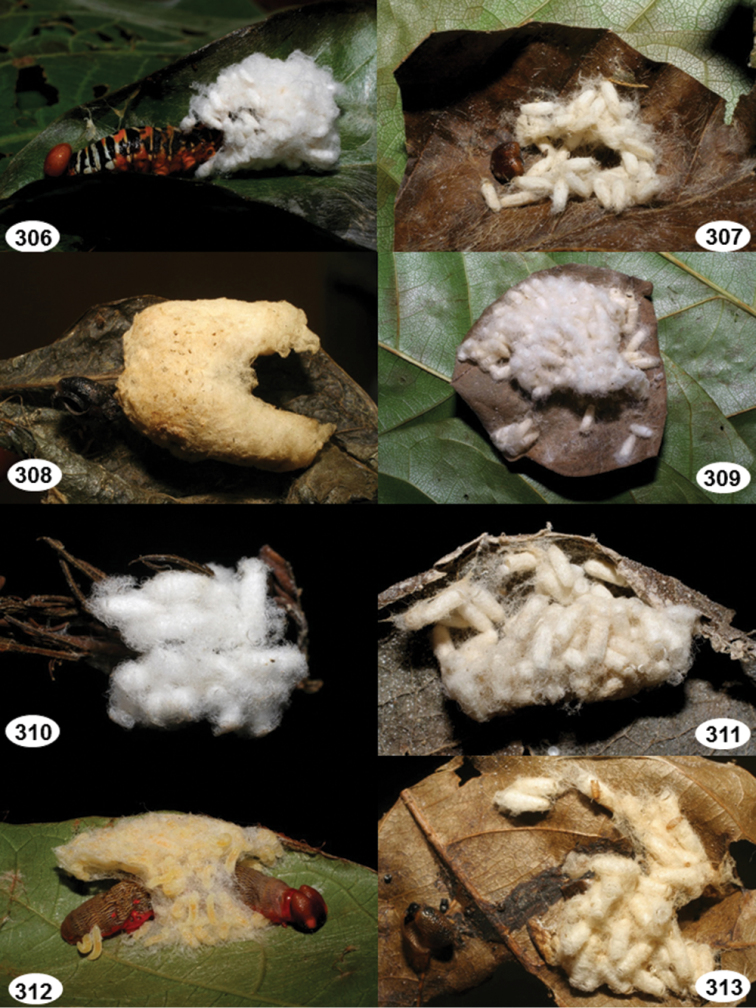
Cocoons of *Apanteles* species of Area de Conservación de Guanacaste. **306** *Apanteles bernardoespinozai* **307** *Apanteles carlosviquezi* **308** *Apanteles ciriloumanai* **309** *Apanteles diniamartinezae* **310** *Apanteles eliethcantillanoae* **311** *Apanteles gladysrojasae* **312** *Apanteles hazelcambroneroae* **313** *Apanteles inesolisae*.

**Figures 314–321.**
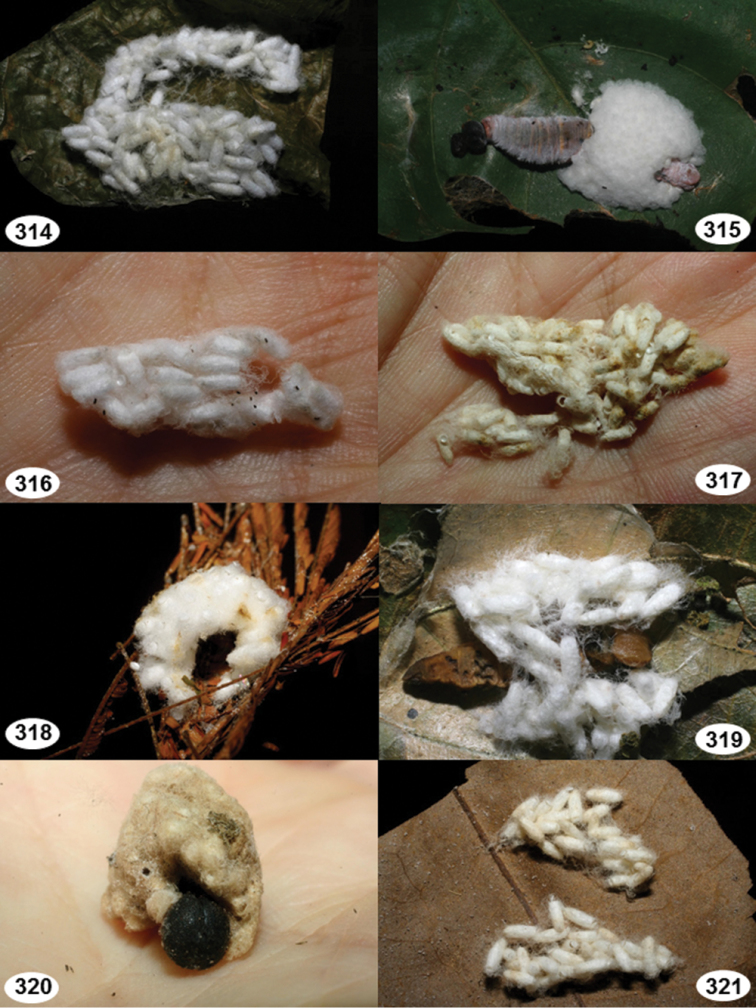
Cocoons of *Apanteles* species of Area de Conservación de Guanacaste. **314** *Apanteles jesusugaldei* **315** *Apanteles josecortesi* **316** *Apanteles josemonteroi* **317** *Apanteles juanmatai* **318** *Apanteles lilliammenae* **319** *Apanteles manuelzumbadoi* **320** *Apanteles mariachavarriae* **321** *Apanteles minorcarmonai*.

**Figures 322–330.**
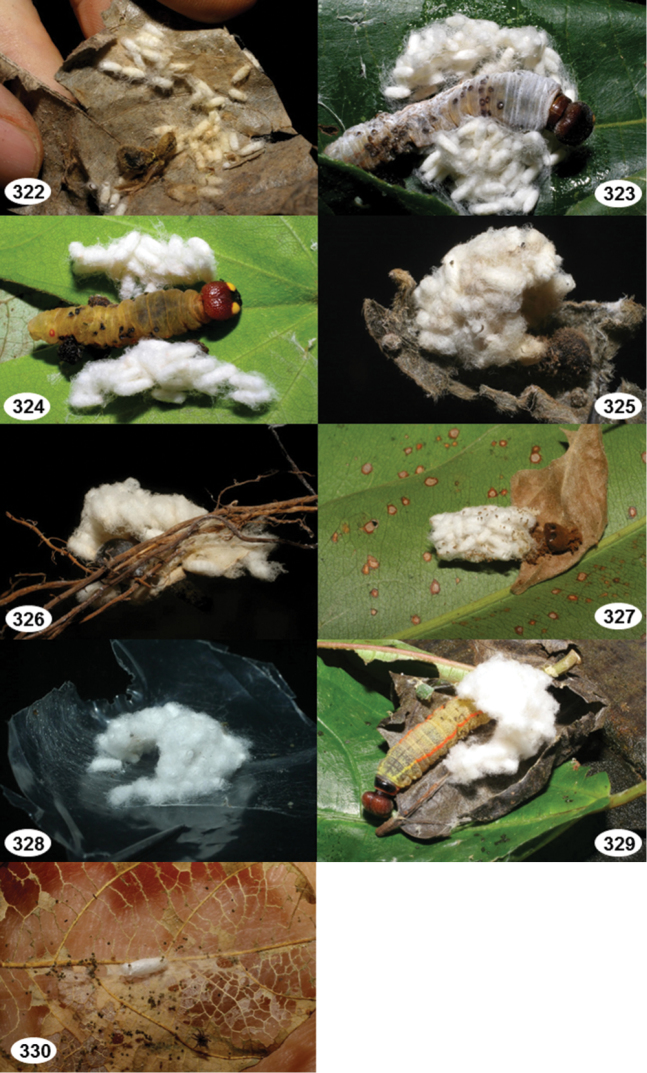
Cocoons of *Apanteles* species of Area de Conservación de Guanacaste. **322** *Apanteles pabloumanai* **323** *Apanteles randallgarciai* **324** *Apanteles ricardocaleroi* **325** *Apanteles sergioriosi* **326** *Apanteles wadyobandoi* **327** *Apanteles federicomatarritai* **328** *Apanteles robertoespinozai* **329** *Apanteles jorgehernandezi* **330** *Apanteles rostermoragai*.

## Appendix 1

Details of morphological terms and measurements used in the paper. (doi: 10.3897/zookeys.383.6418.app1) File format: Microsoft Word file (doc).

**Explanation note:** This Appendix contains two sections: A table detailing all morphological terms and measurements used in this paper; followed by a discussion of some characters that are prone to variable results when measuring.

## Appendix 2

DNA barcodes for all ACG inventory Microgastrinae. (doi: 10.3897/zookeys.383.6418.app2) File format: Microsoft Excel file (xlsx).

**Explanation note:** DNA barcodes for all ACG inventory Microgastrinae were obtained using DNA extracts prepared from single legs using a glass fibre protocol (Ivanova et al. 2006).
