# Supplementary material for: Review of Apanteles sensu stricto (Hymenoptera, Braconidae, Microgastrinae) from Area de Conservación Guanacaste, northwestern Costa Rica, with keys to all described species from Mesoamerica
Source: Zookeys. 2014 Feb 24;(383):1–565. doi: 10.3897/zookeys.383.6418 (PMC3950464; doi:10.3897/zookeys.383.6418)
Supplement: Supplementary file 1 — Details of morphological terms and measurements used in the paper. (doi: 10.3897/zookeys.383.6418.app1) File format: Microsoft Word file (doc). [file ZooKeys-383-001-s001.doc]

**Supplementary Appendix 1: Details of morphological terms and measurements used in the paper.**

This Appendix contains two sections: A table detailing all morphological terms and measurements used in this paper; followed by a discussion of some characters that are prone to variable results when measuring.

Morphological terms and measurements used throughtout the paper. “(**Fig. #)**” refers to the Figure number in this paper where the term/measurement is illustrated. “**Synonym term**” refers to synonym terms/measurements used in previous Microgastrinae papers. New terms and/or measuremts proposed for the first time in this paper are indicated as “NEW” in the URI column.

| **Term** | **Concept** | **URI (link to the Hymenoptera Anatomy and Ontology website)** | **Synonym term** |
| --- | --- | --- | --- |
| antenna | The anatomical structure that is composed of ringlike sclerites and the anatomical structures encircled by these sclerites and that is articulated with the cranium | <http://purl.obolibrary.org/obo/HAO_0000101> |  |
| antennal flagellomerus | The annulus that is located distally of the pedicel | <http://purl.obolibrary.org/obo/HAO_0000342> |  |
| antennal flagellomerus 2 | The flagellomerus that is located immediately distal to the first flagellomerus | <http://purl.obolibrary.org/obo/HAO_0001883> |  |
| antennal flagellomerus 2 length | The anatomical line that is the shortest between the distal and proximal margins of the second flagellomerus from dorsal view | NEW |  |
| antennal flagellomerus 2 width | The anatomical line that is the shortest among the lines extending between the lateral margins of the second flagellomeres from dorsal view | NEW |  |
| antennal flagellomerus 14 | The flagellomerus that is located immediately distal to flagellomerus 13 | <http://purl.obolibrary.org/obo/HAO_0002067> |  |
| antennal flagellomerus 14 length | The anatomical line that is the shortest between the distal and proximal margins of flagellomerus 14 from dorsal view | NEW |  |
| antennal flagellomerus 14 width | The anatomical line that is the shortest among the lines extending between the lateral margins of flagellomerus 14 from dorsal view | NEW |  |
| antenna length | The anatomical line that is median, extends between the proximalmost point of the scape and the distalmost point of the distalmost antennomere | NEW |  |
| anteromesoscutum | The area that is located anterior to the transscutal articulation | <http://purl.obolibrary.org/obo/HAO_0001490> | Mesoscutum |
| area | The anatomical structure that is delimited by material or immaterial anatomical entities | <http://purl.obolibrary.org/obo/HAO_0000146> |  |
| propodeum areola | The area on the propodeum that is median and is delimited anteriorly and laterally by carinae | <http://purl.obolibrary.org/obo/HAO_0002008> |  |
| basal tooth | The projection that is located on the distal half of the tarsal claw and proximal to the apex | <http://purl.obolibrary.org/obo/HAO_0001219> |  |
| body | The anatomical cluster that is composed of the whole organism but which excludes the antennae, legs and wings | <http://purl.obolibrary.org/obo/HAO_0000182> |  |
| body length | The anatomical line that is median and extends between the anteriormost point of the head and the posteriormost point of the metasoma | NEW |  |
| carina | The process that is linear and external | <http://purl.obolibrary.org/obo/HAO_0000188> |  |
| coxa | The leg segment that is connected to the body and to the trochanter via conjunctivae and muscles | <http://purl.obolibrary.org/obo/HAO_0000228> |  |
| eye | The compound organ that is composed of ommatidia | <http://purl.obolibrary.org/obo/HAO_0000217> |  |
| femora | The leg segment that is distal to the trochanter and proximal to the tibia | <http://purl.obolibrary.org/obo/HAO_0000327> |  |
| flagellomerus | The annulus that is located distally of the pedicel | <http://purl.obolibrary.org/obo/HAO_0000342> |  |
| flagellum | The anatomical cluster composed of flagellomeres | <http://purl.obolibrary.org/obo/HAO_0000343> |  |
| fore wing | The wing that is located on the mesothorax | <http://purl.obolibrary.org/obo/HAO_0000351> |  |
| fore wing base | The wing base that is located in the mesothorax | <http://purl.obolibrary.org/obo/HAO_0001747> |  |
| fore wing length | The anatomical line that extends between the median margin of the first axillary sclerite and the distalmost point of the wing blade | NEW **(Fig 208 a)** |  |
| fore wing vein | The wing vein that is on the fore wing | <http://purl.obolibrary.org/obo/HAO_0002066> |  |
| head | The tagma that is located anterior to the thorax | <http://purl.obolibrary.org/obo/HAO_0000397> |  |
| head width | The anatomical line that is the longest among the transverse lines extending between the lateral eye margins in frontal view | NEW |  |
| head height | The anatomical line that extends between the distalmost point of the mandible and the apex of the lateral ocellus in lateral view | NEW |  |
| humeral complex | The complex that is located on the fore wing | <http://purl.obolibrary.org/obo/HAO_0001524> |  |
| hypopygium | The abdominal sternum that is the posteriormost visible sclerite located ventrally in the abdomen | <http://purl.obolibrary.org/obo/HAO_0000410> |  |
| impression, impressions | The groove that does not correspond to a ridge. | <http://purl.obolibrary.org/obo/HAO_0000417> |  |
| interocellar distance | The anatomical line that is the shortest between the margin of the lateral ocelli. | <http://purl.obolibrary.org/obo/HAO_0000759> |  |
| lateral ocellar length | The anatomical line that is the shortest between the margins of the median and lateral ocelli. | <http://purl.obolibrary.org/obo/HAO_0000480> |  |
| lateral face of mesoscutellum | The area that is located laterally to the mesoscutellar disc and is comprised by the mesoscutellar trough and the mesoscutellar arm | NEW **(Fig 206 b)** |  |
| lateral face of mesoscutellum height | The anatomical line that is median and extends between the anteriormost and the posteriormost margin of the lateral face of scutellum | NEW **(Fig 206 b)** |  |
| laterotergites | The tergite that is located laterally on the abdominal tergum | <http://purl.obolibrary.org/obo/HAO_0000493> |  |
| laterotergites | The area that is located laterally on the tergum and is delimited by a longitudinal edge | <http://purl.obolibrary.org/obo/HAO_0001861> |  |
| margin, margins | The line that delimits the periphery of an area | <http://purl.obolibrary.org/obo/HAO_0000510> |  |
| margin, margins | The anatomical region that extends along the margin | <http://purl.obolibrary.org/obo/HAO_0001981> |  |
| mediotergite | The tergite that is located medially on the tergum | <http://purl.obolibrary.org/obo/HAO_0001783> |  |
| mediotergite | The area that is located medially on the tergum and is separated laterally by a line from the laterotergite | <http://purl.obolibrary.org/obo/HAO_0001860> |  |
| mediotergite 1 | The mediotergite that is located on abdominal tergum 1 | <http://purl.obolibrary.org/obo/HAO_0001463> |  |
| mesoscutellar disc | The area that is median, convex and is delimited anteriorly by the scutoscutellar sulcus and laterally by the axillar depression | <http://purl.obolibrary.org/obo/HAO_0000915> | Scutellum |
| mesoscutellar disc length | The anatomical line that is median and extends between the anterior and the posterior margin of the mesocutellar disc. | NEW | Scutellum length |
| mesoscutellar disc width | The line that is the shortest among the lines that extends between the lateral margins of the mesoscutellar disc and that are perpendicular to the median longitudinal line of the body. | NEW | Scutellum width |
| Mesoscutellum | The scutellum that is located on the mesonotum. | <http://purl.obolibrary.org/obo/HAO_0000574> |  |
| mesoscutellum lunula | The polished area in the lateral face of the mesoscutellum that extends from the mesoscutellar arm to the mesoscutellar trough | NEW **(Fig 206 b)** |  |
| mesoscutellum lunula height | The anatomical line that is median and extends between the anterior and the posterior margin of the mesoscutellum lunula | NEW **(Fig 206 b)** |  |
| mesosoma | The anatomical cluster that is composed of the prothorax, mesothorax and the metapectal-propodeal complex | <http://purl.obolibrary.org/obo/HAO_0000576> |  |
| metabasitarsus | The basitarsus that is located on the hind leg | <http://purl.obolibrary.org/obo/HAO_0001142> |  |
| metabasitarsus length | The anatomical line that is the shortes among the lines extending between the proximal margin and the distalmost point of the metabasitarsus | NEW **(Fig 209 b)** |  |
| metacoxa | The coxa that is located on the hind leg | <http://purl.obolibrary.org/obo/HAO_0000587> |  |
| metafemur | The femur that is located on the hind leg | <http://purl.obolibrary.org/obo/HAO_0001140> |  |
| metafemur length | The anatomical line that is the shortes among the lines extending between the proximal and distal margins of the metafemur | NEW **(Fig 209 b)** |  |
| metafemur width | The anatomical line that is the shortest among the lines extending between the lateral margins of the metafemur from posterior (?) view | NEW **(Fig 209 b)** |  |
| metasoma | The tagma that is connected anteriorly to the metapectal-propodeal complex at the propodeal foramen and consists of abdominal segments | <http://purl.obolibrary.org/obo/HAO_0000626> |  |
| metatibia | The tibia that is located on the hind leg | <http://purl.obolibrary.org/obo/HAO_0000631> |  |
| metatibia inner spur | The spur on metatibia that is closer to the body -when the leg is facing towards the apex of metasoma | NEW **(Fig 209 b)** |  |
| metatibia inner spur length | The anatomical line that is the shortes among the lines extending between the proximal margin and the distalmost point of the metatibial inner spur | NEW **(Fig 209 b)** |  |
| metatibia outer spur | The spur on metatibia that is farther from the body -when the leg is facing towards the apex of metasoma | NEW **(Fig 209 b)** |  |
| metatibia outer spur length | The anatomical line that is the shortes among the lines extending between the proximal margin and the distalmost point of the metatibial outer spur | NEW **(Fig 209 b)** |  |
| nucha | The area that is raised and surrounds the propodeal foramen | <http://purl.obolibrary.org/obo/HAO_0000651> |  |
| ocellus | The multi-tissue structure that is located on the top of the head, composed of the corneal lens, pigment cell, rhabdoms and synaptic plexus | <http://purl.obolibrary.org/obo/HAO_0000661> |  |
| ocular-ocellar line | The anatomical line that is shortest and connects the compound eye and the lateral ocellus | <http://purl.obolibrary.org/obo/HAO_0000662> |  |
| ovipositor | The anatomical cluster that is composed of the first valvulae, second valvulae, third valvulae, first valvifers and second valvifers | <http://purl.obolibrary.org/obo/HAO_0000679> |  |
| ovipositor sheaths | The area that is located posterior to the second valvifer and is connected to the second valvifer via conjuntiva | <http://purl.obolibrary.org/obo/HAO_0001012> |  |
| ovipositor sheaths | The anatomical cluster that is composed of the third valvulae | <http://purl.obolibrary.org/obo/HAO_0000680> |  |
| pedicel | The antennal segment that is the second segment of the antenna and is connected proximally with the scape and distally with the flagellum | <http://purl.obolibrary.org/obo/HAO_0000706> |  |
| pits in scutoscutellar sulcus | The impression that is located in th scutoscutellar sulcus. | NEW **(Fig 206 b)** |  |
| posterior ocellus | The ocellus that is paired | <http://purl.obolibrary.org/obo/HAO_0000481> |  |
| posterior ocellus diameter | The anatomical line that is the longest among the lines extending between two points of the eye margin | NEW **(Fig 206 a)** |  |
| propodeum | The area that is located posterior to the metapleural carina | <http://purl.obolibrary.org/obo/HAO_0001249> |  |
| pterostigma | The patch on the wing that is sclerotized and is on the anterior margin of the fore wing | <http://purl.obolibrary.org/obo/HAO_0000957> |  |
| punctures | The depression that is point-like, without a flat bottom, and occurs in areas of punctate surface sculpture | <http://purl.obolibrary.org/obo/HAO_0000885> |  |
| scape | The antennal segment that is proximal to the pedicel and is connected with the head via the radicle | <http://purl.obolibrary.org/obo/HAO_0000908> |  |
| sculpture | The area that is located on the sclerite and that is composed of repetitive anatomical structures | <http://purl.obolibrary.org/obo/HAO_0000913> |  |
| scutellar disc | The area that is median, convex and is delimited anteriorly by the scutoscutellar sulcus and laterally by the axillar depression | <http://purl.obolibrary.org/obo/HAO_0000915> |  |
| scutoscutellar sulcus | The sulcus that extends along the scutoscutellar suture | <http://purl.obolibrary.org/obo/HAO_0000919> |  |
| seta, setae | The sensillum that is multicellular and consists of trichogen, tormogen, and sense cells and the cuticle secreted by and adjacent with the trichogen cell | <http://purl.obolibrary.org/obo/HAO_0000935> | hairs |
| spiracle | The anatomical cluster that is composed of the distal end of the trachea and the margin of the sclerite or conjunctiva surrounding the spiracular opening | <http://purl.obolibrary.org/obo/HAO_0000950> |  |
| sternites | The sternite that is located on the abdominal sternum | <http://purl.obolibrary.org/obo/HAO_0002065> |  |
| stigma | The patch on the wing that is sclerotized and is on the anterior margin of the fore wing | <http://purl.obolibrary.org/obo/HAO_0000957> |  |
| tarsal claws | The spur that is curved and projects from the apex of the last tarsal segment on either side of the arolium of the pretarsus | <http://purl.obolibrary.org/obo/HAO_0000989> |  |
| tegula | The sclerite that is located laterally of the preaxilla and obscures the anterior mesonoto-first axillary articulation and the mesopleuro-second axillary sclerite joints | <http://purl.obolibrary.org/obo/HAO_0000993> |  |
| tibiae | The leg segment that is proximal to the tarsus and distal to the femur | <http://purl.obolibrary.org/obo/HAO_0001017> |  |
| vein, veins | The area that is linear and sclerotised and acts as support for the wing membrane | <http://purl.obolibrary.org/obo/HAO_0001095> |  |
| wing vein, wing veins | The area that is linear and sclerotised and acts as support for the wing membrane | <http://purl.obolibrary.org/obo/HAO_0001095> |  |

Characters that are prone to variable results when measuring:

1 - Length of first mediotergite (T1). Two reasons contribute to make measuring the length of T1 difficult. First, unless the metasoma is completely detached from the mesosoma -or can be moved away from the mesosoma in specimens with a relative flexible body (e.g., those prepared by critical point drying), it is usually difficult to visualize the exact starting point of T1. Second, the shape of T1 in many species is arched – a feature that can be better observed in lateral view, so estimating the length of T1 in dorsal view implies some inaccuracy. Opting to measure the length of T1 in lateral view instead of dorsally can also be challenging, as often the laterotergites tend to obscure the view of the mediotergite. In this paper we keep the “traditional” way of measuring the length of T1, i.e., in dorsal view and without detaching the metasoma because that will be the best alternative for most users when dealing with their own specimens. However, we estimate that the variation when measuring T1 length that way can amount to 10% error. As a result, whenever comparing two specimens and/or species with T1 length differing by less than 10% other characters should ideally also be examined and compared. We have tried to provide for that in the key as much as possible. [Add caption to Figure that illustrates how we measure T1 length].

2 - Length of the ovipositor sheaths. This is perhaps the character that is most error-prone when measuring. An obvious problem is when the sheaths are curved or twisted to some degree. There have been some previous efforts (summarized in Aguiar, 2005) to measure the ovipositor itself but, as the author comments, that method cannot be applied to the sheaths but just the ovipositor. Another problem is the definition of the starting point of the sheaths. It has been suggested to use the area covered by setae (Reference?) but that is not always clearly delimited. A better approach could be to start at the basal constriction of the sheaths (Fig.), but even there it is not always possible to define the starting point unambiguously. We estimate that variation (between different users) when measuring the length of ovipositor sheaths can amount to as much as 15%.

3 - Fore wing veins and pterostigma measurements. Limits of the pterostigma, especially apically, are subjective. As for veins, the end points should be clearly established. Here we follow the same approach as Valerio and Whitifield (200X, Figure X). Even so, spectral veins (*sensu* Wharton et al. 1997), pale coloured or transparent veins may be difficult to assess unambigously. Additionally, the wing is often curved to some degree, i.e., it is rarely flat in a mounted specimen. This problem can be fixed by removing the wing and preparing a slide mount, but often that will not be possible, allowed (depending on collection regulations), or even desired. Alternatively, it is always possible to check for the endpoints of a measurement to be at the same depth of field (if so, there is no need to adjust the microscope when moving from one point to the other).

4 - Length of metatibia. Though a relatively easy and accurate measurements to take, one needs to pay attention to what is chosen as the end of the metatibia. In our work we have chosen to use the maximum length of the metatibia (Fig. X).
